# Frontotemporal dementia: insights into the biological underpinnings of disease through gene co-expression network analysis

**DOI:** 10.1186/s13024-016-0085-4

**Published:** 2016-02-24

**Authors:** Raffaele Ferrari, Paola Forabosco, Jana Vandrovcova, Juan A. Botía, Sebastian Guelfi, Jason D. Warren, UK Brain Expression Consortium (UKBEC), Parastoo Momeni, Michael E. Weale, Mina Ryten, John Hardy

**Affiliations:** Department of Molecular Neuroscience, Institute of Neurology, University College London, Russell Square House, 9-12 Russell Square House, London, WC1N 3BG UK; Istituto di Ricerca Genetica e Biomedica, Cittadella Universitaria di Cagliari, 09042 Monserrato, Sardinia Italy; King’s College London, Department of Medical & Molecular Genetics, Guy’s Hospital, London, SE1 9RT UK; Dementia Research Centre, UCL Institute of Neurology, University College London, London, WC1N 3BG UK; Omixy, 107 Cheapside, EC2V 6DN London, UK

**Keywords:** Frontotemporal dementia, Gene expression, Co-expression, Network, Systems biology, DNA protection, Immune system, Protein catabolism

## Abstract

**Background:**

In frontotemporal dementia (FTD) there is a critical lack in the understanding of biological and molecular mechanisms involved in disease pathogenesis. The heterogeneous genetic features associated with FTD suggest that multiple disease-mechanisms are likely to contribute to the development of this neurodegenerative condition.

We here present a systems biology approach with the scope of i) shedding light on the biological processes potentially implicated in the pathogenesis of FTD and ii) identifying novel potential risk factors for FTD. We performed a gene co-expression network analysis of microarray expression data from 101 individuals without neurodegenerative diseases to explore regional-specific co-expression patterns in the frontal and temporal cortices for 12 genes (*MAPT*, *GRN*, *CHMP2B*, *CTSC*, *HLA-DRA*, *TMEM106B*, *C9orf72*, *VCP*, *UBQLN2*, *OPTN*, *TARDBP* and *FUS*) associated with FTD and we then carried out gene set enrichment and pathway analyses, and investigated known protein-protein interactors (PPIs) of FTD-genes products.

**Results:**

Gene co-expression networks revealed that several FTD-genes (such as *MAPT* and *GRN*, *CTSC* and *HLA-DRA*, *TMEM106B*, and *C9orf72*, *VCP*, *UBQLN2* and *OPTN*) were clustering in modules of relevance in the frontal and temporal cortices. Functional annotation and pathway analyses of such modules indicated enrichment for: i) DNA metabolism, i.e. transcription regulation, DNA protection and chromatin remodelling (*MAPT* and *GRN* modules); ii) immune and lysosomal processes (*CTSC* and *HLA-DRA* modules), and; iii) protein meta/catabolism (*C9orf72*, *VCP*, *UBQLN2* and *OPTN*, and *TMEM106B* modules). PPI analysis supported the results of the functional annotation and pathway analyses.

**Conclusions:**

This work further characterizes known FTD-genes and elaborates on their biological relevance to disease: not only do we indicate likely impacted regional-specific biological processes driven by FTD-genes containing modules, but also do we suggest novel potential risk factors among the FTD-genes interactors as targets for further mechanistic characterization in hypothesis driven cell biology work.

**Electronic supplementary material:**

The online version of this article (doi:10.1186/s13024-016-0085-4) contains supplementary material, which is available to authorized users.

## Background

Frontotemporal dementia (FTD) is the second most common early onset form of dementia after Alzheimer’s disease (AD) [1]. Its main clinical presentations (behavioural or language variants) directly reflect atrophy patterns in the frontal or temporal lobes [2]. Sub-cortical regions are also implicated [3–5] as damage to white matter (uncinated fasciculus, cingulum bundle and corpus callosum) [6, 7] and deep grey matter structures (putamen, insula, thalamus and hippocampus) have been recently reported [8, 9].

The majority of cases show either tau (FTLD-tau) or ubiquitin/TDP-43 (FTLD-TDP) inclusions (≤40–50 %), whereas a minority has FUS (≤10 %; FTLD-FUS) or ubiquitin/p62 (≤1–2 %; FTLD-UPS) inclusions [10]. Some (but not all) FTLD-tau cases carry mutations in the microtubule associated protein tau (*MAPT*) [11], whilst FTLD-TDP cases are almost never associated with variability in the TAR-DNA binding protein 43 (*TARDBP*), rather, in progranulin (*GRN*) and the chromosome 9 open reading frame 72 (*C9orf72*) genes [3]. Furthermore, the FTLD-FUS cases do not have a clear genetic component, whilst the rare FTLD-UPS cases have been associated with variability in the charged multivesicular body protein 2B (*CHMP2B*) gene [3]. These features suggest that there is no unidirectional relationship between the genetics and the molecular pathology of FTD and that the observed pathological signatures result from complex molecular mechanisms.

To date a handful of genes has been associated with FTD: besides *MAPT*, *GRN* and *C9orf72* (found in 2–11 %, 5–11 % and 7–20 % of cases, respectively) [11, 12], genetic variability in other genes, including *CHMP2B*, valosin containing protein (*VCP*), sequestosome 1 (*SQSTM1*) and ubiquilin 2 (*UBQLN2*), is extremely rare [3, 11]. Furthermore, pathogenic variants in *TARDBP* and the fused in sarcoma (*FUS*) genes seem nearly absent in FTD comparatively to ALS or ALS-FTD cases [13]; however, given that TARDBP and FUS are pathological hallmarks of FTLD subtypes [10], they likely hold functional relevance in the pathogenesis of FTD. Finally, recent genome wide association studies (GWAS) revealed association with FTD for the modifying factor transmembrane protein 106B (*TMEM106B*) [14, 15] and two further loci, one containing the RAB38, member RAS oncogene family (*RAB38*) and catepsin C (*CTSC*) genes, and one pointing to the *HLA*-locus [16].

In this study we used a systems biology approach based on gene co-expression network analysis of microarray expression data generated from 101 individuals without neurodegenerative diseases (UK Human Brain Expression Consortium [UKBEC]) [17] to further investigate genes and loci associated with FTD and, particularly, to: i) evaluate their co-expression patterns in brain areas known to be affected in FTD; ii) annotate and highlight biological processes potentially implicated in disease mechanisms, and; iii) identify novel potential risk factors for FTD.

## Results

We grouped the FTD-genes into two categories: the pure (= mainly or exclusively associated with FTD) and the spectrum (= associated with more than one condition) genes (Table 1). We evaluated gene expression levels across different brain regions (frontal and temporal cortices as well as putamen, thalamus, hippocampus, white matter, cerebellum and medulla; see Methods section for details), assessed co-expression profiles and performed functional annotation and pathway analyses for the relevant modules (= we defined ‘relevant modules’ those modules containing one or more FTD-genes with hub status [1-q < 0.1] and/or module membership [MM] values > 0.5; see Methods section for details). Finally, we investigated whether the observed gene clustering is supported by known protein-protein interactors (PPIs) of FTD-genes products to infer the potential extent of the translation of the regional-specific co-expression patterns into the protein domain.

**Table 1:** FTD-genes analysed in this study

	Genes	Type of variant	Clinical phenotype	Pathology	Type of association	Ref
Pure FTD-genes	*MAPT*	exonic/intronic point mutations	bvFTD; FTD-17	FTLD-Tau	mendelian	3, 11
		small/large indels				
		missense				
	*GRN*	non-sense	bvFTD; PNFA	FTLD-TDP		3, 11
		missense				
		frame-shift				
		large deletions				
	*CHMP2B*	non-sense	FTD3	FTLD-UPS		3, 11
		missense				
	*RAB38*	unknown	bvFTD	unknown	from GWAS	16
	*CTSC*	unknown	bvFTD	unknown		
	*BTNL2*	unknown	bvFTD; PNFA; SD	unknown		
	*HLA-DRA*	unknown	bvFTD; PNFA; SD	unknown		
	*HLA-DRB5*	unknown	bvFTD; PNFA; SD	unknown		
	*TMEM106B*	unknown	FTD	FTLD-TDP		14
Spectrum FTD-genes	*C9orf72*	expansion	ALS; ALS-FTD	FTLD-TDP	mendelian	12, 13
	*VCP*	missense	IBMPFD; ALS; FTD	FTLD-TDP		3, 11, 12
	*SQSTM1*	non-sense	PBD; ALS; FTD	FTLD-TDP		3, 11, 12
		missense				
	*UBQLN2*	non-sense	MS; ALS; FTD	FTLD-TDP		
		missense				
	*OPTN*	non-sense	PDB; ALS-FTD	FTLD-TDP		
		missense				
	*TDP-43*	missense	ALS; ALS-FTD	FTLD-TDP		
	*FUS*	missense	ALS; ALS-FTD	FTLD-FUS		
		frame-shift				
		indel				

The FTD-genes used in our analysis were divided into two groups: the pure and the spectrum genes. For each gene, the associated genetic variability, as well as the associated clinical and pathological features are summarized

*bvFTD* behavioural variant FTD, *FTD-17* frontotemporal dementia linked to chromosome 17, *PNFA* progressive non-fluent aphasia, *FTD3* frontotemporal dementia linked to chromosome 3, *SD* semantic dementia, *ALS* amyotrophic lateral sclerosis, *IBMPFD* Inclusion body myopathy with early-onset Paget disease and frontotemporal dementia, *PBD* Paget’s disease of bone, *MS* multiple sclerosis

### Expression levels in brain

**Fig. 1:**
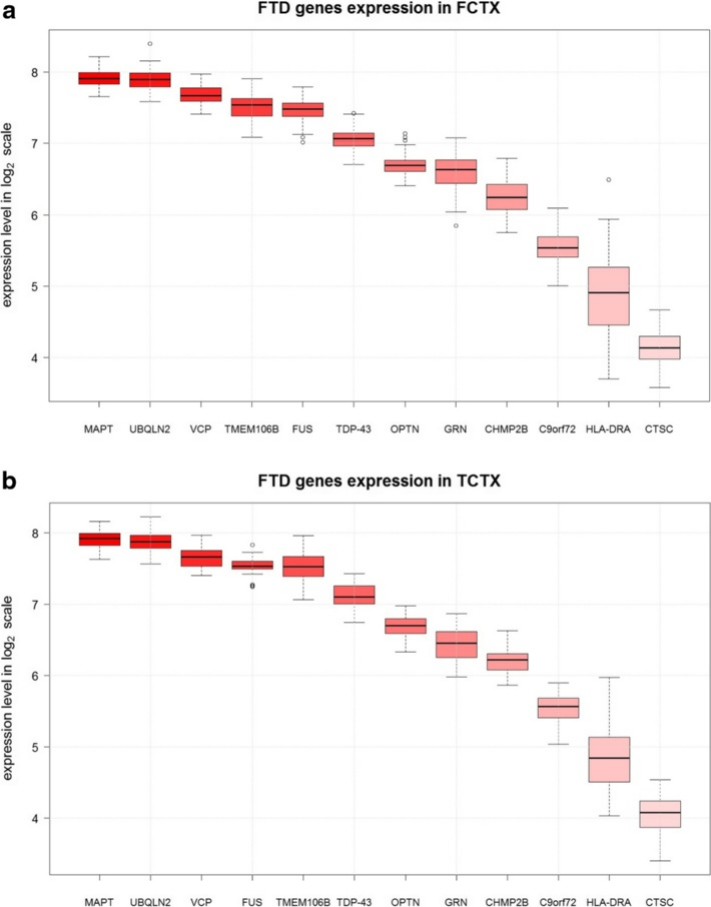
Expression levels. Expression levels of the FTD-genes considered in the network analysis in frontal cortex (**a**) and temporal cortex (**b**). *FUS* here was represented by Affymetrix transcript ID 3656904

To gain insight into the expression profile of FTD-genes in brain we searched for expression levels and patterns online and in our own repositories (Human Brain Atlas and Braineac, respectively; see Methods section for further details). The general, and cross-supportive, outcome was that all FTD-genes are indeed expressed in brain: for the pure FTD-genes, *MAPT*, *GRN*, *CHMP2B*, *CTSC*, *HLA-DRA* and *TMEM106B* showed moderate to high expression levels across brain tissues throughout the lifespan of an individual, whilst, comparatively, expression levels for *RAB38*, *BTNL2* and *HLA-DRB5* appeared to be lower. All spectrum FTD-genes, *C9orf72*, *VCP*, *SQSTM1*, *UBQLN2*, *OPTN*, *TARDBP* and *FUS* had high expression levels across brain tissues during development and aging, and *C9orf72*, *TARDBP* and *FUS* showed exceptionally high levels in the cerebellum. A focused assessment of the expression levels and patterns in frontal and temporal cortices revealed that *MAPT*, *UBQLN2*, *VCP*, *TMEM106B*, *FUS*, *TARDBP*, *OPTN*, *GRN* and *CHMP2B* had high expression rates, whilst these were comparatively lower for *C9orf72*, *HLA-DRA* and *CTSC* (Fig. 1a-b).

For a more detailed description and visualization of all genes’ expression patterns across brain tissues see the Additional file 1 (pp 1–4) and Additional file 1: Figures S1-S16 (pp 8–23).

### Weighted gene co-expression network analysis (WGCNA)

We performed the WGCNA with a primary focus on frontal and temporal cortices, the classically affected brain areas in FTD. Nevertheless, due to their recent increasing relevance to disease, we also extended the analysis to other brain regions such as putamen, thalamus, hippocampus and white matter for both the pure and spectrum genes, and the cerebellum and medulla for the spectrum genes only. Assessments on frontal and temporal cortices are presented hereafter and summarized in Table 2, whilst evaluations on any other brain region are shown in the Additional file 1 (pp 8–9).

**Table 2:** Module assignments for FTD-genes in frontal and temporal cortex

FTD genes	Affymetrix ID	type	Frontal cortex				Temporal cortex			
			module	Sz (n)	MM	1-q	module	Sz (n)	MM	1-q
***MAPT***	3723687	**pure**	**black**	791	**0.83**	**0.03**	lightyellow	210	**0.67**	0.31
***GRN***	3722917				**0.66**	0.34	cyan	276	0.56	0.51
*CHMP2B*	2631845		darkolivegreen	63	**0.76**	0.48	green	1439	0.53	0.49
***HLA-DRA***	2903189		**darkred (** ***p*** ** = 0.0046)**	141	**0.8**	0.23	**lightcyan (** ***p*** ** = 0.0139)**	250	**0.66**	0.51
***CTSC***	3385769				**0.73**	0.5			**0.69**	0.47
***TMEM106B***	2990342		**red**	925	**0.78**	**0.04**	**darkturquoise**	164	**0.86**	**0.01**
***C9orf72***	3202421	**spectrum**	**purple (** ***p*** ** = 0.0226)**	1559	**0.74**	**0.1**	**purple (** ***p*** ** = 0.0206)**	830	**0.75**	0.14
***VCP***	3204404				**0.68**	0.19			**0.71**	0.24
***UBQLN2***	3978999				0.55	0.45			**0.63**	0.45
***OPTN***	3235726				0.59	0.38	grey60	220	0.53	0.8
*FUS*	3656904		lightcyan	209	0.58	0.61	midnightblue	268	**0.64**	0.31
*FUS*	3656950		blue	3329	0.31	0.69	pink	2979	0.39	0.58
*FUS*	3656954				0.5	0.38	magenta	1000	0.44	0.72
*TDP-43*	2320048		turquoise	4759	0.22	0.89			0.53	0.54

The FTD-genes (with corresponding Affymetrix IDs) are listed. Co-expression modules for the FTD-genes and their relevance within modules are displayed. Sz = size, i.e. number of transcripts contained in the module; MM = module membership; 1-q = 1-quantile of MM. The bolded parts highlight the fact that WGCNA indicated the following: 1) (*MAPT* and *GRN*), (*HLA-DRA* and *CTSC*), (*C9orf72*, *VCP*, *UBQLN2* and *OPTN*) and (2 *FUS*) transcripts were respectively present in the same modules in frontal cortex, and; 2) (*HLA-DRA* and *CTSC*), (*C9orf72*, *VCP* and *UBQLN2*) and (*FUS* and *TARDBP*) transcripts were respectively present in the same modules in temporal cortex. The modules containing (*HLA-DRA* and *CTSC*) and (*C9orf72*, *VCP*, *UBQLN2* [and *OPTN*]) were significantly enriched for FTD transcripts

We found four modules of interest in frontal cortex (Table 2): one included *MAPT* and *GRN*, where *MAPT* was a hub gene. Another module comprised *HLA-DRA* and *CTSC*: both genes showed high module membership (MM) values, whilst none was a hub gene. A third module displayed *TMEM106B* that was a hub, and the fourth included *C9orf72*, *VCP*, *UBQLN2* and *OPTN*, where *C9orf72* was a hub. The remaining genes (*CHMP2B*, *FUS* and *TARDBP*) had poor module assignments (Table 2).

In the temporal cortex, besides *MAPT* and *GRN* that were assigned to two distinct modules (where neither one was a hub), we observed similar co-expression patterns as in the case of the frontal cortex (Table 2): *HLA-DRA* and *CTSC* were again co-assigned to the same module with moderately high MM values (>0.65) but no hub status; *TMEM106B* was again a hub within its module of membership, and *C9orf72*, *VCP* and *UBQLN2* were all again assigned to the same module where each had high MM values, but none was a hub. As seen in the frontal cortex, *CHMP2B*, *OPTN*, *FUS* and *TARDBP* did not hold major relevance in temporal cortex modules (Table 2).

**Fig. 2:**
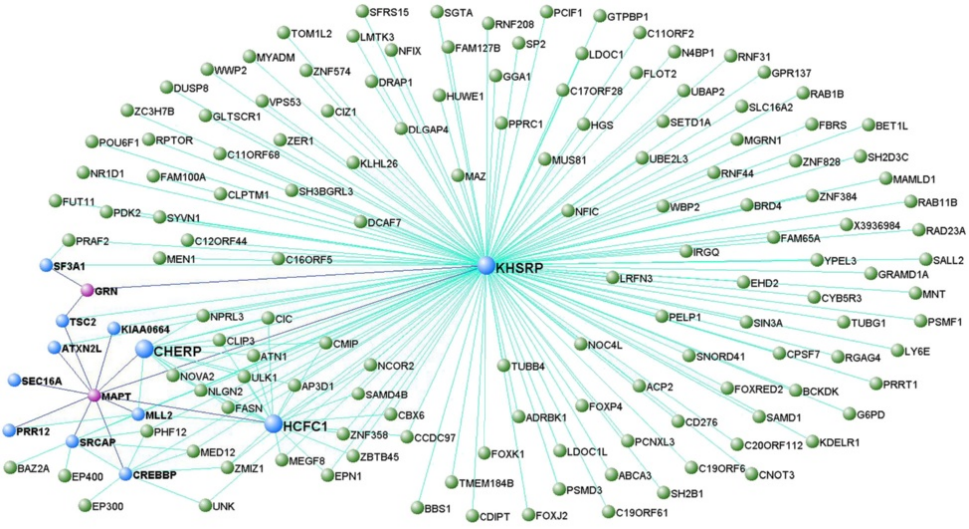
Network visualisation of genes of the black module in frontal cortex. VisANT plot shows genes (blue, gene names in bold) directly connected with a topological overlap measure TOM > 0.10 to FTD-genes, *MAPT* and *GRN* (purple, gene names in bold). All such genes are important hubs within this module whose connections are based on TOM > 0.115

**Fig. 3:**
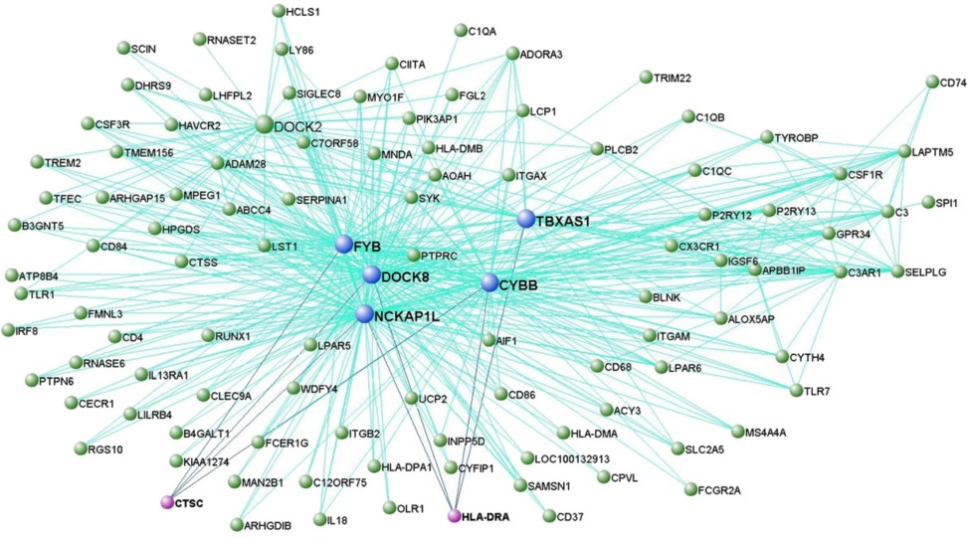
Network visualisation of genes of the darkred module in frontal cortex. VisANT plot shows genes (blue, gene names in bold) directly connected to FTD-genes, *CTSC* and *HLA-DRA* (purple, gene names in bold). All such genes are important hubs within this module and all connections shown here are based on TOM >0.165

**Fig. 4:**
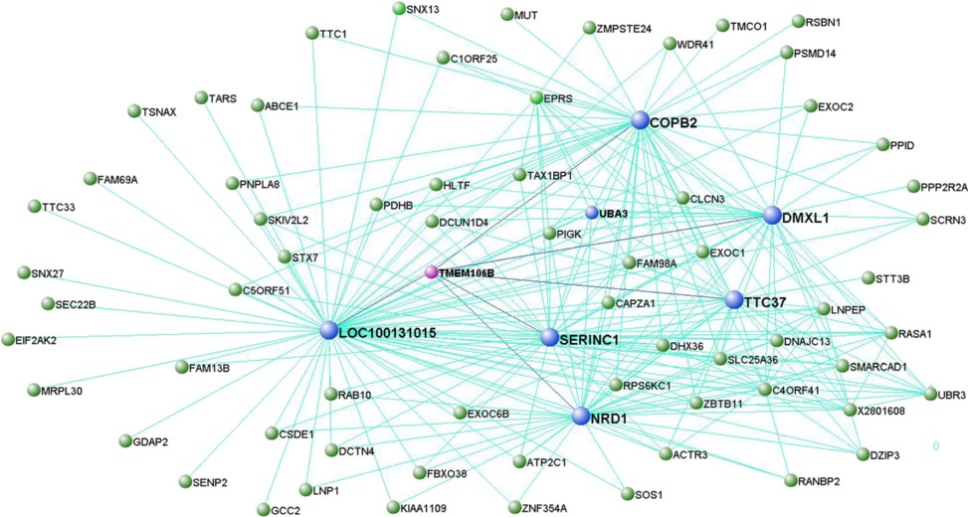
Network visualisation of genes of the red module in frontal cortex. VisANT plot shows genes (blue, gene names in bold) directly connected to FTD gene, *TMEM106B* (purple, gene name in bold). All such genes are important hubs within this module and all connections shown here are based on TOM > 0.1

**Fig. 5:**
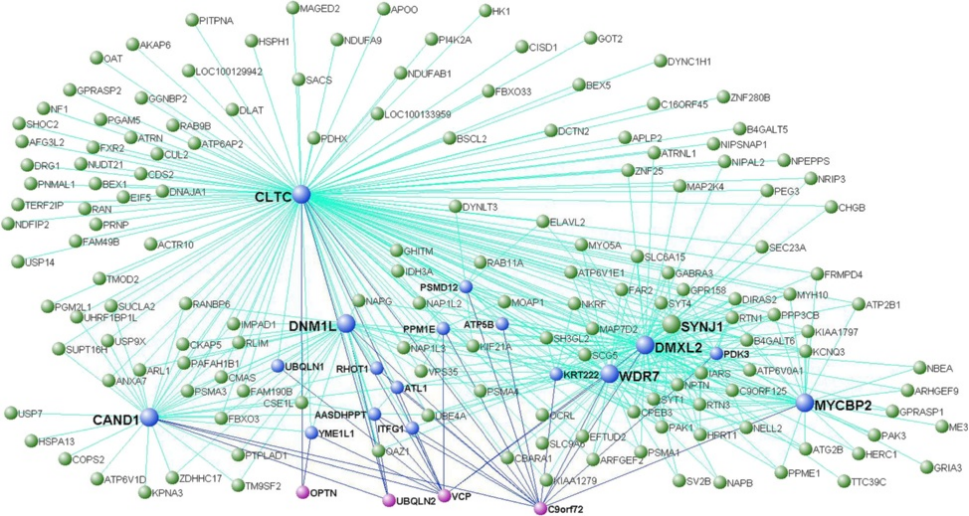
Network visualisation of genes of the purple module in frontal cortex. VisANT plot shows genes (blue, gene names in bold) directly connected with a topological overlap measure TOM > 0.08 to FTD-genes, *OPTN, UBQLN2, VCP and C9orf72* (purple, gene names in bold). All such genes are important hubs within this module whose remaining connections are based on TOM >0.11

Enrichment analysis identified the modules including *HLA-DRA* and *CTSC*, and *C9orf72*, *VCP*, *UBQLN2* (and *OPTN*) as significantly enriched for FTD-genes (*p* < 0.05; Table 2). To visualize these as well as the interconnection networks of *MAPT*, *GRN* and *TMEM106B* we generated Figs. 2, 3, 4 and 5 for which the main nodes are listed in Additional file 2: Table S20.

Since the FTD-genes distributed rather similarly within the modules in frontal and temporal cortex, we sought to further investigate the clustering across other brain tissues. We discovered moderate to high quantitative overlap (i.e. number of shared genes) between frontal and temporal cortex modules as shown and explained in the Additional file 1: Figure S17 (p 26). Also, we verified through composite Z-summary preservation statistics that the modules containing FTD-genes in frontal and temporal cortex were, for the most, preserved across other brain regions (Additional file 1, p 27).

### Replication

**Fig. 6:**
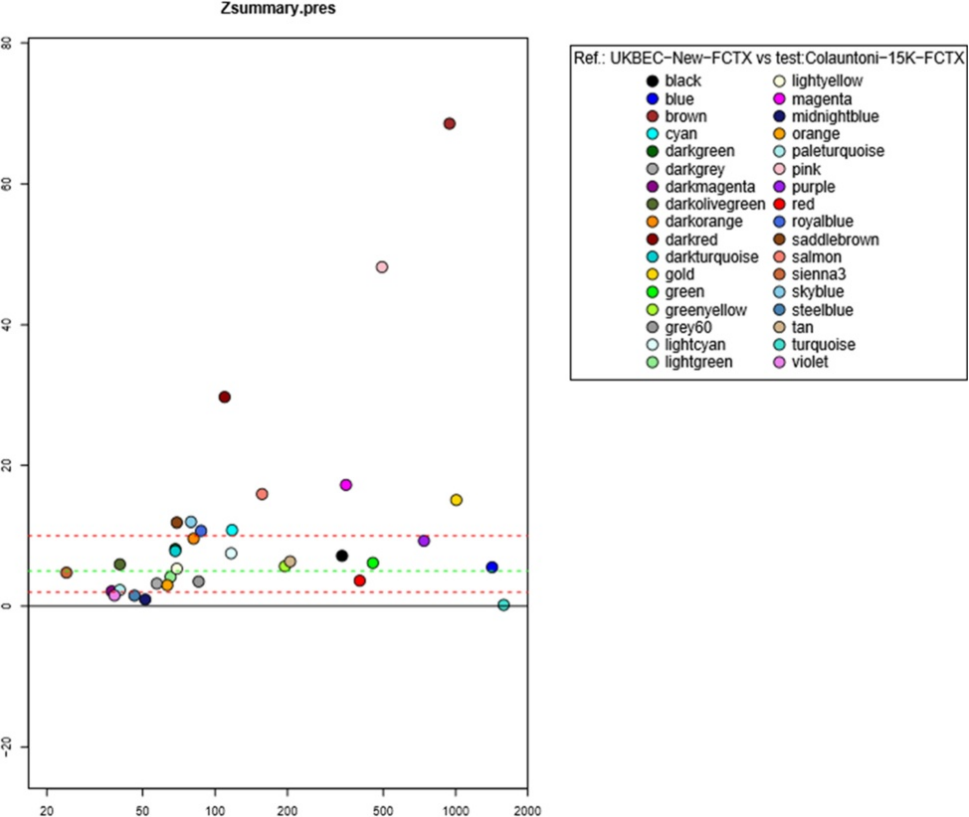
Z-summary graph. The ‘Z-summary preservation estimate’ estimates how interaction patterns of genes within modules one dataset are preserved in another dataset, i.e. it estimates how the genes which cluster together in the reference tissue maintain that clustering in the other dataset. The Z preservation summary is a mean of both estimates. The colour codes are those of reference, i.e. from our own dataset

In order to replicate and support our findings, we assessed the reproducibility of the frontal cortex networks as a ‘whole ‘and ‘module by module’, using an independent and well established dataset [18]. Our WGCNA TOM (topological overlap measure) was significantly reproduced in the WGCNA TOM matrix constructed from the Colauntoni dataset (*p*-value = 1x10^-3^), thus supporting robust correlation between the two datasets. Further, through the Z-summary preservation estimate we noted that 10 of our frontal cortex modules showed strong evidence of preservation (Z-summary > 10) in the Colauntoni dataset, and 30 had moderate to high preservation levels (2 < Z-summary < 10). Particularly, the Z scores for the modules of interest – based on Table 2 – were all supportive of preservation: red (*TMEM106B*) module (Z = 3.61), black (*MAPT* and *GRN*) module (Z = 7.15), purple (*C9orf72*, *VCP*, *UBQLN2* and *OPTN*) module (Z = 9.28) and darkred (*HLA-DRA* and *CTSC*) module (Z = 29.73) (Fig. 6).

### Functional annotation and pathway analyses

To gain insight into their biological significance, we annotated genes in the network assessing gene ontology (GO) terms for biological processes (BPs), cellular components (CCs) and molecular functions (MFs) and performed pathways analyses for the relevant FTD-modules. The analyses of modules in frontal and temporal cortices (based on Table 2) are presented hereafter, whilst those of other brain tissues are summarized in the Additional file 1 (pp 8–9) and Additional file 2: Table S19.

In frontal cortex, significant GO terms associated with the module containing *MAPT* and *GRN* indicated transcription processes and chromatin metabolism with a particular involvement of the histone methyltransferase complex as well as transcription factor and nucleic acid binding activities (Table 3a); such annotations were supported by pathways analysis that also indicated the metabolism of the chromatin. Evaluation of the list of predefined enrichment for brain terms in WGCNA (see Additional file 1, pp 28–29) revealed significant overlap with the green M10 module associated with Glutamatergic Synaptic Function in neurons identified in [19] (Table 3a). In temporal cortex, the *MAPT*-containing module was enriched for transcription processes (Table 3b and Additional file 1, p 5), whereas the *GRN*-containing module for broad biology of membrane-bounded organelle (Table 3b and Additional file 1, p 5). Here, the list of predefined enrichment for brain terms in WGCNA showed significant overlap particularly with the green M10 module associated with Glutamatergic Synaptic Function in neurons identified in [19] for both modules. The complete list of GO terms associated with the modules including *MAPT* and *GRN* in frontal and temporal cortices can be found in Additional file 2: Tables S2, S6 and S7.

**Table 3:** Summary of most relevant GO terms, pathways and brain lists annotation for relevant modules in frontal cortex (a) and temporal cortex (b)

FTD gene	MM	1-q	type	module	sz	G:Profiler Annotation (GO terms & Pathways analyses)	WGCNA – Brain lists
a.							
*MAPT*	0.83	**0.03**	pure	black	791	**BP**: Transcription, DNA-templated (p=2.77x10^-5^); RNA biosynthetic process (p=3.17x10^-5^); Chromatin modification (p=9.45x10^-5^) **CC**: Nuclear lumen (p=2.81x10^-13^); Histone methyltransferase complex (p=3.2x10^-7^) **MF**: Transcription factor binding transcription factor activity (p=6.26x10^-6^); Nucleic acid binding (p=7.19x10^-6^)c3v1234 **Pathways**: Chromatin organization and Chromatin modifying enzymes (p=4.84x10^-3ZX^)	Glutamatergic Synaptic Function (CTX) (p=1.36x10^-7^)
*GRN*	0.66	0.34					
*HLA-DRA*	0.8	0.23		darkred	141	**BP**: Immune system process (p=1.39x10^-31^); Defense response (p=5.23x10^-29^); Innate immune response (p=2.51x10^-19^); Adaptive immune response (p=1.71x10^-7^); Phagocytosis (p=6.2x10^-3^) **CC**: MHC class II protein complex (p=1.17x10^-7^); Lysosome (p=7.69x10^-5^); Lytic vacuole (p=7.69x10^-5^) **MF**: Receptor activity (p=1.88x10^-8^); Signal transducer activity (p=6.45x10^-6^) **Pathways**: Immune system (p=4.8x10^-21^); Phagosome (p=6.69 x 10^-12^); Adaptive immune system (p=5.08 x 10^-10^); Cytokine signaling in immune system (p=2.63 x 10^-7^); Lysosome (p=4.89 x 10^-2^)	Microglia (Type1) (HumanMeta) (p=1.53x10^-84^) Microglia (Type1) (CTX) (p=7.01x10^-31^) Up In Frontal Cortex (Early AD) (p=2.96x10^-19^)
*CTSC*	0.73	0.5					
*TMEM106B*	0.78	**0.04**		red	925	**BP**: Cellular protein metabolic process (p=3.84x10^-5^) **CC**: Nuclear lumen (p=9.36x10^-6^); Catalytic complex (p=1.36x10^-2^) **MF**: Ligase activity (p=1.96x10^-2^) **Pathways**: Chromatin organization (p=1.1x10^-2^); HATs acetylate histones (p=1.3 x 10^-2^); HDACs deacetylate histones (p=3.2 x 10^-2^)	Protein metabolism (CTX) (p=7.57x10^-10^) Neuron (CTX) (p=4.83x10^-5^) Nucleus (HumanMeta) (p=1.09x10^-5^) Oligodendrocyte probable (Cahoy) (p=2.42x10^-5^) Brown pyramidal Neurons Layer5/basolateral Amygdala (Sugino/Winden) (p=2.80x10^-5^)
*C9orf72*	0.74	**0.1**	spectrum	purple	1559	**BP**: Modification-dependent macromolecule catabolic process (p=3.9x10^-7^); Ubiquitin-dependent protein catabolic process (p=1.08x10^-6^); Proteolysis involved in cellular protein catabolic process (p=1.72x10^-6^) **CC**: Cytoplasm (p=2.95x10^-14^); Mitochondrion (p=2.4x10^-12^); Proteasome complex (p=2.81x10^-4^) **MF**: Catalytic activity (p=3.55x10^-4^) **Pathways**: Protein processing in endoplasmic reticulum (p=1.01x10^-5^); Mitochondrial translation (p=2.28x10^-3^)	Neuron (CTX) (p=8.76x10^-27^) Neuron (HumanMeta) (p=9.02x10^-25^) Mitochondrion (p=1.63x10^-19^) Post Synaptic Density proteins (Bayes) (p=8.73x10^-18^)
*VCP*	0.68	0.19					
*UBQLN2*	0.55	0.45					
*OPTN*	0.59	0.38					
b.							
MAPT	0.67	0.31	pure	lightyellow	210	**BP**: Transcription from RNA polymerase II promoter (p=1.23x10^-4^) **CC**: Nucleus (p=3.69x10^-5^) **MF**: Protein binding (p=2.93x10^-4^); Transcription factor binding (p=1.42x10^-2^) **Pathways**: RNA Polymerase II Transcription Elongation (p=4.21x10^-2^)	Glutamatergic Synaptic Function (CTX) (p=3.79x10^-5^)
GRN	0.56	0.51		cyan	276	**CC**: intracellular membrane-bounded organelle (p=7.56x10^-3^) **Pathways**: Signaling by Wnt (p=7.61x10^-3^); Lysosome (p=1.21x10^-2^)	Glutamatergic Synaptic Function (CTX) (p=9.89x10^-8^) Mitochondria (HumanMeta) (p=1.74 x10^-5^)
HLA-DRA	0.66	0.51		lightcyan	250	**BP**: Immune system process (p=1.89x10^-37^); Defense response (p=1.51x10^-32^); Phagocytosis (p=1.39x10^-5^) **CC**: MHC class II protein complex (p=3.02x10^-6^); Lysosome (p=5.43x10^-5^); Lytic vacuole (p=5.43x10^-5^) **MF**: Receptor activity (p=4.41x10^-10^) **Pathways**: Immune system (p=8x10^-22^); Phagosome (p=7.2x10^-13^); Innate immune system (p=1x10^-11^); Adaptive immune system (p=2.56x10^-9^)	Microglia (Type1) (HumanMeta) (p=1.53x10^-84^) Microglia (Type1) (CTX) (p=1.08x10^-30^) Up In Frontal Cortex (EarlyAD) (p=7.85x10^-19^)
CTSC	0.69	0.47					
TMEM106B	0.86	**0.01**		darkturquoise	164	**CC**: Cytoplasm (p=2.8x10^-5^); Nucleus (p=2.49x10^-4^) **Pathways**: Protein processing in endoplasmic reticulum (p=4.86x10^-5^)	Nucleus (CTX) (p=3.67x10^-9^) Metabolism (CTX) (p=3.48x10^-5^)
C9orf72	0.75	0.14	spectrum	purple	830	**BP**: Proteolysis involved in cellular protein catabolic process (p=1.81x10^-8^); Ubiquitin-dependent protein catabolic process (p=3.04x10^-8^); Protein catabolic process (p=1.87x10^-6^) **CC**: Cytoplasm (p=1.28x10^-12^); Proteasome complex (p=1.16x10^-5^); Endoplasmic reticulum membrane (p=5.21x10^-3^) **MF**: Catalytic activity (p=1.08x10^-4^) **Pathways**: Protein processing in endoplasmic reticulum (p=5.25x10^-6^)	Neuron (CTX) (p=9.29x10^-24^) Neuron (HumanMeta) (p=6.96x10^-11^) Post Synaptic Density proteins (Bayes) (p=4.34x10^-5^)
VCP	0.71	0.24					
UBQLN2	0.63	0.45					

Functional annotation analysis for the modules comprising *HLA-DRA* and *CTSC* indicated modulation of the immune responses via the innate and adaptive systems in both the frontal (Table 3a) and temporal cortices (Table 3b). Pathways analysis revealed implication of immune system, phagosomes, antigen processing and presentation, interferon gamma- and cytokine-signalling, and lysosomes (Table 3a and b, and Additional file 2: Table S17), and the list of predefined enrichment for brain terms in WGCNA suggested enrichment for microglia markers given significant overlap with pink M10 and purple M4 Microglia (Type1) modules identified in [17, 19, 20] (Table 3a and b). The complete list of GO terms associated with the modules containing *HLA-DRA* and *CTSC* in the frontal and temporal cortices can be found in Additional file 2: Tables S4 and S9.

For the modules including *TMEM106B*, we noted GO terms indicating protein metabolic processes exerted through catalytic complexes in frontal cortex (Table 3a), whereas terms were rather general in temporal cortex (Table 3b). Pathway analysis pointed to chromatin metabolism for the frontal cortex (Table 3a) and protein processing in endoplasmic reticulum (ER) for the temporal cortex (Table 3b). The list of predefined enrichment for brain terms in WGCNA indicated that both the *TMEM106B*-containing modules in frontal and temporal cortex held overlapping features with the yellow M18 (enriched for protein metabolism) and the blue M16 Neuron modules identified in [19] (Table 3a). In addition, there was further overlap – for the frontal cortex module only – with the turquoise M14 Nucleus, Oligodendrocyte probable and brown pyramidal Neurons Layer5/basolateral Amygdala modules found in [20–23] (Table 3a), indicating that the TMEM106B protein most probably takes part in different biological processes in diverse brain areas. The complete list of GO terms associated with the modules including *TMEM106B* in frontal and temporal cortices can be found in Additional file 2: Tables S5 and S10.

Functional annotation analysis for the module containing *C9orf72*, *VCP*, *UBQLN2* (and *OPTN*) in the frontal and temporal cortices pointed to ubiquitin-mediated protein catabolic process entailing proteasome and proteolysis activities, and to ER-associated ubiquitin-dependent protein catabolic process and endosomal transport (Table 3a and b). Pathways analysis hinted to protein processing in ER and also supported the ubiquitin-dependent degradation of proteins and the proteasome biology (Table 3a and b, and Additional file 2: Table S17). In addition, there was significant overlap with numerous WGCNA lists particularly pointing to the blue M16 Neuron module identified in [19] (Table 3a and b). The complete list of GO terms associated with the modules including *C9orf72*, *VCP*, *UBQLN2* (and *OPTN*) in the frontal and temporal cortices can be found in Additional file 2: Tables S11 and S14.

For completeness, we also performed functional annotation and pathway analysis for the genes with poor module assignments (*CHMP2B*, *FUS* and *TARDBP*; Table 2): results are briefly detailed in the Additional file 1, pp 4–8 and in Additional file 2: Table S19.

### Protein-protein interactors (PPIs) of FTD-genes

We sought to verify whether genes clustering together with FTD-genes in the relevant FTD-modules are found to interact at the protein level with the FTD-genes products. We searched for known PPIs of *MAPT*, *GRN*, *HLA-DRA*, *CTSC*, *TMEM106B*, *C9orf72*, *VCP*, *UBQLN2* and *OPTN* (Additional file 2: Table S21a and b), and assessed any nominal overlap with genes co-clustering in modules containing the FTD-genes in the frontal and temporal cortices. Only for *TMEM106B* no nominal overlap between PPIs and related transcripts was seen.

Among the MAPT-PPIs, *MARK2* (MAP/microtubule affinity-regulating kinase 2), *MARK4* (MAP/microtubule affinity-regulating kinase 4) and *EP300* (E1A binding protein p300) were assigned to the module containing *MAPT* in frontal cortex. Here, *MARK2* was a hub, whilst *MARK4* and *EP300* were among the top ~15 % interactive genes (Additional file 2: Table S1a). *MARK2* and *MARK4* encode kinases that target proteins involved in stabilizing the microtubules, while *EP300* encodes an acetyltransferase; of note, *MARK2* and *EP300* co-clustered with *MAPT* within GO terms pointing to the nucleus and indicated features such as DNA-, RNA- and protein-binding (*MARK2*), and transferase/catalitic complexes and nuclear chromatin (*EP300*) in frontal cortex (Additional file 2: Table S2). Conversely, *MARK2*, *EP300* and *AKT1* (v-akt murine thymoma viral oncogene homolog 1) were assigned to the *MAPT*-containing module in temporal cortex (Additional file 2: Table S6), where *MARK2* was a hub (Additional file 2: Table S1b). The protein kinase *AKT1* is a critical mediator of growth factor-induced neuronal survival in the developing nervous system. These 3 genes were found in GO terms pointing to the nucleus, together with *MAPT*, and transcription or RNA metabolic processes (Additional file 2: Table S6).

Among the GRN-PPIs, *ATN1* (atrophin 1), *SGTA* (small glutamine-rich tetratricopeptide repeat (TPR)-containing, alpha), *CRKL* (v-crk avian sarcoma virus CT10 oncogene homolog-like) and *TLE3* (transducin-like enhancer of split 3) were assigned to the module containing *GRN* in frontal cortex. Here, *ATN1*, that encodes atrophin 1, a conserved transcriptional co-repressor [24] and whose expansions have been associated with neurodegeneration [25], was a hub (Additional file 2: Table S1a). *SGTA* encodes a small glutamine-rich tetratricopeptide repeat (TPR)-containing, alpha that might be involved in neuronal apoptotic processes [26]. *CRKL* encodes an oncogene and seems pleiotropic in physiologic signalling [27], whilst *TLE3* is a transcriptional co-repressor. *ATN1* and *TLE3* were found in gene lists indicating GO terms pointing to the nucleus and transcription related processes (Additional file 2: Table S2). No relevant functional annotations were available for *SGTA*, whereas *CRKL* appeared to be involved in DNA- and RNA-binding (with *GRN*; Additional file 2: Table S2). Conversely, *TLE3* and *CRKL* were assigned to the *GRN*-containing module in temporal cortex and annotation analysis indicated GO terms involving the biology of membrane-bounded organelle (with *GRN*; Additional file 2: Table S7).

There was nominal overlap between HLA-DRA-PPIs and relative genes (Additional file 2: Table S21a), whereas there was none for CTSC. *HLA-DMB* (major histocompatibility complex, class II, DM beta), *HLA-DMA* (major histocompatibility complex, class II, DM alpha) and *CD74* (CD74 molecule, major histocompatibility complex, class II invariant chain) were present in both modules containing *HLA-DRA* in the frontal (Additional file 2: Table S4) and temporal cortex (Additional file 2: Table S9). *HLA-DMA* and *HLA-DMB* encode the major histocompatibility complex, class II, DM alpha and beta, which is anchored in the membrane of intracellular vesicles and plays a central role in the peptide loading of MHC class II molecules [28]. *CD74* encodes a chaperone that regulates antigen presentation during immune response. *HLA-DMB*, *HLA-DMA* and *CD74* were included within all or most of the GO terms indicated by our functional annotation analysis together with *HLA-DRA* and/or *CTSC* (Additional file 2: Tables S4 and S9).

A large number of PPIs of VCP, UBQLN2 and OPTN nominally overlapped with the genes co-expressed with *C9orf72*, *VCP*, *UBQLN2* (and *OPTN*) in the relative modules in the frontal and temporal cortices; conversely, this was the case only for a few of the C9orf72-PPIs (Additional file 2: Table S21b). We found up to 10 PPIs of VCP that were also hubs in frontal and/or temporal cortex (Additional file 2: Table S1a and b): *CUL2* (cullin 2), *UBQLN1* (ubiquilin 1), *NF1* (neurofibromin 1), *NIPSNAP1* (nipsnap homolog 1), *BTRC* (beta-transducin repeat containing E3 ubiquitin protein ligase), *ARFGEF2* (ADP ribosylation factor guanine nucleotide exchange factor 2), *COPS3* (COP9 signalosome subunit 3), *PLAA* (phospholipase A2-activating protein), *CLTA* (clathrin, light chain A) and *ANXA7* (annexin A7); all such proteins are involved, mainly, in either protein catabolic or intracellular vesicle transport processes (Table 4). For UBQLN2 we found 5 PPIs that were also hubs in the frontal and/or temporal cortex (Additional file 2: Table S1a and b): *UBQLN1*, *SEC23A* (Sec23 homolog A, COPII coat complex component), *USP9X* (ubiquitin specific peptidase 9, X-linked), *STAM* (signal transducing adaptor molecule) and *HSPA13* (heat shock protein family A (Hsp70) member 13); these are, overall, involved in protein degradation and ER-Golgi protein transport processes (Table 4). In the case of OPTN we counted two PPIs that were also hubs (Additional file 2: Table S1a and b): *RAB11A* (RAB11A, member RAS oncogene family) and *RTN3* (reticulon 3), involved in protein transport and modulation of ß-amyloid production, respectively (Table 4). Finally, for C9orf72 we found only three PPIs (none of which was a hub): *APP* (amyloid beta (A4) precursor protein), *ELAVL1* (ELAV like RNA binding protein 1) and *EIF2B2* (eukaryotic translation initiation factor 2B subunit beta), linking C9orf72, provided further evidence, to the ß-amyloid production, RNA metabolism and protein synthesis, respectively (Table 4). Interestingly, we noticed that all genes mentioned above (and PPI interactors of VCP, UBQLN2, OPTN or C9orf72) were included together with the spectrum FTD-genes in the gene lists supporting GO terms indicating functions pointing to protein catabolism pathways and cytoplasmic protein transport and/or vesicle trafficking as highlighted in Additional file 2: Tables S11 and/or S14.

**Table 4:** Novel potential risk factors in FTD

Novel potential risk factor	Interactor of	Function	Topography	Evidence
*KHSRP*	*MAPT, GRN*	involved in alternative pre-mRNA splicing and mRNA localization	FCTX	WGCNA + VisANT
*SF3A1*	*GRN*			
*CREBBP*	*MAPT*	acetyltransferase involved in chromatin remodelling and transcriptional activation/regulation		
*MLL2*		methyl-transferase involved in chromatin remodelling		
*SRCAP*		involved in transcriptional activation/regulation		
*HCFC1*				
***MARK2***	***MAPT***	**kinase involved in stabilizing the microtubules and tau’s phosphorylation**	**FCTX, TCXT**	**WGCNA + PPI**
***MARK4***			**FCTX**	
***EP300***		**acetyltransferase involved in tau’s acetylation**	**FCTX, TCXT**	
***AKT1***		**kinase involved in growth factor-induced neuronal survival in the developing nervous system**	**TCTX**	
***ATN1***	***GRN***	**transcriptional co-repressor factor**	**FCTX**	
***SGTA***		**involved in neuronal apoptotic processes**		
***CRKL***		**oncogene pleiotropic in physiologic signalling**	**FCTX, TCXT**	
***TLE3***		**transcriptional co-repressor factor**		
*CYBB*	*HLA-DRA, CTSC*	critical component of the oxidase system of phagocytes	FCTX	WGCNA + VisANT
*DOCK8*		involved in neuronal development and immune cells shaping		
***HLA-DMA***	***HLA-DRA***	**transmembrane protein of intracellular vesicles involved in peptide loading of MHC class II molecules**	**FCTX, TCXT**	**WGCNA + PPI**
***HLA-DMB***				
***CD74***		**chaperone involved in antigen presentation during immune response**		
*COPB2*	*TMEM106B*	involved in Golgi budding and vesicular trafficking	FCTX	WGCNA + VisANT
*SERINC1*		involved in lipid biosynthesis in neurons at the ER level		
*NRD1*		metalloprotease with potential neuropathogenic role		
*TTC37*		protein-protein interactor with chaperone activity		
*CAND1*	*C9orf72, VCP*	involved in ubiquitin ligase network	FCTX	WGCNA + VisANT
*PSMD12*	*C9orf72*	subunit of a multi-catalytic proteinase complex		
*MYCBP2*	*C9orf72*	E3 ubiquitin protein ligase (alias)		
*ATL1*	*C9orf72, VCP*	involved in axonal maintenance		
*UBQLN1*	*VCP*	ubiquitin-like protein which links the ubiquitination and proteasome machineries		
***APP***	***C9orf72***	**cell surface receptor and transmembrane precursor protein cleaved by secretases into different peptides: some of these can bind to the acetyltransferase complex (APBB1/TIP60) to promote transcriptional activation; others form the protein basis of the amyloid plaques**	**FCTX, TCTX**	**WGCNA + PPI**
***ELAVL1***		**RNA-binding protein that contain several RNA recognition motifs, and selectively bind AU-rich elements (AREs) found in the 3' untranslated regions of mRNAs. AREs signal degradation of mRNAs as a means to regulate gene expression; the ELAVL family stabilizes ARE-containing mRNAs**		
***EIF2B2***		**beta subunit of eukaryotic initiation factor-2B (EIF2B). EIF2B is involved in protein synthesis and exchanges GDP and GTP for its activation and deactivation**	**FCTX**	
***CUL2***	***VCP***	**Cullins are a family of NEDD8 targets important in the stabilization and degradation of proteins**	**FCTX, TCTX**	
***UBQLN1***		**part of the ubiquitination machinery of the proteasome to affect in vivo protein degradation**		
***NF1***		**negative regulator of the ras signal transduction pathway (control such processes as actin cytoskeletal integrity, proliferation, differentiation, cell adhesion, apoptosis and cell migration)**		
***NIPSNAP1***		**family of proteins involved in vesicular transport**	**FCTX**	
***BTRC***		**constitutes one of the four subunits of ubiquitin protein ligase complex called SCFs (SKP1-cullin-F-box) that function in phosphorylation-dependent ubiquitination**	**FCTX, TCTX**	
***ARFGEF2***		**plays an important role in intracellular vesicular trafficking; involved in Golgi transport**	**FCTX**	
***COPS3***		**kinase activity that phosphorylates regulators involved in signal transduction**		
***PLAA***		**activation of protein kinase C (PKC) and PKC-dependent responses (in response to inflammatory mediators and release during apoptosis)**		
***CLTA***		**part of structural component of the lattice-type cytoplasmic face of coated pits and vesicles which entrap specific macromolecules during receptor-mediated endocytosis with regulatory function**		
***ANXA7***		**a membrane binding protein with diverse properties (voltage-sensitive calcium channel activity, ion selectivity and membrane fusion)**		
***UBQLN1***	***UBQLN2***	**part of the ubiquitination machinery of the proteasome to affect in vivo protein degradation**	**FCTX, TCTX**	
***SEC23A***		**suggested to play a role in the ER-Golgi protein trafficking**		
***USP9X***		**protein similar to ubiquitin-specific proteases**		
***STAM***		**mediates downstream signaling of cytokine receptors and also play a role in ER to Golgi trafficking**		
***HSPA13***		**member of the heat shock protein 70 family and is found associated with microsomes. Members of this protein family play a role in the processing of cytosolic and secretory proteins, as well as in the removal of denatured or incorrectly-folded proteins**		
***RAB11A***	***OPTN***	**involvedin constitutive, regulated secretory pathways and protein transport**	**FCTX**	
***RTN3***		**expressed in neuroendocrine tissues: interacts with and modulates the activity of beta-amyloid converting enzyme 1 (BACE1), and the production of amyloid-beta**		

Each novel potential risk factor is listed along with the interacting FTD-gene(s). The evidence of interaction is primarily defined by our WGCNA data that assigned each transcript to a module containing one (or more) FTD-gene(s) in frontal and/or temporal cortex. The nomenclature (WGCNA + VisANT) indicates that the novel potential risk factor is chosen because of its hub status and its interaction with FTD-gene(s) based on topological overlap measure (TOM) > 0.10 (see also Figs. 2, 3, 4 and 5). The nomenclature (WGCNA + PPI) indicates that the novel potential risk factor is chosen based on nominal overlap between interactive transcript(s) and protein(s). The potential risk factors belonging to the latter category are bolded as an indication that the WGCNA + PPI combination could be a strong indicator for regional-specific impacted functional networks. The main known function(s) of each novel potential genetic and/or functional risk factor is included in the central column. FCTX = frontal cortex; TCTX = temporal cortex

Taken all together, the module assignments in the frontal and temporal cortices as well as the functional annotation, pathways and PPIs analyses provide not only a more comprehensive picture of the potential biological and cellular mechanisms involved in the development of FTD but also an enlarged domain of novel potential genetic or functional risk factors associated with FTD (that are comprehensively summarized in Table 4).

## Discussion

In this study we used a systems biology approach based on gene co-expression network analysis of microarray expression data to investigate genes known to be associated with FTD. After a general assessment of their expression levels in different brain regions, we particularly evaluated co-expression patterns in brain areas known to be affected in FTD with a major focus on the frontal and temporal cortices and inferred biological processes potentially implicated in the pathogenesis of FTD. We also sought to identify novel potential risk factors for FTD.

Prior to putting our results into context, a number of limitations that apply to this work need to be acknowledged: i) although the prevalence of FTD is almost equal among males and females [3], data available for this study were generated mainly in male individuals (78/101); ii) the disease generally manifests between mid 50s to early 60s years of age and, although expression data were corrected for individual effects including sex and age at death, the study cohort had a mean age of 50 years (ranging from 16 to 83 years); iii) besides that there is no golden standard approach or pipeline in systems biology studies, it is important to note that data supporting functional annotation and enrichment analyses presented here are based on the current literature (i.e. focused on a restricted number of targets or pathways) and that data mining has been manually curated or generated by semantic associations, thus some or novel interactions might be overlooked.

The data on expression levels across brain tissues indicated that *MAPT*, *GRN*, *CHMP2B*, *CTSC*, *HLA-DRA*, *TMEM106B*, *C9orf72*, *VCP*, *SQSTM1*, *UBQLN2*, *OPTN*, *TARDBP* and *FUS* are all robustly expressed in (thus clearly hold relevance for the biology of) the aging brain. Comparatively, by showing rather lower expression rates, it is a possibility that *RAB38*, *BTNL2* and *HLA-DRB5* might rather represent sensitive cellular markers and sudden changes in their expression levels might impact cellular homeostasis.

The current work revealed that *MAPT*–*GRN*, *HLA-DRA*–*CTSC*, *TMEM106B* and *C9orf72*–*VCP*–*UBQLN2*–*OPTN* clustered in interesting modules in the frontal and/or temporal cortex and that, particularly on the basis of functional annotation and pathway analyses, three main biological processes with a direct relation to FTD-genes hold relevance to the pathogenesis of FTD: i) DNA & chromatin biology; ii) immune & lysosomal processes, and; iii) protein meta/catabolism.

**Fig. 7:**
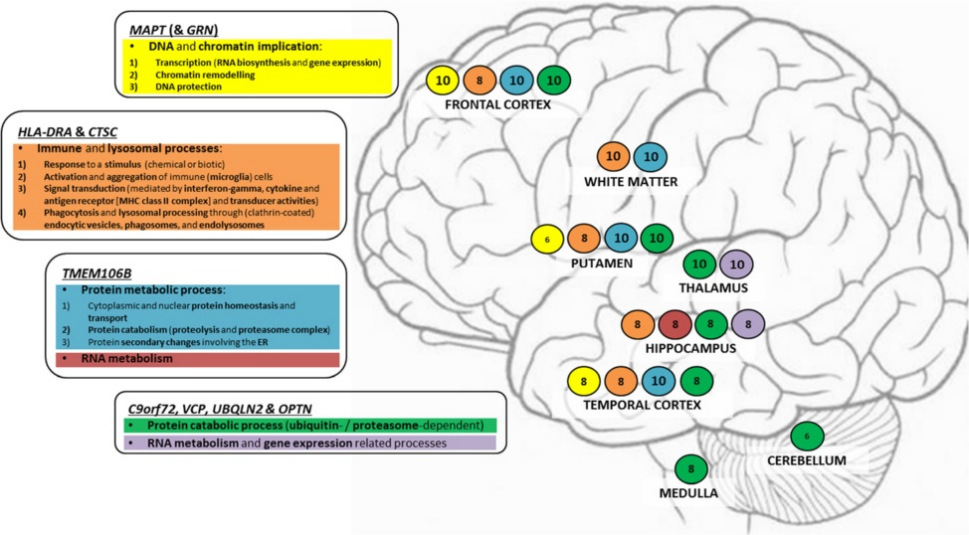
Scheme of the regional distribution of the FTD-genes and associated annotated biological processes or functions. Rectangles contain FTD-gene(s) and their main associated regional-specific biological process. Each colour indicates a particular biological process. Font size differs based on statistics associated with the transcript(s) within their assigned modules. Font 10 = hub (1-q < 0.1) + MM > 0.7; Font 8 = 1-q > 0.1 + MM > 0.6, and; Font 6 = 1-q > 0.1 + MM ≤ 0.6. If multiple transcripts belong to a module, at least 1 has values that justify the font size

The first biological process, i.e. DNA and chromatin implication (including transcription [i.e. RNA biosynthesis and gene expression] and chromatin remodelling), was defined by modules containing *MAPT* (and *GRN*), and appeared to be specific to neurons in the frontotemporal cortices, and the putamen (Fig. 7). This is, overall, a relatively novel concept in FTD, and it is noteworthy to appreciate that genes responsible for alternative pre-mRNA splicing and mRNA localization (*KHSRP* [KH-type splicing regulatory protein] and *SF3A1* [splicing factor 3a, subunit 1]), chromatin remodelling such as acetyl- (*CREBBP* [CREB binding protein]) and methyl-transferases (*MLL2* [lysine (K)-specific methyltransferase]), and transcriptional activation/regulation (*CREBBP, SRCAP* [Snf2-related CREBBP activator protein] and *HCFC1* [host cell factor C1]) were strong hubs and highly co-expressed and interconnected with *MAPT* and/or *GRN* (Additional file 2: Table S1a and S20). Even more importantly, we noted that genes such as *MARK2*, *MARK4*, *EP300* and *AKT1*, and *ATN1*, *SGTA*, *CRKL* and *TLE3* not only were co-expressed with *MAPT* and *GRN*, respectively, in frontal cortex and/or temporal cortex, but also that they are PPIs of either tau or GRN proteins: these could be novel functional risk factors to be further investigated in the functional environment. Particularly, *MARK2*, *MARK4*, *AKT1* and *EP300* influence Tau’s phosphorylation and acetylation [29–32], whilst *ATN1* and *TLE3* regulate transcription, and *CRKL* is involved in DNA-binding processes. If the protein interactors of *GRN* support transcription related processes, those of *MAPT* appear to directly influence tau’s activities and function, fostering some intriguing considerations. Tau is classically known for binding and stabilizing microtubules in neuronal soma and axons [33], but it also localizes in their nucleus [34]. Recent data showed that when DNA damage occurs, tau is dephosphorylated and imported into the nucleus to exert protective effect in stressed neurons [35, 36]. Conversely, acetylation was suggested to affect tau’s phosphorylation patterns leading to early events of tau pathology [29]. All this suggests that MAPT-phosphorylation dynamics are highly sensitive and critical in regulating not only i) microtubules homeostasis and ii) aberrant cytoplasmic accumulation of hyper-phosphorylated tau, but also iii) tau’s shuttling between the cytoplasm and the nucleus. Impairment of the latter might have detrimental downstream effect on DNA protection. Our data support tau’s involvement in DNA binding and given evidence for its involvement in DNA protection [35], it follows that tau must hold relevance in protecting neurons from apoptosis and/or aberrant transcription events, thus supporting neuronal longevity. Finally, our data also indicate that tau’s RNA might be target of genes regulating mRNA splicing and gene expression and determining which tau isoform is being expressed; this supports the hypothesis that aberrant regulation of tau splicing/expression might contribute to tau pathology [31]. With this work we are now highlighting potential effectors of tau’s splicing/expression regulation such as the above mentioned *KHSRP* and *SF3A1* (which are splicing regulatory protein), and *CREBBP, SRCAP* and *HCFC1* (which are transcriptional activators/regulators); these factors should be further investigated in the cell-biology setting.

The second biological process was defined by *HLA-DRA* and *CTSC* that clustered together in all assessed brain regions (thalamus, cerebellum and medulla excluded; Fig. 7) within preserved modules indicating immune- and lysosomal-related processes in microglia. Our data support the idea of a synergistic interplay between immune system and degradation processes relying on the activation of immune responses, phagocytosis and lysis in the lysosomes, particularly through: i) response to a stimulus (stress, chemical [interferon-gamma or cytokine] or biotic stimulus); ii) activation and aggregation of immune (microglial) cells; iii) signal transduction (mediated by interferon-gamma, cytokine and antigen receptor [MHC class II protein complexes] and transducer activities), and; iv) phagocytosis and lysosomal processing through endocytic vesicles, phagosomes, and endolysosomes. Interestingly, a number of transcripts highly interconnected with both the *HLA-DRA* and *CTSC* also support these processes, and might be novel functional markers to be further investigated in the cell-biology setting, such as: *CYBB* (cytochrome b-245, beta polypeptide) encodes a critical component of the oxidase system of phagocytes [37] and *DOCK8* (dedicator of cytokinesis 8) encodes a protein involved in neuronal development [38] and immune cells shaping [39]. Furthermore, and probably more importantly, we identified overlap between our transcripts data and protein interactors of HLA-DRA such as HLA-DMB, HLA-DMA and CD74 to be considered for further investigation in the functional environment. These not only appeared to support immune cell (microglia) activation and maturation, cell-cell adhesion and MHC class II complex activity, but also the biology of extracellular vesicles such as exosomes, and that of lysosomes and lytic vacuoles. These data support the results of our GWAS [16] and the idea that immune processes and lysosomal biology are important and likely common elements across different neurodegenerative diseases [17, 40, 41].

The third biological process was defined by *TMEM106B*, *C9orf72*, *VCP*, *UBQLN2* and *OPTN. TMEM106B* was ubiquitous across all assessed tissues (thalamus, cerebellum and medulla excluded; Fig. 7) in various brain cell types (neurons, oligodendrocytes, pyramidal cells) and associated, from a functional perspective, with various protein metabolism processes. Particularly, we found that TMEM106B appears to play a key role in processes supporting protein homeostasis and transport, catabolism and protein secondary changes involving the ER. Interestingly, our data confirm the importance of these processes in neurodegeneration and indicate potential interactors or targets of TMEM106B among the transcripts highly interconnected with *TMEM106B* that should be considered for further investigation in the cell-biology setting, including: *COPB2* (coatomer protein complex, subunit beta 2, an essential protein for Golgi budding and vesicular trafficking), *SERINC1* (serine incorporator 1, a carrier protein involved in lipid biosynthesis in neurons at the ER level) [42], *NRD1* (nardilysin [N-arginine dibasic convertase], a metalloprotease with potential neuropathogenic role) [43] and *TTC37* (tetratricopeptide repeat domain 37, a protein-protein interactor with chaperone activity).

*C9orf72*, *VCP*, *UBQLN2* and *OPTN* were relevant in both the frontal and temporal cortices where they clustered within modules indicating primarily protein catabolic processes; this was also evident for the putamen, thalamus, hippocampus, cerebellum and medulla (Fig. 7). It is relevant to note not only that PPIs data strongly pointed towards ‘protein catabolism’ pathways (mainly driven by VCP, UBQLN2, OPTN and their associated PPIs), but also that our transcripts interconnectivity data indicated further novel potential factors, involved in catabolic processes and interactors of *C9orf72*, *VCP*, *UBQLN2* and *OPTN*, such as: *CAND1* (cullin-associated and neddylation-dissociated 1), a factor involved in ubiquitin ligase network, critical for substrate degradation [44]; *PSMD12* (proteasome 26S subunit, non-ATPase 12) that encodes a subunit of a multi-catalytic proteinase complex, and; *MYCBP2* (MYC binding protein 2, E3 ubiquitin protein ligase) that functions as a E3 ubiquitin protein ligase. All these factors, such as *CAND1*, *PSMD12* and *MYCBP2* (among the interconnected transcripts) as well as *ELAVL1, EIF2B2, CUL2, UBQLN1, NF1, NIPSNAP1, BTRC, ARFGEF2, COPS3, PLAA, CLTA, ANXA7, SEC23A, USP9X, STAM, HSPA13, RAB11A* and *RTN3* (co-expressed transcripts and PPIs of *C9orf72*, *VCP*, *UBQLN2* and *OPTN*; see Table 4 for detailed associations with the spectrum FTD-genes) should be considered for further investigation in the functional environment.

Finally, *CHMP2B*, *FUS* and *TARDBP* not only did not hold major relevance in their respective modules in the frontal and/or temporal cortex, but also such modules either contained very small or very large numbers of co-expressed genes (see Table 2); nevertheless, we assessed their potential biological meaning through functional annotation and pathways analyses (see Additional file 1 pp 4-5 and 7) we found very general (thus negligible) processes for *CHMP2B*, whilst we noted processes mainly associated with the RNA metabolism for *FUS* and *TARDBP*, confirming their involvement in the modulation of RNA processing [45].

## Conclusion

In summary, with this study we further characterize known FTD-genes by providing insight into their regional-specific functional networks and associated biological processes that might be implicated in the pathogenesis of FTD (Fig. 7). Particularly, we: i) show the probable involvement of transcription regulation, chromatin remodelling and DNA protection through the networks of *MAPT* and *GRN*; ii) further support the likely involvement of immune and lysosomal processes through the networks of *CTSC* and *HLA-DRA*, and; iii) confirm implication of protein meta/catabolism through the networks of *C9orf72*, *VCP*, *UBQLN2* and *OPTN*, and *TMEM106B*.

In addition, we also highlight novel potential genetic and/or functional risk factors (Table 4) to be further explored in focused and extended hypothesis driven cell biology work.

As a final remark, our work overall suggests that we are at a point in time in which there is a critical need for a shift in the study of complex traits and diseases from a reductionist ‘gene → pathology’ type of approach to a more holistic ‘gene ↔ networks ↔ pathways’ strategy.

## Methods

### Expression during development and aging across brain tissues

We assessed expression for FTD-genes in different brain areas by means of the Human Brain Atlas (HBA; [46]) and Braineac [47, 48]. Data from HBA allow to assess expression levels in the cerebellar cortex, mediodorsal nucleus of the thalamus, striatum, amygdala, hippocampus, and 11 areas of the neocortex; data from Braineac allow to assess expression levels in ten distinct brain areas such as the frontal cortex, temporal cortex, putamen, thalamus, hippocampus, white matter, cerebellum, medulla, substantia nigra and occipital cortex. We also extracted expression quantitative trait loci (eQTL) data from Braineac for further analysis and characterization of the newly identified potential risk factors for FTD.

### Ethics

Expression data analysed in this study were generated from 101 control individuals was collected by the Medical Research Council Sudden Death Brain and Tissue Bank, Edinburgh, UK [48, 49] and are available in NCBI’s GEO through accession number GSE46706. All samples had fully informed consent for retrieval and were authorized for ethically approved scientific investigation (Research Ethics Committee [REC] number 10/H0716/3).

### Weighted gene co-expression network analysis (WGCNA)

Networks were generated by weighted gene co-expression network analysis (WGCNA) (see [50, 51]) and modules of highly correlated genes were determined in an unsupervised manner based on co-expression patterns in the ten distinct brain areas: frontal cortex, temporal cortex, putamen, thalamus, hippocampus, white matter, cerebellum, medulla, substantia nigra and occipital cortex. Comparatively to previous work (see details in [17, 52]) we generated networks using 19,152 transcripts. We assessed 14 transcripts corresponding to 12 FTD-genes (*MAPT*, *GRN*, *CHMP2B*, *CTSC*, *HLA-DRA*, *TMEM106B*, *C9orf72*, *VCP*, *UBQLN2*, *OPTN*, *TARDBP* and *FUS*; Table 2). *HLA-DRB5* was not present in the Braineac dataset; *RAB38*, *SQSTM1*, and *BTNL2* were excluded before network analysis due to preliminary inclusion/exclusion criteria (see [17]), and; *FUS* had three transcripts (ID3656904, 3656950, and 3656954). We particularly focused on frontal and temporal cortex, the classically affected brain areas in FTD; we also analysed other brain regions such as the putamen, thalamus, hippocampus and white matter (pure and spectrum genes), and the cerebellum and medulla (spectrum genes only), where the FTD-genes were highly co-expressed or were hubs. To identify highly interconnected genes within each module (hubs) we used the measure of module membership (MM), a Pearson correlation between gene-expression level and module-eigengene. An elevated MM (>0.6) suggests strong inter-correlations between genes in a module. We used the 1-quantile measure of the MMs to define inter-modular hubs as any top 10 % gene (i.e. genes with a 1-quantile <0.10). We evaluated whether modules (containing a minimum of two FTD-transcripts) were significantly enriched with FTD-genes using a hypergeometric distribution (with Bonferroni correction for multiple testing) and, to validate modules’ consistency and preservation across tissues, we calculated a composite Z-summary statistic that aggregates different module preservation statistics (Z-summary > 10 = module preservation; Z-summary < 2 = no preservation) [53].

As transcriptome organization in a given biological system is highly reproducible [19], we compared the modules containing FTD-genes to previous WGCNA studies in human brain, gene-markers for cell types, and region-enriched or disease-specific genes using the WGCNA function userListEnrichment [54]. Such lists are referenced in the Additional file 1 and significant overlap (Bonferroni corrected) with each FTD-module is reported in the results section.

### Mantel test and construction of replication network

To assess the reproducibility, and thus replicate, the frontal cortex networks we used the Colantuoni dataset [18] with GEO accession number GSE30272 (Illumina Human 49K Oligo array, HEEBO-7 Set). This comprises 269 prefrontal cortex human samples but we restricted our analysis to 175 (i.e. those with age > 16). We used the pre-processed/normalized data version of such dataset. In order to validate the frontal cortex WGCNA networks we designed two procedures aiming at evaluating their reproducibility at two levels.

On the one hand we wanted to assess whether the WGCNA topological overlap measure (TOM) matrix from the frontal cortex tissue was significantly reproduced in the WGCNA TOM matrix constructed for the Colauntoni dataset. For such purpose we applied a Mantel test [55] with the ape R package, version 3.4: this is a permutation test that, using two squared matrices of the same range (i.e. the two TOMs in this case), calculates a Z-statistic defined as the sum of the pairwise product of the lower triangles of the permuted TOMs. The test then compares the permuted distribution with the Z-statistic observed for the actual data and generates a *p*-value.

On the other hand, for evaluating the quality of each frontal cortex network’s module we constructed a signed WGCNA network on the Colauntoni dataset by using the same method employed for the construction of the signed network from our frontal cortex microarray gene expression profiles with beta value of 11, guaranteeing scale free topology property. The generated WGCNA network had 23 modules with sizes between 174 and 2293 genes. As we wanted to assess the level of replication of our frontal cortex co-expression network on the Colauntoni et al. network we carried out a preservation analysis by using the preservation R function implemented by WGCNA software. For that we focused on the Z-summary preservation estimate which is obtained by the aggregation of various estimates focused on two main aspects. On the one hand, estimates on how interaction patterns of genes within the modules, as they are seen in the reference tissue (i.e. in our case our frontal cortex samples), are preserved in the other tissue (i.e. the Colauntoni network). On the other hand, it includes as well clustering estimates which focus on how the genes which cluster together in the reference tissue maintain that clustering in the other dataset. The Z preservation summary is a mean of both estimates. And the estimates are obtained by a permutation analysis [53].

### Gene set enrichment and pathway analysis with g:Profiler

We performed functional annotation and pathways analysis for the highly co-expressed genes within the FTD-genes containing modules to characterise their biological relevance assessing GO terms for biological processes (BPs), cellular components (CCs) and molecular functions (MFs) by means of the bioinformatics tool gProfiler (accessed in March and April 2015) [56]. We used the whole set of 19,152 transcripts as a tissue specific background and considered significant those GO terms with *p* < 0.05 based on gProfiler’s custom threshold g:SCS [56].

### VisANT visualization

Interconnections between transcripts, defined by topological overlap measures (TOMs), of relevant modules containing FTD-genes were visualized using VisANT [57]. We only showed the strongest interconnected genes by visualizing TOMs greater than specific thresholds indicated for each module (explained in Figs. 2, 3, 4 and 5). We limited our analysis to relevant modules (= modules containing one or more FTD-genes with hub status [1-q < 0.1] and/or module membership [MM] values > 0.5) identified in frontal cortex.

### Protein-protein interaction (PPI) analysis

We searched for currently known protein-protein interactors (PPIs) of the pure and spectrum FTD-genes and compared them with the lists of genes included in the modules containing FTD-genes identified in the frontal and temporal cortices. Briefly, a list of protein interactors associated with each FTD-gene was downloaded from Biogrid [58], IntAct and MINT [59] databases; such databases provide a constantly updated survey of PPIs based on manual curation of peer reviewed literature. We then filtered PPIs manually to remove interactions whose taxid was non-human. OnIy PPIs whose respective genes were expressed in the same modules containing the FTD-genes were considered of relevance based on the convergent evidence that they are: i) expressed in brain; ii) co-expressed with FTD-genes, and; iii) proven protein interactors of FTD-genes.

## Additional files

Additional file 1:
**This file includes additional and complementary text as well as supplementary figures.** (PDF 1444 kb) Additional file 2:
**This file includes additional tables to complement and support the main text.** (PDF 18446 kb)

## Abbreviations

AD: Alzheimer’s disease
AKT1: V-akt murine thymoma viral oncogene homolog 1
ALS: amyotrophic lateral sclerosis
ANXA7: annexin A7
APP: amyloid beta (A4) precursor protein
ARFGEF2: ADP ribosylation factor guanine nucleotide exchange factor 2
ATN1: atrophin 1
BPs: biological processes
BTNL2: butyrophilin-Like 2
BTRC: beta-transducin repeat containing E3 ubiquitin protein ligase
bvFTD: behavioural variant FTD
C9orf72: chromosome 9 open reading frame 72
CAND1: Cullin-associated and neddylation-dissociated 1
CCs: cellular components
CD74: CD74 molecule, major histocompatibility complex, class II invariant chain
CHMP2B: charged multivesicular body protein 2B
CLTA: clathrin, light chain A
COPB2: coatomer protein complex, subunit beta 2
COPS3: COP9 signalosome subunit 3
CREBBP: CREB binding protein
CRKL: V-crk avian sarcoma virus CT10 oncogene homolog-like
CTSC: catepsin C
CUL2: Cullin 2
CYBB: cytochrome b-245, beta polypeptide
DOCK8: dedicator of cytokinesis 8
EIF2B2: eukaryotic translation initiation factor 2B subunit beta
ELAVL1: ELAV like RNA binding protein 1
EP300: E1A binding protein p300
ER: endoplasmic reticulum
FCTX: frontal cortex
FTD: frontotemporal dementia
FTD-17: frontotemporal dementia linked to chromosome 17
FTD-3: frontotemporal dementia linked to chromosome 3
FTLD-FUS: frontotemporal lobar degeneration with FUS pathology
FTLD-tau: frontotemporal lobar degeneration with tau pathology
FTLD-TDP: frontotemporal lobar degeneration with TDP-43 pathology
FTLD-UPS: frontotemporal lobar degeneration with p-62 (ubiquitin-proteasome system) pathology
FUS: fused in sarcoma
GO: gene ontology
GRN: progranulin
GWAS: genome wide association studies
HCFC1: host cell factor C1
HLA: human leukocyte antigen
HLA-DMA: major histocompatibility complex, class II, DM alpha
HLA-DMB: major histocompatibility complex, class II, DM beta
HLA-DRA: major histocompatibility complex, class II, DR alpha
HLA-DRB5: major histocompatibility complex, class II, DR beta 5
HSPA13: heat shock protein family A (Hsp70) member 13
IBMPFD: inclusion body myopathy with early-onset Paget disease and frontotemporal dementia
KHSRP: KH-type splicing regulatory protein
MAPT: microtubule associated protein tau
MARK2: MAP/microtubule affinity-regulating kinase 2
MARK4: MAP/microtubule affinity-regulating kinase 4
MFs: molecular functions
MHC: major histocompatibility complex
MLL2: lysine (K)-specific methyltransferase
MM: module membership
MS: multiple sclerosis
MYCBP2: MYC binding protein 2, E3 ubiquitin protein ligase
NF1: neurofibromin 1
NIPSNAP1: nipsnap homolog 1
NRD1: nardilysin N-arginine dibasic convertase
OPTN: optinuerin
PBD: Paget’s disease of bone
PLAA: phospholipase A2-activating protein
PNFA: progressive non-fluent aphasia
PPIs: protein-protein interactors
PSMD12: proteasome 26S subunit, non-ATPase 12
RAB11A: RAB11A, member RAS oncogene family
RAB38: RAB38, member RAS oncogene family
RTN3: reticulon 3
SD: semantic dementia
SEC23A: Sec23 homolog A, COPII coat complex component
SERINC1: serine incorporator 1
SF3A1: splicing factor 3a, subunit 1
SGTA: small glutamine-rich tetratricopeptide repeat (TPR)-containing, alpha
SQSTM1: sequestosome 1
SRCAP: Snf2-related CREBBP activator protein
STAM: signal transducing adaptor molecule
TARDBP: TAR-DNA binding protein 43
TCTX: temporal cortex
TLE3: transducin-like enhancer of split 3
TMEM106B: transmembrane protein 106B
TOM: topological overlap measure
TTC37: tetratricopeptide repeat domain 37
UBQLN1: ubiquilin 1
UBQLN2: ubiquilin 2
UKBEC: UK human brain expression consortium
UPS: ubiquitin proteasome system
USP9X: ubiquitin specific peptidase 9, X-linked
VCP: valosin containing protein
WGCNA: weighted gene co-expression network analysis
