# Supplementary material for: Frontotemporal dementia: insights into the biological underpinnings of disease through gene co-expression network analysis
Source: Mol Neurodegener. 2016 Feb 24;11:21. doi: 10.1186/s13024-016-0085-4 (PMC4765225; doi:10.1186/s13024-016-0085-4)
Supplement: Additional file 1: — This file includes additional and complementary text as well as supplementary figures. (PDF 1444 kb) [file 13024_2016_85_MOESM1_ESM.pdf]

Supplementary Data

Expression levels during development and adulthood (extended version)

Pure FTD-genes

MAPT

Data from HBA showed high expression rates for *MAPT* in all assessed brain tissues (**Supplementary Figure** [**SF**] **1a**); except for the cerebellum that exhibited a constant pattern, transcripts levels were increasing during the prenatal phase (from 8-11 log_2 intensity levels) and then remained constant after birth and during aging for all tissues (10<log_2<12 intensity levels). This tendency was confirmed by transcription data from Braineac, where brain areas such as FCTX, TCTX and OCTX, together with HIPP, THAL and CRBL exhibited highest expression rates (log_2≤8.5; **SF 1b**). In Braineac the expression levels ranged from a maximum of 8.5 (FCTX) to a minimum of 7.9 (WHMT) log_2 intensity levels, with a 1.5 increase fold between the maximum and the minimum. These data suggest that *MAPT* transcripts are elevated across brain areas (especially FCTX, TCTX, OCTX, HIPP, THAL and CRBL) and that MAPT is important throughout the lifespan of an individual. The FCTX and TCTX are the main brain areas affected in FTD associated with *MAPT* variability, and THAL and HIPP may also be affected; conversely the OCTX and CRBL are generally not affected in FTD, whereas the WHMT, which has recently shown to be affected in FTD [1], exhibits the lowest transcript levels.

GRN

Data from HBA showed high expression rates for *GRN* in all assessed brain tissues, with 8<log_2<10 intensity levels throughout all life stages (**SF 2a**). In Braineac the brain regions showing highest expression (log_2=7.5) were SNIG, WHMT and MEDU, followed by the HIPP, THAL, FCTX, OCTX and TCTX (that in Braineac had all similar expression rates [log_2~7.4]), whereas there was a substantial decrease in the CRBL (log_2~6.8; **SF 2b**) with a 1.6 increase fold between the maximum and the minimum levels. Also in this case, data from both datasets clearly suggest that GRN appears to be an important factor throughout life. Variability in *GRN* has been associated to some extent with TDP-43 pathology; this signature is identified mainly in the FCTX, subcortical regions and, more rarely, in the HIPP. In all of these brain areas expression rates for *GRN* were similar (log_2~7.4).

CHMP2B

Data from HBA for *CHMP2B* showed high expression that remained almost constant (log_2~9) in all brain tissues during all life stages (**SF 3a**). Only the CRBL revealed a gradual decrease form 10 in the pre-natal phase to log_2<8 in the late stages of life. In Braineac the brain regions showing highest expression (log_2~6.6) were WHMT, MEDU, and SNIG, followed by the THAL, HIPP, TCTX, FCTX and OCTX (log_2~6.2), whereas there was a substantial decrease in the CRBL (log_2~5.4) with a 2.4

increase fold between the maximum and the minimum levels (**SF 3b**). *CHMP2B* variability has been associated with the FTLD-UPS type of pathology, which is mainly affecting the FCTX and other cortical areas and the dentate gyrus which are all brain areas that exhibited similar expression rates for *CHMP2B* ($6<\log_2<6.2$).

RAB38

Data from HBA for *RAB38* showed low expression levels in all brain tissues with a decrease during the pre-natal stage from $\log_2$~6 to below 5, level that then remains constant throughout late pre-natal stages as well as during infancy and adulthood (**SF 4a**). Data from Braineac showed that expression levels for *RAB38* were low ($4<\log_2<4.5$) in all brain tissues; specifically, expression was highest in WHMT and lowest in CRBL (with a fold change of 1.2; **SF 4b**); considering that *RAB38* was associated with the bvFTD subtype [2] a closer look at the FCTX revealed intensity levels $\log_2$~4.4. ; *RAB38* showed rather lower expression rates, potentially implying that RAB38 is a sensitive cellular marker thus, given this peculiar threshold, a sudden change in expression levels might significantly impact cellular homeostasis.

CTSC

Data from HBA for *CTSC* showed high expression levels in all assessed tissues during early development stages and then a decrease in the late pre-natal stage; after birth the levels are moderate and remain constant during aging with highest levels in the THAL ($7<\log_2<8$) and lowest in the CRBL ($\log_2$~6; **SF 5a**). Data from Braineac also showed highest expression in the THAL ($\log_2$~6.8) with a notable 3.8 increase fold compared to the CRBL where expression is lowest ($\log_2<4.8$; **SF 5b**); considering that *CTSC* was associated with the bvFTD subtype [2] a closer look at the FCTX revealed moderate intensity levels ($\log_2<5.6$) throughout life suggesting that CTSC is an important factor at every age.

BTNL2

Data from HBA for *BTNL2* showed low to moderate ($5<\log_2<6$) expression levels throughout development and aging (**SF 6a**). Data from Braineac also showed that *BTNL2* had overall moderate and conserved expression levels across all tissues ($\log_2$~5.6; **SF 6b**), with a 1.2 increase fold between SNIG ($\log_2$~5.7) and CRBL ($\log_2$~5.5). BTNL2 was associated with the entire FTD spectrum (bvFTD, PPA and FTD-MND), thus a closer look at all brain areas affected in FTD (FCTX, TCTX, THAL, WHTM and HIPP) showed moderate expression levels ($\log_2$~5.6) throughout life suggesting that BTNL2 is an important element at every age.

HLA-DRA

Data from HBA for *HLA-DRA* showed relatively low ($\log_2$~4) expression levels during development (pre-natal stages) and in the early stages of life ($\log_2$~5), whereas expression increased in later stages

of adulthood reaching peaks of log_2~8–9 for all tissues, besides the CRBL that showed intensity levels of log_2~7 (**SF 7a**). Braineac showed highest expression rate in MEDU (immediately followed by SNIG and WHMT; log_2~7) and lowest in CRBL (log_2~5), with a fold change of 3.8 (**SF 7b**). *HLA-DRA* was associated with the entire FTD spectrum (bvFTD, PPA and FTD-MND), thus a closer look at all brain areas affected in FTD (FCTX, TCTX, THAL, WHTM and HIPP) showed intensity levels varying between log_2~5.7 (FCTX and TCTX), ~6.1 (THAL), ~6.5 (HIPP) and ~7 (WHTM) suggesting that HLA-DRA is an important element in all brain tissues that are vulnerable in FTD.

HLA-DRB5

Data from HBA for *HLA-DRB5* revealed low expression levels at all stages (pre-natal, during infancy and adulthood) with transcript intensity levels of log_2~4 in all assesse brain tissues (**SF 8**); of note, data from Braineac were not available for this gene. These data suggest that *HLA-DRB5* has in general low expression rates, thus changes in expression levels might be dependent on stimuli and/or signalling involving immune response.

TMEM106B

Data from HBA for *TMEM106B* showed high (log_2~10) expression levels throughout life in all tissues (**SF 9a**); data from Braineac showed robust and consistent expression ($log_2 \leq 7.5$) across all tissues, with a minimal fold change of 1.1 between WHMT (highest expression rate) and SNIG (lowest expression rate) (**SF 9b**). *TMEM106B* is mainly associated with FTLD-TDP and GRN variability and the main affected areas (FCTX, sub-cortex and HIPP) revealed robust constitutive expression levels ($log_2 \leq 7.5$).

Spectrum FTD-genes

Data from HBA revealed that all genes (*C9orf72*, *VCP*, *SQSTM1*, *UBQLN2*, *OPTN*, *TDP-43* and *FUS*) had moderate to high expression rates across all tissues during development and aging (**SF 10-16**) and it was noteworthy that *C9orf72*, *TDP-43* and *FUS* had highest expression levels in CRBL ($8<log_2<9$; $9<log_2<10$; $10<log_2<11$, respectively; **SF 10, 15-16a**) in adulthood. These data were reflected in the Braineac dataset where expression rates for these three genes were highest in the CRBL (log_2~7.4; log_2=7.5; log_2~7.9, respectively; **SF 10, 15-16b**). For the other genes (*VCP*, *SQSTM1*, *UBQLN2* and *OPTN*) data from HB Atlas revealed overall robust expression levels in all tissues ($10<log_2<11$; $7<log_2<9$; $10<log_2<11$; $7<log_2<9$, respectively) and this trend was confirmed in Braineac; of note, expression levels across tissues were homogeneous for *VCP* ($7.7<log_2<7.9$ with a fold change of 1.2 between MEDU [highest expression levels] and CRBL [lowest expression levels]) and *SQSTM1* ($7.3<log_2<7.5$ with a fold change of 1.2 between SNIG [highest expression levels] and PUTM [lowest expression levels]) (**SF 11-14a**). Conversely, there was a slight slope in the expression levels across tissues in Braineac for *UBQLN2* ($7.9<log_2<8.4$) and *OPTN* ($6.3<log_2<6.9$): respectively,

highest expression was seen in FCTX and TCTX with a 1.5 increase fold compared to WHMT (*UBQLN2*) and in TCTX and FCTX with a 1.6 increase fold comparatively to THAL (*OPTN*) (**SF 11-14b**).

Functional annotation of additional modules in FCTX and TCTX

***CHMP2B* – Darkolivegreen module – FCTX**

No significant BPs neither MFs could be identified for this module, whereas for the CCs only three terms were evident indicating the “cytoplasm” ($p=3.76x10^{-4}$) and elements such as “bounding membrane of organelle” ($p=6.81x10^{-3}$; **Supplementary Table** [**ST**] **3**).

Pathway analysis revealed 3 significant terms ($p<0.05$): among these were the “peroxisome” ($p=1.21x10^{-2}$) and “peroxisomal lipid metabolism” ($p=1.8x10^{-2}$; **Table 3**).

Interestingly, enrichment analysis for the WGCNA Brain list provided evidence for overrepresentation of oligodendrocyte markers: “turquoise M9 – Oligodendrocyte” (CTX) ($p=1.13x10^{-9}$), “blue M2 – Oligodendrocytes” (HumanMeta) ($p=2.49x10^{-8}$), and “Oligodendrocyte probable” (Cahoy) ($p=5.19x10^{-6}$).

***FUS* and *TDP-43* – Lightcyan, blue and turquoise modules – FCTX**

Functional annotation analysis for *FUS* (ID3656904) in the lightcyan module showed 18 BPs, 3 CCs and 4 MFs ($p<0.05$) (**ST 12**).

The BPs for this module revealed a number of precise terms: “RNA metabolic process” ($p=9.49x10^{-8}$), “RNA processing” ($p=5.61x10^{-7}$), “gene expression” ($p=8.38x10^{-7}$), “mRNA processing” ($p=2.1x10^{-5}$) and “RNA splicing” ($p=2.58x10^{-3}$). The CCs clearly indicated the “nucleus” ($p=8.65x10^{-7}$). Finally, for the MFs “nucleic acid binding” was the most significant term ($p=5.63x10^{-5}$).

After pathway analysis we found 2 significant ($p<0.05$) terms: “gene expression” ($p=1.57x10^{-2}$) and “spliceosome” ($p=2.52x10^{-2}$; **Table 3**).

The module was enriched for different GO terms associated with “metabolic process and RNA processing”, whereas the only Brain list term found enriched was with “turquoise M14 Nucleus” (p=4.13E-05) from Miller et al. (2010). Similar results were obtained for TCTX.

Then, considering *FUS* identified by the two transcripts (ID3656950 and 3656954) and clustering in the blue module, we only found 1 term, “DNA binding” ($p=8.82x10^{-4}$; **Table 3**), for the MFs.

Pathway analysis indicated 3 significant terms ($p<0.05$), including “generic transcription pathway” ($p=1.45x10^{-11}$) and “gene expression” ($p=2.46x10^{-4}$; **Table 3**).

Finally, *TDP-43* that clustered in the turquoise module revealed 17 BPs, 11 CCs and 12 MFs ($p<0.05$; **ST 13**).

Among the most significant BPs we found various terms, including “sensory perception of chemical stimulus” ($p=4.76x10^{-8}$) and “sensory perception” ($p=2.47x10^{-7}$). Among the CCs several terms indicated the “extracellular space” ($p=8.68x10^{-10}$), including the “extracellular matrix” ($p=9.57x10^{-7}$); finally, the MFs showed “G-protein coupled receptor activity” ($p=4.79x10^{-11}$) and “transmembrane signaling receptor activity” ($p=4.89x10^{-10}$) among other signal transduction terms.

Pathway analysis revealed up to 15 significant ($p<0.05$) terms. These were variable and difficult to interpret for association with brain disorder.

Of note, both the turquoise and blue modules include a high number of co-expressed genes (4759 and 3329, respectively); this feature creates a noisy environment, which, to some extent, represents a limitation in our analysis of *FUS* and *TDP-43*.

***MAPT* – Lightyellow module MAPT – TCTX**

The functional annotation analysis for this module revealed up to 24 BPs, 11 CCs and 7 MFs with $p<0.05$ (**ST 6**).

The most significant term pointed strictly towards transcription processes through “transcription from RNA polymerase II promoter” ($p=1.23x10^{-4}$), which was further supported by “transcription DNA-templated” ($p=2.1x10^{-2}$) and “RNA metabolic process” ($p=3.96x10^{-2}$). When assessing the CCs, two terms were highly significant indicating the “nucleus” ($p=3.69x10^{-5}$) and the “nuclear lumen” ($p=8.95x10^{-4}$). Finally, the MFs highlighted one highly significant term: “protein binding” ($p=2.93x10^{-4}$). In addition other interesting terms here were “transcription factor”, “nucleic acid” and “poly(A) RNA binding” ($p=1.42x10^{-2}$, $1.63x10^{-2}$ and $2.98x10^{-2}$).

Pathway analysis through gProfiler indicated 6 terms ($p<0.05$): the most sensible and in line with our functional annotation analysis was “RNA Polymerase II Transcription Elongation” ($p=4.21x10^{-2}$; **Table 4**).

Again, significant overlap “green M10 – Glutamatergic Synaptic Function” (CTX) (p=3.79E-05) was observed.

***GRN* – Cyan module – TCTX**

No significant BPs neither MFs could be identified, whereas there were up to 5 significant CCs ($p<0.05$; **ST 8**). These indicated general terms such as “Intracellular part” ($p=5.64x10^{-3}$) and “membrane-bounded organelle” ($p=3.27x10^{-2}$).

Pathways analysis revealed 7 significant terms associated with this module ($p<0.05$); of note were “signaling by Wnt” ($p=7.61x10^{-3}$) and “lysosome” ($p=1.21x10^{-2}$; **Table 4**).

Also the cyan module was enriched for green M10 GlutamatergicSynapticFunction CTX (9.89E-08), and also for green M5 Mitochondria HumanMeta (p=1.74E-05).

***CHMP2B* – Green module – TCTX**

After functional annotation analysis there were 37 significant BPs, 29 CCs and 8 MFs (p<0.05; **ST 8**).

“Intracellular transport” ($p=8.91x10^{-8}$), including “protein transport” ($p=1.76x10^{-7}$) and “protein localization” ($p=1.97x10^{-5}$) were among the most significant BPs. Other interesting terms were “cytoplasmic transport” ($p=1.31x10^{-6}$), “mitochondrion organization” ($p=3.24x10^{-5}$), “ribosome biogenesis” ($p=4.9x10^{-4}$) and “RNA processing” ($p=5.87x10^{-4}$), particularly indicating “ncRNA metabolic process” ($p=1.35x10^{-3}$) and “rRNA metabolic process” ($p=8.27x10^{-3}$). The most significant CC was “intracellular organelle part” ($p=3.14x10^{-13}$) that was further specified by “cytoplasm” ($p=3.34x10^{-7}$) as well as “nuclear lumen” ($p=4.3x10^{-7}$). In addition there were also terms referring to the “mitochondrion” ($p=3.83x10^{-3}$), and “catalytic” and “ribonucleoprotein complex” activity ($p=7x10^{-4}$ and $1.6x10^{-2}$). Finally, the most significant MFs pointed to “poly(A) RNA binding” ($p=3.27x10^{-9}$) and “catalytic activity” ($p=2.67x10^{-4}$), as well as “transferase” and “protein transporter activity” ($p=2.57x10^{-3}$ and $4.34x10^{-3}$).

Pathway analysis showed up to 9 significant terms (p<0.05). Interestingly, the most significant indicated “antigen processing: ubiquitination and proteasome degradation” ($p=2.3x10^{-5}$), which was supported by two further pathways such as “class I MHC mediated antigen processing and presentation” ($p=1.17x10^{-4}$) and “ubiquitin mediated proteolysis” ($p=2.24x10^{-3}$). In addition, also “chromatin organization” and “chromatin modifying enzymes” ($p=2.4x10^{-4}$, both) were evident (**Table 3**).

Using the Brain list, we obtained among the top results a significant overlap with the “blue M16 – Neuron” module (CTX) (p=8.75E-36), whereas no other significant term was pointing to oligodendrocytes. Looking at the cross-tabulation of module in FCTX vs TCTX, we observed that the darkolivegreen module in FCTX was split into a darkolivegreen module and green module in TCTX, with the darkolivegreen module maintaining the evidence for oligodendrocytes signature (“blue M2 – Oligodendrocytes”, p=1.75E-06). Given that FCTX is the main brain area of interest, and also that CHMP2B showed a higher MM in the FCTX module, we consider more appropriate the oligodendrocytes signature for CHMP2B as a result of this study.

***OPTN* – grey60 module – TCTX**

This module only revealed 2 BPs and 3 CCs, whilst no MFs (p<0.05; not shown).

The BPs indicated “synaptic transmission” ($p=5.37x10^{-6}$) and, more generally “cell-cell signaling” ($p=8.83x10^{-6}$), whereas the CCs showed “membrane part” ($p=1.26x10^{-2}$) and “cell periphery” ($p=2.09x10^{-2}$).

Pathway analysis highlighted 7 potential pathways ($p<0.05$). These were showing elements active in the brain such as "Neuroactive ligand-receptor interaction" ($p=3.64x10^{-3}$).

***FUS* and *TDP-43* – midnightblue, pink and magenta modules – TCTX**

The *FUS* transcript with ID3656904 clustered in the midnightblue module (n=268); 73 BPs, 29 CCs and 10 MFs reached significance ($p<0.05$; **ST 15**).

The BPs indicated clearly "RNA metabolic process" ($p=7.49x10^{-12}$), "gene expression" ($p=1.2x10^{-11}$) and "mRNA processing" ($p=4.15x10^{-8}$). Of note, also "RNA splicing" ($p=5.04x10^{-3}$) was shown. Among the CCs we saw that the major compartment was the nucleus ($p=3.68x10^{-12}$) and for the MFs the main terms were "RNA binding" ($p=3.08x10^{-12}$) and "poly(A) RNA binding" ($p=5.15x10^{-12}$). In addition, of interest was also "transcription factor binding transcription factor activity" ($p=1.16x10^{-2}$).

Pathway analysis identified 12 potential pathways ($p<0.05$). "Gene Expression" ($p=8.33x10^{-5}$) was the most significant followed by "Processing of Capped Intron-Containing Pre-mRNA" ($p=1.3x10^{-2}$), clearly relating to the metabolism of the RNA (**Table 3**).

The *FUS* transcript with ID3656950 clustered in the pink module (n=2979); 13 BPs and 2 MFs were significant ($p<0.05$; not shown), whereas there were no CCs.

The BPs in which *FUS* was involved clearly indicated "nucleic acid metabolic process" ($p=7.5x10^{-8}$) and that was further supported by the MFs "DNA binding" ($p=1.3x10^{-7}$) and "nucleic acid binding" ($p=2.32x10^{-4}$).

The pathway analysis just revealed 3 significant pathways ($p<0.05$) for which the most statistically significant were "Generic Transcription Pathway" ($p=5.68x10^{-11}$) and "Gene Expression" ($p=1.84x10^{-5}$; **Table 3**).

Finally, the *FUS* transcript with ID3656954 and *TDP-43* co-clustered within the magenta module (n=1000). Here 16 BPs, 2 CCs and 16 MFs were significant ($p<0.05$; **ST 16**). *FUS* and *TDP-43* were not among the genes supporting the GO terms in neither category.

For the BPs we noted that "inorganic cation transmembrane transport" ($p=9.53x10^{-6}$) and "ion transmembrane transport" ($p=9.55x10^{-6}$) were the most significant; in addition, we also identified "synaptic transmission" ($p=1.09x10^{-3}$) and "potassium ion transmembrane transport" ($p=4x10^{-3}$). The CCs further supported the previous category revealing "voltage-gated potassium channel complex" ($p=2.86x10^{-2}$) and "potassium channel complex" ($p=3.47x10^{-2}$). Finally the MFs showed "cation transmembrane transporter activity" ($p=3.72x10^{-5}$) and "potassium channel activity" ($p=1.82x10^{-2}$) among others providing consistency to this module. Pathway analysis revealed 3 significant potential pathways ($p<0.05$). These confirmed the functional annotation data revealing "Neuronal System" ($p=3.15x10^{-6}$), "Potassium Channels" ($p=5.85x10^{-3}$) and "Voltage gated Potassium channels" ($p=9.81x10^{-3}$).

Brain areas other than frontal and temporal cortex

Here we report *relevant modules* in brain areas other than frontal cortex and temporal cortex. All statistics and modules' functional annotations described hereafter are summarized in ST 18 and 19, respectively.

Putamen

MAPT and *GRN* co-clustered in the purple module. None of the transcripts was a hub and their MM values were > 0.5; this module was enriched for transcription-related processes. *CHMP2B* and *TMEM106B* were found in the green module: *TMEM106B* was a hub with high MM values, whilst *CHMP2B* among the 25% most interactive genes. This module was associated with cytoplasmic protein catabolism and transport. *HLA-DRA* and *CTSC* were together in the royalblue module: neither transcript was a hub while *HLA-DRA* showed high MM value. This module indicated strong association with immune system processes. *UBQLN2* and *C9orf72* were found together in the brown module: here *UBQLN2* was a hub with high MM value, whilst *C9orf72* had weak statistics. This module associated with vesicle trafficking, proteolysis and protein catabolism in the synapse.

Thalamus

VCP and *C9orf72* co-clustered in the lightyellow module. *VCP* was a hub and *C9orf72* was among the 15% most interconnected genes; both exhibited elevated MM values. This module indicated protein catabolic process through proteolysis and biology of the proteasome, and RNA processing or gene expression related processes.

Hippocampus

CHMP2B and *TMEM106B* were found in the greenyellow module. *TMEM106B* was a hub while both transcripts had MM values > 0.6. This module indicated implication of RNA metabolism, particularly, mRNA splicing. We found *HLA-DRA* and *CTSC* together in the darkgrey module. Neither transcript was a hub, whilst both showed high MM values. This module revealed association with immune system processes. *C9orf72* and *OPTN* co-clustered in the grey60 module: both had MM values > 0.5

but none was a hub. Functional annotation here indicated gene expression and RNA metabolism. *VCP* and *UBQLN2* were together in the black module: both had high MM values and were almost hubs. This module indicated general protein catabolic processes.

White matter

HLA-DRA and *CTSC* co-clustered in the black module. *CTSC* was a hub and *HLA-DRA* was among the 21% most interconnected transcripts; both transcripts exhibited high MM values. This module associated with immune system processes. *TMEM106B* was a hub with high MM value in the tan module that indicated general protein metabolism and transport. *VCP* was a hub with high MM value in the darkturquoise module that indicated association with the biology of the ER-membrane network. *C9orf72* and *UBQLN2* co-clustered in the royalblue module: both had MM values > 0.5, but neither was a hub. Functional annotation pointed to protein modification and organization of the mitochondrion.

Cerebellum

C9orf72 and *OPTN* were found in the purple module: neither was a hub and MM values were barely > 0.5. Overall, this module indicated protein catabolism.

Medulla

C9orf72 and *VCP* co-clustered in the grey60 module. Both had MM values > 0.5, but none was a hub. This module indicated ubiquitin-dependent protein catabolic process and proteasome complex activity.

Supplementary Figures

Supplementary Figure 1

a)

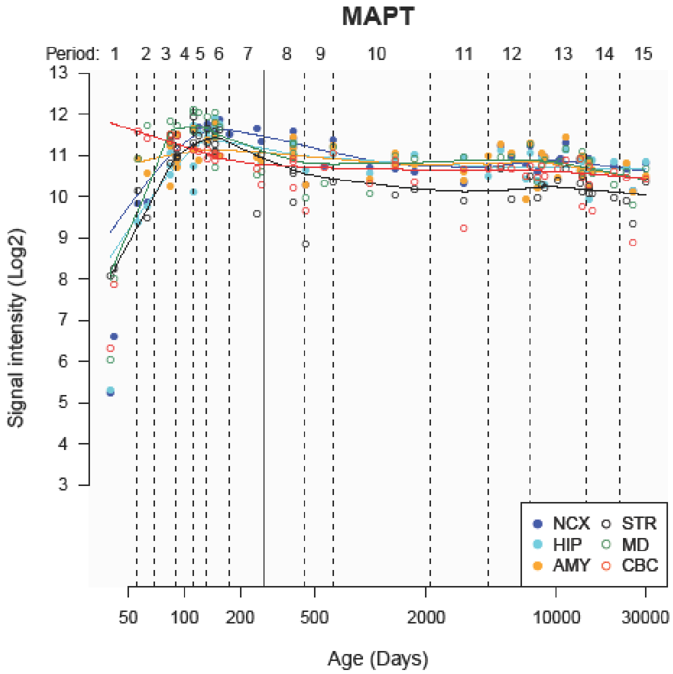

days	500	2000	10000	30000
years	1.37	5.48	27.40	82.19

b)

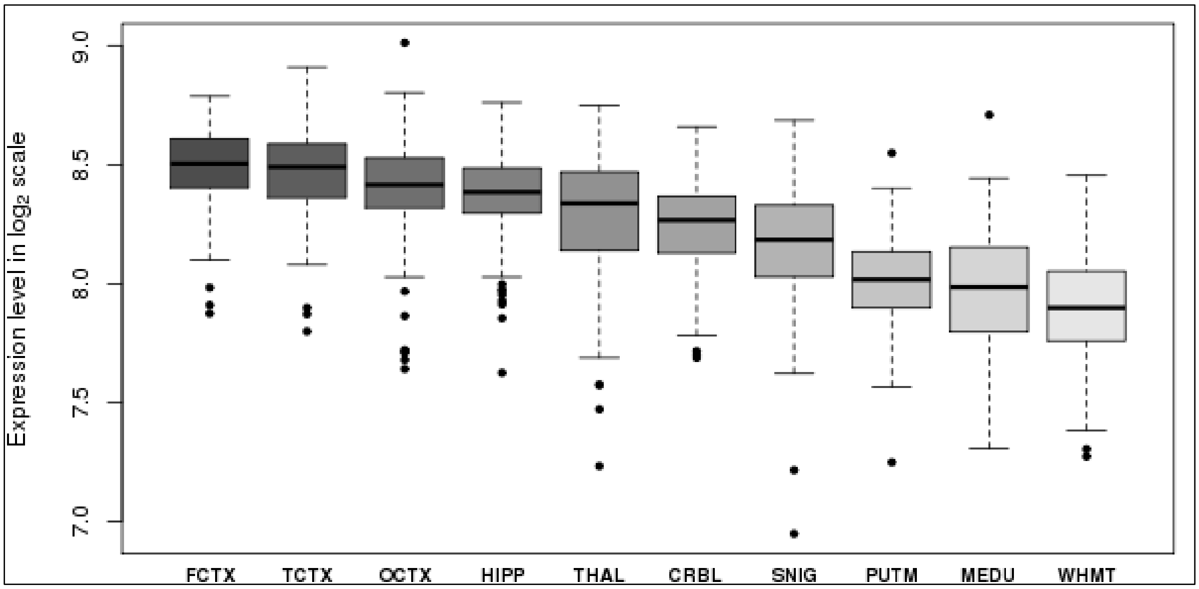

Supplementary Figure 2

a)

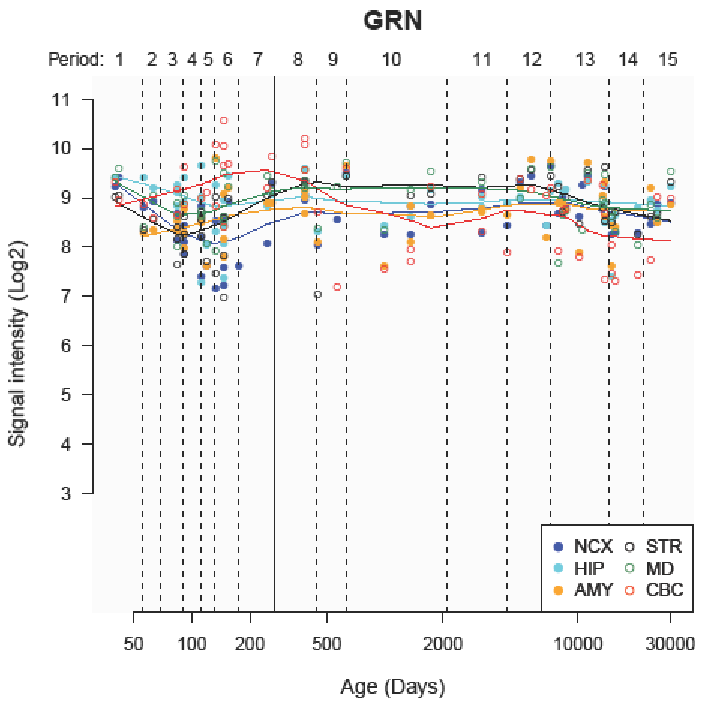

days	500	2000	10000	30000
years	1.37	5.48	27.40	82.19

b)

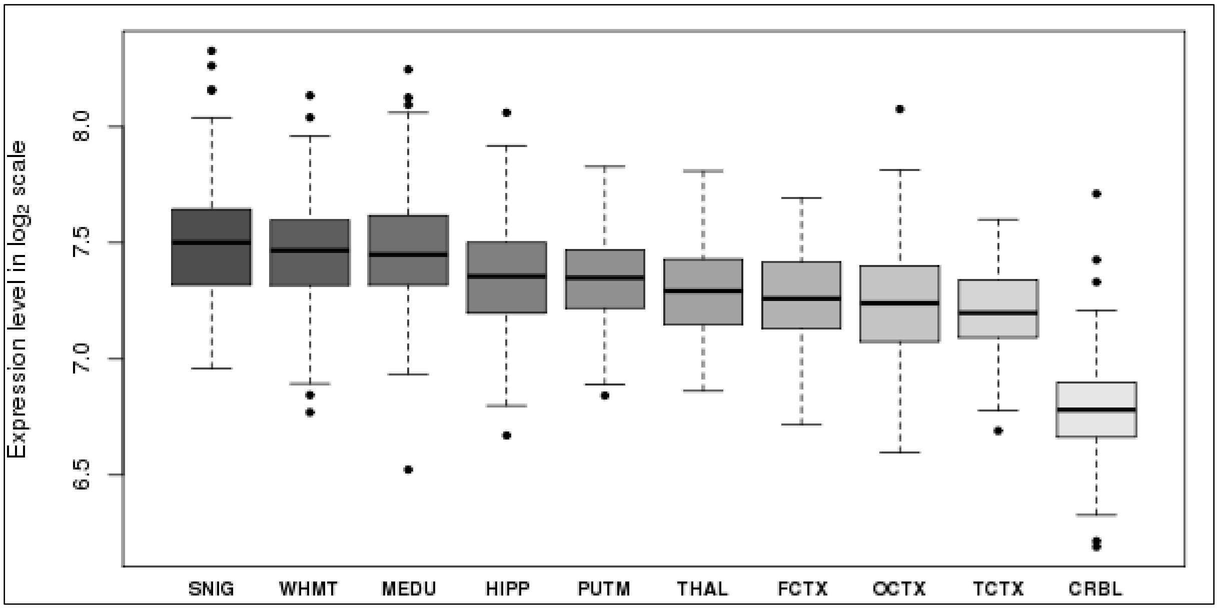

Supplementary Figure 3

a)

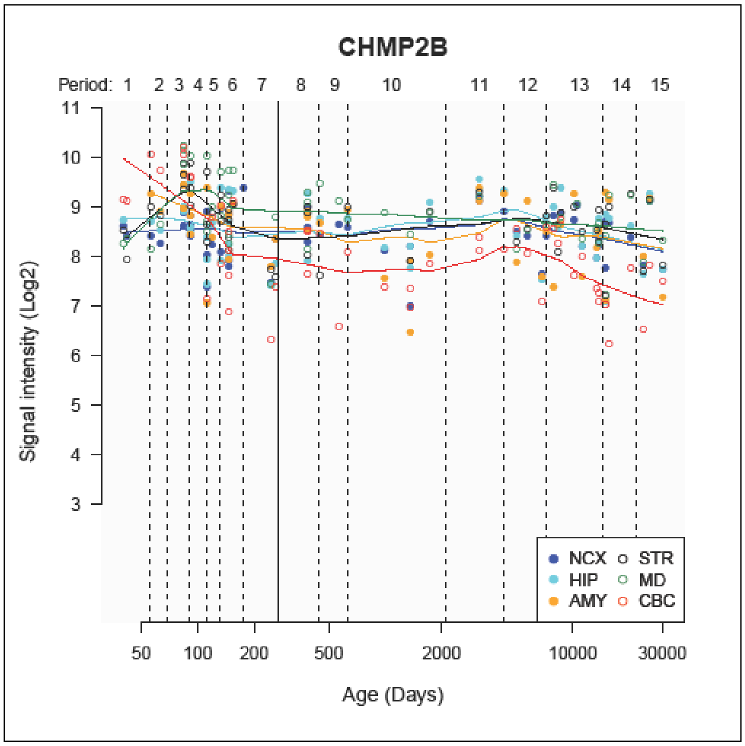

days	500	2000	10000	30000
years	1.37	5.48	27.40	82.19

b)

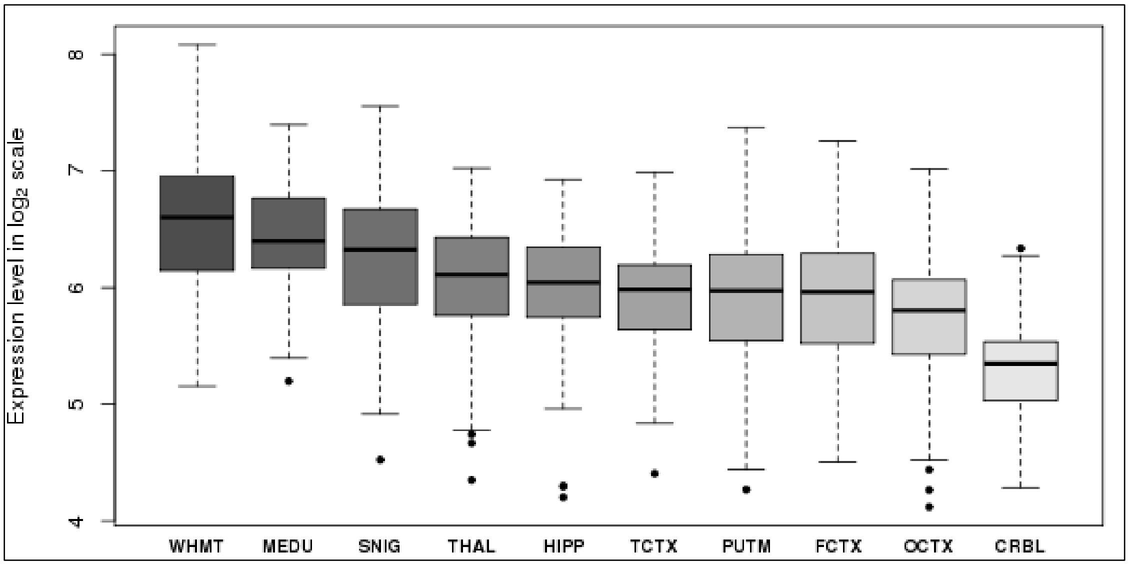

Supplementary Figure 4

a)

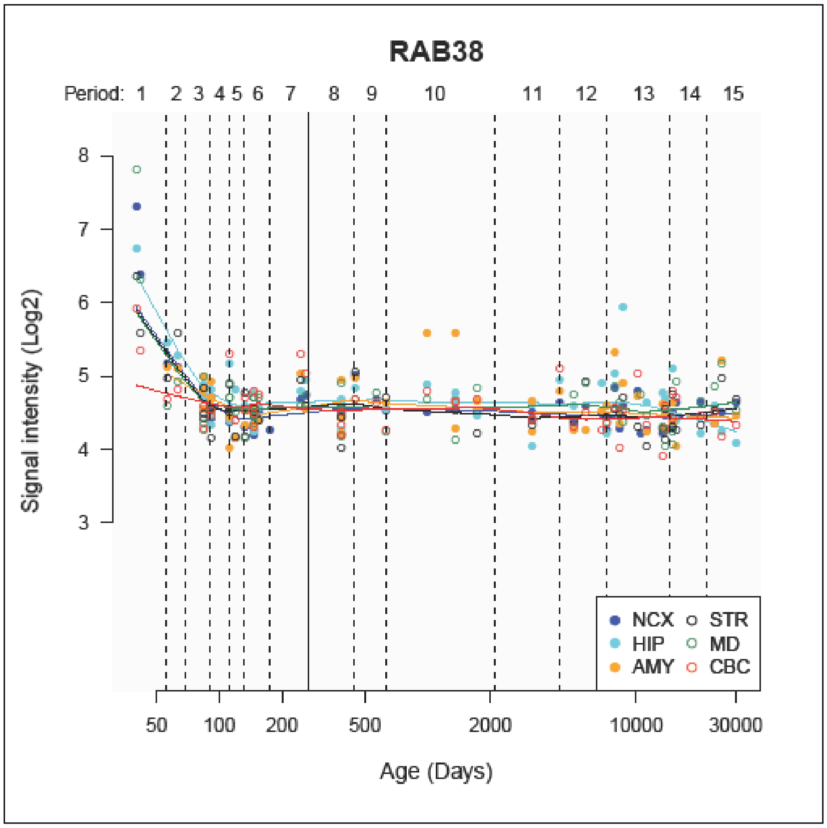

days	500	2000	10000	30000
years	1.37	5.48	27.40	82.19

b)

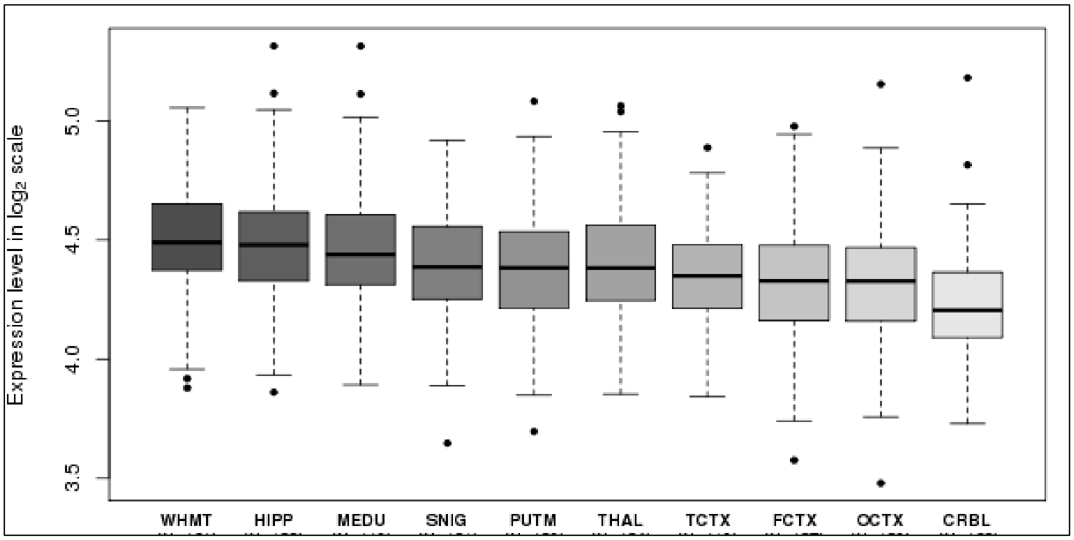

Supplementary Figure 5

a)

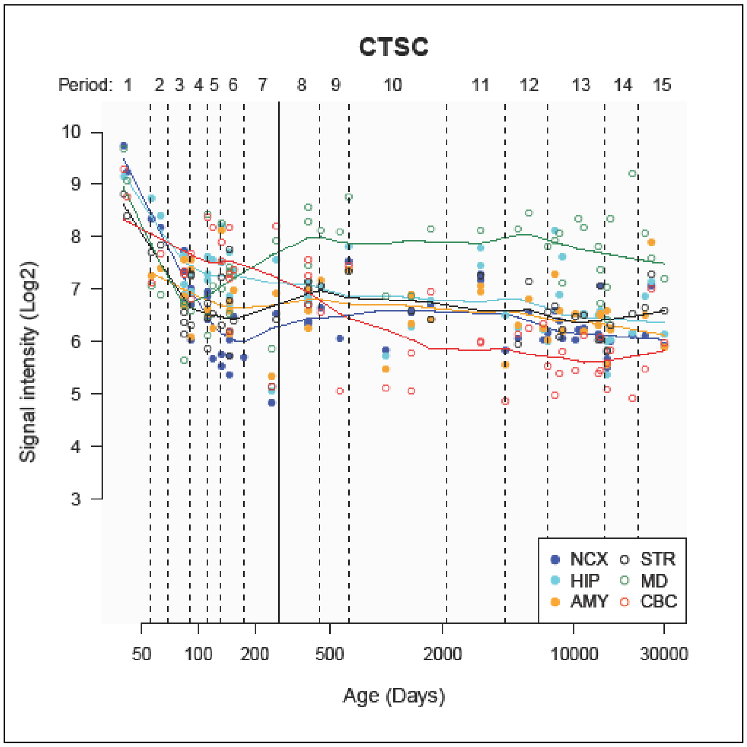

days	500	2000	10000	30000
years	1.37	5.48	27.40	82.19

b)

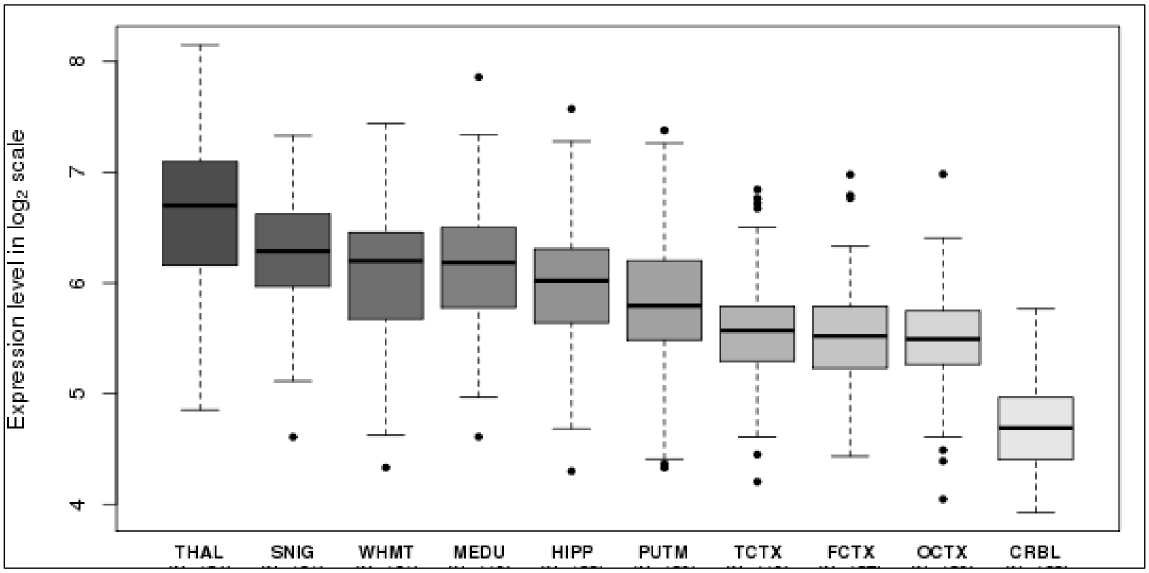

Supplementary Figure 6

a)

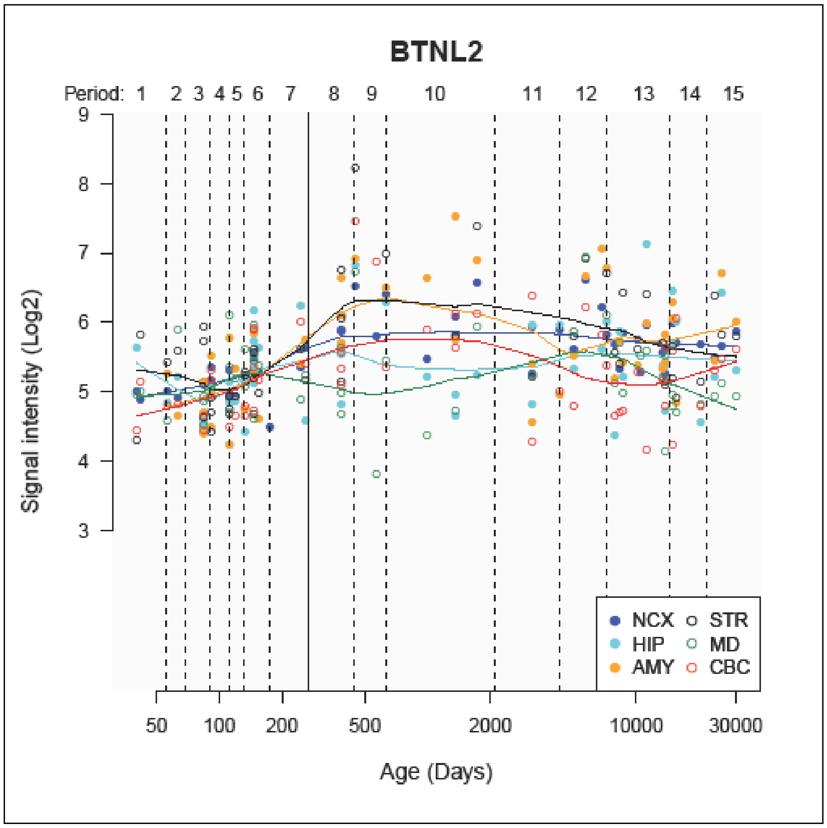

days	500	2000	10000	30000
years	1.37	5.48	27.40	82.19

b)

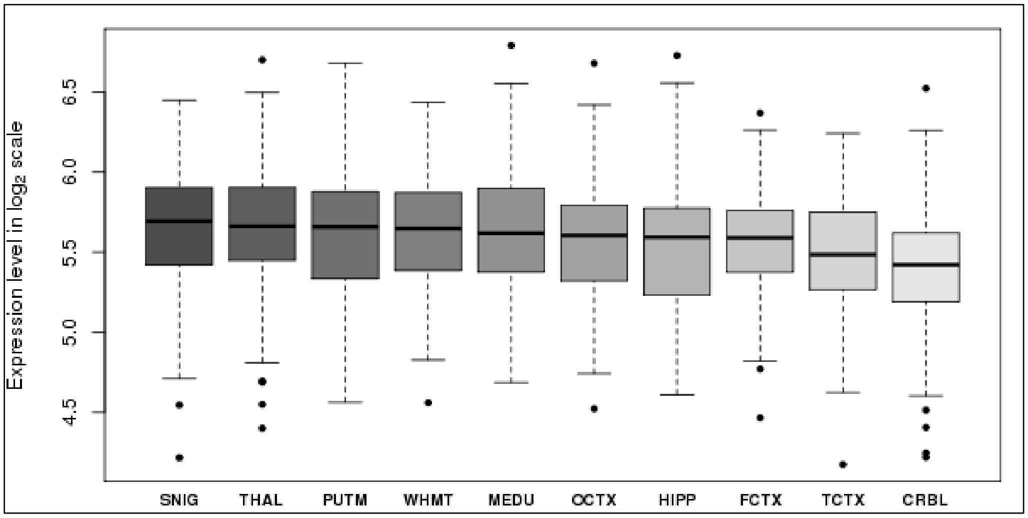

Supplementary Figure 7

a)

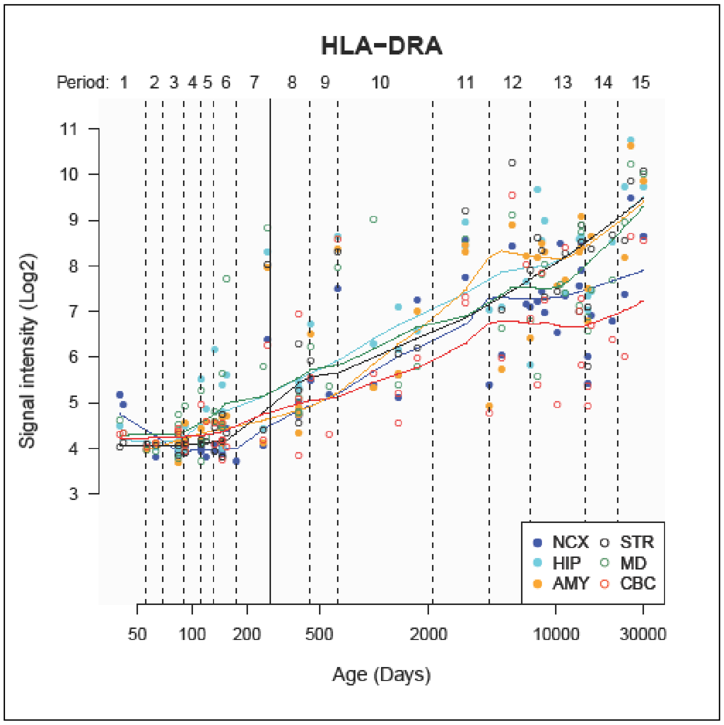

days	500	2000	10000	30000
years	1.37	5.48	27.40	82.19

b)

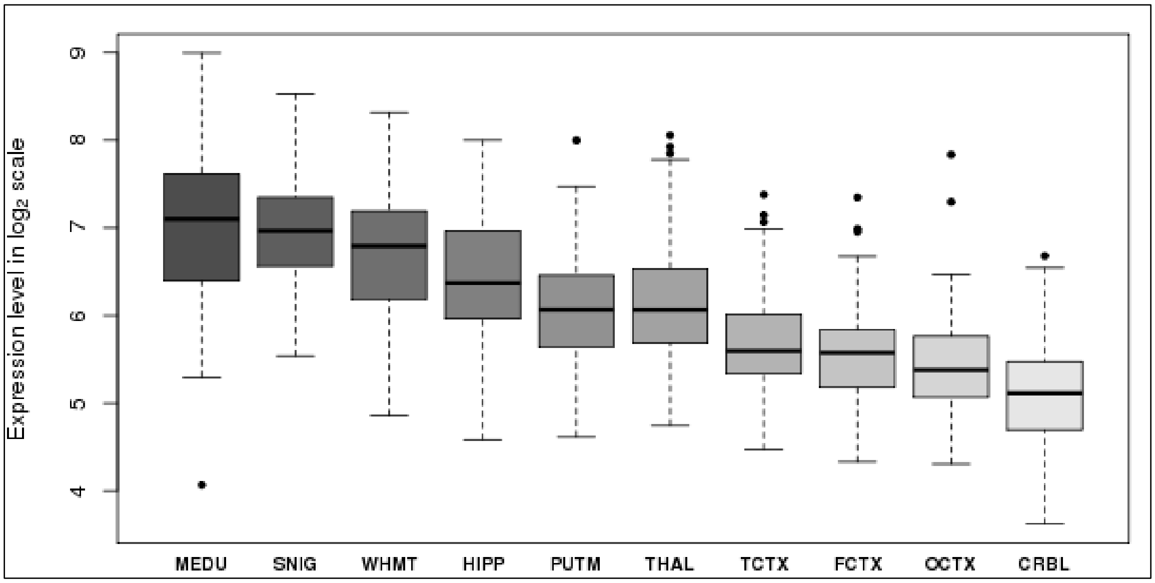

Supplementary Figure 8

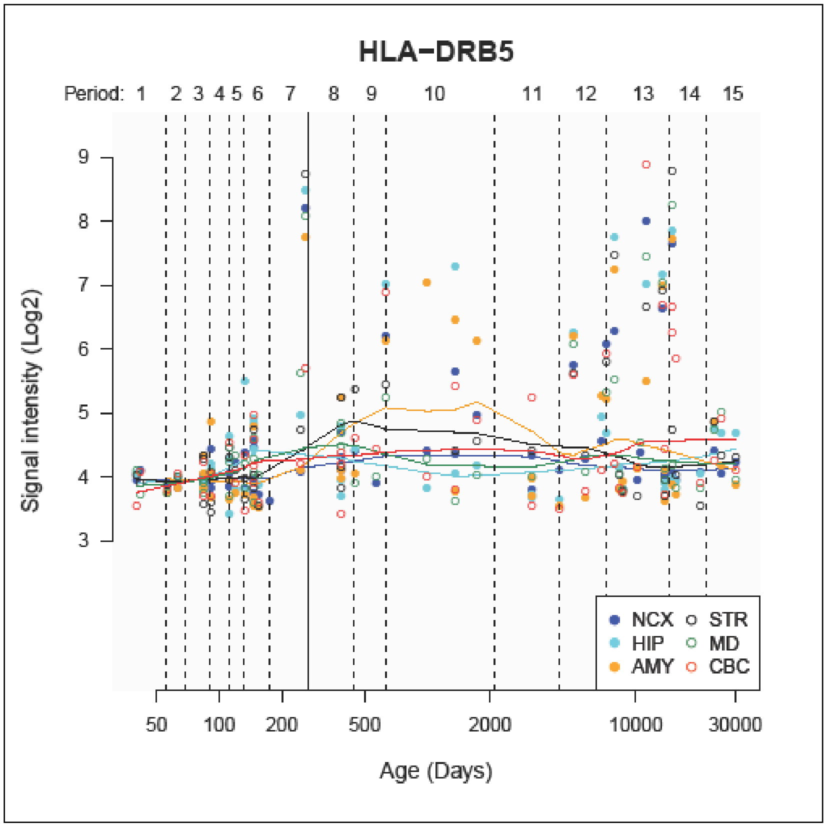

days	500	2000	10000	30000
years	1.37	5.48	27.40	82.19

Supplementary Figure 9

a)

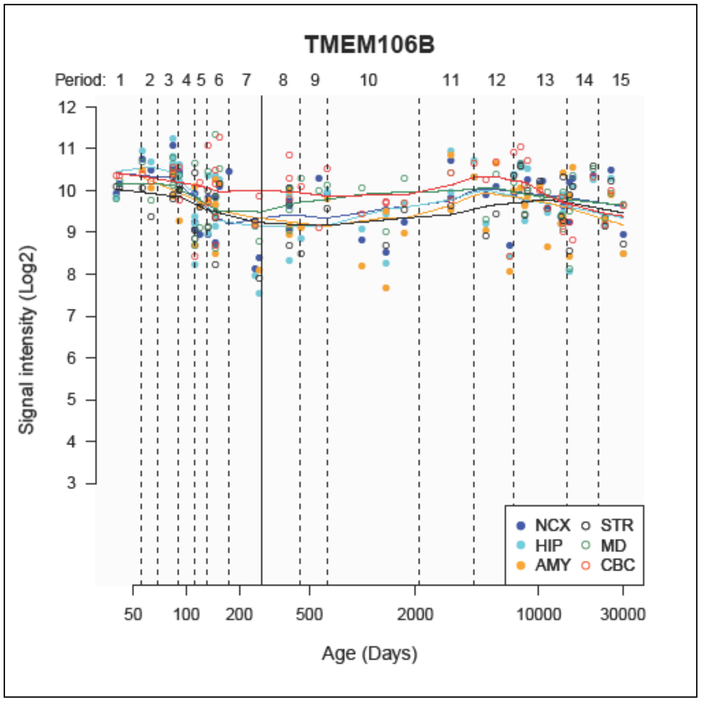

days	500	2000	10000	30000
years	1.37	5.48	27.40	82.19

b)

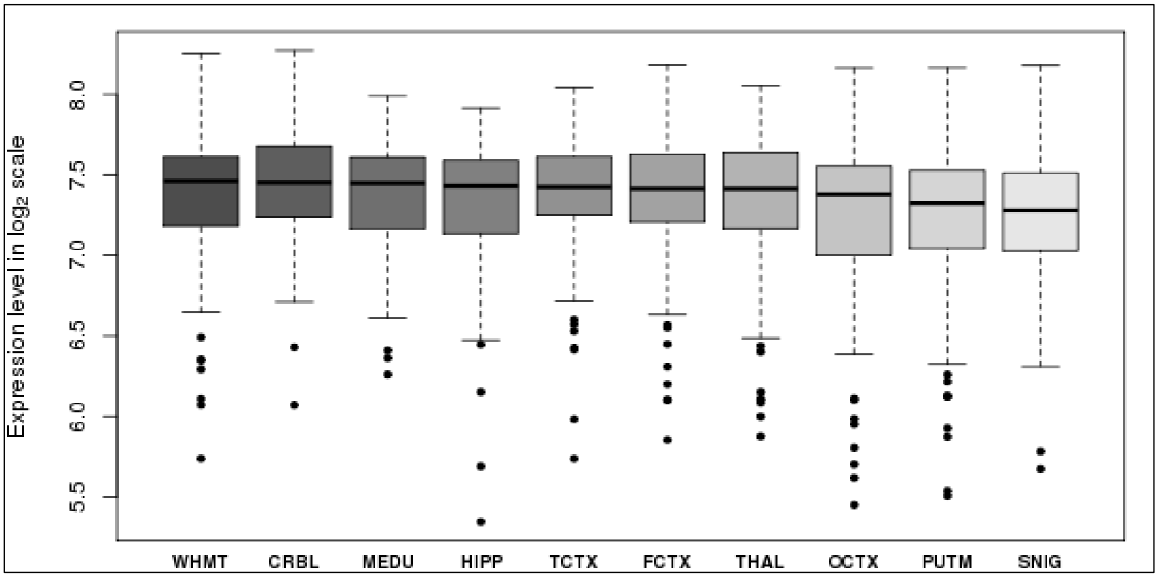

Supplementary Figure 10

a)

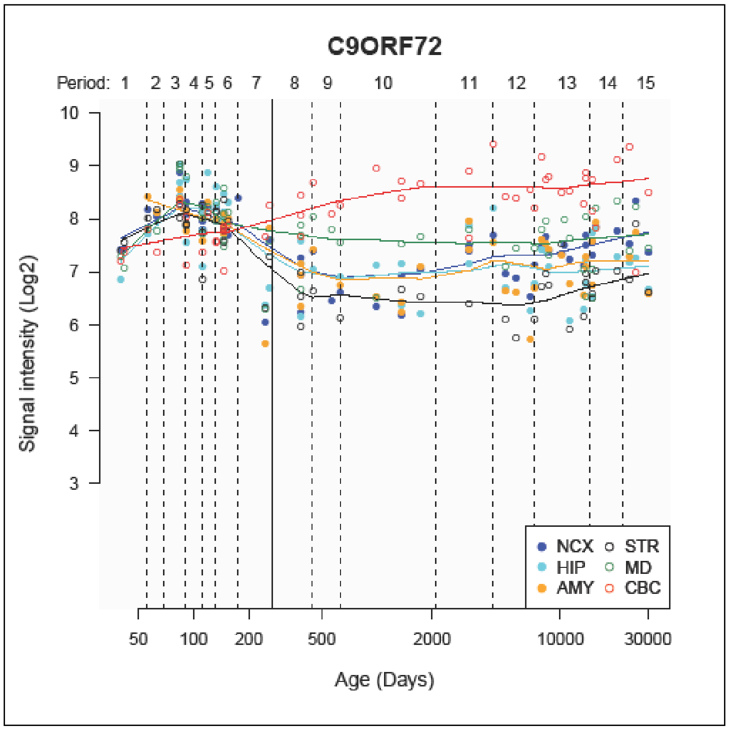

days	500	2000	10000	30000
years	1.37	5.48	27.40	82.19

b)

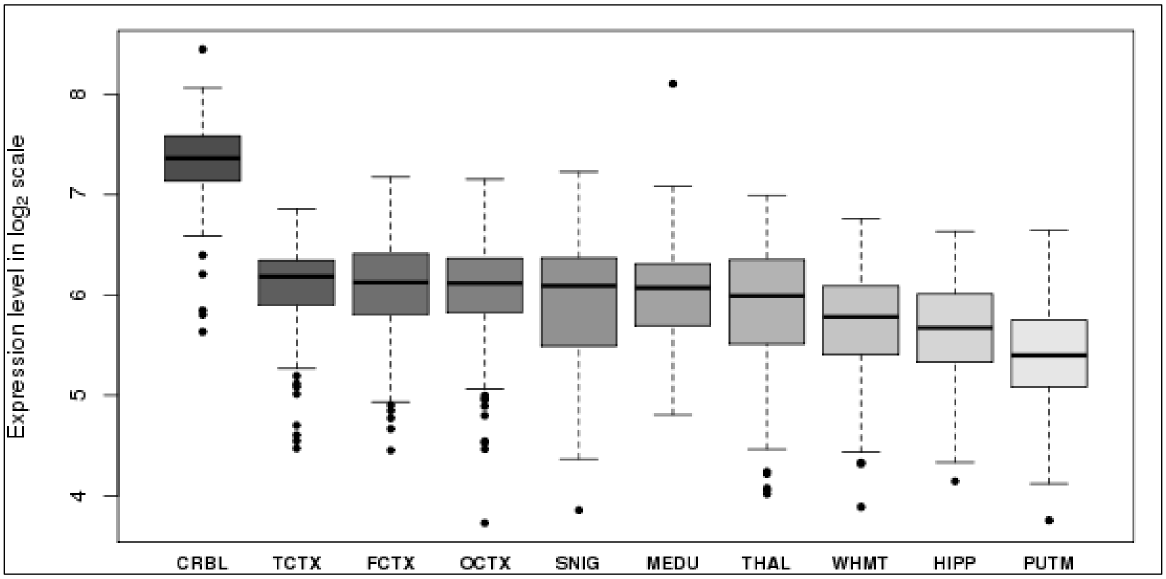

Supplementary Figure 11

a)

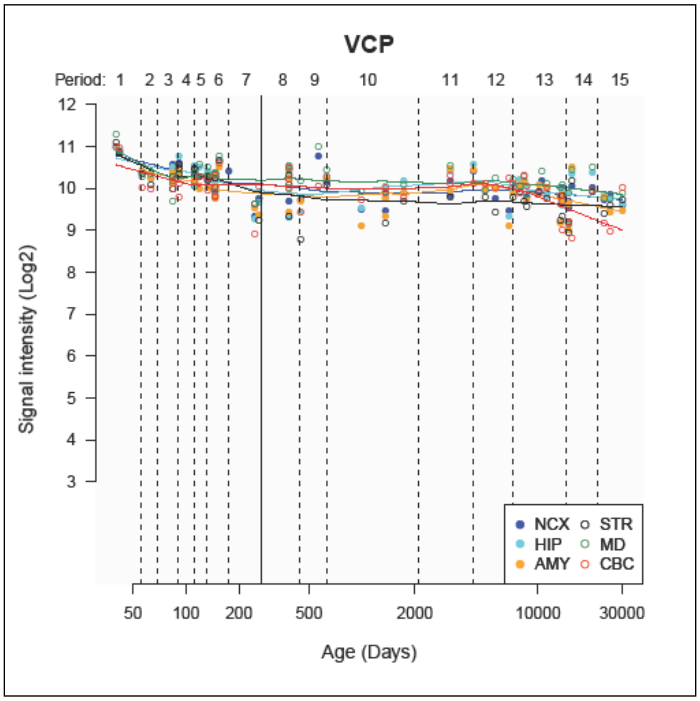

**days**	500	2000	10000	30000
**years**	1.37	5.48	27.40	82.19

b)

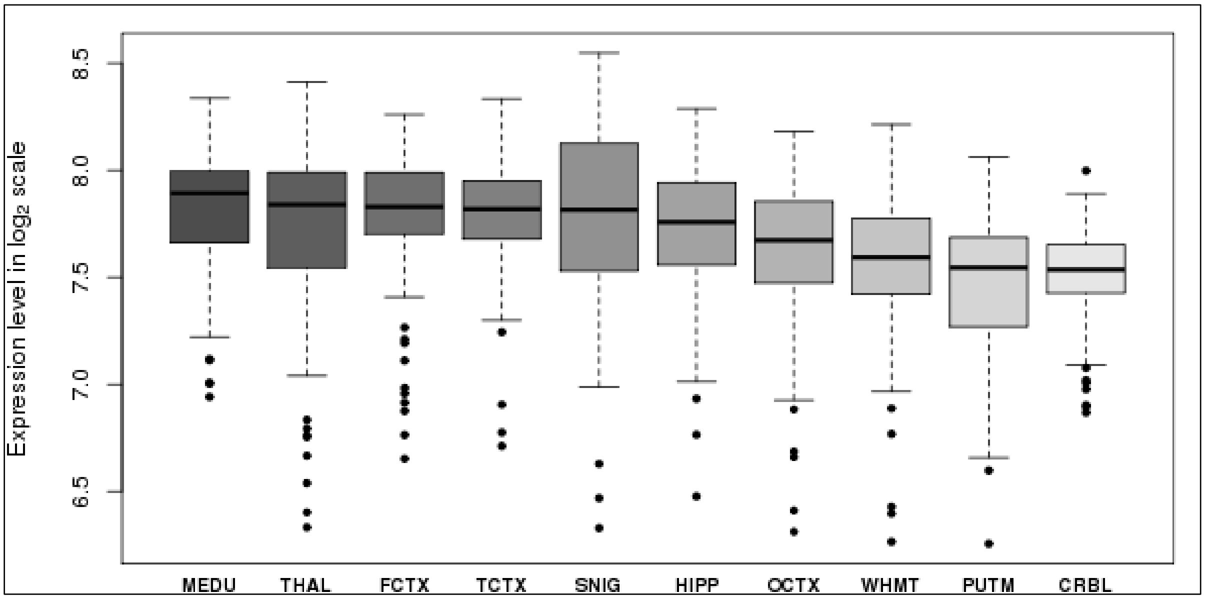

Supplementary Figure 12

a)

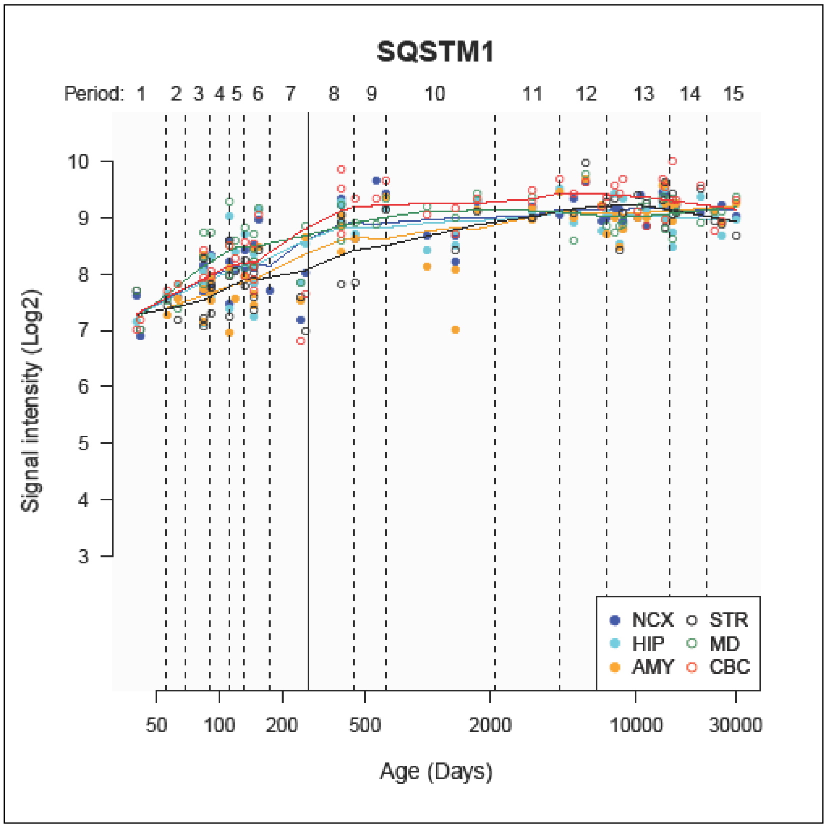

days	500	2000	10000	30000
years	1.37	5.48	27.40	82.19

b)

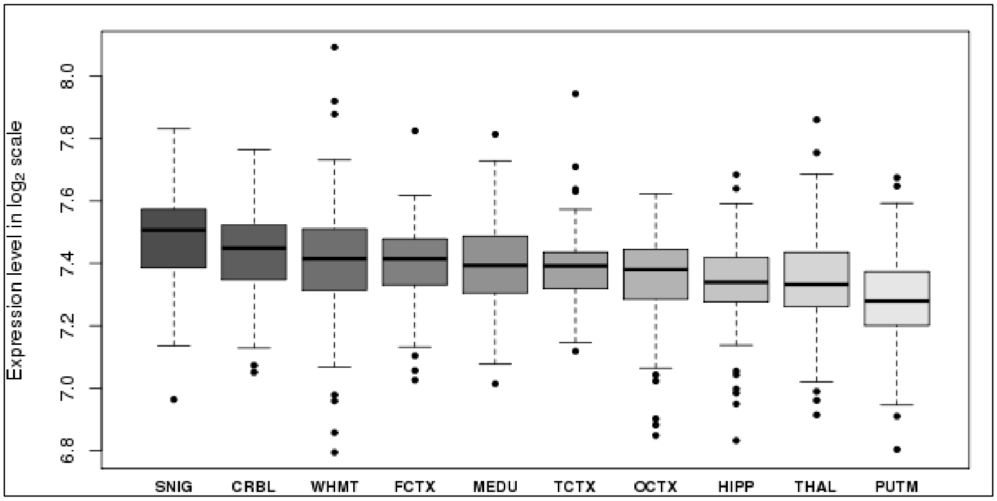

Supplementary Figure 13

a)

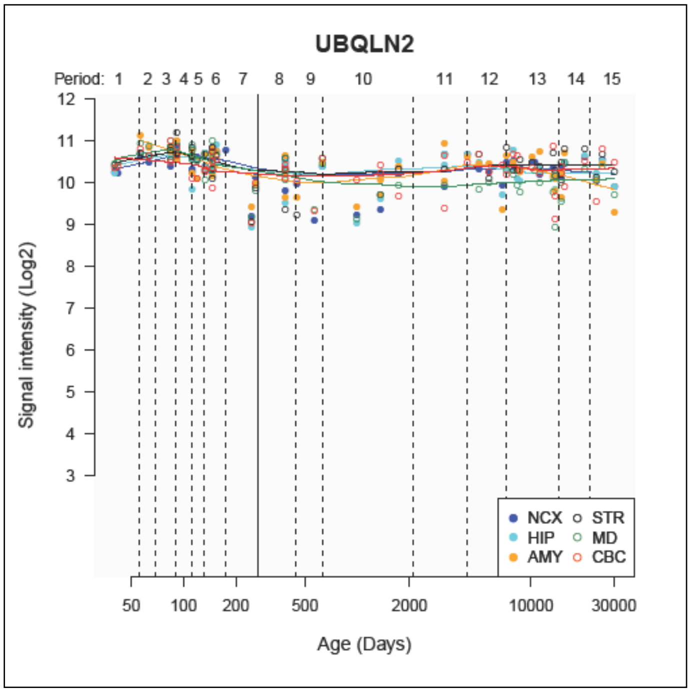

days	500	2000	10000	30000
**years**	1.37	5.48	27.40	82.19

b)

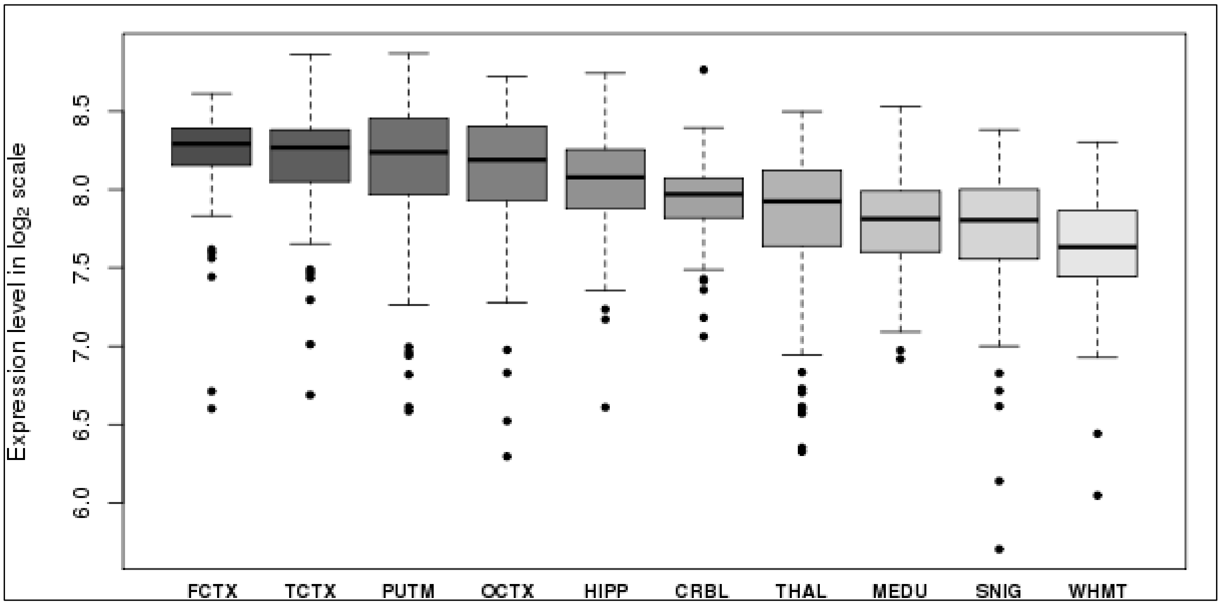

Supplementary Figure 14

a)

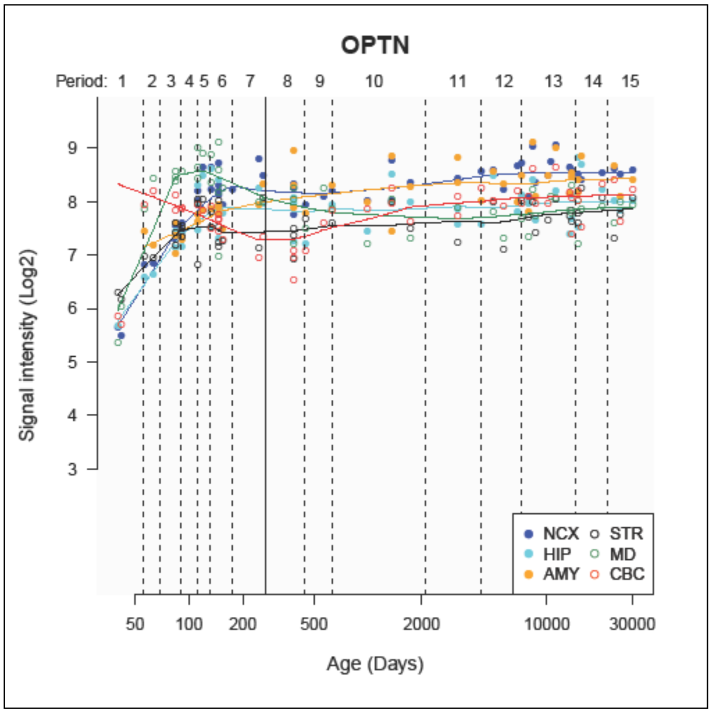

**days**	500	2000	10000	30000
**years**	1.37	5.48	27.40	82.19

b)

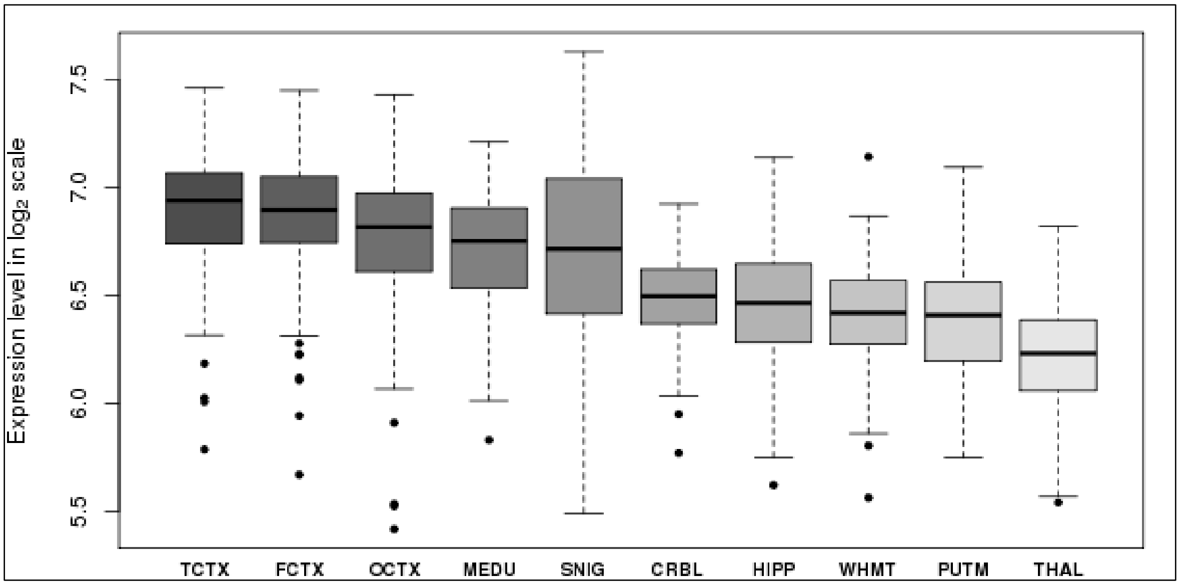

Supplementary Figure 15

a)

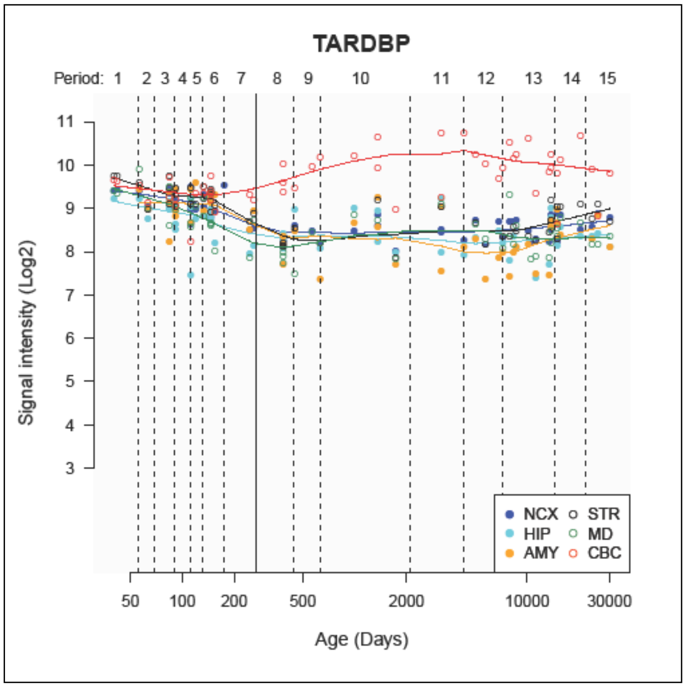

days	500	2000	10000	30000
years	1.37	5.48	27.40	82.19

b)

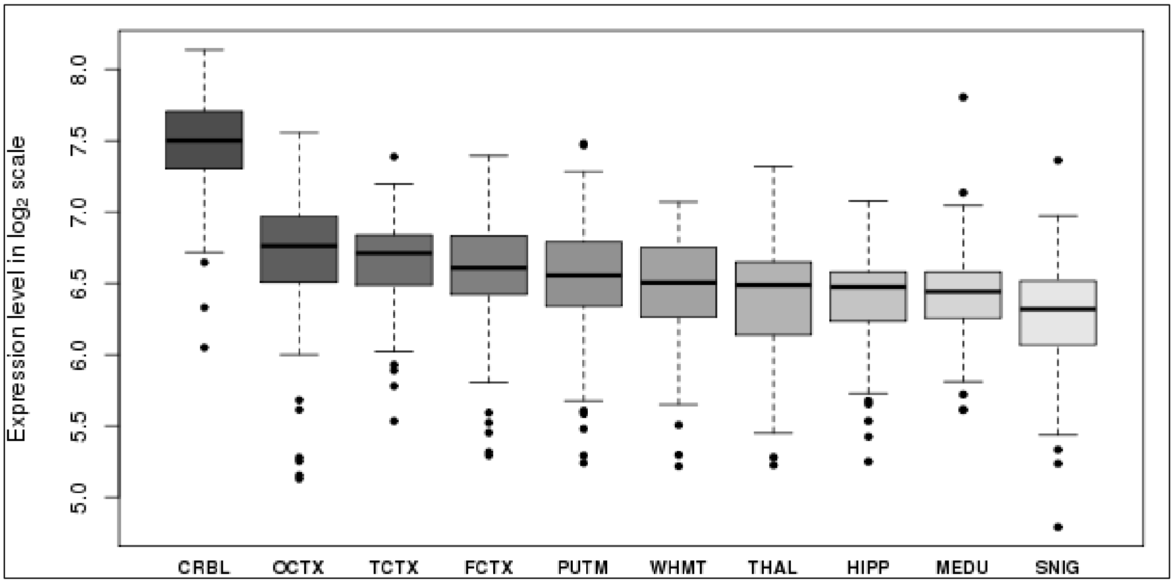

Supplementary Figure 16

a)

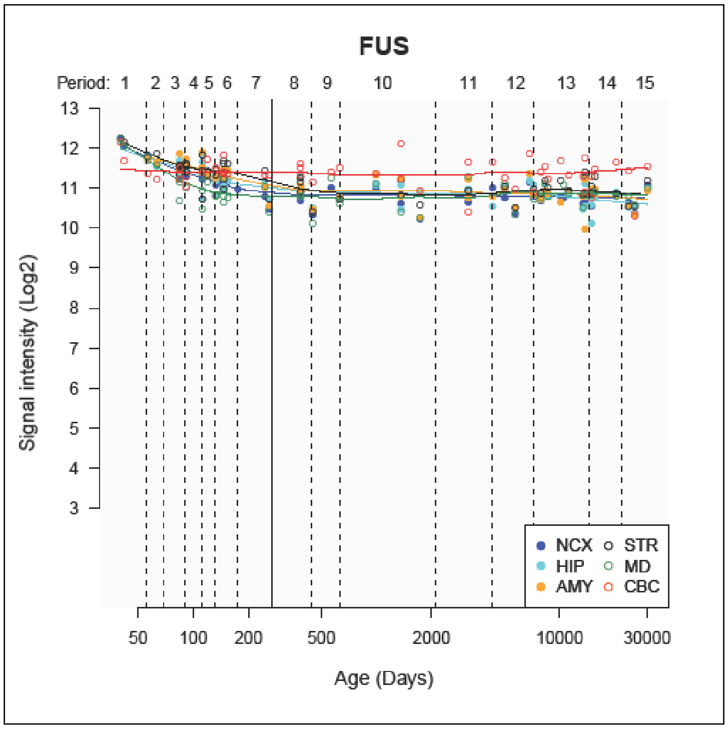

**days**	500	2000	10000	30000
**years**	1.37	5.48	27.40	82.19

b)

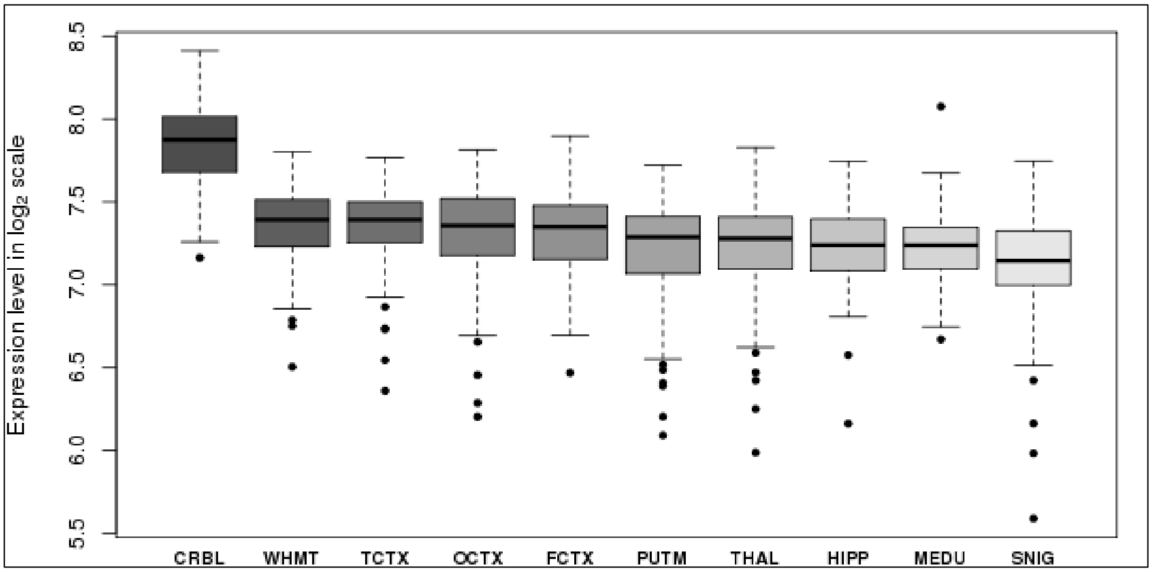

Supplementary Figure 17

Number of genes that fall into modules in FCTX (rows) versus modules in TCTX (columns) (the module colours are not matched). Module overlap is quantified using Fisher's exact test to assign a significance level to each module overlap, which is displayed by a colour scale based on $\log_{10}(p)$.

Genes included in FCTX and TCXT modules were moderately shared: the black module in FCTX (comprising *MAPT* and *GRN*) showed 147 transcripts (including *MAPT*) overlapping with the lightyellow and 127 (including *GRN*) with the cyan modules in TCTX. The darkred module (containing *HLA-DRA* and *CTSC*) had 123 transcripts (including *HLA-DRA* and *CTSC*) shared with the lightcyan module in TCTX. The red module (comprising *TMEM106B*) had 64 transcripts (including *TMEM106B*) overlapping with the darkturquoise and 436 shared with the green modules in TCTX. The purple module (containing *C9orf72*, *VCP*, *UBQLN2* and *OPTN*) showed 577 transcripts (including *C9orf72*, *VCP* and *UBQLN2*) overlapping with the purple module in TCTX.

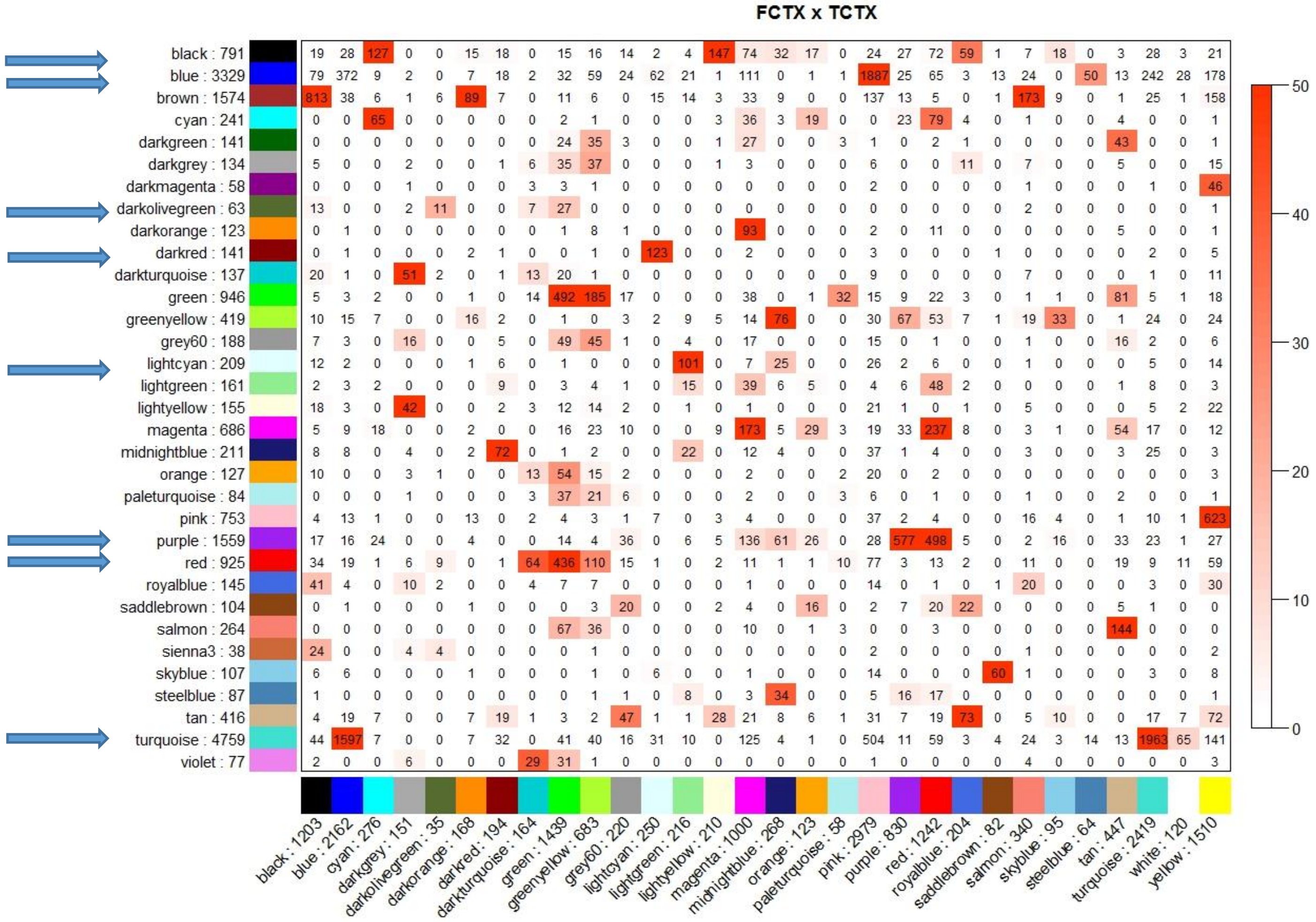

Composite Z-summary statistics for each module in FCTX and TCTX. a) The modules identified in FCTX showed all elevated Z-summary score (>10); this was indicative of moderate to high preservation of each module across tissues. Only the darkolivegreen module was not preserved in PUTM and SNIG. **b**) The modules identified in TCTX showed, for the most, elevated Z-summary score (>10); only the grey60 module was not preserved, and the magenta module had low Z-summary score (<10) exclusively in the PUTM.

a

FCTX modules	size	TCTX	PUTM	THAL	HIPP	WHMT	CRBL	MEDU	SNIG	OCTX	mean
**black**	791	25.93	22.66	16.81	23.26	17.3	22.41	19.68	20.08	32.75	22.32
**blue**	3329	31.86	26.18	22.97	28.45	30.74	25.37	18.41	27.64	27.46	26.56
**darkolivegreen**	63	16.81	**8.33**	11.53	18.37	15.44	15.03	18.5	**8.74**	18.74	14.61
**darkred**	141	31.84	23.5	29.53	38.6	40.86	31.95	38.53	32.3	31.31	33.16
**lightcyan**	209	21.17	17.59	14.78	25.76	28.27	28.72	22.9	11.03	31.67	22.43
**purple**	1559	40.62	25.63	37.56	43.33	27.84	32.01	29.85	44.4	48.18	36.6
**red**	925	28.21	21.47	19.47	23.39	14.43	26.81	23.33	15.41	33.57	22.9
**turquoise**	4759	34.21	42.31	30.72	34.47	34.13	39.46	31.47	30.6	39.24	35.18

b

TCTX modules	size	FCTX	PUTM	THAL	HIPP	WHMT	CRBL	MEDU	SNIG	OCTX	mean
**cyan**	276	25.56	15.31	18.84	17.19	14.43	16.43	15.81	20.36	23.7	18.63
**darkturquoise**	164	27.45	16.05	14.13	16.06	17.49	27.34	14.4	13.06	29.61	19.51
**green**	1439	38.23	26.38	25.43	29.92	24.7	33.96	27.36	24.82	45.3	30.68
**grey60**	220	11.47	10.16	**4.49**	13	**8.58**	**6.22**	**5.61**	**2.25**	24.42	**9.58**
**lightcyan**	250	31.33	18.65	25.8	36.8	39.21	28.51	37.67	32.87	28.81	31.07
**lightyellow**	210	30.88	15.22	11.88	14.5	14.19	13.4	11.62	13.28	26.13	16.79
**magenta**	1000	23.45	**1.41**	15.97	17.15	14.03	11.25	20.24	13.57	26.21	15.92
**midnightblue**	268	19.63	14.01	11.77	18.5	14.1	29.73	11.87	12.01	29.85	17.94
**pink**	2979	32.19	29.44	23.9	26.83	31.42	23.1	20.53	25.17	30.94	27.06
**purple**	830	40.39	32.11	37.78	48.99	24.9	34.36	29.73	44.44	58.59	39.03

References for the pre-defined brain lists (useBrainLists=TRUE, in the userListEnrichment WGCNA function) as described in the manuscript.

Category	References
**ABA**	Cell type markers from: Lein ES, et al. (2007) Genome-wide atlas of gene expression in the adult mouse brain. Nature 445:168-176.
**ADvsCT_inCA1**	Lists of genes found to be increasing or decreasing with Alzheimer's disease in 3 studies:<br>o Blalock E, Geddes J, Chen K, Porter N, Markesbery W, Landfield P (2004) Incipient Alzheimer's disease: microarray correlation analyses reveal major transcriptional and tumor suppressor responses. PNAS 101:2173-2178.<br>o Colangelo V, Schurr J, Ball M, Pelaez R, Bazan N, Lukiw W (2002) Gene expression profiling of 12633 genes in Alzheimer hippocampal CA1: transcription and neurotrophic factor down-regulation and up-regulation of apoptotic and pro-inflammatory signaling. J Neurosci Res 70:462-473.<br>o Liang WS, et al (2008) Altered neuronal gene expression in brain regions differentially affected by Alzheimer's disease: a reference data set. Physiological genomics 33:240-56.
**Bayes**	Postsynaptic Density Proteins from: Bayes A, et al. (2011) Characterization of the proteome, diseases and evolution of the human postsynaptic density. Nat Neurosci. 14(1):19-21.
**Blalock_AD**	Modules from a network using the data from: Blalock E, Geddes J, Chen K, Porter N, Markesbery W, Landfield P (2004) Incipient Alzheimer's disease: microarray correlation analyses reveal major transcriptional and tumor suppressor responses. PNAS 101:2173-2178.
**CA1vsCA3**	Lists of genes enriched in CA1 and CA3 relative to other each and to other areas of the brain, from several studies:<br>o Ginsberg SD, Che S (2005) Expression profile analysis within the human hippocampus: comparison of CA1 and CA3 pyramidal neurons. J Comp Neurol 487:107-118.<br>o Lein E, Zhao X, Gage F (2004) Defining a molecular atlas of the hippocampus using DNA microarrays and high-throughput in situ hybridization. J Neurosci 24:3879-3889.<br>o Newrzella D, et al (2007) The functional genome of CA1 and CA3 neurons under native conditions and in response to ischemia. BMC Genomics 8:370. 4. Torres<br>o Torres-Munoz JE, Van Waveren C, Keegan MG, Bookman RJ, Petito CK (2004) Gene expression profiles in microdissected neurons from human hippocampal subregions. Brain Res Mol Brain Res 127:105-114.<br>o In either Ginsberg or Lein or Torres list.
**Cahoy**	Definite (10+ fold) and probable (1.5+ fold) enrichment from: Cahoy JD, et al. (2008) A transcriptome database for astrocytes, neurons, and oligodendrocytes: A new resource for understanding brain development and function. J Neurosci 28:264-278.
**CTX**	Modules from the CTX (cortex) network from: Oldham MC, et al. (2008) Functional organization of the transcriptome in human brain. Nat Neurosci 11:1271-1282.
**DiseaseGenes**	Probable (C or better rating as of 16 Mar 2011) and possible (all genes in database as of ~2008) genetics-based disease genes from: http://www.alzforum.org/
**EarlyAD**	Genes whose expression is related to cognitive markers of early Alzheimer's disease vs. non-demented controls with AD pathology, from: Parachikova, A., et al (2007) Inflammatory changes parallel the early stages of Alzheimer disease. Neurobiology of Aging 28:1821-1833.
**HumanChimp**	Modules showing region-specificity in both human and chimp from: Oldham MC, Horvath S, Geschwind DH (2006) Conservation and evolution of gene coexpression networks in human and chimpanzee brains. Proc Natl Acad Sci USA 103: 17973-17978.
**HumanMeta**	Modules from the human network from: Miller J, Horvath S, Geschwind D (2010) Divergence of human and mouse brain transcriptome highlights Alzheimer disease pathways. Proc Natl Acad Sci 107:12698-12703.

**JAXdiseaseGene**	Genes where mutations in mouse and/or human are known to cause any disease. WARNING: this list represents an oversimplification of data! This list was created from the Jackson Laboratory: Bult CJ, Eppig JT, Kadin JA, Richardson JE, Blake JA; Mouse Genome Database Group (2008) The Mouse Genome Database (MGD): Mouse biology and model systems. Nucleic Acids Res 36 (database issue):D724-D728.
**Lu_Aging**	Modules from a network using the data from: Lu T, Pan Y, Kao S-Y, Li C, Kohane I, Chan J, Yankner B (2004) Gene regulation and DNA damage in the ageing human brain. Nature 429:883-891.
**MicroglialMarkers**	Markers for microglia and macrophages from several studies:<br>o Gan L, et al. (2004) Identification of cathepsin B as a mediator of neuronal death induced by Abeta-activated microglial cells using a functional genomics approach. J Biol Chem 279:5565-5572.<br>o Albright AV, Gonzalez-Scarano F (2004) Microarray analysis of activated mixed glial (microglia) and monocyte-derived macrophage gene expression. J Neuroimmunol 157:27-38.<br>o Ait-Ghezala G, Mathura VS, Laporte V, Quadros A, Paris D, Patel N, et al. Genomic regulation after CD40 stimulation in microglia: relevance to Alzheimer's disease. Brain Res Mol Brain Res 2005;140(1-2):73-85.<br>o Thomas, DM, Francescutti-Verbeem, DM, Kuhn, DM (2006) Gene expression profile of activated microglia under conditions associated with dopamine neuronal damage. The FASEB Journal 20:515-517.
**MitochondrialType**	Mitochondrial genes from the somatic vs. synaptic fraction of mouse cells from: Winden KD, et al. (2009) The organization of the transcriptional network in specific neuronal classes. Mol Syst Biol 5:291.
**MO**	Markers for many different things provided to my by Mike Oldham. These were originally from several sources:<br>o Genetics-based disease genes in two or more studies from http://www.alzforum.org/ (compiled by Mike Oldham).<br>o Bachoo, R.M. et al. (2004) Molecular diversity of astrocytes with implications for neurological disorders. PNAS 101, 8384-8389.<br>o Foster, LJ, de Hoog, CL, Zhang, Y, Zhang, Y, Xie, X, Mootha, VK, Mann, M. (2006) A Mammalian Organelle Map by Protein Correlation Profiling. Cell 125(1): 187-199.<br>o Morciano, M. et al. Immunoisolation of two synaptic vesicle pools from synaptosomes: a proteomics analysis. J. Neurochem. 95, 1732-1745 (2005).<br>o Sugino, K. et al. Molecular taxonomy of major neuronal classes in the adult mouse forebrain. Nat. Neurosci. 9, 99-107 (2006).
**MouseMeta**	Modules from the mouse network from: Miller J, Horvath S, Geschwind D (2010) Divergence of human and mouse brain transcriptome highlights Alzheimer disease pathways. Proc Natl Acad Sci 107:12698-12703.
**Sugino/Winden**	Conservative list of genes in modules from the network from: Winden K, Oldham M, Mirnics K, Ebert P, Swan C, Levitt P, Rubenstein J, Horvath S, Geschwind D (2009). The organization of the transcriptional network in specific neuronal classes. Molecular systems biology 5. NOTE: Original data came from this neuronal-cell-type-selection experiment in mouse: Sugino K, Hempel C, Miller M, Hattox A, Shapiro P, Wu C, Huang J, Nelson S (2006). Molecular taxonomy of major neuronal classes in the adult mouse forebrain. Nat Neurosci 9:99-107
**Voineagu**	Several Autism-related gene categories from: Voineagu I, Wang X, Johnston P, Lowe JK, Tian Y, Horvath S, Mill J, Cantor RM, Blencowe BJ, Geschwind DH. (2011). Transcriptomic analysis of autistic brain reveals convergent molecular pathology. Nature 474(7351):380-4

References

1. Lam, B.Y., et al., *Longitudinal white matter changes in frontotemporal dementia subtypes.* Hum Brain Mapp, 2014. **35**(7): p. 3547-57.
2. Ferrari, R., et al., *Frontotemporal dementia and its subtypes: a genome-wide association study.* Lancet Neurol, 2014. **13**(7): p. 686-99.
