# Supplementary material for: Frontotemporal dementia: insights into the biological underpinnings of disease through gene co-expression network analysis
Source: Mol Neurodegener. 2016 Feb 24;11:21. doi: 10.1186/s13024-016-0085-4 (PMC4765225; doi:10.1186/s13024-016-0085-4)
Supplement: Additional file 2: — This file includes additional tables to complement and support the main text. (PDF 18446 kb) [file 13024_2016_85_MOESM2_ESM.pdf]

ST 1. Totality of genes/transcripts belonging to the modules containing FTD-genes in Frontal and temporal cortex. All transcripts/genes with 1-quantile <0.09 are hubs.

a. FCTX

Frontal cortex															
Black module		Darkolivegreen module		Darkred module		Red module		Purple module		Lightcyan module		Blue module		Turquoise module	
1-quantile	Gene	1-quantile	Gene	1-quantile	Gene	1-quantile	Gene	1-quantile	Gene	1-quantile	Gene	1-quantile	Gene	1-quantile	Gene
0.00000	**KHSRP**	0.00000	CD164	0.00000	**DOCK8**	0.00000	**COPB2**	0.00000	**DNM1L**	0.00000	UHRF2	0.00000	ENPP3	0.00000	NOTCH4
0.00126	**SEC16A**	0.01587	SLC35A5	0.00709	**NCKAP1L**	0.00108	SNX13	0.00064	**CLTC**	0.00478	VPS13C	0.00030	OR14A2	0.00021	WDFY4
0.00253	**PRR12**	0.03175	TMEM59	0.01418	**FYB**	0.00216	**LOC100131015**	0.00128	**CAND1**	0.00957	BTAF1	0.00060	YIPF7	0.00042	IFRD2
0.00379	**KIAA0664**	0.04762	SNX9	0.02128	**CYBB**	0.00324	SSR1	0.00192	**PSMD12**	0.01435	TTC17	0.00090	LOC285423	0.00063	IL1F7
0.00506	**ATXN2L**	0.06349	RICTOR	0.02837	DOCK2	0.00432	SENP2	0.00257	**AASDHPPT**	0.01914	C21orf66	0.00120	CCDC18	0.00084	SFTPB
0.00632	**SF3A1**	0.07937	RTN4	0.03546	C3AR1	0.00541	**SERINC1**	0.00321	**WDR7**	0.02392	THOC1	0.00150	ADCY10	0.00105	MUC2
0.00759	**CHERP**	0.09524	FXR1	0.04255	**TBXAS1**	0.00649	**DMXL1**	0.00385	**PPM1E**	0.02871	PRPF39	0.00180	LOC199899	0.00126	FAM92B
0.00885	**CREBBP**	0.11111	AGPS	0.04965	LCP1	0.00757	DNAJC13	0.00449	**MYCBP2**	0.03349	ZFC3H1	0.00210	HOXA9	0.00147	RECQL4
0.01011	**TSC2**	0.12698	SGCB	0.05674	ADORA3	0.00865	PNPLA8	0.00513	NOLC1	0.03828	SMC5	0.00240	HFM1	0.00168	CPA1
0.01138	**SRCAP**	0.14286	MTDH	0.06383	PTPRC	0.00973	C4orf41	0.00577	**DMXL2**	0.04306	LUC7L3	0.00270	EZH2	0.00189	GDPD4
0.01264	ACP2	0.15873	X2421271 (SEP15)	0.07092	C7orf58	0.01081	EPRS	0.00641	**ITFG1**	0.04785	RBM39	0.00300	GBP5	0.00210	LOC149086
0.01391	**HCFC1**	0.17460	TRIM2	0.07801	APBB1IP	0.01189	SKIV2L2	0.00706	DENND5B	0.05263	ANKRD10	0.00330	HMCN1	0.00231	CYP2C9
0.01517	PDK2	0.19048	LANCL1	0.08511	ADAM28	0.01297	X2801608	0.00770	CUL2	0.05742	RBM25	0.00360	TMC2	0.00252	KRT3
0.01643	N4BP1	0.20635	FBXL5	0.09220	AIF1	0.01405	**NRD1**	0.00834	**RHOT1**	0.06220	COG3	0.00391	KYNU	0.00273	IL17RE
0.01770	POU6F1	0.22222	SORT1	0.09929	IGSF6	0.01514	ATP13A3	0.00898	ATP6V1D	0.06699	KDM4C	0.00421	GVIN1	0.00294	CPZ
0.01896	SH2D3C	0.23810	CEP97	0.10638	CD68	0.01622	EDEM3	0.00962	UHRF1BP1L	0.07177	ARGLU1	0.00451	C11orf53	0.00315	DHRS4
0.02023	ZNF384	0.25397	GCLC	0.11348	GPR34	0.01730	EXOC2	0.01026	**YME1L1**	0.07656	NEK9	0.00481	STAC	0.00336	FRMD8
0.02149	RPTOR	0.26984	KIAA0776	0.12057	ITGAX	0.01838	**TTC37**	0.01090	ATP6AP2	0.08134	ATM	0.00511	C18orf34	0.00357	NKG7
0.02276	SETD1A	0.28571	GNAI1	0.12766	PLCB2	0.01946	NBAS	0.01155	ATRN	0.08612	NT5C2	0.00541	HELLS	0.00378	ZAN
0.02402	**MLL2**	0.30159	ZNF451	0.13475	CD84	0.02054	STX7	0.01219	ANKRD46	0.09091	EWSR1	0.00571	OR4C3	0.00399	COL6A3
0.02528	ATN1	0.31746	MANEA	0.14184	CIITA	0.02162	PPID	0.01283	**ATL1**	0.09569	INVS	0.00601	BEYLA	0.00420	KRT13
0.02655	CPSF7	0.33333	HSD17B4	0.14894	P2RY13	0.02270	RAB10	0.01347	NDUFA9	0.10048	LOC100287127	0.00631	CCDC73	0.00441	IL17A
0.02781	BRD4	0.34921	TTLL7	0.15603	BLNK	0.02378	MUT	0.01411	CKAP5	0.10526	NPEPL1	0.00661	PIK3C2G	0.00462	PROK1
0.02908	C20orf112	0.36508	PSME4	0.16312	SYK	0.02486	CLCN3	0.01475	RANBP6	0.11005	SFRS5	0.00691	POLE2	0.00483	C9orf171
0.03034	BBS1	0.38095	TNPO1	0.17021	AOAH	0.02595	FBXO38	0.01539	**PDK3**	0.11483	FLJ44342	0.00721	GPR141	0.00504	LYPD4
0.03161	LRFN3	0.39683	C3orf1	0.17730	CYTH4	0.02703	DHX36	0.01604	**UBQLN1**	0.11962	UBR5	0.00751	ADAM7	0.00525	LMX1A
0.03287	FOXRED2	0.41270	LMBRD1	0.18440	SLC2A5	0.02811	RPS6KC1	0.01668	PDHX	0.12440	DKFZp547G183	0.00781	SLC35F4	0.00546	HAL
0.03413	MAPT	0.42857	BNIP3L	0.19149	HPGDS	0.02919	C1orf25	0.01732	SNAPC3	0.12919	NAA16	0.00811	C14orf25	0.00567	C4orf6
0.03540	FAM65A	0.44444	PAPSS1	0.19858	ITGAM	0.03027	PSMD14	0.01796	IARS	0.13397	PUS7L	0.00841	DGKK	0.00588	DNAH12
0.03666	SNORD41	0.46032	RAPGEF5	0.20567	CSF1R	0.03135	GOPC	0.01860	STRBP	0.13876	THOC2	0.00871	C12orf33	0.00609	CD300LF
0.03793	LMTK3	0.47619	CHMP2B	0.21277	PIK3AP1	0.03243	AGGF1	0.01924	ZNF25	0.14354	DDX17	0.00901	BRDT	0.00630	ALDH16A1
0.03919	PSMF1	0.49206	NUDT12	0.21986	TLR7	0.03351	CSDE1	0.01988	C12orf4	0.14833	ZNF280D	0.00931	TMEM99	0.00651	ACOXL
0.04046	ZER1	0.50794	ATP8A1	0.22695	HLA-DRA	0.03459	USP39	0.02053	SEC23A	0.15311	MLLT10	0.00961	LOC81691	0.00672	ABCA12
0.04172	VPS53	0.52381	DHX29	0.23404	ALOX5AP	0.03568	SNX27	0.02117	USP7	0.15789	C12orf29	0.00991	TMEM207	0.00693	IL28RA

0.04298	EPN1	0.53968	USP38	0.24113	LPAR6	0.03676	RASA1	0.02181	CHM	0.16268	WSB1	0.01021	SLC26A5	0.00714	LOC100124692
0.04425	ABCA3	0.55556	WWP1	0.24823	LAPTM5	0.03784	DCUN1D4	0.02245	TMTC3	0.16746	WDR59	0.01051	LOC285638	0.00735	ABCC2
0.04551	HUWE1	0.57143	NAA15	0.25532	C1QB	0.03892	XPO7	0.02309	SACS	0.17225	FNBP4	0.01081	FRAT1	0.00756	BATF2
0.04678	FUT11	0.58730	SUMF2	0.26241	HAVCR2	0.04000	TMEM106B	0.02373	FAM190B	0.17703	MON2	0.01111	NCR3	0.00777	FCN1
0.04804	PCNXL3	0.60317	TICAM2	0.26950	ABCC4	0.04108	RANBP2	0.02437	DPP8	0.18182	DLG2	0.01141	LEKR1	0.00798	LOC731789
0.04930	CLPTM1	0.61905	ENPP4	0.27660	SERPINA1	0.04216	HEXB	0.02502	MTMR7	0.18660	CREBZF	0.01172	MSH4	0.00819	LCE2A
0.05057	RAI1	0.63492	SESTD1	0.28369	TREM2	0.04324	TNPO3	0.02566	**KRT222**	0.19139	CDC16	0.01202	RAG1	0.00841	FCER2
0.05183	CIC	0.65079	CSNK1A1	0.29078	LST1	0.04432	UBA3	0.02630	KRAS	0.19617	WDR11	0.01232	CD96	0.00862	C14orf93
0.05310	LPHN1	0.66667	HBS1L	0.29787	INPP5D	0.04541	DZIP3	0.02694	ACTR10	0.20096	ZNF493	0.01262	GALNT5	0.00883	DLEC1
0.05436	PSMD3	0.68254	ECHDC1	0.30496	C1QC	0.04649	SYT11	0.02758	CDS2	0.20574	SMG1	0.01292	MCOLN2	0.00904	SLC5A9
0.05563	UBAP2	0.69841	MTRR	0.31206	C3	0.04757	FBXO11	0.02822	NF1	0.21053	C10orf137	0.01322	KIAA0087	0.00925	MLPH
0.05689	KDELR1	0.71429	LOC100289616	0.31915	TMEM156	0.04865	DCTD	0.02886	ARL1	0.21531	RUFY2	0.01352	HFM1	0.00946	CLCA3P
0.05815	MEN1	0.73016	GNPAT	0.32624	FCER1G	0.04973	SESN1	0.02951	FBXL20	0.22010	PAN3	0.01382	BAIAP2L1	0.00967	DUOXA1
0.05942	RAB11B	0.74603	MIER3	0.33333	ITGB2	0.05081	UBR3	0.03015	USP9X	0.22488	ZNF782	0.01412	STK31	0.00988	FLJ20712
0.06068	C17orf28	0.76190	CDC42BPA	0.34043	RUNX1	0.05189	PPP1CB	0.03079	RAB11A	0.22967	ANKRD26	0.01442	TNFSF18	0.01009	PAPPA2
0.06195	ATP13A1	0.77778	CCNG1	0.34752	LHFPL2	0.05297	EXOC1	0.03143	ARCN1	0.23445	KDM5A	0.01472	OR5H14	0.01030	RGL4
0.06321	LDOC1	0.79365	LPGAT1	0.35461	CLEC9A	0.05405	WDR41	0.03207	SHOC2	0.23923	CCDC84	0.01502	LOC100129297	0.01051	KRT83
0.06448	C16orf5	0.80952	QARS	0.36170	CSF3R	0.05514	C1orf43	0.03271	KLHDC2	0.24402	KIAA0831	0.01532	ZNF28	0.01072	SPP2
0.06574	MEGF8	0.82540	PTPLB	0.36879	CD37	0.05622	UBR3	0.03335	SLC9A6	0.24880	NARG2	0.01562	UGT2A1	0.01093	TFCP2L1
0.06700	DAGLA	0.84127	SEC62	0.37589	SIGLEC8	0.05730	ANO10	0.03400	FAM160B1	0.25359	HNRNPH3	0.01592	ZNF663	0.01114	SIM1
0.06827	CISD3	0.85714	RAB6C	0.38298	HLA-DMA	0.05838	DCTN4	0.03464	NIPSNAP1	0.25837	PSMC6	0.01622	HSD17B13	0.01135	TBC1D27
0.06953	TRAPPC9	0.87302	PDGFA	0.39007	MNDA	0.05946	FAM98A	0.03528	NAPG	0.26316	NIN	0.01652	ACCN5	0.01156	COLEC11
0.07080	FLOT2	0.88889	TATDN3	0.39716	HLA-DPA1	0.06054	RRAGC	0.03592	NUDT21	0.26794	ZMAT1	0.01682	EGF	0.01177	MUC6
0.07206	NUP214	0.90476	ARRDC3	0.40426	HLA-DMB	0.06162	PDHB	0.03656	RNMT	0.27273	RBMX	0.01712	NCAPG2	0.01198	GAPT
0.07332	SCAF1	0.92063	SH3BP5	0.41135	IRF8	0.06270	ZMPSTE24	0.03720	CNOT7	0.27751	ZNF528	0.01742	CLCA2	0.01219	FAM54A
0.07459	MARK2	0.93651	SQLE	0.41844	IL13RA1	0.06378	C5orf51	0.03784	B4GALT6	0.28230	ZNF518A	0.01772	ZNF165	0.01240	MEGF6
0.07585	MBD1	0.95238	TSGA10	0.42553	LPAR5	0.06486	ZNF148	0.03849	SCYL2	0.28708	MARK3	0.01802	PTPN22	0.01261	C6orf25
0.07712	EIF4ENIF1	0.96825	PLA2G12A	0.43262	FGL2	0.06595	LRRC40	0.03913	PDS5B	0.29187	FLJ31306	0.01832	RIMBP3B	0.01282	IGSF22
0.07838	GGA1	0.98413	SEC62	0.43972	PTPN6	0.06703	ORC5L	0.03977	LOC100131102	0.29665	CEP192	0.01862	GNAT3	0.01303	ADAMDEC1
0.07965	SFRS15			0.44681	TYROBP	0.06811	SLC25A36	0.04041	NDFIP2	0.30144	ZNF226	0.01892	GLT6D1	0.01324	CCDC114
0.08091	RNF208			0.45390	IL18	0.06919	TAX1BP1	0.04105	BTRC	0.30622	ZCCHC6	0.01922	SYCP1	0.01345	LOC286177
0.08217	HGS			0.46099	RGS10	0.07027	HLTF	0.04169	MYO9A	0.31100	ZNF207	0.01953	ZMYM1	0.01366	GLRA4
0.08344	IRGQ			0.46809	MYO1F	0.07135	LNPEP	0.04233	CISD1	0.31579	PHKA2	0.01983	EPC1	0.01387	CLEC1B
0.08470	LDOC1L			0.47518	DHRS9	0.07243	KIAA1109	0.04298	PAFAH1B1	0.32057	FAM48A	0.02013	CHPT1	0.01408	TMEM14E
0.08597	NCOR2			0.48227	SELPLG	0.07351	ZBTB11	0.04362	ATRNL1	0.32536	GOLGA1	0.02043	GPR18	0.01429	LOC729549
0.08723	NOVA2			0.48936	CD86	0.07459	MRPL30	0.04426	SYNJ1	0.33014	FGD6	0.02073	KLF4	0.01450	GATA2
0.08850	ZMIZ1			0.49645	CTSC	0.07568	ME1	0.04490	KIF21A	0.33493	C9orf93	0.02103	FLJ42393	0.01471	TMC4
0.08976	SIN3A			0.50355	CD4	0.07676	SMARCAD1	0.04554	ATP6V0A1	0.33971	LUC7L	0.02133	AOX1	0.01492	PRRG2
0.09102	DEPDC5			0.51064	MPEG1	0.07784	KIAA1109	0.04618	IREB2	0.34450	SPG11	0.02163	SYTL4	0.01513	GPR182
0.09229	BCKDK			0.51773	LY86	0.07892	GDAP2	0.04682	RTF1	0.34928	FAM66C	0.02193	NCRNA00161	0.01534	TROAP
0.09355	TUBG1			0.52482	CPVL	0.08000	DHX9	0.04747	C9orf125	0.35407	ARMCX4	0.02223	C6orf103	0.01555	LTBP2
0.09482	R3HDM2			0.53191	TLR1	0.08108	TSNAX	0.04811	**ATP5B**	0.35885	SCAPER	0.02253	C12orf56	0.01576	LOC63930

0.09608	RNF38	0.53901	CTSS	0.08216	ABCE1	0.04875	BHLHB9	0.36364	MPDZ	0.02283	LOC100129036	0.01597	KLHL33
0.09735	NPRL3	0.54610	P2RY12	0.08324	GPD1L	0.04939	ARFGEF2	0.36842	DYNC2H1	0.02313	BANK1	0.01618	MYH6
0.09861	DLGAP4	0.55319	FCGR2A	0.08432	EXOC8	0.05003	OCRL	0.37321	ZNF532	0.02343	IRX2	0.01639	C17orf99
0.09987	CLIP3	0.56028	HCLS1	0.08541	MAK16	0.05067	ARFGEF1	0.37799	ARHGAP21	0.02373	AMICA1	0.01660	LOC642511
0.10114	WWP2	0.56738	CX3CR1	0.08649	PPIG	0.05131	MUDENG	0.38278	NSUN6	0.02403	LOC440970	0.01681	STAB2
0.10240	X3936984	0.57447	WDFY4	0.08757	LNP1	0.05196	PSME3	0.38756	TIAL1	0.02433	ASB15	0.01702	SERPINA4
0.10367	DUSP8	0.58156	SAMSN1	0.08865	SRP72	0.05260	HPRT1	0.39234	MRE11A	0.02463	LOC339894	0.01723	MUC7
0.10493	ZNF358	0.58865	TFEC	0.08973	RNGTT	0.05324	ACSL4	0.39713	ERCC6	0.02493	CCL4	0.01744	KIF23
0.10619	YPEL3	0.59574	B3GNT5	0.09081	VPS45	0.05388	FBXO34	0.40191	CCDC45	0.02523	LRRC69	0.01765	GPR113
0.10746	FOXJ2	0.60284	B4GALT1	0.09189	KBTBD2	0.05452	FRMPD4	0.40670	KIN	0.02553	PIP5K1P1	0.01786	LOC100294450
0.10872	GPR137	0.60993	MS4A6A	0.09297	EXOC6B	0.05516	TRUB1	0.41148	SMCHD1	0.02583	C14orf53	0.01807	C6orf124
0.10999	PRR12	0.61702	CECR1	0.09405	TTC33	0.05581	EIF2AK4	0.41627	MALAT1	0.02613	NOD1	0.01828	C15orf48
0.11125	ZC3H7B	0.62411	C1QA	0.09514	HDAC2	0.05645	VPS13A	0.42105	TAOK3	0.02643	EMR2	0.01849	APOL4
0.11252	TUBB4	0.63121	OLR1	0.09622	CDV3	0.05709	GHITM	0.42584	MBTD1	0.02673	OR52E5	0.01870	C8A
0.11378	DLG4	0.63830	ATP8B4	0.09730	TMCO1	0.05773	SRP68	0.43062	PPP4R1	0.02704	CATSPER2P1	0.01891	TGM7
0.11504	SARS2	0.64539	MS4A4A	0.09838	SLC30A6	0.05837	ANO5	0.43541	DNAJC9	0.02734	C21orf89	0.01912	LOC202181
0.11631	CIZ1	0.65248	ARHGAP15	0.09946	ATP2C1	0.05901	CMAS	0.44019	XPO4	0.02764	DLEU2	0.01933	ITIH5L
0.11757	KIAA0652	0.65957	ACY3	0.10054	RNF180	0.05965	USP14	0.44498	FBN1	0.02794	C1orf101	0.01954	KRT4
0.11884	DPYSL4	0.66667	UCP2	0.10162	TRIM33	0.06030	RTN3	0.44976	CCDC82	0.02824	LASS3	0.01975	NKX6-3
0.12010	GGA3	0.67376	MAN2B1	0.10270	NAA50	0.06094	COPS3	0.45455	MLH3	0.02854	FRRS1	0.01996	ROR2
0.12137	SETD1B	0.68085	RNASE6	0.10378	CAPZA1	0.06158	UQCRC2	0.45933	SUZ12P	0.02884	ZIM3	0.02017	HSPG2
0.12263	INCENP	0.68794	SP100	0.10486	SOS1	0.06222	SAMM50	0.46411	ZNF337	0.02914	FANCC	0.02038	NCRNA00213
0.12389	PNPLA6	0.69504	SCIN	0.10595	C1orf103	0.06286	PPP3CB	0.46890	KIAA1370	0.02944	C7orf10	0.02059	KRT25
0.12516	CACNG7	0.70213	KIAA1274	0.10703	MMADHC	0.06350	UBE4A	0.47368	SFRS6	0.02974	KIF14	0.02080	LAMB4
0.12642	FAM168A	0.70922	FMNL3	0.10811	ACTR3	0.06414	CCNDBP1	0.47847	SBF2	0.03004	BPI	0.02101	FLVCR2
0.12769	MARK4	0.71631	FCGR3A	0.10919	STX12	0.06479	FBXO3	0.48325	HERC4	0.03034	DNAH14	0.02122	TNNT3
0.12895	UNK	0.72340	CASP4	0.11027	EPC2	0.06543	STAM	0.48804	KIAA1731	0.03064	DNA2	0.02143	LAD1
0.13021	DNAJC5	0.73050	TRIM22	0.11135	PIGK	0.06607	MYO5A	0.49282	CHUK	0.03094	ABCB5	0.02164	GABRR2
0.13148	SH2B1	0.73759	TLR3	0.11243	USF1	0.06671	ATP6V1E1	0.49761	TNRC6C	0.03124	C2orf83	0.02185	CD1E
0.13274	GPS2	0.74468	ARHGDIB	0.11351	C7orf54	0.06735	DLAT	0.50239	CADM1	0.03154	LPAL2	0.02206	TRAF3IP3
0.13401	ADRBK1	0.75177	LOC100132913	0.11459	UBE2J1	0.06799	OAT	0.50718	PCID2	0.03184	ATP6V0A4	0.02227	LOC644762
0.13527	SEMA6B	0.75887	LILRB4	0.11568	KDM1A	0.06863	CSE1L	0.51196	ZNF177	0.03214	CYP39A1	0.02248	ITGA10
0.13654	EP400	0.76596	CD74	0.11676	ACAD9	0.06928	NAP1L3	0.51675	DTWD1	0.03244	SERPINC1	0.02269	LOC100288130
0.13780	NLGN2	0.77305	MYOF	0.11784	DCK	0.06992	AP1G1	0.52153	KCNQ1OT1	0.03274	WDR93	0.02290	WDR62
0.13906	MYADM	0.78014	SIPA1	0.11892	TTC1	0.07056	EXT2	0.52632	ZMYM5	0.03304	FRMD7	0.02311	RBP2
0.14033	MGRN1	0.78723	GIMAP4	0.12000	PGRMC2	0.07120	PLAA	0.53110	STX2	0.03334	NMI	0.02332	MUC5B
0.14159	CNOT3	0.79433	RNASET2	0.12108	WDFY1	0.07184	CPEB3	0.53589	TUG1	0.03364	HERC5	0.02353	LOC645645
0.14286	FOXK1	0.80142	C12orf75	0.12216	ADAM9	0.07248	USP15	0.54067	EPC1	0.03394	MORC1	0.02374	EPHA1
0.14412	MAMLD1	0.80851	SPI1	0.12324	RSBN1	0.07312	VPS35	0.54545	CCDC41	0.03424	LIPJ	0.02395	OTOA
0.14539	GRLF1	0.81560	UNC93B1	0.12432	AKR1A1	0.07377	GTF2H1	0.55024	CRYZL1	0.03454	PI4K2B	0.02416	LILRA3
0.14665	FASN	0.82270	TLR10	0.12541	RSBN1L	0.07441	FAM49B	0.55502	INTS2	0.03485	C9orf53	0.02437	ZNF843
0.14791	MBOAT7	0.82979	FCGR1A	0.12649	STT3B	0.07505	AP3S2	0.55981	ATP9B	0.03515	BEND2	0.02458	ASGR1

0.14918	EP300	0.83688	IRAK3	0.12757	TARS	0.07569	LOC729696	0.56459	CYP2R1	0.03545	SCML4	0.02480	PPEF2
0.15044	MAZ	0.84397	HLA-DQA2	0.12865	TRIM23	0.07633	SCAI	0.56938	LOC100287865	0.03575	LOC253724	0.02501	FLJ46358
0.15171	ZBTB45	0.85106	PLEK	0.12973	NCOA1	0.07697	KLHDC5	0.57416	MAGOHB	0.03605	RSPH4A	0.02522	OSGIN1
0.15297	RNF31	0.85816	HLA-DPB1	0.13081	VPS24	0.07761	CLTA	0.57895	CTTN	0.03635	PRSS42	0.02543	C20orf71
0.15424	MNT	0.86525	PTPLAD2	0.13189	CCT7	0.07826	RLIM	0.58373	INTS4	0.03665	ERAP2	0.02564	CEACAM5
0.15550	AMBRA1	0.87234	NFATC2	0.13297	LYSMD3	0.07890	EIF5	0.58852	BIRC2	0.03695	PLK4	0.02585	TMEM40
0.15676	UBE2L3	0.87943	CXCL16	0.13405	RAB1A	0.07954	ANXA7	0.59330	C21orf59	0.03725	PDZD9	0.02606	ARHGAP4
0.15803	PPRC1	0.88652	VSIG4	0.13514	THRAP3	0.08018	NAP1L2	0.59809	ERI2	0.03755	BTN2A2	0.02627	UNC5CL
0.15929	NOC4L	0.89362	PTGS1	0.13622	SNRNP200	0.08082	HSPA13	0.60287	ERCC5	0.03785	MRGPRX4	0.02648	SMTNL2
0.16056	NR1D1	0.90071	CYFIP1	0.13730	RCHY1	0.08146	PITPNA	0.60766	MNAT1	0.03815	GLYATL1P2	0.02669	CD5L
0.16182	FAM89B	0.90780	TAGAP	0.13838	CCT4	0.08210	PI4K2A	0.61244	FUS	0.03845	RNF133	0.02690	KRTAP5-3
0.16308	NMT1	0.91489	GPNMB	0.13946	C6orf120	0.08275	FAM179B	0.61722	RPAIN	0.03875	TAAR1	0.02711	AQP8
0.16435	CMIP	0.92199	SLC7A7	0.14054	PDK1	0.08339	LTA4H	0.62201	KIAA0586	0.03905	FLJ42875	0.02732	LOC730755
0.16561	GLTSCR1	0.92908	DOCK11	0.14162	TMF1	0.08403	APOO	0.62679	STARD9	0.03935	ESR2	0.02753	LOC100288922
0.16688	SLC25A11	0.93617	OLFML3	0.14270	PUM1	0.08467	RPP30	0.63158	C9orf3	0.03965	CFTR	0.02774	EVPL
0.16814	EDC4	0.94326	MAML3	0.14378	FH	0.08531	TRAPPC6B	0.63636	DNHD1	0.03995	SPINK6	0.02795	OTOR
0.16941	ZNF828	0.95035	OAS1	0.14486	C6orf153	0.08595	MAP7D2	0.64115	CNOT2	0.04025	LOC100289954	0.02816	C17orf73
0.17067	ASAP1	0.95745	C8orf34	0.14595	ZBTB41	0.08659	HK1	0.64593	C14orf149	0.04055	ATP6V0D2	0.02837	SLC22A1
0.17193	SLC16A2	0.96454	NAGA	0.14703	SERAC1	0.08724	MKKS	0.65072	ARID2	0.04085	DUS4L	0.02858	HCK
0.17320	TNFAIP1	0.97163	SIRPB2	0.14811	AKIRIN1	0.08788	ZNF280B	0.65550	PIGN	0.04115	HELB	0.02879	KIAA1614
0.17446	SGTA	0.97872	SAMD9	0.14919	AGPAT5	0.08852	C9orf5	0.66029	MALT1	0.04145	C2orf82	0.02900	C13orf34
0.17573	BAZ2A	0.98582	KCTD12	0.15027	PSMA2	0.08916	TM9SF2	0.66507	LOC100287644	0.04175	ACSM3	0.02921	IFNA8
0.17699	BET1L	0.99291	GAB3	0.15135	GCC2	0.08980	NME1	0.66986	ZNF397	0.04205	CNPY1	0.02942	PSMC3IP
0.17826	PCIF1			0.15243	CDC42SE2	0.09044	DCTN5	0.67464	NOL8	0.04236	UTS2	0.02963	UCMA
0.17952	GTPBP1			0.15351	PCGF3	0.09108	UGCG	0.67943	IL1RAPL1	0.04266	TMEM68	0.02984	SH3TC1
0.18078	CDIPT			0.15459	LOC100292648	0.09173	NPEPPS	0.68421	PABPN1	0.04296	ZNF180	0.03005	CPSF4L
0.18205	SPPL3			0.15568	KDM5B	0.09237	PAK1	0.68900	GOLGA8A	0.04326	SLFN13	0.03026	C4BPB
0.18331	FLJ22184			0.15676	RINT1	0.09301	CAMSAP1	0.69378	TAS2R50	0.04356	H2AFZ	0.03047	COL7A1
0.18458	FBRS			0.15784	ZRANB3	0.09365	LEMD3	0.69856	NUP88	0.04386	CARD14	0.03068	C15orf42
0.18584	PHF12			0.15892	UROD	0.09429	EFTUD2	0.70335	ZNF83	0.04416	MIR382	0.03089	COL29A1
0.18710	ATP6V0C			0.16000	EIF2AK2	0.09493	IDH3A	0.70813	MAP3K3	0.04446	DNAH12	0.03110	C1orf125
0.18837	BAT2L1			0.16108	RGL1	0.09557	C9orf72	0.71292	C20orf74	0.04476	HTR3E	0.03131	MYEOV
0.18963	KDM5C			0.16216	CALU	0.09622	UBR1	0.71770	CHKA	0.04506	DNAJA2	0.03152	CCDC27
0.19090	C19orf61			0.16324	MIOS	0.09686	CLCN4	0.72249	BRWD3	0.04536	OGG1	0.03173	SLC5A10
0.19216	CBX6			0.16432	PLRG1	0.09750	IMPAD1	0.72727	SAFB	0.04566	CUZD1	0.03194	NR1H3
0.19343	ERCC2			0.16541	ZNF354A	0.09814	SBNO1	0.73206	RALGPS1	0.04596	LOC100128317	0.03215	XKR3
0.19469	NAT15			0.16649	BRD8	0.09878	AKAP6	0.73684	CEP57	0.04626	HOXB2	0.03236	SLC12A3
0.19595	UBN1			0.16757	SCRN3	0.09942	PRKDC	0.74163	KDELC1	0.04656	RCC1	0.03257	NBPF7
0.19722	C11orf68			0.16865	DNAJB9	0.10006	RNF160	0.74641	PDE7A	0.04686	FSD2	0.03278	MGC24125
0.19848	BCORL1			0.16973	PPP2R2A	0.10071	VPS33A	0.75120	CARS2	0.04716	SMR3B	0.03299	ADAM8
0.19975	C19orf43			0.17081	SSBP2	0.10135	RTN1	0.75598	C11orf61	0.04746	CNR2	0.03320	USH2A
0.20101	KCTD13			0.17189	KIAA1244	0.10199	PRNP	0.76077	ZNF527	0.04776	ANKDD1B	0.03341	CCDC135

0.20228	ZFP64					0.17297	ZNF623	0.10263	HECTD1	0.76555	ZW10	0.04806	PBOV1	0.03362	SPON2
0.20354	SNAPC2					0.17405	RARS	0.10327	CHIC1	0.77033	LOC100287515	0.04836	PML	0.03383	ASPG
0.20480	TMEM184B					0.17514	AP1AR	0.10391	ARHGEF9	0.77512	TSTD2	0.04866	PCBD2	0.03404	SLC22A7
0.20607	KSR2					0.17622	PPA2	0.10455	UBE2W	0.77990	UTP6	0.04896	LOC134466	0.03425	LOC100292090
0.20733	RGAG4					0.17730	UFSP2	0.10520	SLC41A2	0.78469	TAS2R13	0.04926	ESCO2	0.03446	TRPM8
0.20860	NFIX					0.17838	ALDH5A1	0.10584	C20orf177	0.78947	ATP6V0A2	0.04956	C20orf141	0.03467	TNNI1
0.20986	ADCY9					0.17946	HIAT1	0.10648	MDGA2	0.79426	ZNF10	0.04986	GCNT2	0.03488	NKD2
0.21113	C22orf29					0.18054	PPIL4	0.10712	MRPL42	0.79904	ZNF18	0.05017	HOXA2	0.03509	SLC26A6
0.21239	ULK1					0.18162	FAM13B	0.10776	C10orf46	0.80383	RAD51L3	0.05047	NCAPH	0.03530	MUC1
0.21365	FXYD7					0.18270	MRPL19	0.10840	MOAP1	0.80861	JAK2	0.05077	LEAP2	0.03551	SH2D6
0.21492	ZBTB7A					0.18378	SEC22B	0.10904	APPBP2	0.81340	PCBP2	0.05107	TRPC6	0.03572	TP63
0.21618	SEC16A					0.18486	GMCL1	0.10969	TERF2IP	0.81818	CCAR1	0.05137	NOX1	0.03593	MGC10814
0.21745	BSG					0.18595	PI4KB	0.11033	CBARA1	0.82297	HERC2P7	0.05167	MYO3A	0.03614	ACRBP
0.21871	KATNB1					0.18703	NR1I3	0.11097	PSMA4	0.82775	DLG2	0.05197	IDO1	0.03635	LSP1
0.21997	C9orf25					0.18811	ATL2	0.11161	RAB27B	0.83254	HMGN1	0.05227	LOC728095	0.03656	PSD4
0.22124	TAOK2					0.18919	WDR82	0.11225	VPS36	0.83732	KTN1	0.05257	LRRC56	0.03677	XPNPEP2
0.22250	MYBBP1A					0.19027	MCCC2	0.11289	ATG2B	0.84211	RBM17	0.05287	ISL1	0.03698	LOC388692
0.22377	ZNF213					0.19135	KLHL20	0.11353	ZDHHC13	0.84689	TBC1D22A	0.05317	LOC654841	0.03719	BCORL2
0.22503	ZFYVE1					0.19243	MKRN2	0.11418	NKRF	0.85167	TASP1	0.05347	BRCA1	0.03740	MMRN1
0.22630	PELP1					0.19351	JMY	0.11482	KIAA0368	0.85646	ZNF460	0.05377	PTCH1	0.03761	ANO7
0.22756	BRSK2					0.19459	FAF2	0.11546	DPM1	0.86124	UBE2G2	0.05407	UOX	0.03782	LOC100288233
0.22882	RAD23A					0.19568	ABL2	0.11610	SUPT16H	0.86603	U2AF1	0.05437	DDC	0.03803	FER1L5
0.23009	KNDC1					0.19676	CHIC2	0.11674	RPAP3	0.87081	PDE3B	0.05467	ZNF677	0.03824	C1orf167
0.23135	PACS1					0.19784	SDCBP	0.11738	SUCLA2	0.87560	ZNF17	0.05497	C10orf11	0.03845	C19orf45
0.23262	SAMD4B					0.19892	UAP1	0.11802	TAF2	0.88038	RRP8	0.05527	IGJ	0.03866	AIM1L
0.23388	ACO2					0.20000	SSR3	0.11867	PTPLAD1	0.88517	MSL3	0.05557	BCAS2	0.03887	C1orf68
0.23515	SIPA1L3					0.20108	HCG18	0.11931	TTPAL	0.88995	LCOR	0.05587	OR51B2	0.03908	SULT1C3
0.23641	ZNF574					0.20216	FBXO45	0.11995	ATXN10	0.89474	LOC644397	0.05617	NEDD4	0.03929	SLC26A9
0.23767	WBP2					0.20324	C1orf163	0.12059	CSRNP2	0.89952	SPATA7	0.05647	C1QTNF9B	0.03950	OXER1
0.23894	RANBP10					0.20432	ZNF639	0.12123	BTBD1	0.90431	ATRX	0.05677	ATF7IP	0.03971	FOXA2
0.24020	FURIN					0.20541	FEM1C	0.12187	MRPL34	0.90909	ALKBH3	0.05707	MS4A14	0.03992	AQP6
0.24147	PRAF2					0.20649	TMEM181	0.12251	POP4	0.91388	LOC283357	0.05737	ANKAR	0.04013	LCE5A
0.24273	CD276					0.20757	CCM2	0.12316	CKAP2	0.91866	MIPOL1	0.05767	USP44	0.04034	TPPP2
0.24399	USP20					0.20865	EIF2A	0.12380	KIAA1279	0.92344	ALDH1L2	0.05798	TAAR8	0.04055	LCTL
0.24526	AP3D1					0.20973	WBSCR22	0.12444	PPP2R4	0.92823	ZNF37A	0.05828	CAPN13	0.04076	LOC732275
0.24652	ATP5D					0.21081	C2orf74	0.12508	CINP	0.93301	ZNF793	0.05858	CASC5	0.04097	DEGS2
0.24779	LY6E					0.21189	RABL3	0.12572	PSMA1	0.93780	ZNF615	0.05888	THSD7B	0.04119	HMCN2
0.24905	UBL7					0.21297	PLEKHA2	0.12636	LOC100133959	0.94258	MAPKAPK5	0.05918	TFAMP1	0.04140	RASAL3
0.25032	CSNK1E					0.21405	MGAT4A	0.12700	SEH1L	0.94737	LOC642236	0.05948	LOC643401	0.04161	IGHA1
0.25158	FAM127B					0.21514	FAM69A	0.12765	EIF2S1	0.95215	SLC25A16	0.05978	C12orf48	0.04182	ENDOU
0.25284	C11orf2					0.21622	CRBN	0.12829	KCNQ3	0.95694	MTG1	0.06008	C10orf112	0.04203	PKHD1L1
0.25411	ILF3					0.21730	PPP1R7	0.12893	AP2B1	0.96172	ZBED5	0.06038	AKR1D1	0.04224	MPP4

0.25537	SNX32					0.21838	C2orf49	0.12957	PDK3	0.96651	CCDC144A	0.06068	VWA3B	0.04245	UMOD
0.25664	EZH1					0.21946	HMGN4	0.13021	ZDHHC17	0.97129	SAT1	0.06098	GRAP2	0.04266	TCP11
0.25790	PATL1					0.22054	ASH1L	0.13085	UBE2A	0.97608	METT5D1	0.06128	EOMES	0.04287	CHRNG
0.25917	ATXN2					0.22162	GEMIN5	0.13149	AFG3L2	0.98086	LOC100132077	0.06158	CASS4	0.04308	ARHGEF15
0.26043	TBC1D13					0.22270	KLHL2	0.13214	PSMA3	0.98565	NTRK2	0.06188	SRPX2	0.04329	CLEC4GP1
0.26169	BTBD2					0.22378	ORMDL1	0.13278	FBXO33	0.99043	TAS2R10	0.06218	IQCA1L	0.04350	TPTE
0.26296	SF1					0.22486	DOCK4	0.13342	DYNC1H1	0.99522	ERC1	0.06248	RAD51AP1	0.04371	MIXL1
0.26422	X3735847					0.22595	CAPZA2	0.13406	GPR180			0.06278	RPS20P27	0.04392	PUS1
0.26549	LRFN1					0.22703	KDM3A	0.13470	GNB5			0.06308	SPRR2F	0.04413	MSLNL
0.26675	ATP9A					0.22811	PPP2R2B	0.13534	FAR2			0.06338	SLC18A1	0.04434	GABRP
0.26802	CCNK					0.22919	TRIM27	0.13598	M6PR			0.06368	ERVFRDE1	0.04455	LOC389997
0.26928	CRY2					0.23027	MRFAP1	0.13663	ATP6V1H			0.06398	MAK	0.04476	SALL4
0.27054	FBXO31					0.23135	NSMCE2	0.13727	DGKE			0.06428	TBC1D8B	0.04497	SLC15A1
0.27181	NEURL					0.23243	ASNSD1	0.13791	ARPC5L			0.06458	FBXL21	0.04518	FLJ43860
0.27307	NCOA6					0.23351	MPP6	0.13855	BSCL2			0.06488	KRTAP7-1	0.04539	DGAT2L7
0.27434	C13orf23					0.23459	KIAA0947	0.13919	TMOD2			0.06518	ZNF99	0.04560	MYOZ3
0.27560	RARA					0.23568	ZNF512	0.13983	TBK1			0.06549	PRRG4	0.04581	NTRK1
0.27686	WDR37					0.23676	LSM5	0.14047	SH3GL2			0.06579	CRYGA	0.04602	AFAP1L1
0.27813	KLHL26					0.23784	EDEM1	0.14112	NIPA2			0.06609	SPAG17	0.04623	MYOG
0.27939	TXNDC11					0.23892	SLC30A5	0.14176	PWP1			0.06639	LOC100132798	0.04644	NEK2
0.28066	LINGO1					0.24000	CEP70	0.14240	C16orf52			0.06669	CNTLN	0.04665	CCDC36
0.28192	MESDC1					0.24108	YIPF4	0.14304	NDUFA12			0.06699	TCF19	0.04686	SLC9A10
0.28319	GTF3C1					0.24216	RIPK2	0.14368	LOC646482			0.06729	BEST3	0.04707	C7orf57
0.28445	SMARCA4					0.24324	LRPAP1	0.14432	SAMD12			0.06759	C1orf168	0.04728	SPINK5
0.28571	SGK223					0.24432	IPO9	0.14496	KPNA3			0.06789	LOC100129318	0.04749	MTMR9L
0.28698	STIM1					0.24541	C2orf69	0.14561	APLP2			0.06819	MAGEB5	0.04770	NR1I3
0.28824	CPSF1					0.24649	RABGGTB	0.14625	AMN1			0.06849	CENPE	0.04791	CD207
0.28951	BCL9L					0.24757	C1orf52	0.14689	CDC27			0.06879	GDF15	0.04812	ALDH3A1
0.29077	CABIN1					0.24865	HNRNPA3	0.14753	MTFMT			0.06909	PEX7	0.04833	LOC100129027
0.29204	KIAA1267					0.24973	MTIF2	0.14817	ACOT9			0.06939	GPC3	0.04854	TG
0.29330	MED12					0.25081	RNASEN	0.14881	MYH10			0.06969	LOC728218	0.04875	ZCWPW1
0.29456	ANKRD13D					0.25189	SKIL	0.14945	CCT2			0.06999	LOC650095	0.04896	PTCRA
0.29583	SPIRE1					0.25297	MRPL44	0.15010	CDC37L1			0.07029	PGBD2	0.04917	SUSD3
0.29709	IRF2BP1					0.25405	KLHL7	0.15074	LOC100129942			0.07059	UGT1A1	0.04938	KIAA1683
0.29836	SSRP1					0.25514	USP45	0.15138	CDC123			0.07089	FLJ37396	0.04959	SLC25A45
0.29962	VAC14					0.25622	THUMPD3	0.15202	PSMD5			0.07119	SAMD3	0.04980	ALOX15B
0.30088	SIRPA					0.25730	MFSD1	0.15266	KIAA2022			0.07149	LOC647107	0.05001	ANKRD20B
0.30215	DCAF7					0.25838	LPHN3	0.15330	DLG3			0.07179	AQPEP	0.05022	MAD1L1
0.30341	E2F4					0.25946	FAM162A	0.15394	MORF4L2			0.07209	IL15	0.05043	KIAA1875
0.30468	GPSM1					0.26054	TGOLN2	0.15459	OSBP			0.07239	RPSA	0.05064	PRIC285
0.30594	ZNF335					0.26162	TWISTNB	0.15523	ACADSB			0.07269	CEP135	0.05085	SUCLG2
0.30721	DIDO1					0.26270	SUPT3H	0.15587	PHLPP2			0.07299	ASGR2	0.05106	OR11H4

0.30847	TBC1D25	0.26378	DCP2	0.15651	CCDC109A	0.07330	ALB	0.05127	SLC13A1
0.30973	CKB	0.26486	MRFAP1L1	0.15715	CSTF3	0.07360	C12orf50	0.05148	ANKRD33
0.31100	ARRB2	0.26595	MRPL53	0.15779	MAPK8	0.07390	LOC646214	0.05169	FZD10
0.31226	ZMYM3	0.26703	SNX4	0.15843	DYNLT3	0.07420	CCL20	0.05190	LTB4R
0.31353	RIC8A	0.26811	MIER1	0.15908	RBM11	0.07450	PCA3	0.05211	COL15A1
0.31479	TTLL12	0.26919	MCM6	0.15972	MID2	0.07480	SPATA5L1	0.05232	LOC100289673
0.31606	WIPF2	0.27027	FAM119A	0.16036	NBEA	0.07510	C14orf38	0.05253	HOXB13
0.31732	CRAT	0.27135	MXD1	0.16100	C20orf7	0.07540	CCDC11	0.05274	BLK
0.31858	DCP1B	0.27243	LOC653160	0.16164	PGAM5	0.07570	VEPH1	0.05295	WDR64
0.31985	TSHZ1	0.27351	CYB5R1	0.16228	NPTN	0.07600	CENPH	0.05316	SLC23A3
0.32111	MED25	0.27459	RPAP2	0.16292	NIPAL2	0.07630	WHAMML1	0.05337	FANCD2
0.32238	RNF214	0.27568	C7orf36	0.16357	RBM18	0.07660	MAP1D	0.05358	PLA1A
0.32364	NRXN2	0.27676	SLC30A7	0.16421	MAP3K7IP3	0.07690	HNF4G	0.05379	IGSF9
0.32491	SAMD1	0.27784	RPF1	0.16485	PARP2	0.07720	DTL	0.05400	PPP1R13L
0.32617	BRSK1	0.27892	DCTN6	0.16549	ASAH2B	0.07750	C13orf33	0.05421	LOC283485
0.32743	CHAD	0.28000	C2orf44	0.16613	OSBPL8	0.07780	COX17	0.05442	BASE
0.32870	CDH22	0.28108	IARS2	0.16677	SLC12A6	0.07810	LOC728675	0.05463	SPATA3
0.32996	C16orf58	0.28216	CREB1	0.16742	LIN7A	0.07840	TNIP3	0.05484	ZNF385C
0.33123	SLC7A5	0.28324	CWC22	0.16806	POMP	0.07870	LOC642357	0.05505	CCDC157
0.33249	PRR24	0.28432	BCS1L	0.16870	NETO2	0.07900	GIPC2	0.05526	FES
0.33375	CAPN5	0.28541	YWHAQ	0.16934	BLOC1S2	0.07930	LPA	0.05547	NEURL3
0.33502	TESK1	0.28649	ETF1	0.16998	AKAP11	0.07960	LOC100128908	0.05568	BANF2
0.33628	SALL2	0.28757	NEK4	0.17062	NT5DC3	0.07990	LOC283314	0.05589	C21orf129
0.33755	C19orf6	0.28865	GPN1	0.17126	PAPD5	0.08020	TMEM26	0.05610	NXF4
0.33881	NFIC	0.28973	POLB	0.17191	IFNAR1	0.08050	ZSCAN16	0.05631	C9orf100
0.34008	INPP5K	0.29081	NRAS	0.17255	TMEM62	0.08081	LOC100294489	0.05652	GCM2
0.34134	ATMIN	0.29189	OXNAD1	0.17319	SCFD1	0.08111	ZNF726	0.05673	CD33
0.34260	C17orf39	0.29297	ZNF398	0.17383	KRR1	0.08141	LOC283867	0.05694	APOC1
0.34387	GRN	0.29405	DRAM2	0.17447	ACACA	0.08171	LOC151234	0.05715	LOC646960
0.34513	G6PD	0.29514	NUB1	0.17511	TCEB1	0.08201	CRTAM	0.05736	ANTXRL
0.34640	ZC3H18	0.29622	C6orf62	0.17575	ADRBK2	0.08231	MMEL1	0.05758	KRTAP6-1
0.34766	NDST2	0.29730	POLR1B	0.17640	VCPIP1	0.08261	DKFZp686F0839	0.05779	ABCA13
0.34893	FADS3	0.29838	GTDC1	0.17704	ARMCX5	0.08291	FAM71D	0.05800	CDC42BPG
0.35019	X3687752	0.29946	GET4	0.17768	PRKAG1	0.08321	RBL1	0.05821	HTRA4
0.35145	SLC39A3	0.30054	C5orf44	0.17832	UBE3A	0.08351	ZNF599	0.05842	XCR1
0.35272	SBK1	0.30162	WDR3	0.17896	HERC1	0.08381	ACSBG2	0.05863	MYL7
0.35398	UBE3B	0.30270	RIOK2	0.17960	USP8	0.08411	LOC100132686	0.05884	LOC642357
0.35525	MKNK2	0.30378	SEC22A	0.18024	SH3KBP1	0.08441	HPGD	0.05905	FCER1A
0.35651	MSMP	0.30486	HCG18	0.18089	NF2	0.08471	CCT6P4	0.05926	SIGLEC1
0.35777	NPDC1	0.30595	ERCC8	0.18153	FBXO21	0.08501	NPVF	0.05947	HEATR7B2
0.35904	MUS81	0.30703	PKIA	0.18217	POLR3B	0.08531	LOC728597	0.05968	PLA2G2A
0.36030	ELFN2	0.30811	TXNDC15	0.18281	RAB39B	0.08561	KIF15	0.05989	MYO3B

0.36157	MMS19	0.30919	PTPRK	0.18345	BCAS3	0.08591	TARP	0.06010	TEKT2
0.36283	FUT1	0.31027	PDE6D	0.18409	XK	0.08621	ASB8	0.06031	C17orf90
0.36410	PQLC1	0.31135	RALGPS2	0.18473	RAB11FIP2	0.08651	REEP3	0.06052	UGT3A1
0.36536	FAM100A	0.31243	SLC4A7	0.18538	ATAD1	0.08681	ESR1	0.06073	FCRL6
0.36662	FAM19A5	0.31351	SIKE1	0.18602	RAB12	0.08711	LOC100292664	0.06094	TMC5
0.36789	PGAP3	0.31459	PCCB	0.18666	TSG101	0.08741	C15orf33	0.06115	WISP2
0.36915	SRRM2	0.31568	APC	0.18730	PEG3	0.08771	ZNF66	0.06136	CCDC19
0.37042	DPP9	0.31676	WDR1	0.18794	TMEM85	0.08801	ZNF321	0.06157	SLC2A7
0.37168	PCDH19	0.31784	MOBKL1B	0.18858	MGEA5	0.08831	C2orf58	0.06178	CACNA2D4
0.37295	C12orf44	0.31892	COX18	0.18922	TOR1A	0.08862	TGDS	0.06199	TNFRSF9
0.37421	PES1	0.32000	RAB4A	0.18987	VCP	0.08892	ZIC3	0.06220	FAM197Y1
0.37547	PRRT1	0.32108	SEC62	0.19051	PRMT5	0.08922	RWDD3	0.06241	LOC221946
0.37674	POLDIP3	0.32216	VPS13D	0.19115	TAF4B	0.08952	LOC100131781	0.06262	MFSD7
0.37800	PCBP3	0.32324	IMPDH2	0.19179	PPME1	0.08982	SCML2	0.06283	DCST1
0.37927	FOXP4	0.32432	C3orf37	0.19243	THYN1	0.09012	OR11A1	0.06304	IGFN1
0.38053	COASY	0.32541	PFDN2	0.19307	TEX10	0.09042	GABRR1	0.06325	C1orf54
0.38180	GNAO1	0.32649	TFB2M	0.19371	GRIA3	0.09072	TEPP	0.06346	PKD1L1
0.38306	RNF44	0.32757	POLR3D	0.19436	MBD2	0.09102	LOC729032	0.06367	SFTPC
0.38432	CHID1	0.32865	BZW1	0.19500	PGK1	0.09132	LRRC66	0.06388	RDH16
0.38559	CLCN7	0.32973	MINA	0.19564	CCDC113	0.09162	UBE2DNL	0.06409	LOC253724
0.38685	HUNK	0.33081	NR3C1	0.19628	DYM	0.09192	CXorf56	0.06430	MARVELD3
0.38812	ADCK1	0.33189	VPRBP	0.19692	SLC16A7	0.09222	OR5B21	0.06451	TCF7
0.38938	BAIAP2	0.33297	KIAA1109	0.19756	CAPRIN1	0.09252	KIF20B	0.06472	LOC100287877
0.39064	FAM89B	0.33405	SFRS12IP1	0.19820	LRRC49	0.09282	ZNF501	0.06493	PKHD1
0.39191	NCS1	0.33514	DNAJC21	0.19885	COPS2	0.09312	CASR	0.06514	C20orf203
0.39317	SH3BGRL3	0.33622	WRNIP1	0.19949	MLLT3	0.09342	BHMT2	0.06535	MYH15
0.39444	SLITRK2	0.33730	PTP4A1	0.20013	DNAJA1	0.09372	C9orf153	0.06556	LOC145845
0.39570	PEX26	0.33838	RLF	0.20077	MTPAP	0.09402	DNAH12	0.06577	ACE2
0.39697	KDM2A	0.33946	RNF146	0.20141	ATP2B1	0.09432	SLFN12	0.06598	FRMD1
0.39823	ZBTB4	0.34054	FUCA1	0.20205	ATXN7L3B	0.09462	IL7R	0.06619	TMEM72
0.39949	SP2	0.34162	ZNHIT1	0.20269	DRG1	0.09492	EBF2	0.06640	AFM
0.40076	DRAP1	0.34270	CDC23	0.20334	PAK3	0.09522	NCRNA00236	0.06661	XIRP1
0.40202	ZNF219	0.34378	JAKMIP1	0.20398	FAM199X	0.09552	NAALADL2	0.06682	IL11
0.40329	LMF2	0.34486	GTF2H5	0.20462	KIAA1797	0.09582	MYO1H	0.06703	FAM178B
0.40455	SFRS2B	0.34595	PACRGL	0.20526	MYO5A	0.09612	PPIC	0.06724	GTSE1
0.40582	SPNS1	0.34703	PMM1	0.20590	C16orf45	0.09643	FLJ43879	0.06745	LOC286135
0.40708	SYVN1	0.34811	SEC62	0.20654	GABRB3	0.09673	AVPR1A	0.06766	FLJ16124
0.40834	CYTSA	0.34919	RFC2	0.20718	C9orf4	0.09703	GYPE	0.06787	TSGA10IP
0.40961	RAB1B	0.35027	SLBP	0.20783	SUPV3L1	0.09733	MCM9	0.06808	ANKRD31
0.41087	GRAMD1A	0.35135	EIF2AK3	0.20847	PNMAL1	0.09763	CD44	0.06829	MYBPC3
0.41214	IRS2	0.35243	IPP	0.20911	RNF128	0.09793	C6	0.06850	OFCC1
0.41340	CDC34	0.35351	TADA2B	0.20975	DCTN2	0.09823	HMMR	0.06871	SAGE1

0.41466	SUPT5H	0.35459	ALS2CR4	0.21039	NAPB	0.09853	LOC441453	0.06892	IL31RA
0.41593	OBFC1	0.35568	PEX13	0.21103	GTF2A1	0.09883	RAET1L	0.06913	WFDC10B
0.41719	SMPD1	0.35676	RABGEF1	0.21167	KLHL11	0.09913	ARSH	0.06934	SYT8
0.41846	SOCS7	0.35784	FASTKD1	0.21232	TIMM22	0.09943	LOC100288366	0.06955	HOXD4
0.41972	LZTS1	0.35892	S100A13	0.21296	SCG5	0.09973	BEND3	0.06976	APOC4
0.42099	STAT6	0.36000	RPE	0.21360	NRIP3	0.10003	RG9MTD2	0.06997	LCA10
0.42225	PRSS3	0.36108	KCTD20	0.21424	PITPNB	0.10033	PLSCR2	0.07018	XAGE-4
0.42351	ZNF385A	0.36216	RAB33B	0.21488	SPIN1	0.10063	FKBP7	0.07039	LOC100101266
0.42478	SIAH3	0.36324	PRKAR2A	0.21552	KCNMA1	0.10093	RBPMS	0.07060	NPC1L1
0.42604	DBP	0.36432	RFWD2	0.21616	CNTN1	0.10123	GPR128	0.07081	MUC12
0.42731	SPRN	0.36541	NMD3	0.21681	NARS	0.10153	C1orf61	0.07102	OR2Y1
0.42857	EGR3	0.36649	FEZ2	0.21745	C10orf118	0.10183	LDHAL6A	0.07123	SLC22A2
0.42984	C17orf68	0.36757	SNCA	0.21809	MRPL45	0.10213	WEE1	0.07144	KRT19P2
0.43110	FAM195B	0.36865	C5orf43	0.21873	COG6	0.10243	ATAD5	0.07165	GMIP
0.43236	LOC728743	0.36973	SEC62	0.21937	GLYR1	0.10273	TFAP2D	0.07186	GAB4
0.43363	JAK3	0.37081	DDX20	0.22001	KIAA0564	0.10303	ATP8B5P	0.07207	F11
0.43489	SYMPK	0.37189	USP46	0.22065	PRPS2	0.10333	LOC286467	0.07228	LOC345051
0.43616	RBM23	0.37297	CCDC149	0.22130	GPRASP2	0.10363	FAM176A	0.07249	CABP4
0.43742	LSM14B	0.37405	ZNF717	0.22194	RB1	0.10394	TRIP6	0.07270	FAM86D
0.43869	KIAA1659	0.37514	C6orf108	0.22258	KIAA1012	0.10424	ASPN	0.07291	CEACAM1
0.43995	AGPAT3	0.37622	AMD1	0.22322	MAP2K4	0.10454	LOC285045	0.07312	AIM1
0.44121	JMJD8	0.37730	C2orf64	0.22386	SLC8A3	0.10484	TMPRSS11BNL	0.07333	TGM4
0.44248	TSPAN14	0.37838	TADA1	0.22450	B4GALT5	0.10514	C4orf49	0.07354	LOC100130857
0.44374	TSC1	0.37946	PLD5	0.22514	RC3H2	0.10544	C1orf135	0.07375	C5orf20
0.44501	LDB1	0.38054	VPS37A	0.22579	LCMT1	0.10574	C8orf45	0.07397	DHODH
0.44627	RANBP3	0.38162	LYRM2	0.22643	GABRA3	0.10604	FAM26E	0.07418	DSG1
0.44753	EXOC7	0.38270	SNAPIN	0.22707	ZDHHC5	0.10634	TSPAN1	0.07439	MPL
0.44880	LPHN1	0.38378	SPCS3	0.22771	GGNBP2	0.10664	CLEC4A	0.07460	LOC100287712
0.45006	SETD1B	0.38486	STARD3NL	0.22835	DCAF10	0.10694	C17orf87	0.07481	C15orf2
0.45133	ERGIC3	0.38595	ZNF7	0.22899	PGM2L1	0.10724	BCL2L15	0.07502	LBP
0.45259	SUPT6H	0.38703	SKAP2	0.22963	NOVA1	0.10754	C2orf84	0.07523	MAS1L
0.45386	CCDC97	0.38811	SLC33A1	0.23028	TMEM55A	0.10784	RARG	0.07544	KCP
0.45512	TOM1L2	0.38919	NLGN1	0.23092	RNF6	0.10814	LOC284798	0.07565	LOC100133746
0.45638	UBE2L6	0.39027	MRPS9	0.23156	HMG20A	0.10844	LOC644554	0.07586	GAS2L2
0.45765	TERF2	0.39135	CCBL2	0.23220	SNRPN	0.10874	MTL5	0.07607	PRDM6
0.45891	DGCR11	0.39243	SP4	0.23284	PDK3	0.10904	ACER2	0.07628	KRT9
0.46018	FAM57A	0.39351	CRBN	0.23348	SLC2A13	0.10934	CLEC7A	0.07649	C9
0.46144	MED15	0.39459	TMEM167A	0.23412	GDPD1	0.10964	PCOLCE2	0.07670	KLRG2
0.46271	KIAA0284	0.39568	SF3B14	0.23477	PPM1A	0.10994	PLN	0.07691	CSNK1A1P
0.46397	PRDM10	0.39676	EIF2B3	0.23541	MTCH2	0.11024	LETM2	0.07712	TNFRSF10A
0.46523	HRH3	0.39784	SETD7	0.23605	EAPP	0.11054	LYAR	0.07733	WDR90
0.46650	POLR1D	0.39892	LTV1	0.23669	ARIH1	0.11084	ARMC3	0.07754	MFAP2

0.46776	TCF25	0.40000	PPP1R15B	0.23733	ARHGAP20	0.11114	CD180	0.07775	LOC100132529
0.46903	DACT3	0.40108	TMEM192	0.23797	GPR158	0.11144	SAMD13	0.07796	CCL11
0.47029	PPM1G	0.40216	ZFYVE20	0.23861	TTC39C	0.11175	SFRP4	0.07817	TMEM37
0.47155	MAP1S	0.40324	DIMT1L	0.23926	GOT2	0.11205	OR52J3	0.07838	CNGB3
0.47282	SNRPA	0.40432	GCLM	0.23990	RAB2B	0.11235	SLC24A1	0.07859	FCHSD1
0.47408	TOM1L2	0.40541	ESYT2	0.24054	GSPT1	0.11265	RPS26P11	0.07880	CCDC150
0.47535	TMEM8A	0.40649	OTUD6B	0.24118	ZFAND1	0.11295	C6orf150	0.07901	KRT71
0.47661	MAPK1IP1L	0.40757	ZNF12	0.24182	LYSMD4	0.11325	CYP2C18	0.07922	COL2A1
0.47788	SLC19A1	0.40865	MOBKL1A	0.24246	HSPH1	0.11355	FBXW12	0.07943	PADI4
0.47914	MED24	0.40973	ZNF281	0.24310	TMED8	0.11385	C1orf185	0.07964	PTGIR
0.48040	INPP5E	0.41081	KLHL8	0.24375	KCNJ6	0.11415	HOXA4	0.07985	BPIL2
0.48167	DPH1	0.41189	CXXC5	0.24439	ATXN3	0.11445	FMO1	0.08006	CORL2
0.48293	PRR12	0.41297	TOR1AIP2	0.24503	C20orf24	0.11475	ST7	0.08027	GPR171
0.48420	GAL3ST3	0.41405	GNL2	0.24567	TM2D2	0.11505	LOC645752	0.08048	DSPP
0.48546	LOC100126784	0.41514	EIF4E	0.24631	NBN	0.11535	WHAMML2	0.08069	CATSPERB
0.48673	TM7SF2	0.41622	DNAJC2	0.24695	IPO7	0.11565	RPL13AP17	0.08090	TNFSF14
0.48799	NOC2L	0.41730	HGSNAT	0.24759	PDK3	0.11595	POPDC2	0.08111	C20orf173
0.48925	AGRN	0.41838	RER1	0.24824	PPP1R12A	0.11625	IFLTD1	0.08132	CYP3A5
0.49052	PTGES2	0.41946	LEPROTL1	0.24888	C14orf2	0.11655	LRGUK	0.08153	WAS
0.49178	SREBF2	0.42054	CEP170	0.24952	APP	0.11685	ZNF30	0.08174	PRSS45
0.49305	C11orf30	0.42162	SAP30L	0.25016	LGI1	0.11715	PDCL2	0.08195	ECSCR
0.49431	ARL3	0.42270	WDR35	0.25080	MLF2	0.11745	LOC100287822	0.08216	ADAM33
0.49558	TTC28	0.42378	YEATS2	0.25144	CUL5	0.11775	CRYGC	0.08237	GPR55
0.49684	CYB5R3	0.42486	ABI2	0.25208	KBTBD3	0.11805	RRN3P1	0.08258	CYP11B1
0.49810	H1F0	0.42595	FAM120B	0.25273	ZNF675	0.11835	EEF1B2	0.08279	BCL3
0.49937	SNAP29	0.42703	TOR1AIP2	0.25337	ELAVL2	0.11865	PRSS55	0.08300	OVOL1
0.50063	FLII	0.42811	LOC100216479	0.25401	AQR	0.11895	COL28A1	0.08321	PIF1
0.50190	CRKL	0.42919	PDE4DIP	0.25465	ERGIC2	0.11926	C2orf7	0.08342	LOC100132015
0.50316	UNKL	0.43027	PFDN6	0.25529	NDUFAB1	0.11956	N4BP2	0.08363	A1BG
0.50442	SEZ6	0.43135	NAPEPLD	0.25593	CCDC6	0.11986	LBR	0.08384	COL20A1
0.50569	TUFM	0.43243	EBAG9	0.25657	TTC39B	0.12016	ACRC	0.08405	TPO
0.50695	SENP3	0.43351	NUCKS1	0.25722	SRP54	0.12046	CDK15	0.08426	SLC22A8
0.50822	TECR	0.43459	TFDP2	0.25786	RAB14	0.12076	LOC100128507	0.08447	PROM2
0.50948	PPP1CA	0.43568	RSPH3	0.25850	USP32	0.12106	CXCL13	0.08468	KLHDC7A
0.51075	RPAP1	0.43676	SMARCAL1	0.25914	ZNF510	0.12136	AFP	0.08489	TMPRSS13
0.51201	CHD8	0.43784	POMGNT1	0.25978	ABCD2	0.12166	CARD8	0.08510	MCM2
0.51327	NFKBIB	0.43892	POT1	0.26042	NCBP1	0.12196	LOC100130065	0.08531	TIMELESS
0.51454	PRKCSH	0.44000	MRPL3	0.26106	C12orf24	0.12226	OR13C3	0.08552	PATL2
0.51580	CLPP	0.44108	ZNF643	0.26171	RRM2B	0.12256	C8orf73	0.08573	SMEK3P
0.51707	FHL3	0.44216	CSNK1A1	0.26235	ZBTB33	0.12286	ADAM18	0.08594	CLCNKB
0.51833	SLC25A39	0.44324	MTA3	0.26299	C13orf34	0.12316	TSPAN19	0.08615	KCNH6
0.51960	CCDC106	0.44432	GNL1	0.26363	LOC100128608	0.12346	FREM1	0.08636	ABHD1

0.52086	SLC9A8					0.44541	GBAS	0.26427	NFS1			0.12376	C4orf21	0.08657	VILL
0.52212	KIAA0182					0.44649	UBE2B	0.26491	ZADH2			0.12406	CCDC9	0.08678	PRSS38
0.52339	TRIM47					0.44757	TMEM56	0.26555	LOC100129775			0.12436	LILRB1	0.08699	CYP4B1
0.52465	C18orf49					0.44865	PPCS	0.26620	DNAJC7			0.12466	CCT8P1	0.08720	FCRL2
0.52592	RAB36					0.44973	CBR4	0.26684	ADK			0.12496	FBXO15	0.08741	ANPEP
0.52718	RBM14					0.45081	HS2ST1	0.26748	GPRASP1			0.12526	IL1F6	0.08762	KRTAP5-6
0.52845	MRPS16					0.45189	ZMAT2	0.26812	ZNF597			0.12556	PIWIL4	0.08783	FOXN4
0.52971	RAI2					0.45297	TADA3	0.26876	KLHL9			0.12586	OLAH	0.08804	LOC644992
0.53097	NYNRIN					0.45405	C5orf24	0.26940	SLC9A7			0.12616	FLJ39095	0.08825	MMP13
0.53224	PVRL1					0.45514	DUSP19	0.27004	LOC100131208			0.12646	OR2W1	0.08846	KRT1
0.53350	SGSH					0.45622	ROBO1	0.27069	HMOX2			0.12676	ZNF546	0.08867	CLDN19
0.53477	LOC100128332					0.45730	MLF1	0.27133	CDKL5			0.12707	CDH26	0.08888	WNT10A
0.53603	EHD2					0.45838	ATIC	0.27197	KLHL15			0.12737	AKAP14	0.08909	AKNAD1
0.53729	LOC92973					0.45946	TSEN15	0.27261	CDH8			0.12767	FAM133B	0.08930	C2orf54
0.53856	SEC16A					0.46054	MARS2	0.27325	LRRTM3			0.12797	CEP110	0.08951	RHAG
0.53982	DTX3					0.46162	STAT1	0.27389	C13orf37			0.12827	TGIF1	0.08972	LAMA3
0.54109	XPO6					0.46270	SLC7A14	0.27453	HCCS			0.12857	NBLA00301	0.08993	C16orf11
0.54235	PTPRJ					0.46378	KIAA1109	0.27518	SVIP			0.12887	CPS1	0.09014	UPK2
0.54362	C16orf46					0.46486	TCEB3	0.27582	NDEL1			0.12917	CYP3A43	0.09036	SLC28A2
0.54488	ZNF512B					0.46595	CDYL	0.27646	STK38L			0.12947	SLC47A2	0.09057	NUDT16P1
0.54614	PRR4					0.46703	AKIRIN2	0.27710	ERO1L			0.12977	TCEB1P20	0.09078	AMBP
0.54741	AKAP1					0.46811	EXOSC9	0.27774	DOPEY2			0.13007	RPLP0	0.09099	COL10A1
0.54867	CTNND1					0.46919	OSTM1	0.27838	XPOT			0.13037	PM20D1	0.09120	RORC
0.54994	MED16					0.47027	C1orf123	0.27903	ARNTL			0.13067	CUBN	0.09141	LOC100129999
0.55120	E2F1					0.47135	GPM6A	0.27967	TBRG1			0.13097	LOC100287731	0.09162	DNMT3B
0.55247	FLRT1					0.47243	ZNF518B	0.28031	GRIN2A			0.13127	PHEX	0.09183	COL1A1
0.55373	PHB2					0.47351	FAM200B	0.28095	SEPHS2			0.13157	OR8J3	0.09204	ZNF541
0.55499	DIDO1					0.47459	PIK3R1	0.28159	FXR2			0.13187	BAAT	0.09225	MYL1
0.55626	LIN37					0.47568	LOC100129518	0.28223	PNMA2			0.13217	PYCARD	0.09246	SLC16A5
0.55752	EDEM2					0.47676	MRPL47	0.28287	AHNAK2			0.13247	KRT78	0.09267	LOC650621
0.55879	TEF					0.47784	ENAH	0.28352	LIG4			0.13277	ECM2	0.09288	LOC100129060
0.56005	KAT5					0.47892	SEC24A	0.28416	PCNX			0.13307	CXorf24	0.09309	LCA5L
0.56131	MPP2					0.48000	DCAF16	0.28480	PHKB			0.13337	C6orf167	0.09330	KLRB1
0.56258	DMWD					0.48108	PDCD10	0.28544	HLCS			0.13367	C6orf186	0.09351	CDHR2
0.56384	C11orf24					0.48216	GUCY1A3	0.28608	USPL1			0.13397	C10orf4	0.09372	LOC646936
0.56511	PAFAH1B3					0.48324	HINT3	0.28672	NAE1			0.13427	LYPLA2	0.09393	PCDH12
0.56637	CDC6					0.48432	CTSB	0.28736	LOC728477			0.13457	LOC100129697	0.09414	CACNA1F
0.56764	LRSAM1					0.48541	RQCD1	0.28801	RPA1			0.13488	CXorf22	0.09435	LOC646179
0.56890	ELAVL3					0.48649	HIST2H2BF	0.28865	SYT4			0.13518	EFHB	0.09456	PCK1
0.57016	TPST2					0.48757	DNAJB11	0.28929	GOLT1B			0.13548	DNAH5	0.09477	KCNJ5
0.57143	EVI5L					0.48865	BOD1L	0.28993	HN1			0.13578	LOC100129935	0.09498	LOC643962
0.57269	HPS1					0.48973	METTL13	0.29057	NPAT			0.13608	CLRN1	0.09519	FCRLA

0.57396	HINFP	0.49081	MRPS22	0.29121	MMGT1	0.13638	PTX3	0.09540	MCM10
0.57522	NCLN	0.49189	ZCCHC17	0.29185	CERK	0.13668	CCDC38	0.09561	TMPRSS15
0.57649	SF3B3	0.49297	BTD	0.29250	SLC1A1	0.13698	RPL35A	0.09582	LOC286094
0.57775	EI24	0.49405	TANK	0.29314	BEX1	0.13728	CDC25C	0.09603	KRTCAP3
0.57901	TNRC6B	0.49514	ANKRD50	0.29378	PTEN	0.13758	ZNF665	0.09624	APOL5
0.58028	C14orf4	0.49622	ZNF391	0.29442	BCAT1	0.13788	TMEM218	0.09645	NUDC
0.58154	KCNQ4	0.49730	POR	0.29506	RAB9B	0.13818	SLC26A3	0.09666	SLC22A11
0.58281	OPA3	0.49838	ODC1	0.29570	RAN	0.13848	LOC644303	0.09687	LOC100286928
0.58407	MLEC	0.49946	PRDX1	0.29634	POLE3	0.13878	KIF11	0.09708	SLC5A12
0.58534	UBE2I	0.50054	CGGBP1	0.29699	LARP4	0.13908	C4orf43	0.09729	LOC646085
0.58660	PPIL2	0.50162	DIRC2	0.29763	MRPL39	0.13938	CAPSL	0.09750	OR8A1
0.58786	MAFG	0.50270	MBLAC2	0.29827	FAM155A	0.13968	KIAA1524	0.09771	UNC13D
0.58913	NCAPH2	0.50378	KDELR2	0.29891	C12orf51	0.13998	DEFB112	0.09792	MYH16
0.59039	ABAT	0.50486	DCP1A	0.29955	GRIA4	0.14028	ZNF67P	0.09813	LOC284933
0.59166	USP2	0.50595	COPS7B	0.30019	COX16	0.14058	ATRIP	0.09834	CDKN2A
0.59292	MCRS1	0.50703	HCG18	0.30083	C16orf72	0.14088	TLR5	0.09855	SH2D4A
0.59418	PNPLA2	0.50811	TMEM48	0.30148	ETNK1	0.14118	C1orf69	0.09876	USP6
0.59545	DCAKD	0.50919	COMMD10	0.30212	ITSN1	0.14148	OR5L1	0.09897	ACCS
0.59671	AXIN2	0.51027	CCDC75	0.30276	SPA17	0.14178	HLA-DOA	0.09918	NCRNA00207
0.59798	ACCN2	0.51135	TRAF3IP1	0.30340	C13orf1	0.14208	LOH3CR2A	0.09939	SYNPO2L
0.59924	ABI1	0.51243	POLR1C	0.30404	RSL1D1	0.14239	TNAP	0.09960	IYD
0.60051	GNPTG	0.51351	RNF216	0.30468	BICD1	0.14269	TFAP4	0.09981	MYCL1
0.60177	PCSK1N	0.51459	HIST1H4E	0.30532	FAM81A	0.14299	RNF32	0.10002	AMDHD1
0.60303	AK1	0.51568	QRSL1	0.30597	BEX4	0.14329	LOC100289061	0.10023	LCORL
0.60430	TMEM109	0.51676	SMURF1	0.30661	P2RX5	0.14359	MSTN	0.10044	LY6D
0.60556	AKT1S1	0.51784	RPN1	0.30725	FBXW2	0.14389	PAXIP1	0.10065	LGALS14
0.60683	PTOV1	0.51892	KIAA0240	0.30789	BTBD10	0.14419	MGST2	0.10086	DHRS2
0.60809	TOM1L2	0.52000	FAM200A	0.30853	KCNMA1	0.14449	UFM1	0.10107	C16orf91
0.60936	CACNA1G	0.52108	CREG1	0.30917	SEL1L	0.14479	SI	0.10128	HHLA1
0.61062	SSH2	0.52216	ZNF238	0.30981	SMCR7L	0.14509	ZNF829	0.10149	SLC10A3
0.61188	KCNN1	0.52324	ATG10	0.31046	LOC283270	0.14539	OR1N2	0.10170	LOC157860
0.61315	ISG20L2	0.52432	MTO1	0.31110	AP1M1	0.14569	BRCA2	0.10191	WDR90
0.61441	C14orf118	0.52541	HSPA9	0.31174	GNAQ	0.14599	LMOD1	0.10212	SERINC2
0.61568	DIDO1	0.52649	NAMPT	0.31238	SV2B	0.14629	LOC100130178	0.10233	NLRP13
0.61694	NKIRAS2	0.52757	IGF2R	0.31302	FAM199X	0.14659	DRAM1	0.10254	GPATCH3
0.61820	NDUFA3	0.52865	RYBP	0.31366	AKAP2	0.14689	LOC344887	0.10275	KIAA1656
0.61947	ARHGEF18	0.52973	BAI3	0.31430	PDHA1	0.14719	CCDC108	0.10296	RNASE12
0.62073	BCAT2	0.53081	C6orf89	0.31495	EEA1	0.14749	CENPM	0.10317	MYLK
0.62200	MIDN	0.53189	PRICKLE4	0.31559	PDXDC1	0.14779	C2orf63	0.10338	KLK8
0.62326	C21orf56	0.53297	LRP1B	0.31623	C10orf88	0.14809	CEBPZ	0.10359	LOC254028
0.62453	VPS53	0.53405	C8orf46	0.31687	MBTPS2	0.14839	USP49	0.10380	EXOC3L2
0.62579	FAM127C	0.53514	KIAA1614	0.31751	GSS	0.14869	HLA-L	0.10401	ALDOB

0.62705	SHROOM2	0.53622	SPEF2	0.31815	CDK8	0.14899	ALG6	0.10422	LOC729686		
0.62832	TAF3	0.53730	KLHL23	0.31879	DERL1	0.14929	LOC120824	0.10443	NLRP3		
0.62958	RILPL1	0.53838	PIGM	0.31944	TXNRD1	0.14959	ZNF561	0.10464	DNAH8		
0.63085	HIST1H2BM	0.53946	FAM174A	0.32008	NELL2	0.14989	TMEM51	0.10485	IFITM4P		
0.63211	VPS28	0.54054	DPYSL2	0.32072	NARS2	0.15020	MAP3K8	0.10506	SCN11A		
0.63338	C12orf73	0.54162	PURA	0.32136	KCNH5	0.15050	ZNF596	0.10527	KRT84		
0.63464	EMID2	0.54270	NSUN3	0.32200	C14orf129	0.15080	BLM	0.10548	C16orf58		
0.63590	FBXW5	0.54378	AKD1	0.32264	DSN1	0.15110	GYPA	0.10569	DUOX1		
0.63717	DNMT1	0.54486	HECW2	0.32328	PHF20	0.15140	LOC100289181	0.10590	C10orf111		
0.63843	ELL	0.54595	JMJD4	0.32393	CDC14B	0.15170	COPZ2	0.10611	SLAMF1		
0.63970	GRIN2D	0.54703	BBS12	0.32457	TRHDE	0.15200	IL18R1	0.10632	PLA2G2E		
0.64096	TLE3	0.54811	LOC285484	0.32521	TCTN3	0.15230	SGMS2	0.10653	LOC338579		
0.64223	LOC399904	0.54919	ZNF654	0.32585	PPM1H	0.15260	STATH	0.10675	LOC285692		
0.64349	LRRC27	0.55027	CDKN2AIP	0.32649	NSDHL	0.15290	SERPINB13	0.10696	SPATA19		
0.64475	MLL	0.55135	PHF7	0.32713	GNS	0.15320	ITIH2	0.10717	P2RX2		
0.64602	DIDO1	0.55243	ING3	0.32777	TM7SF3	0.15350	MOBKL2C	0.10738	DUSP13		
0.64728	TSPYL3	0.55351	C5orf30	0.32842	TUBGCP5	0.15380	C1orf112	0.10759	DECR2		
0.64855	SGPP1	0.55459	TMEM232	0.32906	PDK3	0.15410	LOC646048	0.10780	C14orf178		
0.64981	CSRP2BP	0.55568	LPCAT1	0.32970	PPM1E	0.15440	FBXO31	0.10801	RASSF7		
0.65107	SEC16A	0.55676	DPY30	0.33034	RAE1	0.15470	MYB	0.10822	MATN4		
0.65234	SLC37A1	0.55784	RAD17	0.33098	CHGB	0.15500	TTC18	0.10843	TYMP		
0.65360	MLXIP	0.55892	DDI2	0.33162	ASAH1	0.15530	C12orf28	0.10864	IL8		
0.65487	MCF2L	0.56000	UNC80	0.33226	ZFP1	0.15560	SULT1B1	0.10885	FCGR2B		
0.65613	COL6A1	0.56108	NCK1	0.33291	PSMB7	0.15590	GLYATL2	0.10906	KCNK4		
0.65740	USP22	0.56216	PIGF	0.33355	GAPVD1	0.15620	CASP8	0.10927	RFX6		
0.65866	OSBP2	0.56324	SEC62	0.33419	CSTF1	0.15650	IFIH1	0.10948	LOC644277		
0.65992	MIER2	0.56432	DAP3	0.33483	GRIN2A	0.15680	LOC644456	0.10969	PROC		
0.66119	MRPL10	0.56541	MAN2B2	0.33547	SYNRG	0.15710	OSTBETA	0.10990	C6orf58		
0.66245	C20orf112	0.56649	SAP30L	0.33611	ZNF542	0.15740	C5orf46	0.11011	ZNF738		
0.66372	LOC100127980	0.56757	ZNF436	0.33675	EXOC5	0.15771	LEPREL1	0.11032	C14orf148		
0.66498	SAP30BP	0.56865	PDE12	0.33740	FUNDC2	0.15801	BTG1	0.11053	NCRNA00119		
0.66625	EGLN2	0.56973	INO80D	0.33804	GRIN2A	0.15831	TEP1	0.11074	SCARA5		
0.66751	CPNE1	0.57081	TMEM170B	0.33868	FKBP4	0.15861	ODF3	0.11095	LOC100130736		
0.66877	DAK	0.57189	USP13	0.33932	SNUPN	0.15891	MIOX	0.11116	PDX1		
0.67004	LOC149832	0.57297	LOC100288608	0.33996	RCN2	0.15921	CALCA	0.11137	XIRP2		
0.67130	LOC349196	0.57405	SOAT1	0.34060	UQCR10	0.15951	MGC15885	0.11158	JRK		
0.67257	C1QL2	0.57514	PCDHB15	0.34124	DAAM1	0.15981	ZP2	0.11179	KRT77		
0.67383	PRKAB1	0.57622	OSGIN2	0.34189	OAZ1	0.16011	DDX60L	0.11200	REN		
0.67509	CDK12	0.57730	INTS12	0.34253	CNOT1	0.16041	VWDE	0.11221	CCDC153		
0.67636	JAG2	0.57838	TMEM14C	0.34317	REPS2	0.16071	ZNF700	0.11242	KRT23		
0.67762	TRAPPC10	0.57946	HDDC2	0.34381	GRIN2B	0.16101	FLJ45482	0.11263	PLA2G4F		
0.67889	TADA2A	0.58054	SP4	0.34445	TXNL4A	0.16131	HBE1	0.11284	DUOX2		

0.68015	ZDHHC16	0.58162	ENY2	0.34509	CTDSPL2	0.16161	SGCG	0.11305	RAD54L
0.68142	DCAF5	0.58270	GTF2H5	0.34573	ME3	0.16191	GPR87	0.11326	PLCZ1
0.68268	DDX54	0.58378	LOC100294255	0.34638	DSTN	0.16221	MMP3	0.11347	EYS
0.68394	SLC29A3	0.58486	PUS7	0.34702	AHSA1	0.16251	C3orf67	0.11368	ANXA9
0.68521	DLX5	0.58595	CPSF3	0.34766	NSFL1C	0.16281	POU5F1	0.11389	IL32
0.68647	RASIP1	0.58703	DPH3	0.34830	SNX16	0.16311	MND1	0.11410	MYO7A
0.68774	MGLL	0.58811	LPHN3	0.34894	IDE	0.16341	GADL1	0.11431	LOC100129724
0.68900	LOC100133161	0.58919	PARL	0.34958	TXNL1	0.16371	TRDN	0.11452	LOC100128302
0.69027	POLR2A	0.59027	KIAA1191	0.35022	MAP2K1	0.16401	ENPEP	0.11473	CLEC12B
0.69153	FLJ42627	0.59135	CCDC90A	0.35087	KBTBD6	0.16431	UBQLN3	0.11494	DNHD1
0.69279	RALBP1	0.59243	GNB4	0.35151	AKAP10	0.16461	C21orf63	0.11515	LOC150051
0.69406	MYST1	0.59351	DCHS2	0.35215	HTR2A	0.16491	PROL1	0.11536	C14orf177
0.69532	FKBP8	0.59459	ADCK2	0.35279	LOC401504	0.16521	LOC389493	0.11557	SLC44A4
0.69659	C9orf37	0.59568	TXNDC9	0.35343	CDH2	0.16552	SLC2A4	0.11578	LOC646324
0.69785	C9orf9	0.59676	GUCY1A3	0.35407	VPS26B	0.16582	TRIM31	0.11599	ZNF761
0.69912	WRAP53	0.59784	ELL2	0.35471	FAM32A	0.16612	RAB27A	0.11620	STAB1
0.70038	CBFA2T2	0.59892	COMMD2	0.35536	EIF3M	0.16642	LOC729065	0.11641	PRICKLE3
0.70164	ACTR6	0.60000	KBTBD11	0.35600	HDHD2	0.16672	FLJ35776	0.11662	BIRC3
0.70291	ARHGAP39	0.60108	DNAJC19	0.35664	UROS	0.16702	ETAA1	0.11683	TECTB
0.70417	MYL9	0.60216	PDGFA	0.35728	NUP93	0.16732	RNASEH2A	0.11704	TMEM148
0.70544	SSH1	0.60324	C5orf41	0.35792	FSD1L	0.16762	C6orf163	0.11725	COL17A1
0.70670	SLC39A4	0.60432	TAF7	0.35856	PPP2R5E	0.16792	TSLP	0.11746	SERPINE3
0.70796	GAS7	0.60541	ADAP1	0.35920	NHP2L1	0.16822	IL18RAP	0.11767	BIRC5
0.70923	PC	0.60649	AHR	0.35985	RFPL1S	0.16852	FMO5	0.11788	CLDN8
0.71049	SFXN4	0.60757	CANX	0.36049	ACTR1A	0.16882	IL12A	0.11809	SVOPL
0.71176	MGC2752	0.60865	SRA1	0.36113	NDUFA6	0.16912	LOC730811	0.11830	OR10K1
0.71302	TXN2	0.60973	KIAA1109	0.36177	CRK	0.16942	C21orf82	0.11851	ZC3H12D
0.71429	ACCN1	0.61081	HECA	0.36241	COPZ1	0.16972	TMEM220	0.11872	LOC100289243
0.71555	PRELP	0.61189	ODZ3	0.36305	MRPL17	0.17002	SUGT1L1	0.11893	CACNA1S
0.71681	ING4	0.61297	EIF3I	0.36369	TSTA3	0.17032	CFP	0.11914	POF1B
0.71808	NR2F2	0.61405	PAICS	0.36434	ARL6IP1	0.17062	FBXL13	0.11935	ERMAP
0.71934	PLXNA3	0.61514	C1orf114	0.36498	GRIN2A	0.17092	C3orf77	0.11956	LOC100129417
0.72061	CCDC86	0.61622	TATDN2	0.36562	ANKS1B	0.17122	LIPH	0.11977	NR1I2
0.72187	MORC2	0.61730	NUP35	0.36626	GLT1D1	0.17152	MYH1	0.11998	SYTL1
0.72314	SCNM1	0.61838	SEMA3C	0.36690	CABYR	0.17182	GRHL1	0.12019	LOC100287484
0.72440	TACC2	0.61946	ST3GAL6	0.36754	LYRM5	0.17212	HIST2H2BF	0.12040	FLJ31662
0.72566	HOMEZ	0.62054	HLA-DPB2	0.36818	AHNAK2	0.17242	LOC100129940	0.12061	AIPL1
0.72693	SUV39H1	0.62162	KIAA0232	0.36883	DTD1	0.17272	GPR65	0.12082	AMHR2
0.72819	GALT	0.62270	SEMA3A	0.36947	MRPL46	0.17302	VCAM1	0.12103	KRTAP21-2
0.72946	KIAA1217	0.62378	NOM1	0.37011	CSNK2A2	0.17333	HDX	0.12124	SCN5A
0.73072	PGAM4	0.62486	CALN1	0.37075	NDUFA8	0.17363	NF1	0.12145	LOC100288073
0.73198	SDK2	0.62595	LOC100129309	0.37139	IMPA1	0.17393	FCF1	0.12166	C22orf45

0.73325	TOX	0.62703	ZBTB24	0.37203	ZDHHC15	0.17423	FLJ25996	0.12187	MYL10
0.73451	CCDC134	0.62811	RBMS1	0.37267	LRRC59	0.17453	C6orf217	0.12208	KRBA1
0.73578	ST3GAL1	0.62919	SOCS5	0.37332	INPP5F	0.17483	LOC400940	0.12229	MYO18B
0.73704	FRS3	0.63027	CWC27	0.37396	DIRAS2	0.17513	FRMPD2	0.12250	HK3
0.73831	TMEM200C	0.63135	STOX2	0.37460	COPB1	0.17543	SPTA1	0.12271	ACCN4
0.73957	FJX1	0.63243	HMBOX1	0.37524	OPTN	0.17573	LOC154860	0.12292	ELF3
0.74083	CHTF8	0.63351	SPAG16	0.37588	MRPL16	0.17603	KCNMB2	0.12314	CLCN1
0.74210	PACS2	0.63459	YOD1	0.37652	SCN2B	0.17633	DCDC5	0.12335	OLFML2A
0.74336	PEX5	0.63568	CCT3	0.37716	LOC100335030	0.17663	ST6GALNAC4	0.12356	FTCD
0.74463	HMGXB4	0.63676	ZBTB40	0.37781	ST8SIA3	0.17693	C9orf84	0.12377	SIRPD
0.74589	IGHMBP2	0.63784	DPY19L1	0.37845	ARL5B	0.17723	SLC1A7	0.12398	SPATA21
0.74716	LOC100130951	0.63892	TRIO	0.37909	UBAP1	0.17753	C14orf105	0.12419	BARHL2
0.74842	AMZ2P1	0.64000	C8orf40	0.37973	SV2B	0.17783	IKIP	0.12440	IL1RL2
0.74968	SPSB3	0.64108	SLC25A26	0.38037	X3258092	0.17813	DMBT1	0.12461	HSD3B2
0.75095	ZNF575	0.64216	PLA2G2C	0.38101	NEDD4L	0.17843	STL	0.12482	WT1
0.75221	LOC100130157	0.64324	CDH6	0.38165	EXOSC3	0.17873	DFNB59	0.12503	CARD10
0.75348	TCF20	0.64432	ACN9	0.38230	CNKSR2	0.17903	DNAJB7	0.12524	GSX2
0.75474	ZNF385C	0.64541	NDUFS4	0.38294	SAMD12	0.17933	ALPK1	0.12545	DQX1
0.75601	PDPR	0.64649	LAPTM4A	0.38358	SDCCAG1	0.17963	ARL6IP6	0.12566	WFDC10A
0.75727	C11orf93	0.64757	ZNF138	0.38422	KITLG	0.17993	RGS18	0.12587	HFE
0.75853	ZEB1	0.64865	GIN1	0.38486	X3947227	0.18023	HTR2B	0.12608	RPS14P3
0.75980	LOC646762	0.64973	SMAD1	0.38550	LINGO2	0.18053	LOXL3	0.12629	PKN3
0.76106	DDA1	0.65081	SESTD1	0.38614	PAPOLA	0.18084	LOC387771	0.12650	ANG
0.76233	DCAF11	0.65189	RAB5A	0.38679	SLC6A15	0.18114	SPTLC3	0.12671	HYAL3
0.76359	GPR120	0.65297	C7orf49	0.38743	PLDN	0.18144	LOC100130744	0.12692	PKD1L2
0.76485	ZNF256	0.65405	LYRM7	0.38807	TSFM	0.18174	MCF2L	0.12713	LOC283050
0.76612	SMARCB1	0.65514	PDE8B	0.38871	AUH	0.18204	CDRT1	0.12734	ZCCHC3
0.76738	KCTD10	0.65622	DUSP11	0.38935	SLC39A9	0.18234	CTHRC1	0.12755	C19orf48
0.76865	ZFP92	0.65730	IGSF8	0.38999	AAGAB	0.18264	MMP1	0.12776	CHRND
0.76991	ZNF319	0.65838	HSD17B11	0.39064	CAMK1D	0.18294	PSMB8	0.12797	LOC729505
0.77118	SLC7A4	0.65946	MPZL1	0.39128	GNPNAT1	0.18324	RBM44	0.12818	FOXA1
0.77244	ACTN4	0.66054	MAN1A1	0.39192	ZNF540	0.18354	FMO4	0.12839	SCUBE3
0.77370	SMAD4	0.66162	LOC100292534	0.39256	BBS4	0.18384	GAFA3	0.12860	BMP2
0.77497	GPR173	0.66270	BUD31	0.39320	LOC286052	0.18414	TMEM45A	0.12881	FGR
0.77623	XAB2	0.66378	C5orf36	0.39384	YWHAB	0.18444	SLCO5A1	0.12902	BUB1B
0.77750	TRIM66	0.66486	MTPN	0.39448	PRPF4	0.18474	CENPI	0.12923	LOC283501
0.77876	PGS1	0.66595	RC3H1	0.39513	EIF2B1	0.18504	AMPD1	0.12944	LOC100289675
0.78003	CCND1	0.66703	ADSS	0.39577	FAM175B	0.18534	COL21A1	0.12965	PTPRVP
0.78129	SMAD7	0.66811	SSR1	0.39641	BCAP31	0.18564	TRPV3	0.12986	LOC100288724
0.78255	ALKBH1	0.66919	ARIH2	0.39705	ARHGEF7	0.18594	ZNF506	0.13007	OAZ3
0.78382	LOC100289611	0.67027	MANF	0.39769	RAP2A	0.18624	GAFA2	0.13028	TCF3
0.78508	TINF2	0.67135	LOC100289398	0.39833	C16orf70	0.18654	TSHR	0.13049	KCNN4

0.78635	RASL10B			0.67243	ACTB	0.39897	CWC15		0.18684	RPL6	0.13070	HSPA7
0.78761	NMRAL1			0.67351	BBS5	0.39962	RUNDC1		0.18714	COMMD3	0.13091	HLA-DQB2
0.78887	C9orf172			0.67459	LOC151760	0.40026	AHNAK2		0.18744	ZNF138	0.13112	BSX
0.79014	TCF15			0.67568	C1orf156	0.40090	SNORD113-4		0.18774	PAPPA	0.13133	MUTYH
0.79140	IP6K1			0.67676	STK16	0.40154	C11orf63		0.18804	LAMC2	0.13154	OVOL2
0.79267	KCNF1			0.67784	PDIK1L	0.40218	SYT1		0.18834	LOC284825	0.13175	FBN3
0.79393	C7orf51			0.67892	BRPF1	0.40282	TLN2		0.18865	LOC100128739	0.13196	CXorf48
0.79520	DIDO1			0.68000	HOMER1	0.40346	DHX32		0.18895	CRNDE	0.13217	H19
0.79646	ASPHD1			0.68108	ARSB	0.40411	AP3M1		0.18925	LOC100129489	0.13238	KLHL6
0.79772	ZNF827			0.68216	HDLBP	0.40475	PSMG1		0.18955	LIN9	0.13259	TREML3
0.79899	NUP188			0.68324	LOC349114	0.40539	AMZ2		0.18985	FAM114A2	0.13280	SP140
0.80025	ASB6			0.68432	FBXO36	0.40603	PHF6		0.19015	C13orf38	0.13301	NFATC4
0.80152	NCRNA00086			0.68541	C2orf43	0.40667	PAFAH1B2		0.19045	GDNF	0.13322	GATA1
0.80278	C17orf65			0.68649	GRB10	0.40731	BCAS4		0.19075	POU1F1	0.13343	HIST1H2AA
0.80405	EIF3L			0.68757	AIDA	0.40795	PIP4K2C		0.19105	NCRNA00032	0.13364	TGM5
0.80531	TBKBP1			0.68865	SRFBP1	0.40860	CD59		0.19135	GIN1	0.13385	C12orf54
0.80657	RNF24			0.68973	PTCD2	0.40924	SYAP1		0.19165	KLHDC4	0.13406	C14orf86
0.80784	SLC32A1			0.69081	PVRL3	0.40988	RIT2		0.19195	MCOLN3	0.13427	TRAF4
0.80910	SNX19			0.69189	ADCY5	0.41052	CIRH1A		0.19225	DCAF13	0.13448	NCCRP1
0.81037	UPF1			0.69297	FSTL5	0.41116	SLC36A4		0.19255	LOC100133219	0.13469	GATA6
0.81163	DPF2			0.69405	PCDHB4	0.41180	STIP1		0.19285	SNRPE	0.13490	CEACAM19
0.81290	TMEM180			0.69514	PLEKHA8	0.41244	LOC441383		0.19315	WIBG	0.13511	WDR88
0.81416	AP3S2			0.69622	MFN2	0.41309	CYB5B		0.19345	DUSP23	0.13532	CRYGN
0.81542	FAM53B			0.69730	PDCD2	0.41373	BFAR		0.19375	DNAH14	0.13553	CD2
0.81669	THTPA			0.69838	ZNF518B	0.41437	TMEM66		0.19405	KNTC1	0.13574	PNLIPRP1
0.81795	NUDT14			0.69946	MMAA	0.41501	LOC158402		0.19435	BTN3A1	0.13595	LOX
0.81922	DIDO1			0.70054	STARD4	0.41565	FBXO16		0.19465	LOC100288260	0.13616	OR2Z1
0.82048	PRPF31			0.70162	HCG18	0.41629	MTHFD1		0.19495	LOC730338	0.13637	TNN
0.82174	C1orf93			0.70270	PAPD7	0.41693	EXOC6		0.19525	RASSF6	0.13658	KRT42P
0.82301	NDUFV3			0.70378	KIAA1109	0.41758	MCAT		0.19555	DDX60L	0.13679	ACTL7B
0.82427	PER2			0.70486	PPM1K	0.41822	ACAT1		0.19585	PDE5A	0.13700	C17orf46
0.82554	TBC1D10B			0.70595	NUDT9	0.41886	ZNF329		0.19616	WNT8A	0.13721	ATP2A1
0.82680	PEX12			0.70703	DCP1A	0.41950	STOML2		0.19646	NPSR1	0.13742	NFKB2
0.82807	PER1			0.70811	HAAO	0.42014	ZNF365		0.19676	IK	0.13763	LOC100128909
0.82933	EMD			0.70919	KAZ	0.42078	AIFM1		0.19706	MIR17	0.13784	FOLR1
0.83059	LOC100128398			0.71027	LOC100129550	0.42142	KLF12		0.19736	FLJ39080	0.13805	LOC100131801
0.83186	KSR1			0.71135	SRD5A3	0.42207	RSL24D1		0.19766	OR2AJ1	0.13826	SLC15A1
0.83312	RAB40A			0.71243	LOC550112	0.42271	DDX10		0.19796	RPL39L	0.13847	A4GNT
0.83439	IL4I1			0.71351	SEC62	0.42335	NCOA3		0.19826	FLJ44674	0.13868	NAPSA
0.83565	NLE1			0.71459	ZRANB1	0.42399	PANK2		0.19856	DCAF8L2	0.13889	C1orf147
0.83692	IGF1R			0.71568	LOC157503	0.42463	PTS		0.19886	MTBP	0.13910	RPS10
0.83818	IFNAR2			0.71676	C2orf72	0.42527	DDX3X		0.19916	C1orf158	0.13931	TNFAIP8

0.83944	LOC100128253	0.71784	C6orf115	0.42591	MLX	0.19946	CCHCR1	0.13953	IGF2AS
0.84071	ANXA11	0.71892	KIAA0895	0.42656	FAM188A	0.19976	AGBL2	0.13974	LOC148709
0.84197	PCDHB19P	0.72000	PTPRF	0.42720	CCDC53	0.20006	SERPINB10	0.13995	SCRIB
0.84324	KHNYN	0.72108	SATB2	0.42784	MED4	0.20036	MRGPRX2	0.14016	TP73
0.84450	BTBD12	0.72216	CDKN1A	0.42848	DDB1	0.20066	DPH5	0.14037	HNF4A
0.84576	NUDT19	0.72324	TMEM110	0.42912	MRPS16	0.20096	PARP12	0.14058	OBSCN
0.84703	COPE	0.72432	LOC643438	0.42976	ZNF295	0.20126	LOC728147	0.14079	ZNF90
0.84829	GPR124	0.72541	SPOCK1	0.43040	NCALD	0.20156	CCDC110	0.14100	NRADDP
0.84956	SEC16A	0.72649	FAM40B	0.43105	GSTO1	0.20186	C10orf67	0.14121	LOC284551
0.85082	LRRC37B	0.72757	CCT5	0.43169	DIS3L	0.20216	IHH	0.14142	COL4A6
0.85209	INO80C	0.72865	CASP9	0.43233	PANX1	0.20246	LGSN	0.14163	TEAD2
0.85335	POLR2I	0.72973	RAP2B	0.43297	ZNF483	0.20276	SASS6	0.14184	TGM1
0.85461	PRMT1	0.73081	ENO1	0.43361	SOD1	0.20306	CASP12	0.14205	OR1J1
0.85588	KCNIP3	0.73189	ANAPC10	0.43425	APOOL	0.20336	CD302	0.14226	C6orf15
0.85714	ZNF418	0.73297	CDK7	0.43489	DLST	0.20366	LOC439938	0.14247	CSNK1G1
0.85841	SH2B2	0.73405	RUVBL1	0.43554	C14orf104	0.20397	SLC15A5	0.14268	CLDN2
0.85967	MAX	0.73514	SCRN1	0.43618	DLEU7	0.20427	ZNF578	0.14289	MRPL27
0.86094	GNL3L	0.73622	KCNAB1	0.43682	CBL	0.20457	METTL8	0.14310	YSK4
0.86220	ADAMTS8	0.73730	CEPT1	0.43746	PANK1	0.20487	MST1P9	0.14331	PRSS37
0.86346	DMWD	0.73838	DCAF8	0.43810	C16orf80	0.20517	FANCI	0.14352	BPESC1
0.86473	C19orf70	0.73946	FAM50A	0.43874	ATP5C1	0.20547	SETD8	0.14373	TRIM34
0.86599	TMEM107	0.74054	LOC441086	0.43938	SUDS3	0.20577	PARP9	0.14394	GPR112
0.86726	LRRC4B	0.74162	ZNF518B	0.44003	FAM19A2	0.20607	CXCL10	0.14415	IQCH
0.86852	ANKRD13B	0.74270	GCC1	0.44067	ABCB7	0.20637	BLID	0.14436	COL4A2
0.86979	MRPL30	0.74378	TBC1D1	0.44131	NCOA2	0.20667	FAM111A	0.14457	NES
0.87105	SHROOM3	0.74486	ATP5F1	0.44195	NAA30	0.20697	SERPINB8	0.14478	LOC100131673
0.87231	FREM3	0.74595	MTERF	0.44259	KPNB1	0.20727	C4orf47	0.14499	GCGR
0.87358	LOC100130027	0.74703	UTP3	0.44323	ARID5B	0.20757	CXorf21	0.14520	SPAG11A
0.87484	GAPDHL7	0.74811	BRIX1	0.44387	TM9SF3	0.20787	RP2	0.14541	GYLTL1B
0.87611	C11orf1	0.74919	AGPAT6	0.44452	DOK5	0.20817	ARHGEF35	0.14562	IGDCC3
0.87737	MOB2	0.75027	NOP14	0.44516	C14orf101	0.20847	QTRTD1	0.14583	PAX1
0.87863	MRPL14	0.75135	PID1	0.44580	FSD1L	0.20877	ASIP	0.14604	COL3A1
0.87990	TRAF3	0.75243	ARL5A	0.44644	ST8SIA1	0.20907	WDR63	0.14625	SLC16A11
0.88116	U2AF2	0.75351	C6orf64	0.44708	OGFOD1	0.20937	LOC100133994	0.14646	TSPO2
0.88243	LOC728003	0.75459	IDH1	0.44772	C15orf23	0.20967	GPR126	0.14667	DOCK4
0.88369	PNMAL2	0.75568	TMEM170B	0.44836	CTSB	0.20997	LOC729806	0.14688	LOC284260
0.88496	EXTL1	0.75676	C5orf41	0.44901	PNMA3	0.21027	TP53	0.14709	CELA2B
0.88622	DCAF12L2	0.75784	MDH1B	0.44965	DERL2	0.21057	FAM3D	0.14730	APOBEC3D
0.88748	FKBP15	0.75892	ACOT13	0.45029	SV2B	0.21087	LOC100130357	0.14751	TTLL6
0.88875	FREM3	0.76000	ATAD2B	0.45093	PTPN9	0.21117	SVEP1	0.14772	SLC43A1
0.89001	FAM100B	0.76108	DMRTB1	0.45157	GPR137C	0.21147	LIMS1	0.14793	OR6C74
0.89128	NR1H2	0.76216	BAG2	0.45221	JKAMP	0.21178	BCHE	0.14814	LOC645277

0.89254	BRMS1	0.76324	TMEM170B	0.45285	UBE2D1	0.21208	FANCM	0.14835	CAV3
0.89381	CRLF1	0.76432	MGAT4B	0.45350	RRM1	0.21238	AKD1	0.14856	LOC728317
0.89507	FAM113A	0.76541	LOC100132345	0.45414	UBQLN2	0.21268	FAM46A	0.14877	PRRT4
0.89633	NCOA5	0.76649	NUS1	0.45478	RFX7	0.21298	ANKHD1	0.14898	NEU4
0.89760	ZNF587	0.76757	TOR1AIP1	0.45542	INPP5A	0.21328	CLPS	0.14919	ZPLD1
0.89886	RFT1	0.76865	STMN1	0.45606	THOC5	0.21358	LOC284900	0.14940	LILRB5
0.90013	ADAMTSL2	0.76973	PTPRG	0.45670	GMPR2	0.21388	ANKRD39	0.14961	ITGA2B
0.90139	GTF2A2	0.77081	AFAP1	0.45734	FMN1	0.21418	FAM69A	0.14982	FLJ46134
0.90265	DUSP18	0.77189	RNF2	0.45799	GANAB	0.21448	CCDC141	0.15003	MAGEB6
0.90392	ZDHHC7	0.77297	WTAP	0.45863	SON	0.21478	C6orf26	0.15024	RNH1
0.90518	HSD17B1	0.77405	CCNYL1	0.45927	RASGEF1A	0.21508	TMEM45B	0.15045	KRT37
0.90645	KIF27	0.77514	RN28S1	0.45991	DNAJC28	0.21538	TXLNB	0.15066	EFCAB8
0.90771	CNNM3	0.77622	ATXN1	0.46055	RRAGB	0.21568	SHMT1	0.15087	ACOT12
0.90898	SEMA5B	0.77730	ZNF518B	0.46119	MOSPD1	0.21598	TRA@	0.15108	TLR8
0.91024	GDF10	0.77838	AMMECR1L	0.46183	PCMTD1	0.21628	HAUS6	0.15129	C12orf70
0.91150	CCDC85C	0.77946	LOC729088	0.46248	TPD52L2	0.21658	PRL	0.15150	ADAMTS6
0.91277	FOXF2	0.78054	ZRANB1	0.46312	TXNL4B	0.21688	NRK	0.15171	LOC729966
0.91403	GADD45G	0.78162	LOC401321	0.46376	TRMT6	0.21718	C1orf50	0.15192	C1orf106
0.91530	LOC100129773	0.78270	GGPS1	0.46440	DSCR3	0.21748	IMPDH1	0.15213	NPHS1
0.91656	BICC1	0.78378	HIST1H2BD	0.46504	C12orf5	0.21778	IRX5	0.15234	CDH3
0.91783	PCDHB19P	0.78486	COG2	0.46568	POLR2C	0.21808	GLI2	0.15255	MOV10L1
0.91909	HEATR7A	0.78595	C1orf58	0.46632	GRM5	0.21838	FLJ20444	0.15276	TEX14
0.92035	JAZF1	0.78703	PINK1	0.46697	VAPA	0.21868	DKFZp434J0226	0.15297	TNS4
0.92162	ISOC2	0.78811	FAM13A	0.46761	TCP11L1	0.21898	NXF5	0.15318	C21orf7
0.92288	NAV1	0.78919	RBM15B	0.46825	KIAA0391	0.21929	LOC339260	0.15339	ERN2
0.92415	CCDC85C	0.79027	C4orf34	0.46889	SESN3	0.21959	MORC4	0.15360	OGFOD2
0.92541	KIAA0232	0.79135	GBE1	0.46953	GPR107	0.21989	C10orf115	0.15381	DKFZp779M0652
0.92668	TBP	0.79243	PPAT	0.47017	TMEM132B	0.22019	FAM122C	0.15402	IQCD
0.92794	ZNF506	0.79351	FAM47E	0.47081	NUDCD1	0.22049	DBF4	0.15423	TEAD4
0.92920	SERHL	0.79459	EPHB2	0.47146	KIAA1377	0.22079	C11orf73	0.15444	FLJ30430
0.93047	GRK6	0.79568	GORASP1	0.47210	VBP1	0.22109	FLJ30901	0.15465	SDF4
0.93173	ZNF185	0.79676	NSUN5	0.47274	C17orf81	0.22139	SPAM1	0.15486	MAMDC4
0.93300	AGBL5	0.79784	RGNEF	0.47338	SLITRK4	0.22169	LOC374443	0.15507	PAPL
0.93426	LOC729218	0.79892	INO80D	0.47402	MRPL40	0.22199	USP50	0.15528	GNLY
0.93552	NYNRIN	0.80000	PNO1	0.47466	NFAT5	0.22229	LOC284669	0.15549	KRT24
0.93679	LOC100289415	0.80108	PINK1	0.47530	CCDC34	0.22259	MUSK	0.15570	OR10G6
0.93805	SHC2	0.80216	ZNF518B	0.47595	TPD52	0.22289	C11orf95	0.15592	EXD3
0.93932	DIDO1	0.80324	KHDRBS1	0.47659	PTPRM	0.22319	ST6GALNAC3	0.15613	SLC25A18
0.94058	TMCC2	0.80432	UBXN2B	0.47723	PPPDE2	0.22349	DSCR8	0.15634	C21orf128
0.94185	TRAPPC2	0.80541	ERV3	0.47787	NANS	0.22379	LCT	0.15655	CD3EAP
0.94311	GLT25D1	0.80649	FAM110C	0.47851	TMED2	0.22409	TMEM194B	0.15676	SLC36A2
0.94437	LOC284395	0.80757	FAM105A	0.47915	SRXN1	0.22439	LOC100131217	0.15697	LOC730179

0.94564	RPL28	0.80865	RRP15	0.47979	MED21	0.22469	ASB12	0.15718	THAP8
0.94690	HTR6	0.80973	SH3RF1	0.48044	BEX5	0.22499	TTC25	0.15739	PCK2
0.94817	MTX1	0.81081	HNRNPA0	0.48108	SENP8	0.22529	ZNF573	0.15760	ZNF850P
0.94943	TBC1D16	0.81189	ACVR1	0.48172	GUCY1A2	0.22559	CCDC67	0.15781	ENAM
0.95070	FAM120AOS	0.81297	LOC442155	0.48236	ARNTL2	0.22589	GIMAP6	0.15802	CDCA5
0.95196	VIPR1	0.81405	GOLPH3	0.48300	ZNF264	0.22619	FAM198A	0.15823	AZI1
0.95322	ECH1	0.81514	GALNT13	0.48364	GNE	0.22649	TSPAN12	0.15844	KRT15
0.95449	DIDO1	0.81622	CACNA1E	0.48428	ADCYAP1	0.22679	LOC440570	0.15865	LOC647286
0.95575	SLC7A3	0.81730	LOC157503	0.48493	PRDX3	0.22710	PRIM2	0.15886	SPHK1
0.95702	PARP16	0.81838	MKI67IP	0.48557	MAPK1	0.22740	PRR7	0.15907	PRSS36
0.95828	LOH12CR1	0.81946	RC3H1	0.48621	TTC8	0.22770	LOC100128093	0.15928	NT5DC4
0.95954	EXPH5	0.82054	LOC100287359	0.48685	LARP4B	0.22800	LOC100132529	0.15949	PSMD4
0.96081	C12orf47	0.82162	TMEM170B	0.48749	UTP18	0.22830	MAP7D3	0.15970	ATHL1
0.96207	HHLA3	0.82270	HERPUD2	0.48813	TOMM22	0.22860	ZNF578	0.15991	ZP4
0.96334	ADRA2A	0.82378	ZBTB2	0.48877	PLA2G15	0.22890	GPR64	0.16012	GP9
0.96460	NKAIN1	0.82486	MUL1	0.48942	USP25	0.22920	ETV1	0.16033	DNAH10
0.96587	NS3BP	0.82595	HOXB4	0.49006	ADAT1	0.22950	PDC	0.16054	LOC149620
0.96713	IER5L	0.82703	FAM169A	0.49070	HELZ	0.22980	POU4F2	0.16075	KRTAP10-7
0.96839	MIF	0.82811	LOC100287552	0.49134	MCTS1	0.23010	CPA2	0.16096	TEX28
0.96966	PAX6	0.82919	FLJ36848	0.49198	MTMR2	0.23040	C1orf162	0.16117	LOC154092
0.97092	CDR1	0.83027	CCDC115	0.49262	API5	0.23070	UFM1	0.16138	VSIG10L
0.97219	C7orf50	0.83135	C6orf162	0.49326	REPS2	0.23100	DHDPSL	0.16159	GUCY2F
0.97345	CISD3	0.83243	PNRC1	0.49391	KGFLP2	0.23130	TFAP2A	0.16180	G6PC
0.97472	FLJ34208	0.83351	C2orf40	0.49455	NDUFB6	0.23160	GAGE1	0.16201	LOC256374
0.97598	ELMO2	0.83459	FCRL4	0.49519	SAMD12	0.23190	LOC100291860	0.16222	LOC649133
0.97724	RCOR2	0.83568	CCDC93	0.49583	MPP7	0.23220	ZIC5	0.16243	PLB1
0.97851	NPTX2	0.83676	RPL22	0.49647	ADNP2	0.23250	NYNRIN	0.16264	TREML4
0.97977	TEX10	0.83784	FLJ42709	0.49711	LRRTM3	0.23280	LIMS1	0.16285	IKZF1
0.98104	FOXK1	0.83892	C1orf159	0.49775	CREBL2	0.23310	FLJ37638	0.16306	LTF
0.98230	KIAA0247	0.84000	ZEB2	0.49840	GALK2	0.23340	HNRNPL	0.16327	DCST2
0.98357	ZNF70	0.84108	SHQ1	0.49904	C11orf41	0.23370	IQCF5	0.16348	FMO2
0.98483	LOC100288226	0.84216	METTL5	0.49968	ALKBH5	0.23400	MST4	0.16369	VGLL2
0.98609	LOC100131296	0.84324	PLEKHO1	0.50032	ZNF585A	0.23430	LOC730100	0.16390	TAT
0.98736	SORD	0.84432	GPRIN3	0.50096	MID2	0.23460	DNASE2B	0.16411	ELN
0.98862	C1orf51	0.84541	JAGN1	0.50160	DENR	0.23491	MCM3APAS	0.16432	STRA6
0.98989	RNF40	0.84649	GATAD2B	0.50225	ECD	0.23521	TMC1	0.16453	DND1
0.99115	DNAJC14	0.84757	LOC401093	0.50289	DKK3	0.23551	LOC100130642	0.16474	MYO1G
0.99241	PPP1R15A	0.84865	MOSC2	0.50353	TCEAL8	0.23581	CDK1	0.16495	PEX11A
0.99368	KIF2A	0.84973	TMCO4	0.50417	FLRT3	0.23611	BCL2L15	0.16516	CATSPERG
0.99494	IGF1R	0.85081	TTC33	0.50481	DDX52	0.23641	PTPRQ	0.16537	SIGLEC9
0.99621	MAP3K12	0.85189	L3MBTL3	0.50545	LOC100128510	0.23671	MDP1	0.16558	TMIGD2
0.99747	PCDH17	0.85297	KIAA2018	0.50609	RAD51C	0.23701	PLAG1	0.16579	LOC503519

0.99874	STARD9					0.85405	ZBTB8OS	0.50674	SC5DL			0.23731	CCT6P3	0.16600	CYB561D2
						0.85514	HIST1H4C	0.50738	DCUN1D5			0.23761	ZNF556	0.16621	LAMA5
						0.85622	TYW1	0.50802	GNAL			0.23791	ADPGK	0.16642	LOC613206
						0.85730	JUN	0.50866	ZNF484			0.23821	RP11-90M2.3	0.16663	ZBTB7B
						0.85838	CRIM1	0.50930	TRAPPC4			0.23851	ARID3B	0.16684	ANKRD62
						0.85946	ZFYVE16	0.50994	ABHD13			0.23881	C1orf150	0.16705	OR10R3P
						0.86054	FLJ39534	0.51058	FDX1			0.23911	LOC283658	0.16726	COL22A1
						0.86162	GTDC1	0.51123	C17orf79			0.23941	ESPN	0.16747	FCRL3
						0.86270	SLC35E2	0.51187	PTPN3			0.23971	RPL3	0.16768	NHLRC4
						0.86378	GAR1	0.51251	VAPB			0.24001	CDKN2C	0.16789	CCDC120
						0.86486	POLD2	0.51315	CENPN			0.24031	PHACTR4	0.16810	KRT37
						0.86595	LOC730101	0.51379	MRPS11			0.24061	PRR3	0.16831	AIRE
						0.86703	DNAH6	0.51443	SH3BGRL			0.24091	ZPBP	0.16852	HMGCS2
						0.86811	SYNPO2	0.51507	HSD17B12			0.24121	SVIL	0.16873	GPR109A
						0.86919	CYB5RL	0.51572	KCNRG			0.24151	FAM149B2	0.16894	HEATR7B1
						0.87027	PCDHB6	0.51636	RBBP6			0.24181	LOC100130451	0.16915	MYBL2
						0.87135	C7orf25	0.51700	CCDC47			0.24211	LOC728003	0.16936	ATP4A
						0.87243	C8orf33	0.51764	CHMP1B			0.24242	FAM105A	0.16957	PLIN1
						0.87351	PARP3	0.51828	ANKRD34C			0.24272	TLE4	0.16978	SEMA3G
						0.87459	ETV3	0.51892	CDKL1			0.24302	RPLP0P2	0.16999	RPGRIP1
						0.87568	LOC100131311	0.51956	LOC646214			0.24332	FLJ35379	0.17020	COL4A3
						0.87676	C4orf42	0.52021	SAR1A			0.24362	GLP1R	0.17041	WDR73
						0.87784	C1orf183	0.52085	PINX1			0.24392	ICA1L	0.17062	FLJ16171
						0.87892	ZNF141	0.52149	NETO1			0.24422	C20orf72	0.17083	TBX4
						0.88000	PPP2R2C	0.52213	LSM4			0.24452	WBP2NL	0.17104	ACP5
						0.88108	LOC348751	0.52277	PPP1CC			0.24482	BTF3	0.17125	TLCD2
						0.88216	STRA13	0.52341	LOC550643			0.24512	SLC26A2	0.17146	C1orf213
						0.88324	CRADD	0.52405	NDUFC2			0.24542	AFARP1	0.17167	AURKB
						0.88432	LPL	0.52470	JOSD1			0.24572	TRIP13	0.17188	TERT
						0.88541	C6orf203	0.52534	NTRK3			0.24602	S100A14	0.17209	C19orf41
						0.88649	MCM9	0.52598	DCAF4			0.24632	ASB18	0.17231	MARVELD2
						0.88757	HMBS	0.52662	GDE1			0.24662	SLC25A5	0.17252	OR4X1
						0.88865	LNPEP	0.52726	STRBP			0.24692	RGS22	0.17273	SERPINA9
						0.88973	MTPN	0.52790	WNK3			0.24722	MAGEB18	0.17294	NUP210L
						0.89081	ZNF264	0.52854	PTGR1			0.24752	GPX5	0.17315	COX6A2
						0.89189	LOC100128822	0.52919	DRP2			0.24782	OR10C1	0.17336	HOXB5
						0.89297	GNG4	0.52983	ARMC1			0.24812	FAM129A	0.17357	OR52N1
						0.89405	TMEM170B	0.53047	C20orf3			0.24842	HIST1H2BL	0.17378	LOC730134
						0.89514	YWHAQP8	0.53111	DNTTIP1			0.24872	OBFC2B	0.17399	PRR15
						0.89622	AURKC	0.53175	PDPK1			0.24902	H2BFWT	0.17420	SYNGR4
						0.89730	LOC100127984	0.53239	NDUFAF1			0.24932	ZNF77	0.17441	MB
						0.89838	LOC286002	0.53303	MAPRE2			0.24962	AKT2	0.17462	C16orf86

0.89946	CAMK1	0.53368	L2HGDH	0.24992	LOC643441	0.17483	SLC5A5
0.90054	TMEM221	0.53432	GLIPR1	0.25023	THSD1	0.17504	GYS2
0.90162	CAP1	0.53496	TMEM188	0.25053	VANGL1	0.17525	ACAP1
0.90270	PMS2CL	0.53560	ST8SIA3	0.25083	FAM167B	0.17546	LOC100287704
0.90378	KIF1B	0.53624	GARNL1	0.25113	IL20RB	0.17567	IQGAP2
0.90486	C8orf38	0.53688	PIP5K1B	0.25143	EP400NL	0.17588	LOC100289527
0.90595	RPP38	0.53752	STAMBPL1	0.25173	LHX8	0.17609	MKI67
0.90703	GPR88	0.53817	USP35	0.25203	LOC344328	0.17630	FLJ43763
0.90811	GPR107	0.53881	MAGED2	0.25233	EED	0.17651	IRX3
0.90919	GCNT4	0.53945	PGRMC1	0.25263	POM121L2	0.17672	C15orf42
0.91027	BCL11B	0.54009	PPFIA2	0.25293	HESX1	0.17693	KRT74
0.91135	C2orf79	0.54073	FMN1	0.25323	MYEF2	0.17714	ANGPTL5
0.91243	FKRP	0.54137	FEM1B	0.25353	ATXN8OS	0.17735	TRIM7
0.91351	CELF3	0.54201	CDK17	0.25383	ATP8A2	0.17756	KRT20
0.91459	COL25A1	0.54266	HDHD1A	0.25413	ENO3	0.17777	ACOT6
0.91568	FOXO3	0.54330	KCNMA1	0.25443	FLJ46321	0.17798	ITPR3
0.91676	FRG1	0.54394	ANK3	0.25473	IRS1	0.17819	ANKLE1
0.91784	FLAD1	0.54458	USP27X	0.25503	PATE2	0.17840	KLHDC7B
0.91892	LOC100129434	0.54522	NIPSNAP3A	0.25533	FLJ10661	0.17861	KRTAP10-1
0.92000	MOXD1	0.54586	PSMC3	0.25563	DYDC1	0.17882	C6orf94
0.92108	SCP2	0.54650	C10orf84	0.25593	MLN	0.17903	ZNF491
0.92216	RBM6	0.54715	GRIN2A	0.25623	LOC727947	0.17924	EIF4E1B
0.92324	LOC644285	0.54779	PPP4R4	0.25653	IMMP1L	0.17945	C3orf20
0.92432	LYRM7	0.54843	PSD3	0.25683	DEFB1	0.17966	TEC
0.92541	UBE2E3	0.54907	CPD	0.25713	SNHG5	0.17987	GALK1
0.92649	RBM22	0.54971	ZFP90	0.25743	CTLA4	0.18008	LOC100129365
0.92757	RBBP4	0.55035	ATMIN	0.25774	POLG2	0.18029	LOC440518
0.92865	ATAD2B	0.55099	VPS26A	0.25804	PPPDE1	0.18050	CYP1A2
0.92973	AKR1E2	0.55164	TM2D3	0.25834	FAM103A1	0.18071	MUC13
0.93081	LOC221710	0.55228	HIST4H4	0.25864	C1orf87	0.18092	SLC12A9
0.93189	LOC339822	0.55292	SNX12	0.25894	ZNF468	0.18113	LAT
0.93297	TSPAN32	0.55356	ACER3	0.25924	ITGBL1	0.18134	TBX6
0.93405	CIR1	0.55420	ARMCX3	0.25954	TTLL9	0.18155	DNAH2
0.93514	PRDM2	0.55484	LONP2	0.25984	GALNT8	0.18176	SLC25A34
0.93622	TYW1B	0.55548	C12orf29	0.26014	ATAD2B	0.18197	LOC388428
0.93730	TRIM11	0.55613	UQCRB	0.26044	CYP4Z2P	0.18218	FAM108A1
0.93838	METTL8	0.55677	C12orf51	0.26074	ABCG8	0.18239	C22orf34
0.93946	CDKN2D	0.55741	IGBP1	0.26104	SYTL3	0.18260	DOK2
0.94054	NT5DC2	0.55805	C15orf24	0.26134	LOC151658	0.18281	PDIA2
0.94162	B3GNT2	0.55869	MTRF1	0.26164	SNORA33	0.18302	TDRD1
0.94270	ARL6IP4	0.55933	DOK6	0.26194	PMFBP1	0.18323	SLC17A4
0.94378	ZNF773	0.55997	CYLD	0.26224	C15orf63	0.18344	TOP2A

			0.94486	TMEM74	0.56062	HSDL1		0.26254	SGOL2	0.18365	CLEC12A
			0.94595	C19orf29	0.56126	ZNF720		0.26284	ZNF525	0.18386	PABPN1L
			0.94703	MRPS18B	0.56190	ELAVL1		0.26314	ZC3H8	0.18407	G6PC2
			0.94811	PLXNA2	0.56254	HSP90B1		0.26344	CSRP3	0.18428	C16orf73
			0.94919	CDK11A	0.56318	RNF185		0.26374	LOC100289019	0.18449	VWCE
			0.95027	JAKMIP1	0.56382	C12orf5		0.26404	LEPRE1	0.18470	IL10
			0.95135	LOC257396	0.56446	MSRB2		0.26434	FHDC1	0.18491	ZNF212
			0.95243	C8orf85	0.56511	ISCA2		0.26464	OR51S1	0.18512	MYH2
			0.95351	GRIK3	0.56575	ENOX1		0.26494	TACR3	0.18533	BST1
			0.95459	GPATCH4	0.56639	FUNDC1		0.26524	APAF1	0.18554	FLJ30698
			0.95568	LZTR1	0.56703	C14orf142		0.26555	DYNLRB2	0.18575	LACRT
			0.95676	INGX	0.56767	DDX6		0.26585	C13orf16	0.18596	PROCR
			0.95784	LOC401127	0.56831	BBS10		0.26615	FAS	0.18617	MYCBPAP
			0.95892	HIST1H2BG	0.56895	OSTF1		0.26645	SLC25A24	0.18638	GUCY2GP
			0.96000	PCDHB3	0.56960	DDX47		0.26675	GPR183	0.18659	SLC4A9
			0.96108	C12orf64	0.57024	RAB6A		0.26705	BCL2L10	0.18680	LEMD1
			0.96216	LOC157503	0.57088	ST8SIA3		0.26735	RPGR	0.18701	KIAA0495
			0.96324	ZMYM6	0.57152	UBE2G1		0.26765	PRTG	0.18722	UPK1A
			0.96432	LOC100129195	0.57216	TMX3		0.26795	FAM160A1	0.18743	GP2
			0.96541	DYRK2	0.57280	CCT8		0.26825	KIF26B	0.18764	P2RY4
			0.96649	LOC100289340	0.57344	SCAI		0.26855	OTOP1	0.18785	CHAT
			0.96757	EPPK1	0.57409	CCNA1		0.26885	KCTD9	0.18806	F7
			0.96865	SFRS2B	0.57473	LOC144438		0.26915	GKAP1	0.18827	FANCA
			0.96973	ZNF749	0.57537	NTRK3		0.26945	CCDC138	0.18848	ATXN7L2
			0.97081	TRIM40	0.57601	CBLN4		0.26975	CHEK1	0.18870	EFCAB4B
			0.97189	FOXR1	0.57665	GART		0.27005	ACY1	0.18891	LCK
			0.97297	CBLN1	0.57729	STRN3		0.27035	FANCI	0.18912	LOC100290265
			0.97405	ZNF468	0.57793	CNTN5		0.27065	ACAA1	0.18933	BPIL1
			0.97514	ARTN	0.57858	C17orf75		0.27095	PARD6G	0.18954	TTTY5
			0.97622	KALRN	0.57922	SLC38A2		0.27125	G3BP1	0.18975	MGC4473
			0.97730	GADD45A	0.57986	RORA		0.27155	FLJ37505	0.18996	ADRA2B
			0.97838	C4orf27	0.58050	GABARAPL1		0.27185	DYNLT1	0.19017	DENND1C
			0.97946	TMEM178	0.58114	CLIP1		0.27215	DSE	0.19038	CMTM7
			0.98054	LOC100287803	0.58178	TRPC4AP		0.27245	TMC7	0.19059	STK11IP
			0.98162	GRIK3	0.58242	TAF13		0.27275	VANGL1	0.19080	C11orf86
			0.98270	TMEM168	0.58307	AHNAK2		0.27305	UBAP2	0.19101	TMPRSS7
			0.98378	RAB3B	0.58371	MTMR7		0.27336	KLRC4	0.19122	FHOD1
			0.98486	PLXNA2	0.58435	LOC100129781		0.27366	CCNB2	0.19143	MUC16
			0.98595	CDKL4	0.58499	AGPAT3		0.27396	AFG3L1	0.19164	TNFRSF19
			0.98703	KCNJ8	0.58563	NMT2		0.27426	ASCL3	0.19185	CSN1S2A
			0.98811	DGKG	0.58627	MTMR7		0.27456	ZCWPW2	0.19206	GNAT2
			0.98919	SSR1	0.58691	C9orf80		0.27486	RPS24	0.19227	SLAMF9

0.99027	GRIK3	0.58756	KATNAL1	0.27516	UTP20	0.19248	KCNK17
0.99135	PBX2	0.58820	HEXIM1	0.27546	FKBP15	0.19269	SMC6
0.99243	ADORA2A	0.58884	IMPA1	0.27576	LOC100289292	0.19290	GPR142
0.99351	ERVK2	0.58948	IMP3	0.27606	SNORD7	0.19311	OR2T8
0.99459	GAST	0.59012	NGFRAP1	0.27636	ZNF764	0.19332	FER1L5
0.99568	LOC100289441	0.59076	KLF8	0.27666	CHST7	0.19353	C8B
0.99676	PINK1	0.59140	PAG1	0.27696	ZNF215	0.19374	SLC14A2
0.99784	LOC284023	0.59205	SMC2	0.27726	SDC1	0.19395	C10orf122
0.99892	LOC100131581	0.59269	GRB2	0.27756	TLCD1	0.19416	FZD2
		0.59333	VIPAR	0.27786	TMEM182	0.19437	INTS3
		0.59397	ZNF91	0.27816	RRH	0.19458	UTS2D
		0.59461	DHPS	0.27846	DDX60	0.19479	LOC100130673
		0.59525	SLC2A13	0.27876	IL28A	0.19500	CD1C
		0.59589	CDH13	0.27906	FILIP1L	0.19521	LOC100292665
		0.59654	LOC96610	0.27936	DLK2	0.19542	COL11A2
		0.59718	PTPRO	0.27966	PXDNL	0.19563	OR2H2
		0.59782	BECN1	0.27996	KLRA1	0.19584	MYOM3
		0.59846	ALDH3A2	0.28026	ZKSCAN1	0.19605	CHIA
		0.59910	LOC286367	0.28056	C9orf131	0.19626	GRHL3
		0.59974	GFOD2	0.28087	RPH3AL	0.19647	SHCBP1
		0.60038	ZNF32	0.28117	KCNU1	0.19668	C20orf114
		0.60103	PSMG2	0.28147	MAF1	0.19689	TNNT1
		0.60167	NPLOC4	0.28177	BTBD16	0.19710	LRRC17
		0.60231	LOC399959	0.28207	PLEKHA7	0.19731	CD70
		0.60295	ALG1	0.28237	C21orf81	0.19752	FCN3
		0.60359	METTL4	0.28267	LOC400756	0.19773	ABCA4
		0.60423	XRCC6BP1	0.28297	C20orf30	0.19794	NLRC5
		0.60487	NDN	0.28327	GOLGA6L9	0.19815	CGA
		0.60552	ANKRD16	0.28357	TIFA	0.19836	MST1R
		0.60616	EXOSC5	0.28387	FREM1	0.19857	RARRES2
		0.60680	FXC1	0.28417	LOC348840	0.19878	OVCH1
		0.60744	TRMT112	0.28447	ZNF580	0.19899	C17orf93
		0.60808	JRKL	0.28477	ZNF197	0.19920	LOC255130
		0.60872	PSMD8	0.28507	ULBP1	0.19941	C20orf185
		0.60936	TIMM8B	0.28537	TNFRSF10D	0.19962	C1QTNF6
		0.61001	GLCE	0.28567	NEK5	0.19983	TRPM5
		0.61065	PCCA	0.28597	TXNRD3	0.20004	LOC100128615
		0.61129	LYSMD2	0.28627	ZUFSP	0.20025	TRPM4
		0.61193	LIN52	0.28657	LOC729041	0.20046	RIBC1
		0.61257	OS9	0.28687	CCDC148	0.20067	ABCC3
		0.61321	MTMR1	0.28717	COX8C	0.20088	LOC400622
		0.61386	FLRT2	0.28747	TRIM38	0.20109	LCE4A

				0.61450	C14orf156		0.28777	C17orf67	0.20130	HHIPL2
				0.61514	SCN3B		0.28807	SOHLH2	0.20151	LOC440461
				0.61578	CDADC1		0.28837	LOC100131851	0.20172	NHLH2
				0.61642	SLC2A3		0.28868	BTK	0.20193	NPY6R
				0.61706	MAPK6		0.28898	GNGT1	0.20214	IL1F9
				0.61770	LYRM1		0.28928	LOC100289536	0.20235	SPINT1
				0.61835	HAGH		0.28958	FLT3	0.20256	DCAF4L2
				0.61899	MEGF8		0.28988	GPR27	0.20277	RETNLB
				0.61963	MPPED2		0.29018	ORMDL3	0.20298	FGF6
				0.62027	LOC158696		0.29048	ARPC5	0.20319	MIRLET7B
				0.62091	CCDC56		0.29078	LOC285540	0.20340	ZG16B
				0.62155	PSMA7		0.29108	ZNF525	0.20361	SLAMF8
				0.62219	LOC149832		0.29138	ZNF765	0.20382	ARID5A
				0.62284	BTBD3		0.29168	PPP4R1L	0.20403	PHF21B
				0.62348	ELAC2		0.29198	ZNF566	0.20424	PRDM9
				0.62412	RRN3		0.29228	TTC38	0.20445	CTSW
				0.62476	KLHL28		0.29258	TBX21	0.20466	DAOA
				0.62540	SLC2A14		0.29288	LOC100128841	0.20487	ZNF783
				0.62604	SRRD		0.29318	ALKBH8	0.20509	SLC4A1
				0.62668	NDUFB8		0.29348	SLC35D1	0.20530	LOC727930
				0.62733	PDRG1		0.29378	FLJ10038	0.20551	PRSS33
				0.62797	OTUD5		0.29408	C18orf56	0.20572	GBP3
				0.62861	PRCP		0.29438	AGBL3	0.20593	MYLK2
				0.62925	SECISBP2		0.29468	LMOD3	0.20614	TNK1
				0.62989	USE1		0.29498	ATG4A	0.20635	LOC283332
				0.63053	LOC100379224		0.29528	FREM1	0.20656	CCDC74B
				0.63117	MASTL		0.29558	TTC27	0.20677	CD274
				0.63182	HINT2		0.29588	S100A12	0.20698	LOC100129845
				0.63246	TMEM106C		0.29619	X3766013	0.20719	FBXO24
				0.63310	PURG		0.29649	LOC339929	0.20740	SLC23A1
				0.63374	DUSP26		0.29679	C15orf5	0.20761	KRTAP10-5
				0.63438	KCTD2		0.29709	CNTD1	0.20782	CLCN2
				0.63502	SLC2A13		0.29739	VSX1	0.20803	ZNF446
				0.63566	GNPTAB		0.29769	LOC100291776	0.20824	PCOLCE
				0.63631	SERPINF1		0.29799	ZNF557	0.20845	LOC100290860
				0.63695	LOC147670		0.29829	CALML4	0.20866	PTPRH
				0.63759	SLC30A4		0.29859	CCDC150	0.20887	SIGLEC6
				0.63823	FOXK2		0.29889	MT1X	0.20908	RPS7
				0.63887	TMEM65		0.29919	PHB	0.20929	DAPK2
				0.63951	PTPRE		0.29949	SPINK9	0.20950	LOC100133029
				0.64015	RFK		0.29979	CXorf42	0.20971	TCOF1
				0.64080	DCC		0.30009	RIPK1	0.20992	IL17B

				0.64144	HERC2		0.30039	OR5B3	0.21013	IRF7
				0.64208	C8orf76		0.30069	GTF2A1L	0.21034	OPLAH
				0.64272	HS6ST2		0.30099	C2orf65	0.21055	FLJ31958
				0.64336	NUP50		0.30129	LIPC	0.21076	LOC146429
				0.64400	PARG		0.30159	ZNF569	0.21097	LOC730227
				0.64464	GNG2		0.30189	AGPAT6	0.21118	FBN2
				0.64529	CCDC91		0.30219	PAPOLB	0.21139	HSPC157
				0.64593	MRPL51		0.30249	SEC61G	0.21160	LRP5
				0.64657	TRMT2B		0.30279	RASA4	0.21181	DKFZP434L187
				0.64721	PRKAR1A		0.30309	DDX31	0.21202	C17orf53
				0.64785	FBXO22		0.30339	LOC644841	0.21223	LOC100287213
				0.64849	DYNLL1		0.30369	WDR76	0.21244	SCNN1G
				0.64913	CSNK1G1		0.30400	FSIP1	0.21265	C1orf92
				0.64978	SIP1		0.30430	YBX1	0.21286	C10orf62
				0.65042	ALDH1B1		0.30460	KMO	0.21307	NUBP2
				0.65106	CHD6		0.30490	PRDXDD1P	0.21328	SLC30A8
				0.65170	UVRAG		0.30520	LOC400965	0.21349	LRCH4
				0.65234	TXLNG		0.30550	LOC100131117	0.21370	TAC4
				0.65298	MORN4		0.30580	GK2	0.21391	CD79B
				0.65362	KATNAL1		0.30610	THAP5	0.21412	C16orf47
				0.65427	C18orf8		0.30640	DDX50	0.21433	HLX
				0.65491	COMMD9		0.30670	FP6628	0.21454	TRPV6
				0.65555	DAPK3		0.30700	LOC100130345	0.21475	PLG
				0.65619	COX6C		0.30730	MTFMT	0.21496	OR5V1
				0.65683	TDP1		0.30760	CXorf58	0.21517	HLA-A
				0.65747	TMEM97		0.30790	WFDC6	0.21538	LOC148638
				0.65811	ATAD2		0.30820	ZNF534	0.21559	CYP21A2
				0.65876	NGDN		0.30850	C15orf56	0.21580	APOA2
				0.65940	CDH7		0.30880	IFI44	0.21601	FER1L4
				0.66004	VMA21		0.30910	BUB1	0.21622	GFI1B
				0.66068	TMEM14D		0.30940	SYCP2L	0.21643	CEACAM22P
				0.66132	IL34		0.30970	C5orf53	0.21664	FGD3
				0.66196	COX4I1		0.31000	LOC644727	0.21685	TBC1D10C
				0.66260	PTPRD		0.31030	GPX6	0.21706	SOLH
				0.66325	C12orf49		0.31060	BNC2	0.21727	BTNL9
				0.66389	ZNF134		0.31090	ZNF684	0.21748	SAG
				0.66453	ZRANB1		0.31120	DOPEY1	0.21769	GPCRLTM7
				0.66517	ATP5O		0.31150	RPS15A	0.21790	LAT2
				0.66581	TMTC1		0.31181	LOC440288	0.21811	MAP3K15
				0.66645	LOC729082		0.31211	C20orf94	0.21832	SLC17A9
				0.66709	ZBTB43		0.31241	GNMT	0.21853	FAM179A
				0.66774	FBXO18		0.31271	ZNF831	0.21874	POTEC

0.66838	C10orf76	0.31301	SNHG1	0.21895	CAPN8
0.66902	HDAC8	0.31331	LOC644196	0.21916	C21orf29
0.66966	ARMCX2	0.31361	ZNF420	0.21937	LOC100134360
0.67030	NUDT7	0.31391	DNAH14	0.21958	JPH2
0.67094	RORA	0.31421	FLJ33065	0.21979	ARMCX4
0.67158	IER3IP1	0.31451	C9orf11	0.22000	FLJ39609
0.67223	IDI1	0.31481	C6orf141	0.22021	PRDM13
0.67287	RPS6KA6	0.31511	ALG5	0.22042	LOC100133047
0.67351	FAM127A	0.31541	RORA	0.22063	CCL19
0.67415	KLHL22	0.31571	C21orf45	0.22084	FGFBP1
0.67479	COX10	0.31601	RPL32	0.22105	LOC644070
0.67543	CAMK1D	0.31631	PQLC3	0.22126	GPR114
0.67607	SPTAN1	0.31661	C20orf56	0.22148	PPM1F
0.67672	C9orf40	0.31691	DDX55	0.22169	CNGA2
0.67736	EXOC5	0.31721	FKBP9	0.22190	KIF22
0.67800	PDXK	0.31751	LSM14B	0.22211	PAQR5
0.67864	ZNF136	0.31781	AP4E1	0.22232	CD160
0.67928	GPR12	0.31811	TAF3	0.22253	CKAP2L
0.67992	SERGEF	0.31841	MST1P9	0.22274	APOBEC4
0.68056	FAM98B	0.31871	SPATA5	0.22295	SSPO
0.68121	FLJ41278	0.31901	RNF148	0.22316	SLC17A8
0.68185	XBP1	0.31932	PPP1R1C	0.22337	HABP2
0.68249	TMEM117	0.31962	BCL2L11	0.22358	CD6
0.68313	NCAM2	0.31992	DISC1	0.22379	TAS2R39
0.68377	NT5C3L	0.32022	DARS2	0.22400	LOC200726
0.68441	LSM14B	0.32052	IMPG2	0.22421	SIX1
0.68505	STX5	0.32082	ARMC4	0.22442	CDC20B
0.68570	C18orf19	0.32112	ZNF696	0.22463	LOC642574
0.68634	ZNF383	0.32142	BTN3A3	0.22484	HYAL1
0.68698	WNT3	0.32172	ELAC1	0.22505	MT1E
0.68762	LEO1	0.32202	FAP	0.22526	DDX4
0.68826	AKAP11	0.32232	DSCC1	0.22547	SUN5
0.68890	USMG5	0.32262	NANOS1	0.22568	UGT3A2
0.68954	MINPP1	0.32292	LOC100129335	0.22589	LCE1B
0.69019	AHNAK2	0.32322	MOSC1	0.22610	STAC3
0.69083	ZNF8	0.32352	BDKRB2	0.22631	WDR38
0.69147	C10orf35	0.32382	SPATA1	0.22652	SPRR2G
0.69211	RDH11	0.32412	FAM150B	0.22673	C20orf200
0.69275	C17orf100	0.32442	GLYATL3	0.22694	GSTT1
0.69339	TMEM90B	0.32472	CDCA7L	0.22715	LOC100289602
0.69403	VPS33B	0.32502	LOC100129236	0.22736	BET3L
0.69468	GTF3C4	0.32532	MECOM	0.22757	SCIN

0.69532	C11orf46	0.32562	PRTG	0.22778	CXorf30
0.69596	ALG8	0.32592	FAM194A	0.22799	DMC1
0.69660	RRAGA	0.32622	ITGA7	0.22820	ABCC6
0.69724	DCUN1D2	0.32652	LOC100131431	0.22841	MARCO
0.69788	POLR3F	0.32682	SCN9A	0.22862	PPIL5
0.69852	CCDC92	0.32713	SMTN	0.22883	GUCY2D
0.69917	KIF5A	0.32743	CASP1	0.22904	MAPK15
0.69981	ERP44	0.32773	FGF2	0.22925	CD300LG
0.70045	NKX3-1	0.32803	SERPINB1	0.22946	LOC442028
0.70109	RAD23B	0.32833	C17orf68	0.22967	PPIEL
0.70173	HSPA14	0.32863	ARSF	0.22988	OR5AP2
0.70237	LMO7	0.32893	HEMK1	0.23009	TAS1R1
0.70301	TP53BP1	0.32923	HGF	0.23030	FGF23
0.70366	TMEM135	0.32953	C20orf199	0.23051	LOC100130256
0.70430	MBTPS1	0.32983	LOC400986	0.23072	ASTL
0.70494	GPN3	0.33013	HSD17B8	0.23093	SMO
0.70558	MGC5566	0.33043	LOC100131512	0.23114	ERLEC1P1
0.70622	AKAP5	0.33073	ZXDA	0.23135	MS4A10
0.70686	MGAT3	0.33103	CRYGS	0.23156	LOC730036
0.70750	GABRA5	0.33133	ZNF678	0.23177	CAPN14
0.70815	TRAPPC5	0.33163	C5orf36	0.23198	LAX1
0.70879	GABARAP	0.33193	PRSS23	0.23219	OR4Q2
0.70943	KATNAL1	0.33223	FBXO5	0.23240	WTIP
0.71007	ZNF681	0.33253	MRPL55	0.23261	LOC100287955
0.71071	MUM1L1	0.33283	ARL5C	0.23282	COL4A4
0.71135	LOC727896	0.33313	ERI1	0.23303	CNGA3
0.71199	NUP98	0.33343	KIAA0485	0.23324	GGT6
0.71264	C10orf18	0.33373	NPR3	0.23345	SLC38A4
0.71328	SLC11A2	0.33403	SUMO4	0.23366	SLC9A3
0.71392	NDUFS3	0.33433	TMEM50A	0.23387	CAPN9
0.71456	TACO1	0.33464	GCOM1	0.23408	ZNF205
0.71520	MFHAS1	0.33494	ZNF254	0.23429	RPS18
0.71584	AHNAK2	0.33524	LOC100233209	0.23450	MUC4
0.71648	DIABLO	0.33554	LOC100288937	0.23471	C6orf54
0.71713	ANKRD27	0.33584	PRSS53	0.23492	TRAIP
0.71777	ETS2	0.33614	RPL23	0.23513	TMEM212
0.71841	ATP5L	0.33644	CHD1	0.23534	PSPN
0.71905	C1QBP	0.33674	CCDC125	0.23555	C4orf26
0.71969	DKFZp761E198	0.33704	COL8A1	0.23576	SAMD11
0.72033	AACS	0.33734	CC2D2B	0.23597	ATP13A5
0.72097	TRUB2	0.33764	C3orf35	0.23618	OR5W2
0.72162	TMEM199	0.33794	IMMP2L	0.23639	C2

				0.72226	DIDO1		0.33824	LOC100216479	0.23660	LOC388630
				0.72290	C1QL3		0.33854	ST7OT4	0.23681	C14orf180
				0.72354	ZNF583		0.33884	CORIN	0.23702	GPR144
				0.72418	RNF219		0.33914	TNIP1	0.23723	RNF222
				0.72482	PSMB3		0.33944	PAX6	0.23744	TULP1
				0.72547	DGKH		0.33974	RPTN	0.23765	KRTAP3-2
				0.72611	RPP25		0.34004	LRAT	0.23787	GUCA2A
				0.72675	KGFLP2		0.34034	CASP2	0.23808	MYLPF
				0.72739	C12orf62		0.34064	PIK3CG	0.23829	GCNT3
				0.72803	C19orf42		0.34094	BCL2L15	0.23850	ARHGEF5
				0.72867	FGF13		0.34124	TFB1M	0.23871	H1FOO
				0.72931	BDNF		0.34154	UMPS	0.23892	PLBD1
				0.72996	SNORD114-26		0.34184	FERMT1	0.23913	NLRP11
				0.73060	PPFIA3		0.34214	TTC12	0.23934	LINGO4
				0.73124	RERG		0.34245	MDFIC	0.23955	PAX4
				0.73188	RAB39		0.34275	CLTCL1	0.23976	SLC1A5
				0.73252	EPHA6		0.34305	DDX59	0.23997	RGS3
				0.73316	STT3A		0.34335	SPAG1	0.24018	C1orf224
				0.73380	CRNKL1		0.34365	LOC388456	0.24039	ITK
				0.73445	CYB5D1		0.34395	HINT1	0.24060	ATF5
				0.73509	MRPL21		0.34425	LMNB1	0.24081	TRMT61A
				0.73573	SAT2		0.34455	ZCCHC10	0.24102	NAT8B
				0.73637	ZNF749		0.34485	MLL3	0.24123	FLJ14100
				0.73701	BC036928		0.34515	IQGAP3	0.24144	RNF113A
				0.73765	SAE1		0.34545	DAPL1	0.24165	LOC646471
				0.73829	COMMD7		0.34575	SYCP2	0.24186	FAM170A
				0.73894	LCOR		0.34605	IQCF3	0.24207	TTLL10
				0.73958	CCDC3		0.34635	ENPP1	0.24228	LOC100134368
				0.74022	RBBP7		0.34665	TRIM54	0.24249	LOC100128531
				0.74086	BEX5		0.34695	C12orf26	0.24270	CYP2F1
				0.74150	DONSON		0.34725	LOC613126	0.24291	XDH
				0.74214	SNAPC5		0.34755	SRF	0.24312	MAGEA8
				0.74278	C14orf169		0.34785	ALPI	0.24333	TRAPPC6A
				0.74343	CHCHD1		0.34815	LOC100131673	0.24354	VIT
				0.74407	C11orf31		0.34845	NID1	0.24375	ZDHHC19
				0.74471	C17orf71		0.34875	C7orf63	0.24396	SLFN12L
				0.74535	LOC90110		0.34905	SHISA5	0.24417	PSMG4
				0.74599	CACNA1C		0.34935	HSF2	0.24438	NR0B1
				0.74663	MAGEH1		0.34965	C9orf142	0.24459	CYP24A1
				0.74727	GOSR1		0.34995	BTG3	0.24480	MFAP5
				0.74792	STYK1		0.35026	CDC7	0.24501	INSRR
				0.74856	B9D1		0.35056	ENGASE	0.24522	X3678343

				0.74920	LETMD1		0.35086	FAM20A	0.24543	ZMYND15
				0.74984	VPS29		0.35116	KATNA1	0.24564	LOC729569
				0.75048	CBX7		0.35146	ZNF428	0.24585	HSF5
				0.75112	KLHL28		0.35176	TRIM63	0.24606	LOC100129411
				0.75176	SSR4		0.35206	PLSCR1	0.24627	SMYD1
				0.75241	VWC2		0.35236	LOC283711	0.24648	SBSN
				0.75305	FDX1L		0.35266	KATNAL2	0.24669	FGD2
				0.75369	CKAP4		0.35296	C21orf49	0.24690	BTBD19
				0.75433	C14orf166		0.35326	ZNF835	0.24711	C21orf58
				0.75497	CPNE8		0.35356	EDEM2	0.24732	LOC100129223
				0.75561	NFIL3		0.35386	HELQ	0.24753	LOC220077
				0.75625	UBE2Z		0.35416	BATF3	0.24774	BOLA1
				0.75690	KIF5A		0.35446	SLC9A11	0.24795	EMILIN3
				0.75754	DHX8		0.35476	WNT4	0.24816	SPTBN5
				0.75818	FBXL14		0.35506	FGFBP3	0.24837	DNAH10
				0.75882	LOC728392		0.35536	LOC286189	0.24858	HOXB3
				0.75946	STXBP6		0.35566	CEP76	0.24879	PKD1L3
				0.76010	FAM161B		0.35596	CCDC111	0.24900	MYBPHL
				0.76074	SNX11		0.35626	LOC100287440	0.24921	DAB1
				0.76139	KCTD17		0.35656	BTN3A2	0.24942	LOC646982
				0.76203	NDNL2		0.35686	C3orf75	0.24963	ADAM5P
				0.76267	LOC728653		0.35716	WDR49	0.24984	AMELX
				0.76331	EIF2B2		0.35746	C8orf58	0.25005	LOC441897
				0.76395	GNA15		0.35777	RHBDF1	0.25026	TDRD10
				0.76459	C11orf74		0.35807	ST6GALNAC6	0.25047	KIF24
				0.76523	USP6NL		0.35837	BCCIP	0.25068	INO80B
				0.76588	YWHAE		0.35867	CHRNB1	0.25089	LIN28A
				0.76652	HS3ST4		0.35897	HOPX	0.25110	COL13A1
				0.76716	KCNRG		0.35927	EYA4	0.25131	GSG1
				0.76780	SAPS2		0.35957	ART3	0.25152	KRT27
				0.76844	SPIN3		0.35987	ACBD6	0.25173	OR6T1
				0.76908	C9orf116		0.36017	C2orf63	0.25194	C6orf52
				0.76972	LGI1		0.36047	LOC441666	0.25215	TULP2
				0.77037	HINT1		0.36077	GPX7	0.25236	TREH
				0.77101	SPINT2		0.36107	LOC644714	0.25257	FLJ46361
				0.77165	PSAP		0.36137	PHKG1	0.25278	PSAPL1
				0.77229	C9orf69		0.36167	RPL9	0.25299	CALHM2
				0.77293	KIAA0406		0.36197	OSTN	0.25320	LOC283904
				0.77357	TMEM135		0.36227	RNF43	0.25341	C4orf40
				0.77421	WDR47		0.36257	NEB	0.25362	LIMS2
				0.77486	TMEM9B		0.36287	EFCAB5	0.25383	LOC340113
				0.77550	UBA52		0.36317	FAM13A	0.25404	RAB41

0.77614	CPSF6	0.36347	PLCD4	0.25426	PFKFB4
0.77678	MGC23284	0.36377	C12orf55	0.25447	LOC100289074
0.77742	EXD2	0.36407	ATF3	0.25468	LOC283875
0.77806	SDR16C5	0.36437	MMP2	0.25489	GATA3
0.77870	ZFYVE27	0.36467	RRAGD	0.25510	GLYCTK
0.77935	NUP50	0.36497	TMEM99	0.25531	LOC100287737
0.77999	PPEF1	0.36527	LXN	0.25552	C1orf130
0.78063	MRPS7	0.36558	C20orf26	0.25573	PCNAP1
0.78127	DCC	0.36588	MSTO1	0.25594	CCR8
0.78191	YEATS4	0.36618	CCDC163P	0.25615	LOC100131973
0.78255	SFRS2B	0.36648	MTHFS	0.25636	CHEK2
0.78319	LOC283267	0.36678	GDPD2	0.25657	LOC100289629
0.78384	MRRF	0.36708	FZD4	0.25678	ASF1B
0.78448	AGPAT3	0.36738	HIST1H2BF	0.25699	C16orf72
0.78512	GLE1	0.36768	LOC645355	0.25720	SRMS
0.78576	GABRA5	0.36798	ZXDA	0.25741	C9orf57
0.78640	LOC642345	0.36828	EIF4B	0.25762	FLJ39061
0.78704	LIG3	0.36858	C1orf98	0.25783	ABCB11
0.78768	CXorf40A	0.36888	CHCHD5	0.25804	ATP5J2
0.78833	BCL7A	0.36918	PRCD	0.25825	RPL29P2
0.78897	UPRT	0.36948	ABCA5	0.25846	KIF4A
0.78961	ZNF253	0.36978	TRIM16	0.25867	TBPL2
0.79025	CARS	0.37008	PFDN1	0.25888	PAGE5
0.79089	GSPT2	0.37038	ANGPT1	0.25909	C7orf66
0.79153	ETHE1	0.37068	PRO2852	0.25930	IL5RA
0.79217	FECH	0.37098	MAP3K1	0.25951	FLJ27243
0.79282	GTF3A	0.37128	LOC283682	0.25972	SSPO
0.79346	ZDHHC6	0.37158	GALNT3	0.25993	SLC6A20
0.79410	SMUG1	0.37188	LOC100130345	0.26014	LOC100291212
0.79474	COQ6	0.37218	LOC678655	0.26035	AMAC1
0.79538	FBXL20	0.37248	CTBS	0.26056	TAAR9
0.79602	H2AFY2	0.37278	DDX58	0.26077	LOC100292959
0.79666	NHS	0.37309	C4orf23	0.26098	C1orf116
0.79731	FAT3	0.37339	ZNF187	0.26119	ALS2CL
0.79795	ZNF304	0.37369	PDGFB	0.26140	NEUROG2
0.79859	PLEKHJ1	0.37399	ASB7	0.26161	MYO15A
0.79923	LOC100288114	0.37429	LOC399815	0.26182	FBXL6
0.79987	LOC100294416	0.37459	CER1	0.26203	CCDC26
0.80051	BEX2	0.37489	ILKAP	0.26224	PHOX2B
0.80115	IGF1	0.37519	SPEF1	0.26245	GBP7
0.80180	C18orf21	0.37549	PDAP1	0.26266	MBD3L3
0.80244	CETN2	0.37579	ACADL	0.26287	SLC6A18

				0.80308	TMPO		0.37609	TBX19	0.26308	DHX34
				0.80372	SORCS1		0.37639	ZNF701	0.26329	SH2D3A
				0.80436	NME2		0.37669	LOC100132738	0.26350	SC65
				0.80500	TUBGCP3		0.37699	SVEP1	0.26371	SSPO
				0.80564	LOC286272		0.37729	SLC22A25	0.26392	FSD2
				0.80629	ZNF610		0.37759	FUS	0.26413	GPR3
				0.80693	MRPL49		0.37789	C17orf60	0.26434	LOC100131373
				0.80757	RMI1		0.37819	LOC642340	0.26455	MIR1287
				0.80821	TSPAN31		0.37849	GALNT12	0.26476	LOC100294360
				0.80885	ZNF667		0.37879	MURC	0.26497	TNFSF8
				0.80949	MCTS1		0.37909	MAFK	0.26518	PLA2G4E
				0.81013	ZNF566		0.37939	FAM72A	0.26539	SEMA4G
				0.81078	GLG1		0.37969	CCDC123	0.26560	LOC440934
				0.81142	USP10		0.37999	LOC100132832	0.26581	LOC157931
				0.81206	YAF2		0.38029	FLJ37060	0.26602	C14orf176
				0.81270	MRPL28		0.38059	MDM4	0.26623	OTX1
				0.81334	BLMH		0.38090	ILK	0.26644	LY6G5C
				0.81398	ZNF506		0.38120	GLIPR1L2	0.26665	SCEL
				0.81462	MIR21		0.38150	STAP1	0.26686	NR2E3
				0.81527	TMEM50B		0.38180	WDR52	0.26707	HYALP1
				0.81591	MRPS34		0.38210	C9orf68	0.26728	IL9
				0.81655	EXOSC2		0.38240	SH3BP5L	0.26749	LOC100133817
				0.81719	GAD2		0.38270	LOC100289565	0.26770	CRNN
				0.81783	MPRIP		0.38300	DNAH14	0.26791	COL29A1
				0.81847	NDUFB10		0.38330	MCM8	0.26812	NFATC1
				0.81911	RBM41		0.38360	CCDC104	0.26833	CYBA
				0.81976	ASPDH		0.38390	LOC149351	0.26854	LIN28B
				0.82040	EDF1		0.38420	OGN	0.26875	LOC100128334
				0.82104	KLHL28		0.38450	ATAD2B	0.26896	CCDC85B
				0.82168	THNSL1		0.38480	LOC100130345	0.26917	LOC643810
				0.82232	CELF6		0.38510	CYP2U1	0.26938	LOC100131726
				0.82296	CREB3		0.38540	C17orf42	0.26959	APOM
				0.82360	SORCS3		0.38570	ARL13A	0.26980	GP1BA
				0.82425	DAPK1		0.38600	ZNF551	0.27001	GUCY2C
				0.82489	EID2B		0.38630	MYT1	0.27022	LOC643387
				0.82553	DRG2		0.38660	ZNF107	0.27043	OR6C68
				0.82617	ZNF132		0.38690	KBTBD10	0.27065	LOC728716
				0.82681	PTPRR		0.38720	ITGA4	0.27086	NANOS1
				0.82745	URM1		0.38750	TAS2R14	0.27107	ITLN2
				0.82809	TIMM23		0.38780	NFU1	0.27128	C5orf17
				0.82874	GALNT1		0.38810	MTMR14	0.27149	LOC100130698
				0.82938	ZNF41		0.38840	ZIC4	0.27170	LOC339529

0.83002	ATP6V1G1	0.38871	HCG8	0.27191	GOLGA4
0.83066	C20orf11	0.38901	IKBKB	0.27212	ROBO3
0.83130	TIMM8A	0.38931	ZNF702P	0.27233	GJA3
0.83194	FAM171A1	0.38961	LOC645261	0.27254	SDCBP2
0.83258	LYRM7	0.38991	LOC441268	0.27275	OR7E47P
0.83323	SLC25A14	0.39021	NEXN	0.27296	RHCG
0.83387	TOM1L1	0.39051	SPATA22	0.27317	MXRA5
0.83451	GPR12	0.39081	C15orf51	0.27338	LOC286154
0.83515	ZNF263	0.39111	C2orf77	0.27359	LOC399904
0.83579	METTL9	0.39141	CREB1	0.27380	OR52E8
0.83643	LRRC4C	0.39171	LOC642924	0.27401	SNHG11
0.83708	TOX4	0.39201	LRRIQ1	0.27422	C7orf47
0.83772	UNC13C	0.39231	ZC3HAV1L	0.27443	SSPO
0.83836	PPP2R3C	0.39261	LOC100132963	0.27464	LOC100128655
0.83900	LOC643837	0.39291	C9orf130	0.27485	POU2F2
0.83964	LNX2	0.39321	CCR6	0.27506	CD163L1
0.84028	MBNL2	0.39351	SMARCE1	0.27527	CHRNA9
0.84092	RBBP9	0.39381	SNCAIP	0.27548	TRIM72
0.84157	ZNF673	0.39411	PARP14	0.27569	ZIC4
0.84221	PARD6B	0.39441	IL11RA	0.27590	KRT76
0.84285	CENPN	0.39471	NFKBIL1	0.27611	PGPEP1L
0.84349	KIAA1024	0.39501	ZNF169	0.27632	KIF26A
0.84413	AHNAK2	0.39531	PSG9	0.27653	GOLGA6L2
0.84477	HIST1H1A	0.39561	SMC4	0.27674	RHBG
0.84541	C16orf13	0.39591	RUNX2	0.27695	LOC643486
0.84606	LSM1	0.39622	HELZ	0.27716	EPYC
0.84670	RNF126	0.39652	LOC441795	0.27737	DKFZP434K028
0.84734	CTSF	0.39682	ANKRD32	0.27758	NADK
0.84798	SLC5A3	0.39712	TTN	0.27779	TMEM139
0.84862	CANT1	0.39742	TNFAIP3	0.27800	PROK2
0.84926	BCL2L13	0.39772	NKX1-2	0.27821	TTC21A
0.84990	FLJ10038	0.39802	C14orf138	0.27842	IL16
0.85055	ZNF774	0.39832	MACROD2	0.27863	D2HGDH
0.85119	ZNF175	0.39862	METTL2B	0.27884	ABHD14B
0.85183	ANO3	0.39892	ZNF287	0.27905	GSTTP1
0.85247	CAPS2	0.39922	LRRIQ4	0.27926	WDR83
0.85311	DIDO1	0.39952	SMAGP	0.27947	LOC100130430
0.85375	ZNF529	0.39982	SEPHS1	0.27968	SLC35A2
0.85439	ZNF331	0.40012	HOXA3	0.27989	AKR1CL1
0.85504	ODZ1	0.40042	C14orf64	0.28010	HAUS5
0.85568	LRRTM3	0.40072	EGFLAM	0.28031	EMILIN1
0.85632	LACTB	0.40102	BPI	0.28052	NPR1

0.85696	SFRS2B	0.40132	PYCR2	0.28073	CCT8L2
0.85760	DNAJA4	0.40162	MOSPD3	0.28094	PAX2
0.85824	IFIT1	0.40192	MDM1	0.28115	C1orf46
0.85888	CHMP1A	0.40222	ZNF552	0.28136	TACC3
0.85953	LOC643529	0.40252	LOC100287689	0.28157	WFDC13
0.86017	C9orf123	0.40282	NDUFAF2	0.28178	MMP11
0.86081	NELF	0.40312	PXDNL	0.28199	ZDHHC12
0.86145	B3GALTL	0.40342	HSD17B7P2	0.28220	SPNS3
0.86209	ZNF468	0.40372	TBX2	0.28241	TAPT1
0.86273	TOM1L1	0.40403	ZFP37	0.28262	ALX1
0.86337	DYNLL2	0.40433	FANCI	0.28283	SLC2A2
0.86402	NEU3	0.40463	LOC100271722	0.28304	ZBTB22
0.86466	CPNE7	0.40493	HLA-DPB2	0.28325	SCGB2A2
0.86530	EARS2	0.40523	GSTCD	0.28346	HAVCR1
0.86594	BRI3BP	0.40553	SYDE2	0.28367	LOC100128494
0.86658	SIN3B	0.40583	TRDMT1	0.28388	LRRC19
0.86722	BAG5	0.40613	PRG4	0.28409	TCTE1
0.86786	TMED3	0.40643	ALDH8A1	0.28430	FCAMR
0.86851	CCNB1IP1	0.40673	FLJ10213	0.28451	MTTP
0.86915	ABHD12B	0.40703	IQCK	0.28472	ZNF579
0.86979	ZNF557	0.40733	SLAMF6	0.28493	MYBPC2
0.87043	LOC84856	0.40763	ADAMTS5	0.28514	ACOX2
0.87107	PBX3	0.40793	OSTN	0.28535	SERPINA10
0.87171	FAM122A	0.40823	IL10RB	0.28556	CSF2RB
0.87235	TMEM147	0.40853	TRAM1L1	0.28577	LCN8
0.87300	TMEM205	0.40883	CD36	0.28598	LOC728228
0.87364	RSPO2	0.40913	ZSCAN2	0.28619	IQCA1L
0.87428	PCDHB19P	0.40943	TMEM126B	0.28640	NAALADL1
0.87492	MYO16	0.40973	LOC100132919	0.28661	FOLR4
0.87556	DHX35	0.41003	ZNF781	0.28682	MTMR8
0.87620	C9orf6	0.41033	FAM188B2	0.28704	PP14571
0.87684	SMPX	0.41063	ARMCX5	0.28725	ST8SIA2
0.87749	KIF5A	0.41093	LOC100216001	0.28746	H3F3A
0.87813	TLL2	0.41123	C21orf62	0.28767	HEJ1
0.87877	UBR7	0.41153	VOPP1	0.28788	TMEM198
0.87941	C21orf34	0.41184	LOC100289473	0.28809	C19orf75
0.88005	GAS2	0.41214	SCN7A	0.28830	NAGS
0.88069	ZNF780B	0.41244	KIAA1804	0.28851	GIMAP8
0.88133	SMARCA1	0.41274	WBP11P1	0.28872	KERA
0.88198	THUMPD1	0.41304	ST7L	0.28893	LOC100290696
0.88262	DOK6	0.41334	MGC45800	0.28914	MYH13
0.88326	WFDC1	0.41364	AKD1	0.28935	NINL

				0.88390	LOC400456		0.41394	ARHGEF33	0.28956	LOC100129072
				0.88454	ZNF629		0.41424	RANBP17	0.28977	LOC440337
				0.88518	ZNF189		0.41454	GBP1	0.28998	RFX1
				0.88582	RABEPK		0.41484	LOC642103	0.29019	ELANE
				0.88647	ARMCX1		0.41514	MICA	0.29040	KCNK16
				0.88711	SEMA6D		0.41544	C9orf23	0.29061	GPR111
				0.88775	FXN		0.41574	GUK1	0.29082	CLC
				0.88839	PDCD7		0.41604	ZNHIT3	0.29103	FGF7
				0.88903	ARMCX5		0.41634	ZWILCH	0.29124	RPL3L
				0.88967	ZNF264		0.41664	C17orf37	0.29145	ZBTB17
				0.89031	CLCN5		0.41694	MBL2	0.29166	LRRC70
				0.89096	LOC729860		0.41724	FAM18B	0.29187	TFAP2B
				0.89160	X3418513		0.41754	ZNF389	0.29208	CHTF8
				0.89224	RHOXF1		0.41784	ZNF737	0.29229	LOC642587
				0.89288	NRP1		0.41814	WDR31	0.29250	SLC34A2
				0.89352	SPG7		0.41844	SPDYE3	0.29271	HBQ1
				0.89416	NUDT22		0.41874	GALNT7	0.29292	JUB
				0.89480	TFAM		0.41904	RNF34	0.29313	GJD2
				0.89545	LOC100288738		0.41935	EMP2	0.29334	LOC283079
				0.89609	ZNF490		0.41965	MIG7	0.29355	LOC100128590
				0.89673	CLUL1		0.41995	CLGN	0.29376	GJA5
				0.89737	STK24		0.42025	PMM2	0.29397	PADI1
				0.89801	GRIN3A		0.42055	KLHL34	0.29418	SPN
				0.89865	KARS		0.42085	NPFF	0.29439	SYCE1L
				0.89929	COG4		0.42115	CLIC6	0.29460	IMP5
				0.89994	C10orf76		0.42145	EPSTI1	0.29481	LOC100130951
				0.90058	PTCHD1		0.42175	DOCK6	0.29502	LOC100289560
				0.90122	GK		0.42205	DPPA4	0.29523	SLC22A4
				0.90186	FLJ43390		0.42235	IQUB	0.29544	C1orf141
				0.90250	MACROD2		0.42265	ZNF625	0.29565	ALLC
				0.90314	ERCC4		0.42295	LOC645146	0.29586	RPS7P5
				0.90378	KBTBD7		0.42325	PMS1	0.29607	WDR90
				0.90443	PEX16		0.42355	TRAF5	0.29628	RTBDN
				0.90507	LSM14B		0.42385	RCSD1	0.29649	TTC16
				0.90571	SLC5A3		0.42415	FAIM3	0.29670	LOC285389
				0.90635	GSTO2		0.42445	SCAND2	0.29691	DSG2
				0.90699	C17orf76		0.42475	CENPL	0.29712	CRISPLD2
				0.90763	PEX11G		0.42505	NUP54	0.29733	ANKRD35
				0.90827	SFRS2B		0.42535	RFPL3S	0.29754	LOC401021
				0.90892	BRWD3		0.42565	FOXK2	0.29775	IL3
				0.90956	AASDHPPT		0.42595	DNAJC12	0.29796	TTC4
				0.91020	IGSF9B		0.42625	DLEU1	0.29817	C14orf70

0.91084	TRIM68	0.42655	KANK3	0.29838	FKSG83
0.91148	ASNA1	0.42685	POLQ	0.29859	MUC19
0.91212	TOP3A	0.42716	DCAF7	0.29880	CLDN3
0.91276	LRRC43	0.42746	DKFZp686L14188	0.29901	SEL1L2
0.91341	DDHD1	0.42776	LOC100130207	0.29922	CCDC12
0.91405	NOB1	0.42806	ACVR2B	0.29943	HCP5
0.91469	RORB	0.42836	RP11-410N8.4	0.29964	PAX3
0.91533	SLC1A6	0.42866	CRISPLD1	0.29985	GFRA3
0.91597	RAB5C	0.42896	LOC100133284	0.30006	WDR45
0.91661	TMEM2	0.42926	LOC440386	0.30027	RAB19
0.91725	PYGB	0.42956	METTL8	0.30048	C11orf85
0.91790	ANKK1	0.42986	SVEP1	0.30069	NR2C2AP
0.91854	LOC729204	0.43016	LILRA1	0.30090	LOC285577
0.91918	TCEAL5	0.43046	ZNF582	0.30111	NYX
0.91982	PSMC5	0.43076	KIAA1407	0.30132	CD19
0.92046	C20orf19	0.43106	FLJ45950	0.30153	C19orf33
0.92110	DPP3	0.43136	C12orf63	0.30174	NLRP7
0.92174	SOX5	0.43166	SERPINB2	0.30195	EEF1G
0.92239	TYRP1	0.43196	FLJ44653	0.30216	ARHGAP27
0.92303	MRP63	0.43226	TAB2	0.30237	RBP5
0.92367	KCMF1	0.43256	KIAA1328	0.30258	TMEM125
0.92431	ACTA2	0.43286	LOC100133991	0.30279	SF3A3
0.92495	RASSF4	0.43316	CDCA2	0.30300	LPIN3
0.92559	TMEM223	0.43346	NCRNA00085	0.30321	MLANA
0.92623	FBXL19	0.43376	VNN2	0.30343	LOC645949
0.92688	LOC100286844	0.43406	WDHD1	0.30364	LOC100289552
0.92752	C12orf64	0.43436	C19orf23	0.30385	MLNR
0.92816	RPRD1B	0.43467	ZNF619	0.30406	ITIH4
0.92880	SPCS2	0.43497	FAM70B	0.30427	SPATA4
0.92944	DCUN1D3	0.43527	AQP3	0.30448	YY2
0.93008	AMFR	0.43557	FAM55B	0.30469	TBC1D21
0.93072	LOC284408	0.43587	HIST1H2AE	0.30490	KCNE1
0.93137	TNFRSF13B	0.43617	LRRC14	0.30511	HCG22
0.93201	FLJ10038	0.43647	MOCS2	0.30532	TMPRSS2
0.93265	NUDT11	0.43677	OR9H1P	0.30553	C17orf56
0.93329	ZNF641	0.43707	MYO5C	0.30574	KLK12
0.93393	CRYBB2P1	0.43737	LOC284219	0.30595	LOC151438
0.93457	CHRDL2	0.43767	FAM175A	0.30616	LOC400499
0.93521	COQ9	0.43797	IFRD1	0.30637	ALOX15
0.93586	COTL1	0.43827	LOC100335030	0.30658	LGALS4
0.93650	C19orf10	0.43857	PLEKHF1	0.30679	GPR109B
0.93714	RBM14	0.43887	TFDP2	0.30700	CDHR4

0.93778	MRPS26	0.43917	ZNF681	0.30721	BEAN
0.93842	HTR7	0.43947	LOC643723	0.30742	SDR42E1
0.93906	TRIM13	0.43977	BTN3A2	0.30763	HCST
0.93970	SLC16A8	0.44007	FLJ39061	0.30784	NUAK2
0.94035	CHMP5	0.44037	SPAG7	0.30805	MXD4
0.94099	MEPE	0.44067	RRP15	0.30826	GKN3P
0.94163	RASD1	0.44097	KRTCAP2	0.30847	KRT8
0.94227	C12orf57	0.44127	FOXE3	0.30868	GLI1
0.94291	SETDB2	0.44157	HSPA1L	0.30889	SLC11A1
0.94355	NTAN1	0.44187	MED18	0.30910	LMX1B
0.94419	ZNF28	0.44217	RRN3P1	0.30931	LOC100134259
0.94484	ZNF544	0.44248	PHLDA3	0.30952	PSMB2
0.94548	ZNF33A	0.44278	RPE65	0.30973	MYO1A
0.94612	SAMD12	0.44308	GHR	0.30994	CD48
0.94676	OPRK1	0.44338	ZNF630	0.31015	LOC283440
0.94740	NEDD8	0.44368	IL13RA2	0.31036	GHRHR
0.94804	TNFAIP8L3	0.44398	OR6C3	0.31057	SLC22A16
0.94869	BAD	0.44428	ZNF669	0.31078	PTTG1IP
0.94933	LOC439914	0.44458	KLK15	0.31099	DZIP1L
0.94997	CCND2	0.44488	PCDHA1	0.31120	GC
0.95061	ZNF440	0.44518	PGGT1B	0.31141	PCSK9
0.95125	LOC100293882	0.44548	ZCCHC8	0.31162	SCNN1D
0.95189	ZNF548	0.44578	DNAH14	0.31183	ILDR1
0.95253	PPIB	0.44608	AMY2B	0.31204	C4orf51
0.95318	ATP8B3	0.44638	CRCP	0.31225	MSR1
0.95382	DDX6	0.44668	ARL5A	0.31246	SCARF2
0.95446	TTC5	0.44698	PUS3	0.31267	WISP3
0.95510	NUDT10	0.44728	ASPRV1	0.31288	LOC100129767
0.95574	DNAJB1	0.44758	CCDC112	0.31309	STAP2
0.95638	LOC157740	0.44788	CENPQ	0.31330	F11
0.95702	GRID1	0.44818	ZNF761	0.31351	UNCX
0.95767	LOC100288205	0.44848	ALG13	0.31372	SLC15A1
0.95831	SOCS2	0.44878	SAAL1	0.31393	TBX5
0.95895	LSMD1	0.44908	VDAC2	0.31414	RPL38
0.95959	ME2	0.44938	SOCS6	0.31435	RUNX3
0.96023	HS3ST2	0.44968	AMBN	0.31456	TMEM101
0.96087	C10orf76	0.44998	ANGPTL1	0.31477	C8orf86
0.96151	LAGE3	0.45029	NDUFB1	0.31498	KANK4
0.96216	ST8SIA6	0.45059	SCGN	0.31519	LOC729897
0.96280	MPPED1	0.45089	PIGC	0.31540	GPR1
0.96344	WIBG	0.45119	FZD6	0.31561	GINS2
0.96408	LOC283454	0.45149	IGDCC4	0.31582	GATA4

0.96472	LOC144481	0.45179	GPRIN3	0.31603	FLJ25715
0.96536	FAM192A	0.45209	PAX6	0.31624	EPS8L2
0.96600	C12orf64	0.45239	PI15	0.31645	SCUBE1
0.96665	DKK3	0.45269	CLIC2	0.31666	CYP17A1
0.96729	C22orf30	0.45299	NUSAP1	0.31687	LOC727941
0.96793	SPTB	0.45329	C11orf70	0.31708	CXorf67
0.96857	UXT	0.45359	COQ2	0.31729	FXYD3
0.96921	METRNL	0.45389	AMN1	0.31750	LOC100129969
0.96985	GVIN1	0.45419	TMEM216	0.31771	OTOP2
0.97049	ACOT4	0.45449	C14orf183	0.31792	SLC16A13
0.97114	MMAB	0.45479	LMAN2L	0.31813	LOC100132813
0.97178	URB1	0.45509	HNRNPC	0.31834	CTU2
0.97242	ZNF805	0.45539	LOC100287841	0.31855	DMBX1
0.97306	GDA	0.45569	CACYBP	0.31876	LOXL4
0.97370	A4GALT	0.45599	CDKL3	0.31897	C9orf128
0.97434	DUSP6	0.45629	VPS37C	0.31918	PIWIL3
0.97498	MPPE1	0.45659	HCG4P6	0.31939	ABO
0.97563	LOC642852	0.45689	PIGT	0.31960	LOC572558
0.97627	KCNK10	0.45719	MPDU1	0.31982	CLEC17A
0.97691	NTN4	0.45749	CPXM2	0.32003	LOC388996
0.97755	ZNF91	0.45780	WDR78	0.32024	OPN5
0.97819	MCF2L	0.45810	ZNF705D	0.32045	LOC100129098
0.97883	FLJ39303	0.45840	RBP1	0.32066	LOC284072
0.97947	KCNK10	0.45870	TTC7A	0.32087	LOC100128556
0.98012	BMS1P4	0.45900	MYD88	0.32108	IL1RL1
0.98076	LOC100127930	0.45930	ZNF814	0.32129	TRIM75
0.98140	RPS27A	0.45960	KRTAP5-7	0.32150	C1QL4
0.98204	LRRC56	0.45990	LOC100131244	0.32171	KCTD19
0.98268	CTSD	0.46020	DDX18	0.32192	SCGB1A1
0.98332	ZNF81	0.46050	ZSCAN12	0.32213	NXNL2
0.98396	LOC100128239	0.46080	FLJ00049	0.32234	PMPCA
0.98461	LOC728192	0.46110	LOC283089	0.32255	AGPAT2
0.98525	FLJ42627	0.46140	HAUS3	0.32276	LOC389120
0.98589	LOC727804	0.46170	SETDB2	0.32297	SDCCAG3
0.98653	NUDT4	0.46200	EFHC2	0.32318	FLG
0.98717	TATDN1	0.46230	PVRIG	0.32339	ARMC5
0.98781	ZNF426	0.46260	RBMS2	0.32360	F2
0.98845	KLF6	0.46290	X2849859	0.32381	FAAH2
0.98910	BRWD3	0.46320	ALS2	0.32402	PIAS4
0.98974	ZNF816A	0.46350	C3orf65	0.32423	CFB
0.99038	SEC61B	0.46380	DCLRE1C	0.32444	LOC729707
0.99102	JMJD6	0.46410	NUP37	0.32465	LOC100132163

0.99166	OVOS	0.46440	LOC644285	0.32486	LOC643802
0.99230	POLD3	0.46470	C5orf54	0.32507	RG9MTD3
0.99294	C16orf55	0.46500	NYNRIN	0.32528	ATP2C2
0.99359	TMEM121	0.46530	LOC100289409	0.32549	LOC400743
0.99423	LRRC55	0.46561	MTRF1L	0.32570	PODNL1
0.99487	CPLX3	0.46591	DEPDC1	0.32591	LOC100287115
0.99551	LOC100288047	0.46621	SDS	0.32612	KIRREL2
0.99615	ZNF737	0.46651	MIR543	0.32633	DMKN
0.99679	ATP6V0A2	0.46681	TUBGCP2	0.32654	TTC31
0.99743	AHCY	0.46711	TTK	0.32675	LOC100131773
0.99808	TBK1	0.46741	RUFY1	0.32696	WEE2
0.99872	POLL	0.46771	RFC3	0.32717	CEACAM20
0.99936	LOC286382	0.46801	LOC100288882	0.32738	FLJ35024
		0.46831	GPR107	0.32759	VAMP8
		0.46861	ZFP28	0.32780	AKR1B15
		0.46891	PCDHB7	0.32801	OR6P1
		0.46921	LRRC9	0.32822	C6orf138
		0.46951	C3orf31	0.32843	TRIM55
		0.46981	MLL	0.32864	APOA5
		0.47011	PHF10	0.32885	LOC100134576
		0.47041	GYPB	0.32906	LHCGR
		0.47071	RILPL1	0.32927	ZNF587
		0.47101	MRPL30	0.32948	COL27A1
		0.47131	DMGDH	0.32969	PPM1N
		0.47161	CENPC1	0.32990	PAK4
		0.47191	HHEX	0.33011	COLQ
		0.47221	LDHA	0.33032	PAX8
		0.47251	BCAR3	0.33053	CCDC42B
		0.47281	NUDT13	0.33074	CA9
		0.47312	RAG1AP1	0.33095	UPK3B
		0.47342	ZNF264	0.33116	C14orf182
		0.47372	REEP4	0.33137	AWAT2
		0.47402	CCDC15	0.33158	ACSM1
		0.47432	HOXA7	0.33179	ARHGAP30
		0.47462	TBC1D10A	0.33200	CRB3
		0.47492	INPP5B	0.33221	LOC643008
		0.47522	PEX6	0.33242	FAM123B
		0.47552	OLFML1	0.33263	SNRPC
		0.47582	LOC728339	0.33284	THAP6
		0.47612	CCDC46	0.33305	TMEM134
		0.47642	BET1	0.33326	DISC2
		0.47672	CXorf57	0.33347	LOC100133116

0.47702	SETD6	0.33368	RNF186
0.47732	RNF217	0.33389	C20orf123
0.47762	DBR1	0.33410	C8orf83
0.47792	COL19A1	0.33431	TMEM201
0.47822	CCT6B	0.33452	PTCH2
0.47852	FBXO4	0.33473	ISX
0.47882	C10orf134	0.33494	CYP4F8
0.47912	CERKL	0.33515	WNT1
0.47942	PTGER3	0.33536	SP8
0.47972	LOC284561	0.33557	FLJ26245
0.48002	VKORC1L1	0.33578	ZIC4
0.48032	STXBP4	0.33599	C15orf50
0.48062	PYGL	0.33621	FLJ23152
0.48093	FLJ31485	0.33642	FBXL15
0.48123	LYPD6	0.33663	TNFRSF4
0.48153	RUNX1T1	0.33684	HCFC1R1
0.48183	ZNF564	0.33705	IBSP
0.48213	PTPLA	0.33726	PBX4
0.48243	ZNF200	0.33747	FLJ46365
0.48273	FASTKD3	0.33768	GPR111
0.48303	SNORD16	0.33789	C8orf80
0.48333	KIAA1009	0.33810	IRF6
0.48363	LOC100128842	0.33831	TRPA1
0.48393	LRP2BP	0.33852	USP51
0.48423	SLC25A43	0.33873	AHSG
0.48453	SVEP1	0.33894	C9orf27
0.48483	EPOR	0.33915	CGN
0.48513	SNHG12	0.33936	REG1P
0.48543	C10orf12	0.33957	KNCN
0.48573	TIGD7	0.33978	tcag7.935
0.48603	RXFP2	0.33999	IL2RG
0.48633	ATP10D	0.34020	SLC22A12
0.48663	C3orf15	0.34041	HK2
0.48693	GGCX	0.34062	FAM71E2
0.48723	C18orf16	0.34083	CCDC105
0.48753	ARHGEF33	0.34104	INHA
0.48783	C1orf132	0.34125	RHBDD3
0.48813	WHAMM	0.34146	LOC730079
0.48843	DNAH14	0.34167	EVX1
0.48874	KCNRG	0.34188	LOC158376
0.48904	LOC100131551	0.34209	TAAR3
0.48934	FOXL1	0.34230	C11orf52

						0.48964	AP1S2	0.34251	MAGEC3
						0.48994	LOC100129773	0.34272	ACSM5
						0.49024	VGLL3	0.34293	ADAMTS12
						0.49054	RAB12	0.34314	LOC441005
						0.49084	SCLY	0.34335	VEGFC
						0.49114	LYZ	0.34356	GBGT1
						0.49144	VSIG10	0.34377	ADCY7
						0.49174	MVD	0.34398	CELSR1
						0.49204	SLC35E4	0.34419	CAPN12
						0.49234	DYX1C1	0.34440	ITGA5
						0.49264	PAFAH2	0.34461	SPACA1
						0.49294	CCDC109B	0.34482	KIF7
						0.49324	CAPN6	0.34503	CLSPN
						0.49354	CLEC2B	0.34524	C1orf126
						0.49384	HEATR3	0.34545	TP53TG5
						0.49414	TMEM87B	0.34566	LOC100133991
						0.49444	MREG	0.34587	SCGB2A1
						0.49474	DNAH6	0.34608	MGC16121
						0.49504	SDHD	0.34629	SKINTL
						0.49534	ZNF835	0.34650	GCNT7
						0.49564	VN1R5	0.34671	RFNG
						0.49594	SERPINE1	0.34692	NBEAL2
						0.49625	C2orf63	0.34713	CMKLR1
						0.49655	RAC1	0.34734	KIAA1210
						0.49685	LOC100101938	0.34755	SBNO2
						0.49715	DKFZP564C196	0.34776	GPR139
						0.49745	FAM184B	0.34797	CAPN10
						0.49775	TRPT1	0.34818	SCGBL
						0.49805	OMD	0.34839	SEMA3F
						0.49835	C11orf58	0.34860	LOC100289095
						0.49865	RNF181	0.34881	SMPDL3B
						0.49895	ZNF486	0.34902	C8orf71
						0.49925	LOC644538	0.34923	FCAR
						0.49955	SAP30	0.34944	LOC100130954
						0.49985	C6orf170	0.34965	IGHV7-81
						0.50015	PUS10	0.34986	CDK3
						0.50045	MDGA1	0.35007	DSCR6
						0.50075	SEC61A1	0.35028	COL6A4P2
						0.50105	MRPS28	0.35049	LOC441239
						0.50135	C14orf147	0.35070	HYI
						0.50165	F2RL2	0.35091	CIDEC
						0.50195	ENDOG	0.35112	TRBV30

0.50225	LOC730005	0.35133	ASB10
0.50255	FAM21A	0.35154	LHX1
0.50285	ABCB10	0.35175	MSX1
0.50315	LOC284669	0.35196	PCSK4
0.50345	EIF2B5	0.35217	LOC399744
0.50375	CLLU1	0.35238	SLC16A3
0.50406	LOC100291644	0.35260	EMR1
0.50436	PDLIM1	0.35281	LOC651337
0.50466	LOC100302650	0.35302	PTCHD3
0.50496	C6orf1	0.35323	TMPRSS6
0.50526	MANBA	0.35344	GYPC
0.50556	REXO4	0.35365	C6orf218
0.50586	C9orf95	0.35386	FLJ41350
0.50616	LOC339505	0.35407	IGFBP6
0.50646	NDST4	0.35428	LOC389333
0.50676	DUSP28	0.35449	TINAG
0.50706	LYPLA1	0.35470	LOC441177
0.50736	BIN2	0.35491	HAP1
0.50766	ZNF672	0.35512	ANKRD30A
0.50796	TMEM84	0.35533	COL9A1
0.50826	LOC100130000	0.35554	FLJ42291
0.50856	ABHD3	0.35575	DNAJC5B
0.50886	TMEM67	0.35596	PDZD3
0.50916	GTF3A	0.35617	BFSP2
0.50946	ADAMTS5	0.35638	LOC100292080
0.50976	KLHL23	0.35659	STK10
0.51006	HPSE2	0.35680	hCG_1651160
0.51036	ZNF732	0.35701	ALPK3
0.51066	EPPK1	0.35722	LOC729224
0.51096	ZNF606	0.35743	LOC642929
0.51126	CRYBB2P1	0.35764	SCUBE2
0.51157	XPO4	0.35785	CDHR5
0.51187	ECHDC2	0.35806	TUBA4B
0.51217	ANKRD7	0.35827	C22orf23
0.51247	KPTN	0.35848	FLJ32255
0.51277	CCNO	0.35869	TMEM61
0.51307	RGR	0.35890	SMCR7
0.51337	CCDC39	0.35911	GPC2
0.51367	LOC150568	0.35932	MIIP
0.51397	APOC2	0.35953	LCE3A
0.51427	GMPPA	0.35974	C11orf35
0.51457	CLCN5	0.35995	HAND2

0.51487	ABCG5	0.36016	PLAUR
0.51517	LOC100131507	0.36037	FLJ37543
0.51547	LOC642384	0.36058	LOC100288738
0.51577	LOC100291206	0.36079	LOC100129427
0.51607	ATAD2B	0.36100	MIR320A
0.51637	CD226	0.36121	LOC644397
0.51667	ATAD2B	0.36142	CNPY4
0.51697	INSIG2	0.36163	PRODH2
0.51727	ZNF714	0.36184	CCDC121
0.51757	FLJ37396	0.36205	GPR150
0.51787	C9orf46	0.36226	RAB17
0.51817	CCDC66	0.36247	RCN1
0.51847	IKBKG	0.36268	LOC100133050
0.51877	FAM70A	0.36289	CBFA2T3
0.51907	PTTG1	0.36310	LOC100129463
0.51938	EHF	0.36331	NACAP1
0.51968	CXCR4	0.36352	LIG1
0.51998	LOC399815	0.36373	LOC100130135
0.52028	CYP19A1	0.36394	LRRC45
0.52058	CSGALNACT1	0.36415	AMIGO3
0.52088	EGFL7	0.36436	PRG3
0.52118	MOP-1	0.36457	DSP
0.52148	FAM182B	0.36478	ODF4
0.52178	C8orf78	0.36499	MS4A8B
0.52208	FAM82A1	0.36520	PIWIL2
0.52238	ITGB3BP	0.36541	LOC100287852
0.52268	ADAM19	0.36562	CDH15
0.52298	NUDT15	0.36583	LOC100288802
0.52328	SOX30	0.36604	FSHR
0.52358	MRPL22	0.36625	HEYL
0.52388	CCDC126	0.36646	LOC283711
0.52418	CYP51A1	0.36667	MMP28
0.52448	LOC100131929	0.36688	C16orf79
0.52478	TRIM14	0.36709	ESRRB
0.52508	TMX1	0.36730	MALL
0.52538	C5orf34	0.36751	MPG
0.52568	NTS	0.36772	LOC641515
0.52598	F5	0.36793	ACCN3
0.52628	MIF4GD	0.36814	LOC728316
0.52658	FBXO8	0.36835	LOC400696
0.52688	LNP1	0.36856	TSPAN9
0.52719	DNAJB6	0.36877	LOC253842

0.52749	E2F5	0.36899	EIF4H
0.52779	HIST1H2AK	0.36920	FLG2
0.52809	ACTR5	0.36941	LOC727710
0.52839	TES	0.36962	LAG3
0.52869	STARD9	0.36983	IL1R1
0.52899	LOC284373	0.37004	RPL18
0.52929	TRIM71	0.37025	PACS2
0.52959	CMAH	0.37046	LOC100288839
0.52989	PHF13	0.37067	RP1
0.53019	TEX9	0.37088	ESRP2
0.53049	SLC39A8	0.37109	PRDM15
0.53079	GRM6	0.37130	FAM151A
0.53109	CCNB1	0.37151	LOC643677
0.53139	RMND5B	0.37172	ESPL1
0.53169	THAP6	0.37193	LOC100288093
0.53199	ACPP	0.37214	LOC100132014
0.53229	ANKRD30B	0.37235	GZMH
0.53259	WDR67	0.37256	BNIPL
0.53289	QDPR	0.37277	ZNF600
0.53319	LOC100129387	0.37298	C16orf59
0.53349	ABCD4	0.37319	SLC46A2
0.53379	ZNF20	0.37340	NUF2
0.53409	C3orf25	0.37361	LOC100287035
0.53439	GALNTL6	0.37382	LOC158434
0.53470	BOD1P	0.37403	FGF10
0.53500	LUC7L3	0.37424	TMEM149
0.53530	C5orf49	0.37445	KCNK5
0.53560	KIAA0922	0.37466	PLEKHG7
0.53590	ZNF121	0.37487	UROC1
0.53620	ARHGAP19	0.37508	COL8A2
0.53650	ZNF320	0.37529	ITLN1
0.53680	IGSF10	0.37550	SELP
0.53710	ZNF442	0.37571	LAMC3
0.53740	HDHD3	0.37592	LRRC52
0.53770	CRY1	0.37613	C14orf50
0.53800	ARL13B	0.37634	CASZ1
0.53830	ZNF485	0.37655	CCNYL2
0.53860	AGER	0.37676	LOC100128588
0.53890	BET1	0.37697	C1orf118
0.53920	ZDHHC4	0.37718	LOC100128760
0.53950	VEGFA	0.37739	LIPF
0.53980	C1orf145	0.37760	TREML2

0.54010	ARHGAP18	0.37781	C16orf92
0.54040	LOC729143	0.37802	GPR172B
0.54070	SNORD73A	0.37823	LOC390298
0.54100	ZNF506	0.37844	C17orf57
0.54130	ATP9B	0.37865	ASTE1
0.54160	LOC100287468	0.37886	CEACAM21
0.54190	CARD18	0.37907	GRHL2
0.54220	LRRC50	0.37928	LOC100130924
0.54251	IRAK3	0.37949	GAPDHS
0.54281	LY96	0.37970	WDR72
0.54311	CCR7	0.37991	THEG
0.54341	N4BP2L1	0.38012	RPL31P11
0.54371	LOC729350	0.38033	FLJ27351
0.54401	ZNF738	0.38054	UNC45B
0.54431	C1orf194	0.38075	LOC642278
0.54461	ATP6AP1L	0.38096	PKLR
0.54491	DTX3L	0.38117	DOK7
0.54521	C2orf84	0.38138	LOC100293962
0.54551	DNPEP	0.38159	ALG6
0.54581	C8orf50	0.38180	HHAT
0.54611	ANKRD2	0.38201	GLTSCR2
0.54641	ZNF167	0.38222	SLC12A1
0.54671	TTC26	0.38243	EMR4P
0.54701	WDR66	0.38264	C20orf70
0.54731	SVEP1	0.38285	SPRR4
0.54761	ZNF616	0.38306	GSDMD
0.54791	CD2AP	0.38327	INHBA
0.54821	HSD17B7	0.38348	KRTAP10-10
0.54851	C16orf88	0.38369	KLK1
0.54881	ZFP112	0.38390	SERPINA6
0.54911	MTCP1	0.38411	COLEC10
0.54941	LOC646241	0.38432	LOC100287659
0.54971	CD69	0.38453	ARSD
0.55002	TTF2	0.38474	CLIC1
0.55032	NHEDC1	0.38495	DNAH11
0.55062	SEMA4B	0.38516	ELF5
0.55092	SVEP1	0.38538	AQP7
0.55122	CA13	0.38559	FEM1A
0.55152	MOV10	0.38580	ZNF268
0.55182	ATXN7L1	0.38601	IL12B
0.55212	GDPD3	0.38622	CD3D
0.55242	ATOH7	0.38643	PEAR1

						0.55272	CPO	0.38664	RAB44
						0.55302	TNFAIP6	0.38685	LOC651900
						0.55332	TMEM196	0.38706	SUN3
						0.55362	SP3P	0.38727	MICB
						0.55392	COQ7	0.38748	STAG3
						0.55422	C15orf44	0.38769	LOC645561
						0.55452	DNMBP	0.38790	ABCA7
						0.55482	TTC6	0.38811	CSRNP1
						0.55512	MCF2L	0.38832	EHBP1L1
						0.55542	BMP8A	0.38853	LOC100130502
						0.55572	KIAA0509	0.38874	SLC22A6
						0.55602	HYLS1	0.38895	LOC728597
						0.55632	C19orf50	0.38916	ZFHX2
						0.55662	RNPEPL1	0.38937	CCDC17
						0.55692	CDNF	0.38958	HMCN2
						0.55722	AZI2	0.38979	CEMP1
						0.55752	LOC100288183	0.39000	CHIT1
						0.55783	TRPC4	0.39021	WNK4
						0.55813	GALNT3	0.39042	C12orf72
						0.55843	GCKR	0.39063	STX10
						0.55873	ARL9	0.39084	KCTD14
						0.55903	TRIM69	0.39105	NCR2
						0.55933	AASDH	0.39126	RAET1G
						0.55963	IL6R	0.39147	TSPAN8
						0.55993	LOC440122	0.39168	LOC729175
						0.56023	MEGF8	0.39189	KRTAP5-1
						0.56053	C8orf31	0.39210	LOC342346
						0.56083	PDDC1	0.39231	MEOX2
						0.56113	MED9	0.39252	EN2
						0.56143	UBTD2	0.39273	FAM63A
						0.56173	C7orf54	0.39294	PLA2G2F
						0.56203	AGBL1	0.39315	GPR133
						0.56233	KLHL9	0.39336	TRIM61
						0.56263	PRKD2	0.39357	APRT
						0.56293	C20orf74	0.39378	AKAP3
						0.56323	ANKHD1	0.39399	LOC149837
						0.56353	ANGPTL3	0.39420	CEACAM8
						0.56383	ADAM12	0.39441	LOC100130817
						0.56413	LOC100130855	0.39462	AGR2
						0.56443	OFD1	0.39483	LOC400685
						0.56473	NHLRC2	0.39504	DDX39
						0.56503	FAM76B	0.39525	FNDC7

0.56533	BHLHE22	0.39546	C6orf126
0.56564	CBWD2	0.39567	CFD
0.56594	LOC100131510	0.39588	AOX2P
0.56624	GIPC1	0.39609	MYLK4
0.56654	ARHGAP11A	0.39630	GPR97
0.56684	SFMBT1	0.39651	KLHL22
0.56714	OR1F1	0.39672	LOC400499
0.56744	LOC100133991	0.39693	TKTL1
0.56774	PPP1R14C	0.39714	ZNF613
0.56804	C2orf34	0.39735	SLC30A2
0.56834	STEAP3	0.39756	NRG2
0.56864	THG1L	0.39777	TM4SF20
0.56894	LOC286071	0.39798	C7orf53
0.56924	KIAA0040	0.39819	LOC400804
0.56954	NUDT19	0.39840	PLEKHN1
0.56984	FLJ45964	0.39861	C10orf108
0.57014	NXN	0.39882	DMP1
0.57044	LOC729570	0.39903	C2orf71
0.57074	C3orf50	0.39924	KCNJ15
0.57104	GPR19	0.39945	ADAM21
0.57134	POLM	0.39966	PDE6G
0.57164	LOC100131530	0.39987	BDKRB1
0.57194	DHDDS	0.40008	OR10S1
0.57224	SLC35F2	0.40029	BIN3
0.57254	EYS	0.40050	OR2K2
0.57284	BNIP1	0.40071	KLK2
0.57315	FAM91A1	0.40092	PDZD7
0.57345	PLA2R1	0.40113	LOC100130419
0.57375	PROS1	0.40134	WDR87
0.57405	PI4KAP1	0.40155	TMEM200C
0.57435	RPIA	0.40177	NOD2
0.57465	DEPDC1B	0.40198	ADCK5
0.57495	ATP8B1	0.40219	OR10A3
0.57525	ZNF273	0.40240	LOC284542
0.57555	HMGN3	0.40261	RBM47
0.57585	SH3GL1	0.40282	NKX1-1
0.57615	GPR39	0.40303	LRTM1
0.57645	MKRN1	0.40324	FAM169B
0.57675	ZAK	0.40345	C10orf31
0.57705	FLJ23865	0.40366	GGT8P
0.57735	PEX2	0.40387	VASP
0.57765	PCDH15	0.40408	TRIM42

0.57795	ERCC6	0.40429	HNRPA1P5
0.57825	ITGAE	0.40450	RSPO1
0.57855	C2orf66	0.40471	FAM133A
0.57885	C5orf39	0.40492	AVIL
0.57915	APITD1	0.40513	HSF2BP
0.57945	LOC100130691	0.40534	GIF
0.57975	FAM190A	0.40555	ZNF385C
0.58005	BRWD3	0.40576	LOC283663
0.58035	FAM35A	0.40597	PLAC1
0.58065	PLS1	0.40618	GALNTL5
0.58096	SCML1	0.40639	KIFC1
0.58126	EML5	0.40660	TM6SF2
0.58156	CUTA	0.40681	SLC38A8
0.58186	EIF3B	0.40702	FNDC8
0.58216	CRLF3	0.40723	ZNF311
0.58246	LOC344065	0.40744	IL1R2
0.58276	ZNF674	0.40765	DCAF8L1
0.58306	TMEM159	0.40786	PFN4
0.58336	UPK1B	0.40807	FLJ31813
0.58366	ELMOD3	0.40828	MMP7
0.58396	ZNF836	0.40849	OR9G4
0.58426	TRNAU1AP	0.40870	PRX
0.58456	XPC	0.40891	PRAMEF12
0.58486	DOCK4	0.40912	WNT2
0.58516	RAB30	0.40933	SPAG8
0.58546	ZNF468	0.40954	LOC100130172
0.58576	APH1B	0.40975	NCRNA00115
0.58606	EPHX2	0.40996	MYO7B
0.58636	ST6GALNAC2	0.41017	CNKSR1
0.58666	TFPI	0.41038	WDFY4
0.58696	FLJ39061	0.41059	LIMD2
0.58726	NCRNA00185	0.41080	SIX3
0.58756	LOC388559	0.41101	LOC401180
0.58786	SVEP1	0.41122	FAM65C
0.58816	RRP7B	0.41143	LOC283856
0.58847	ZNF45	0.41164	CLP1
0.58877	CYP4F3	0.41185	OR1E1
0.58907	TIMP4	0.41206	RPL34
0.58937	NUTF2	0.41227	ZNF560
0.58967	RGS1	0.41248	MAP2K3
0.58997	LOC100289332	0.41269	IGF2BP2
0.59027	DDX26B	0.41290	C19orf57

						0.59057	AQP9	0.41311	DNAI1
						0.59087	ELMOD2	0.41332	RREB1
						0.59117	SLC22A5	0.41353	MPZL3
						0.59147	KCNMB1	0.41374	NCF2
						0.59177	WDR69	0.41395	LOC100128751
						0.59207	LOC388692	0.41416	ITGB1BP3
						0.59237	TNFRSF11B	0.41437	AARSD1
						0.59267	ZNF354B	0.41458	C11orf75
						0.59297	DAB1	0.41479	ARHGAP40
						0.59327	NHLRC1	0.41500	ZNF296
						0.59357	INADL	0.41521	TM4SF4
						0.59387	C17orf74	0.41542	TACR2
						0.59417	FANCE	0.41563	CCL15
						0.59447	ZSCAN23	0.41584	SLC6A3
						0.59477	LOC390940	0.41605	ZNF683
						0.59507	SF3B4	0.41626	LOC100128788
						0.59537	RAB33A	0.41647	ZNF19
						0.59567	ZNF208	0.41668	ELL3
						0.59597	RPL31	0.41689	KRTAP5-1
						0.59628	ERN1	0.41710	RNF216L
						0.59658	UBXN8	0.41731	MAGEA10
						0.59688	DNAH7	0.41752	OIT3
						0.59718	LOC149832	0.41773	C8orf47
						0.59748	C21orf130	0.41794	FGF18
						0.59778	DOT1L	0.41816	S100P
						0.59808	SSBP1	0.41837	HOXD3
						0.59838	LOC100289292	0.41858	LOC643201
						0.59868	C15orf28	0.41879	FAM129C
						0.59898	SEPX1	0.41900	TPH1
						0.59928	RFESD	0.41921	FER1L6
						0.59958	DNAJB14	0.41942	FAM181B
						0.59988	PHRF1	0.41963	MUC19
						0.60018	PGM2	0.41984	KRTDAP
						0.60048	PLAGL2	0.42005	OR2J3
						0.60078	FAM45A	0.42026	BTF3L1
						0.60108	TEKT1	0.42047	FDXR
						0.60138	DDB2	0.42068	REG1A
						0.60168	TDRD9	0.42089	WDR90
						0.60198	AKD1	0.42110	PROCA1
						0.60228	YWHAE	0.42131	LOC339316
						0.60258	FLJ16734	0.42152	KIAA0415
						0.60288	DCAF8	0.42173	FAM125A

0.60318	DLX1	0.42194	AEN
0.60348	ARL2	0.42215	CD52
0.60378	HNRNPA1L2	0.42236	TBX20
0.60409	CLIC5	0.42257	C1orf49
0.60439	CTBP2	0.42278	LOC100287412
0.60469	DAO	0.42299	NDOR1
0.60499	ZNF710	0.42320	VNN1
0.60529	BPA-1	0.42341	TSSK4
0.60559	GIMAP7	0.42362	NUP50
0.60589	RNF139	0.42383	LOC152586
0.60619	EPCAM	0.42404	CD247
0.60649	PSG6	0.42425	PTPN7
0.60679	EIF4A2	0.42446	MYADML
0.60709	TCEAL7	0.42467	OR13F1
0.60739	PRSS12	0.42488	ALDH3B2
0.60769	INTS3	0.42509	TRABD
0.60799	CLEC14A	0.42530	LOC285857
0.60829	EPB41L4A	0.42551	CATSPER3
0.60859	CCDC79	0.42572	PADI3
0.60889	ADH5	0.42593	EDN3
0.60919	ABCA6	0.42614	KPNA7
0.60949	TPX2	0.42635	OR10P1
0.60979	AMMECR1	0.42656	LOC285286
0.61009	CTDP1	0.42677	C1orf86
0.61039	ZNF227	0.42698	LOC100128683
0.61069	CMTM6	0.42719	OR1F2P
0.61099	ZNF529	0.42740	SLC9A4
0.61129	ZNF471	0.42761	LOC285889
0.61160	GCDH	0.42782	COL14A1
0.61190	ASB4	0.42803	UBE2F
0.61220	PCDH18	0.42824	ALDH1A2
0.61250	SFRS12IP1	0.42845	GOLGA2B
0.61280	CCDC142	0.42866	RDH8
0.61310	CAV2	0.42887	IGF2
0.61340	GCH1	0.42908	CCDC81
0.61370	ZNF264	0.42929	C1orf97
0.61400	EMP1	0.42950	AKNA
0.61430	AGXT2L2	0.42971	RESP18
0.61460	FAM89A	0.42992	FAM91A1
0.61490	PLEKHH2	0.43013	C10orf27
0.61520	SLC35C2	0.43034	ZFAND2B
0.61550	HES1	0.43055	SCNN1B

						0.61580	MME	0.43076	MPZL2
						0.61610	SNORA31	0.43097	NYNRIN
						0.61640	C1GALT1C1	0.43118	NANOS3
						0.61670	TAF5	0.43139	NXF3
						0.61700	IFI44L	0.43160	ANO9
						0.61730	RGS17	0.43181	FLJ42392
						0.61760	ZNF616	0.43202	CCDC24
						0.61790	LDHB	0.43223	RXFP3
						0.61820	TMPRSS11B	0.43244	EFR3B
						0.61850	BCL2A1	0.43265	KIRREL
						0.61880	GRB14	0.43286	C6orf132
						0.61910	EDNRA	0.43307	SOAT2
						0.61941	TSPAN2	0.43328	SLC5A8
						0.61971	FLJ45340	0.43349	NODAL
						0.62001	FTSJ1	0.43370	C14orf166B
						0.62031	SLC27A3	0.43391	KRT5
						0.62061	DNM1P35	0.43412	PSTPIP1
						0.62091	LOC387646	0.43433	ALPK2
						0.62121	C5orf28	0.43455	TPRG1
						0.62151	CFLP1	0.43476	RAB12
						0.62181	ZNF622	0.43497	CYB561D1
						0.62211	TMEM173	0.43518	DCDC2B
						0.62241	INO80B	0.43539	C1orf81
						0.62271	ATP6V1G3	0.43560	TRIM45
						0.62301	ZNF506	0.43581	SSPO
						0.62331	LYVE1	0.43602	EPPK1
						0.62361	FAM36A	0.43623	STC1
						0.62391	CNOT8	0.43644	GPR61
						0.62421	GALNTL6	0.43665	ZFP42
						0.62451	C15orf40	0.43686	CITED1
						0.62481	PLCH2	0.43707	PRSS22
						0.62511	ZNF438	0.43728	UAP1L1
						0.62541	SERPINB6	0.43749	ARHGAP25
						0.62571	ABI3	0.43770	PLA2G4D
						0.62601	HRH1	0.43791	XKR5
						0.62631	CCPG1	0.43812	COX6B2
						0.62661	GPR101	0.43833	RADIL
						0.62691	LOC100128340	0.43854	COL18A1
						0.62722	PRDM1	0.43875	C17orf66
						0.62752	MATN3	0.43896	JDP2
						0.62782	C5orf25	0.43917	NCRNA00083
						0.62812	NKAPL	0.43938	FLJ39582

						0.62842	MRPL43	0.43959	L3MBTL2
						0.62872	LOC728537	0.43980	TREML1
						0.62902	CXCL3	0.44001	LOC100133985
						0.62932	SEC23IP	0.44022	FLJ38723
						0.62962	C6orf72	0.44043	IGF2BP3
						0.62992	C8orf84	0.44064	TTC23L
						0.63022	LOC100132356	0.44085	ZNF839
						0.63052	LOC100292236	0.44106	FAM83H
						0.63082	C10orf79	0.44127	TMEM184A
						0.63112	UPP1	0.44148	MLL
						0.63142	PRMT6	0.44169	ADH1B
						0.63172	MKS1	0.44190	C3orf32
						0.63202	FHIT	0.44211	AFG3L1
						0.63232	TTC32	0.44232	SELE
						0.63262	LETM1	0.44253	KCNJ12
						0.63292	LOC284408	0.44274	RPS5
						0.63322	TRIM4	0.44295	CCNA2
						0.63352	RPS6KA6	0.44316	DHRS1
						0.63382	CHMP4B	0.44337	GPBAR1
						0.63412	HMGB2	0.44358	CD40
						0.63442	LOC100291668	0.44379	SLC6A16
						0.63473	NCRNA00171	0.44400	ZAR1L
						0.63503	ZAP70	0.44421	DMRTC2
						0.63533	ARHGAP6	0.44442	FLJ46257
						0.63563	WDR77	0.44463	CYP2D7P1
						0.63593	WDR61	0.44484	PARP10
						0.63623	TMEM116	0.44505	MSX2P1
						0.63653	TOR3A	0.44526	BMP5
						0.63683	LYRM7	0.44547	LOC154822
						0.63713	CGRRF1	0.44568	GHDC
						0.63743	TMEM41A	0.44589	NEIL2
						0.63773	ZNF749	0.44610	OR52L1
						0.63803	CPN1	0.44631	SLMO1
						0.63833	NPHP1	0.44652	FAM19A3
						0.63863	SLC46A3	0.44673	SLC34A1
						0.63893	FBP1	0.44694	IL15RA
						0.63923	IGF2	0.44715	C10orf114
						0.63953	TEX15	0.44736	LOC100128098
						0.63983	LOC100129858	0.44757	EGFL6
						0.64013	LOC100287731	0.44778	LOC100287301
						0.64043	DKFZP686I15217	0.44799	RELB
						0.64073	FAM175A	0.44820	ONECUT1

0.64103	TRMT5	0.44841	SLC38A10
0.64133	GAPDH	0.44862	ITPKC
0.64163	SLC24A6	0.44883	LOC100129126
0.64193	ZKSCAN5	0.44904	MYH8
0.64223	FKSG29	0.44925	C11orf76
0.64254	MRPS18A	0.44946	SIAH1
0.64284	PDIA5	0.44967	CA3
0.64314	SLFN11	0.44988	LAIR1
0.64344	GAS2L3	0.45009	FMO3
0.64374	ZNF550	0.45030	GPR15
0.64404	TTC9C	0.45051	CCL1
0.64434	SLCO2A1	0.45072	SUV420H2
0.64464	MAN1B1	0.45094	KRT38
0.64494	C3orf34	0.45115	RTP4
0.64524	CRIPAK	0.45136	CPAMD8
0.64554	IRAK1	0.45157	KCNMB3
0.64584	GRK7	0.45178	FOXP3
0.64614	C10orf76	0.45199	PLK3
0.64644	KIAA1715	0.45220	SQRDL
0.64674	MAP1LC3B	0.45241	ROPN1B
0.64704	C11orf17	0.45262	PRDM12
0.64734	TNFSF13B	0.45283	OBSCN
0.64764	UBXN2A	0.45304	C15orf39
0.64794	C14orf159	0.45325	LOC147791
0.64824	CBWD5	0.45346	DKFZp761P0212
0.64854	PLAC8L1	0.45367	POLD4
0.64884	NDUFB11	0.45388	SLC4A11
0.64914	KNG1	0.45409	MGC3771
0.64944	ZNF469	0.45430	FCRL1
0.64974	FLJ40453	0.45451	MSMP
0.65005	NPR2	0.45472	C3orf56
0.65035	SIGIRR	0.45493	C1orf110
0.65065	GLRX5	0.45514	LOC100129498
0.65095	SNTG2	0.45535	CRIP3
0.65125	POU2F1	0.45556	ZNF69
0.65155	IKZF4	0.45577	X3051800
0.65185	GLA	0.45598	PRDM7
0.65215	C3orf55	0.45619	NCRNA00176
0.65245	TMCO7	0.45640	LOC100287866
0.65275	GJB2	0.45661	ZNF367
0.65305	TCEA1	0.45682	ITIH1
0.65335	CHRNA7	0.45703	AHRR

0.65365	C9orf68	0.45724	OR51B6
0.65395	MSX2	0.45745	PRPH
0.65425	AKR1C1	0.45766	FCRL5
0.65455	LOC387876	0.45787	IQCC
0.65485	LOC100291104	0.45808	PRKCZ
0.65515	SLC25A30	0.45829	ZNF474
0.65545	DDX28	0.45850	ANXA10
0.65575	SLC5A3	0.45871	UNQ6975
0.65605	SP5	0.45892	TFAP2E
0.65635	LOC283588	0.45913	CLLU1OS
0.65665	POLA2	0.45934	FGF17
0.65695	FAM105B	0.45955	UBA7
0.65725	CEBPA	0.45976	LOC284757
0.65755	SLC18A2	0.45997	PLCG2
0.65786	KLRC1	0.46018	C2orf62
0.65816	BCL10	0.46039	SEMG1
0.65846	FAM55C	0.46060	CD8A
0.65876	SIVA1	0.46081	CDCA4
0.65906	HIF3A	0.46102	PTCD1
0.65936	RGS12	0.46123	PKIB
0.65966	GEN1	0.46144	CENPK
0.65996	CCDC158	0.46165	CA5A
0.66026	RNFT1	0.46186	CYP3A7
0.66056	LOC284998	0.46207	LOC220980
0.66086	LOC100292748	0.46228	AAA1
0.66116	SLFN12L	0.46249	CRISP2
0.66146	RPL10	0.46270	KCNG1
0.66176	SCFD1	0.46291	KCNK13
0.66206	LOC283922	0.46312	CYAT1
0.66236	ZBTB8A	0.46333	METTL11A
0.66266	C13orf27	0.46354	GLYATL1
0.66296	DHX33	0.46375	RHBDF2
0.66326	PRKCDBP	0.46396	C20orf160
0.66356	TBX1	0.46417	ASS1
0.66386	LOC642891	0.46438	DRGX
0.66416	ST7OT2	0.46459	LSR
0.66446	VPS24	0.46480	NTN3
0.66476	PCGF6	0.46501	LOC440742
0.66506	ATP5E	0.46522	TMEM27
0.66536	CCDC59	0.46543	TRIM21
0.66567	TDRD5	0.46564	RBM46
0.66597	C10orf107	0.46585	LOC284475

						0.66627	CCDC30	0.46606	P2RX1
						0.66657	LUM	0.46627	CCKAR
						0.66687	COL4A1	0.46648	GLOD4
						0.66717	MTMR14	0.46669	CCDC102B
						0.66747	LOC644285	0.46690	LOC388387
						0.66777	SLC6A4	0.46711	HEXIM2
						0.66807	HEXDC	0.46733	EMR3
						0.66837	C2orf15	0.46754	SSPO
						0.66867	C3orf31	0.46775	SSPO
						0.66897	PRIM1	0.46796	KRTAP5-2
						0.66927	SLC2A8	0.46817	DAD1
						0.66957	UTRN	0.46838	LOC100129931
						0.66987	TIMM9	0.46859	LOC645591
						0.67017	UBB	0.46880	LOC100288376
						0.67047	TMEM20	0.46901	LOC285593
						0.67077	TRIM52	0.46922	C1orf113
						0.67107	ZBTB26	0.46943	IL24
						0.67137	KY	0.46964	LOC100129626
						0.67167	LOC100132099	0.46985	CAPG
						0.67197	KLRD1	0.47006	LOC100130480
						0.67227	DAB2	0.47027	LOC100133893
						0.67257	STARD7	0.47048	CRYBB3
						0.67287	FLJ43944	0.47069	BTLA
						0.67318	LOC100132167	0.47090	GTSF1
						0.67348	PLSCR3	0.47111	DEFB129
						0.67378	GABRA6	0.47132	EPS8L3
						0.67408	LAMA4	0.47153	C17orf58
						0.67438	FTSJ3	0.47174	CR1
						0.67468	MGC87042	0.47195	CD1D
						0.67498	ZNF193	0.47216	TGOLN2
						0.67528	SYS1	0.47237	DKFZp434F142
						0.67558	C10orf67	0.47258	TMC3
						0.67588	EXD1	0.47279	AXIN2
						0.67618	VSIG1	0.47300	LOC100133180
						0.67648	ZNF799	0.47321	LOC100288083
						0.67678	EYS	0.47342	PRAM1
						0.67708	ZNF785	0.47363	TRIP10
						0.67738	CRTC2	0.47384	CASP14
						0.67768	C6orf165	0.47405	C1QTNF5
						0.67798	ACVR2B	0.47426	ANKS3
						0.67828	SNX7	0.47447	PABPC1L
						0.67858	KCNQ1OT1	0.47468	C4orf52

0.67888	ZNF616	0.47489	SEC16A
0.67918	ZNF286A	0.47510	EVX2
0.67948	B4GALT4	0.47531	HNF1B
0.67978	IFT52	0.47552	TMEM31
0.68008	PDPK1	0.47573	GTPBP3
0.68038	AR	0.47594	ANKMY1
0.68068	PHLDB2	0.47615	LIN7B
0.68099	BCL2L1	0.47636	LOC401445
0.68129	SEC16A	0.47657	RINL
0.68159	RND3	0.47678	TEX13B
0.68189	NCRNA00158	0.47699	ITGA11
0.68219	ZXDA	0.47720	ADAM3A
0.68249	SPATA17	0.47741	HINT1
0.68279	PRLR	0.47762	LOC100132396
0.68309	ZDHHC1	0.47783	SLC10A2
0.68339	LOC340017	0.47804	CBX8
0.68369	CSNK2A1	0.47825	LOC100130027
0.68399	SLITRK6	0.47846	CCDC62
0.68429	TNFRSF1A	0.47867	TIMM16
0.68459	SCARNA13	0.47888	KIAA1609
0.68489	AQP11	0.47909	DDX11
0.68519	C9orf43	0.47930	TBL2
0.68549	SNF8	0.47951	FLJ35934
0.68579	TNFRSF11A	0.47972	FAM83C
0.68609	PDIA3P	0.47993	TLX3
0.68639	DCT	0.48014	C1orf131
0.68669	LOC732096	0.48035	KIAA1908
0.68699	LOC147804	0.48056	PIK3R6
0.68729	RHEBL1	0.48077	LOC729994
0.68759	IFI44L	0.48098	IVL
0.68789	ZNF14	0.48119	UBQLNL
0.68819	ECE2	0.48140	EEF1A1
0.68850	ST8SIA2	0.48161	CFI
0.68880	C9orf130	0.48182	UBE2K
0.68910	RAB3IP	0.48203	PLEKHA4
0.68940	GIMAP2	0.48224	ACD
0.68970	NANOGP1	0.48245	DSCR10
0.69000	TEX12	0.48266	SRPK3
0.69030	ZNF800	0.48287	LY6G6C
0.69060	MR1	0.48308	SPANXN4
0.69090	REEP6	0.48329	FAM119B
0.69120	LMCD1	0.48350	CCDC108

0.69150	PKDCC	0.48372	LOC100125918
0.69180	ZNF267	0.48393	TUBB6
0.69210	MAP7D1	0.48414	P4HA2
0.69240	BNC2	0.48435	MNX1
0.69270	E2F6	0.48456	ENTPD2
0.69300	POLR2L	0.48477	TSP50
0.69330	MYLK	0.48498	ZNF816A
0.69360	TK2	0.48519	SLFNL1
0.69390	FUS	0.48540	CHTF18
0.69420	MYL3	0.48561	ZMYND12
0.69450	CDK5RAP3	0.48582	TMEM132E
0.69480	ERO1LB	0.48603	C16orf81
0.69510	WDR54	0.48624	CTRL
0.69540	LOC646627	0.48645	C19orf46
0.69570	SYNPO	0.48666	HMHA1
0.69600	SUOX	0.48687	ZNF430
0.69631	CASQ2	0.48708	CLEC4F
0.69661	C6orf202	0.48729	NOX5
0.69691	BAX	0.48750	KIF25
0.69721	FOXD4L2	0.48771	RNF4
0.69751	TMEM219	0.48792	CDK2
0.69781	CMYA5	0.48813	PCSK6
0.69811	CP	0.48834	PIWIL1
0.69841	FLJ27352	0.48855	ZNF554
0.69871	ADPRHL1	0.48876	LOC100129316
0.69901	ZNF284	0.48897	GPR84
0.69931	MAD2L2	0.48918	ZNF143
0.69961	ZNF789	0.48939	KHNYN
0.69991	LOC100288968	0.48960	GGT1
0.70021	CXorf1	0.48981	RPS21
0.70051	LOC645323	0.49002	LOC644093
0.70081	LOC729156	0.49023	POLD1
0.70111	PM20D2	0.49044	HTRA3
0.70141	EGR2	0.49065	FAM109A
0.70171	RNF122	0.49086	CHMP1B
0.70201	ATXN8OS	0.49107	LOC497256
0.70231	KLHDC9	0.49128	CCDC72
0.70261	PHF11	0.49149	ZP1
0.70291	EEF1E1	0.49170	ALDH1A2
0.70321	TIMP2	0.49191	FANK1
0.70351	FAM96A	0.49212	PFAS
0.70381	DIDO1	0.49233	ENTPD8

0.70412	COL11A1	0.49254	TARBP2
0.70442	B3GALNT2	0.49275	HLA-DOB
0.70472	XRCC3	0.49296	C1orf204
0.70502	TET2	0.49317	ACTG2
0.70532	TGFBI	0.49338	EME1
0.70562	C1orf84	0.49359	LOC100287158
0.70592	MMP16	0.49380	A1CF
0.70622	FNDC1	0.49401	WNT16
0.70652	MTUS2	0.49422	SSPO
0.70682	PACS2	0.49443	ZNF576
0.70712	C7orf59	0.49464	RPS6
0.70742	LOC100129917	0.49485	GCNT6
0.70772	C6orf114	0.49506	GLB1L
0.70802	ZBTB10	0.49527	SIGLEC10
0.70832	SH3D20	0.49548	ECHDC3
0.70862	NDUFB3	0.49569	C1orf175
0.70892	SPOP	0.49590	GPR172A
0.70922	RUVBL2	0.49611	COL6A2
0.70952	DEM1	0.49632	FAM160B2
0.70982	LOC100289402	0.49653	RIN1
0.71012	DDX60L	0.49674	NFKBIE
0.71042	FKBP4	0.49695	CKS1B
0.71072	ANKRD34B	0.49716	MEX3A
0.71102	ZNF789	0.49737	PHF11
0.71132	CCL2	0.49758	KIAA1751
0.71163	CTTNBP2NL	0.49779	DDX41
0.71193	ZNF788	0.49800	C19orf56
0.71223	FAM91A1	0.49821	KLB
0.71253	CCDC160	0.49842	TCEAL6
0.71283	MPHOSPH6	0.49863	SLC10A6
0.71313	LOC642648	0.49884	C8orf82
0.71343	HCG4	0.49905	LOC442676
0.71373	FAM82A2	0.49926	PSG8
0.71403	APOBEC3G	0.49947	ARMC6
0.71433	MIPEP	0.49968	C3P1
0.71463	OR7A5	0.49989	LOC730011
0.71493	RBAK	0.50011	SIX2
0.71523	FKBP14	0.50032	C9orf30
0.71553	PPIL6	0.50053	HKDC1
0.71583	LOC100288750	0.50074	THPO
0.71613	C1orf59	0.50095	IL22RA1
0.71643	ZNF516	0.50116	SF4

						0.71673	LOC100131727	0.50137	KLK5
						0.71703	TMUB2	0.50158	LOC100130171
						0.71733	CYTH2	0.50179	SLC13A4
						0.71763	COX7A2	0.50200	VWA2
						0.71793	OR4K14	0.50221	FMOD
						0.71823	FOXC2	0.50242	LOC100133142
						0.71853	ZBTB49	0.50263	TRAF2
						0.71883	KLKB1	0.50284	IL17RC
						0.71913	IGSF3	0.50305	HSP90AA4P
						0.71944	EXOG	0.50326	LOC389901
						0.71974	FAM123B	0.50347	CTRL
						0.72004	THEM4	0.50368	CD300LB
						0.72034	DFNA5	0.50389	FOLR2
						0.72064	LRRN2	0.50410	ASAP1IT1
						0.72094	RPL23AP53	0.50431	RFXANK
						0.72124	RHEB	0.50452	ST6GAL1
						0.72154	FPGS	0.50473	HDAC7
						0.72184	FLJ33534	0.50494	WDR90
						0.72214	FAM18A	0.50515	IRAK2
						0.72244	GUSBP1	0.50536	SLC13A2
						0.72274	PITPNM3	0.50557	C3orf51
						0.72304	MUSTN1	0.50578	USHBP1
						0.72334	LOC202181	0.50599	SLC22A9
						0.72364	ANAPC16	0.50620	HAS1
						0.72394	PLCE1	0.50641	PKP1
						0.72424	LOC100287921	0.50662	CREB3L3
						0.72454	TRPC3	0.50683	DHH
						0.72484	ZNF503	0.50704	MPO
						0.72514	CXCL12	0.50725	SERPINB5
						0.72544	ZNF711	0.50746	KAZALD1
						0.72574	PHF17	0.50767	MYH4
						0.72604	hCG_2015435	0.50788	C4orf23
						0.72634	LOC441046	0.50809	ZNF787
						0.72664	ANTXR2	0.50830	IRF2BP2
						0.72695	INO80D	0.50851	ZNF182
						0.72725	C7	0.50872	LRG1
						0.72755	STEAP1	0.50893	C19orf28
						0.72785	TLL1	0.50914	HIC1
						0.72815	SPRYD4	0.50935	SSPO
						0.72845	TMEM194A	0.50956	CD3G
						0.72875	KLF8	0.50977	LOC145216
						0.72905	PA2G4	0.50998	LOC100288032

						0.72935	LOC100287123	0.51019	CPB2
						0.72965	RASL11A	0.51040	ITGB6
						0.72995	LOC100128551	0.51061	BCL2L14
						0.73025	ARSJ	0.51082	ZBTB42
						0.73055	ZC3HC1	0.51103	GLYCAM1
						0.73085	ACOT1	0.51124	LOC100128262
						0.73115	LOC339788	0.51145	AK7
						0.73145	LYPD6B	0.51166	ZCCHC18
						0.73175	LOC100133928	0.51187	ADH6
						0.73205	MRPL13	0.51208	ANO1
						0.73235	LPCAT3	0.51229	HLA-G
						0.73265	ALG2	0.51250	SLC2A10
						0.73295	MYOM2	0.51271	LOXHD1
						0.73325	CCDC144A	0.51292	WFDC2
						0.73355	ZNF229	0.51313	KRTAP1-5
						0.73385	C14orf33	0.51334	MFSD9
						0.73415	PPIL5	0.51355	LOC222699
						0.73445	MRPL11	0.51376	UCP3
						0.73476	GVIN1	0.51397	C16orf93
						0.73506	LOC100272228	0.51418	NOL12
						0.73536	ALS2CR12	0.51439	PLK3
						0.73566	SATL1	0.51460	CLNK
						0.73596	UNC80	0.51481	CLEC5A
						0.73626	DNAJC25	0.51502	CCDC155
						0.73656	UQCRB	0.51523	TIMD4
						0.73686	ZNF266	0.51544	MAGEB10
						0.73716	LOC729222	0.51565	HSCB
						0.73746	LCORL	0.51586	ABCF1
						0.73776	ORAI3	0.51607	C20orf96
						0.73806	DYDC2	0.51628	ZNF414
						0.73836	LOC284581	0.51650	LOC390760
						0.73866	CTSK	0.51671	LOC339535
						0.73896	CD27	0.51692	ALAS2
						0.73926	ADAMTS19	0.51713	LOC100190938
						0.73956	GINS3	0.51734	LIPG
						0.73986	RAD54B	0.51755	TSEN34
						0.74016	POFUT1	0.51776	TRIB3
						0.74046	ZNF470	0.51797	GRWD1
						0.74076	UBE2CBP	0.51818	TMEM204
						0.74106	SS18L2	0.51839	C21orf41
						0.74136	PFN1	0.51860	CNGA1
						0.74166	SLC16A10	0.51881	KIF24

						0.74196	SLC3A2	0.51902	BHMT
						0.74226	LOC100289277	0.51923	LOC100294067
						0.74257	PAPL	0.51944	WDFY4
						0.74287	PAIP2	0.51965	LOC619207
						0.74317	LOC645676	0.51986	SARDH
						0.74347	PRKG2	0.52007	CCDC108
						0.74377	CPB1	0.52028	LOC100130933
						0.74407	PXDN	0.52049	EBF3
						0.74437	C4orf22	0.52070	SMOC2
						0.74467	UMPS	0.52091	PPCDC
						0.74497	LOC100130950	0.52112	LOC284630
						0.74527	RAD18	0.52133	NFKBIL2
						0.74557	PANK4	0.52154	BOKAS
						0.74587	HNRNPA1	0.52175	IRAK3
						0.74617	NXT1	0.52196	ACSF3
						0.74647	ANKRD6	0.52217	OTOG
						0.74677	MRPL33	0.52238	OR7C1
						0.74707	HUS1	0.52259	C2orf48
						0.74737	C9orf98	0.52280	HMOX1
						0.74767	ACAD10	0.52301	NCRNA00110
						0.74797	MIMT1	0.52322	ATP2A3
						0.74827	RPL8	0.52343	PRDM16
						0.74857	UNC119B	0.52364	ARR3
						0.74887	CDRT4	0.52385	PUSL1
						0.74917	CALML4	0.52406	IGF2BP1
						0.74947	BTBD11	0.52427	EFCAB4A
						0.74977	KCNJ12	0.52448	RUFY4
						0.75008	ACYP1	0.52469	ZNF668
						0.75038	LOC100131851	0.52490	GRB7
						0.75068	LOXL1	0.52511	SNX3
						0.75098	DNAH6	0.52532	FLJ30064
						0.75128	RFC4	0.52553	DENND2D
						0.75158	EIF1AY	0.52574	PKD2L2
						0.75188	CTAGE5	0.52595	ADRB3
						0.75218	STOM	0.52616	MGC27345
						0.75248	ZBTB34	0.52637	MAP3K6
						0.75278	LOC728153	0.52658	LOC340508
						0.75308	LOC100130463	0.52679	LOC642511
						0.75338	G2E3	0.52700	NRAP
						0.75368	ZFP82	0.52721	SLC4A5
						0.75398	LOC100126784	0.52742	NFIB
						0.75428	SLC6A4	0.52763	FAM154B

						0.75458	FREM3	0.52784	LOC339803
						0.75488	C4orf10	0.52805	H2AFJ
						0.75518	CIDEB	0.52826	ARL16
						0.75548	LOC100287578	0.52847	LOC646249
						0.75578	LOC647309	0.52868	PSG5
						0.75608	LOC100289347	0.52889	NCOR2
						0.75638	ZNF323	0.52910	FLJ39639
						0.75668	FN1	0.52931	IL7
						0.75698	ZNF595	0.52952	CENPF
						0.75728	ZNF736	0.52973	FLJ38122
						0.75758	LOC440742	0.52994	LOC100131193
						0.75789	HSPD1	0.53015	CCDC142
						0.75819	AG2	0.53036	ISY1
						0.75849	C12orf64	0.53057	ABHD11
						0.75879	LSM6	0.53078	AMT
						0.75909	HOXB3	0.53099	ODF1
						0.75939	BUD13	0.53120	RNASEH2C
						0.75969	CSDA	0.53141	LOC100128574
						0.75999	CHRNB3	0.53162	LOC440863
						0.76029	ACVR2B	0.53183	LOC152274
						0.76059	PGR	0.53204	MFI2
						0.76089	TNFRSF10C	0.53225	C5orf62
						0.76119	C15orf37	0.53246	FAM46D
						0.76149	EIF2B4	0.53267	LOC93432
						0.76179	ACVR2B	0.53289	MEX3B
						0.76209	GRIP2	0.53310	ECEL1
						0.76239	FAM160A2	0.53331	FAM60A
						0.76269	ZFP2	0.53352	FLJ35390
						0.76299	TAS2R46	0.53373	C2orf81
						0.76329	SPATA18	0.53394	KCNA6
						0.76359	RACGAP1	0.53415	C2orf50
						0.76389	ATAD2B	0.53436	CRB2
						0.76419	POLR2J4	0.53457	SOX14
						0.76449	IFNGR1	0.53478	LOC440131
						0.76479	CD151	0.53499	HAUS8
						0.76509	COMMD9	0.53520	FLJ25328
						0.76540	ZNF681	0.53541	OTX2OS1
						0.76570	XKR7	0.53562	PRDM11
						0.76600	SEC13	0.53583	SYCE1L
						0.76630	LACE1	0.53604	EPB42
						0.76660	DGKA	0.53625	CAV2
						0.76690	LGI1	0.53646	MEGF11

0.76720	C4orf23	0.53667	LOC285735
0.76750	UPF3A	0.53688	DNAH17
0.76780	SLC27A2	0.53709	LOC100130557
0.76810	SLC25A17	0.53730	OTOF
0.76840	RBM8A	0.53751	UBASH3A
0.76870	RBMS2	0.53772	YIF1B
0.76900	SRP14	0.53793	POP1
0.76930	GTPBP4	0.53814	ATP7B
0.76960	IL1RAPL2	0.53835	FUK
0.76990	SIAH1	0.53856	SYPL2
0.77020	ALKBH2	0.53877	MPV17L2
0.77050	SKA3	0.53898	SOX15
0.77080	OLFML2B	0.53919	ABCA17P
0.77110	NOV	0.53940	PABPC1
0.77140	KIAA0408	0.53961	HTR3A
0.77170	ANO8	0.53982	LATS2
0.77200	CASC1	0.54003	SSPO
0.77230	GORAB	0.54024	LOC729978
0.77260	GCNT1	0.54045	C7orf40
0.77290	ZKSCAN2	0.54066	PLB1
0.77321	ATG9B	0.54087	ARFRP1
0.77351	ZBTB1	0.54108	CYP4Z1
0.77381	C20orf108	0.54129	PLEKHG4B
0.77411	LOC441461	0.54150	NRL
0.77441	MYNN	0.54171	PCP2
0.77471	C10orf112	0.54192	EDC3
0.77501	CYP1B1	0.54213	LOC100131067
0.77531	BOC	0.54234	CST7
0.77561	GEMIN7	0.54255	CEP72
0.77591	NPFFR2	0.54276	TRIM66
0.77621	PARP11	0.54297	RNU12
0.77651	ALG3	0.54318	HSPC072
0.77681	TAS2R31	0.54339	LOC100287684
0.77711	ZNF660	0.54360	ING5
0.77741	KLRG1	0.54381	ZNF280A
0.77771	CASC2	0.54402	ZNF646
0.77801	FLJ38717	0.54423	KRT72
0.77831	TECTA	0.54444	AGTR1
0.77861	L1TD1	0.54465	INTS5
0.77891	GALNT2	0.54486	ALK
0.77921	RPL7L1	0.54507	LOC646730
0.77951	CD300A	0.54528	LOC651714

0.77981	WDR52	0.54549	RSPH6A
0.78011	ZNF761	0.54570	LOC100287491
0.78041	SNRPB	0.54591	EDA2R
0.78071	FAM86C	0.54612	SLC22A3
0.78102	FLJ30838	0.54633	NCRNA00173
0.78132	ESYT3	0.54654	ZSCAN21
0.78162	C9orf130	0.54675	TRAT1
0.78192	RIOK1	0.54696	DSC1
0.78222	WDR65	0.54717	WFDC3
0.78252	PPP1R2	0.54738	SOX11
0.78282	BCAM	0.54759	SLC5A7
0.78312	ADAM32	0.54780	ARAP3
0.78342	LTBP3	0.54801	PRKAG3
0.78372	ZBTB8A	0.54822	QSOX2
0.78402	URB2	0.54843	LOC100287776
0.78432	XRCC2	0.54864	C19orf36
0.78462	RPP40	0.54885	DNASE1
0.78492	CENPP	0.54906	ZSCAN12P1
0.78522	ZNF534	0.54928	SHH
0.78552	LOC100134868	0.54949	BMF
0.78582	LOC100133106	0.54970	FLJ38379
0.78612	STARD5	0.54991	MAPK12
0.78642	CNTD2	0.55012	YBX1P2
0.78672	FRMPD3	0.55033	LOC100134229
0.78702	ZNF619	0.55054	DKFZp451A211
0.78732	ZNF155	0.55075	ARID5A
0.78762	ATP7A	0.55096	FLJ11292
0.78792	ADPRH	0.55117	LOC645435
0.78822	LPHN1	0.55138	ADAMTS2
0.78853	SFRS2B	0.55159	DRD3
0.78883	B3GALT6	0.55180	DHRS13
0.78913	KBTBD8	0.55201	COL1A2
0.78943	OBFC1	0.55222	CCDC162
0.78973	RAP1B	0.55243	ALDH3B1
0.79003	EIF1AX	0.55264	GLB1L2
0.79033	AP2S1	0.55285	LOC100288659
0.79063	PPARG	0.55306	ANKRD6
0.79093	ASTN2	0.55327	BCDIN3D
0.79123	LOC145837	0.55348	GMPPB
0.79153	CCL23	0.55369	FLJ16779
0.79183	C5orf13	0.55390	LOC644662
0.79213	SUMO3	0.55411	LY9

						0.79243	POSTN	0.55432	B3GALT5
						0.79273	LOC731282	0.55453	LOC285463
						0.79303	CEBPG	0.55474	RGS19
						0.79333	ZC4H2	0.55495	LOC285191
						0.79363	EFCAB1	0.55516	FLJ36116
						0.79393	LHFPL3	0.55537	CBY1
						0.79423	HMGB3	0.55558	ZNF389
						0.79453	PDIA3	0.55579	ALMS1P
						0.79483	CYorf15A	0.55600	STAT5A
						0.79513	FAM71E1	0.55621	TDH
						0.79543	C1orf70	0.55642	B3GNT3
						0.79573	FLJ35024	0.55663	GP1BB
						0.79603	SCO1	0.55684	SCN10A
						0.79634	EDA	0.55705	DCDC2
						0.79664	ZNF334	0.55726	TLR9
						0.79694	DKFZp686M1136	0.55747	LOC100129292
						0.79724	ZNF404	0.55768	TM4SF5
						0.79754	AOX2P	0.55789	PARVG
						0.79784	C3orf64	0.55810	HCG27
						0.79814	SAFB2	0.55831	SYPL2
						0.79844	LOC100130906	0.55852	LEFTY2
						0.79874	LOC100131826	0.55873	TCHHL1
						0.79904	ZNF225	0.55894	DTNBP1
						0.79934	FAIM	0.55915	MPP3
						0.79964	GRID2	0.55936	PTGES3
						0.79994	C1QTNF1	0.55957	SPATC1
						0.80024	TGFB1I1	0.55978	FAM109B
						0.80054	ATP8B1	0.55999	LOC100131492
						0.80084	HAUS3	0.56020	KLHL17
						0.80114	NSFP1	0.56041	MKRN9P
						0.80144	PTGFR	0.56062	MGC16703
						0.80174	ZNF214	0.56083	TCTEX1D2
						0.80204	MINA	0.56104	NAGLU
						0.80234	ABCA11P	0.56125	LOC100130581
						0.80264	ZNF43	0.56146	C6orf81
						0.80294	FAM131C	0.56167	PSEN2
						0.80324	LOC100132850	0.56188	TMEM86B
						0.80354	GPR107	0.56209	P4HA3
						0.80384	LOC400590	0.56230	SULT2A1
						0.80415	TMSB15B	0.56251	ZNF385C
						0.80445	PPAPDC1B	0.56272	DGCR14
						0.80475	ETNK2	0.56293	MCEE

0.80505	KIAA1704	0.56314	LOC729177
0.80535	AKTIP	0.56335	EPB41
0.80565	RSU1	0.56356	DPYS
0.80595	C15orf58	0.56377	LOC100134018
0.80625	GPATCH4	0.56398	THRSP
0.80655	RBMS2	0.56419	LRRC25
0.80685	GSTM4	0.56440	IL6ST
0.80715	HPR	0.56461	DARC
0.80745	FRZB	0.56482	TNMD
0.80775	BACH2	0.56503	CDKN2BAS
0.80805	PSMD10	0.56524	C9orf147
0.80835	FAM91A1	0.56545	ZNF497
0.80865	LOC642533	0.56567	ASCL2
0.80895	ZFP41	0.56588	TBX18
0.80925	ADARB2	0.56609	BEND4
0.80955	ANKLE2	0.56630	FANCG
0.80985	ZNF322A	0.56651	TSPAN4
0.81015	BAT5	0.56672	PGC
0.81045	DKFZp686K1684	0.56693	SDPR
0.81075	TAS2R14	0.56714	GLIS1
0.81105	DLL3	0.56735	ZNF316
0.81135	COCH	0.56756	C20orf27
0.81166	TPRA1	0.56777	WDR5B
0.81196	ITGA8	0.56798	USP43
0.81226	DYRK3	0.56819	LOC646471
0.81256	RPS28	0.56840	HTR3D
0.81286	ZFAND6	0.56861	GTPBP5
0.81316	CIDEB	0.56882	LOC100131606
0.81346	LOC729680	0.56903	HOXC9
0.81376	MYOZ2	0.56924	COL4A5
0.81406	CREM	0.56945	KRT40
0.81436	MFSD10	0.56966	BEGAIN
0.81466	FZD7	0.56987	LOC647252
0.81496	NCAPD3	0.57008	RLTPR
0.81526	GLRX	0.57029	C21orf119
0.81556	PPARGC1A	0.57050	MXRA8
0.81586	GPR26	0.57071	IRX4
0.81616	SMPD2	0.57092	RAD9A
0.81646	TAL1	0.57113	APOL1
0.81676	SMPDL3A	0.57134	FLJ45256
0.81706	ANKRD45	0.57155	WDR34
0.81736	LOC643085	0.57176	IL8RB

						0.81766	LOC285141	0.57197	LOC100132905
						0.81796	CKLF	0.57218	ZBTB48
						0.81826	GPC6	0.57239	FLJ13224
						0.81856	EIF2S3	0.57260	MYBPH
						0.81886	SPDYE7P	0.57281	MFNG
						0.81916	LOC100289204	0.57302	SOX17
						0.81947	SPTY2D1	0.57323	LOC728264
						0.81977	CCDC101	0.57344	NYNRIN
						0.82007	PDZRN3	0.57365	WNT9B
						0.82037	CBLB	0.57386	C9orf62
						0.82067	C12orf41	0.57407	ADAMTS15
						0.82097	PPAPDC2	0.57428	RASSF1
						0.82127	LOC100288459	0.57449	UBL5
						0.82157	AIMP2	0.57470	SLC39A5
						0.82187	CCDC69	0.57491	LOC100287816
						0.82217	NRBF2	0.57512	LOC219731
						0.82247	LOC728142	0.57533	CLDN2
						0.82277	ZNF620	0.57554	SUSD2
						0.82307	KLHDC8A	0.57575	LOC100289637
						0.82337	ZNF28	0.57596	EPHA8
						0.82367	COL24A1	0.57617	SRD5A1P1
						0.82397	ZNF222	0.57638	EPHA10
						0.82427	CBLL1	0.57659	MICALCL
						0.82457	MED30	0.57680	LOC151171
						0.82487	PIGO	0.57701	PTGFR
						0.82517	ALDH18A1	0.57722	RPS9
						0.82547	PDSS2	0.57743	TAF1L
						0.82577	ZCCHC7	0.57764	ADAMTS16
						0.82607	PPOX	0.57785	FLJ90757
						0.82637	ST7OT4	0.57806	PADI6
						0.82667	MID2	0.57827	ARSE
						0.82698	HIST1H2BC	0.57848	TUBA8
						0.82728	LOC441204	0.57869	HOXA6
						0.82758	FASTK	0.57890	CD3E
						0.82788	NYNRIN	0.57911	TNFSF11
						0.82818	MID2	0.57932	LOC151146
						0.82848	KIAA1383	0.57953	LOC200772
						0.82878	TP53I3	0.57974	C20orf134
						0.82908	RPS4X	0.57995	CCDC87
						0.82938	RPS6KB1	0.58016	PQLC2
						0.82968	RERGL	0.58037	PDXDC2
						0.82998	ANKRD37	0.58058	LCE3B

0.83028	SEMA4G	0.58079	TSPAN16
0.83058	PRR16	0.58100	SHISA3
0.83088	MTF1	0.58121	NDUFA4L2
0.83118	CYR61	0.58142	FAM9B
0.83148	LOC389895	0.58163	MVK
0.83178	DTX4	0.58184	GAL3ST2
0.83208	C13orf18	0.58206	LOC100128714
0.83238	KIAA0408	0.58227	DDX49
0.83268	EIF1	0.58248	ADAMTSL1
0.83298	DAGLB	0.58269	TMC8
0.83328	HYAL2	0.58290	TRERF1
0.83358	C6orf182	0.58311	DBF4B
0.83388	ZNF587	0.58332	MYH3
0.83418	DACT1	0.58353	GLIPR1L1
0.83448	FANCF	0.58374	DHRS12
0.83479	ARHGEF33	0.58395	DNAH17
0.83509	LOC100009676	0.58416	XIST
0.83539	TAP2	0.58437	PRDM14
0.83569	AGFG2	0.58458	ZNF71
0.83599	FAM19A4	0.58479	TPM4
0.83629	MED31	0.58500	LOC100288925
0.83659	POLR3H	0.58521	RPL19
0.83689	MCM3AP	0.58542	CXCL17
0.83719	TMEM19	0.58563	SP9
0.83749	FAM96B	0.58584	SMCP
0.83779	FAM91A2	0.58605	CNN1
0.83809	C12orf64	0.58626	LOC100132764
0.83839	OCA2	0.58647	DSG4
0.83869	OXTR	0.58668	LOC400752
0.83899	ZNF322A	0.58689	ACAD9
0.83929	LOXL2	0.58710	PARD6A
0.83959	PXMP2	0.58731	LOC729658
0.83989	LOC100131342	0.58752	LRRC33
0.84019	THBS1	0.58773	CCBP2
0.84049	MGMT	0.58794	PTPRCAP
0.84079	CCDC102B	0.58815	CROCC
0.84109	KIAA1310	0.58836	C6orf191
0.84139	TDO2	0.58857	COMTD1
0.84169	C19orf12	0.58878	C9orf140
0.84199	C8orf59	0.58899	RBM20
0.84229	SLC9A2	0.58920	FAM117A
0.84260	C8orf83	0.58941	ANGPT2

0.84290	C8orf41	0.58962	PROZ
0.84320	RBMS1	0.58983	C1orf105
0.84350	ROR1	0.59004	ZNF563
0.84380	NDUFB1	0.59025	C7orf16
0.84410	REL	0.59046	WDR4
0.84440	UBXN11	0.59067	LOC286114
0.84470	ACVR2B	0.59088	ESAM
0.84500	ZNF284	0.59109	LOC645955
0.84530	ASAH2	0.59130	CKLF
0.84560	RAD1	0.59151	KRT75
0.84590	CASP7	0.59172	RRP9
0.84620	C3orf33	0.59193	NDUFB7
0.84650	KRT18	0.59214	HJURP
0.84680	CABC1	0.59235	C4orf44
0.84710	C12orf64	0.59256	LOC100422781
0.84740	ATF7IP2	0.59277	FEZF1
0.84770	CCNJ	0.59298	PSAPL1
0.84800	STK32B	0.59319	MAB21L2
0.84830	C7orf68	0.59340	SLC2A9
0.84860	PCDHA6	0.59361	PROKR2
0.84890	GMPR	0.59382	LOC100292680
0.84920	RPL37A	0.59403	OBFC2A
0.84950	VWC2L	0.59424	TNKS1BP1
0.84980	GRP	0.59445	LOC100128818
0.85011	ARL4D	0.59466	NAT9
0.85041	PKP2	0.59487	MFAP4
0.85071	PXMP4	0.59508	GATA5
0.85101	GRK4	0.59529	DLX2
0.85131	SLC15A3	0.59550	RPA2
0.85161	MRPL32	0.59571	AXIN1
0.85191	BTN2A1	0.59592	KRT31
0.85221	CHRNA6	0.59613	SLC12A7
0.85251	RBMS3	0.59634	LOC100287684
0.85281	SLC35A3	0.59655	FAM173A
0.85311	POLR2D	0.59676	LOC100288756
0.85341	FBXO48	0.59697	ANGPTL6
0.85371	FOXK1	0.59718	CCDC88C
0.85401	ZNF48	0.59739	TRIM10
0.85431	KIAA0232	0.59760	DCLK3
0.85461	FILIP1L	0.59781	ASAP3
0.85491	MGAT1	0.59802	KLK10
0.85521	FAM91A1	0.59823	MEI1

						0.85551	ZBTB6	0.59845	C20orf57
						0.85581	POLR3K	0.59866	IRF3
						0.85611	GALNT4	0.59887	SUMO1
						0.85641	ZNF350	0.59908	LOC100289162
						0.85671	VAV3	0.59929	ZDHHC8
						0.85701	WARS2	0.59950	WDR53
						0.85731	TMEM68	0.59971	APOA1
						0.85761	ADCY2	0.59992	SVEP1
						0.85792	FLJ36031	0.60013	ZNF707
						0.85822	DET1	0.60034	ADCY4
						0.85852	ARHGAP24	0.60055	NRIP1
						0.85882	ZNF642	0.60076	RAPH1
						0.85912	BAHD1	0.60097	GPSM3
						0.85942	LCMT2	0.60118	MYH7
						0.85972	EXOSC4	0.60139	AQP5
						0.86002	LOC147727	0.60160	FLJ40852
						0.86032	C12orf64	0.60181	FOSB
						0.86062	SOX4	0.60202	SEMA4F
						0.86092	ZNF285	0.60223	C6orf57
						0.86122	ZNF417	0.60244	FETUB
						0.86152	UHRF1BP1	0.60265	CHMP2A
						0.86182	PPIE	0.60286	CILP
						0.86212	SUV39H2	0.60307	PPAN-P2RY11
						0.86242	NUP54	0.60328	MOGS
						0.86272	SEC62	0.60349	CHMP4C
						0.86302	ARHGAP10	0.60370	FTHL17
						0.86332	SERPINI2	0.60391	WDR81
						0.86362	ZNF836	0.60412	CCL21
						0.86392	LOC283045	0.60433	MBLAC1
						0.86422	ABCD2	0.60454	CHCHD7
						0.86452	HP1BP3	0.60475	LMAN1L
						0.86482	FLNA	0.60496	C19orf52
						0.86512	PI15	0.60517	SLC37A2
						0.86543	PNPLA5	0.60538	TLR6
						0.86573	BRMS1L	0.60559	ZNF333
						0.86603	CHST5	0.60580	LOC100287578
						0.86633	SUMO2	0.60601	PXMP4
						0.86663	GTF2E2	0.60622	RBP3
						0.86693	CHAC2	0.60643	UNKL
						0.86723	RNF213	0.60664	RPA3
						0.86753	ABCD2	0.60685	FAM113B
						0.86783	BTG2	0.60706	ZNF498

0.86813	LOC100287428	0.60727	FAM154A
0.86843	FLJ35390	0.60748	ASB5
0.86873	BEND7	0.60769	HILS1
0.86903	HOMER2	0.60790	CD72
0.86933	AHCTF1	0.60811	LOC120364
0.86963	FREM3	0.60832	WIBG
0.86993	CYP26B1	0.60853	XKR8
0.87023	CA12	0.60874	DPH5
0.87053	MBD6	0.60895	NCRNA00183
0.87083	C12orf66	0.60916	ESRP1
0.87113	PTRH2	0.60937	OR10G4
0.87143	LOC100133286	0.60958	FLJ35816
0.87173	C12orf64	0.60979	ATG16L2
0.87203	METTL3	0.61000	SCAND2
0.87233	DUS2L	0.61021	ACVR2B
0.87263	NUP210	0.61042	RSAD2
0.87293	MAFB	0.61063	SULT1A1
0.87324	GAL	0.61084	TBL1Y
0.87354	LOC401093	0.61105	MAB21L1
0.87384	C1orf161	0.61126	C3orf47
0.87414	LAMA2	0.61147	LOC730102
0.87444	CACNA1E	0.61168	LOC55908
0.87474	RBBP4	0.61189	FLJ41649
0.87504	CDHR3	0.61210	MX2
0.87534	MAGI1	0.61231	LOC391359
0.87564	IDO2	0.61252	KCNK10
0.87594	UBN2	0.61273	GLP2R
0.87624	ZNF724P	0.61294	DAZL
0.87654	SLN	0.61315	LOC643368
0.87684	REXO2	0.61336	TRIAP1
0.87714	ZNF773	0.61357	ARMCX6
0.87744	PPA1	0.61378	RPL21P44
0.87774	GNB2L1	0.61399	QRFP
0.87804	EID3	0.61420	LOC100126584
0.87834	FAM188B	0.61441	HSPBP1
0.87864	NYNRIN	0.61462	SLC29A2
0.87894	PLEKHG4	0.61484	NOS3
0.87924	PRKCH	0.61505	NDUFA4L2
0.87954	AGPHD1	0.61526	TOP
0.87984	MED17	0.61547	SCARNA22
0.88014	TNRC18	0.61568	ATP6V0E2
0.88044	EXOSC7	0.61589	LOC100133142

0.88074	PPP1R1A	0.61610	CAD
0.88105	MRPS31	0.61631	LOC100130128
0.88135	PHIP	0.61652	CSK
0.88165	FTSJD1	0.61673	FLJ44606
0.88195	NR2C1	0.61694	CREB3L4
0.88225	SST	0.61715	LOC729314
0.88255	ST8SIA4	0.61736	OPN4
0.88285	C12orf64	0.61757	ANKS4B
0.88315	MXD4	0.61778	C19orf55
0.88345	ENTPD7	0.61799	TPSD1
0.88375	RIT1	0.61820	NFIB
0.88405	POLR3G	0.61841	WDR70
0.88435	LOC100287988	0.61862	QPRT
0.88465	CHRNA5	0.61883	PHLDB2
0.88495	DKFZP434I0714	0.61904	LOC284412
0.88525	ABCB8	0.61925	MLKL
0.88555	GLRX3	0.61946	NUP62CL
0.88585	CADPS2	0.61967	LLGL2
0.88615	GRPR	0.61988	LOC149134
0.88645	FTSJ2	0.62009	HPX
0.88675	GEMIN6	0.62030	MXD3
0.88705	CLYBL	0.62051	CXorf36
0.88735	RNMTL1	0.62072	DIRC1
0.88765	FAM164C	0.62093	LRRC18
0.88795	RPS6KA6	0.62114	BCAR1
0.88825	SBNO1	0.62135	OSBPL7
0.88856	C1orf85	0.62156	PTK7
0.88886	CETN4P	0.62177	EEF1DP3
0.88916	COL5A3	0.62198	ZNF433
0.88946	TIGD1	0.62219	LIME1
0.88976	ZFR2	0.62240	PHLDA2
0.89006	H6PD	0.62261	LOC100292721
0.89036	NTF3	0.62282	KIR3DX1
0.89066	DPF3	0.62303	C9orf100
0.89096	LOC222070	0.62324	AMH
0.89126	KDELR3	0.62345	ITGB3
0.89156	G0S2	0.62366	PLAU
0.89186	CFL1	0.62387	LOC100133545
0.89216	MGAT2	0.62408	MAPK3
0.89246	REXO1L1	0.62429	FLJ46121
0.89276	FUT4	0.62450	AGAP11
0.89306	ANKRD49	0.62471	VPS53

0.89336	PHF1	0.62492	C1orf187
0.89366	E2F3	0.62513	HERC2P4
0.89396	TAGLN2	0.62534	ANKFY1
0.89426	GNA14	0.62555	LOC100287824
0.89456	CRH	0.62576	PRMT7
0.89486	ZNF461	0.62597	DAXX
0.89516	ZNF389	0.62618	CBLC
0.89546	CASP5	0.62639	LOC100129203
0.89576	SPG21	0.62660	C1orf69
0.89606	TMEM53	0.62681	SOX12
0.89637	PRKD1	0.62702	SAMD10
0.89667	CCND3	0.62723	IL18BP
0.89697	PDCD2L	0.62744	IL17F
0.89727	TIMM10	0.62765	C1orf104
0.89757	TM2D1	0.62786	SIRPG
0.89787	CCT6A	0.62807	TCF7L1
0.89817	ZMYM1	0.62828	PIP5KL1
0.89847	SIAH2	0.62849	C4orf48
0.89877	GINS1	0.62870	DIRC3
0.89907	SCTR	0.62891	ZNF496
0.89937	HNMT	0.62912	DCPS
0.89967	C1orf212	0.62933	SSX8
0.89997	C3orf18	0.62954	FMO6P
0.90027	LOC100131089	0.62975	SPDYC
0.90057	LOC391073	0.62996	NANP
0.90087	LOC100131180	0.63017	HOXD9
0.90117	TYW3	0.63038	IRX6
0.90147	C10orf32	0.63059	CCDC147
0.90177	PDSS1	0.63080	ZBTB32
0.90207	RPL17	0.63101	LOC729454
0.90237	MTHFD2	0.63123	PKMYT1
0.90267	TRMU	0.63144	ZFAND5
0.90297	PLEKHM1	0.63165	C16orf71
0.90327	SPAG6	0.63186	OBFC1
0.90357	ZBTB25	0.63207	MAGEC1
0.90388	C4orf31	0.63228	C1orf35
0.90418	SDHAP1	0.63249	HPD
0.90448	LPXN	0.63270	LOC100129196
0.90478	GTF2H2B	0.63291	FLJ43826
0.90508	ZC3H10	0.63312	LOC100287379
0.90538	PRKY	0.63333	LOC643749
0.90568	C3orf19	0.63354	ORM2

0.90598	RPL41	0.63375	CHRNE
0.90628	NPNT	0.63396	DLX6AS
0.90658	PCNXL2	0.63417	SCAND1
0.90688	C6orf155	0.63438	FAM81B
0.90718	CXorf26	0.63459	ZNF618
0.90748	DUT	0.63480	SSBP3
0.90778	WDR8	0.63501	HSPB2
0.90808	SLC24A3	0.63522	STAR
0.90838	ACVR1C	0.63543	ZNF799
0.90868	FLJ44253	0.63564	NLRC3
0.90898	RPS4Y1	0.63585	ADAD2
0.90928	DHRS7	0.63606	IL1RN
0.90958	KLHL14	0.63627	RBPMS2
0.90988	GRIK4	0.63648	HMGA2
0.91018	MDC1	0.63669	BRIP1
0.91048	SGCZ	0.63690	FAM105B
0.91078	LOC100287465	0.63711	TSSC4
0.91108	KIAA0754	0.63732	LOC100131320
0.91138	LOC100289338	0.63753	TCL6
0.91169	GALM	0.63774	ALOX12P2
0.91199	ZBTB39	0.63795	PRAMEF10
0.91229	GYG2	0.63816	LOC389676
0.91259	IER2	0.63837	NID2
0.91289	GSTZ1	0.63858	TRH
0.91319	COX4NB	0.63879	LOC642620
0.91349	CIB2	0.63900	C1orf109
0.91379	FREM3	0.63921	DNAH9
0.91409	LOC100129721	0.63942	C1orf126
0.91439	IGFBP4	0.63963	CTNS
0.91469	CHCHD6	0.63984	FCGRT
0.91499	TLR6	0.64005	TMEM44
0.91529	GPR83	0.64026	DNAH2
0.91559	TAS2R20	0.64047	ZNF385C
0.91589	MAFA	0.64068	PPP1R16A
0.91619	LOC401097	0.64089	JMJD4
0.91649	LOC254100	0.64110	CDH23
0.91679	FAM163A	0.64131	PLEKHH3
0.91709	KIAA0020	0.64152	FLJ36777
0.91739	FREM3	0.64173	TNFRSF1B
0.91769	IL1RAP	0.64194	LOC139201
0.91799	LMO1	0.64215	RPS6KA4
0.91829	LOC100132301	0.64236	LOC285902

0.91859	AGAP6	0.64257	SLC16A12
0.91889	FAM169A	0.64278	C15orf27
0.91919	PDGFRL	0.64299	KRTAP11-1
0.91950	PTPN2	0.64320	ZSCAN18
0.91980	C2orf52	0.64341	UPP2
0.92010	LOC100130218	0.64362	SIGLEC12
0.92040	MMAB	0.64383	LOC729153
0.92070	CALR3	0.64404	C19orf40
0.92100	NSUN2	0.64425	C10orf2
0.92130	COIL	0.64446	GPR45
0.92160	CIDEB	0.64467	ZIK1
0.92190	RRP7A	0.64488	SYCE2
0.92220	CHSY1	0.64509	LOC400043
0.92250	ZNF35	0.64530	CYP2S1
0.92280	MTERFD1	0.64551	FBF1
0.92310	B3GAT3	0.64572	THSD4
0.92340	ATP5G2	0.64593	TSPAN11
0.92370	TCF7L2	0.64614	ZC3H12A
0.92400	CECR7	0.64635	ARHGAP36
0.92430	PCDHB11	0.64656	CDCA3
0.92460	HSPA8	0.64677	CDH7
0.92490	LOC100289263	0.64698	MGC20647
0.92520	PNOC	0.64719	SVEP1
0.92550	PCDH11X	0.64740	C7orf29
0.92580	FAM65B	0.64762	STX18
0.92610	ALG10	0.64783	LOC100128075
0.92640	PDLIM2	0.64804	CCDC40
0.92670	TCEA3	0.64825	TSPAN9
0.92701	TMEM86A	0.64846	MMP14
0.92731	KDM4D	0.64867	LOC100129510
0.92761	SMAD4	0.64888	IGK@
0.92791	LOC400541	0.64909	CES7
0.92821	SKIL	0.64930	C3orf22
0.92851	VASH2	0.64951	MPZ
0.92881	SET	0.64972	JAKMIP3
0.92911	WBSCR16	0.64993	GNG8
0.92941	C14orf23	0.65014	DACT2
0.92971	PHC3	0.65035	LOC731957
0.93001	ALOX5	0.65056	LOC100132787
0.93031	HEG1	0.65077	HNRNPCL1
0.93061	C8orf37	0.65098	PUF60
0.93091	DUSP2	0.65119	RGSL1

0.93121	SAMD5	0.65140	SSPO
0.93151	ZNF221	0.65161	FBXO31
0.93181	LOC100129461	0.65182	TDRD12
0.93211	LOC388692	0.65203	PMVK
0.93241	C20orf30	0.65224	ZDHHC3
0.93271	LOC339290	0.65245	TFPI
0.93301	HAPLN1	0.65266	C7orf52
0.93331	TXNDC17	0.65287	SDK1
0.93361	SP140L	0.65308	NACA
0.93391	SLC35E3	0.65329	SHARPIN
0.93421	LOC401220	0.65350	RNF166
0.93451	LOC286382	0.65371	FBLN2
0.93482	ARHGEF33	0.65392	LRFN4
0.93512	LOC283713	0.65413	FMNL1
0.93542	LOC100131721	0.65434	MIR494
0.93572	SLC35D3	0.65455	ZFP36
0.93602	PDE7B	0.65476	CRYBA2
0.93632	SETDB1	0.65497	TEAD3
0.93662	DIDO1	0.65518	HTR1D
0.93692	ZNF689	0.65539	EN1
0.93722	ADAMTSL1	0.65560	ZNF434
0.93752	FN3K	0.65581	CLEC1A
0.93782	C15orf61	0.65602	MASP2
0.93812	NCRNA00169	0.65623	ZNF792
0.93842	C20orf4	0.65644	PGAM1
0.93872	BEND4	0.65665	MUCL1
0.93902	FBXO40	0.65686	F11R
0.93932	PNLDC1	0.65707	DTX2
0.93962	ARL4A	0.65728	ESPNL
0.93992	POLR2J4	0.65749	ALX3
0.94022	ROMO1	0.65770	MAP4K1
0.94052	EPHB3	0.65791	SNIP1
0.94082	CHST9	0.65812	LPO
0.94112	CXXC4	0.65833	SF3A3
0.94142	TMEM135	0.65854	KLC4
0.94172	C1S	0.65875	SLC25A26
0.94202	LSM10	0.65896	C9orf86
0.94233	CA8	0.65917	LOC100288669
0.94263	FOSL2	0.65938	NCRNA00116
0.94293	ZNF2	0.65959	CELA3A
0.94323	THSD7A	0.65980	C1orf89
0.94353	LOC645431	0.66001	SIGLEC15

0.94383	VIP	0.66022	LOC100132330
0.94413	TFDP2	0.66043	LOC100286961
0.94443	DKFZP686I15217	0.66064	TRPV2
0.94473	C2orf85	0.66085	SORBS3
0.94503	ZNF230	0.66106	LOC100132774
0.94533	SHISA6	0.66127	FAM71A
0.94563	FAM115C	0.66148	PLP2
0.94593	ZC3HAV1L	0.66169	LOC339290
0.94623	CD53	0.66190	MTHFD1L
0.94653	DDOST	0.66211	IKBKE
0.94683	SLC25A38	0.66232	FPR1
0.94713	MED7	0.66253	CROCCL1
0.94743	FAM118A	0.66274	UMODL1
0.94773	RPL14	0.66295	TEX264
0.94803	PTGS2	0.66316	CEP152
0.94833	TMEM100	0.66337	HAR1B
0.94863	C17orf106	0.66358	ANKRD31
0.94893	CD24	0.66379	NAT1
0.94923	MRPS33	0.66401	CENPBD1
0.94953	DPM3	0.66422	LTBR
0.94983	PIN4	0.66443	CNGB1
0.95014	THBD	0.66464	LRRC16B
0.95044	C2orf42	0.66485	ALG12
0.95074	BCL8	0.66506	TRIM66
0.95104	USP27X	0.66527	COL5A1
0.95134	C7orf13	0.66548	ACE
0.95164	MRTO4	0.66569	ROBLD3
0.95194	TNFSF9	0.66590	ZNF771
0.95224	SRRT	0.66611	CCNJL
0.95254	RCC2	0.66632	ICAM3
0.95284	GSTT1	0.66653	MRPL22
0.95314	RANGRF	0.66674	TBC1D16
0.95344	ZNF211	0.66695	CCR6
0.95374	TP53RK	0.66716	MCM5
0.95404	RND2	0.66737	LOC646870
0.95434	KL	0.66758	PIGW
0.95464	FLJ39739	0.66779	SLC6A5
0.95494	KLF2	0.66800	CARD11
0.95524	DDTL	0.66821	LTBP4
0.95554	MGC39372	0.66842	RPS27
0.95584	LOC677759	0.66863	MYL5
0.95614	C17orf103	0.66884	OTX2

0.95644	LOC153546	0.66905	DNAJC17
0.95674	DDX3Y	0.66926	LOC283050
0.95704	BEND4	0.66947	LOC389333
0.95734	LOC100128288	0.66968	ZNF678
0.95764	GEM	0.66989	COMMD5
0.95795	MTF1	0.67010	IQCA1L
0.95825	LRRC3	0.67031	ZNF234
0.95855	ZNF79	0.67052	TSNARE1
0.95885	ZNF571	0.67073	FADS6
0.95915	NCBP1	0.67094	SPINK4
0.95945	C12orf32	0.67115	LMOD2
0.95975	FLJ41757	0.67136	GPR161
0.96005	TNIP2	0.67157	C11orf48
0.96035	ZRSR2	0.67178	SVEP1
0.96065	C22orf32	0.67199	LOC100291029
0.96095	LOC389247	0.67220	MAP2K2
0.96125	JUNB	0.67241	LRDD
0.96155	C12orf64	0.67262	LOC100128505
0.96185	LOC147804	0.67283	PGM5
0.96215	SPEM1	0.67304	EPHB4
0.96245	ZNRD1	0.67325	PXN
0.96275	IFNAR2	0.67346	LOC284628
0.96305	ZDHHC24	0.67367	LOC400680
0.96335	RRP1B	0.67388	GABRE
0.96365	FAM188B2	0.67409	CBX4
0.96395	DKFZP779L1853	0.67430	HSD3B1
0.96425	NBR2	0.67451	CLDN1
0.96455	ZIC1	0.67472	EIF3K
0.96485	GPM6A	0.67493	MZF1
0.96515	CHODL	0.67514	BANP
0.96546	IER3	0.67535	COQ5
0.96576	MARVELD1	0.67556	HES5
0.96606	SMARCD3	0.67577	GBX2
0.96636	CXorf23	0.67598	KCNIP4
0.96666	C1orf151	0.67619	CD14
0.96696	GUCA1A	0.67640	FAM58A
0.96726	CCDC130	0.67661	GALNS
0.96756	JTB	0.67682	KRTAP12-4
0.96786	SHISA6	0.67703	DKFZp564H213
0.96816	DDIT3	0.67724	SLC22A13
0.96846	ZNHIT3	0.67745	LOC285547
0.96876	TGFBR1	0.67766	MYC

						0.96906	C3orf38	0.67787	C17orf101
						0.96936	JAG1	0.67808	ZXDC
						0.96966	UTY	0.67829	FLJ13744
						0.96996	LOC729680	0.67850	TRIM65
						0.97026	MCM9	0.67871	SMYD4
						0.97056	CRABP1	0.67892	C12orf42
						0.97086	FLJ34077	0.67913	ROR1
						0.97116	COMMD6	0.67934	CXXC1
						0.97146	ZFY	0.67955	AVPR2
						0.97176	S100A10	0.67976	ARAF
						0.97206	ZBED4	0.67997	TYSND1
						0.97236	ZBTB5	0.68018	PI4KA
						0.97266	SEC11C	0.68040	LOC728533
						0.97296	LOC344065	0.68061	FAM158A
						0.97327	CECR5	0.68082	EMILIN2
						0.97357	SATB2	0.68103	SPAG5
						0.97387	IMPG1	0.68124	SLC10A1
						0.97417	STH	0.68145	FLJ46120
						0.97447	RGS8	0.68166	VWA5B1
						0.97477	ZNF585B	0.68187	RAB11FIP1
						0.97507	EIF4A3	0.68208	LOC286370
						0.97537	GPR26	0.68229	C15orf58
						0.97567	KIAA1012	0.68250	ZNF324B
						0.97597	LOC153328	0.68271	ST8SIA2
						0.97627	BID	0.68292	ARFGAP1
						0.97657	EFTUD1	0.68313	GJB7
						0.97687	FAM101B	0.68334	ADAMTSL2
						0.97717	TAS2R19	0.68355	PDZD11
						0.97747	LYSMD1	0.68376	MYH7B
						0.97777	LOC654433	0.68397	SPAG4
						0.97807	PIGL	0.68418	CCDC51
						0.97837	LOC100272228	0.68439	LOC284080
						0.97867	ZNF614	0.68460	LOC100289624
						0.97897	SLC46A1	0.68481	LCNL1
						0.97927	NR4A3	0.68502	MAGOH
						0.97957	ADSL	0.68523	ZNF548
						0.97987	LOC729409	0.68544	STRC
						0.98017	RPL36AL	0.68565	ZNF691
						0.98047	CBX3	0.68586	LLPH
						0.98078	CH25H	0.68607	LOC392232
						0.98108	LOC153546	0.68628	RAMP1
						0.98138	LOC283027	0.68649	LOC401588

						0.98168	CSNK2B	0.68670	WDR25
						0.98198	FOS	0.68691	HDDC3
						0.98228	GRIN2A	0.68712	LOC100132159
						0.98258	FBLIM1	0.68733	NOG
						0.98288	FAM53B	0.68754	GEMIN8P4
						0.98318	NMNAT1	0.68775	UMPS
						0.98348	ZNF28	0.68796	FIBCD1
						0.98378	ZBTB16	0.68817	SCAMP3
						0.98408	MLYCD	0.68838	ABP1
						0.98438	C11orf51	0.68859	ARHGEF10L
						0.98468	LOC401052	0.68880	LOC100289116
						0.98498	NCRNA00201	0.68901	DERL3
						0.98528	COLEC12	0.68922	OR2J1
						0.98558	NEDD9	0.68943	RPL13A
						0.98588	TRERF1	0.68964	DUSP5P
						0.98618	AMIGO2	0.68985	P2RX4
						0.98648	ZNF558	0.69006	LOC100289671
						0.98678	LOC100288312	0.69027	INHBB
						0.98708	TMEM126A	0.69048	IRS1
						0.98738	MBTD1	0.69069	CEP250
						0.98768	ZNF766	0.69090	C19orf26
						0.98798	LOC285375	0.69111	S100A8
						0.98828	ST14	0.69132	TCEANC
						0.98859	C8orf4	0.69153	AARS2
						0.98889	SEMA4G	0.69174	PPP1R3F
						0.98919	DAZAP1	0.69195	PIK3CD
						0.98949	FLJ37453	0.69216	LOC728086
						0.98979	FCRLB	0.69237	PTAFR
						0.99009	C11orf10	0.69258	LOC730129
						0.99039	GAS8	0.69279	EBF1
						0.99069	SIRT5	0.69300	ANGPTL7
						0.99099	C21orf33	0.69321	APOL3
						0.99129	ZNF425	0.69342	B3GNTL1
						0.99159	LOC285456	0.69363	LOC729680
						0.99189	IFNGR2	0.69384	OR52K2
						0.99219	TMEM93	0.69405	ADAMTSL4
						0.99249	MCART1	0.69426	HOMER3
						0.99279	LOC284112	0.69447	LRRC23
						0.99309	TTTY15	0.69468	GALE
						0.99339	C19orf53	0.69489	DUS3L
						0.99369	NYNRIN	0.69510	AGMAT
						0.99399	PCDHB8	0.69531	TMEM150A

0.99429	SMCR8	0.69552	ZNF513
0.99459	SPCS2	0.69573	OPN1LW
0.99489	TBC1D15	0.69594	GFER
0.99519	NSMCE1	0.69615	HEATR4
0.99549	NLGN4Y	0.69636	MORF4L1
0.99579	TRPM3	0.69657	LOC646214
0.99609	PMAIP1	0.69679	SNRPD2
0.99640	RCN3	0.69700	HIST1H3F
0.99670	ZFAND5	0.69721	KRT14
0.99700	SMNDC1	0.69742	LOC285033
0.99730	VWA5A	0.69763	CDC23
0.99760	ZNF224	0.69784	NPFFR1
0.99790	C22orf40	0.69805	KIF26B
0.99820	TBCA	0.69826	PMS2L5
0.99850	SMU1	0.69847	OPRL1
0.99880	RPSAP58	0.69868	MED28
0.99910	LOC100287615	0.69889	CTNNBIP1
0.99940	SLC25A4	0.69910	SOX1
0.99970	KDM5D	0.69931	SLC7A9
		0.69952	TRHR
		0.69973	ILVBL
		0.69994	SCN4A
		0.70015	C3orf27
		0.70036	FAM123B
		0.70057	ITGAL
		0.70078	SNORD1C
		0.70099	CDK2AP2
		0.70120	HTR7P
		0.70141	IZUMO1
		0.70162	LOC401533
		0.70183	SVEP1
		0.70204	HDAC10
		0.70225	CCDC33
		0.70246	CRLS1
		0.70267	BARHL1
		0.70288	TTC30B
		0.70309	CHCHD4
		0.70330	C17orf62
		0.70351	HCN3
		0.70372	CATSPER2
		0.70393	ZNF341
		0.70414	ADAP2

0.70435	SCARF1
0.70456	SKIV2L
0.70477	IRAK1BP1
0.70498	IFT140
0.70519	ARSA
0.70540	CYHR1
0.70561	ITGB7
0.70582	LOC100130373
0.70603	NMUR1
0.70624	LY6K
0.70645	GTF2IRD1
0.70666	LOC100129365
0.70687	FLJ41170
0.70708	MYLK3
0.70729	DBNL
0.70750	PAPLN
0.70771	SILV
0.70792	PRM3
0.70813	TTC4
0.70834	SH3BP1
0.70855	ZFHX2
0.70876	KCNE4
0.70897	C2CD4D
0.70918	NCRNA00112
0.70939	LANCL3
0.70960	DCTPP1
0.70981	LOC154822
0.71002	TAZ
0.71023	LHFPL5
0.71044	FIZ1
0.71065	TBL3
0.71086	TMPRSS9
0.71107	C2orf16
0.71128	DIRAS3
0.71149	DNAH2
0.71170	SVEP1
0.71191	C19orf44
0.71212	SHE
0.71233	VANGL1
0.71254	C6orf35
0.71275	CHRM2
0.71296	PABPC4

							0.71318	VAX2
							0.71339	TMEM138
							0.71360	TJP3
							0.71381	GTF2H4
							0.71402	SSPO
							0.71423	LOC285780
							0.71444	CYP4A11
							0.71465	ADAMTS17
							0.71486	ZNF133
							0.71507	TOR2A
							0.71528	ZFPM1
							0.71549	TCP1P3
							0.71570	DPT
							0.71591	CCDC88B
							0.71612	NTHL1
							0.71633	CASP10
							0.71654	LGR6
							0.71675	SEMA6C
							0.71696	FAM110C
							0.71717	LOC286437
							0.71738	APOH
							0.71759	KRT80
							0.71780	PLD4
							0.71801	TPH2
							0.71822	OR2M4
							0.71843	RHPN1
							0.71864	SYT12
							0.71885	HSPB3
							0.71906	PSRC1
							0.71927	GNRH1
							0.71948	IMPA2
							0.71969	FAM128A
							0.71990	ZSCAN1
							0.72011	RAPSN
							0.72032	ONECUT2
							0.72053	KCNK12
							0.72074	FAM155B
							0.72095	PIRT
							0.72116	GOLT1A
							0.72137	FXYD6
							0.72158	COL28A1
							0.72179	ZNF821

							0.72200 C10orf71
							0.72221 LOC100287846
							0.72242 FAM159A
							0.72263 ARHGEF10
							0.72284 TMEM187
							0.72305 ANKRD5
							0.72326 C1orf217
							0.72347 ZSCAN22
							0.72368 LOC100134015
							0.72389 SERPINF2
							0.72410 IL20
							0.72431 TMEM71
							0.72452 FUT6
							0.72473 STOML1
							0.72494 LOC100286934
							0.72515 CTRL
							0.72536 ERAS
							0.72557 UBE2E1
							0.72578 LYG1
							0.72599 HAX1
							0.72620 ZNF71
							0.72641 FBXO46
							0.72662 RPL22L1
							0.72683 RCL1
							0.72704 SFRS13B
							0.72725 LHX6
							0.72746 AP4M1
							0.72767 ANKRD23
							0.72788 TMSB4Y
							0.72809 KAZ
							0.72830 CBLN3
							0.72851 SLA
							0.72872 LRRN4
							0.72893 LOC400553
							0.72914 APEX2
							0.72935 AGTRAP
							0.72957 ZNF549
							0.72978 GRAMD2
							0.72999 LOC440330
							0.73020 ASCL5
							0.73041 DHRS3
							0.73062 C18orf22

0.73083	DLK1
0.73104	MICALL2
0.73125	FTLP17
0.73146	VTN
0.73167	CBX2
0.73188	GLTPD2
0.73209	TNFAIP2
0.73230	PDZD11
0.73251	C9orf24
0.73272	TRIM25
0.73293	ZNF526
0.73314	LOC646214
0.73335	HIC2
0.73356	IFI27L1
0.73377	S100Z
0.73398	C14orf43
0.73419	NUDT1
0.73440	TRIM65
0.73461	C3orf52
0.73482	APOB
0.73503	PXN
0.73524	ACTA1
0.73545	RABL5
0.73566	LOC283104
0.73587	ORC6L
0.73608	SOSTDC1
0.73629	CD209
0.73650	LOC100129103
0.73671	LOC122038
0.73692	FLJ44896
0.73713	OAS3
0.73734	GNRHR2
0.73755	ZNF343
0.73776	CMTM8
0.73797	GLI4
0.73818	FAM101A
0.73839	LOC145678
0.73860	CABC1
0.73881	SUFU
0.73902	GPR149
0.73923	LOC390561
0.73944	AP2A2

							0.73965	MAMDC2
							0.73986	SEMA4G
							0.74007	NPPC
							0.74028	LOC729378
							0.74049	LEFTY1
							0.74070	UPF0639
							0.74091	LHX9
							0.74112	HTR1F
							0.74133	ZNF500
							0.74154	FAM73B
							0.74175	PSKH1
							0.74196	DGCR8
							0.74217	DMRT3
							0.74238	LAMB2L
							0.74259	IL10RA
							0.74280	BRD1
							0.74301	LOC100287149
							0.74322	EBP
							0.74343	PRR12
							0.74364	C5orf38
							0.74385	LOC131055
							0.74406	BACH1
							0.74427	SERF2
							0.74448	TMPRSS11D
							0.74469	LOC100130387
							0.74490	ZNF394
							0.74511	E4F1
							0.74532	LAMP3
							0.74553	LOC100129480
							0.74574	LOC285500
							0.74596	CSNK1D
							0.74617	FBLL1
							0.74638	KIFC3
							0.74659	TRPV1
							0.74680	ASL
							0.74701	MAGIX
							0.74722	PPP4C
							0.74743	BSPRY
							0.74764	RNF39
							0.74785	C8orf45
							0.74806	LOC442075
							0.74827	LOC100127947

0.74848	C11orf66
0.74869	TSPAN9
0.74890	TMIE
0.74911	TF
0.74932	ARC
0.74953	CCDC65
0.74974	SULT1A2
0.74995	CNTROB
0.75016	LOC391334
0.75037	SSPO
0.75058	PITX2
0.75079	BARX2
0.75100	DDX56
0.75121	CRELD2
0.75142	ODAM
0.75163	RAB12
0.75184	TMSB4X
0.75205	CARD6
0.75226	CHRD
0.75247	LOC100132284
0.75268	TLE2
0.75289	TMED6
0.75310	PHOSPHO1
0.75331	ZNF736
0.75352	GVIN1
0.75373	PCDHB16
0.75394	RGS16
0.75415	LOC729810
0.75436	LOC283404
0.75457	GHRLOS
0.75478	PTMA
0.75499	C1orf69
0.75520	RIOK3
0.75541	PSMD9
0.75562	LOC346702
0.75583	TMEM18
0.75604	RAMP3
0.75625	TBC1D17
0.75646	LOC100288305
0.75667	PRSS54
0.75688	WNT3A
0.75709	C9orf16

							0.75730	SNAPC4
							0.75751	CNFN
							0.75772	SH2D1B
							0.75793	CRAMP1L
							0.75814	VRK3
							0.75835	DACH2
							0.75856	PLA2G4A
							0.75877	TEX11
							0.75898	CYP26A1
							0.75919	CFDP1
							0.75940	TMEM208
							0.75961	FLJ38717
							0.75982	TH
							0.76003	GMFG
							0.76024	PAOX
							0.76045	MMP15
							0.76066	VAV1
							0.76087	LOC390595
							0.76108	C12orf40
							0.76129	CCDC93
							0.76150	EDN1
							0.76171	LOC645181
							0.76192	CTRL
							0.76213	CELA3B
							0.76235	GJC1
							0.76256	GSG1L
							0.76277	C8orf55
							0.76298	FAM78A
							0.76319	C1orf220
							0.76340	TMEM135
							0.76361	LOC100128131
							0.76382	CYCS
							0.76403	DBH
							0.76424	CXorf40A
							0.76445	STARD9
							0.76466	BOLA3
							0.76487	TCHH
							0.76508	OPRM1
							0.76529	PRSS21
							0.76550	DCLRE1B
							0.76571	POU4F1
							0.76592	NPPB

							0.76613	UBTD1
							0.76634	UFD1L
							0.76655	ZNF250
							0.76676	DPP4
							0.76697	CCNF
							0.76718	NBPF3
							0.76739	RBPJL
							0.76760	PLAC4
							0.76781	MCM3
							0.76802	WNT10B
							0.76823	NUFIP1
							0.76844	CHAF1A
							0.76865	UQCRQ
							0.76886	TCP10L
							0.76907	LOC339240
							0.76928	ARVCF
							0.76949	TAB1
							0.76970	FLJ37307
							0.76991	CSTB
							0.77012	FGFR1
							0.77033	LOC730109
							0.77054	LOC399715
							0.77075	DENR
							0.77096	TSPO
							0.77117	BLVRB
							0.77138	IGSF5
							0.77159	TUT1
							0.77180	ZNF782
							0.77201	PRTN3
							0.77222	LOC646513
							0.77243	CDK9
							0.77264	LOC100131389
							0.77285	C7orf61
							0.77306	EXOC7
							0.77327	C20orf46
							0.77348	C17orf51
							0.77369	C16orf48
							0.77390	ZNF75D
							0.77411	ISG20
							0.77432	SLC26A11
							0.77453	HMGCL
							0.77474	CCNE1

							0.77495	LOC100127983
							0.77516	PDE6H
							0.77537	CPSF3L
							0.77558	WRB
							0.77579	KLF11
							0.77600	RBM38
							0.77621	GPR6
							0.77642	GRTP1
							0.77663	ZNF225
							0.77684	AFMID
							0.77705	GLIS2
							0.77726	DFFB
							0.77747	ZBTB46
							0.77768	EPHA2
							0.77789	C1orf107
							0.77810	FLJ41327
							0.77831	CSNK1D
							0.77852	QPCTL
							0.77874	METTL7B
							0.77895	VNN3
							0.77916	PLK1
							0.77937	TRIM46
							0.77958	IGF2
							0.77979	LOC100129884
							0.78000	PLA2G6
							0.78021	UGGT1
							0.78042	ZNF784
							0.78063	ZNF816A
							0.78084	TRAF6
							0.78105	C12orf64
							0.78126	C20orf202
							0.78147	ADAMTS6
							0.78168	SPATS1
							0.78189	KBTBD5
							0.78210	ZNF419
							0.78231	B4GALT7
							0.78252	FAT2
							0.78273	TMEM132C
							0.78294	KIAA1920
							0.78315	HCRTR1
							0.78336	LOC645212
							0.78357	SNED1

							0.78378	CLEC4G
							0.78399	CLDN9
							0.78420	FDPS
							0.78441	HNRNPH2
							0.78462	ATXN7L1
							0.78483	ZBTB7C
							0.78504	RAVER1
							0.78525	HIST1H4H
							0.78546	DNAJB12
							0.78567	GPATCH2
							0.78588	PDIA6
							0.78609	KCND1
							0.78630	ZNF653
							0.78651	ZNF444
							0.78672	WDR16
							0.78693	LY6G6F
							0.78714	CDC45
							0.78735	HAPLN1
							0.78756	C17orf48
							0.78777	TMCO6
							0.78798	FAM165B
							0.78819	KRTAP12-3
							0.78840	LOC643327
							0.78861	LOC100129534
							0.78882	NFAT5
							0.78903	LOC390956
							0.78924	MESP1
							0.78945	FAM166A
							0.78966	CNGB1
							0.78987	TMEM42
							0.79008	ZIC1
							0.79029	MAGEE2
							0.79050	NFAM1
							0.79071	ZFAT
							0.79092	HAMP
							0.79113	DGAT2L7
							0.79134	LOC100130454
							0.79155	PILRA
							0.79176	FLJ30403
							0.79197	KCNQ1OT1
							0.79218	FBXL18
							0.79239	LOC730961

0.79260	TICAM1
0.79281	CHST12
0.79302	HDAC6
0.79323	PFDN5
0.79344	KIAA1949
0.79365	LOC100289081
0.79386	TSSK3
0.79407	FSIP2
0.79428	RSU1
0.79449	OR5F1
0.79470	FAM123B
0.79491	BMP1
0.79513	LOC285626
0.79534	C7orf55
0.79555	GADD45B
0.79576	GRM4
0.79597	WDR46
0.79618	EEF1A1P24
0.79639	CYBASC3
0.79660	ATAD2B
0.79681	REM2
0.79702	SPPL2B
0.79723	MTMR11
0.79744	RPL19P12
0.79765	LOC100294204
0.79786	KDM6B
0.79807	PWWP2B
0.79828	CSTF2
0.79849	LOC100128477
0.79870	SDSL
0.79891	NONO
0.79912	PSMD7
0.79933	DBT
0.79954	B3GALT4
0.79975	BYSL
0.79996	RYR1
0.80017	LOC100293158
0.80038	CNGA4
0.80059	PAPPA
0.80080	LOC645939
0.80101	CLNS1A
0.80122	LRRC61

							0.80143	C9orf131
							0.80164	HIST1H2BK
							0.80185	GREM2
							0.80206	LRRC26
							0.80227	NYNRIN
							0.80248	ZIC5
							0.80269	SMAD6
							0.80290	DCTN3
							0.80311	GMEB1
							0.80332	C20orf132
							0.80353	COPS5
							0.80374	C7orf27
							0.80395	SSPO
							0.80416	BACH1
							0.80437	GALR1
							0.80458	MTHFSD
							0.80479	PCNP
							0.80500	LOC100131170
							0.80521	NEU3
							0.80542	FAM128B
							0.80563	PTCHD2
							0.80584	DHX37
							0.80605	TMEM169
							0.80626	GMEB1
							0.80647	LAMB3
							0.80668	SDF2L1
							0.80689	LOC100189589
							0.80710	SEMA5A
							0.80731	PLEKHG2
							0.80752	HCRTR2
							0.80773	C16orf82
							0.80794	IPO4
							0.80815	PAGE1
							0.80836	OXSM
							0.80857	KCNJ1
							0.80878	CNN2
							0.80899	METTL2A
							0.80920	PRPH2
							0.80941	JMJD5
							0.80962	C9orf119
							0.80983	PTGIS
							0.81004	C6orf147

0.81025	C8orf38
0.81046	CAMP
0.81067	KIAA0664P3
0.81088	ZC3H3
0.81109	GPRC5C
0.81130	RBM28
0.81152	KLHL25
0.81173	STARD9
0.81194	RBP5
0.81215	REC8
0.81236	PIK3R5
0.81257	LOC151877
0.81278	TSC1
0.81299	CLK3
0.81320	FLJ37201
0.81341	LOC648262
0.81362	PDLIM4
0.81383	RWDD4A
0.81404	TAS2R60
0.81425	PIGR
0.81446	OXCT2
0.81467	RNF112
0.81488	TNNT2
0.81509	BTN2A3
0.81530	DPM2
0.81551	P4HA2
0.81572	ZBTB47
0.81593	TELO2
0.81614	MED19
0.81635	FLJ14107
0.81656	PSMD6
0.81677	PPP3CC
0.81698	HOTAIR
0.81719	LOC100291791
0.81740	SLC22A14
0.81761	NUCB2
0.81782	MGP
0.81803	HERC2P2
0.81824	LOC389831
0.81845	FAM120A
0.81866	C12orf45
0.81887	SHF

0.81908	LOC347376
0.81929	C10orf47
0.81950	GABRQ
0.81971	KIAA1652
0.81992	PSMD13
0.82013	FEN1
0.82034	RANBP1
0.82055	LOC441455
0.82076	LOC220930
0.82097	C10orf82
0.82118	RDH12
0.82139	RFXAP
0.82160	TAF11
0.82181	GUSB
0.82202	LOC90784
0.82223	PWRN1
0.82244	PPP2R2D
0.82265	MEP1A
0.82286	TCAP
0.82307	LOC100288714
0.82328	ERP29
0.82349	PNMA5
0.82370	MOCS3
0.82391	OR2A2
0.82412	DDX51
0.82433	NYNRIN
0.82454	TIRAP
0.82475	SPHK2
0.82496	SLC12A8
0.82517	C3orf53
0.82538	DNASE1L1
0.82559	TFR2
0.82580	CC2D1A
0.82601	C5orf45
0.82622	ZGPAT
0.82643	MED20
0.82664	C8ORFK29
0.82685	FAM132B
0.82706	ULBP2
0.82727	TXNDC6
0.82748	DRP2
0.82769	BMP4

0.82791	ATPIF1
0.82812	THAP3
0.82833	NARFL
0.82854	OR4A5
0.82875	FBXO17
0.82896	SLCO4C1
0.82917	TRIM67
0.82938	B3GNT9
0.82959	TUBB2C
0.82980	KIAA0174
0.83001	VPS53
0.83022	TNFRSF18
0.83043	ZNF76
0.83064	KLHL35
0.83085	C2orf89
0.83106	C4orf36
0.83127	NPY2R
0.83148	ATF4
0.83169	LOC401433
0.83190	C22orf36
0.83211	ZYG11A
0.83232	LCAT
0.83253	GAPDHL7
0.83274	RARRES1
0.83295	MLST8
0.83316	GPAT2
0.83337	LOC100132005
0.83358	SBF1P1
0.83379	ZNF747
0.83400	PDGFD
0.83421	P2RY11
0.83442	PQBP1
0.83463	FAM53A
0.83484	ZNF835
0.83505	NFKBID
0.83526	TSEN2
0.83547	LOC729324
0.83568	LOC100288472
0.83589	MON1A
0.83610	DEFB136
0.83631	WDR86
0.83652	CRMP1

							0.83673	VWC2L
							0.83694	CWC25
							0.83715	DGAT2L7
							0.83736	YY1AP1
							0.83757	COPG2
							0.83778	CCDC22
							0.83799	MGC16025
							0.83820	C1orf228
							0.83841	NMNAT3
							0.83862	LRRC48
							0.83883	VPS8
							0.83904	TRMT2A
							0.83925	SULT1A3
							0.83946	NOL3
							0.83967	TM9SF1
							0.83988	METTL1
							0.84009	ZNF282
							0.84030	C2orf24
							0.84051	HMX1
							0.84072	ATP9B
							0.84093	MRPL12
							0.84114	C10orf44
							0.84135	CCDC25
							0.84156	FRMD3
							0.84177	SPTB
							0.84198	GPR153
							0.84219	NPM3
							0.84240	SRCAP
							0.84261	KIF17
							0.84282	C10orf41
							0.84303	FAM13AOS
							0.84324	TMED4
							0.84345	DNAH6
							0.84366	BTBD11
							0.84387	SPEG
							0.84408	CCDC48
							0.84430	ICAM1
							0.84451	SGOL1
							0.84472	SHFM1
							0.84493	COMMD4
							0.84514	SLC25A41
							0.84535	ECM1

0.84556	LOC100131943
0.84577	ABCB8
0.84598	NHSL2
0.84619	HRH4
0.84640	TCTA
0.84661	SLC43A3
0.84682	C1RL
0.84703	EGR4
0.84724	LOC285014
0.84745	DNAH6
0.84766	FLJ21408
0.84787	CHRNA1
0.84808	EFNB1
0.84829	CCL5
0.84850	MYO5B
0.84871	IQCE
0.84892	CACNG5
0.84913	ARMC7
0.84934	FLJ36000
0.84955	TP53TG1
0.84976	PPARGC1B
0.84997	F2R
0.85018	ZNF317
0.85039	C19orf24
0.85060	MRPL48
0.85081	CYCS
0.85102	C17orf108
0.85123	NCKAP5L
0.85144	C14orf72
0.85165	DDHD1
0.85186	FOXP4
0.85207	PSMC1
0.85228	FFAR1
0.85249	NT5C
0.85270	C1S
0.85291	PET112L
0.85312	BLVRA
0.85333	LOC100287743
0.85354	TIGD5
0.85375	FADD
0.85396	LOC347549
0.85417	FAM83A

							0.85438	EIF2S2
							0.85459	PIH1D1
							0.85480	ZBTB3
							0.85501	OR9Q2
							0.85522	HIST1H1D
							0.85543	CWF19L1
							0.85564	LOC100131878
							0.85585	ELK3
							0.85606	CCDC117
							0.85627	NNMT
							0.85648	LOC643923
							0.85669	DPCD
							0.85690	PTRH1
							0.85711	TSNAXIP1
							0.85732	QRICH2
							0.85753	N4BP2L1
							0.85774	ZNF233
							0.85795	PML
							0.85816	RTL1
							0.85837	CLDND2
							0.85858	ISYNA1
							0.85879	PRSS8
							0.85900	PTP4A3
							0.85921	FKBP2
							0.85942	KLRG2
							0.85963	RCCD1
							0.85984	C14orf145
							0.86005	MDFI
							0.86026	PRPF40B
							0.86047	CHMP6
							0.86069	PDZK1
							0.86090	SSR2
							0.86111	KLK13
							0.86132	WFDC9
							0.86153	MRGPRF
							0.86174	FLJ42022
							0.86195	KRT10
							0.86216	GNA11
							0.86237	C10orf76
							0.86258	C19orf12
							0.86279	SRRM5
							0.86300	LOC100268168

0.86321	RPSAP19
0.86342	AFAP1L2
0.86363	CHRNA3
0.86384	RHO
0.86405	TMEM186
0.86426	FZR1
0.86447	ZFHX2
0.86468	C3orf42
0.86489	CHAF1B
0.86510	TRAM2
0.86531	ZIC2
0.86552	FREM3
0.86573	C1orf66
0.86594	XPO7
0.86615	N6AMT2
0.86636	C1orf53
0.86657	FAM196A
0.86678	LOC100294096
0.86699	LOC729291
0.86720	TSPAN11
0.86741	SHOX2
0.86762	POFUT2
0.86783	HSD3B7
0.86804	C1QTNF2
0.86825	C7orf26
0.86846	IGFBPL1
0.86867	N4BP3
0.86888	CENPJ
0.86909	PON1
0.86930	POLG
0.86951	SH3RF2
0.86972	LOC100127974
0.86993	NUDT13
0.87014	ZNF598
0.87035	CBY1
0.87056	ADRB2
0.87077	ZNF618
0.87098	ZNF737
0.87119	CYP2R1
0.87140	FUT2
0.87161	ARHGAP8
0.87182	PYCRL

0.87203	PDPN
0.87224	CYP2C19
0.87245	C20orf152
0.87266	CDC25B
0.87287	NTSR1
0.87308	RHOD
0.87329	LOC388796
0.87350	CDR2
0.87371	NPAS4
0.87392	ZNF33B
0.87413	RECQL5
0.87434	OTUD3
0.87455	SH2D5
0.87476	LOC286161
0.87497	LOC100289246
0.87518	GADD45GIP1
0.87539	MATK
0.87560	CTSZ
0.87581	GPR4
0.87602	TTLL3
0.87623	LRRC37A3
0.87644	LOC100130363
0.87665	LOC407835
0.87686	SEMA4G
0.87708	RPUSD2
0.87729	SYT6
0.87750	CECR4
0.87771	VWA5B2
0.87792	SOCS3
0.87813	DNAH6
0.87834	SCRN2
0.87855	RSPH1
0.87876	SCYL3
0.87897	GRID2IP
0.87918	UTP14A
0.87939	SLC10A4
0.87960	C17orf91
0.87981	FLJ45671
0.88002	BAIAP2L2
0.88023	KLHL13
0.88044	TMEM91
0.88065	C10orf58

							0.88086	NHLH2
							0.88107	C18orf18
							0.88128	SARM1
							0.88149	WIPI2
							0.88170	UBE2F
							0.88191	HTR1A
							0.88212	LOC100128164
							0.88233	SURF6
							0.88254	ZNF239
							0.88275	ZBTB47
							0.88296	DUS1L
							0.88317	NTN1
							0.88338	DBNL
							0.88359	PPM1M
							0.88380	CALCB
							0.88401	KRBA2
							0.88422	CAND2
							0.88443	BAHCC1
							0.88464	ITPRIPL1
							0.88485	HTR4
							0.88506	LOC283392
							0.88527	KLK4
							0.88548	SETMAR
							0.88569	CCDC78
							0.88590	PFDN4
							0.88611	RNF25
							0.88632	DALRD3
							0.88653	FAM83E
							0.88674	LYSMD4
							0.88695	SLC28A1
							0.88716	FLJ30375
							0.88737	SCP2
							0.88758	NARF
							0.88779	EPHB1
							0.88800	FABP6
							0.88821	ANKRD55
							0.88842	ANKRD18B
							0.88863	KIAA0802
							0.88884	SS18L1
							0.88905	LOC643699
							0.88926	LOC729862
							0.88947	RBMS1

							0.88968	CD7
							0.88989	LOC100132805
							0.89010	TARDBP
							0.89031	LOC440419
							0.89052	ARHGEF2
							0.89073	IL16
							0.89094	TPK1
							0.89115	LOC284440
							0.89136	UNC45A
							0.89157	KCNS3
							0.89178	DCDC1
							0.89199	LOC644063
							0.89220	BOD1L
							0.89241	PTGDR
							0.89262	IGSF9B
							0.89283	KIAA1609
							0.89304	LOC647302
							0.89325	ZFR2
							0.89347	ACTC1
							0.89368	CYP27C1
							0.89389	YJEFN3
							0.89410	JAKMIP3
							0.89431	GLRA1
							0.89452	SLC25A42
							0.89473	HPS6
							0.89494	KLHL29
							0.89515	RPS6KL1
							0.89536	SRCRB4D
							0.89557	SH3GL1P1
							0.89578	OTUB2
							0.89599	VANGL1
							0.89620	AMOTL1
							0.89641	C10orf110
							0.89662	ZNF259
							0.89683	LOC339524
							0.89704	C20orf197
							0.89725	RCE1
							0.89746	RSPO3
							0.89767	LOC100128191
							0.89788	DNAH2
							0.89809	SOX18
							0.89830	CEBPB

							0.89851	PTHLH
							0.89872	LOC100286934
							0.89893	CRHR2
							0.89914	RBM3
							0.89935	DBNL
							0.89956	TCF24
							0.89977	ZSWIM3
							0.89998	TSPAN18
							0.90019	NOP2
							0.90040	NEK8
							0.90061	CCL28
							0.90082	C2orf72
							0.90103	HTR1B
							0.90124	LOC339674
							0.90145	PCTP
							0.90166	GDPD1
							0.90187	EFNA4
							0.90208	LOC348926
							0.90229	SLC39A8
							0.90250	MSH2
							0.90271	ZNF593
							0.90292	ZNF140
							0.90313	BAT4
							0.90334	RP1L1
							0.90355	FAM43A
							0.90376	FBXW9
							0.90397	MED6
							0.90418	CLCNKA
							0.90439	HES3
							0.90460	CCDC13
							0.90481	PIK3R3
							0.90502	KCNK2
							0.90523	CCS
							0.90544	FLJ39061
							0.90565	C17orf102
							0.90586	ARPC1B
							0.90607	C12orf64
							0.90628	HTR2C
							0.90649	C21orf57
							0.90670	KIAA2022
							0.90691	LOC100293014
							0.90712	MBIP

							0.90733	GTF2E1
							0.90754	FFAR3
							0.90775	BMS1
							0.90796	PRKRA
							0.90817	NOP56
							0.90838	BFSP1
							0.90859	KIAA0090
							0.90880	MADCAM1
							0.90901	NCRNA00181
							0.90922	LOC100288798
							0.90943	PPIH
							0.90964	ZNF727
							0.90986	CSNK1D
							0.91007	NUDT22
							0.91028	LOC100128281
							0.91049	EMG1
							0.91070	ZNF568
							0.91091	LENG1
							0.91112	RPUSD3
							0.91133	PLAC2
							0.91154	KLHL18
							0.91175	SCP2
							0.91196	ZDHHC8
							0.91217	GPR179
							0.91238	MXRA7
							0.91259	CATSPER1
							0.91280	SPATA5
							0.91301	TCEAL1
							0.91322	STARD9
							0.91343	FBXO39
							0.91364	PCDHB9
							0.91385	AADACL3
							0.91406	C1orf74
							0.91427	SCUBE2
							0.91448	NAA20
							0.91469	FAM50B
							0.91490	CHRNA2
							0.91511	FAM188B2
							0.91532	RIPPLY2
							0.91553	LDLRAD2
							0.91574	C19orf63
							0.91595	SLC25A15

							0.91616	PLD6
							0.91637	AKAP13
							0.91658	SULT2B1
							0.91679	DLX6
							0.91700	C19orf68
							0.91721	X2828716
							0.91742	FOXQ1
							0.91763	ING1
							0.91784	KRT86
							0.91805	THAP1
							0.91826	RHBDL3
							0.91847	MAML1
							0.91868	VN1R2
							0.91889	BRF2
							0.91910	KCNJ11
							0.91931	LOC100130172
							0.91952	DUSP4
							0.91973	C1orf172
							0.91994	DGCR14
							0.92015	IGSF9B
							0.92036	CCDC71
							0.92057	ELP2P
							0.92078	GYS1
							0.92099	ALPPL2
							0.92120	LMO3
							0.92141	C12orf64
							0.92162	LOC400657
							0.92183	LOC201477
							0.92204	WNT2B
							0.92225	PAX5
							0.92246	ONECUT2
							0.92267	ZKSCAN4
							0.92288	TAF6L
							0.92309	DCBLD1
							0.92330	IFFO2
							0.92351	SLC7A6
							0.92372	RBM12B
							0.92393	SCFD1
							0.92414	LOC100131848
							0.92435	AKD1
							0.92456	KCNQ1OT1
							0.92477	TMEM90A

							0.92498	GPR78
							0.92519	TSPYL4
							0.92540	PARS2
							0.92561	TACR1
							0.92582	ZNF23
							0.92603	LOC285378
							0.92625	LOC283143
							0.92646	ECT2L
							0.92667	C10orf54
							0.92688	LOC100288820
							0.92709	HAGHL
							0.92730	TCIRG1
							0.92751	LOC730974
							0.92772	AMDHD2
							0.92793	XPNPEP1
							0.92814	CCL17
							0.92835	GABRQ
							0.92856	SHPK
							0.92877	SH3GL1P1
							0.92898	COTL1
							0.92919	LOC100287599
							0.92940	LRRC68
							0.92961	CHRNA2
							0.92982	CTCFL
							0.93003	LOC283177
							0.93024	KCNA6
							0.93045	ZNF567
							0.93066	FGF20
							0.93087	HIST1H4B
							0.93108	PCDHB17
							0.93129	EFCAB2
							0.93150	LRRC38
							0.93171	IP6K2
							0.93192	C20orf20
							0.93213	MAP1D
							0.93234	SEC16A
							0.93255	DNAH17
							0.93276	COL23A1
							0.93297	ADRA2C
							0.93318	MDK
							0.93339	AMZ1
							0.93360	SNRNP40

							0.93381	FAM71F2
							0.93402	LOC284014
							0.93423	C14orf45
							0.93444	CMPK2
							0.93465	RNU11
							0.93486	TCTN1
							0.93507	RNASEL
							0.93528	ISM1
							0.93549	LOC729313
							0.93570	LOC25845
							0.93591	EFCAB2
							0.93612	RGS14
							0.93633	FAM182A
							0.93654	PRKCZ
							0.93675	GRM8
							0.93696	MPND
							0.93717	DKC1
							0.93738	JAKMIP1
							0.93759	LOC100287552
							0.93780	NT5M
							0.93801	ONECUT2
							0.93822	GGN
							0.93843	ABTB1
							0.93864	MUM1
							0.93885	APOBEC3C
							0.93906	PARK7
							0.93927	RTDR1
							0.93948	C6orf47
							0.93969	MTR
							0.93990	SFRS2B
							0.94011	LOC286382
							0.94032	ZNF467
							0.94053	SLC22A10
							0.94074	TLK2
							0.94095	CCDC43
							0.94116	ZNF181
							0.94137	HOOK2
							0.94158	NEUROD1
							0.94179	FLT4
							0.94200	IGFL4
							0.94221	NLRC4
							0.94242	TEKT3

							0.94264	ZFP30
							0.94285	RASSF10
							0.94306	LOC554223
							0.94327	CCBE1
							0.94348	HSP90AB1
							0.94369	KIF2C
							0.94390	RDH13
							0.94411	SIM2
							0.94432	POLR2J2
							0.94453	TSPAN9
							0.94474	ZNF443
							0.94495	CNGB1
							0.94516	LOC652990
							0.94537	MED22
							0.94558	LRRC42
							0.94579	CALHM1
							0.94600	TEX19
							0.94621	RASL11B
							0.94642	EGFL8
							0.94663	LOC649873
							0.94684	APOF
							0.94705	LOC100287189
							0.94726	FLJ43276
							0.94747	C16orf42
							0.94768	SLC5A6
							0.94789	ING2
							0.94810	ANKH
							0.94831	NGB
							0.94852	SYT10
							0.94873	SLC47A1
							0.94894	SDK2
							0.94915	LOC158402
							0.94936	LOC441617
							0.94957	PGLS
							0.94978	RNF139
							0.94999	WDR55
							0.95020	TTR
							0.95041	TSPAN33
							0.95062	LOC202181
							0.95083	TCEAL4
							0.95104	C21orf88
							0.95125	LOC645139

							0.95146	NDUFS6
							0.95167	C20orf135
							0.95188	CCT8P1
							0.95209	VIPR2
							0.95230	TNFRSF10C
							0.95251	KLHL21
							0.95272	MMP12
							0.95293	TCP10
							0.95314	PPARGC1B
							0.95335	EEF1D
							0.95356	NYNRIN
							0.95377	RASL10A
							0.95398	NFYC
							0.95419	ZNF554
							0.95440	WNT5A
							0.95461	RPL27A
							0.95482	TSHZ2
							0.95503	ATPAF2
							0.95524	ZNF518B
							0.95545	EHD4
							0.95566	RNASE2
							0.95587	IFI30
							0.95608	FAM43B
							0.95629	ERICH1
							0.95650	SNAI2
							0.95671	TXN
							0.95692	PPP1R3D
							0.95713	TCEB3B
							0.95734	LRRC10B
							0.95755	TIMM44
							0.95776	LIMD1
							0.95797	HIST3H2BB
							0.95818	TSEN54
							0.95839	C10orf140
							0.95860	C15orf21
							0.95881	INSM2
							0.95903	ALG9
							0.95924	MIB2
							0.95945	TMEM233
							0.95966	LOC100144603
							0.95987	OPRD1
							0.96008	FAM91A2

0.96029	HRK
0.96050	TC2N
0.96071	TAS2R9
0.96092	TFIP11
0.96113	XPO1
0.96134	FAM161A
0.96155	LOC100131342
0.96176	C7orf11
0.96197	LOC644961
0.96218	LOC644794
0.96239	IL12RB2
0.96260	LOC171220
0.96281	LOC151484
0.96302	CENPV
0.96323	STARD9
0.96344	SIK1
0.96365	KIAA1199
0.96386	RGAG1
0.96407	LBH
0.96428	ZNF740
0.96449	ORAI1
0.96470	PPP1R3A
0.96491	DBT
0.96512	HIST1H4A
0.96533	ANK1
0.96554	OSCAR
0.96575	NMU
0.96596	SLC27A5
0.96617	C12orf64
0.96638	AKAP5
0.96659	AHSP
0.96680	LOC441601
0.96701	KIF9
0.96722	CCHCR1
0.96743	ACTG1
0.96764	ZSCAN5A
0.96785	AKAP8
0.96806	CMKLR1
0.96827	CIDEC
0.96848	FAM176B
0.96869	C9orf89
0.96890	LOC100294201

							0.96911	GAPDHP32
							0.96932	SULF1
							0.96953	AVEN
							0.96974	COX11
							0.96995	DNAJC30
							0.97016	RPUSD4
							0.97037	ORAOV1
							0.97058	LOC100288745
							0.97079	CCBE1
							0.97100	LOC116437
							0.97121	ZNF232
							0.97142	STARD8
							0.97163	MTERFD3
							0.97184	ARHGEF33
							0.97205	GFOD1
							0.97226	HSBP1
							0.97247	LOC254100
							0.97268	ASB2
							0.97289	LOC645332
							0.97310	C12orf64
							0.97331	CAPS
							0.97352	C12orf64
							0.97373	BCL7C
							0.97394	RGN
							0.97415	MRPL38
							0.97436	RPS15
							0.97457	CCDC159
							0.97478	SART1
							0.97499	TDRG1
							0.97520	LOC729770
							0.97542	RCVRN
							0.97563	ZNF766
							0.97584	LOC439911
							0.97605	TRMT1
							0.97626	ZNF534
							0.97647	CHRM4
							0.97668	TRIM29
							0.97689	ADM
							0.97710	FTHL3
							0.97731	MTSS1L
							0.97752	POU3F1
							0.97773	LOC100133211

0.97794	NR5A2
0.97815	WBSCR17
0.97836	KIAA1731
0.97857	PENK
0.97878	CNPY2
0.97899	LOC100288839
0.97920	SNAI3
0.97941	ZMYND10
0.97962	LOC643072
0.97983	DGAT2
0.98004	ZNF506
0.98025	MXD4
0.98046	DRD2
0.98067	SH3GL1P1
0.98088	TAGLN
0.98109	ARNT2
0.98130	KRT8P12
0.98151	GTDC1
0.98172	ANKS6
0.98193	DKK2
0.98214	LOC344065
0.98235	C10orf4
0.98256	FAM162B
0.98277	ELFN1
0.98298	PDGFRB
0.98319	CRELD1
0.98340	ATP6V0A2
0.98361	RASSF3
0.98382	WDR85
0.98403	VSTM2B
0.98424	TMEM90A
0.98445	LOC100144602
0.98466	JOSD2
0.98487	CYTSB
0.98508	LOC727993
0.98529	MBNL2
0.98550	RLIM
0.98571	LOC149832
0.98592	TBC1D28
0.98613	SNORA64
0.98634	ICT1
0.98655	PMS2L4

							0.98676	C17orf89
							0.98697	TPM3
							0.98718	LOC553103
							0.98739	C3orf31
							0.98760	ZNF250
							0.98781	PLK5P
							0.98802	LOC100289589
							0.98823	OCIAD2
							0.98844	PRSS45
							0.98865	ZNF235
							0.98886	PSTK
							0.98907	USP27X
							0.98928	RASGEF1C
							0.98949	C12orf64
							0.98970	PIGP
							0.98991	CEBPD
							0.99012	FRMD6
							0.99033	C9orf130
							0.99054	ZNF441
							0.99075	LOC100093698
							0.99096	CBX1
							0.99117	IGSF9B
							0.99138	LOC100131000
							0.99159	LOC100287008
							0.99181	LOC646719
							0.99202	LOC146336
							0.99223	ITGAD
							0.99244	LOC401233
							0.99265	C13orf26
							0.99286	MED26
							0.99307	TAC1
							0.99328	WNT7A
							0.99349	CTRB1
							0.99370	ZNF157
							0.99391	IRF5
							0.99412	MFI2
							0.99433	PCNA
							0.99454	BCDIN3D
							0.99475	BAK1
							0.99496	ZNF671
							0.99517	DACH1
							0.99538	LOC158402

							0.99559	CYP11A1
							0.99580	GK5
							0.99601	ARID3A
							0.99622	FLJ33360
							0.99643	NYNRIN
							0.99664	LOC644246
							0.99685	LOC100292996
							0.99706	ZNF34
							0.99727	SHISA8
							0.99748	ZNF749
							0.99769	KCNJ9
							0.99790	DSCR9
							0.99811	TTC3L
							0.99832	LOC100288316
							0.99853	CEP192
							0.99874	ZNF439
							0.99895	TNFRSF10B
							0.99916	ONECUT2
							0.99937	NCKAP5L
							0.99958	RPL10L
							0.99979	ZNF518B

b. TCTX

Temporal cortex																			
**Lightyellow module**		**Cyan module**		**Green module**		**Lightcyan module**		**Darkturquoise module**		**Purple module**		**Grey60 module**		**Midnightblue module**		**Pink module**		**Magenta module**	
**1-quantile**	**Gene**	**1-quantile**	**Gene**	**1-quantile**	**Gene**	**1-quantile**	**Gene**	**1-quantile**	**Gene**	**1-quantile**	**Gene**	**1-quantile**	**Gene**	**1-quantile**	**Gene**	**1-quantile**	**Gene**	**1-quantile**	**Gene**
0.00000	HCFC1	0.00000	RUSC2	0.00000	RAB3GAP2	0	NCKAP1L	0.00000	LOC100131015	0.00000	CAND1	0.00	SYT17	0.00000	SMCHD1	0.00000	YIPF7	0	C9orf45
0.00476	ATN1	0.00362	POLDIP2	0.00069	SCAMP1	0.004	PTPRC	0.00610	TRIM33	0.00120	NUDT21	0.00	PPP4R4	0.00373	SON	0.00034	HFM1	0.001	SCN1B
0.00952	MLL2	0.00725	PPP2R1A	0.00139	POLR2B	0.008	DOCK8	0.01220	TMEM106B	0.00241	AASDHPPT	0.01	PLXNC1	0.00746	SDCCAG1	0.00067	ENPP3	0.002	ANK1
0.01429	MEGF8	0.01087	LTBP3	0.00208	USO1	0.012	DOCK2	0.01829	UBA3	0.00361	USP7	0.01	NECAB2	0.01119	MON2	0.00101	CCDC18	0.003	SCRT2
0.01905	ABR	0.01449	NRSN2	0.00278	PJA2	0.016	APBB1IP	0.02439	TBCK	0.00482	IARS	0.02	PRKCD	0.01493	PIK3C3	0.00134	OR14A2	0.004	PVALB
0.02381	CIC	0.01812	C16orf5	0.00347	DDX1	0.02	TBXAS1	0.03049	DCTN4	0.00602	UBQLN1	0.02	C1orf95	0.01866	DDX17	0.00168	LOC199899	0.005	KCNAB3
0.02857	NLGN2	0.02174	GSK3A	0.00417	PSMD1	0.024	FYB	0.03659	ZMYM4	0.00723	ATRN	0.03	C1orf95	0.02239	HELZ	0.00201	ZNF165	0.006	ASB13
0.03333	ATXN2L	0.02536	OTUB1	0.00486	HEATR5B	0.028	C7orf58	0.04268	X2512330	0.00843	ITFG1	0.03	NPTXR	0.02612	DENND4A	0.00235	NCAPG2	0.007	PANX2
0.03810	COBRA1	0.02899	SLC25A11	0.00556	UBR3	0.032	CD68	0.04878	TOP2B	0.00964	DNM1L	0.04	WDR52	0.02985	TRPM7	0.00269	CLCA3P	0.008	HAPLN4
0.04286	ZBTB4	0.03261	ABCA3	0.00625	KPNA1	0.036	CYBB	0.05488	SEC63	0.01084	ACSL4	0.04	NHSL2	0.03358	LONP2	0.00302	HFM1	0.009	SYT2
0.04762	HGS	0.03623	VPS4A	0.00695	OPA1	0.04	ADAM28	0.06098	UBE2J1	0.01205	CKAP5	0.05	CPNE6	0.03731	WHSC1L1	0.00336	C8A	0.01	RAB37
0.05238	C11orf2	0.03986	EDC4	0.00764	GSK3B	0.044	ABCC4	0.06707	MRPL19	0.01325	RTF1	0.05	MAPK1	0.04104	RBM26	0.00369	NPVF	0.011	KCNC3
0.05714	EPN1	0.04348	CARM1	0.00834	X2801608	0.048	IGSF6	0.07317	ANKRD17	0.01446	WDR7	0.05	GLRA2	0.04478	ZNF207	0.00403	HMCN1	0.012	ABCG4
0.06190	FKBP8	0.04710	GRAMD1A	0.00903	ZYG11B	0.052	GPR34	0.07927	HOOK3	0.01566	BTRC	0.06	PRKCG	0.04851	WAC	0.00436	H2AFZ	0.013	C3orf57

0.06667	BBS1	0.05072	CLPTM1	0.00973	HSPA4	0.056	ITGAM	0.08537	HP1BP3	0.01687	USP9X	0.06	HPCAL4	0.05224	JMJD1C	0.00470	GBP5	0.014	KCNC1
0.07143	ZC3H7B	0.05435	TUBG2	0.01042	GRSF1	0.06	AIF1	0.09146	XPO7	0.01807	ATP6V1D	0.07	FAM65B	0.05597	IFNAR1	0.00504	ADCY10	0.015	SEMA7A
0.07619	MLLT1	0.05797	NFE2L1	0.01112	ANKMY2	0.064	LCP1	0.09756	UGGT1	0.01928	UHRF1BP1L	0.07	KIAA1239	0.05970	ZNF507	0.00537	OR6C74	0.016	GABRD
0.08095	ZNF358	0.06159	FARSA	0.01181	WDR47	0.068	C3AR1	0.10366	X2421271	0.02048	ATP6V0A1	0.08	NPTX2	0.06343	SETX	0.00571	IRX2	0.017	STAC2
0.08571	PRR12	0.06522	MEN1	0.01251	LRPPRC	0.072	FGL2	0.10976	PUM2	0.02169	PAFAH1B1	0.08	KCTD4	0.06716	SAPS3	0.00604	EZH2	0.018	MCF2
0.09048	MARK2	0.06884	KIAA0652	0.01320	DLD	0.076	HAVCR2	0.11585	SSR1	0.02289	MYO9A	0.09	EFNB3	0.07090	ZFP90	0.00638	LOC285423	0.019	STRBP
0.09524	RPTOR	0.07246	CAPN1	0.01390	RPP14	0.08	ALOX5AP	0.12195	RAB10	0.02410	EIF2AK4	0.09	CD24	0.07463	C14orf118	0.00671	C1orf168	0.02	VAMP1
0.10000	CREBBP	0.07609	ACP2	0.01459	LMBR1	0.084	SERPINA1	0.12805	ASH1L	0.02530	RHOT1	0.10	SSTR1	0.07836	BRWD1	0.00705	CYP39A1	0.021	SLC45A4
0.10476	SUPT5H	0.07971	CDK16	0.01529	NUP155	0.088	LPAR6	0.13415	NCOA1	0.02651	CLTC	0.10	LPPR3	0.08209	KIAA1012	0.00739	KYNU	0.022	RET
0.10952	GPS2	0.08333	MRPS2	0.01598	COPA	0.092	PIK3AP1	0.14024	SMARCAD1	0.02771	RAB11A	0.10	GRIN3A	0.08582	CHD8	0.00772	STK31	0.023	FGF9
0.11429	FAM89B	0.08696	CAMTA2	0.01668	COPS6	0.096	ADORA3	0.14634	CSDE1	0.02892	IREB2	0.11	MBNL2	0.08955	CPNE1	0.00806	GNAT3	0.024	TBC1D30
0.11905	MAP3K10	0.09058	DEDD2	0.01737	EPT1	0.1	P2RY13	0.15244	DNAJC13	0.03012	STAM	0.11	IL34	0.09328	STRN3	0.00839	ATRIP	0.025	ZMAT4
0.12381	CHERP	0.09420	POU6F1	0.01807	SLC25A36	0.104	PTPN6	0.15854	EIF2AK1	0.03133	SCYL2	0.12	CELF5	0.09701	TAF2	0.00873	TMPRSS15	0.026	KLC2
0.12857	FBXW5	0.09783	GGA3	0.01876	ENPP5	0.108	BLNK	0.16463	SNX13	0.03253	FAM160B1	0.12	C1QL3	0.10075	VEZT	0.00906	SLFN13	0.027	CD99L2
0.13333	RAB1B	0.10145	TM9SF4	0.01946	PDCD6IP	0.112	VAMP8	0.17073	PDS5A	0.03373	NOLC1	0.13	KIAA1644	0.10448	INO80	0.00940	LOC100289954	0.028	NEFH
0.13810	GNA11	0.10507	COPS7A	0.02015	DKFZp434H1419	0.116	IL13RA1	0.17683	VPS45	0.03494	UBE2W	0.13	MAN1A1	0.10821	RB1	0.00973	GVIN1	0.029	SCN4B
0.14286	KIAA0664	0.10870	LPHN1	0.02085	ORC4L	0.12	SYK	0.18293	SLC30A9	0.03614	CHM	0.14	PCDH19	0.11194	SFRS2	0.01007	CCDC73	0.03	STS
0.14762	NPRL3	0.11232	TCF25	0.02154	DHX36	0.124	SLCO2B1	0.18902	RPRD2	0.03735	TMEM85	0.14	ANKRD6	0.11567	C10orf18	0.01041	CCL4	0.031	TTC39B
0.15238	DTX3	0.11594	PSMF1	0.02224	LOC100329109	0.128	HCLS1	0.19512	ZRANB2	0.03855	KRAS	0.15	SCN3B	0.11940	FNDC3A	0.01074	CLEC1B	0.032	AGPAT9
0.15714	DLGAP4	0.11957	UNC119	0.02293	VTA1	0.132	TREM2	0.20122	UBXN2B	0.03976	ARFGEF1	0.15	PTCHD1	0.12313	C14orf135	0.01108	ABCA12	0.033	TTBK2
0.16190	FAM65A	0.12319	MRPL4	0.02363	G3BP2	0.136	ATP8B4	0.20732	RBM27	0.04096	CLCN4	0.15	SEMA4A	0.12687	ADRB1	0.01141	KIAA0087	0.034	TSPAN9
0.16667	KHSRP	0.12681	YIF1B	0.02432	MAPK9	0.14	TMEM156	0.21341	GPBP1	0.04217	OCRL	0.16	PKIA	0.13060	STK4	0.01175	SLC26A5	0.035	PNKD
0.17143	MGRN1	0.13043	KAT5	0.02502	PIK3CA	0.144	CSF1R	0.21951	EPC2	0.04337	PDK3	0.16	DOC2A	0.13433	NCBP1	0.01208	UGT2A1	0.036	CHGA
0.17619	TSC2	0.13406	ERCC1	0.02571	DZIP3	0.148	CYTH4	0.22561	MAN1A2	0.04458	MYCBP2	0.17	MEIS3	0.13806	RBBP6	0.01242	ZNF599	0.037	GRAMD1B
0.18095	CPSF7	0.13768	CLEC16A	0.02641	ERLEC1	0.152	PLCB2	0.23171	XPO1	0.04578	AP1G1	0.17	CDH4	0.14179	GSPT1	0.01276	SCML4	0.038	C16orf52
0.18571	SF3A1	0.14130	SLC23A2	0.02710	PPP2CA	0.156	LST1	0.23780	SNRNP200	0.04699	ATG2B	0.18	TP53I11	0.14552	KIAA1432	0.01309	CLCA2	0.039	ENTPD4
0.19048	USP20	0.14493	PCSK1N	0.02780	BRD8	0.16	TRIM38	0.24390	DHX9	0.04819	C12orf4	0.18	CRIP2	0.14925	NFX1	0.01343	MS4A14	0.04	ESRRG
0.19524	SYMPK	0.14855	NCRNA00094	0.02849	GNPDA2	0.164	SPTLC3	0.25000	MAP3K7	0.04940	EFTUD2	0.19	LOC100128320	0.15299	ZNF484	0.01376	ZNF28	0.041	SCN1A
0.20000	ZNF384	0.15217	RNF26	0.02919	COPB2	0.168	HPGDS	0.25610	WDR26	0.05060	SEC23A	0.19	PID1	0.15672	OGT	0.01410	TARP	0.042	IGFBP2
0.20476	TESK1	0.15580	SLC22A17	0.02988	SACM1L	0.172	SP100	0.26220	EXOC6B	0.05181	PSMA1	0.20	LOC286002	0.16045	ZMYM2	0.01443	HELLS	0.043	RCAN2
0.20952	PRR12	0.15942	LDOC1	0.03058	DYNC1LI1	0.176	TYROBP	0.26829	SOS1	0.05301	ATP6V1E1	0.20	RAB27B	0.16418	NCOR1	0.01477	ABCB5	0.044	KDM1A
0.21429	KCTD13	0.16304	FAM168A	0.03127	NRD1	0.18	ITPRIPL2	0.27439	SERINC1	0.05422	CUL2	0.20	SMAD3	0.16791	PAPOLA	0.01511	PIK3C2G	0.045	CERK
0.21905	TAOK2	0.16667	LOC284939	0.03197	METAP1	0.184	C1QB	0.28049	CDV3	0.05542	CDC123	0.21	DOK6	0.17164	C13orf23	0.01544	POLE2	0.046	FBXO33
0.22381	ZBTB45	0.17029	UBL7	0.03266	SNORA20	0.188	CARD8	0.28659	ABI2	0.05663	ARIH1	0.21	KCNA3	0.17537	KIAA1429	0.01578	UOX	0.047	KCNS1
0.22857	WBP2	0.17391	VAC14	0.03336	PIK3CB	0.192	PARVG	0.29268	FAM172A	0.05783	YME1L1	0.22	TLE1	0.17910	FAM48A	0.01611	HOXA9	0.048	RNF157
0.23333	ATP13A1	0.17754	NR1H2	0.03405	SLC2A12	0.196	CXCL16	0.29878	DCTN6	0.05904	CNOT7	0.22	LRRC2	0.18284	MED13L	0.01645	AFP	0.049	FNDC5
0.23810	KDELR1	0.18116	ADRM1	0.03475	CCT5	0.2	CSF3R	0.30488	KCTD20	0.06024	RALGAPB	0.23	GABRA5	0.18657	C9orf102	0.01678	ESCO2	0.05	CCDC136
0.24286	KATNB1	0.18478	CTSA	0.03544	SLC25A40	0.204	NFATC2	0.31098	SEC24B	0.06145	PSMA4	0.23	SYTL5	0.19030	FMR1	0.01712	GPR141	0.051	PCP4
0.24762	CES2	0.18841	SHMT2	0.03614	SNX27	0.208	CECR1	0.31707	HIAT1	0.06265	RPP30	0.24	ANO3	0.19403	BTAF1	0.01746	PLK4	0.052	KCNA1
0.25238	FAM195B	0.19203	CCDC124	0.03683	TNPO3	0.212	LAPTM5	0.32317	C5orf51	0.06386	ARL1	0.24	ATP2B4	0.19776	MPHOSPH9	0.01779	AQPEP	0.053	ANKRD34C
0.25714	EP300	0.19565	AP2A1	0.03753	PREPL	0.216	LY86	0.32927	FBXL5	0.06506	PSME3	0.25	ELK1	0.20149	CCNT1	0.01813	BANK1	0.054	TMEM145

0.26190	FUT11	0.19928	TRAPPC9	0.03822	C4orf41	0.22	SLC2A5	0.33537	KPNA5	0.06627	PRKDC	0.25	FLJ44635	0.20522	RABEP1	0.01846	TMEM99	0.055	ATP2B2
0.26667	SH2B1	0.20290	MEGF8	0.03892	CLASP1	0.224	CD84	0.34146	CSNK1G3	0.06747	MTMR7	0.25	TMSB10	0.20896	NUP98	0.01880	PTPN22	0.056	EPB41L1
0.27143	ILF3	0.20652	DBNDD1	0.03961	BTF3L4	0.228	P2RY12	0.34756	SENP2	0.06867	PPME1	0.26	LY6H	0.21269	SETD3	0.01913	HERC5	0.057	OSBP2
0.27619	PNPLA6	0.21014	AP1B1	0.04031	ELP3	0.232	RUNX1	0.35366	LOC731139	0.06988	RC3H2	0.26	RRAGB	0.21642	PARN	0.01947	GRAP2	0.058	LYPD5
0.28095	LMTK3	0.21377	YPEL3	0.04100	GORASP2	0.236	TLR10	0.35976	EIF2AK2	0.07108	DYRK1A	0.27	DIAPH2	0.22015	PHKB	0.01981	MSH4	0.059	EIF5A2
0.28571	HNRNPUL1	0.21739	HYOU1	0.04170	FBXO38	0.24	EVI2B	0.36585	TCEB3	0.07229	RAB12	0.27	RAP2B	0.22388	PRO0628	0.02014	RBL1	0.06	LOC285878
0.29048	R3HDM2	0.22101	CBX7	0.04239	VAMP4	0.244	LAIR1	0.37195	NUCKS1	0.07349	LTA4H	0.28	LOC100130713	0.22761	LCOR	0.02048	WHAMML2	0.061	SLC16A7
0.29524	SMARCC2	0.22464	PIP5K1C	0.04309	PMPCB	0.248	FCER1G	0.37805	BCLAF1	0.07470	SPIN1	0.28	RBP4	0.23134	MAPKAPK5	0.02081	AFAP1L1	0.062	SIAE
0.30000	SPRN	0.22826	TRAP1	0.04378	UBR3	0.252	C3	0.38415	STAM2	0.07590	TERF2IP	0.29	CDH8	0.23507	AEBP2	0.02115	HESX1	0.063	RASD2
0.30476	TRAF7	0.23188	PRAF2	0.04448	MOBKL3	0.256	LILRB4	0.39024	PIKFYVE	0.07711	HSPA13	0.29	IL12RB2	0.23881	SUZ12	0.02148	PKHD1L1	0.064	MACF1
0.30952	LDOC1L	0.23551	C9orf25	0.04517	BBS7	0.26	SELPLG	0.39634	CLINT1	0.07831	DENND5B	0.30	MMD	0.24254	MORC3	0.02182	VCAM1	0.065	DCBLD2
0.31429	MAPT	0.23913	ABHD12	0.04587	PIK3R4	0.264	LPCAT2	0.40244	TBL1XR1	0.07952	UBR1	0.30	HRASLS5	0.24627	YLPM1	0.02216	OR5H14	0.066	KIAA0802
0.31905	NACC1	0.24275	KIAA1543	0.04656	C5orf22	0.268	CLEC9A	0.40854	JAKMIP2	0.08072	NAPG	0.30	PACRG	0.25000	KIAA0586	0.02249	DLEU2	0.067	DCUN1D2
0.32381	MBD1	0.24638	SIK2	0.04726	BMPR2	0.272	CTSS	0.41463	ATF6	0.08193	SEL1L	0.31	SLIT3	0.25373	SNORD5	0.02283	ITGBL1	0.068	THAP10
0.32857	SRCAP	0.25000	TUBGCP2	0.04795	TMEM30A	0.276	CASP4	0.42073	GOLGB1	0.08313	ARCN1	0.31	LRRC57	0.25746	TNRC6B	0.02316	HSD17B13	0.069	D4S234E
0.33333	C19orf61	0.25362	FXR2	0.04864	DNAJC16	0.28	CD37	0.42683	MAP4K3	0.08434	B4GALT5	0.32	CNIH2	0.26119	TM9SF3	0.02350	DKFZp686F0839	0.07	TAF4B
0.33810	POLR2A	0.25725	NDUFB10	0.04934	NOL7	0.284	MNDA	0.43293	ACTR3	0.08554	KPNA3	0.32	NUDT11	0.26493	C10orf76	0.02383	C18orf34	0.071	ANKRD29
0.34286	BSG	0.26087	TNFAIP1	0.05003	ASH2L	0.288	HLA-DMA	0.43902	OCIAD1	0.08675	SRP68	0.33	GNG2	0.26866	C16orf61	0.02417	MAGEB5	0.072	SYT3
0.34762	GTPBP1	0.26449	EIF4ENIF1	0.05073	MTMR9	0.292	GPR65	0.44512	SSBP2	0.08795	NF1	0.33	LOC730045	0.27239	ATXN2	0.02450	MYH15	0.073	PPTC7
0.35238	PELP1	0.26812	CACNG7	0.05142	CUL1	0.296	APOC2	0.45122	TBC1D5	0.08916	AKAP11	0.34	ST6GALNAC5	0.27612	SFRS15	0.02484	NHLH2	0.074	OSBPL3
0.35714	ABHD8	0.27174	CTSD	0.05212	DNAJC18	0.3	ALOX5	0.45732	RBM16	0.09036	SUPT4H1	0.34	PNCK	0.27985	KIAA0196	0.02518	C14orf53	0.075	CNNM1
0.36190	VPS53	0.27536	ALKBH6	0.05281	KIAA1109	0.304	TLR7	0.46341	KIAA1109	0.09157	ATL1	0.35	ARHGAP6	0.28358	CG030	0.02551	NCAPH	0.076	ARL4C
0.36667	RNF214	0.27899	SLC35E1	0.05351	RASA1	0.308	LOC100233209	0.46951	APPL1	0.09277	COPS2	0.35	PTPRR	0.28731	EIF3A	0.02585	BTN2A2	0.077	PELI3
0.37143	RNF167	0.28261	SEC16A	0.05420	FARSB	0.312	TLR8	0.47561	LYSMD3	0.09398	VPS35	0.35	GABRA5	0.29104	ZNF570	0.02618	C7orf10	0.078	SLC4A8
0.37619	SIPA1L3	0.28623	POLR2E	0.05490	KPNA6	0.316	ITGAX	0.48171	PFN2	0.09518	DPM1	0.36	LOC285780	0.29478	MLL	0.02652	BRDT	0.079	EXTL2
0.38095	ULK1	0.28986	IRGQ	0.05559	LNPEP	0.32	LOC387646	0.48780	MAP3K2	0.09639	FBXL20	0.36	GABRA3	0.29851	CCPG1	0.02685	FRAT1	0.08	ASAM
0.38571	SLC25A39	0.29348	DAGLA	0.05629	BNIP3L	0.324	TSPAN6	0.49390	MTX2	0.09759	EIF5	0.37	CNR1	0.30224	ANKRD12	0.02719	UBQLN3	0.081	GLS2
0.39048	CIZ1	0.29710	RABGGTA	0.05698	MTDH	0.328	ARHGDIB	0.50000	FOXJ3	0.09880	PDHX	0.37	FREM3	0.30597	DDHD1	0.02753	CENPE	0.082	VAV3
0.39524	GAS2L1	0.30072	SYNGR1	0.05768	GARS	0.332	IRAK3	0.50610	CRIPT	0.10000	ATP6AP2	0.38	GABRQ	0.30970	FUS	0.02786	AOX1	0.083	FMN1
0.40000	SAMD1	0.30435	TUBG1	0.05837	IPO11	0.336	TEP1	0.51220	AGFG1	0.10120	SLC9A6	0.38	CNGB1	0.31343	FLJ44342	0.02820	MCOLN2	0.084	FAM135B
0.40476	NMT1	0.30797	PSMD3	0.05907	RANBP2	0.34	LPAR5	0.51829	MED23	0.10241	NDUFA9	0.39	COG1	0.31716	ARID4A	0.02853	TMPRSS11BNL	0.085	GPLD1
0.40952	EDF1	0.31159	EPS15L1	0.05976	ATP6V1C1	0.344	CLEC7A	0.52439	SR140	0.10361	CAPRIN1	0.39	KCNA4	0.32090	NBN	0.02887	ACCN5	0.086	PDE4A
0.41429	CABIN1	0.31522	TBC1D25	0.06046	NUP153	0.348	SPI1	0.53049	RC3H1	0.10482	CDS2	0.40	KLK7	0.32463	GARNL1	0.02920	LOC100129036	0.087	NCOA3
0.41905	U2AF2	0.31884	PPP2R5B	0.06115	FAM73A	0.352	C1orf162	0.53659	AKT3	0.10602	DLAT	0.40	FXYD6	0.32836	PPHLN1	0.02954	LEKR1	0.088	CLEC2L
0.42381	CDC34	0.32246	ALDOA	0.06185	UBE3C	0.356	C1QC	0.54268	PARP1	0.10723	PRKAG1	0.40	TMEM158	0.33209	TNKS2	0.02988	LRRC17	0.089	ADPRHL1
0.42857	UNK	0.32609	PORCN	0.06254	UBE2K	0.36	HLA-DMB	0.54878	CLOCK	0.10843	DCTN5	0.41	FKBP11	0.33582	BIVM	0.03021	FMO1	0.09	ARNTL
0.43333	ELAVL3	0.32971	MLF2	0.06324	FIG4	0.364	LHFPL2	0.55488	RNF146	0.10964	C9orf5	0.41	PGRMC1	0.33955	MYEF2	0.03055	GLYATL1P2	0.091	LGI3
0.43810	EEF2	0.33333	RNASEK	0.06393	XPR1	0.368	TFEC	0.56098	CHL1	0.11084	GHITM	0.42	PLB1	0.34328	USP16	0.03088	C12orf50	0.092	CITED2
0.44286	MPP2	0.33696	CX3CL1	0.06463	ARL6IP5	0.372	AOAH	0.56707	PSMD14	0.11205	CSE1L	0.42	ICAM5	0.34701	HIPK3	0.03122	C12orf56	0.093	ATG4D
0.44762	NCOR2	0.34058	PSAP	0.06532	BAG4	0.376	MYO1F	0.57317	RAB7A	0.11325	CISD1	0.43	LOC285954	0.35075	ZNF248	0.03155	FLJ42393	0.094	C14orf83
0.45238	PDK2	0.34420	GPR137	0.06602	COL4A3BP	0.38	B4GALT1	0.57927	ZFR	0.11446	MBTPS1	0.43	ARMCX2	0.35448	RABGAP1	0.03189	RIMBP3B	0.095	NECAB3
0.45714	BRD4	0.34783	BSCL2	0.06671	AVL9	0.384	FCGR3A	0.58537	UBN2	0.11566	ANXA7	0.44	RNF150	0.35821	CNOT2	0.03223	C1orf125	0.096	SLC38A1

0.46190	HUWE1	0.35145	NUCB1	0.06741	SEC31A	0.388	IKZF1	0.59146	CPEB4	0.11687	APP	0.44	EPB41L4B	0.36194	GNS	0.03256	C19orf41	0.097	CDS1
0.46667	C19orf6	0.35507	AES	0.06810	GPR155	0.392	ITGB2	0.59756	PDHB	0.11807	EXT2	0.45	DNAL4	0.36567	EEA1	0.03290	LOC339894	0.098	FCHSD1
0.47143	ZCCHC14	0.35870	PNPO	0.06880	KIAA1109	0.396	CPVL	0.60366	LRRC40	0.11928	CCDC6	0.45	ADAMTS3	0.36940	SLC25A28	0.03323	ANKAR	0.099	TBC1D4
0.47619	TMEM8B	0.36232	GDI1	0.06949	SRD5A1	0.4	MS4A4A	0.60976	RNF103	0.12048	C17orf51	0.45	RASAL1	0.37313	C17orf95	0.03357	SCN11A	0.1	HS6ST2
0.48095	PCNXL3	0.36594	KNDC1	0.07019	ZMAT3	0.404	MYD88	0.61585	PUM1	0.12169	ZDHHC17	0.46	NAV2	0.37687	ROD1	0.03390	AMICA1	0.101	RASGRF1
0.48571	RNF10	0.36957	GPX4	0.07088	KLHDC10	0.408	UCP2	0.62195	TMED4	0.12289	UBE4A	0.46	PLXNA1	0.38060	BRWD3	0.03424	ERAP2	0.102	ST8SIA1
0.49048	C11orf68	0.37319	ZFYVE27	0.07158	ZC3H15	0.412	C17orf87	0.62805	SDHC	0.12410	SBNO1	0.47	MRAS	0.38433	ZC3H7A	0.03458	BEND2	0.103	SLC25A12
0.49524	C2CD2L	0.37681	PTPRS	0.07227	MAPK14	0.416	C1QA	0.63415	POLR2K	0.12530	CAMSAP1	0.47	FOXRED2	0.38806	NAA25	0.03491	FLJ46321	0.104	SV2C
0.50000	UNKL	0.38043	ARHGEF17	0.07297	CUL3	0.42	SQRDL	0.64024	CCNT2	0.12651	CMAS	0.48	C9orf103	0.39179	NUP214	0.03525	SPON2	0.105	SLC38A2
0.50476	SEC16A	0.38406	MAP4K2	0.07366	TTC37	0.424	FCGR2A	0.64634	LMBRD1	0.12771	CDK17	0.48	NTRK3	0.39552	DDX6	0.03558	DNAH14	0.106	PPARGC1A
0.50952	X3936984	0.38768	X3819522	0.07436	ZBTB11	0.428	CD86	0.65244	ECHDC1	0.12892	ANKRD46	0.49	GALNTL6	0.39925	ADNP	0.03592	HFE	0.107	INPP5J
0.51429	MGC2752	0.39130	ELOF1	0.07505	NCKAP1	0.432	SAMSN1	0.65854	MFSD8	0.13012	OSBPL8	0.49	TMTC1	0.40299	MED14	0.03625	LOC100124692	0.108	ANKRD16
0.51905	NAB2	0.39493	MAPK8IP3	0.07575	MARK1	0.436	RNF135	0.66463	NRAS	0.13133	RPAP3	0.50	PTPN3	0.40672	WAPAL	0.03659	CRTAM	0.109	LRRC4
0.52381	BCAP31	0.39855	PNPLA2	0.07644	MAPKSP1	0.44	ADAP2	0.67073	INTS10	0.13253	PSMD5	0.50	ASXL3	0.41045	SEC16A	0.03693	LOC100128317	0.11	TUBGCP5
0.52857	MKNK2	0.40217	VPS28	0.07714	CASD1	0.444	MS4A6A	0.67683	AGL	0.13373	CCT2	0.50	LOC284395	0.41418	SECISBP2	0.03726	GPR128	0.111	PRKACG
0.53333	AKT1	0.40580	TTC7B	0.07783	FBXO28	0.448	VAV1	0.68293	IPP	0.13494	CNOT1	0.51	C2orf85	0.41791	COG6	0.03760	LOC100289181	0.112	RSPO2
0.53810	NR1D1	0.40942	AMFR	0.07853	LNP1	0.452	CSF2RB	0.68902	KIAA1109	0.13614	TRMT6	0.51	SLC8A2	0.42164	TCF4	0.03793	ADAM7	0.113	GPR158
0.54286	C17orf39	0.41304	ANKRD13D	0.07922	TOPBP1	0.456	MMP14	0.69512	UBXN7	0.13735	GBF1	0.52	ERLIN1	0.42537	CDK12	0.03827	SLC35F4	0.114	TRMT2B
0.54762	C18orf49	0.41667	XAB2	0.07992	EPRS	0.46	LILRA1	0.70122	DOPEY1	0.13855	SAMM50	0.52	ANKRD50	0.42910	RBM41	0.03860	USP44	0.115	PRPS2
0.55238	HDAC5	0.42029	BRSK2	0.08061	KIFAP3	0.464	DHRS9	0.70732	AFF4	0.13976	CCNA1	0.53	CCBE1	0.43284	ZC3H14	0.03894	NEDD4	0.116	DKFZp686O2416
0.55714	SETD1B	0.42391	RAD23A	0.08131	UBE2Q1	0.468	B3GNT5	0.71341	DHFR	0.14096	C9orf72	0.53	RUNX1T1	0.43657	ARHGAP21	0.03927	C14orf25	0.117	CDH7
0.56190	FAM19A5	0.42754	SSSCA1	0.08200	SLC4A1AP	0.472	CTSC	0.71951	ACADM	0.14217	DERL1	0.54	DLG2	0.44030	EXOSC1	0.03961	TRPC6	0.118	LOC400019
0.56667	AGPAT3	0.43116	GNAZ	0.08270	TIPRL	0.476	IRF8	0.72561	CNOT6	0.14337	TMTC3	0.54	CPNE5	0.44403	C14orf101	0.03995	KRTAP10-7	0.119	LOC554202
0.57143	C9orf69	0.43478	PPRC1	0.08339	KIF3A	0.48	PYGL	0.73171	GLO1	0.14458	LOC100131102	0.55	ZCCHC17	0.44776	DERL2	0.04028	GPR18	0.12	HS3ST1
0.57619	AMBRA1	0.43841	CISD3	0.08409	C6orf211	0.484	ARHGAP15	0.73780	LOC653160	0.14578	LOC401504	0.55	CCDC3	0.45149	C16orf62	0.04062	GIPC2	0.121	PKNOX2
0.58095	MNT	0.44203	N4BP1	0.08478	NNT	0.488	CIITA	0.74390	SEC24A	0.14699	LCMT1	0.55	RAB36	0.45522	PIGU	0.04095	LOC440970	0.122	RORA
0.58571	USP2	0.44565	SBK1	0.08548	ELOVL4	0.492	VSIG4	0.75000	TICAM2	0.14819	USP15	0.56	AMIGO2	0.45896	SMCHD1	0.04129	RPLP0	0.123	SLC25A25
0.59048	SEZ6	0.44928	PPPDE2	0.08617	NBAS	0.496	PLEK	0.75610	C3orf17	0.14940	RPS6KA3	0.56	SOCS7	0.46269	COX16	0.04162	LHX8	0.124	SLC16A6
0.59524	CSNK1G2	0.45290	SNX32	0.08687	ATPAF1	0.5	INPP5D	0.76220	SAP130	0.15060	TSG101	0.57	PDE4C	0.46642	SAMD8	0.04196	LOC100294489	0.125	CAMTA1
0.60000	ERCC2	0.45652	WNK2	0.08756	SCFD2	0.504	CX3CR1	0.76829	USP38	0.15181	BTBD1	0.57	SYNPO	0.47015	ATP9B	0.04230	C11orf53	0.126	KCNJ3
0.60476	MED25	0.46014	GPR123	0.08826	KLHDC3	0.508	HLA-DRA	0.77439	RAB28	0.15301	WARS	0.58	KLHDC8B	0.47388	MRPL50	0.04263	C12orf33	0.127	DBC1
0.60952	PCIF1	0.46377	ADRBK1	0.08895	ATF2	0.512	PLCG2	0.78049	TMCC1	0.15422	SHOC2	0.58	OTUB2	0.47761	NCOA6	0.04297	MORC1	0.128	RRP12
0.61429	TRIM28	0.46739	CSNK1E	0.08965	CAB39	0.516	FCGR1A	0.78659	PEX1	0.15542	ACTR10	0.59	SRR	0.48134	TBC1D15	0.04330	DDX60L	0.129	X4019486
0.61905	USF2	0.47101	BRSK1	0.09034	SNX14	0.52	CD4	0.79268	WDFY1	0.15663	MRPL42	0.59	HTR1A	0.48507	METAP2	0.04364	BRCA1	0.13	ASAH2B
0.62381	ZNF385A	0.47464	RNF40	0.09104	TBC1D19	0.524	ARHGAP25	0.79878	SLAIN2	0.15783	USP32	0.60	TSHZ2	0.48881	HSP90B2P	0.04397	SYCP1	0.131	CSDC2
0.62857	SELM	0.47826	ZBTB7A	0.09173	DMXL1	0.528	PARP9	0.80488	HDDC2	0.15904	KLHDC2	0.60	ADRA1B	0.49254	CPSF6	0.04431	GABRR1	0.132	CABP1
0.63333	LRP3	0.48188	F8A1	0.09243	HARS	0.532	WDFY4	0.81098	SP4	0.16024	APPBP2	0.60	FREM3	0.49627	FUBP3	0.04465	FCER1A	0.133	CRTAC1
0.63810	FHL3	0.48551	TUBB4	0.09312	TMEM111	0.536	TLR5	0.81707	TANK	0.16145	ATXN10	0.61	SUMO3	0.50000	RIC3	0.04498	PIP5K1P1	0.134	RIMKLA
0.64286	NCAPH2	0.48913	PPP1CA	0.09382	ATP11B	0.54	TEK	0.82317	SMEK2	0.16265	PLAA	0.61	CETN2	0.50373	RSRC2	0.04532	SCML2	0.135	KLF9
0.64762	LOC92973	0.49275	MYBBP1A	0.09451	LGALS8	0.544	PZP	0.82927	ANKRD28	0.16386	SEH1L	0.62	CA4	0.50746	CUL4B	0.04565	FLJ43879	0.136	SLC39A14
0.65238	CASKIN1	0.49638	EMD	0.09521	TFG	0.548	RNASET2	0.83537	DAP3	0.16506	LOC100128608	0.62	AP2B1	0.51119	ARG2	0.04599	NCR3	0.137	P2RX6
0.65714	SNX12	0.50000	ASPHD1	0.09590	CCDC132	0.552	CD300A	0.84146	TMEM43	0.16627	TMX3	0.63	SLC22A10	0.51493	SPATA7	0.04632	LOC728095	0.138	PRR16

0.66190	BTBD2	0.50362	NDST2	0.09659	PLEKHM3	0.556	KIAA1274	0.84756	MPP6	0.16747	REPS2	0.63	ARL10	0.51866	EZH1	0.04666	DEFB112	0.139	MAGI3
0.66667	NOVA2	0.50725	PEX5	0.09729	WDR41	0.56	LIMS2	0.85366	LOC100288608	0.16867	RAB11FIP2	0.64	FAM110C	0.52239	ISCA2	0.04700	RBPMS	0.14	HYMAI
0.67143	IRS2	0.51087	GRN	0.09798	MEAF6	0.564	WDFY4	0.85976	AKD1	0.16988	RAB2B	0.64	PDYN	0.52612	TTC17	0.04733	SAGE1	0.141	ABCA10
0.67619	CHTF8	0.51449	TTLL12	0.09868	RAP1GDS1	0.568	PALMD	0.86585	UBE2D2	0.17108	CUL5	0.65	FREM3	0.52985	STX3	0.04767	DNAH8	0.142	SPTB
0.68095	ARFGAP2	0.51812	ERGIC3	0.09937	PIGK	0.572	ATP11C	0.87195	TATDN3	0.17229	PRMT5	0.65	GDA	0.53358	NCOA4	0.04800	GIMAP6	0.143	TSPAN9
0.68571	DUSP8	0.52174	HPS1	0.10007	PPT1	0.576	CECR7	0.87805	ANKRA2	0.17349	CSRNP2	0.65	LOC400456	0.53731	ALG13	0.04834	CD44	0.144	C11orf87
0.69048	XYLT2	0.52536	IL4I1	0.10076	KPNA4	0.58	HLA-DPA1	0.88415	PLRG1	0.17470	BTBD10	0.66	TRIM2	0.54104	CALM1	0.04867	LOC100131781	0.145	LRRC49
0.69524	FAM89B	0.52899	FURIN	0.10146	HERC3	0.584	DDX60	0.89024	RPL22	0.17590	ERAL1	0.66	SCPEP1	0.54478	BUB3	0.04901	C4orf49	0.146	KCNT1
0.70000	SF3B2	0.53261	ANKRD11	0.10215	UBA5	0.588	KLRC1	0.89634	IARS2	0.17711	AKAP10	0.67	DOK6	0.54851	ACAT1	0.04935	CEBPZ	0.147	LOC729082
0.70476	GTF2F1	0.53623	SFXN3	0.10285	KIAA1715	0.592	PRKX	0.90244	KIAA0947	0.17831	SERINC3	0.67	VAT1L	0.55224	SF1	0.04968	OR10K1	0.148	CNTNAP5
0.70952	RAB5B	0.53986	LRRC43	0.10354	LOC100130101	0.596	PVRL2	0.90854	CCNYL1	0.17952	ZNF25	0.68	NEUROD6	0.55597	TMEM55B	0.05002	DDC	0.149	SERGEF
0.71429	DACT3	0.54348	INPP5E	0.10424	EXOC8	0.6	SAMD9	0.91463	EVI5	0.18072	MAPK8	0.68	ASXL3	0.55970	CUL4A	0.05035	GPR109A	0.15	RILP
0.71905	NPDC1	0.54710	CLCN7	0.10493	BCAP29	0.604	PLD4	0.92073	DTNB	0.18193	CCNDBP1	0.69	WNT10B	0.56343	SGK269	0.05069	WEE1	0.151	RPP25
0.72381	RAB35	0.55072	LOC100292534	0.10563	DGUOK	0.608	SCIN	0.92683	PCDHB19P	0.18313	RANBP6	0.69	TIMM10	0.56716	BBS10	0.05102	FLJ16124	0.152	RIMS3
0.72857	GPS1	0.55435	WIPF2	0.10632	IPO9	0.612	IRAK3	0.93293	MRPS18B	0.18434	PDXDC1	0.70	PTCHD1	0.57090	EFHA1	0.05136	LOC730179	0.153	KCNB1
0.73333	BAT2	0.55797	NMRAL1	0.10702	AZI2	0.616	GBP1	0.93902	KLHL2	0.18554	PARP6	0.70	C14orf132	0.57463	TIAL1	0.05170	CNPY1	0.154	LPCAT4
0.73810	ACO2	0.56159	JMJD8	0.10771	SESN1	0.62	LYZ	0.94512	DEK	0.18675	KIAA0317	0.70	NISCH	0.57836	BCL2L13	0.05203	KIF20B	0.155	ADCY9
0.74286	GNPTG	0.56522	CORO2B	0.10841	VPS41	0.624	C12orf75	0.95122	KDM3A	0.18795	TIMM50	0.71	KCTD21	0.58209	GTF2H3	0.05237	PRSS42	0.156	GDPD1
0.74762	SETD1B	0.56884	PGS1	0.10910	LMBRD2	0.628	ZNF22	0.95732	SLC35A3	0.18916	CHIC1	0.71	TSHZ2	0.58582	MED1	0.05270	AVPR1A	0.157	RHBDL3
0.75238	SCAF1	0.57246	E2F4	0.10980	EIF4G3	0.632	EXOSC8	0.96341	OSGIN2	0.19036	C17orf81	0.72	MGC23284	0.58955	DDX23	0.05304	NCRNA00161	0.158	CCNB1IP1
0.75714	BLOC1S1	0.57609	X3418513	0.11049	PCMT1	0.636	FBLN2	0.96951	C4orf10	0.19157	POLR3A	0.72	TMEM200A	0.59328	DENR	0.05337	CFTR	0.159	NCKIPSD
0.76190	GGA1	0.57971	MED15	0.11119	ACAD9	0.64	HAUS4	0.97561	FAM53C	0.19277	RBM18	0.73	CCBE1	0.59701	AP3M1	0.05371	SRPX2	0.16	SGPP2
0.76667	LDB1	0.58333	POLDIP3	0.11188	DNAJC27	0.644	STEAP3	0.98171	USP21	0.19398	USP14	0.73	KCNG3	0.60075	CLPX	0.05404	CENPH	0.161	PANK1
0.77143	APBB1	0.58696	DPP7	0.11258	NDUFS2	0.648	SLC15A3	0.98780	ATXN7L1	0.19518	FAM179B	0.74	SLN	0.60448	RNF169	0.05438	LOC134466	0.162	ST8SIA5
0.77619	HIRA	0.59058	DPP9	0.11327	TMEM33	0.652	MPEG1	0.99390	PIAS3	0.19639	APEX1	0.74	PTPRF	0.60821	SMEK1	0.05472	GPR87	0.163	CCDC69
0.78095	TOM1L2	0.59420	C19orf28	0.11397	ARMC8	0.656	PGM5P1			0.19759	CSTF1	0.75	TMEM180	0.61194	RNF20	0.05505	ZSCAN16	0.164	CACNA1A
0.78571	LRFN3	0.59783	LRRK1	0.11466	SNX17	0.66	CASP1			0.19880	PSMC4	0.75	SSTR2	0.61567	EIF3H	0.05539	ASPN	0.165	SCRT1
0.79048	UPF1	0.60145	NCLN	0.11536	ZNF184	0.664	ALPK1			0.20000	CYB5B	0.75	NPY1R	0.61940	CTNND1	0.05572	MYO3B	0.166	C9orf150
0.79524	RNF31	0.60507	KCNN1	0.11605	C7orf60	0.668	BTK			0.20120	PDHA1	0.76	LSM4	0.62313	SLC39A6	0.05606	HMMR	0.167	C17orf75
0.80000	INO80E	0.60870	RBM10	0.11675	EDEM3	0.672	TLR6			0.20241	ZNF280B	0.76	VWA5A	0.62687	UBE2A	0.05639	RCC1	0.168	REEP2
0.80476	PDZD4	0.61232	RANBP3	0.11744	ANKRD39	0.676	CD74			0.20361	NEDD4L	0.77	LOC157562	0.63060	ZCCHC6	0.05673	NCRNA00236	0.169	KBTBD3
0.80952	KCNC1	0.61594	STX5	0.11814	SLC30A5	0.68	TMOD3			0.20482	ERO1L	0.77	ADAMTS8	0.63433	FOXN3	0.05707	ZNF99	0.17	KCNA2
0.81429	MGAT5B	0.61957	SLC7A5	0.11883	EXOC2	0.684	BAX			0.20602	NIPA2	0.78	FREM3	0.63806	SLK	0.05740	RPGRIP1	0.171	KCNK9
0.81905	SH2D3C	0.62319	DTX4	0.11953	RAB2A	0.688	DSE			0.20723	OSBP	0.78	ABCC12	0.64179	ZNF410	0.05774	C7orf66	0.172	ACVR1C
0.82381	NOL6	0.62681	TLE3	0.12022	KDM5B	0.692	SIGLEC8			0.20843	KIAA1279	0.79	PDZD2	0.64552	YEATS4	0.05807	CCT6P4	0.173	HR
0.82857	TBC1D10B	0.63043	UBA2	0.12092	SLC35A5	0.696	FLJ10038			0.20964	MORN4	0.79	RFX3	0.64925	ASAH1	0.05841	NBLA00301	0.174	IRS1
0.83333	ABAT	0.63406	SSNA1	0.12161	ATG5	0.7	FPR1			0.21084	ZDHHC21	0.80	OPTN	0.65299	ZNF566	0.05874	ITIH2	0.175	STRBP
0.83810	ASAP1	0.63768	DCAF5	0.12231	NRBP1	0.704	HMGN5			0.21205	SF3B3	0.80	MGAT4B	0.65672	HIF1A	0.05908	MIOX	0.176	CDC42EP3
0.84286	DGCR11	0.64130	TMEM184B	0.12300	MRPL30	0.708	SLC26A2			0.21325	CDC27	0.80	OCA2	0.66045	USP30	0.05942	THSD7B	0.177	LOC255167
0.84762	PC	0.64493	SNRPA	0.12370	GLMN	0.712	TAGAP			0.21446	EIF2S1	0.81	PPEF1	0.66418	TBC1D20	0.05975	AMDHD1	0.178	SPTB
0.85238	CCDC97	0.64855	CRKL	0.12439	SLC25A46	0.716	HIST1H2BF			0.21566	ZFAND1	0.81	GALNTL4	0.66791	SNX19	0.06009	ISL1	0.179	SLC43A2
0.85714	IRF2BP1	0.65217	INCENP	0.12509	RTCD1	0.72	KIAA1949			0.21687	PAPD5	0.82	ARNT2	0.67164	LOC100127980	0.06042	CDK15	0.18	LOC100128844

0.86190	PTOV1
0.86667	KCNH3
0.87143	SRRM2
0.87619	G6PD
0.88095	JUND
0.88571	FBXL19
0.89048	EDEM2
0.89524	DRAP1
0.90000	DDT
0.90476	DAZAP2
0.90952	OGFR
0.91429	RHBDL1
0.91905	GAL3ST3
0.92381	WWP2
0.92857	C12orf44
0.93333	COPE
0.93810	DPYSL4
0.94286	SSRP1
0.94762	NKAP
0.95238	C17orf80
0.95714	MMS19
0.96190	HEXDC
0.96667	GORASP1
0.97143	TMCO4
0.97619	UBAC1
0.98095	AKT1S1
0.98571	KLF13
0.99048	NDUFA13
0.99524	TEF

0.65580	KIAA1659
0.65942	C11orf51
0.66304	NEURL
0.66667	POLA2
0.67029	SMARCA4
0.67391	BCKDK
0.67754	SAPS2
0.68116	TFIP11
0.68478	EIF3G
0.68841	PRPF6
0.69203	PGAP3
0.69565	FOXK1
0.69928	OAZ2
0.70290	LPHN1
0.70652	AMZ2P1
0.71014	SPG7
0.71377	GLT25D1
0.71739	TINF2
0.72101	ATP5D
0.72464	OBFC1
0.72826	DRG2
0.73188	SIRPA
0.73551	GAA
0.73913	FAM100A
0.74275	H2AFX
0.74638	PRKCA
0.75000	AP3S2
0.75362	SMPD1
0.75725	LSMD1
0.76087	AATF
0.76449	EIF2C2
0.76812	KEAP1
0.77174	EGLN2
0.77536	DGCR14
0.77899	NKIRAS2
0.78261	ANKRD27
0.78623	SLC37A4
0.78986	SAT2
0.79348	TOM1L2
0.79710	CMTM4
0.80072	SH3BGRL3
0.80435	PES1

0.12578	MAPRE3
0.12648	SCP2
0.12717	TMEM184C
0.12787	GNB1
0.12856	CACNA2D1
0.12926	PPA2
0.12995	HISPPD1
0.13065	SENP5
0.13134	WDR82
0.13204	MTX3
0.13273	UGP2
0.13343	DCUN1D4
0.13412	FAM126B
0.13482	CDKL2
0.13551	PCDHB14
0.13621	C1orf128
0.13690	NCOA7
0.13760	FBXO11
0.13829	KIAA0090
0.13899	SIKE1
0.13968	PEX11B
0.14038	HSPC159
0.14107	ABHD10
0.14177	OXCT1
0.14246	PRKACB
0.14315	GMPS
0.14385	SLC16A14
0.14454	DSTYK
0.14524	RAB3GAP1
0.14593	UCHL5
0.14663	SUPT7L
0.14732	AP3M2
0.14802	AGK
0.14871	CLASP2
0.14941	MOCS2
0.15010	IMMT
0.15080	CLCN6
0.15149	IAH1
0.15219	LSG1
0.15288	CLCN3
0.15358	MSH2
0.15427	FAF2

0.724	ZFP36
0.728	GIMAP2
0.732	NCF2
0.736	FAM129B
0.74	IL4R
0.744	ZNF578
0.748	PTAFR
0.752	CIDEB
0.756	OAS1
0.76	SLC29A3
0.764	TWSG1
0.768	KCTD12
0.772	RNASE6
0.776	RGS1
0.78	SLA
0.784	PGM5
0.788	PRKD2
0.792	KIAA1407
0.796	GMFG
0.8	LOC153684
0.804	ELK3
0.808	GIMAP4
0.812	GAB3
0.816	MATN2
0.82	PHF10
0.824	GYPB
0.828	GJB2
0.832	SLC7A7
0.836	HLA-DQA2
0.84	LY96
0.844	CTBS
0.848	STXBP4
0.852	TTF2
0.856	CD14
0.86	PSG6
0.864	IL18
0.868	SLC25A43
0.872	NAGA
0.876	IFI44
0.88	PARP12
0.884	NR2F2
0.888	ACY3

0.21807	PSMA3
0.21928	HCCS
0.22048	POLR3B
0.22169	DCUN1D5
0.22289	ARL5B
0.22410	UQCRC2
0.22530	CSNK2A2
0.22651	LOC646482
0.22771	TM9SF2
0.22892	MRPL45
0.23012	CTSB
0.23133	CDKL5
0.23253	PWP1
0.23373	NDUFA12
0.23494	USPL1
0.23614	SLC36A4
0.23735	TM2D2
0.23855	VCP
0.23976	FAM190B
0.24096	SUCLA2
0.24217	RLIM
0.24337	TRAPPC6B
0.24458	CINP
0.24578	GDE1
0.24699	EIF3M
0.24819	SLC9A7
0.24940	CIRH1A
0.25060	RAB18
0.25181	HTR2A
0.25301	SLC39A9
0.25422	RSL1D1
0.25542	FBXO3
0.25663	DRG1
0.25783	SNAPC3
0.25904	ARL2BP
0.26024	DMXL2
0.26145	CHP
0.26265	NPEPPS
0.26386	PCNX
0.26506	C16orf72
0.26627	ACTR1A
0.26747	ATP6V1H

0.82	LOC100131727
0.83	GUCA1A
0.83	TNFRSF11A
0.84	STX1A
0.84	CDH9
0.85	FREM3
0.85	GNG4
0.85	SMARCA1
0.86	EGR3
0.86	MYO16
0.87	GULP1
0.87	FBLN5
0.88	TSHZ2
0.88	CPNE8
0.89	PTCHD1
0.89	GALNT14
0.90	VAT1
0.90	FREM3
0.90	LIN37
0.91	GPR120
0.91	PJA1
0.92	GRB10
0.92	GALNTL6
0.93	SLC22A9
0.93	SST
0.94	FREM3
0.94	FREM3
0.95	JUN
0.95	PVRL3
0.95	GPR107
0.96	EFR3B
0.96	ANXA2P2
0.97	XKR4
0.97	GRK6
0.98	LOC100132987
0.98	DNAL1
0.99	KCNN2
0.99	LOC157503
1.00	CPLX3

0.67537	NUPL1
0.67910	MAPRE2
0.68284	ELP2
0.68657	BMI1
0.69030	JAK2
0.69403	VPS26A
0.69776	CLUAP1
0.70149	NUP188
0.70522	TXNDC16
0.70896	KIAA1370
0.71269	PHYHIPL
0.71642	CYB5B
0.72015	DLG2
0.72388	PSIP1
0.72761	GOLGA5
0.73134	ERGIC2
0.73507	RNF24
0.73881	CHORDC1
0.74254	RBM4
0.74627	DDX52
0.75000	KIAA0247
0.75373	PATL1
0.75746	IFNAR2
0.76119	SYNRG
0.76493	DDHD1
0.76866	PCBP2
0.77239	SGPP1
0.77612	NBR1
0.77985	ZNF8
0.78358	PDCD11
0.78731	VBP1
0.79104	PPP1CC
0.79478	RALGPS1
0.79851	SH3BGRL
0.80224	RFWD3
0.80597	PHF12
0.80970	SAMD12
0.81343	ZNF615
0.81716	ARMCX4
0.82090	PRPF18
0.82463	NDUFB8
0.82836	SUV420H1

0.06076	CGA
0.06109	WDR93
0.06143	CNTLN
0.06177	LOC283867
0.06210	CASC5
0.06244	FANCC
0.06277	MPP4
0.06311	FAM71D
0.06344	TBC1D8B
0.06378	DUS4L
0.06412	CENPM
0.06445	SERPINC1
0.06479	ZNF663
0.06512	C1orf101
0.06546	EMCN
0.06579	TNIP3
0.06613	CRNDE
0.06647	CHPT1
0.06680	VIT
0.06714	RAG1
0.06747	LOC286467
0.06781	LOC729032
0.06814	NMI
0.06848	CEP135
0.06882	TMEM207
0.06915	GPR55
0.06949	C2orf83
0.06982	GCNT2
0.07016	LOC644456
0.07049	ACSM3
0.07083	CYP2C18
0.07116	SERPINB10
0.07150	SIX1
0.07184	CCDC9
0.07217	GIMAP8
0.07251	LEAP2
0.07284	BEYLA
0.07318	MAK
0.07351	CD180
0.07385	IK
0.07419	NOD1
0.07452	LOC283314

0.181	NRG1
0.182	C8orf79
0.183	BEND6
0.184	L2HGDH
0.185	EPN3
0.186	PLCB4
0.187	MPP7
0.188	RGS6
0.189	ATP2B2
0.19	MID2
0.191	SIX4
0.192	CRHR2
0.193	FMN1
0.194	SLC29A1
0.195	ABCB9
0.196	RELT
0.197	ZYX
0.198	ISCU
0.199	COX5A
0.2	LRRC59
0.201	FBLN7
0.202	ADCYAP1
0.203	FAM83D
0.204	DIRAS1
0.205	SLC25A37
0.206	FAM57B
0.207	NUAK1
0.208	KIAA1797
0.209	CARS
0.21	UQCRB
0.211	LGI2
0.212	PLXDC1
0.213	BCAT1
0.214	SRXN1
0.215	APOL2
0.216	TPST2
0.217	C8orf79
0.218	CPLX1
0.219	LOC100131599
0.22	SMC2
0.221	VIPR2
0.222	LOC100129717

	0.80797	LOC100288205	0.15497	CNIH4	0.892	ST7		0.26867	PHLPP2		0.83209	HSD17B12	0.07486	OR52J3	0.223	DEXI
	0.81159	PQLC1	0.15566	QRICH1	0.896	C5orf35		0.26988	ELAVL1		0.83582	ZNFX1	0.07519	PROL1	0.224	SLIT2
	0.81522	C9orf37	0.15636	BZW2	0.9	ZNF208		0.27108	RAN		0.83955	GPR107	0.07553	ZCWPW2	0.225	ARHGAP9
	0.81884	AK1	0.15705	C3orf10	0.904	HEG1		0.27229	MORF4L2		0.84328	C8orf45	0.07586	RSPH4A	0.226	UQCRC1
	0.82246	CYTH2	0.15775	DEGS1	0.908	TCIRG1		0.27349	M6PR		0.84701	CREBL2	0.07620	POU1F1	0.227	IPW
	0.82609	LONP1	0.15844	ANKRD13C	0.912	DENND2A		0.27470	UTP18		0.85075	CRADD	0.07654	OR51B2	0.228	ALDH1B1
	0.82971	POLL	0.15914	CHN1	0.916	CCDC125		0.27590	DPP8		0.85448	PCGF5	0.07687	AKNAD1	0.229	HM13
	0.83333	FAM127B	0.15983	PAPOLG	0.92	CD53		0.27711	VPS39		0.85821	AKAP1	0.07721	ADAM5P	0.23	ARID5B
	0.83696	MRPL23	0.16053	TAX1BP1	0.924	CXorf38		0.27831	POLE3		0.86194	DHX40	0.07754	CCDC38	0.231	TOX2
	0.84058	C19orf53	0.16122	SLC35B4	0.928	HLA-DPB2		0.27952	KIAA0368		0.86567	ZNF202	0.07788	TNFSF8	0.232	RAD54B
	0.84420	GNA15	0.16192	KIAA0319L	0.932	CD69		0.28072	IPO7		0.86940	TRAPPC2	0.07821	IL15	0.233	PAQR9
	0.84783	BAD	0.16261	PHF14	0.936	TTC7A		0.28193	SCAI		0.87313	EXT1	0.07855	FRMD7	0.234	SPOCK2
	0.85145	STUB1	0.16331	BRAF	0.94	OLFML3		0.28313	C12orf51		0.87687	ZFX	0.07889	MUTYH	0.235	HCN2
	0.85507	PPP1R16B	0.16400	MTOR	0.944	CD2AP		0.28434	DAAM1		0.88060	PITRM1	0.07922	LIPJ	0.236	LRRK2
	0.85870	FXYD7	0.16470	COPS4	0.948	ESD		0.28554	PANX1		0.88433	CBFA2T2	0.07956	FSD2	0.237	RIMKLA
	0.86232	LPXN	0.16539	PPP1R7	0.952	DCAF12		0.28675	MUDENG		0.88806	ACER3	0.07989	TMEM68	0.238	EPB41
	0.86594	C11orf24	0.16609	FAM135A	0.956	C11orf65		0.28795	GRIA3		0.89179	DKC1	0.08023	CCDC36	0.239	SLC25A37
	0.86957	GAS7	0.16678	SMAP1	0.96	TMEM218		0.28916	MKKS		0.89552	TNRC6C	0.08056	ACTL6A	0.24	MYST1
	0.87319	TAF10	0.16748	SLU7	0.964	LOC645146		0.29036	C10orf46		0.89925	TXNL4B	0.08090	LOC149620	0.241	EML2
	0.87681	NONO	0.16817	LOC221710	0.968	UBE2E1		0.29157	SNUPN		0.90299	EXOSC6	0.08124	KIF15	0.242	C12orf10
	0.88043	INO80C	0.16887	RBBP5	0.972	CARD6		0.29277	PTEN		0.90672	TNRC6C	0.08157	TMC2	0.243	SLCO4A1
	0.88406	AXIN2	0.16956	TBC1D22B	0.976	CYFIP1		0.29398	THYN1		0.91045	YPEL2	0.08191	SPINK5	0.244	STARD5
	0.88768	MPRIP	0.17026	PPP1R9A	0.98	HLA-DPB2		0.29518	LOC100129974		0.91418	C17orf28	0.08224	ATP6V0D2	0.245	POU6F2
	0.89130	SPNS1	0.17095	LASS6	0.984	HLA-DPB1		0.29639	STOML2		0.91791	ZNF143	0.08258	SPATA1	0.246	ZNF385D
	0.89493	X3687752	0.17165	RNGTT	0.988	ARL6IP6		0.29759	FBXW2		0.92164	LOC100130363	0.08291	SAMD3	0.247	TRPC3
	0.89855	DCAF12L2	0.17234	RMND5A	0.992	C2orf84		0.29880	ACADSB		0.92537	C17orf70	0.08325	A4GNT	0.248	DAPK3
	0.90217	RNF44	0.17304	MAPK10	0.996	NINJ1		0.30000	RNMT		0.92910	ZNF132	0.08359	MSX1	0.249	VWC2
	0.90580	ZBED4	0.17373	DNM3				0.30120	COPB1		0.93284	ZFP3	0.08392	ZNF501	0.25	AVPI1
	0.90942	ANAPC11	0.17443	SMYD2				0.30241	TMEM188		0.93657	ST13	0.08426	NEDD1	0.251	CHRM2
	0.91304	DNAJB12	0.17512	HMGCR				0.30361	GNPNAT1		0.94030	PACS2	0.08459	FAM26E	0.252	STAT4
	0.91667	NCOA5	0.17582	DPY19L2P2				0.30482	SAR1A		0.94403	MRPS16	0.08493	BTN3A1	0.253	CA7
	0.92029	PEMT	0.17651	C3orf14				0.30602	NAE1		0.94776	BAZ2A	0.08526	RWDD3	0.254	EFNA5
	0.92391	PRR12	0.17721	TSNAX				0.30723	RIT2		0.95149	PHF6	0.08560	LOC100132798	0.255	PLA2R1
	0.92754	CECR5	0.17790	RNASEN				0.30843	C16orf70		0.95522	RP11-756A22.3	0.08593	LRRC69	0.256	FAM81A
	0.93116	MAP3K12	0.17860	FAM164A				0.30964	IPO5		0.95896	SRRM2	0.08627	CATSPER2P1	0.257	CORO2A
	0.93478	SLC9A8	0.17929	MGAT4A				0.31084	IMPAD1		0.96269	LIN7C	0.08661	LDHAL6A	0.258	C12orf24
	0.93841	GPR173	0.17999	C4orf14				0.31205	SEC61A2		0.96642	FUZ	0.08694	MCM2	0.259	RORA
	0.94203	NAA40	0.18068	TMEM131				0.31325	RTN1		0.97015	RBM14	0.08728	ZNF321	0.26	RBBP4
	0.94565	RPAP1	0.18138	ABCE1				0.31446	ZNF828		0.97388	USP36	0.08761	CRYGC	0.261	PRSS16
	0.94928	GMEB2	0.18207	TSGA14				0.31566	SLC41A2		0.97761	RNF219	0.08795	RPL13AP17	0.262	STARD10
	0.95290	LOC25845	0.18277	LMBR1				0.31687	TOR1A		0.98134	UCKL1	0.08828	TAAR8	0.263	FAT3
	0.95652	SEC16A	0.18346	REEP5				0.31807	PAFAH1B2		0.98507	LOC100131878	0.08862	AMPD1	0.264	NT5M

0.96014	DIDO1	0.18416	SLC39A10	0.31928	ALKBH5	0.98881	C16orf53	0.08896	ZIC3	0.265	ARHGAP10
0.96377	ZNF575	0.18485	ARL8B	0.32048	PHF20	0.99254	MICAL3	0.08929	HLA-DOA	0.266	MAST4
0.96739	LOC100128332	0.18555	HNRNPAB	0.32169	IKBKAP	0.99627	FLYWCH1	0.08963	LOC100128739	0.267	ARHGEF11
0.97101	C12orf64	0.18624	DNAJC6	0.32289	VAPA			0.08996	IRAK4	0.268	PCGF1
0.97464	LOC100288312	0.18694	PCYOX1	0.32410	NDUFC2			0.09030	FRRS1	0.269	FAM43A
0.97826	ZGPAT	0.18763	SLC25A4	0.32530	C20orf24			0.09063	NR1I2	0.27	CAMKK1
0.98188	ZNF250	0.18833	FH	0.32651	HN1			0.09097	UGT1A1	0.271	RUSC1
0.98551	ARRDC1	0.18902	PPP3R1	0.32771	MGEA5			0.09131	C1orf185	0.272	C18orf25
0.98913	PWP2	0.18972	SDAD1	0.32892	AQR			0.09164	LOC727941	0.273	CCDC25
0.99275	IGHMBP2	0.19041	FBXO25	0.33012	YWHAB			0.09198	HIST1H2AA	0.274	SHROOM2
0.99638	ZNF385C	0.19110	ATR	0.33133	SAMD12			0.09231	GALNT5	0.275	SLC25A37
		0.19180	DCTD	0.33253	RPA1			0.09265	RTBDN	0.276	GAS6
		0.19249	SGIP1	0.33373	USP25			0.09298	GLYATL2	0.277	ONECUT2
		0.19319	CHMP7	0.33494	PRDM4			0.09332	LOC730100	0.278	NRIP2
		0.19388	SLC17A5	0.33614	NDFIP2			0.09366	CXorf22	0.279	CAMK2G
		0.19458	TMCO1	0.33735	DYM			0.09399	C10orf112	0.28	INPP5F
		0.19527	SLC8A1	0.33855	GTF2H1			0.09433	FLJ27351	0.281	CYTSB
		0.19597	GRPEL2	0.33976	SPPL3			0.09466	FAM133B	0.282	NTNG1
		0.19666	WDR36	0.34096	ARMCX5			0.09500	ATP13A5	0.283	NDRG4
		0.19736	GOPC	0.34217	MBTPS2			0.09533	LOC285638	0.284	ZNF365
		0.19805	EFR3A	0.34337	GRB2			0.09567	ZNF180	0.285	ZDHHC22
		0.19875	CADM2	0.34458	AKAP6			0.09601	CYP3A5	0.286	FLT3
		0.19944	YTHDC2	0.34578	COPS3			0.09634	DCAF13	0.287	SLC9A1
		0.20014	SMG7	0.34699	AAGAB			0.09668	SPRR2F	0.288	C1orf133
		0.20083	PPID	0.34819	KLHL9			0.09701	UBE2DNL	0.289	PHF17
		0.20153	PLEKHB2	0.34940	FAM199X			0.09735	BEST3	0.29	TAF13
		0.20222	GDAP2	0.35060	LIG4			0.09768	LOC441453	0.291	PSMG3
		0.20292	EFCAB7	0.35181	PPP6C			0.09802	BCORL2	0.292	GPCPD1
		0.20361	ESYT2	0.35301	NCOA2			0.09836	FCF1	0.293	GARNL3
		0.20431	SGIP1	0.35422	POP4			0.09869	MAP7D3	0.294	KLF12
		0.20500	GFPT1	0.35542	ZFP1			0.09903	SYTL4	0.295	SRXN1
		0.20570	UBLCP1	0.35663	RBM11			0.09936	LOC399815	0.296	ESYT1
		0.20639	NDUFB5	0.35783	GABARAPL2			0.09970	OR13C3	0.297	FGF5
		0.20709	RTN4	0.35904	OGFOD1			0.10003	TOP2A	0.298	MTUS2
		0.20778	EBNA1BP2	0.36024	SLC2A3			0.10037	RBM44	0.299	NAGPA
		0.20848	SMYD3	0.36145	APOO			0.10070	VGLL2	0.3	LOC201477
		0.20917	MRPL37	0.36265	EAPP			0.10104	HTRA4	0.301	CNTN5
		0.20987	C6orf120	0.36386	LARP4B			0.10138	OR4C3	0.302	TRIM35
		0.21056	RICTOR	0.36506	TM7SF3			0.10171	HHLA1	0.303	OXGR1
		0.21126	PARK2	0.36627	DNAJA1			0.10205	GALNT3	0.304	TBC1D8
		0.21195	MAP1B	0.36747	GAPVD1			0.10238	IHH	0.305	HS6ST3
		0.21265	ZNF623	0.36867	GSR			0.10272	C20orf141	0.306	HLF

		0.21334	SCOC			0.36988	SNX30			0.10305	NAPSA	0.307	KIAA1199
		0.21404	C1orf163			0.37108	C13orf1			0.10339	G6PC2	0.308	PRDM8
		0.21473	DHX29			0.37229	TCEB1			0.10373	IDO1	0.309	LNX2
		0.21543	YTHDF3			0.37349	MTCH2			0.10406	LRRC66	0.31	MAN1C1
		0.21612	RSPH3			0.37470	C18orf45			0.10440	STATH	0.311	INSM2
		0.21682	CSNK1A1			0.37590	BEX4			0.10473	C6	0.312	FIBP
		0.21751	LIAS			0.37711	RCN2			0.10507	KIAA1524	0.313	SLC35C1
		0.21821	SNX2			0.37831	UBE2Z			0.10540	HTR3E	0.314	KIAA0748
		0.21890	LMBR1			0.37952	TRUB1			0.10574	UFM1	0.315	RBMS1
		0.21960	KBTBD2			0.38072	GPR180			0.10608	TGDS	0.316	CDON
		0.22029	MRPL9			0.38193	PPP2R5E			0.10641	C8B	0.317	KCNJ11
		0.22099	TRIM23			0.38313	NPAT			0.10675	OR52E5	0.318	ABCC8
		0.22168	FBXW11			0.38434	CDC37L1			0.10708	LOC644841	0.319	CENPV
		0.22238	C7orf42			0.38554	RNF160			0.10742	CUBN	0.32	ITGA11
		0.22307	TGFBRAP1			0.38675	DPAGT1			0.10775	CCHCR1	0.321	KIAA0182
		0.22377	RNF14			0.38795	HSPA5			0.10809	DISC2	0.322	ESRRA
		0.22446	FASTKD2			0.38916	MRPL34			0.10843	C14orf105	0.323	KNG1
		0.22516	C7orf70			0.39036	CDK8			0.10876	C17orf57	0.324	RWDD1
		0.22585	LOC100292648			0.39157	TEX10			0.10910	C1orf112	0.325	TTC39A
		0.22655	GTPBP10			0.39277	CCDC113			0.10943	EOMES	0.326	ST3GAL6
		0.22724	DGKI			0.39398	DNAJC7			0.10977	TCP11	0.327	PLS1
		0.22794	STXBP5			0.39518	C14orf1			0.11010	LCT	0.328	CYTSB
		0.22863	ABL2			0.39639	PIM2			0.11044	BPI	0.329	IER5
		0.22933	C1orf216			0.39759	POMP			0.11078	NAALADL2	0.33	ONECUT2
		0.23002	GTF2B			0.39880	DDX25			0.11111	IQGAP2	0.331	CYTSB
		0.23072	DYNC1I1			0.40000	TLN2			0.11145	TLR1	0.332	CCNDBP1
		0.23141	NRCAM			0.40120	MLLT3			0.11178	MRGPRX2	0.333	WDR76
		0.23211	WDR75			0.40241	AFG3L2			0.11212	CNR2	0.334	LOC284930
		0.23280	LCLAT1			0.40361	TXNL1			0.11245	MSTN	0.335	ZNF295
		0.23350	LOC152217			0.40482	NFAT5			0.11279	MYO3A	0.336	DCLRE1A
		0.23419	PLCL2			0.40602	FBXO21			0.11313	RAB27A	0.337	PNMAL2
		0.23489	CD47			0.40723	NFS1			0.11346	ZNF254	0.338	MAMDC2
		0.23558	GPD1L			0.40843	GGNBP2			0.11380	C6orf150	0.339	CACNA2D2
		0.23628	ORC5L			0.40964	BCAS3			0.11413	SYT8	0.34	PAG1
		0.23697	KDM1A			0.41084	GOLT1B			0.11447	TPO	0.341	CHAC1
		0.23767	LPGAT1			0.41205	ZDHHC16			0.11480	RPGR	0.342	KIAA0564
		0.23836	VPS8			0.41325	MCTS1			0.11514	HOXB2	0.343	GDPD5
		0.23905	LMLN			0.41446	C15orf17			0.11547	MTTP	0.344	WNT3
		0.23975	NSUN4			0.41566	FAM175B			0.11581	LPAL2	0.345	RORA
		0.24044	SLC25A44			0.41687	KIAA1468			0.11615	LOC647107	0.346	CLN6
		0.24114	ATP13A3			0.41807	PANK2			0.11648	HELB	0.347	HUNK
		0.24183	VPS13D			0.41928	KIAA1632			0.11682	TMEM38B	0.348	CMYA5

		0.24253	NUDT17			0.42048	PSD3			0.11715	LOC253724	0.349	ZFYVE19
		0.24322	TDP2			0.42169	AP3S2			0.11749	OR4Q2	0.35	RHOBTB1
		0.24392	HDAC2			0.42289	TMEM132B			0.11782	FLJ42875	0.351	ACPL2
		0.24461	RPS6KC1			0.42410	PGAM5			0.11816	C6orf94	0.352	SMARCA2
		0.24531	SCRN3			0.42530	POLR2C			0.11850	C6orf103	0.353	FITM2
		0.24600	USP4			0.42651	FASTKD5			0.11883	UTS2D	0.354	MID2
		0.24670	ZMPSTE24			0.42771	LOC100379224			0.11917	DYNLRB2	0.355	KITLG
		0.24739	LYRM4			0.42892	PALB2			0.11950	MND1	0.356	ATP5A1
		0.24809	ARV1			0.43012	SVIP			0.11984	FAM160A1	0.357	ANKRD24
		0.24878	TMEM181			0.43133	PPP1R12A			0.12017	WHAMML1	0.358	PICK1
		0.24948	ELOVL6			0.43253	LOC100131208			0.12051	FANCI	0.359	RTKN2
		0.25017	PRICKLE2			0.43373	AMN1			0.12085	GLT6D1	0.36	EIF1AD
		0.25087	SRPRB			0.43494	P2RX5			0.12118	BMP2	0.361	CADPS2
		0.25156	LOC100292361			0.43614	ZNF597			0.12152	FANCD2	0.362	ITM2A
		0.25226	KTI12			0.43735	RAB39B			0.12185	C2orf58	0.363	SLC25A17
		0.25295	C1orf25			0.43855	UBE3A			0.12219	SGMS2	0.364	SYCP2
		0.25365	ZDBF2			0.43976	API5			0.12252	LOC100292664	0.365	TMEM38A
		0.25434	GPR22			0.44096	HMG20A			0.12286	PGBD2	0.366	PLXNA4
		0.25504	RNF19B			0.44217	RPS6KA6			0.12320	ZNF850P	0.367	AR
		0.25573	SERPINI1			0.44337	KIAA2022			0.12353	MLL3	0.368	AMMECR1
		0.25643	CCT4			0.44458	FAM32A			0.12387	BCHE	0.369	ARHGAP39
		0.25712	VPRBP			0.44578	UBQLN2			0.12420	C2orf84	0.37	PYROXD1
		0.25782	RNF115			0.44699	NTRK3			0.12454	PI4K2B	0.371	CRHR1
		0.25851	MRPS21			0.44819	C10orf88			0.12487	PDZD9	0.372	DYNLL2
		0.25921	C7orf30			0.44940	RNF170			0.12521	ADAM18	0.373	ONECUT2
		0.25990	ADAM9			0.45060	TSTA3			0.12555	PEX7	0.374	HIST1H4A
		0.26060	RYR2			0.45181	LOC387790			0.12588	C9orf53	0.375	PGD
		0.26129	TIMM17A			0.45301	CACNB2			0.12622	PHEX	0.376	SLC38A11
		0.26199	NOP58			0.45422	TXNL4A			0.12655	LOC654841	0.377	CUX1
		0.26268	LZTFL1			0.45542	HDAC8			0.12689	C15orf33	0.378	CEND1
		0.26338	UBE2E2			0.45663	C16orf63			0.12722	PCOLCE2	0.379	RAB11B
		0.26407	USP42			0.45783	BRAP			0.12756	KCNE1	0.38	PRRT3
		0.26477	HTRA2			0.45904	TBK1			0.12790	LOC100287822	0.381	H1F0
		0.26546	ZNF148			0.46024	RPRD1A			0.12823	VANGL1	0.382	ONECUT2
		0.26616	OXSR1			0.46145	LOC90110			0.12857	CKAP2L	0.383	PPARD
		0.26685	ATP2C1			0.46265	ATAD1			0.12890	SYNE2	0.384	HSBP1L1
		0.26755	RSBN1			0.46386	MAPK6			0.12924	MIR382	0.385	CDR2L
		0.26824	HGSNAT			0.46506	C20orf3			0.12957	CFB	0.386	ZBTB7C
		0.26894	HNRNPA3			0.46627	LOC100129942			0.12991	ESR2	0.387	NPM2
		0.26963	GCC2			0.46747	NETO2			0.13025	ZNF677	0.388	LOC283683
		0.27033	GTF3C3			0.46867	PLEKHA5			0.13058	IFT88	0.389	LOC157627
		0.27102	NDUFA5			0.46988	FTO			0.13092	ZNF560	0.39	GAS2

		0.27172	LARS			0.47108	LARP4			0.13125	ULBP1	0.391	DNAJC4
		0.27241	LRRTM2			0.47229	TMED8			0.13159	CLNK	0.392	CDADC1
		0.27311	WWP1			0.47349	KIAA1377			0.13192	TTC27	0.393	RAB11FIP5
		0.27380	MGST3			0.47470	LOC283567			0.13226	TMEM37	0.394	LOC100128668
		0.27450	VKORC1L1			0.47590	RRM2B			0.13259	ZNF491	0.395	MCOLN1
		0.27519	ADAR			0.47711	UQCC			0.13293	PSG9	0.396	GPR137C
		0.27589	UBR3			0.47831	C16orf80			0.13327	PBOV1	0.397	GLCCI1
		0.27658	FAM98A			0.47952	SLC25A14			0.13360	EPC1	0.398	PARD6B
		0.27728	RRAGC			0.48072	CDYL2			0.13394	LOC339260	0.399	LOC286272
		0.27797	ACOT13			0.48193	SRP54			0.13427	NUP107	0.4	PPL
		0.27867	AGGF1			0.48313	PRKAR1A			0.13461	GYS2	0.401	AGPAT3
		0.27936	WDR33			0.48434	TDP1			0.13494	ECM2	0.402	ZADH2
		0.28006	KDELR2			0.48554	BC036928			0.13528	ATP8B5P	0.403	GPR26
		0.28075	PDK1			0.48675	PARP2			0.13562	PLEKHG7	0.404	HEXIM1
		0.28145	DCK			0.48795	C22orf30			0.13595	IGSF22	0.405	NPR2
		0.28214	FAM115A			0.48916	PTPRO			0.13629	C2orf63	0.406	TC2N
		0.28284	LYST			0.49036	XK			0.13662	SNHG11	0.407	SORL1
		0.28353	CCBL2			0.49157	PSMB6			0.13696	DNAH10	0.408	TRPV6
		0.28423	ITGB1BP1			0.49277	WDR74			0.13729	EP400NL	0.409	BTBD11
		0.28492	ARPC2			0.49398	PRPF4			0.13763	RASSF6	0.41	ABCC1
		0.28562	UBE2V2			0.49518	GANAB			0.13797	FLJ37638	0.411	FAM189A1
		0.28631	GTDC1			0.49639	DNTTIP1			0.13830	PIWIL4	0.412	PHYH
		0.28700	TARS			0.49759	DSN1			0.13864	C9orf153	0.413	CAMK2N2
		0.28770	CAMK4			0.49880	SLC6A15			0.13897	LOC100287731	0.414	SLC7A1
		0.28839	CGGBP1			0.50000	PLDN			0.13931	TSPAN19	0.415	PMEPA1
		0.28909	IP6K1			0.50120	TMOD2			0.13964	TCF19	0.416	HRNBP3
		0.28978	FYTTD1			0.50241	VIPAR			0.13998	LOC81691	0.417	ACVR1C
		0.29048	PHAX			0.50361	ABCA5			0.14032	NCRNA00119	0.418	ANK3
		0.29117	WASL			0.50482	TRUB2			0.14065	DLEU1	0.419	ABLIM1
		0.29187	TMEM57			0.50602	SC5DL			0.14099	ZNF30	0.42	FAM98C
		0.29256	LOC90784			0.50723	DCX			0.14132	AKR1D1	0.421	SGK223
		0.29326	PRR3			0.50843	MAK10			0.14166	BCAS2	0.422	BLMH
		0.29395	ATL2			0.50964	COPZ1			0.14199	CPS1	0.423	SLFN5
		0.29465	MCM4			0.51084	ZNF91			0.14233	PLSCR1	0.424	SDR16C5
		0.29534	AGBL4			0.51205	CPD			0.14267	TDRD1	0.425	ARHGAP32
		0.29604	CDC42			0.51325	VPS25			0.14300	CTHRC1	0.426	EMB
		0.29673	NDUFA2			0.51446	C12orf51			0.14334	ZNF546	0.427	LIN52
		0.29743	PCGF3			0.51566	TMEM62			0.14367	VEPH1	0.428	HRH3
		0.29812	HDLBP			0.51687	ETNK1			0.14401	HOXA2	0.429	SLC47A1
		0.29882	CLDN12			0.51807	ZDHHC15			0.14434	IFIH1	0.43	PCP4L1
		0.29951	RINT1			0.51928	NTRK3			0.14468	RPSA	0.431	LOC644397
		0.30021	TDRKH			0.52048	TTPAL			0.14502	TBX21	0.432	TMEM81

		0.30090	MMADHC			0.52169	CCDC53			0.14535	MMEL1	0.433	IDE
		0.30160	NAA50			0.52289	PRPS1			0.14569	CYP20A1	0.434	ZBTB1
		0.30229	FAM169A			0.52410	ALG9			0.14602	PM20D1	0.435	MX1
		0.30299	NDUFS5			0.52530	ACACA			0.14636	ALG6	0.436	NFIC
		0.30368	HEXB			0.52651	NARS2			0.14669	ACADL	0.437	NRSN1
		0.30438	ANGEL2			0.52771	MRPL17			0.14703	LOC100130178	0.438	MAFB
		0.30507	UBR3			0.52892	SNX16			0.14736	DGKK	0.439	IMPG1
		0.30577	SEC22B			0.53012	TERF2			0.14770	CUZD1	0.44	LOC652797
		0.30646	CDC42SE2			0.53133	TTC8			0.14804	SASS6	0.441	FRAS1
		0.30716	HACE1			0.53253	VPS13A			0.14837	PATE2	0.442	MYO5B
		0.30785	GTF2H5			0.53373	MAPK1IP1L			0.14871	SFRP4	0.443	TMEM120B
		0.30855	BLZF1			0.53494	DDB1			0.14904	LOC100288922	0.444	DHX35
		0.30924	DPP6			0.53614	EXOSC2			0.14938	ZNF67P	0.445	ZNF519
		0.30994	FAM136A			0.53735	LGMN			0.14971	C8orf45	0.446	LOC100128253
		0.31063	LRRC8B			0.53855	CUEDC2			0.15005	FREM1	0.447	ARSG
		0.31133	MFAP3			0.53976	C11orf80			0.15039	PYCARD	0.448	IQSEC1
		0.31202	DDI2			0.54096	KCTD2			0.15072	PLSCR2	0.449	CYB5D1
		0.31272	SYT11			0.54217	PRNP			0.15106	CARD14	0.45	LRRC20
		0.31341	TTC33			0.54337	MSMP			0.15139	TRPV6	0.451	ZNF804B
		0.31411	C1orf31			0.54458	MRPL46			0.15173	PSMB8	0.452	TESC
		0.31480	XPO5			0.54578	ZNF542			0.15206	GPC3	0.453	FLYWCH2
		0.31550	C5orf56			0.54699	C14orf2			0.15240	C5orf46	0.454	UNC13C
		0.31619	LANCL1			0.54819	NTRK3			0.15274	ARHGEF5	0.455	CDR2
		0.31689	SGK196			0.54940	NTRK3			0.15307	LOC100288130	0.456	SLC45A1
		0.31758	GFM2			0.55060	C18orf10			0.15341	DNAH10	0.457	GPR26
		0.31828	PCDHB2			0.55181	RAB14			0.15374	KATNA1	0.458	PPP2R2D
		0.31897	MFF			0.55301	SEPHS2			0.15408	COX17	0.459	C8orf79
		0.31967	TMEM170B			0.55422	FBXL20			0.15441	ZNF732	0.46	GLB1L3
		0.32036	DNAJB9			0.55542	C15orf24			0.15475	SLC2A4	0.461	ADCY1
		0.32106	FBXO45			0.55663	TSFM			0.15509	LOC100129318	0.462	MPPED1
		0.32175	WBSCR22			0.55783	TRPC4AP			0.15542	EFR3B	0.463	RTDR1
		0.32245	FLVCR1			0.55904	GMPR2			0.15576	HPGD	0.464	ECM1
		0.32314	RABGGTB			0.56024	DCAF10			0.15609	PCA3	0.465	RNLS
		0.32384	TADA1			0.56145	GTF3C4			0.15643	PRRG4	0.466	LOC729175
		0.32453	TRAPPC3			0.56265	GNAL			0.15676	LRRC56	0.467	CUX2
		0.32523	PSMD2			0.56386	BDNF			0.15710	LOC100128908	0.468	ATP8B1
		0.32592	RABGEF1			0.56506	SUDS3			0.15744	KIF14	0.469	GRIP2
		0.32662	UBE2D3			0.56627	C13orf37			0.15777	TRDN	0.47	RASSF3
		0.32731	DNAJB4			0.56747	GNE			0.15811	NUP37	0.471	SPAG6
		0.32801	STX12			0.56867	PITPNB			0.15844	DNASE2B	0.472	GPR161
		0.32870	SDCBP			0.56988	TRMT112			0.15878	LOH3CR2A	0.473	CABLES2
		0.32940	PPIG			0.57108	YTHDF1			0.15911	C6orf167	0.474	MATK

		0.33009	GNPAT			0.57229	CBX5			0.15945	LOC650095	0.475	TLE2
		0.33079	EXTL3			0.57349	LOC729860			0.15979	PRSS55	0.476	FAM115C
		0.33148	KIAA1967			0.57470	CRK			0.16012	CXorf24	0.477	INTS9
		0.33218	ZFAND2A			0.57590	MRPL39			0.16046	LOC100132529	0.478	PLCH1
		0.33287	VPS24			0.57711	MTHFD1			0.16079	LYAR	0.479	ONECUT2
		0.33356	TOMM20			0.57831	UBE3B			0.16113	CXCL13	0.48	NAT8L
		0.33426	LYRM7			0.57952	RNF38			0.16146	PPIC	0.481	DPP4
		0.33495	SCCPDH			0.58072	ALG8			0.16180	STAC	0.482	ETV6
		0.33565	MAD2L1BP			0.58193	BAG5			0.16213	GPR109B	0.483	MFSD3
		0.33634	ATIC			0.58313	LOC550643			0.16247	TMEM51	0.484	LRCH1
		0.33704	COX18			0.58434	POLR3K			0.16281	LOC221946	0.485	ATP1B3
		0.33773	SAP30L			0.58554	NALCN			0.16314	PYROXD1	0.486	CCNE1
		0.33843	ACTR3B			0.58675	BFAR			0.16348	C11orf73	0.487	XKR6
		0.33912	ANO10			0.58795	PCMTD1			0.16381	LOC100128841	0.488	C15orf27
		0.33982	EBAG9			0.58916	MED6			0.16415	C4orf47	0.489	HTR1B
		0.34051	KIAA0232			0.59036	AKAP5			0.16448	LOC100133994	0.49	SLC17A6
		0.34121	TUFT1			0.59157	PIAS2			0.16482	SLFN12L	0.491	SARS2
		0.34190	JMJD4			0.59277	SCFD1			0.16516	SLC25A5	0.492	UPF2
		0.34260	CDC5L			0.59398	PRDX3			0.16549	PLAC1	0.493	PHOSPHO1
		0.34329	AKAP9			0.59518	CA10			0.16583	OGG1	0.494	MYH7B
		0.34399	TTC1			0.59639	NSDHL			0.16616	WNT4	0.495	ALDH1A3
		0.34468	DR1			0.59759	FAM127A			0.16650	MYO1H	0.496	DKK1
		0.34538	CAPZA1			0.59880	EXOSC3			0.16683	USH2A	0.497	ONECUT2
		0.34607	RIF1			0.60000	VPS33B			0.16717	NUP210L	0.498	CTXN2
		0.34677	CCNC			0.60120	LAGE3			0.16751	DNAJA2	0.499	BTBD3
		0.34746	C5orf44			0.60241	GLG1			0.16784	LOC220077	0.5	MCF2L
		0.34816	CHIC2			0.60361	C12orf29			0.16818	LOC730338	0.501	C18orf21
		0.34885	FAM117B			0.60482	ATXN3			0.16851	LIPC	0.502	ZNF740
		0.34955	AP3S1			0.60602	CBL			0.16885	HOXA7	0.503	FADS6
		0.35024	SERAC1			0.60723	SYAP1			0.16918	PROK2	0.504	SFRS14
		0.35094	COQ10B			0.60843	OAT			0.16952	IL20RB	0.505	TIAM1
		0.35163	UBE4B			0.60964	SENP8			0.16986	TNFSF18	0.506	ECSIT
		0.35233	TMEM48			0.61084	MLX			0.17019	CNGB3	0.507	BMS1
		0.35302	TCERG1			0.61205	HSP90B1			0.17053	ZAN	0.508	MRPS26
		0.35372	RAB4A			0.61325	BLOC1S2			0.17086	EYS	0.509	COASY
		0.35441	GFM1			0.61446	MMGT1			0.17120	FLJ35390	0.51	STAR
		0.35511	C5orf41			0.61566	C15orf23			0.17153	FCRL6	0.511	RORB
		0.35580	PMS2			0.61687	USP10			0.17187	C2orf82	0.512	FAM189B
		0.35650	SRP72			0.61807	ZNF490			0.17221	ZMYM1	0.513	C8orf79
		0.35719	SGIP1			0.61928	KIAA0406			0.17254	LOC100131851	0.514	MTUS2
		0.35789	FAM69A			0.62048	TCTN3			0.17288	FBXL21	0.515	BCL2L2
		0.35858	ZNF639			0.62169	VMA21			0.17321	EEF1B2	0.516	C9orf95

		0.35928	VPS72			0.62289	GOLGA2			0.17355	AKD1	0.517	EEF2K
		0.35997	VWC2L			0.62410	CLTA			0.17388	LRAT	0.518	MYO1B
		0.36067	NOL9			0.62530	METTL9			0.17422	BCL2L15	0.519	TMEM109
		0.36136	FAM91A1			0.62651	CTDSPL2			0.17456	GDF15	0.52	C5orf13
		0.36206	STARD4			0.62771	NTRK3			0.17489	THG1L	0.521	MIAT
		0.36275	ADSS			0.62892	HAUS2			0.17523	CCL20	0.522	SLC32A1
		0.36345	MRPL44			0.63012	NDUFAF1			0.17556	TMEM45A	0.523	MRM1
		0.36414	SRP19			0.63133	RSPRY1			0.17590	BTBD16	0.524	GPR108
		0.36484	SLC35F3			0.63253	DDX42			0.17623	KLRB1	0.525	FAM104B
		0.36553	ACAT2			0.63373	MRPS11			0.17657	FLJ45950	0.526	ACTN4
		0.36623	SLC7A14			0.63494	LOC144438			0.17691	CEP110	0.527	PRC1
		0.36692	GATAD2B			0.63614	MFSD11			0.17724	TPH1	0.528	PARD6A
		0.36762	PANK3			0.63735	GPR12			0.17758	FGF2	0.529	PDP2
		0.36831	PNPLA8			0.63855	GPR12			0.17791	LOC100128507	0.53	CCDC47
		0.36901	DNAJC10			0.63976	MTMR1			0.17825	APAF1	0.531	FBXO32
		0.36970	NUB1			0.64096	RRM1			0.17858	FLJ39095	0.532	LBH
		0.37040	KLHL7			0.64217	YAF2			0.17892	IRX5	0.533	KCNQ4
		0.37109	MRPS10			0.64337	TCP11L1			0.17925	REEP3	0.534	C6orf138
		0.37179	ARL6			0.64458	SRRD			0.17959	BTF3	0.535	PINX1
		0.37248	PTPLB			0.64578	TMEM199			0.17993	TEPP	0.536	C12orf5
		0.37318	DCP1A			0.64699	ADAT1			0.18026	BCL2L15	0.537	MED21
		0.37387	RBM24			0.64819	ZNF483			0.18060	SNRPE	0.538	PHLDB2
		0.37457	C3orf23			0.64940	TRAPPC10			0.18093	TMEM26	0.539	ZBTB34
		0.37526	MBNL1			0.65060	PPIP5K1			0.18127	OR8J3	0.54	LOC388789
		0.37596	MYST2			0.65181	ADNP2			0.18160	LOC100130357	0.541	TARDBP
		0.37665	TTC33			0.65301	CHMP1B			0.18194	NOX1	0.542	C11orf90
		0.37735	RCOR3			0.65422	ZNF264			0.18228	FAM111A	0.543	SPTB
		0.37804	CEP97			0.65542	FAM122A			0.18261	UFM1	0.544	LIMK1
		0.37874	PRKAA2			0.65663	IFT46			0.18295	IQCH	0.545	SLC25A3
		0.37943	USP39			0.65783	SMCR7L			0.18328	C1orf61	0.546	GRIN2A
		0.38013	AKIRIN1			0.65904	RAP2A			0.18362	VWA3B	0.547	YPEL4
		0.38082	WASF1			0.66024	PSMD11			0.18395	C21orf63	0.548	FLJ41278
		0.38151	EXOC1			0.66145	ZNF675			0.18429	CCDC11	0.549	PRKAB1
		0.38221	MTO1			0.66265	TXNDC11			0.18463	IL3	0.55	LOC732096
		0.38290	RGL1			0.66386	UBE2G1			0.18496	PLN	0.551	ULK3
		0.38360	CADM2			0.66506	KLF8			0.18530	GDNF	0.552	UPF0639
		0.38429	SUMF2			0.66627	RPRD1B			0.18563	MAP3K1	0.553	LRRC38
		0.38499	HLTF			0.66747	TMEM66			0.18597	ACSBG2	0.554	ZNF175
		0.38568	C3orf39			0.66867	SPA17			0.18630	CDCA7L	0.555	TRERF1
		0.38638	LYRM7			0.66988	RPGRIP1L			0.18664	SYPL2	0.556	MOSPD1
		0.38707	PEX13			0.67108	UBR7			0.18698	HTR2B	0.557	SLC39A13
		0.38777	TGOLN2			0.67229	TRAPPC5			0.18731	DPH5	0.558	SPNS2

		0.38846	JAZF1			0.67349	ZNRF1			0.18765	DFNB59	0.559	MRPL48
		0.38916	KLF7			0.67470	REPS2			0.18798	C1QTNF9B	0.56	LOC644366
		0.38985	KLRAQ1			0.67590	STYK1			0.18832	SLFN12	0.561	ZNF804A
		0.39055	ARF4			0.67711	DIABLO			0.18865	LOC387771	0.562	C19orf66
		0.39124	MFSD6			0.67831	GSS			0.18899	FLJ35379	0.563	BHLHE22
		0.39194	PDIA6			0.67952	TOX4			0.18933	ALB	0.564	EDNRA
		0.39263	PLK2			0.68072	ZNF134			0.18966	OR11A1	0.565	GTF3A
		0.39333	TADA2B			0.68193	HECTD2			0.19000	DUSP23	0.566	SGCZ
		0.39402	AMACR			0.68313	CCDC56			0.19033	PDCL2	0.567	UBL4A
		0.39472	RABIF			0.68434	MRPS7			0.19067	IL7R	0.568	LOC643988
		0.39541	C1orf43			0.68554	PTPLAD1			0.19100	C12orf48	0.569	C22orf25
		0.39611	GRPEL1			0.68675	FUNDC2			0.19134	DCAF8L2	0.57	CACNA1I
		0.39680	YEATS2			0.68795	DLG2			0.19168	ALDH1A2	0.571	C12orf57
		0.39750	MAK16			0.68916	DNAJA2			0.19201	LOC643441	0.572	ADO
		0.39819	CREB1			0.69036	BEX1			0.19235	SPATA22	0.573	LYSMD4
		0.39889	CDH12			0.69157	NT5DC3			0.19268	DTL	0.574	SAV1
		0.39958	CDO1			0.69277	EEF1A2			0.19302	LOC100129335	0.575	TACR1
		0.40028	MRPS5			0.69398	NAT10			0.19335	C6orf170	0.576	ZNF250
		0.40097	LOC285147			0.69518	SLC8A3			0.19369	CENPC1	0.577	THSD7A
		0.40167	YIPF1			0.69639	CSNK1G1			0.19402	CXCL10	0.578	BID
		0.40236	FAM200B			0.69759	STT3A			0.19436	DDX60L	0.579	MLXIP
		0.40306	ATP6V0B			0.69880	MASTL			0.19470	CCDC102B	0.58	KCNA6
		0.40375	C7orf23			0.70000	SPG21			0.19503	TTC4	0.581	GZF1
		0.40445	NEK10			0.70120	TMEM19			0.19537	GPR64	0.582	PRIM1
		0.40514	CNRIP1			0.70241	ALG11			0.19570	ATXN8OS	0.583	GAD1
		0.40584	GOLPH3L			0.70361	UBA52			0.19604	TSHR	0.584	MLST8
		0.40653	ZNF512			0.70482	CORO1A			0.19637	OR2W1	0.585	MORC2
		0.40723	LMO4			0.70602	TMED2			0.19671	AKAP7	0.586	ZNF774
		0.40792	NAPEPLD			0.70723	TM2D3			0.19705	ZNF665	0.587	UPP1
		0.40862	LYRM7			0.70843	XPOT			0.19738	EED	0.588	VCPIP1
		0.40931	GSTA4			0.70964	SOCS7			0.19772	IYD	0.589	ADRA1D
		0.41001	HTT			0.71084	PPP2R3C			0.19805	BAAT	0.59	ABCA9
		0.41070	PPP1R11			0.71205	ECD			0.19839	ALS2CR11	0.591	NUDT15
		0.41140	DPY30			0.71325	SEC11C			0.19872	TRA@	0.592	DGCR6
		0.41209	C7orf36			0.71446	UROS			0.19906	SLC18A1	0.593	INTS4L1
		0.41279	PURB			0.71566	USE1			0.19940	DDX4	0.594	NOC2L
		0.41348	DHCR24			0.71687	ZBTB33			0.19973	RP2	0.595	SBNO1
		0.41418	INTS7			0.71807	RRN3			0.20007	RAD51L1	0.596	GNG13
		0.41487	LOC100133669			0.71928	NDN			0.20040	ANKDD1B	0.597	GIT2
		0.41557	DUSP11			0.72048	CRYM			0.20074	BRCA2	0.598	NRGN
		0.41626	UAP1			0.72169	DCUN1D3			0.20107	CREB1	0.599	DNMT1
		0.41696	SLC37A3			0.72289	RNF126			0.20141	SLC25A24	0.6	C17orf86

		0.41765	C22orf28			0.72410	DDX47			0.20175	GIMAP7	0.601	RND1
		0.41835	ENOPH1			0.72530	METTL4			0.20208	IKIP	0.602	OSBPL2
		0.41904	CTSB			0.72651	SPTLC1			0.20242	C1orf50	0.603	IGLON5
		0.41974	MYSM1			0.72771	METTL10			0.20275	LOC286189	0.604	ZNF574
		0.42043	MBD5			0.72892	SMAD2			0.20309	KCNU1	0.605	C15orf61
		0.42113	C6orf153			0.73012	ARMCX3			0.20342	LOC100291860	0.606	ATP5O
		0.42182	PGBD5			0.73133	LOC100288114			0.20376	RPE65	0.607	GNAS
		0.42252	RPS23			0.73253	C16orf87			0.20410	LOC100131431	0.608	COX11
		0.42321	PTP4A1			0.73373	SORCS3			0.20443	ASIP	0.609	AS3MT
		0.42391	CIAO1			0.73494	ADK			0.20477	TRMT61A	0.61	MBNL2
		0.42460	CDC42BPA			0.73614	UBE2I			0.20510	FLJ37396	0.611	TMEM196
		0.42530	CEP68			0.73735	DNAJA4			0.20544	DNAH12	0.612	TRERF1
		0.42599	UBE2H			0.73855	C9orf123			0.20577	GAFA3	0.613	LOC113230
		0.42669	RNF11			0.73976	KATNAL1			0.20611	IL20RA	0.614	ONECUT2
		0.42738	NUP205			0.74096	SUPT6H			0.20645	LOC728597	0.615	LOC646762
		0.42808	TNPO1			0.74217	SNW1			0.20678	TLE4	0.616	SMPX
		0.42877	HNRPLL			0.74337	GLE1			0.20712	RPL6	0.617	WDR18
		0.42946	AKR1A1			0.74458	EIF2B1			0.20745	CLEC4A	0.618	C2CD3
		0.43016	UNC80			0.74578	WBP11			0.20779	CENPI	0.619	SPIRE2
		0.43085	DDX46			0.74699	ZDHHC6			0.20812	EGF	0.62	RBMS1
		0.43155	UBR4			0.74819	FSD1L			0.20846	FAM72A	0.621	CKB
		0.43224	GRIA2			0.74940	TXN2			0.20879	FREM1	0.622	DGAT2
		0.43294	NLGN1			0.75060	ARHGEF12			0.20913	CHEK1	0.623	PEPD
		0.43363	AFF3			0.75181	ALDH3A2			0.20947	C4orf21	0.624	RNF148
		0.43433	TXNDC12			0.75301	SLC2A14			0.20980	BLM	0.625	CDKN1B
		0.43502	TNKS			0.75422	SIGMAR1			0.21014	ASCL2	0.626	ZNF124
		0.43572	CCRN4L			0.75542	X3258092			0.21047	LOC100128590	0.627	OBFC1
		0.43641	LRBA			0.75663	MAPKAP1			0.21081	DBF4	0.628	PTGIS
		0.43711	MEA1			0.75783	MACROD2			0.21114	ANKHD1	0.629	CORO6
		0.43780	RNF180			0.75904	ZNF529			0.21148	FBXO15	0.63	QRFPR
		0.43850	ZRANB3			0.76024	FSD1L			0.21182	LOC120824	0.631	SFRS2B
		0.43919	TXNDC15			0.76145	PDRG1			0.21215	DNAH14	0.632	RPS28
		0.43989	HMGCLL1			0.76265	SNTB2			0.21249	AFARP1	0.633	RAI1
		0.44058	GYG1			0.76386	GOSR2			0.21282	ZUFSP	0.634	NAALAD2
		0.44128	PIK3R1			0.76506	PTPRJ			0.21316	MAF1	0.635	GALE
		0.44197	RPE			0.76627	ZNF32			0.21349	PCBD2	0.636	NTN4
		0.44267	MRPL18			0.76747	EIF3J			0.21383	PRIM2	0.637	PTPRD
		0.44336	RPN1			0.76867	LOC728392			0.21417	ETV1	0.638	SMAD7
		0.44406	HIGD1A			0.76988	MTMR7			0.21450	LAMA3	0.639	FOSL2
		0.44475	RRP15			0.77108	SAMD12			0.21484	DAPL1	0.64	LRRC37A4
		0.44545	ARL15			0.77229	FHL1			0.21517	VANGL1	0.641	PNMAL2
		0.44614	CDH10			0.77349	NSFL1C			0.21551	TAAR1	0.642	CKMT1B

		0.44684	NEU1			0.77470	GLCE			0.21584	G3BP1	0.643	CHRNA2
		0.44753	FAM120B			0.77590	PTPN9			0.21618	SAMD13	0.644	KLHL28
		0.44823	MED8			0.77711	CD2BP2			0.21652	MST4	0.645	ST3GAL2
		0.44892	MBD4			0.77831	MRPS6			0.21685	TAAR3	0.646	MMP24
		0.44962	SLC4A7			0.77952	THUMPD1			0.21719	CDC7	0.647	IGF1R
		0.45031	CSRNP3			0.78072	EIF4A1P4			0.21752	LOC100128788	0.648	COQ10A
		0.45101	KIAA0232			0.78193	C10orf58			0.21786	IFLTD1	0.649	TCF7L2
		0.45170	YIPF5			0.78313	PSMB7			0.21819	LOC283711	0.65	GPATCH8
		0.45240	CCDC104			0.78434	DACH2			0.21853	MDP1	0.651	HDAC9
		0.45309	FAM102B			0.78554	KIAA1704			0.21887	SNORA33	0.652	UVRAG
		0.45379	PCDHB15			0.78675	XPNPEP1			0.21920	MSR1	0.653	SEMA4G
		0.45448	ORC3L			0.78795	DUS2L			0.21954	LOC728003	0.654	CYTSA
		0.45518	NLN			0.78916	CRNKL1			0.21987	TMC7	0.655	TTLL11
		0.45587	RUVBL1			0.79036	ZNF583			0.22021	RASA4	0.656	IFFO1
		0.45657	ASTN1			0.79157	RAE1			0.22054	GAFA2	0.657	CHCHD4
		0.45726	BAT2D1			0.79277	LOC92249			0.22088	PRG4	0.658	KIAA1267
		0.45796	PCDHB12			0.79398	AMZ2			0.22122	LOC728316	0.659	LOC100288047
		0.45865	C2orf55			0.79518	MRPS16			0.22155	TACR3	0.66	TXK
		0.45935	UROD			0.79639	GALNT1			0.22189	UTP20	0.661	FCRLB
		0.46004	ZNF330			0.79759	FN3KRP			0.22222	HSF2	0.662	TRPM3
		0.46074	GBA			0.79880	ABCB7			0.22256	KRTAP7-1	0.663	CADPS2
		0.46143	UFSP2			0.80000	G2E3			0.22289	CLRN1	0.664	CDH7
		0.46213	HMGXB3			0.80120	CREB3			0.22323	LIN9	0.665	C12orf5
		0.46282	CAPZA2			0.80241	SFRS2B			0.22356	KATNAL2	0.666	SETBP1
		0.46352	PTBP2			0.80361	VPS29			0.22390	C10orf4	0.667	PLAUR
		0.46421	SLBP			0.80482	C10orf57			0.22424	RPS6	0.668	PLEKHA1
		0.46491	TMEM70			0.80602	PTS			0.22457	NACAP1	0.669	MPPED1
		0.46560	ALDH5A1			0.80723	SH3KBP1			0.22491	FAM170A	0.67	RABEPK
		0.46630	ACTR8			0.80843	VPS4B			0.22524	ST6GALNAC3	0.671	ZRANB1
		0.46699	PCDHB13			0.80964	PSMB3			0.22558	LIMS1	0.672	DDX54
		0.46769	C1orf52			0.81084	SPRYD4			0.22591	CAPN13	0.673	WHAMM
		0.46838	PRKAB2			0.81205	LCOR			0.22625	ZNF468	0.674	JTB
		0.46908	TBC1D23			0.81325	TMEM147			0.22659	JUB	0.675	GJC1
		0.46977	RPF1			0.81446	CTSF			0.22692	BEND3	0.676	NOS2
		0.47047	KRIT1			0.81566	TMEM205			0.22726	SPATA5L1	0.677	CHAF1A
		0.47116	MORN2			0.81687	C14orf166			0.22759	ACRC	0.678	ANKRD2
		0.47186	RAF1			0.81807	FBXL3			0.22793	MORC4	0.679	FLJ39303
		0.47255	DEDD			0.81928	SIN3A			0.22826	C3orf67	0.68	SPIRE1
		0.47325	MRPS15			0.82048	C9orf40			0.22860	ITLN1	0.681	RND2
		0.47394	RCHY1			0.82169	CSTF3			0.22894	AGBL3	0.682	TSHZ3
		0.47464	TTC13			0.82289	C10orf84			0.22927	DNAJB7	0.683	NRIP1
		0.47533	PRICKLE4			0.82410	GSTO2			0.22961	PDE5A	0.684	GPX3

		0.47603	EIF4E2			0.82530	PEX11G			0.22994	HPS5	0.685	ADCY8
		0.47672	UTP23			0.82651	ATP7A			0.23028	C8orf58	0.686	FRMPD1
		0.47741	SNAPIN			0.82771	LOC642345			0.23061	RGS18	0.687	TBC1D16
		0.47811	SUCLG1			0.82892	ZDHHC7			0.23095	LOC284669	0.688	SS18L1
		0.47880	TMEM69			0.83012	SAMHD1			0.23129	RFX6	0.689	RARA
		0.47950	HIGD2A			0.83133	DIS3L			0.23162	ZNF764	0.69	RAB40C
		0.48019	RPS6P6			0.83253	TMEM86A			0.23196	LOC100130744	0.691	SGK494
		0.48089	PPP1CB			0.83373	MRPL40			0.23229	ASB12	0.692	WDR46
		0.48158	MUT			0.83494	ZRANB1			0.23263	ANKRD39	0.693	RGAG4
		0.48228	HNRNPR			0.83614	CALN1			0.23296	FMO5	0.694	RBMS1
		0.48297	FUBP1			0.83735	PSMD10			0.23330	LOC647252	0.695	TADA2A
		0.48367	CSNK1A1			0.83855	STAU1			0.23364	LOC100216479	0.696	LOC100127950
		0.48436	PRCC			0.83976	TMEM68			0.23397	LOC100132686	0.697	OLR1
		0.48506	CDC40			0.84096	MRPL49			0.23431	FAM129A	0.698	PPARGC1B
		0.48575	PEX19			0.84217	C20orf43			0.23464	PAXIP1	0.699	HSD17B1
		0.48645	RASA2			0.84337	LOC647979			0.23498	CYP4Z2P	0.7	KCNK12
		0.48714	NUDT3			0.84458	DCP1B			0.23531	C15orf51	0.701	KCNS3
		0.48784	CHMP2B			0.84578	CALB1			0.23565	FBXO5	0.702	DCBLD1
		0.48853	TMEM167A			0.84699	NUDT5			0.23599	ANO2	0.703	ZNF821
		0.48923	ODF2L			0.84819	NUDT2			0.23632	CASP8	0.704	ALKBH1
		0.48992	PCBP1			0.84940	SNRPB2			0.23666	ZNF678	0.705	SEMA6D
		0.49062	RLF			0.85060	C8orf76			0.23699	C4orf43	0.706	RASGRP2
		0.49131	LOC100130998			0.85181	ANXA11			0.23733	TNFRSF10D	0.707	SFXN4
		0.49201	BOD1			0.85301	DNAJC14			0.23766	OR1N2	0.708	LOC339290
		0.49270	C7orf44			0.85422	NCAM2			0.23800	PDC	0.709	TMEM132C
		0.49340	CAMLG			0.85542	ZRANB1			0.23834	GSTCD	0.71	DHRS7
		0.49409	TXNDC9			0.85663	KATNAL1			0.23867	ZNF726	0.711	TSHZ1
		0.49479	SELT			0.85783	MMP16			0.23901	CC2D2B	0.712	GRID2IP
		0.49548	ZNF518B			0.85904	HTR3B			0.23934	ARL13A	0.713	BTBD11
		0.49618	SMURF1			0.86024	TMEM14D			0.23968	EYS	0.714	RGS2
		0.49687	DNAJB11			0.86145	RPL27A			0.24001	LOC400940	0.715	TCTEX1D1
		0.49757	PDE6D			0.86265	LOC338758			0.24035	ISY1	0.716	LOC100131654
		0.49826	C2orf44			0.86386	DNAJC15			0.24068	BCL2L10	0.717	FUS
		0.49896	SRD5A3			0.86506	PTGS2			0.24102	EIF3F	0.718	LOC284219
		0.49965	KHDRBS3			0.86627	TMEM107			0.24136	CCT6B	0.719	GOLGA7B
		0.50035	PPM1G			0.86747	DGKA			0.24169	RRN3P1	0.72	ZNF383
		0.50104	STIM2			0.86867	ZNF331			0.24203	DOPEY1	0.721	CSTF2T
		0.50174	HIST1H4E			0.86988	TOM1L1			0.24236	MCEE	0.722	SEC16A
		0.50243	ADIPOR1			0.87108	THNSL1			0.24270	LOC100129697	0.723	AP1S3
		0.50313	MXD1			0.87229	LYRM1			0.24303	CCDC15	0.724	ALKBH3
		0.50382	PGRMC2			0.87349	PCDH20			0.24337	LOC148638	0.725	MCRS1
		0.50452	KLHDC10			0.87470	RDH11			0.24371	CASP12	0.726	SBNO1

		0.50521	AGPS			0.87590	FXN			0.24404	CLIC2	0.727	ZBTB16
		0.50591	C2orf49			0.87711	ZNF84			0.24438	LETM2	0.728	ANKRD9
		0.50660	ENY2			0.87831	IDI1			0.24471	TMEM194B	0.729	C10orf140
		0.50730	NECAB1			0.87952	TNFAIP8L1			0.24505	C1QTNF6	0.73	UQCRB
		0.50799	ZNF445			0.88072	MAP2K5			0.24538	KRT20	0.731	SPHKAP
		0.50869	BOD1L			0.88193	RNF185			0.24572	LOC646214	0.732	CTSZ
		0.50938	ACBD3			0.88313	RAB39			0.24606	PMFBP1	0.733	TMEM169
		0.51008	RNF7			0.88434	MEX3C			0.24639	CCDC110	0.734	PPIF
		0.51077	FAM13A			0.88554	URB1			0.24673	RPS20P27	0.735	AIFM3
		0.51147	ORMDL1			0.88675	KLF6			0.24706	CAV2	0.736	PHACTR2
		0.51216	GNPDA1			0.88795	SOCS2			0.24740	LOC644714	0.737	HAGH
		0.51286	EIF2B3			0.88916	C11orf57			0.24773	FLJ35776	0.738	FAM110A
		0.51355	COG5			0.89036	C9orf41			0.24807	ATAD5	0.739	IER5L
		0.51425	EPHA5			0.89157	C16orf13			0.24841	HLA-L	0.74	ZBBX
		0.51494	KLHL8			0.89277	VTI1A			0.24874	LOC151658	0.741	LOC346702
		0.51564	PCLO			0.89398	ALG10			0.24908	KIF11	0.742	GATAD2A
		0.51633	C1orf96			0.89518	TMEM2			0.24941	CDKL3	0.743	CPSF1
		0.51703	SEC62			0.89639	ARD1A			0.24975	RP11-90M2.3	0.744	NOM1
		0.51772	CLIP4			0.89759	KCNMB4			0.25008	FKBP15	0.745	ZSCAN29
		0.51842	INSIG1			0.89880	C12orf32			0.25042	MYEF2	0.746	ALG3
		0.51911	ARSB			0.90000	CHURC1			0.25076	COL28A1	0.747	LEO1
		0.51981	ICA1L			0.90120	TRIP11			0.25109	NOC3L	0.748	RASL10B
		0.52050	RFC2			0.90241	AKAP5			0.25143	GNGT1	0.749	BMP8A
		0.52120	DARC			0.90361	ZNF506			0.25176	NHEDC1	0.75	SULF1
		0.52189	RFWD2			0.90482	ZBED5			0.25210	BNC2	0.751	ETHE1
		0.52259	KLHL12			0.90602	YWHAQP8			0.25243	SMC4	0.752	SPATA2
		0.52328	LOC285378			0.90723	ZNF43			0.25277	SUMO4	0.753	GAL
		0.52397	RPL15			0.90843	ZNF264			0.25311	PHF11	0.754	LOC221710
		0.52467	TMEM115			0.90964	GPN3			0.25344	LOC728086	0.755	ACTR6
		0.52536	FAM54B			0.91084	ZNF189			0.25378	C7orf63	0.756	MRPL33
		0.52606	ANAPC4			0.91205	C9orf21			0.25411	ZNF569	0.757	GSTT1
		0.52675	AMD1			0.91325	FAM192A			0.25445	ZNF566	0.758	ARMCX5
		0.52745	C1orf107			0.91446	LOC729680			0.25478	FBXO31	0.759	CRABP1
		0.52814	HCG18			0.91566	ZDHHC24			0.25512	ATF7IP	0.76	ZNF230
		0.52884	LMAN2			0.91687	EIF4A1			0.25545	RNF32	0.761	LOC285771
		0.52953	SEC22A			0.91807	RFX7			0.25579	ZNF561	0.762	C17orf63
		0.53023	THUMPD3			0.91928	RAB30			0.25613	GALNT8	0.763	C1orf133
		0.53092	TMEM170B			0.92048	C19orf10			0.25646	ZFP112	0.764	SPSB3
		0.53162	KLHL24			0.92169	NR4A3			0.25680	ZNF107	0.765	AURKA
		0.53231	TMEM178			0.92289	HEBP1			0.25713	C3orf77	0.766	INSR
		0.53301	NPRL2			0.92410	DHRS7B			0.25747	DNA2	0.767	TSPAN9
		0.53370	DYNC2LI1			0.92530	GABARAP			0.25780	CKLF	0.768	KCNH4

0.53440	ANAPC13	0.92651	TRMT5	0.25814	LOC100289409	0.769	GOLGA8A
0.53509	STX7	0.92771	SMCHD1	0.25848	TTC18	0.77	ENTPD7
0.53579	FOXO3	0.92892	IPMK	0.25881	PTPRQ	0.771	SLC25A16
0.53648	TAPT1	0.93012	LOC100294416	0.25915	PHF13	0.772	HIST1H4B
0.53718	HDAC3	0.93133	ERCC4	0.25948	RORA	0.773	MIF
0.53787	SLC30A7	0.93253	SUV39H1	0.25982	MTL5	0.774	ASPSCR1
0.53857	ACVR2A	0.93373	ZNF627	0.26015	C12orf28	0.775	KIAA1731
0.53926	LARP1B	0.93494	TP53RK	0.26049	CCDC148	0.776	KBTBD7
0.53996	PHF7	0.93614	C14orf135	0.26083	LOC644196	0.777	FLJ10038
0.54065	ARSK	0.93735	XPO4	0.26116	C10orf11	0.778	MMP15
0.54135	TOR1AIP2	0.93855	SNX11	0.26150	NFIB	0.779	NUDT22
0.54204	C8orf46	0.93976	SLC15A4	0.26183	LSM14A	0.78	PDE7A
0.54274	TSC22D2	0.94096	ETFA	0.26217	ZNF525	0.781	FAM73B
0.54343	PRPF4B	0.94217	RPS6KA5	0.26250	KIAA0040	0.782	URM1
0.54413	MIER3	0.94337	ZNF808	0.26284	LEPREL1	0.783	ANTXR2
0.54482	GALNT11	0.94458	SPRY2	0.26318	COL21A1	0.784	ZNF223
0.54552	EIF2AK3	0.94578	C15orf41	0.26351	LMNB1	0.785	SPTBN4
0.54621	CDK11A	0.94699	GPKOW	0.26385	GPR111	0.786	RCN3
0.54691	FOXP1	0.94819	TMLHE	0.26418	RNF217	0.787	COX7A1
0.54760	NIT1	0.94940	ANP32A	0.26452	LOC284561	0.788	ARL2
0.54830	GPR85	0.95060	DKFZP779L1853	0.26485	RAET1L	0.789	KIAA1217
0.54899	HNRNPD	0.95181	LOC147727	0.26519	COX8C	0.79	ATPAF2
0.54969	YWHAQ	0.95301	DOLPP1	0.26553	HSPC157	0.791	MED17
0.55038	SGIP1	0.95422	CCDC102B	0.26586	CCDC150	0.792	SPTB
0.55108	MEF2D	0.95542	SYNM	0.26620	C18orf56	0.793	GMPR
0.55177	SFRS13B	0.95663	ZNF681	0.26653	NYNRIN	0.794	ABTB1
0.55247	SATB1	0.95783	EMG1	0.26687	FAM18A	0.795	VWC2L
0.55316	TAS2R4	0.95904	ANKK1	0.26720	GTPBP4	0.796	ATP6V0A2
0.55386	SRGAP2	0.96024	ZNF75A	0.26754	LOC400986	0.797	BEAN
0.55455	MRPL53	0.96145	VLDLR	0.26788	LOC728218	0.798	ATAD2
0.55525	OR2L13	0.96265	C9orf156	0.26821	STAC3	0.799	C19orf43
0.55594	TMEM17	0.96386	LOC387647	0.26855	HAUS6	0.8	MLLT10
0.55664	SYNPO2	0.96506	ABHD2	0.26888	RNFT1	0.801	HSPA8
0.55733	TMEM170B	0.96627	C9orf78	0.26922	LOC100131512	0.802	HMBS
0.55803	GET4	0.96747	DPM2	0.26955	CDC25C	0.803	EPHB2
0.55872	PLEKHA2	0.96867	C8ORFK29	0.26989	C12orf26	0.804	TMEM223
0.55942	CNOT4	0.96988	ZNF549	0.27022	RPL23	0.805	LOC100288839
0.56011	KIAA0114	0.97108	HAUS7	0.27056	LOC339529	0.806	C16orf68
0.56081	ZBTB41	0.97229	C12orf64	0.27090	C9orf57	0.807	TAS2R50
0.56150	COX5B	0.97349	LOC730183	0.27123	TBL1Y	0.808	COL5A2
0.56220	SPCS3	0.97470	SAMD8	0.27157	PRL	0.809	ISOC2
0.56289	FAM162A	0.97590	C10orf26	0.27190	MCOLN3	0.81	ROPN1L

		0.56359	C1orf123			0.97711	NOX4			0.27224	WDR49	0.811	QRICH2
		0.56428	UBAP2L			0.97831	ATP8B3			0.27257	ZNF669	0.812	C7orf43
		0.56498	PCDHB19P			0.97952	PRSS3			0.27291	BTN3A3	0.813	DOM3Z
		0.56567	RIOK2			0.98072	SMAD4			0.27325	NCRNA00032	0.814	SPAG4
		0.56637	IMP4			0.98193	C12orf64			0.27358	ZNF420	0.815	LOC100128239
		0.56706	ZNF718			0.98313	LOC100288738			0.27392	SELL	0.816	RIPPLY2
		0.56776	C8orf44			0.98434	C12orf64			0.27425	ZNF596	0.817	RPS6KB1
		0.56845	OAZ1			0.98554	LOC254100			0.27459	GAGE1	0.818	DENND4B
		0.56915	SMARCAL1			0.98675	TEX10			0.27492	HNRNPC	0.819	MAP2K7
		0.56984	C1orf103			0.98795	LOC284014			0.27526	LOC728723	0.82	BRWD3
		0.57054	LRPAP1			0.98916	TMEM117			0.27560	IQCD	0.821	ABCD2
		0.57123	LSAMP			0.99036	C10orf12			0.27593	PTCH1	0.822	NT5C1A
		0.57192	HMGN4			0.99157	DET1			0.27627	ATP5S	0.823	CSMD2
		0.57262	SSR1			0.99277	LOC440040			0.27660	GYPE	0.824	LOC100134018
		0.57331	PSMB2			0.99398	HTATSF1			0.27694	PAX6	0.825	ING1
		0.57401	CTNNB1			0.99518	ALG13			0.27727	C9orf11	0.826	TYRP1
		0.57470	PRKAA2			0.99639	DCAF7			0.27761	PEX2	0.827	CCBL1
		0.57540	STT3B			0.99759	LOC283143			0.27795	TNFSF13B	0.828	CAPS2
		0.57609	SATB1			0.99880	MAX			0.27828	ANUBL1	0.829	STX1B
		0.57679	PHACTR1							0.27862	STAP1	0.83	SNX21
		0.57748	PAK1IP1							0.27895	VPS54	0.831	ELL
		0.57818	PPIL4							0.27929	FAM167B	0.832	APBA2
		0.57887	C6orf203							0.27962	TMEM126B	0.833	POU3F1
		0.57957	FEM1C							0.27996	MURC	0.834	PPP1R3F
		0.58026	HOMER1							0.28030	PRTG	0.835	RDH13
		0.58096	SESTD1							0.28063	LOC100289565	0.836	BLOC1S3
		0.58165	C4orf50							0.28097	ABCF1	0.837	ZNF774
		0.58235	PPAP2A							0.28130	C1orf69	0.838	PKDCC
		0.58304	MAP3K4							0.28164	SNRPC	0.839	C12orf62
		0.58374	TUBB							0.28197	C13orf38	0.84	EIF4G2
		0.58443	UBE2B							0.28231	CDC23	0.841	SRRM3
		0.58513	KIAA1109							0.28265	KIAA1009	0.842	SLC5A6
		0.58582	RAB1A							0.28298	SPINK6	0.843	DRP2
		0.58652	AAMP							0.28332	USP50	0.844	C20orf20
		0.58721	SSB							0.28365	ST7L	0.845	EPN3
		0.58791	BAI3							0.28399	SLC22A4	0.846	KGFLP2
		0.58860	LOC100129550							0.28432	NKX1-2	0.847	FAM19A4
		0.58930	EPM2AIP1							0.28466	RRP15	0.848	RASL11A
		0.58999	UFC1							0.28499	LOC100129935	0.849	PTGS1
		0.59069	C2orf42							0.28533	CCDC62	0.85	ABCA6
		0.59138	COMMD1							0.28567	CHD1	0.851	GOLGA8B
		0.59208	DUSP19							0.28600	CLEC14A	0.852	KLHL29

		0.59277	ZNF454						0.28634	HSD17B7P2	0.853	LOC400553
		0.59347	POLR1B						0.28667	SNHG12	0.854	LOC283713
		0.59416	ANAPC1						0.28701	LOC641515	0.855	LOC100132077
		0.59486	PARP8						0.28734	CXorf56	0.856	MTUS2
		0.59555	UTP11L						0.28768	BET1	0.857	C17orf89
		0.59625	CALM2						0.28802	C1orf135	0.858	C2CD2
		0.59694	ECT2						0.28835	ASB8	0.859	IER2
		0.59764	RQCD1						0.28869	ZNF573	0.86	PENK
		0.59833	SPATS2L						0.28902	COPZ2	0.861	VPS18
		0.59903	LRP11						0.28936	CROCC	0.862	C1QTNF4
		0.59972	YOD1						0.28969	X3678343	0.863	SETD6
		0.60042	DCP2						0.29003	C14orf38	0.864	PRPSAP1
		0.60111	CXXC5						0.29037	DENND2D	0.865	PLEKHA8
		0.60181	TRA2B						0.29070	PROKR2	0.866	PSMC5
		0.60250	ZNF643						0.29104	ST7OT2	0.867	MOB2
		0.60320	RRS1						0.29137	OR51S1	0.868	ZNF830
		0.60389	SGCB						0.29171	AKAP14	0.869	AKAP8
		0.60459	THRAP3						0.29204	TFAMP1	0.87	C6orf176
		0.60528	C1orf190						0.29238	POM121L2	0.871	NRBF2
		0.60598	SEC62						0.29272	SGOL2	0.872	PLD5
		0.60667	ISG20L2						0.29305	DISC1	0.873	GREM2
		0.60737	ZNF354A						0.29339	ZC3H8	0.874	BEND7
		0.60806	WRNIP1						0.29372	TMEM50A	0.875	ZNF213
		0.60876	HSD17B11						0.29406	ZNF700	0.876	MID2
		0.60945	PEX5L						0.29439	ASCL3	0.877	RP9
		0.61015	LRWD1						0.29473	ZNF625	0.878	BOLA3
		0.61084	CDKN2AIPNL						0.29507	RPIA	0.879	XKR6
		0.61154	RG9MTD1						0.29540	TMEM84	0.88	ACRV1
		0.61223	BZW1						0.29574	USP49	0.881	RTCD1
		0.61293	PCDHB3						0.29607	FLJ30901	0.882	MTHFD2
		0.61362	IPO9						0.29641	LOC100129489	0.883	USP6NL
		0.61432	MTF2						0.29674	MYB	0.884	ANKRD5
		0.61501	GPN2						0.29708	DNASE1	0.885	CORO7
		0.61571	TMEM170B						0.29742	ZNF121	0.886	SLC16A10
		0.61640	C2orf64						0.29775	FANCM	0.887	C9orf69
		0.61710	C2orf74						0.29809	SEL1L2	0.888	PLEKHH2
		0.61779	UST						0.29842	PPP1R1C	0.889	ABHD12B
		0.61849	ETF1						0.29876	NRK	0.89	C1RL
		0.61918	TATDN2						0.29909	TIGD7	0.891	NEDD9
		0.61987	HPS3						0.29943	SOHLH2	0.892	PLK5P
		0.62057	PPM1K						0.29977	LOC388387	0.893	KCNJ9
		0.62126	FNIP2						0.30010	KNTC1	0.894	RPS19BP1

0.62196	EIF4BP8	0.30044	OR10C1	0.895	ZFHX2
0.62265	LOC100302640	0.30077	PIGT	0.896	MAN2C1
0.62335	ADCK2	0.30111	POLQ	0.897	OPRL1
0.62404	KIAA0226	0.30144	SLCO5A1	0.898	FKRP
0.62474	YTHDF2	0.30178	GRHL1	0.899	CHST8
0.62543	PFDN2	0.30211	KMO	0.9	KIAA0664P3
0.62613	GPR63	0.30245	BCCIP	0.901	SLC24A4
0.62682	CPLX2	0.30279	LOC100287689	0.902	DIDO1
0.62752	ACOT13	0.30312	LMOD1	0.903	C16orf46
0.62821	WDR3	0.30346	TTK	0.904	SPTBN4
0.62891	ZNF281	0.30379	X3051800	0.905	TMEM160
0.62960	LOC100129518	0.30413	HIST1H2BL	0.906	ATP6V0A2
0.63030	QARS	0.30446	ZNF138	0.907	REPIN1
0.63099	KARS	0.30480	GPR15	0.908	HSPB6
0.63169	C8orf42	0.30514	ERI1	0.909	C12orf5
0.63238	PSMA2	0.30547	METTL12	0.91	ARHGEF33
0.63308	AKIRIN2	0.30581	UBXN2A	0.911	ZC4H2
0.63377	FAM5B	0.30614	LOC441666	0.912	SNN
0.63447	NOL7	0.30648	ST6GALNAC4	0.913	FSTL1
0.63516	SLMAP	0.30681	TRAM1L1	0.914	ZNF641
0.63586	ING3	0.30715	C21orf81	0.915	PCDHB19P
0.63655	DNTTIP2	0.30749	RPTN	0.916	LOC441426
0.63725	FAM92A1	0.30782	HOXA4	0.917	BET1L
0.63794	CNNM4	0.30816	EPCAM	0.918	ZSWIM4
0.63864	SEC62	0.30849	SNORD16	0.919	SLC39A7
0.63933	IGSF21	0.30883	GPX6	0.92	PNOC
0.64003	SFRS7	0.30916	DDX55	0.921	FRS3
0.64072	TRPC1	0.30950	LOC389493	0.922	CEP192
0.64142	TMEM170B	0.30984	MRGPRX4	0.923	DIDO1
0.64211	DCP1A	0.31017	MED18	0.924	KREMEN1
0.64281	DNAJC19	0.31051	SEPHS1	0.925	AKR1B1
0.64350	ZFYVE20	0.31084	FLJ20444	0.926	NOB1
0.64420	TAF1B	0.31118	FAM194A	0.927	PIGL
0.64489	THOC7	0.31151	C19orf56	0.928	SEMA4G
0.64559	BPGM	0.31185	ZNF215	0.929	CALCOCO1
0.64628	CBR4	0.31219	TMEM45B	0.93	LOC100129936
0.64698	SSR3	0.31252	SCUBE2	0.931	COL19A1
0.64767	PTGFRN	0.31286	NID2	0.932	TAF3
0.64837	PCDHB19P	0.31319	MTBP	0.933	TLL2
0.64906	PRKRA	0.31353	C1orf158	0.934	CATSPER2
0.64976	KHDRBS1	0.31386	KLRC4	0.935	SLC25A42
0.65045	MRFAP1	0.31420	WNT8A	0.936	SLC20A2

		0.65115	CNOT8						0.31454	C5orf36	0.937	HPCAL1
		0.65184	LARP1						0.31487	ZNF829	0.938	PEX12
		0.65254	SEMA5B						0.31521	GABPA	0.939	SEL1L3
		0.65323	KIF1B						0.31554	DNAJC12	0.94	ODF2
		0.65393	CALN1						0.31588	PMM2	0.941	C17orf91
		0.65462	ALS2CR4						0.31621	ARHGEF35	0.942	LOC642947
		0.65532	HCG18						0.31655	CRYGA	0.943	LCP2
		0.65601	METTL6						0.31688	ENO3	0.944	PRICKLE1
		0.65671	FSCN1						0.31722	RPL34	0.945	SEMA3C
		0.65740	RRAGD						0.31756	ZNF789	0.946	CTXN3
		0.65810	LYRM7						0.31789	ZNF77	0.947	KCNRG
		0.65879	C7orf54						0.31823	SETD8	0.948	GTF2H2B
		0.65949	ADCY3						0.31856	ZNF404	0.949	SCRN2
		0.66018	STK25						0.31890	GCOM1	0.95	TIRAP
		0.66088	TSN						0.31923	ILKAP	0.951	SEC16A
		0.66157	TMEM14C						0.31957	C6orf141	0.952	RPL17
		0.66227	BCL9						0.31991	TEX14	0.953	SPATS1
		0.66296	NSD1						0.32024	CCDC112	0.954	F13A1
		0.66366	KCNF1						0.32058	C9orf84	0.955	ZNF284
		0.66435	PRUNE						0.32091	EEF1A1	0.956	DIDO1
		0.66505	DGKB						0.32125	LOC344328	0.957	LOC144571
		0.66574	RC3H1						0.32158	SPAM1	0.958	MRPL11
		0.66644	SFRS3						0.32192	LOC100128477	0.959	COL6A1
		0.66713	PEF1						0.32226	C6orf130	0.96	ADM
		0.66782	MRPS36						0.32259	LOC100288937	0.961	REEP6
		0.66852	FAM40A						0.32293	GPR126	0.962	KDELR3
		0.66921	REPS1						0.32326	FAM69A	0.963	C20orf74
		0.66991	NUPL2						0.32360	FNTA	0.964	KLHL28
		0.67060	SNRNP27						0.32393	TXLNB	0.965	DUSP1
		0.67130	C14orf129						0.32427	ASTE1	0.966	CCDC57
		0.67199	LRRC47						0.32461	COMMD3	0.967	ZNF222
		0.67269	LASS6						0.32494	LOC645752	0.968	RPL36AP33
		0.67338	SDHC						0.32528	OR4X1	0.969	CCDC86
		0.67408	C1orf230						0.32561	ALS2	0.97	SELO
		0.67477	HEATR1						0.32595	LOC613126	0.971	WIBG
		0.67547	RABL3						0.32628	FLJ37201	0.972	TAF11
		0.67616	HIVEP1						0.32662	PACS2	0.973	FAM163A
		0.67686	CHST10						0.32696	ANKRD32	0.974	BRWD3
		0.67755	TMEM56						0.32729	FANCI	0.975	LOC121952
		0.67825	SUPT3H						0.32763	LIMS1	0.976	EFTUD1
		0.67894	PROSC						0.32796	ICA1L	0.977	LGALS1
		0.67964	IQCB1						0.32830	LOC727947	0.978	HIGD1B

		0.68033	ZNF238						0.32863	C14orf147	0.979	GRM4
		0.68103	SLC30A6						0.32897	CASP14	0.98	LOC100131000
		0.68172	TAF9						0.32931	CYP2U1	0.981	PCDHB19P
		0.68242	MMP16						0.32964	NSMCE4A	0.982	LOC157278
		0.68311	PCDHB18						0.32998	LOC729177	0.983	FAM123B
		0.68381	STMN1						0.33031	MCM3APAS	0.984	LOC283585
		0.68450	CCT7						0.33065	CCDC141	0.985	TOM1L2
		0.68520	LOC100133211						0.33098	CORIN	0.986	ANKS1A
		0.68589	SPOCK1						0.33132	ZAP70	0.987	ANKRD20A5
		0.68659	LOC493754						0.33165	C21orf49	0.988	HSPA1B
		0.68728	DRD1						0.33199	ZNF678	0.989	PCDH11X
		0.68798	VDAC3						0.33233	LOC441167	0.99	FLJ45508
		0.68867	ARHGAP29						0.33266	ALDH3B2	0.991	ZSCAN5A
		0.68937	NUDT12						0.33300	SCN7A	0.992	SGK3
		0.69006	POLB						0.33333	BANP	0.993	CKMT1A
		0.69076	LOC222159						0.33367	KRTCAP2	0.994	MLXIPL
		0.69145	NME7						0.33400	C2orf63	0.995	MED22
		0.69215	LOC286161						0.33434	PRR7	0.996	LOC100288226
		0.69284	NSUN5						0.33468	ZNF765	0.997	GP6
		0.69354	MON1A						0.33501	LOC100130642	0.998	MRPL30
		0.69423	PDE12						0.33535	KCTD9	0.999	BCL6
		0.69493	TMEM9						0.33568	CCDC111		
		0.69562	CAMK2N1						0.33602	MCM3		
		0.69632	PSMD6						0.33635	FAM60A		
		0.69701	KBTBD12						0.33669	SFMBT1		
		0.69771	EXOG						0.33703	SFRS13A		
		0.69840	TFB2M						0.33736	MICA		
		0.69910	B4GALT3						0.33770	CHMP4C		
		0.69979	MPV17						0.33803	C4orf37		
		0.70049	CCDC75						0.33837	OR5W2		
		0.70118	C6orf89						0.33870	ERAS		
		0.70188	C8orf40						0.33904	MCF2L		
		0.70257	SATB2						0.33938	FLJ25996		
		0.70327	NEK10						0.33971	SPINK9		
		0.70396	SOBP						0.34005	LOC100130557		
		0.70466	ATP6V1G2						0.34038	PHB		
		0.70535	ZNF736						0.34072	HMGB1		
		0.70605	SNX4						0.34105	LOC440742		
		0.70674	CMPK1						0.34139	C15orf5		
		0.70744	SLC35E2						0.34173	C1orf98		
		0.70813	CCDC28A						0.34206	ANKRD30A		
		0.70883	TSSC1						0.34240	GLYATL1		

		0.70952	CASP9							0.34273	SNORD1C
		0.71022	LOC285484							0.34307	C17orf42
		0.71091	ACVR2A							0.34340	ZMYND15
		0.71161	DRAM2							0.34374	ZNF578
		0.71230	TBP							0.34408	DDX59
		0.71300	NMD3							0.34441	LOC100287737
		0.71369	RRP15							0.34475	ADAMTS5
		0.71438	SMYD5							0.34508	N4BP2
		0.71508	PCDHB11							0.34542	LOC440570
		0.71577	C1orf124							0.34575	RG9MTD2
		0.71647	SBDS							0.34609	ELAC1
		0.71716	PSMB1							0.34642	CCDC126
		0.71786	SQLE							0.34676	C8orf73
		0.71855	PSMA5							0.34710	COL4A1
		0.71925	RARS							0.34743	EFCAB5
		0.71994	CREG1							0.34777	ELTD1
		0.72064	CCNG2							0.34810	CDCA2
		0.72133	LOC441086							0.34844	OFD1
		0.72203	TFRC							0.34877	GTF2A1L
		0.72272	CHCHD6							0.34911	CLPS
		0.72342	MTMR12							0.34945	C13orf33
		0.72411	CALU							0.34978	C2CD4D
		0.72481	ATG7							0.35012	ZSCAN21
		0.72550	L3MBTL3							0.35045	C20orf56
		0.72620	ZNF7							0.35079	SEPSECS
		0.72689	HRASLS							0.35112	LOC284900
		0.72759	TRMT12							0.35146	LOC100271722
		0.72828	PACRGL							0.35180	ALX1
		0.72898	MAPK13							0.35213	SLC26A3
		0.72967	IP6K1							0.35247	ACER2
		0.73037	MED7							0.35280	SUGT1L1
		0.73106	ST6GAL1							0.35314	AASDH
		0.73176	DNAJB14							0.35347	CHRNA7
		0.73245	IMPDH2							0.35381	POPDC2
		0.73315	ASAP2							0.35415	RUFY1
		0.73384	KLHL3							0.35448	SNORD104
		0.73454	PART1							0.35482	C10orf67
		0.73523	NR2C2							0.35515	SNHG5
		0.73593	NUS1							0.35549	BCL2L11
		0.73662	MANEA							0.35582	PDZD3
		0.73732	KCNK1							0.35616	CXorf58
		0.73801	CGREF1							0.35650	RAG1AP1

		0.73871	ZFYVE16						0.35683	CLGN
		0.73940	SS18L2						0.35717	MTHFD1L
		0.74010	ZNF436						0.35750	ARHGAP11A
		0.74079	DOCK3						0.35784	ATAD2B
		0.74149	MED10						0.35817	KBTBD10
		0.74218	FLJ43663						0.35851	C9orf142
		0.74288	AMMECR1L						0.35885	GKAP1
		0.74357	LOC401220						0.35918	C1orf69
		0.74427	AGPAT1						0.35952	CYP51A1
		0.74496	LOC100288911						0.35985	ARHGEF33
		0.74566	ZNF717						0.36019	ST6GAL1
		0.74635	LOC348751						0.36052	C1orf97
		0.74705	BCS1L						0.36086	MAFK
		0.74774	PI4KB						0.36120	RNF133
		0.74844	LOC349114						0.36153	ACBD6
		0.74913	MTIF2						0.36187	C6orf182
		0.74983	GMDS						0.36220	LOC348840
		0.75052	SFRS12IP1						0.36254	TMEM179B
		0.75122	RAB33B						0.36287	C2orf77
		0.75191	FAM134B						0.36321	MGST2
		0.75261	C3orf59						0.36354	QSOX2
		0.75330	TTL						0.36388	HNRNPF
		0.75400	RASGEF1B						0.36422	RCN1
		0.75469	BRE						0.36455	C15orf56
		0.75539	APEH						0.36489	C4orf46
		0.75608	PSMB4						0.36522	GABRA6
		0.75678	BUD31						0.36556	LOC100290696
		0.75747	FILIP1L						0.36589	PAPOLB
		0.75817	C2orf69						0.36623	ZNF20
		0.75886	MSL2						0.36657	ZNF19
		0.75956	RDH14						0.36690	PMS1
		0.76025	PUS7						0.36724	FAM114A2
		0.76095	MTA3						0.36757	ENPP1
		0.76164	PPP1R15B						0.36791	CRY1
		0.76233	LOC401321						0.36824	GALNT12
		0.76303	GPAA1						0.36858	LILRB1
		0.76372	STK19						0.36892	POLA1
		0.76442	ZNF518B						0.36925	C2orf7
		0.76511	FLJ35946						0.36959	S100A12
		0.76581	LOC100132111						0.36992	CCDC17
		0.76650	GPATCH4						0.37026	THAP5
		0.76720	SKAP2						0.37059	SULT1B1

		0.76789	SIDT1						0.37093	ZNF681
		0.76859	GTF2H5						0.37127	TMEM182
		0.76928	PRKAR2A						0.37160	GALNT7
		0.76998	SF3A3						0.37194	TRDMT1
		0.77067	RPUSD3						0.37227	DARS2
		0.77137	ATP11B						0.37261	FERMT1
		0.77206	LRRC41						0.37294	FOXL1
		0.77276	ACVR2B						0.37328	KCNMB3
		0.77345	RAD17						0.37362	CENPQ
		0.77415	NUDT16						0.37395	TRPV3
		0.77484	CCDC85C						0.37429	ZIC4
		0.77554	LOC729680						0.37462	DSCR8
		0.77623	GFOD1						0.37496	VWDE
		0.77693	NAF1						0.37529	TUBE1
		0.77762	POLD2						0.37563	SNORD38B
		0.77832	ETV1						0.37597	TMEM67
		0.77901	TLK1						0.37630	ZNF682
		0.77971	PCDHB4						0.37664	C17orf68
		0.78040	MCFD2						0.37697	ZXDA
		0.78110	SEC62						0.37731	PTMA
		0.78179	BRIX1						0.37764	SLC24A1
		0.78249	TBCCD1						0.37798	C6orf112
		0.78318	GPN1						0.37831	TBX19
		0.78388	RFTN1						0.37865	STAG1
		0.78457	RAB5A						0.37899	SNORA52
		0.78527	AFAP1						0.37932	C13orf27
		0.78596	KCNT2						0.37966	CSRP3
		0.78666	ZNF425						0.37999	ARSF
		0.78735	FEZ2						0.38033	E2F5
		0.78805	FUT9						0.38066	NEXN
		0.78874	BRPF3						0.38100	PUS10
		0.78944	CRYZ						0.38134	KCNMB2
		0.79013	ITGA9						0.38167	LOC100289277
		0.79083	ALG14						0.38201	ZNF525
		0.79152	INO80D						0.38234	PPPDE1
		0.79222	KLHL23						0.38268	CEP76
		0.79291	AP4B1						0.38301	PRDM5
		0.79361	C1orf212						0.38335	MMP1
		0.79430	DPY19L1						0.38369	ZNF187
		0.79500	C1orf55						0.38402	AMBN
		0.79569	RRP15						0.38436	TAB2
		0.79639	TSPAN17						0.38469	ZNF506

0.79708	LOC100130798	0.38503	SERPINB13
0.79778	CSNK1A1	0.38536	LSM14B
0.79847	SMAD1	0.38570	LOC151146
0.79917	SEC24D	0.38604	DUSP28
0.79986	C20orf29	0.38637	FAM105A
0.80056	RAD1	0.38671	TEX264
0.80125	FUCA1	0.38704	LOC100133219
0.80195	PPM1K	0.38738	HAUS3
0.80264	TPST1	0.38771	GRB7
0.80334	HIST3H2BB	0.38805	DNAH14
0.80403	CCT3	0.38839	RAB12
0.80473	NFXL1	0.38872	AP4E1
0.80542	ATG12	0.38906	HMGB2
0.80612	CHSY3	0.38939	RPS24
0.80681	METTL5	0.38973	C3orf52
0.80751	ATP6V0E2	0.39006	TTC25
0.80820	LOC100289246	0.39040	CCDC138
0.80890	LOC93622	0.39074	LOC441795
0.80959	FOXP2	0.39107	IQCF5
0.81028	DNAJC21	0.39141	SPEF1
0.81098	MMACHC	0.39174	C3orf20
0.81167	ATP5F1	0.39208	HOPX
0.81237	SENP5	0.39241	SEC61G
0.81306	ODC1	0.39275	AZGP1
0.81376	ADAP1	0.39308	LOC643201
0.81445	SYT14	0.39342	CCNO
0.81515	LOC286063	0.39376	CGRRF1
0.81584	RNF2	0.39409	TRIP13
0.81654	ISPD	0.39443	RARG
0.81723	PDGFA	0.39476	CD226
0.81793	SLC25A4	0.39510	LRRIQ4
0.81862	INO80D	0.39543	DYNLT1
0.81932	ACP1	0.39577	LOC100287921
0.82001	TMEM178	0.39611	ASB18
0.82071	RHO	0.39644	FAM175A
0.82140	NDST3	0.39678	LOC154860
0.82210	ZCCHC9	0.39711	SPAG1
0.82279	C6orf125	0.39745	UBXN8
0.82349	PPP1R10	0.39778	LOC100287440
0.82418	TM2D1	0.39812	POP1
0.82488	OR2L1P	0.39846	ALG5
0.82557	UTP15	0.39879	GLYATL3

		0.82627	KIAA1109						0.39913	TNIP1
		0.82696	ACOT4						0.39946	MATN3
		0.82766	CEP70						0.39980	ZNF345
		0.82835	RAB6C						0.40013	SLC9A11
		0.82905	KIAA0895						0.40047	SYCP2L
		0.82974	RPP21						0.40081	IRS1
		0.83044	GATC						0.40114	MRPS28
		0.83113	ACTB						0.40148	GUK1
		0.83183	NAGK						0.40181	HNRNPL
		0.83252	ANAPC10						0.40215	LOC100130430
		0.83322	C2orf43						0.40248	IMMP2L
		0.83391	OR2L3						0.40282	EPSTI1
		0.83461	NCL						0.40316	RFC4
		0.83530	DUSP22						0.40349	FLJ45482
		0.83600	INTS12						0.40383	ITGA4
		0.83669	RASL11B						0.40416	ZNF816A
		0.83739	BCL11B						0.40450	SPATA5
		0.83808	PARL						0.40483	PPCDC
		0.83878	HSPA9						0.40517	ZNF534
		0.83947	CANX						0.40551	ZNF814
		0.84017	THUMPD2						0.40584	FAM91A1
		0.84086	KCTD6						0.40618	ZNF167
		0.84156	ZMAT2						0.40651	ZNF606
		0.84225	TRMT11						0.40685	HIST1H2AE
		0.84295	DDI2						0.40718	ANGPT1
		0.84364	CAMK1						0.40752	TRIM69
		0.84434	CDK11A						0.40785	FEM1A
		0.84503	LOC392275						0.40819	PPP4R1L
		0.84573	C5orf36						0.40853	ITIH4
		0.84642	CIDEA						0.40886	FP6628
		0.84712	GUCY1A3						0.40920	PRMT6
		0.84781	NICN1						0.40953	DTWD1
		0.84851	BSDC1						0.40987	ZNF260
		0.84920	FAM19A1						0.41020	C11orf67
		0.84990	MBLAC2						0.41054	GYPA
		0.85059	LOC729986						0.41088	LRRC70
		0.85129	LPHN3						0.41121	ZNF814
		0.85198	FRG1						0.41155	PHACTR4
		0.85268	GRINA						0.41188	LOC100131673
		0.85337	SFRS2B						0.41222	C9orf131
		0.85407	HIST1H2BD						0.41255	OR51B6
		0.85476	KIAA1191						0.41289	ZNF389

		0.85546	MUL1					0.41323	ARL5C
		0.85615	ZNF777					0.41356	YBX1
		0.85685	GPR52					0.41390	L3MBTL2
		0.85754	LYSMD1					0.41423	MRPL22
		0.85823	STK39					0.41457	SERPINI2
		0.85893	EIF4G1					0.41490	PDPK1
		0.85962	SLC7A14					0.41524	ZNF287
		0.86032	C1orf156					0.41558	RPL13
		0.86101	TTC30A					0.41591	LOC100131551
		0.86171	NPY5R					0.41625	CCDC67
		0.86240	INO80D					0.41658	SLC35D1
		0.86310	AJAP1					0.41692	FAM76B
		0.86379	RPL37A					0.41725	SLC27A6
		0.86449	C2orf72					0.41759	C10orf28
		0.86518	MANEAL					0.41793	ACYP1
		0.86588	LOC100288947					0.41826	CYP19A1
		0.86657	C6orf62					0.41860	VDAC2
		0.86727	C3orf31					0.41893	BNIP1
		0.86796	C3orf10					0.41927	SEC13
		0.86866	PTCD2					0.41960	FAM105B
		0.86935	LOC654433					0.41994	HINT1
		0.87005	PRO2012					0.42028	WIBG
		0.87074	GGPS1					0.42061	PKIB
		0.87144	RPL14					0.42095	ADAT2
		0.87213	PPAT					0.42128	PGGT1B
		0.87283	CLDN16					0.42162	ZNF702P
		0.87352	APPL1					0.42195	FAM188B2
		0.87422	PNRC1					0.42229	IL28A
		0.87491	ATXN7L1					0.42263	PTTG1
		0.87561	CSNK1A1					0.42296	MAGEC3
		0.87630	CCT6A					0.42330	ZNF701
		0.87700	FLJ41130					0.42363	RBP5
		0.87769	ZNF346					0.42397	LOC283658
		0.87839	CHCHD3					0.42430	OSTBETA
		0.87908	DDX5					0.42464	LOC100291644
		0.87978	PCDHB9					0.42497	CPA2
		0.88047	LOC100287359					0.42531	IQUB
		0.88117	OR2AK2					0.42565	C20orf123
		0.88186	NAMPT					0.42598	CNTD1
		0.88256	LOC100130560					0.42632	CALML4
		0.88325	NAV1					0.42665	GIN1
		0.88395	FLJ37060					0.42699	LOC729806

0.88464	HAUS3	0.42732	SLITRK6
0.88534	PREB	0.42766	ZNF555
0.88603	LOC439914	0.42800	LYG1
0.88673	SRCRB4D	0.42833	FAM184B
0.88742	TMEM232	0.42867	HIST2H2BF
0.88812	LOC100289552	0.42900	MIR543
0.88881	ZNF687	0.42934	EDC3
0.88951	ERCC8	0.42967	MIR17
0.89020	WTAP	0.43001	C2orf60
0.89090	LOC644961	0.43035	SH3BP5L
0.89159	DNAJC8	0.43068	TOR3A
0.89229	SEC62	0.43102	LOC100134259
0.89298	OR2L8	0.43135	C5orf38
0.89368	RNASEL	0.43169	HDX
0.89437	PPCS	0.43202	LOC100289292
0.89507	PIP5K1A	0.43236	TFB1M
0.89576	SSR1	0.43270	C1orf53
0.89646	FOXK1	0.43303	WDR67
0.89715	NDUFAF3	0.43337	WDR53
0.89785	HIST1H4I	0.43370	RILPL1
0.89854	RBM15	0.43404	TNAP
0.89924	C2orf47	0.43437	HPD
0.89993	HTR6	0.43471	MYLK4
0.90063	FN3K	0.43505	POLG2
0.90132	FOXK1	0.43538	NDUFB7
0.90202	C5orf15	0.43572	LOC100302650
0.90271	FIGNL2	0.43605	ATP8B1
0.90341	LOC157503	0.43639	TMEM61
0.90410	FKBP1B	0.43672	DCDC2
0.90479	LOC339524	0.43706	LMO2
0.90549	PLEKHO1	0.43740	PRDXDD1P
0.90618	LOC100288820	0.43773	LMOD3
0.90688	FLJ34208	0.43807	TPX2
0.90757	LNPEP	0.43840	RARS2
0.90827	LOC100289561	0.43874	GTPBP5
0.90896	MAN2B2	0.43907	ZNF696
0.90966	HTR4	0.43941	C6orf1
0.91035	CNIH3	0.43974	THBS4
0.91105	HIST1H2BG	0.44008	KLRA1
0.91174	OTUD4	0.44042	MRPL30
0.91244	RNASEH1	0.44075	BTN2A1
0.91313	LOC100130761	0.44109	MTMR8

0.91383	HIBADH	0.44142	RRH
0.91452	TMEM39B	0.44176	CCDC150
0.91522	RAB24	0.44209	LOC284825
0.91591	CCDC23	0.44243	TIFA
0.91661	IFFO2	0.44277	IRAK1
0.91730	UTP3	0.44310	CHCHD5
0.91800	IP6K2	0.44344	XPC
0.91869	SKI	0.44377	UMPS
0.91939	PPIH	0.44411	ESPN
0.92008	ARL4A	0.44444	B3GALNT2
0.92078	LOC100128822	0.44478	KCNK13
0.92147	FIGN	0.44512	VN1R5
0.92217	PCDH1	0.44545	SNORD7
0.92286	NSMCE2	0.44579	FAIM
0.92356	RHOQ	0.44612	KTN1
0.92425	NUCKS1	0.44646	OSTN
0.92495	KIAA1919	0.44679	FAM190A
0.92564	KIF17	0.44713	CRISPLD1
0.92634	LOC100129852	0.44747	LOC100132764
0.92703	GAK	0.44780	MACROD2
0.92773	STK16	0.44814	ROPN1B
0.92842	PTPRU	0.44847	DNAH14
0.92912	LOC730961	0.44881	ABCA5
0.92981	MKI67IP	0.44914	BLID
0.93051	ATXN7L1	0.44948	CYorf15A
0.93120	WDR55	0.44982	DIMT1L
0.93190	H2AFY	0.45015	ESF1
0.93259	SRRM1	0.45049	BPI
0.93329	AIDA	0.45082	PYCR2
0.93398	CCDC93	0.45116	TTC32
0.93468	GPR45	0.45149	MDFIC
0.93537	HIST2H2BF	0.45183	IMMP1L
0.93607	CMBL	0.45217	TRPC4
0.93676	TAF5L	0.45250	ITGB3BP
0.93746	FBXO6	0.45284	HYAL2
0.93815	CNTN3	0.45317	LOC153546
0.93885	VHL	0.45351	PERP
0.93954	LOC349196	0.45384	PROS1
0.94024	TOR1AIP1	0.45418	ZNF619
0.94093	CNPY3	0.45451	LOC729570
0.94163	XPO7	0.45485	FLJ45964
0.94232	FAM91A1	0.45519	ATP8A2

		0.94302	LOC100132345						0.45552	AP1S2
		0.94371	TMEM170B						0.45586	CEP152
		0.94441	KIAA1211						0.45619	GTF3A
		0.94510	FLJ40330						0.45653	ALDH8A1
		0.94580	LOC100288548						0.45686	ANKRD49
		0.94649	C1orf51						0.45720	PECR
		0.94719	WNT7A						0.45754	FAT4
		0.94788	CYP26B1						0.45787	SNCAIP
		0.94858	SETDB2						0.45821	ZSCAN12
		0.94927	HERC2P7						0.45854	LPA
		0.94997	OSCAR						0.45888	FASTKD3
		0.95066	PPP1R8						0.45921	F5
		0.95136	TRA2B						0.45955	CCDC122
		0.95205	C8orf85						0.45989	TAS2R13
		0.95274	DGKQ						0.46022	SERPINB2
		0.95344	KIAA1549						0.46056	PDCD2L
		0.95413	ABCB6						0.46089	ATAD2B
		0.95483	LOC441617						0.46123	MPP3
		0.95552	DNAH6						0.46156	RXFP2
		0.95622	SH3GL1P1						0.46190	PCDHA1
		0.95691	IGSF8						0.46224	ALKBH2
		0.95761	PDE4DIP						0.46257	CCPG1
		0.95830	ZNF506						0.46291	LOC100132159
		0.95900	GVIN1						0.46324	EIF4B
		0.95969	OR2L2						0.46358	C14orf145
		0.96039	AFAP1						0.46391	BIN2
		0.96108	LOC100129434						0.46425	NTS
		0.96178	ADCY5						0.46459	FZD6
		0.96247	LOC729218						0.46492	TMEM99
		0.96317	FAM5C						0.46526	LOC730651
		0.96386	TADA3						0.46559	CCT8P1
		0.96456	C2orf79						0.46593	CTBP2
		0.96525	DOCK4						0.46626	FAM20A
		0.96595	GAPDHP32						0.46660	VGLL3
		0.96664	JAKMIP1						0.46694	NFYB
		0.96734	C12orf64						0.46727	ZNF468
		0.96803	TAS2R3						0.46761	WDHD1
		0.96873	C1orf74						0.46794	LEPRE1
		0.96942	MCM9						0.46828	SVEP1
		0.97012	FAM188B2						0.46861	GIN1
		0.97081	ILF2						0.46895	MME
		0.97151	C9orf82						0.46928	LOC100292748

		0.97220	ROCK1P1						0.46962	STL
		0.97290	SSR1						0.46996	LXN
		0.97359	ZNF468						0.47029	MTRF1L
		0.97429	DPH2						0.47063	FBXL18
		0.97498	LEFTY1						0.47096	LOC100131117
		0.97568	RNPC3						0.47130	RNASEH2C
		0.97637	ETV3						0.47163	SLC19A3
		0.97707	PIGP						0.47197	CXorf26
		0.97776	MYOM2						0.47231	ST8SIA6
		0.97846	RHOXF1						0.47264	C2orf66
		0.97915	LOC257396						0.47298	CCT6P3
		0.97985	MRPS33						0.47331	EIF1AY
		0.98054	DNAH6						0.47365	LOC100288183
		0.98124	LOC100131342						0.47398	C2orf63
		0.98193	TIGD6						0.47432	ZNF761
		0.98263	PTPN18						0.47466	PPP1R2
		0.98332	LOC644063						0.47499	IMPDH1
		0.98402	PLA2G2C						0.47533	HMGN3
		0.98471	DENND3						0.47566	LOC643723
		0.98541	ZKSCAN3						0.47600	UGGT1
		0.98610	HECTD3						0.47633	BCL10
		0.98680	LOC284408						0.47667	NPR3
		0.98749	SART1						0.47701	ACPP
		0.98819	DYRK2						0.47734	TRNAU1AP
		0.98888	MANF						0.47768	NCRNA00171
		0.98958	PMS2L1						0.47801	ARMC6
		0.99027	FICD						0.47835	KLKB1
		0.99097	LOC729680						0.47868	C3orf31
		0.99166	C17orf76						0.47902	CKLF
		0.99236	SPR						0.47936	MYH3
		0.99305	LOC100131342						0.47969	ZNF564
		0.99375	RUSC2						0.48003	HULC
		0.99444	C16orf82						0.48036	C5orf28
		0.99514	ARL17A						0.48070	LOC642533
		0.99583	CLDN9						0.48103	LMAN2L
		0.99653	C11orf10						0.48137	FAM122C
		0.99722	GAPDHL7						0.48171	HOXB3
		0.99792	C12orf64						0.48204	ZNF736
		0.99861	CYB5RL						0.48238	RIOK1
		0.99931	ZNF812						0.48271	MTFMT
									0.48305	ZNF781
									0.48338	TXNRD3

								0.48372	KIN
								0.48406	NKAPL
								0.48439	WDR70
								0.48473	RRN3P1
								0.48506	AGBL1
								0.48540	CFDP1
								0.48573	C10orf115
								0.48607	CCDC39
								0.48640	KIAA0922
								0.48674	NUSAP1
								0.48708	ZNF831
								0.48741	GPRIN3
								0.48775	CCNA2
								0.48808	SPATA5
								0.48842	FANCI
								0.48875	TMED6
								0.48909	ZCCHC10
								0.48943	C5orf25
								0.48976	CTDP1
								0.49010	LOC100132963
								0.49043	NAP1L4
								0.49077	LOC100289347
								0.49110	CCNB2
								0.49144	CD300LB
								0.49178	DRD3
								0.49211	ZNF431
								0.49245	GNL2
								0.49278	MGC15885
								0.49312	ZNF761
								0.49345	ZNF486
								0.49379	DEFB1
								0.49413	CYP20A1
								0.49446	LRP2BP
								0.49480	FHDC1
								0.49513	ZNF766
								0.49547	TEX12
								0.49580	TBC1D10A
								0.49614	MFSD9
								0.49648	PI15
								0.49681	C8orf84
								0.49715	FAM96A
								0.49748	HSPD1

								0.49782	FAM175A
								0.49815	RPL9
								0.49849	C3orf35
								0.49883	XRCC3
								0.49916	C3orf15
								0.49950	HELQ
								0.49983	ZNF554
								0.50017	DOCK11
								0.50050	IL1RAPL2
								0.50084	CYB5A
								0.50117	CLDN1
								0.50151	SVEP1
								0.50185	FAM82A1
								0.50218	NUDT22
								0.50252	ADAMTS5
								0.50285	FLJ27352
								0.50319	ABCA11P
								0.50352	NPPC
								0.50386	PDAP1
								0.50420	EBF4
								0.50453	C17orf74
								0.50487	FAM21A
								0.50520	LOC646241
								0.50554	C12orf64
								0.50587	ZNF616
								0.50621	C1GALT1C1
								0.50655	ASB5
								0.50688	KIAA0408
								0.50722	HAVCR1
								0.50755	ZNF45
								0.50789	MAB21L1
								0.50822	BRWD3
								0.50856	TIPIN
								0.50890	C6orf163
								0.50923	TMEM186
								0.50957	ZNF264
								0.50990	FAM169A
								0.51024	LOC100292236
								0.51057	RPL31
								0.51091	VPS53
								0.51125	C9orf93
								0.51158	LOC283711

								0.51192	RP11-410N8.4
								0.51225	PSMD9
								0.51259	IL20
								0.51292	MOP-1
								0.51326	ZIK1
								0.51360	LOC731282
								0.51393	XIAP
								0.51427	LOC440742
								0.51460	SLC15A5
								0.51494	RIPK1
								0.51527	TGS1
								0.51561	ZNF320
								0.51594	C11orf54
								0.51628	C9orf130
								0.51662	ARHGEF33
								0.51695	SCARNA13
								0.51729	CEP57
								0.51762	AGPAT6
								0.51796	OSGEPL1
								0.51829	LOC400027
								0.51863	ART3
								0.51897	SPAG7
								0.51930	ADAM19
								0.51964	CRCP
								0.51997	C3orf51
								0.52031	SMARCE1
								0.52064	FAM154B
								0.52098	OR6C3
								0.52132	C14orf145
								0.52165	CYP4F3
								0.52199	ZNF582
								0.52232	ATP6AP1L
								0.52266	SLC39A8
								0.52299	PTGFR
								0.52333	ZWILCH
								0.52367	POT1
								0.52400	TMEM195
								0.52434	DYX1C1
								0.52467	HSD17B7
								0.52501	ZNF616
								0.52534	TMEM187
								0.52568	MANBA

0.52602	LCA5
0.52635	LOC283089
0.52669	EHF
0.52702	DOCK4
0.52736	PRO2852
0.52769	LIPT1
0.52803	CYB561D1
0.52837	ZSWIM5
0.52870	RPS6KA6
0.52904	MRPL22
0.52937	NUDT19
0.52971	NFU1
0.53004	SKA3
0.53038	C19orf50
0.53072	DSCC1
0.53105	ANKHD1
0.53139	SYDE2
0.53172	F2RL2
0.53206	LOC645355
0.53239	PCDHA6
0.53273	RPL32
0.53306	ACVR2B
0.53340	ALS2CR12
0.53374	SLC25A30
0.53407	WDR35
0.53441	TAS2R46
0.53474	LOC100291668
0.53508	ZFP37
0.53541	CXCL12
0.53575	ZNF660
0.53609	LOC729041
0.53642	ELMOD2
0.53676	FBXO4
0.53709	EDN1
0.53743	DDX49
0.53776	ZNF197
0.53810	DYRK3
0.53844	ADAM21
0.53877	FAM13AOS
0.53911	C1orf161
0.53944	C5orf54
0.53978	NDST4

								0.54011	TRIM4
								0.54045	LOC100130000
								0.54079	AMY2B
								0.54112	LOC100129858
								0.54146	OTX2OS1
								0.54179	PXDNL
								0.54213	TTC12
								0.54246	LOC100288260
								0.54280	SFRS12IP1
								0.54314	C10orf4
								0.54347	SCAMP3
								0.54381	C20orf199
								0.54414	SNRNP48
								0.54448	LYPD6
								0.54481	ZNF354B
								0.54515	AKD1
								0.54549	SRP14
								0.54582	NUDCD1
								0.54616	CCDC152
								0.54649	PABPC1L
								0.54683	C6orf202
								0.54716	C11orf70
								0.54750	REL
								0.54783	LOC284998
								0.54817	SETDB2
								0.54851	GK
								0.54884	CCT8P1
								0.54918	ZNF14
								0.54951	CERKL
								0.54985	CMAH
								0.55018	ADPGK
								0.55052	GEN1
								0.55086	CCDC66
								0.55119	ANGPT2
								0.55153	IMPG2
								0.55186	AKD1
								0.55220	SLC27A2
								0.55253	ZNF557
								0.55287	TMX1
								0.55321	SRF
								0.55354	CYP2R1
								0.55388	LYRM7

0.55421	PDCD2
0.55455	LOC642103
0.55488	NDUFB3
0.55522	KIR3DX1
0.55556	CYP2R1
0.55589	UQCRQ
0.55623	LOC644285
0.55656	PIBF1
0.55690	EHMT1
0.55723	LOC149351
0.55757	PPAPDC2
0.55791	CWF19L2
0.55824	ZNF737
0.55858	MDM1
0.55891	PDLIM1
0.55925	ZIC5
0.55958	TIMM44
0.55992	LOC730102
0.56026	ZFAND6
0.56059	G3BP1
0.56093	INTS2
0.56126	C3orf75
0.56160	NSUN2
0.56193	EPC1
0.56227	TAS2R14
0.56260	GCH1
0.56294	MREG
0.56328	TMEM20
0.56361	GUSBL2
0.56395	INO80B
0.56428	LOC643085
0.56462	SLC22A25
0.56495	C9orf130
0.56529	DNAJB6
0.56563	N6AMT1
0.56596	MFAP1
0.56630	SIRT1
0.56663	ZNF595
0.56697	LOC100132832
0.56730	DNPEP
0.56764	CTAGE5
0.56798	CHI3L2

								0.56831	RANBP17
								0.56865	FAM103A1
								0.56898	EIF3B
								0.56932	OSTN
								0.56965	STC1
								0.56999	TIMM9
								0.57033	VANGL1
								0.57066	ADH6
								0.57100	MGC16703
								0.57133	ZNF737
								0.57167	C10orf32
								0.57200	ZNF630
								0.57234	RPS15A
								0.57268	RTN4IP1
								0.57301	BACH1
								0.57335	SVEP1
								0.57368	SVEP1
								0.57402	MTERFD1
								0.57435	ZNF407
								0.57469	FEN1
								0.57503	LSM8
								0.57536	NDUFAF2
								0.57570	DNM1P35
								0.57603	C7orf28B
								0.57637	C14orf126
								0.57670	LOC100144602
								0.57704	MRPS17
								0.57737	RGS17
								0.57771	LOC100129387
								0.57805	ZNF253
								0.57838	GK5
								0.57872	ZNF33B
								0.57905	RPL19
								0.57939	CNOT10
								0.57972	HP1BP3
								0.58006	FUS
								0.58040	SP5
								0.58073	ZNF836
								0.58107	CCDC104
								0.58140	HSPA14
								0.58174	C2orf15
								0.58207	BPNT1

								0.58241	C1orf114
								0.58275	OR5F1
								0.58308	OTX2
								0.58342	G0S2
								0.58375	PDIA3P
								0.58409	LEF1
								0.58442	TMEM5
								0.58476	PIGC
								0.58510	PABPC1
								0.58543	ZNF284
								0.58577	RPL36AL
								0.58610	LOC100130345
								0.58644	FLJ31485
								0.58677	NEB
								0.58711	FAM55B
								0.58745	PXDNL
								0.58778	ZNF642
								0.58812	CRYBB2P1
								0.58845	RBM17
								0.58879	LOC646851
								0.58912	RPP40
								0.58946	CEP192
								0.58980	MAGI1
								0.59013	TTC26
								0.59047	ALG10B
								0.59080	ZNF286A
								0.59114	PGM2
								0.59147	CCDC76
								0.59181	B3GNT3
								0.59215	ZBTB24
								0.59248	ARMCX5
								0.59282	H6PD
								0.59315	LOC645212
								0.59349	ZC3HAV1L
								0.59382	RARRES1
								0.59416	C11orf58
								0.59449	PFDN5
								0.59483	ARL16
								0.59517	ZNF835
								0.59550	TES
								0.59584	ZSCAN12P1
								0.59617	KCNQ1OT1

								0.59651	SVEP1
								0.59684	PHRF1
								0.59718	HEMK1
								0.59752	CEP78
								0.59785	FBXO8
								0.59819	ZNF81
								0.59852	ZNF35
								0.59886	TRIM5
								0.59919	RAC1
								0.59953	DUSP18
								0.59987	C8orf34
								0.60020	HEATR1
								0.60054	COQ7
								0.60087	ZNF323
								0.60121	LOC642340
								0.60154	ZNF684
								0.60188	CBWD2
								0.60222	SHISA6
								0.60255	ISYNA1
								0.60289	CMTM6
								0.60322	ZSCAN20
								0.60356	LOC283027
								0.60389	PRKCDBP
								0.60423	LOC389895
								0.60457	ACAD10
								0.60490	CCL23
								0.60524	ZNF485
								0.60557	SLC2A8
								0.60591	LOC100009676
								0.60624	PA2G4
								0.60658	C9orf43
								0.60692	LOC642384
								0.60725	RMI1
								0.60759	FBLL1
								0.60792	ADAMTS16
								0.60826	PLSCR3
								0.60859	LOC219347
								0.60893	OR5B21
								0.60926	PLCD4
								0.60960	C3orf55
								0.60994	WDR69
								0.61027	TAF3

								0.61061	TRPM1
								0.61094	MAP1LC3B
								0.61128	TRIM39
								0.61161	UMPS
								0.61195	MPDU1
								0.61229	ZBTB49
								0.61262	C6orf35
								0.61296	ITGAE
								0.61329	DMRT3
								0.61363	NVL
								0.61396	RPLP0P2
								0.61430	RAPH1
								0.61464	C14orf28
								0.61497	SAAL1
								0.61531	C2orf65
								0.61564	LOC100101938
								0.61598	SP3P
								0.61631	C3orf19
								0.61665	NCBP1
								0.61699	APITD1
								0.61732	FLJ33065
								0.61766	NF1
								0.61799	ARPC5
								0.61833	LOC100133106
								0.61866	UBTD2
								0.61900	MGMT
								0.61934	CWC25
								0.61967	DIDO1
								0.62001	TRAF2
								0.62034	NHLH2
								0.62068	CTSL1
								0.62101	XRCC2
								0.62135	ZCCHC8
								0.62169	TMUB2
								0.62202	SDHD
								0.62236	LOC150776
								0.62269	HNF1B
								0.62303	CREM
								0.62336	SLC25A26
								0.62370	MAP7D1
								0.62403	HELZ
								0.62437	ZNF41

								0.62471	LOC100216001
								0.62504	GIMAP1
								0.62538	HYLS1
								0.62571	HOMER2
								0.62605	ST7
								0.62638	LOC100130855
								0.62672	MTHFD2L
								0.62706	METTL2B
								0.62739	LOC644093
								0.62773	DKFZp686K1684
								0.62806	KCNIP4
								0.62840	C10orf67
								0.62873	NUDT13
								0.62907	MITD1
								0.62941	DOT1L
								0.62974	BAHD1
								0.63008	C9orf64
								0.63041	ENDOG
								0.63075	SNORA31
								0.63108	ZNF551
								0.63142	PIK3CG
								0.63176	ATF1
								0.63209	ATP10D
								0.63243	IFRD1
								0.63276	LOC100287644
								0.63310	MRPL13
								0.63343	LOC642891
								0.63377	LOC100272228
								0.63411	RNF34
								0.63444	LOC147804
								0.63478	GRB14
								0.63511	SSPO
								0.63545	SKIL
								0.63578	C1orf59
								0.63612	ZNF496
								0.63646	MOCS2
								0.63679	CA13
								0.63713	CYCS
								0.63746	C12orf64
								0.63780	ALKBH8
								0.63813	DDX50
								0.63847	MEP1A

0.63880	KIAA0020
0.63914	STEAP1
0.63948	ARHGAP18
0.63981	SNORD4A
0.64015	COQ2
0.64048	EIF2B5
0.64082	SCFD1
0.64115	DPT
0.64149	TTN
0.64183	ERCC8
0.64216	DEF8
0.64250	GTF2E1
0.64283	FAM150B
0.64317	SVEP1
0.64350	BMP6
0.64384	WDSUB1
0.64418	CHMP4B
0.64451	GORAB
0.64485	CAD
0.64518	EYS
0.64552	ZNF792
0.64585	DNAH14
0.64619	ZNF724P
0.64653	ZNF785
0.64686	SOCS6
0.64720	GAS2L3
0.64753	SFRS2B
0.64787	REXO2
0.64820	ZNF598
0.64854	TRIM52
0.64888	C7orf28A
0.64921	VPS24
0.64955	KCNQ1OT1
0.64988	THAP6
0.65022	MTMR14
0.65055	CDC6
0.65089	EXD1
0.65123	ZBTB26
0.65156	UNCX
0.65190	FRMPD3
0.65223	SART3
0.65257	RCAN1

																0.65290	PLEKHF1
																0.65324	HP1BP3
																0.65358	RMRP
																0.65391	CDKN3
																0.65425	METTL1
																0.65458	ZNF836
																0.65492	C10orf110
																0.65525	ATP6V1G3
																0.65559	RNF139
																0.65592	ZNF48
																0.65626	CEP164
																0.65660	LOC339674
																0.65693	AKR1C1
																0.65727	C10orf107
																0.65760	TRIM25
																0.65794	C1orf187
																0.65827	MTHFSD
																0.65861	TRAF5
																0.65895	IFT81
																0.65928	LUC7L3
																0.65962	ZNF552
																0.65995	HAPLN1
																0.66029	ADAMTS19
																0.66062	CRLF3
																0.66096	EXOSC7
																0.66130	LOC100287599
																0.66163	TMX1
																0.66197	LOC285014
																0.66230	DKFZP586I1420
																0.66264	NUTF2
																0.66297	ERN1
																0.66331	C7orf59
																0.66365	LOC729314
																0.66398	POU2F1
																0.66432	PRDM11
																0.66465	ZNF799
																0.66499	DHX15
																0.66532	OBFC1
																0.66566	NHLRC1
																0.66600	ZFHX2
																0.66633	SLFN11
																0.66667	ATAD2B

								0.66700	LOC100129436
								0.66734	C6orf64
								0.66767	RPS6KB2
								0.66801	POSTN
								0.66835	SF3B4
								0.66868	ZFP2
								0.66902	THSD4
								0.66935	DTX2
								0.66969	PTDSS2
								0.67002	ZNF593
								0.67036	C9orf68
								0.67069	PLA2G4A
								0.67103	DDX18
								0.67137	LDHA
								0.67170	CASC1
								0.67204	DHX33
								0.67237	SMPDL3A
								0.67271	IFT52
								0.67304	WDR61
								0.67338	CCDC158
								0.67372	GRWD1
								0.67405	ZNF28
								0.67439	C20orf12
								0.67472	TMEM200C
								0.67506	WWC2
								0.67539	CEPT1
								0.67573	RFESD
								0.67607	FMNL1
								0.67640	LDLRAD2
								0.67674	VSIG1
								0.67707	EDEM2
								0.67741	LOC100131507
								0.67774	AGMAT
								0.67808	MAGEB18
								0.67842	LOC100126784
								0.67875	MEMO1
								0.67909	PARP11
								0.67942	DDX60L
								0.67976	AMN1
								0.68009	CABC1
								0.68043	C4orf23
								0.68077	SNF8

								0.68110	THAP2
								0.68144	TMEM87B
								0.68177	LSM1
								0.68211	PQLC2
								0.68244	RIT1
								0.68278	ZNF567
								0.68312	ZNF547
								0.68345	AFMID
								0.68379	SLCO4C1
								0.68412	OVGP1
								0.68446	SNX7
								0.68479	DAB1
								0.68513	LETM1
								0.68546	EDA
								0.68580	CCDC106
								0.68614	DNAH14
								0.68647	MRE11A
								0.68681	MRPS9
								0.68714	ATF3
								0.68748	NRG4
								0.68781	KLHL26
								0.68815	OBFC1
								0.68849	E2F6
								0.68882	CTNNAL1
								0.68916	IL10RB
								0.68949	BOD1P
								0.68983	SP3
								0.69016	PLAG1
								0.69050	CDYL
								0.69084	SLC5A3
								0.69117	TFAM
								0.69151	ZNF259
								0.69184	HIST1H4H
								0.69218	ZNF711
								0.69251	IRAK1BP1
								0.69285	AKTIP
								0.69319	GPR135
								0.69352	CROT
								0.69386	ZNF334
								0.69419	HCFC2
								0.69453	C21orf130
								0.69486	HMGN2

0.69520	PNLDC1
0.69554	FLJ37060
0.69587	LOC100128842
0.69621	MTERFD3
0.69654	CASQ2
0.69688	MPHOSPH6
0.69721	NEURL1B
0.69755	C20orf108
0.69789	FLCN
0.69822	PARD6G
0.69856	MIPEP
0.69889	DCLRE1C
0.69923	DDHD1
0.69956	PNO1
0.69990	GBE1
0.70023	ZNF225
0.70057	GTPBP8
0.70091	METTL8
0.70124	FAM36A
0.70158	FLJ45340
0.70191	TMPRSS11B
0.70225	TLR6
0.70258	ZNF526
0.70292	SVEP1
0.70326	PTRH2
0.70359	LOC730005
0.70393	ZNF140
0.70426	KRBA2
0.70460	NBPF3
0.70493	MTMR14
0.70527	NUDT13
0.70561	DIRC3
0.70594	C11orf46
0.70628	SH3D20
0.70661	ORC6L
0.70695	POLR2D
0.70728	CRBN
0.70762	GNAI3
0.70796	FEZF1
0.70829	C12orf64
0.70863	CWC27
0.70896	DKFZP686I15217

																0.70930	NAPRT1
																0.70963	DUSP4
																0.70997	BRIP1
																0.71031	SLC9A2
																0.71064	CASP3
																0.71098	CIDEB
																0.71131	POLR3GL
																0.71165	OR1F1
																0.71198	EIF3K
																0.71232	ZNF91
																0.71266	KDSR
																0.71299	DKFZP564C196
																0.71333	LOC646870
																0.71366	LECT1
																0.71400	FLJ16734
																0.71433	MCM6
																0.71467	LOC100131929
																0.71501	PRDM1
																0.71534	C20orf72
																0.71568	DDX21
																0.71601	TCEANC
																0.71635	LOC100335030
																0.71668	C12orf29
																0.71702	ZNF681
																0.71735	GPR19
																0.71769	TMEM194A
																0.71803	FNDC1
																0.71836	LOC729350
																0.71870	PPIE
																0.71903	DUT
																0.71937	LCORL
																0.71970	LOC100293158
																0.72004	DDOST
																0.72038	RBMS2
																0.72071	ACAP2
																0.72105	DMWD
																0.72138	PPP1R14C
																0.72172	FLJ33534
																0.72205	SLC16A4
																0.72239	TRIP4
																0.72273	C1orf132
																0.72306	GCLM

0.72340	ZNF773
0.72373	PAWR
0.72407	TPM3
0.72440	HOXA3
0.72474	LOC285540
0.72508	CBR3
0.72541	RNASE2
0.72575	C10orf93
0.72608	LACE1
0.72642	ZNF234
0.72675	ZXDA
0.72709	ING5
0.72743	LOC729156
0.72776	ZNF227
0.72810	MINA
0.72843	GPR149
0.72877	GINS1
0.72910	MRPL3
0.72944	RAB33A
0.72978	ZNF550
0.73011	ADPRH
0.73045	EPHB3
0.73078	BRMS1L
0.73112	LOC100133284
0.73145	PPP1R3D
0.73179	C3orf34
0.73212	TRIM16
0.73246	LOC150568
0.73280	C19orf46
0.73313	MYO5C
0.73347	NCK1
0.73380	TSPAN2
0.73414	DTWD2
0.73447	TBC1D2B
0.73481	RNMTL1
0.73515	SLC46A3
0.73548	F11R
0.73582	SUGT1
0.73615	TNFRSF11B
0.73649	ZC3HC1
0.73682	UNC119B
0.73716	ROMO1

0.73750	MRP63
0.73783	ZNF441
0.73817	SLC25A16
0.73850	CCNB1
0.73884	MAGEE2
0.73917	RAD23B
0.73951	PHOSPHO2
0.73985	PUS3
0.74018	ST8SIA4
0.74052	LMBR1L
0.74085	BCL2L15
0.74119	GPLD1
0.74152	ATXN1L
0.74186	PDZK1
0.74220	HEG1
0.74253	C22orf40
0.74287	TDRD7
0.74320	TBC1D16
0.74354	MIG7
0.74387	RNF112
0.74421	FKBP9
0.74455	ANKLE2
0.74488	GMCL1
0.74522	C6orf114
0.74555	C14orf106
0.74589	PLAC8L1
0.74622	EML4
0.74656	ANKRD36
0.74689	PARD6G
0.74723	C5orf53
0.74757	LOC284373
0.74790	CPSF3L
0.74824	SUV39H2
0.74857	SNED1
0.74891	FOXC2
0.74924	EYA4
0.74958	HEY1
0.74992	PKP2
0.75025	KIFC3
0.75059	LOC283404
0.75092	QRSL1
0.75126	CFLP1

								0.75159	REXO4
								0.75193	ANKH
								0.75227	FREM1
								0.75260	RPS6KA6
								0.75294	KIAA0485
								0.75327	CHODL
								0.75361	RFC3
								0.75394	FAM158A
								0.75428	LOC388559
								0.75462	TM9SF1
								0.75495	C17orf48
								0.75529	LOC442421
								0.75562	ATAD2B
								0.75596	LRRN2
								0.75629	C12orf64
								0.75663	ZKSCAN2
								0.75697	OXSM
								0.75730	SLC27A3
								0.75764	RASA4
								0.75797	LAMC1
								0.75831	LOC647309
								0.75864	ZNF835
								0.75898	SH3BGR
								0.75932	LOC253039
								0.75965	SAMD5
								0.75999	LOC284581
								0.76032	JAG1
								0.76066	ZNF720
								0.76099	LOC401233
								0.76133	SETMAR
								0.76166	LOC729409
								0.76200	C9orf130
								0.76234	TSPAN1
								0.76267	CLTB
								0.76301	FAM71E1
								0.76334	PDE3B
								0.76368	C6orf72
								0.76401	SNAPC1
								0.76435	ATAD2B
								0.76469	LYVE1
								0.76502	ARL5A
								0.76536	NOV

								0.76569	NUP54
								0.76603	PIN4
								0.76636	TMEM126A
								0.76670	SPDYA
								0.76704	LOC401093
								0.76737	GCKR
								0.76771	ZNF503
								0.76804	C14orf33
								0.76838	LOC100129884
								0.76871	ADAM32
								0.76905	ACBD4
								0.76939	KCNRG
								0.76972	ZNF28
								0.77006	LOC344065
								0.77039	MMAA
								0.77073	RALGPS2
								0.77106	LOC100132787
								0.77140	PAPPA
								0.77174	E2F3
								0.77207	BTG2
								0.77241	FOXD4L2
								0.77274	DKFZp564H213
								0.77308	TMEM159
								0.77341	PCDHB16
								0.77375	GDPD1
								0.77409	CLIC5
								0.77442	DGCR14
								0.77476	ZNF193
								0.77509	HSPBAP1
								0.77543	NUDCD2
								0.77576	SFRS13B
								0.77610	CDKN2AIP
								0.77644	RNY5
								0.77677	ZNF616
								0.77711	LOC678655
								0.77744	DBT
								0.77778	ZNHIT3
								0.77811	SYF2
								0.77845	ESCO1
								0.77878	METT5D1
								0.77912	ATF7IP2
								0.77946	POLR3E

								0.77979	CHSY1
								0.78013	RPH3AL
								0.78046	SPTY2D1
								0.78080	SLC25A15
								0.78113	THBS3
								0.78147	PFN1
								0.78181	LAMB2L
								0.78214	ATP5I
								0.78248	NTF3
								0.78281	CCNJ
								0.78315	TGFBR1
								0.78348	RHEBL1
								0.78382	RAD18
								0.78416	ZNF654
								0.78449	GTF2A2
								0.78483	NME3
								0.78516	CACYBP
								0.78550	TGFB1I1
								0.78583	TAF7
								0.78617	DPYS
								0.78651	PROX1
								0.78684	SLC22A5
								0.78718	AGPHD1
								0.78751	LYPLA1
								0.78785	CUTA
								0.78818	MTERF
								0.78852	C1orf70
								0.78886	DPY19L1P1
								0.78919	ANAPC16
								0.78953	ZFP82
								0.78986	ARMC7
								0.79020	C3orf64
								0.79053	BMP3
								0.79087	PRDX4
								0.79121	PDSS1
								0.79154	CIR1
								0.79188	FLJ37060
								0.79221	CRYGS
								0.79255	ZNF799
								0.79288	LOC283588
								0.79322	ACVR2B
								0.79355	ADARB2

								0.79389	LRRC68
								0.79423	HPSE2
								0.79456	PM20D2
								0.79490	OLFML1
								0.79523	MRPS24
								0.79557	JDP2
								0.79590	RBM45
								0.79624	HIST1H3E
								0.79658	ZNF506
								0.79691	PRKCH
								0.79725	TMSB15B
								0.79758	ATP5G2
								0.79792	C7orf16
								0.79825	LOC100288968
								0.79859	X2849859
								0.79893	LOC652990
								0.79926	GP1BB
								0.79960	TECTA
								0.79993	PWWP2B
								0.80027	PR47
								0.80060	FAM91A1
								0.80094	GNA11
								0.80128	C12orf66
								0.80161	C4orf23
								0.80195	TMEM173
								0.80228	COL25A1
								0.80262	CBWD5
								0.80295	ADSL
								0.80329	LOC677759
								0.80363	NAT1
								0.80396	SERTAD2
								0.80430	GPATCH2
								0.80463	LOC100130463
								0.80497	ARHGEF33
								0.80530	UTP6
								0.80564	HNMT
								0.80598	CHRNA6
								0.80631	FLJ38717
								0.80665	XRCC2
								0.80698	INTS6
								0.80732	LOC100131311
								0.80765	REXO1L1

0.80799	RAB12
0.80832	ZNF23
0.80866	LOC442155
0.80900	CPO
0.80933	ZNF121
0.80967	KLHL13
0.81000	XPO4
0.81034	TIMM23
0.81067	TRIM54
0.81101	CBX3
0.81135	NCRNA00158
0.81168	GOLT1A
0.81202	TASP1
0.81235	ZNF773
0.81269	TDO2
0.81302	LOC399815
0.81336	CDKL4
0.81370	KLHDC8A
0.81403	RUVBL2
0.81437	KIAA0232
0.81470	COMMD6
0.81504	ZMYND19
0.81537	PPP1R12C
0.81571	RAC3
0.81605	FLJ30375
0.81638	PDIA3
0.81672	ZNF470
0.81705	CCDC79
0.81739	ZNF502
0.81772	ATAD2B
0.81806	RPS4Y1
0.81840	FKTN
0.81873	NDUFB1
0.81907	TUBD1
0.81940	TYW3
0.81974	LOC339822
0.82007	TMEM135
0.82041	ZNF18
0.82075	TSPAN9
0.82108	LOC285074
0.82142	FANCF
0.82175	PGAM1

0.82209	PABPC5
0.82242	LOC100131434
0.82276	CSNK2A1
0.82309	LOC284385
0.82343	C1orf109
0.82377	MRPS25
0.82410	MED26
0.82444	MTPN
0.82477	APOLD1
0.82511	YIPF4
0.82544	AKT2
0.82578	AG2
0.82612	ANKRD45
0.82645	C8orf31
0.82679	C21orf33
0.82712	TAF8
0.82746	ZNF571
0.82779	RUNX1T1
0.82813	EXPH5
0.82847	CTNNBL1
0.82880	MLF1
0.82914	LOC643327
0.82947	PTCHD2
0.82981	EXOSC4
0.83014	VTN
0.83048	C2orf52
0.83082	WDR47
0.83115	SHISA6
0.83149	COTL1
0.83182	LOC441204
0.83216	SKIL
0.83249	C15orf44
0.83283	TSNARE1
0.83317	C6orf191
0.83350	LOC100132099
0.83384	ZNF620
0.83417	SULT1A1
0.83451	MED31
0.83484	MPPE1
0.83518	KIAA2026
0.83552	HSP90AA1
0.83585	LOC100130169

								0.83619	ZNF10
								0.83652	LSM6
								0.83686	C1orf151
								0.83719	SDSL
								0.83753	SLC25A26
								0.83787	SLC7A3
								0.83820	IGF2R
								0.83854	ABCB10
								0.83887	TWISTNB
								0.83921	RSPO3
								0.83954	LOC100288163
								0.83988	STK11
								0.84021	C19orf12
								0.84055	PRKG2
								0.84089	RBM4B
								0.84122	PI15
								0.84156	ITGA8
								0.84189	RAB11FIP4
								0.84223	GNA14
								0.84256	TTF1
								0.84290	SOX4
								0.84324	NUCKS1
								0.84357	HIST1H4D
								0.84391	ZFP41
								0.84424	ZNF527
								0.84458	HDGFRP2
								0.84491	IQCJ
								0.84525	LINS1
								0.84559	LOC100288316
								0.84592	HNMT
								0.84626	ANKRD19
								0.84659	ZNF91
								0.84693	C1orf203
								0.84726	MIER2
								0.84760	CXorf23
								0.84794	SF3A2
								0.84827	POLR1D
								0.84861	MGC87042
								0.84894	TIGD1
								0.84928	MRPL32
								0.84961	BFSP1
								0.84995	DKK3

								0.85029	C8orf83
								0.85062	MEGF8
								0.85096	ZNF211
								0.85129	ZNF835
								0.85163	BCL2L1
								0.85196	C3orf33
								0.85230	RCE1
								0.85264	MEG8
								0.85297	WBP4
								0.85331	ZNF749
								0.85364	NOSIP
								0.85398	SNHG8
								0.85431	LOC100129113
								0.85465	LOC284219
								0.85498	ZNF417
								0.85532	SMAD6
								0.85566	ZNF74
								0.85599	CBLL1
								0.85633	DBT
								0.85666	LOC100130278
								0.85700	DNAJC2
								0.85733	ULK4
								0.85767	ZNF389
								0.85801	TCERG1L
								0.85834	MRTO4
								0.85868	DENND1B
								0.85901	LOC100130451
								0.85935	FAM105A
								0.85968	DYRK4
								0.86002	IKBKG
								0.86036	SIAH1
								0.86069	FKBP7
								0.86103	CCDC30
								0.86136	AKD1
								0.86170	LRRC4B
								0.86203	KCNE4
								0.86237	SMARCB1
								0.86271	KLF11
								0.86304	ERO1LB
								0.86338	LOC285141
								0.86371	KRT18
								0.86405	CHRNA5

								0.86438	C12orf64
								0.86472	RERGL
								0.86506	INPP5B
								0.86539	TRIM66
								0.86573	LOC729810
								0.86606	LOC441046
								0.86640	DMAP1
								0.86673	MIDN
								0.86707	C7orf54
								0.86741	ProSAPiP1
								0.86774	STAT1
								0.86808	PDCD4
								0.86841	FAM151B
								0.86875	C10orf105
								0.86908	LOC100133091
								0.86942	TMEM129
								0.86975	LOC100288750
								0.87009	UBN2
								0.87043	C2orf34
								0.87076	ANO8
								0.87110	TAF5
								0.87143	LOC641298
								0.87177	SLC35E2
								0.87210	GRHPR
								0.87244	IFNGR1
								0.87278	LOC153546
								0.87311	ZNF514
								0.87345	ARPC1B
								0.87378	TMEM60
								0.87412	ZNF267
								0.87445	ABHD11
								0.87479	DDX20
								0.87513	KRTAP5-7
								0.87546	CSDA
								0.87580	C18orf23
								0.87613	CCDC93
								0.87647	SYNCRIP
								0.87680	PET112L
								0.87714	PDE7B
								0.87748	SLC26A4
								0.87781	CCDC68
								0.87815	BCL2A1

								0.87848	ZNF271
								0.87882	LOC728142
								0.87915	TMCO7
								0.87949	ETFB
								0.87983	LOC400657
								0.88016	FBXO48
								0.88050	LOC100130360
								0.88083	ZNF525
								0.88117	DUSP2
								0.88150	RIT1
								0.88184	LOC151171
								0.88218	ZDHHC1
								0.88251	ZW10
								0.88285	JRKL
								0.88318	TCEAL7
								0.88352	C16orf88
								0.88385	EIF1AX
								0.88419	EEFSEC
								0.88453	NUP54
								0.88486	ZNF468
								0.88520	BEND4
								0.88553	ZDHHC11
								0.88587	B3GAT3
								0.88620	LOC339400
								0.88654	GRK5
								0.88687	ZNF169
								0.88721	TECPR1
								0.88755	BRD9
								0.88788	FTSJ2
								0.88822	FIGF
								0.88855	EHMT2
								0.88889	ZMYND10
								0.88922	SCYL3
								0.88956	ABCD2
								0.88990	LOC100132356
								0.89023	C15orf59
								0.89057	LOC100132292
								0.89090	FOXK1
								0.89124	BRMS1
								0.89157	BAI1
								0.89191	RMND5B
								0.89225	C19orf22

								0.89258	FUT4
								0.89292	NR2F1
								0.89325	NLRP2
								0.89359	RPL27A
								0.89392	MYOZ2
								0.89426	KIAA0509
								0.89460	BMP4
								0.89493	LOC202181
								0.89527	PLEKHA3
								0.89560	ATP5L2
								0.89594	TAS2R10
								0.89627	C13orf18
								0.89661	ZNF155
								0.89695	TPCN2
								0.89728	CHRNA2
								0.89762	ZNF836
								0.89795	SATL1
								0.89829	DNAJC24
								0.89862	SVEP1
								0.89896	CCDC129
								0.89930	ZNF518B
								0.89963	FAM96B
								0.89997	C4orf42
								0.90030	C1orf93
								0.90064	DHDDS
								0.90097	NDUFB11
								0.90131	VCL
								0.90164	CTTNBP2NL
								0.90198	FAM86C
								0.90232	RAP1B
								0.90265	NEK4
								0.90299	CYR61
								0.90332	UCHL3
								0.90366	FLJ44124
								0.90399	FLJ25917
								0.90433	FAM120A
								0.90467	TYW1B
								0.90500	ACVR2B
								0.90534	CLNS1A
								0.90567	LOC100287743
								0.90601	ZMYM1
								0.90634	FAM57A

								0.90668	DGKD
								0.90702	ZBTB8OS
								0.90735	ZNF589
								0.90769	BCDIN3D
								0.90802	RFT1
								0.90836	RRP7A
								0.90869	MAP2K6
								0.90903	SLC35E4
								0.90937	PPIL5
								0.90970	C9orf24
								0.91004	C12orf64
								0.91037	KLHL21
								0.91071	TAS2R14
								0.91104	C14orf23
								0.91138	ARHGAP29
								0.91172	ZNF320
								0.91205	PFDN6
								0.91239	C5orf34
								0.91272	XRCC4
								0.91306	KIAA0174
								0.91339	MECR
								0.91373	SLC7A4
								0.91407	CCDC144A
								0.91440	TF
								0.91474	ZFY
								0.91507	MGAT2
								0.91541	SNORD60
								0.91574	SOCS4
								0.91608	ZNF587
								0.91641	SURF6
								0.91675	GGCT
								0.91709	ZNF749
								0.91742	UBAP2
								0.91776	OR7A5
								0.91809	RND3
								0.91843	PVRIG
								0.91876	PTPLA
								0.91910	C1orf91
								0.91944	DDHD1
								0.91977	DEM1
								0.92011	ZNF174
								0.92044	CCND1

								0.92078	CHST5
								0.92111	GCNT1
								0.92145	LOXL2
								0.92179	ZNF736
								0.92212	INTS1
								0.92246	MRPS22
								0.92279	LOC285375
								0.92313	ZNF766
								0.92346	CDHR3
								0.92380	C12orf64
								0.92414	MTX1
								0.92447	FGFR1OP2
								0.92481	LOC100287123
								0.92514	FIGNL1
								0.92548	LSM10
								0.92581	KIF1B
								0.92615	SPAG5
								0.92649	PER1
								0.92682	SYS1
								0.92716	BAG1
								0.92749	RPS3
								0.92783	PEX16
								0.92816	ATG9B
								0.92850	LOC645431
								0.92884	MRPS18A
								0.92917	C1orf85
								0.92951	ZFP161
								0.92984	TRAPPC2L
								0.93018	VWA5B2
								0.93051	C12orf64
								0.93085	LOC645676
								0.93118	C21orf88
								0.93152	GADD45A
								0.93186	LOC727993
								0.93219	MET
								0.93253	INO80D
								0.93286	PPIL1
								0.93320	TMEM22
								0.93353	OCLM
								0.93387	HAPLN1
								0.93421	ATPIF1
								0.93454	DCAF7

								0.93488	PTGES2
								0.93521	ARL6IP4
								0.93555	TOP1MT
								0.93588	CCDC142
								0.93622	DCI
								0.93656	RAP2C
								0.93689	USP27X
								0.93723	C4orf3
								0.93756	WDR78
								0.93790	FAM35A
								0.93823	FAM161A
								0.93857	C2orf18
								0.93891	ERV3
								0.93924	USH1G
								0.93958	RRAGD
								0.93991	FLJ41757
								0.94025	TAC1
								0.94058	L1TD1
								0.94092	IGSF9B
								0.94126	LOC339505
								0.94159	FAM76A
								0.94193	TRAF3
								0.94226	TTR
								0.94260	ZNF443
								0.94293	NDUFB1
								0.94327	WRB
								0.94361	STARD3NL
								0.94394	TIMM13
								0.94428	ZNF585B
								0.94461	ADAM17
								0.94495	EYA3
								0.94528	MXD4
								0.94562	SLC22A5
								0.94596	KIAA0090
								0.94629	MNS1
								0.94663	SCO1
								0.94696	GHRL
								0.94730	C4orf22
								0.94763	C8orf33
								0.94797	ZNF141
								0.94830	FZR1
								0.94864	PCDHB7

0.94898	VWA3A
0.94931	PSMC1
0.94965	AIMP2
0.94998	ANKRD54
0.95032	RWDD4A
0.95065	C1orf212
0.95099	TRIB2
0.95133	C12orf64
0.95166	RCCD1
0.95200	ESYT3
0.95233	LOC642236
0.95267	PP12708
0.95300	PGR
0.95334	DDIT3
0.95368	NSFP1
0.95401	TOMM7
0.95435	PAQR7
0.95468	LOC400680
0.95502	NDUFS6
0.95535	KLHL23
0.95569	ZBTB39
0.95603	FOXP4
0.95636	LOC100132167
0.95670	NOP2
0.95703	LOC100289338
0.95737	C19orf44
0.95770	ZNF558
0.95804	CLK2
0.95838	CROCCL1
0.95871	SFT2D3
0.95905	C14orf153
0.95938	FLJ40453
0.95972	ADRB1
0.96005	IGFL2
0.96039	GRPR
0.96073	GPSM3
0.96106	C3orf62
0.96140	HTR1E
0.96173	NUDT19
0.96207	LOC284408
0.96240	ZNF266
0.96274	HCG4

0.96307	SCMH1
0.96341	BET1
0.96375	ZC3HAV1L
0.96408	KLF10
0.96442	LRFN2
0.96475	EIF3D
0.96509	FASTK
0.96542	HIST1H2AD
0.96576	DBNL
0.96610	ATP5E
0.96643	CDKN2B
0.96677	NARFL
0.96710	ZNF391
0.96744	C8orf50
0.96777	MAGOHB
0.96811	RBBP9
0.96845	LOC285456
0.96878	FOXD4L3
0.96912	JUNB
0.96945	POLN
0.96979	MTPN
0.97012	KIAA2022
0.97046	ZNF439
0.97080	LOC100130950
0.97113	HIRIP3
0.97147	ZNF518B
0.97180	LOC100128164
0.97214	KLHL14
0.97247	ZNF438
0.97281	C8orf4
0.97315	LOC344065
0.97348	LOC100286934
0.97382	GP1BB
0.97415	PCDHB8
0.97449	LOC284023
0.97482	PINK1
0.97516	SPRY4
0.97550	TRMU
0.97583	RFC5
0.97617	XKR7
0.97650	KIAA1671
0.97684	KLF8

								0.97717	RGS8
								0.97751	BICC1
								0.97784	LOC286382
								0.97818	CYP2C8
								0.97852	MFI2
								0.97885	ZNF426
								0.97919	LOC151760
								0.97952	SEMA4G
								0.97986	HNRNPA1L2
								0.98019	UNC119B
								0.98053	PINK1
								0.98087	AURKC
								0.98120	TGFBI
								0.98154	PXMP2
								0.98187	RANGRF
								0.98221	ASXL3
								0.98254	C20orf74
								0.98288	LOC646470
								0.98322	C12orf64
								0.98355	ST14
								0.98389	LOC100287552
								0.98422	RPL11
								0.98456	LOC100127984
								0.98489	FAM169A
								0.98523	C15orf37
								0.98557	GPR107
								0.98590	ZNF799
								0.98624	FLJ39534
								0.98657	LOC441455
								0.98691	RING1
								0.98724	ACOT13
								0.98758	C11orf74
								0.98792	C14orf179
								0.98825	EFCAB2
								0.98859	C3orf31
								0.98892	FBXO17
								0.98926	ZNF440
								0.98959	CHMP6
								0.98993	LOC100289230
								0.99027	ZNF434
								0.99060	EFCAB2
								0.99094	SLC46A1

								0.99127	ZNF614	
								0.99161	PCDHB6	
								0.99194	PRPH2	
								0.99228	LOC254100	
								0.99261	TRIM11	
								0.99295	SIK1	
								0.99329	C21orf15	
								0.99362	NAALADL2	
								0.99396	ZNF789	
								0.99429	CORO1B	
								0.99463	ERVK2	
								0.99496	ZNF789	
								0.99530	FLAD1	
								0.99564	MED26	
								0.99597	GUSBP1	
								0.99631	NLE1	
								0.99664	RASD1	
								0.99698	NFYC	
								0.99731	GALNT3	
								0.99765	LOC100289263	
								0.99799	TBK1	
								0.99832	LOC100130906	
								0.99866	C2orf40	
								0.99899	C13orf26	
								0.99933	MAMSTR	
								0.99966	FOS	

ST 2. Functional annotation for BPs, CCs and MFs for the black module in FCTX. Summary of functional annotation analysis for the black module in FCTX. All significant terms are shown (p<0.05) and those with p<10-4 are highlighted in grey. BPs=biological processes; CCs= cellular compartments; MFs=molecular functions. The terms where our genes of interest are included are indicated with a 'x'. In addition, the protein-protein interactors (PPIs; see **Table IV**) of our genes of interest and their distribution within the terms are also indicated.

BPs											
**p-value**	**term ID**	**term name**	**Distribution of genes of interest (and PPIs) within terms**								
			MAPT	*MARK2*	*MARK4*	*EP300*	***GRN***	*ATN1*	*SGTA*	*CRKL*	*TLE3*
2.57E-05	GO:0097659	nucleic acid-templated transcription				x		x			x
2.77E-05	GO:0006351	transcription, DNA-templated				x		x			x
3.17E-05	GO:0032774	RNA biosynthetic process				x		x			x
6.53E-05	GO:2000112	regulation of cellular macromolecule biosynthetic process				x		x			x
6.91E-05	GO:0006366	transcription from RNA polymerase II promoter				x		x			
7.07E-05	GO:0006325	chromatin organization				x					
9.45E-05	GO:0016568	chromatin modification				x					
1.21E-04	GO:0010556	regulation of macromolecule biosynthetic process				x		x			x
1.59E-04	GO:0010629	negative regulation of gene expression				x		x			
2.04E-04	GO:0018205	peptidyl-lysine modification				x					
2.05E-04	GO:0090304	nucleic acid metabolic process				x		x			x
2.55E-04	GO:0019219	regulation of nucleobase-containing compound metabolic process				x		x			x
2.69E-04	GO:1903507	negative regulation of nucleic acid-templated transcription				x		x			
2.79E-04	GO:0080090	regulation of primary metabolic process		x		x		x		x	x
2.81E-04	GO:0034645	cellular macromolecule biosynthetic process				x		x			x
2.91E-04	GO:0051252	regulation of RNA metabolic process				x		x			x
3.32E-04	GO:0045892	negative regulation of transcription, DNA-templated				x		x			
3.79E-04	GO:0051253	negative regulation of RNA metabolic process				x		x			
3.94E-04	GO:2001141	regulation of RNA biosynthetic process				x		x			x
4.20E-04	GO:0006355	regulation of transcription, DNA-templated				x		x			x
4.30E-04	GO:0045934	negative regulation of nucleobase-containing compound metabolic process				x		x			
4.46E-04	GO:1903506	embryo development ending in birth or egg hatching				x		x			x
4.50E-04	GO:1902679	negative regulation of RNA biosynthetic process				x		x			
4.61E-04	GO:0031323	regulation of cellular metabolic process	**x**	x		x		x		x	x
4.62E-04	GO:0043009	chordate embryonic development				x	**x**				
4.71E-04	GO:0031326	regulation of cellular biosynthetic process				x		x			x
5.02E-04	GO:0009059	macromolecule biosynthetic process				x		x			x
5.88E-04	GO:0009792	embryo development ending in birth or egg hatching				x	**x**				
7.10E-04	GO:0016070	RNA metabolic process				x		x			x
7.51E-04	GO:0016570	histone modification				x					
7.63E-04	GO:0044260	cellular macromolecule metabolic process		x	x	x		x		x	x
7.63E-04	GO:0034654	nucleobase-containing compound biosynthetic process				x		x			x
1.02E-03	GO:0016569	covalent chromatin modification				x					

1.04E-03	GO:0009889	regulation of biosynthetic process				x		x			x
1.05E-03	GO:2000113	negative regulation of cellular macromolecule biosynthetic process				x		x			
1.49E-03	GO:0031324	negative regulation of cellular metabolic process				x		x			
1.60E-03	GO:0010558	negative regulation of macromolecule biosynthetic process				x		x			
1.70E-03	GO:0010468	regulation of gene expression				x		x			x
1.84E-03	GO:0018130	heterocycle biosynthetic process				x		x			x
2.02E-03	GO:0019438	aromatic compound biosynthetic process				x		x			x
2.02E-03	GO:0051171	regulation of nitrogen compound metabolic process				x		x			x
2.34E-03	GO:0016573	histone acetylation				x					
2.39E-03	GO:0060255	regulation of macromolecule metabolic process		x		x		x		x	x
2.59E-03	GO:0031327	negative regulation of cellular biosynthetic process				x		x			
3.08E-03	GO:0010467	gene expression				x		x			x
3.22E-03	GO:1901362	organic cyclic compound biosynthetic process				x		x			x
3.54E-03	GO:0018393	internal peptidyl-lysine acetylation				x					
3.57E-03	GO:0000122	negative regulation of transcription from RNA polymerase II promoter				x		x			
3.73E-03	GO:0010605	negative regulation of macromolecule metabolic process				x		x			
3.81E-03	GO:0019222	regulation of metabolic process	**x**	x		x		x		x	x
4.29E-03	GO:0044271	cellular nitrogen compound biosynthetic process				x		x			x
4.61E-03	GO:0009890	negative regulation of biosynthetic process				x		x			
4.89E-03	GO:0006357	regulation of transcription from RNA polymerase II promoter				x		x			
5.59E-03	GO:0043543	protein acylation				x					
5.94E-03	GO:0051172	negative regulation of nitrogen compound metabolic process				x		x			
6.02E-03	GO:0018394	peptidyl-lysine acetylation				x					
6.02E-03	GO:0006475	internal protein amino acid acetylation				x					
6.80E-03	GO:0051276	chromosome organization				x					
7.62E-03	GO:1901576	organic substance biosynthetic process				x		x			x
8.22E-03	GO:0043170	macromolecule metabolic process		x	x	x		x		x	x
8.69E-03	GO:0009892	negative regulation of metabolic process				x		x			
8.75E-03	GO:0009058	biosynthetic process				x		x			x
9.37E-03	GO:0044238	primary metabolic process		x	x	x		x		x	x
1.03E-02	GO:0044249	cellular biosynthetic process				x		x			x
1.04E-02	GO:0006139	nucleobase-containing compound metabolic process				x		x			x
3.01E-02	GO:0046483	heterocycle metabolic process				x		x			x
3.80E-02	GO:0006725	cellular aromatic compound metabolic process				x		x			x
4.41E-02	GO:0006473	protein acetylation				x					
4.52E-02	GO:0030518	intracellular steroid hormone receptor signaling pathway				x					
4.86E-02	GO:0006996	organelle organization	**x**	x	x	x					

CCs

p-value	term ID	term name	Distribution of genes of interest (and PPIs) within terms								
			MAPT	*MARK2*	*MARK4*	*EP300*	***GRN***	*ATN1*	*SGTA*	*CRKL*	*TLE3*
2.81E-13	GO:0031981	nuclear lumen	**x**			x		x			x

1.85E-12	GO:0070013	intracellular organelle lumen	**x**			x		x			x
3.89E-12	GO:0005654	nucleoplasm				x		x			x
5.17E-12	GO:0043233	organelle lumen	**x**			x		x			x
2.73E-11	GO:0031974	membrane-enclosed lumen	**x**			x		x			x
5.13E-11	GO:0044428	nuclear part	**x**			x		x			x
1.00E-08	GO:0005634	nucleus	**x**	x		x		x			x
4.26E-08	GO:0044451	nucleoplasm part				x					
2.14E-07	GO:0044446	intracellular organelle part	**x**	x	x	x		x			x
3.20E-07	GO:0035097	histone methyltransferase complex									
7.53E-07	GO:0043231	intracellular membrane-bounded organelle	**x**	x		x	**x**	x		x	x
3.59E-06	GO:0044422	organelle part	**x**	x	x	x		x			x
4.17E-06	GO:0071339	MLL1 complex									
4.17E-06	GO:0044665	MLL1/2 complex									
1.09E-05	GO:0034708	methyltransferase complex									
2.98E-05	GO:1990234	transferase complex				x					
7.60E-05	GO:0043229	intracellular organelle	**x**	x	x	x	**x**	x		x	x
1.76E-04	GO:0000785	chromatin				x					
1.14E-03	GO:0005730	nucleolus									
3.47E-03	GO:1902494	catalytic complex				x					
5.36E-03	GO:0044424	intracellular part	**x**	x	x	x	**x**	x	x	x	x
1.12E-02	GO:0005622	intracellular	**x**	x	x	x	**x**	x	x	x	x
1.36E-02	GO:0000790	nuclear chromatin				x					
1.62E-02	GO:0043227	membrane-bounded organelle	**x**	x		x	**x**	x		x	x
4.11E-02	GO:0043232	intracellular non-membrane-bounded organelle	**x**	x	x	x					
4.11E-02	GO:0043228	non-membrane-bounded organelle	**x**	x	x	x					
4.45E-02	GO:0044454	nuclear chromosome part				x					

MFs

p-value	term ID	term name	Distribution of genes of interest (and PPIs) within terms								
			MAPT	*MARK2*	*MARK4*	*EP300*	***GRN***	*ATN1*	*SGTA*	*CRKL*	*TLE3*
6.26E-06	GO:0000989	transcription factor binding transcription factor activity				x		x			
7.19E-06	GO:0003676	nucleic acid binding		x		x	**x**			x	
7.25E-06	GO:0000988	protein binding transcription factor activity				x		x			
4.42E-05	GO:0003712	transcription cofactor activity				x		x			
1.11E-03	GO:0008134	transcription factor binding				x					
4.54E-03	GO:0003682	chromatin binding				x					

6.78E-03	GO:0003677	DNA binding				x					
1.02E-02	GO:0003714	transcription corepressor activity						x			
2.32E-02	GO:0005515	protein binding	**x**	x	x	x		x	x	x	x
2.84E-02	GO:0044212	transcription regulatory region DNA binding				x					
2.85E-02	GO:0044822	poly(A) RNA binding		x			**x**			x	
3.32E-02	GO:0000975	regulatory region DNA binding				x					
3.32E-02	GO:0001067	regulatory region nucleic acid binding				x					
3.41E-02	GO:0051427	hormone receptor binding				x					

ST 3. Functional annotation for BPs, CCs and MFs for the darkolivegreen module in FCTX. Summary of functional annotation analysis for the darkolivegreen module in FCTX. All significant terms are shown (p<0.05) and those with p<10-4 are highlighted in grey. CCs= cellular compartments (only data available). The terms where our gene of interest is included are indicated with a 'x'.

CCs			
**p-value**	**term ID**	**term name**	**Distribution of genes of interest within terms**
			CHMP2B
9.26E-05	GO:0044444	cytoplasmic part	**x**
3.76E-04	GO:0005737	cytoplasm	**x**
6.81E-03	GO:0098588	bounding membrane of organelle	**x**

ST 4. Functional annotation for BPs, CCs and MFs for the darkred module in FCTX. Summary of functional annotation analysis for the darkred module in FCTX. All significant terms are shown (p<0.05) and those with p<10-4 are highlighted in grey. BPs=biological processes; CCs= cellular compartments; MFs=molecular functions. The terms where our genes of interest are included are indicated with a 'x'. In addition, the protein-protein interactors (PPIs; see **Table IV**) of our genes of interest and their distribution within the terms are also indicated.

BPs							
**p-value**	**term ID**	**term name**	**Distribution of genes of interest (and PPIs) within terms**				
			HLA-DRA	***CTSC***	*HLA-DMB*	*HLA-DMA*	*CD74*
1.39E-31	GO:0002376	immune system process	**x**	**x**	x	x	x
3.62E-31	GO:0006955	immune response	**x**	**x**	x	x	x
5.23E-29	GO:0006952	defense response	**x**				x
8.25E-25	GO:0002684	positive regulation of immune system process	**x**		x	x	x
3.30E-24	GO:0002682	regulation of immune system process	**x**		x	x	x
2.29E-23	GO:0050778	positive regulation of immune response	**x**		x	x	x
5.75E-22	GO:0002253	activation of immune response	**x**				
6.17E-22	GO:0050776	regulation of immune response	**x**		x	x	x
3.38E-21	GO:0045321	leukocyte activation	**x**		x	x	x
5.16E-21	GO:0001775	cell activation	**x**		x	x	x
2.51E-19	GO:0045087	innate immune response	**x**				
4.74E-18	GO:0002252	immune effector process		**x**	x	x	x
8.29E-18	GO:0002757	immune response-activating signal transduction	**x**				
2.50E-16	GO:0006950	response to stress	**x**				x
7.34E-15	GO:0002764	immune response-regulating signaling pathway	**x**				
4.17E-14	GO:0046651	lymphocyte proliferation			x		x
4.23E-14	GO:0050896	response to stimulus	**x**	**x**	x	x	x
4.69E-14	GO:0032943	mononuclear cell proliferation			x		x
1.30E-13	GO:0070661	leukocyte proliferation			x		x
1.92E-13	GO:0050865	regulation of cell activation	**x**		x	x	x
2.00E-13	GO:0046649	lymphocyte activation	**x**		x	x	x
3.55E-13	GO:0002694	regulation of leukocyte activation	**x**		x	x	x
1.82E-12	GO:0048584	positive regulation of response to stimulus	**x**	**x**	x	x	x
3.60E-12	GO:0050867	positive regulation of cell activation	**x**		x	x	x
5.78E-12	GO:0048583	regulation of response to stimulus	**x**	**x**	x	x	x
1.92E-11	GO:0050670	regulation of lymphocyte proliferation			x		x
2.06E-11	GO:0002696	positive regulation of leukocyte activation	**x**		x	x	x
2.14E-11	GO:0051249	regulation of lymphocyte activation	**x**		x	x	x
2.18E-11	GO:0032944	regulation of mononuclear cell proliferation			x		x
2.44E-11	GO:0050900	leukocyte migration					x
4.01E-11	GO:0070663	regulation of leukocyte proliferation			x		x
8.70E-11	GO:0034341	response to interferon-gamma	**x**				
2.62E-10	GO:0002429	immune response-activating cell surface receptor signaling pathway	**x**				
3.13E-10	GO:0007159	leukocyte cell-cell adhesion	**x**		x	x	x

6.02E-10	GO:0002274	myeloid leukocyte activation					x
6.57E-10	GO:0051251	positive regulation of lymphocyte activation	**x**		x	x	x
1.02E-09	GO:0034109	homotypic cell-cell adhesion	**x**		x	x	x
1.10E-09	GO:0006954	inflammatory response					
1.27E-09	GO:0042098	T cell proliferation			x		
2.23E-09	GO:0048518	positive regulation of biological process	**x**	**x**	x	x	x
2.30E-09	GO:0002521	leukocyte differentiation				x	x
3.20E-09	GO:0071345	cellular response to cytokine stimulus	**x**				x
4.15E-09	GO:0051716	cellular response to stimulus	**x**	**x**			x
4.52E-09	GO:0042110	T cell activation	**x**		x	x	x
4.52E-09	GO:0070489	T cell aggregation	**x**		x	x	x
4.52E-09	GO:0034097	response to cytokine	**x**				x
4.53E-09	GO:0071346	cellular response to interferon-gamma	**x**				
4.80E-09	GO:0071593	lymphocyte aggregation	**x**		x	x	x
6.10E-09	GO:0070486	leukocyte aggregation	**x**		x	x	x
1.27E-08	GO:0007165	signal transduction	**x**	**x**			x
1.32E-08	GO:0016337	single organismal cell-cell adhesion	**x**		x	x	x
1.77E-08	GO:0050870	positive regulation of T cell activation	**x**		x	x	x
1.96E-08	GO:1903039	positive regulation of leukocyte cell-cell adhesion	**x**		x	x	x
2.16E-08	GO:0034112	positive regulation of homotypic cell-cell adhesion	**x**		x	x	x
2.75E-08	GO:0009611	response to wounding					
3.90E-08	GO:0002768	immune response-regulating cell surface receptor signaling pathway	**x**				
5.56E-08	GO:0098602	single organism cell adhesion	**x**		x	x	x
6.15E-08	GO:0007155	cell adhesion	**x**		x	x	x
6.64E-08	GO:0022610	biological adhesion	**x**		x	x	x
8.80E-08	GO:0050671	positive regulation of lymphocyte proliferation			x		x
9.06E-08	GO:0060333	interferon-gamma-mediated signaling pathway	**x**				
1.01E-07	GO:0032946	positive regulation of mononuclear cell proliferation			x		x
1.46E-07	GO:0001817	regulation of cytokine production					x
1.49E-07	GO:0070665	positive regulation of leukocyte proliferation			x		x
1.53E-07	GO:0050863	regulation of T cell activation	**x**		x	x	x
1.60E-07	GO:0030097	hemopoiesis				x	x
1.71E-07	GO:0002250	adaptive immune response		**x**		x	x
1.71E-07	GO:0022409	positive regulation of cell-cell adhesion	**x**		x	x	x
2.03E-07	GO:0002520	immune system development				x	x
2.06E-07	GO:1903037	regulation of leukocyte cell-cell adhesion	**x**		x	x	x
2.16E-07	GO:0001816	cytokine production					x
2.65E-07	GO:0044700	single organism signaling	**x**	**x**			x
2.81E-07	GO:0023052	signaling	**x**	**x**			x
3.30E-07	GO:0019221	cytokine-mediated signaling pathway	**x**				x
3.40E-07	GO:0034110	regulation of homotypic cell-cell adhesion	**x**		x	x	x
4.20E-07	GO:0048534	hematopoietic or lymphoid organ development				x	x

4.26E-07	GO:0043207	response to external biotic stimulus					
4.26E-07	GO:0051707	response to other organism					
4.52E-07	GO:0050851	antigen receptor-mediated signaling pathway	**x**				
5.47E-07	GO:0002495	antigen processing and presentation of peptide antigen via MHC class II	**x**		x	x	x
6.06E-07	GO:0007154	cell communication	**x**	**x**			x
6.27E-07	GO:0002504	antigen processing and presentation of peptide or polysaccharide antigen via MHC class II	**x**		x	x	x
7.70E-07	GO:1903706	regulation of hemopoiesis				x	x
8.81E-07	GO:0031347	regulation of defense response					
1.17E-06	GO:0009607	response to biotic stimulus					
1.24E-06	GO:0007166	cell surface receptor signaling pathway	**x**				x
1.37E-06	GO:0006959	humoral immune response					
1.50E-06	GO:1902105	regulation of leukocyte differentiation				x	x
2.56E-06	GO:0016064	immunoglobulin mediated immune response				x	x
2.79E-06	GO:0042116	macrophage activation					x
2.81E-06	GO:0042129	regulation of T cell proliferation			x		
3.39E-06	GO:0019724	B cell mediated immunity				x	x
4.23E-06	GO:0009605	response to external stimulus					x
4.32E-06	GO:0002683	negative regulation of immune system process					x
4.49E-06	GO:0051240	positive regulation of multicellular organismal process				x	x
5.13E-06	GO:0001819	positive regulation of cytokine production					x
6.08E-06	GO:0002443	leukocyte mediated immunity		**x**		x	x
7.04E-06	GO:0002366	leukocyte activation involved in immune response			x		
7.04E-06	GO:0002263	cell activation involved in immune response			x		
7.49E-06	GO:0019886	antigen processing and presentation of exogenous peptide antigen via MHC class II	**x**		x	x	x
8.01E-06	GO:0048002	antigen processing and presentation of peptide antigen	**x**		x	x	x
8.63E-06	GO:0042102	positive regulation of T cell proliferation			x		
9.95E-06	GO:0022407	regulation of cell-cell adhesion	**x**		x	x	x
1.09E-05	GO:0050864	regulation of B cell activation					x
1.25E-05	GO:0030595	leukocyte chemotaxis					x
1.49E-05	GO:0031349	positive regulation of defense response					
1.49E-05	GO:0070887	cellular response to chemical stimulus	**x**				x
1.80E-05	GO:0032103	positive regulation of response to external stimulus					x
2.11E-05	GO:0002478	antigen processing and presentation of exogenous peptide antigen	**x**		x	x	x
2.21E-05	GO:0098542	defense response to other organism					
2.22E-05	GO:0030593	neutrophil chemotaxis					x
2.44E-05	GO:0097529	myeloid leukocyte migration					x
2.53E-05	GO:0002697	regulation of immune effector process			x		x
2.66E-05	GO:0002275	myeloid cell activation involved in immune response					
3.12E-05	GO:0019884	antigen processing and presentation of exogenous antigen	**x**		x	x	x
3.75E-05	GO:1990266	neutrophil migration					x
3.89E-05	GO:0030155	regulation of cell adhesion	**x**		x	x	x
3.92E-05	GO:0002460	adaptive immune response based on somatic recombination of immune receptors built from immunoglobulin superfamily domains		**x**		x	x

4.09E-05	GO:0016477	cell migration					x
5.89E-05	GO:0002224	toll-like receptor signaling pathway					
6.79E-05	GO:0019882	antigen processing and presentation	**x**		x	x	x
7.03E-05	GO:0030099	myeloid cell differentiation					
9.29E-05	GO:0042113	B cell activation					x
1.01E-04	GO:0002699	positive regulation of immune effector process			x		x
1.12E-04	GO:0032101	regulation of response to external stimulus					x
1.29E-04	GO:0071621	granulocyte chemotaxis					x
1.39E-04	GO:0045785	positive regulation of cell adhesion	**x**		x	x	x
1.54E-04	GO:0048870	cell motility					x
1.54E-04	GO:0051674	localization of cell					x
2.17E-04	GO:0065007	biological regulation	**x**	**x**	x	x	x
2.21E-04	GO:0002449	lymphocyte mediated immunity		**x**		x	x
2.23E-04	GO:0097530	granulocyte migration					x
2.54E-04	GO:0045621	positive regulation of lymphocyte differentiation				x	x
2.62E-04	GO:0009617	response to bacterium					
2.74E-04	GO:0002221	pattern recognition receptor signaling pathway					
2.94E-04	GO:0002573	myeloid leukocyte differentiation					
3.15E-04	GO:0002758	innate immune response-activating signal transduction					
3.37E-04	GO:0060326	cell chemotaxis					x
3.58E-04	GO:0045088	regulation of innate immune response					
3.70E-04	GO:0050729	positive regulation of inflammatory response					
3.71E-04	GO:0051239	regulation of multicellular organismal process				x	x
4.30E-04	GO:0042221	response to chemical	**x**	**x**			x
4.35E-04	GO:1903708	positive regulation of hemopoiesis				x	x
4.43E-04	GO:0002218	activation of innate immune response					
4.53E-04	GO:0030225	macrophage differentiation					
4.64E-04	GO:0030098	lymphocyte differentiation				x	x
5.76E-04	GO:0051607	defense response to virus					
5.91E-04	GO:0030888	regulation of B cell proliferation					x
6.42E-04	GO:0040011	locomotion					x
6.64E-04	GO:1902107	positive regulation of leukocyte differentiation				x	x
6.80E-04	GO:0007596	blood coagulation					
7.03E-04	GO:0002455	humoral immune response mediated by circulating immunoglobulin					
7.73E-04	GO:0050817	coagulation					
7.93E-04	GO:0050852	T cell receptor signaling pathway	**x**				
7.95E-04	GO:0050871	positive regulation of B cell activation					x
7.98E-04	GO:0007599	hemostasis					
8.65E-04	GO:0045619	regulation of lymphocyte differentiation				x	x
1.06E-03	GO:0045058	T cell selection				x	x
1.11E-03	GO:0042060	wound healing					
1.14E-03	GO:0042089	cytokine biosynthetic process					

1.17E-03	GO:0002283	neutrophil activation involved in immune response					
1.17E-03	GO:0002281	macrophage activation involved in immune response					
1.17E-03	GO:0001774	microglial cell activation					
1.21E-03	GO:0045582	positive regulation of T cell differentiation				x	x
1.32E-03	GO:1903036	positive regulation of response to wounding					
1.38E-03	GO:0031294	lymphocyte costimulation	**x**				
1.38E-03	GO:0031295	T cell costimulation	**x**				
1.40E-03	GO:0042107	cytokine metabolic process					
1.86E-03	GO:0002503	peptide antigen assembly with MHC class II protein complex	**x**		x	x	
1.86E-03	GO:0002399	MHC class II protein complex assembly	**x**		x	x	
2.16E-03	GO:0050789	regulation of biological process	**x**	**x**	x	x	x
2.20E-03	GO:0032729	positive regulation of interferon-gamma production					
2.30E-03	GO:0042100	B cell proliferation					x
2.33E-03	GO:0009615	response to virus					
2.55E-03	GO:0045089	positive regulation of innate immune response					
2.83E-03	GO:0080134	regulation of response to stress					x
3.07E-03	GO:0050853	B cell receptor signaling pathway					
3.67E-03	GO:0050730	regulation of peptidyl-tyrosine phosphorylation					x
4.11E-03	GO:0032675	regulation of interleukin-6 production					
4.21E-03	GO:0042108	positive regulation of cytokine biosynthetic process					
4.50E-03	GO:0050878	regulation of body fluid levels					
4.89E-03	GO:1901570	fatty acid derivative biosynthetic process					x
4.89E-03	GO:0046456	icosanoid biosynthetic process					x
4.98E-03	GO:0050794	regulation of cellular process	**x**	**x**	x	x	x
5.02E-03	GO:0044707	single-multicellular organism process	**x**			x	x
5.38E-03	GO:0045059	positive thymic T cell selection				x	x
5.68E-03	GO:0032635	interleukin-6 production					
5.78E-03	GO:0045577	regulation of B cell differentiation					
6.20E-03	GO:0006909	phagocytosis					
7.03E-03	GO:0002695	negative regulation of leukocyte activation					x
7.09E-03	GO:0071216	cellular response to biotic stimulus					
7.25E-03	GO:0044699	single-organism process	**x**	**x**	x	x	x
7.39E-03	GO:0034154	toll-like receptor 7 signaling pathway					
7.39E-03	GO:0002396	MHC protein complex assembly	**x**		x	x	
7.39E-03	GO:0002501	peptide antigen assembly with MHC protein complex	**x**		x	x	
7.73E-03	GO:0042035	regulation of cytokine biosynthetic process					
8.01E-03	GO:0002888	positive regulation of myeloid leukocyte mediated immunity					
8.60E-03	GO:0045637	regulation of myeloid cell differentiation					
9.05E-03	GO:0032501	multicellular organismal process	**x**			x	x
1.16E-02	GO:0044763	single-organism cellular process	**x**	**x**	x	x	x
1.26E-02	GO:0006636	unsaturated fatty acid biosynthetic process					x
1.72E-02	GO:0050866	negative regulation of cell activation					x

1.79E-02	GO:0045580	regulation of T cell differentiation				x	x
1.84E-02	GO:0006928	movement of cell or subcellular component					x
1.85E-02	GO:0030890	positive regulation of B cell proliferation					x
2.25E-02	GO:0071219	cellular response to molecule of bacterial origin					
2.50E-02	GO:0051250	negative regulation of lymphocyte activation					x
2.54E-02	GO:0032649	regulation of interferon-gamma production					
2.66E-02	GO:1903034	regulation of response to wounding					
2.86E-02	GO:0046633	alpha-beta T cell proliferation					
2.94E-02	GO:0007229	integrin-mediated signaling pathway					
3.12E-02	GO:0050727	regulation of inflammatory response					
3.28E-02	GO:0008283	cell proliferation			x		x
3.49E-02	GO:0071310	cellular response to organic substance	**x**				x
3.64E-02	GO:0045588	positive regulation of gamma-delta T cell differentiation					
3.86E-02	GO:0002700	regulation of production of molecular mediator of immune response					x
4.06E-02	GO:0050848	regulation of calcium-mediated signaling					
4.06E-02	GO:0002702	positive regulation of production of molecular mediator of immune response					x
4.68E-02	GO:0046635	positive regulation of alpha-beta T cell activation					
4.74E-02	GO:0035588	G-protein coupled purinergic receptor signaling pathway					
4.74E-02	GO:0042119	neutrophil activation					
4.87E-02	GO:0030217	T cell differentiation				x	x
**CCs**							
**p-value**	**term ID**	**term name**	**Distribution of genes of interest (and PPIs) within terms**				
			HLA-DRA	***CTSC***	*HLA-DMB*	*HLA-DMA*	*CD74*
5.53E-08	GO:0016021	integral component of membrane	**x**		x	x	x
1.17E-07	GO:0042613	MHC class II protein complex	**x**		x	x	
1.54E-07	GO:0044425	membrane part	**x**		x	x	x
1.71E-07	GO:0031224	intrinsic component of membrane	**x**		x	x	x
4.50E-06	GO:0016020	membrane	**x**	**x**	x	x	x
1.62E-05	GO:0042611	MHC protein complex	**x**		x	x	
5.97E-05	GO:0031988	membrane-bounded vesicle	**x**	**x**		x	x
6.78E-05	GO:0031982	vesicle	**x**	**x**		x	x
7.69E-05	GO:0005764	lysosome	**x**	**x**	x	x	x
7.69E-05	GO:0000323	lytic vacuole	**x**	**x**	x	x	x
1.08E-04	GO:0005887	integral component of plasma membrane		**x**			
1.44E-04	GO:0098552	side of membrane	**x**				x
2.48E-04	GO:0031226	intrinsic component of plasma membrane	**x**				
2.76E-04	GO:0043235	receptor complex					x
5.42E-04	GO:0005773	vacuole	**x**	**x**	x	x	x

5.67E-04	GO:0030669	clathrin-coated endocytic vesicle membrane	**x**				x
1.16E-03	GO:0009986	cell surface	**x**			x	x
1.28E-03	GO:0045334	clathrin-coated endocytic vesicle	**x**				x
1.29E-03	GO:0009897	external side of plasma membrane					x
2.17E-03	GO:0071556	integral component of lumenal side of endoplasmic reticulum membrane	**x**				x
2.17E-03	GO:0098553	lumenal side of endoplasmic reticulum membrane	**x**				x
2.17E-03	GO:0098576	lumenal side of membrane	**x**				x
2.41E-03	GO:0030139	endocytic vesicle	**x**				x
6.67E-03	GO:1903561	extracellular vesicle	**x**	**x**		x	x
6.67E-03	GO:0070062	extracellular vesicular exosome	**x**	**x**		x	x
6.67E-03	GO:0043230	extracellular organelle	**x**	**x**		x	x
6.67E-03	GO:0065010	extracellular membrane-bounded organelle	**x**	**x**		x	x
7.39E-03	GO:0032009	early phagosome					
1.03E-02	GO:0005765	lysosomal membrane	**x**		x	x	x
1.17E-02	GO:0044437	vacuolar part	**x**		x	x	x
1.49E-02	GO:0030666	endocytic vesicle membrane	**x**				x
1.85E-02	GO:0012507	ER to Golgi transport vesicle membrane	**x**				x
2.19E-02	GO:0032588	trans-Golgi network membrane	**x**				x
2.79E-02	GO:0005576	extracellular region	**x**	**x**		x	x
3.64E-02	GO:0036019	endolysosome					
3.83E-02	GO:0005774	vacuolar membrane	**x**		x	x	x

MFs

p-value	term ID	term name	Distribution of genes of interest (and PPIs) within terms				
			HLA-DRA	***CTSC***	*HLA-DMB*	*HLA-DMA*	*CD74*
1.88E-08	GO:0004872	receptor activity	**x**				x
4.50E-07	GO:0004888	transmembrane signaling receptor activity	**x**				x
1.24E-06	GO:0038023	signaling receptor activity	**x**				x
6.45E-06	GO:0060089	molecular transducer activity	**x**				x
6.45E-06	GO:0004871	signal transducer activity	**x**				x
1.49E-04	GO:0003823	antigen binding	**x**		x	x	x
2.54E-04	GO:0019864	IgG binding					
5.58E-03	GO:0032403	protein complex binding	**x**		x	x	x
1.15E-02	GO:0023026	MHC class II protein complex binding	**x**		x	x	x
1.25E-02	GO:0001948	glycoprotein binding					
1.60E-02	GO:0019865	immunoglobulin binding					

2.16E-02	GO:0023023	MHC protein complex binding	**x**	x	x	x
3.64E-02	GO:0032395	MHC class II receptor activity	**x**			

ST 5. Functional annotation for BPs, CCs and MFs for the red module in FCTX. Summary of functional annotation analysis for the red module in FCTX. All significant terms are shown (p<0.05) and those with p<10-4 are highlighted in grey. BPs=biological processes; CCs= cellular compartments; MFs=molecular functions. The terms where our gene of interest is included are indicate with a 'x'. No protein-protein interactors (PPIs; see **Table IV**) of TMEM106B distributed within the terms.

BPs			
**p-value**	**term ID**	**term name**	**Distribution of genes of interest within terms**
			TMEM106B
4.43E-06	GO:0044260	cellular macromolecule metabolic process	
3.84E-05	GO:0044267	cellular protein metabolic process	
3.34E-04	GO:0044237	cellular metabolic process	
7.87E-04	GO:0043170	macromolecule metabolic process	
1.56E-03	GO:0044238	primary metabolic process	
1.22E-02	GO:0071704	organic substance metabolic process	
1.32E-02	GO:0006396	RNA processing	
1.88E-02	GO:0019538	protein metabolic process	
2.73E-02	GO:0008152	metabolic process	

CCs			
**p-value**	**term ID**	**term name**	**Distribution of genes of interest within terms**
			TMEM106B
2.83E-10	GO:0005622	intracellular	x
3.89E-09	GO:0044424	intracellular part	x
1.67E-07	GO:0043231	intracellular membrane-bounded organelle	x
2.63E-07	GO:0043227	membrane-bounded organelle	x
9.16E-07	GO:0044428	nuclear part	
1.10E-06	GO:0070013	intracellular organelle lumen	
2.29E-06	GO:0044422	organelle part	x
2.67E-06	GO:0043229	intracellular organelle	x
4.14E-06	GO:0044446	intracellular organelle part	x
4.37E-06	GO:0043233	organelle lumen	
5.03E-06	GO:0043226	organelle	x
6.22E-06	GO:0031974	membrane-enclosed lumen	
9.36E-06	GO:0031981	nuclear lumen	
1.89E-04	GO:0005634	nucleus	
4.74E-03	GO:0005737	cytoplasm	x
8.49E-03	GO:0005654	nucleoplasm	
8.65E-03	GO:0044464	cell part	x
8.65E-03	GO:0005623	cell	x
1.36E-02	GO:1902494	catalytic complex	
4.85E-02	GO:0044444	cytoplasmic part	x

MFs			
p-value	term ID	term name	Distribution of genes of interest within terms
1.96E-02	GO:0016874	ligase activity	***TMEM106B***

ST 6. Functional annotation for BPs, CCs and MFs for the lightyellow module in TCTX. Summary of functional annotation analysis for the lightyellow module in TCTX. All significant terms are shown (p<0.05) and those with p<10-4 are highlighted in grey. BPs=biological processes; CCs= cellular compartments; MFs=molecular functions. The terms where our genes of interest are included are indicated with a 'x'. In addition, the protein-protein interactors (PPIs; see **Table IV**) of our genes of interest and their distribution within the terms are also indicated.

BPs						
**p-value**	**term ID**	**term name**	**Distribution of genes of interest (and PPIs) within terms**			
			MAPT	*MARK2*	*EP300*	*AKT1*
1.23E-04	GO:0006366	transcription from RNA polymerase II promoter			x	x
3.66E-03	GO:0019222	regulation of metabolic process	**x**	x	x	x
6.34E-03	GO:0034645	cellular macromolecule biosynthetic process			x	x
7.22E-03	GO:2000112	regulation of cellular macromolecule biosynthetic process			x	x
9.83E-03	GO:0006357	regulation of transcription from RNA polymerase II promoter			x	x
1.16E-02	GO:0090304	nucleic acid metabolic process			x	x
1.27E-02	GO:0080090	regulation of primary metabolic process		x	x	x
1.54E-02	GO:0010557	positive regulation of macromolecule biosynthetic process			x	x
1.65E-02	GO:0051254	positive regulation of RNA metabolic process			x	x
1.83E-02	GO:0010556	regulation of macromolecule biosynthetic process			x	x
1.91E-02	GO:0060255	regulation of macromolecule metabolic process		x	x	x
2.10E-02	GO:0006351	transcription, DNA-templated			x	x
2.30E-02	GO:0009059	macromolecule biosynthetic process			x	x
2.41E-02	GO:0051252	regulation of RNA metabolic process			x	x
2.48E-02	GO:0044260	cellular macromolecule metabolic process		x	x	x
2.52E-02	GO:0097659	nucleic acid-templated transcription			x	x
2.62E-02	GO:0031323	regulation of cellular metabolic process	**x**	x	x	x
2.75E-02	GO:0048524	positive regulation of viral process			x	
3.75E-02	GO:0031326	regulation of cellular biosynthetic process			x	x
3.96E-02	GO:0016070	RNA metabolic process			x	x
4.00E-02	GO:0050792	regulation of viral process			x	
4.14E-02	GO:0019219	regulation of nucleobase-containing compound metabolic process			x	x
4.85E-02	GO:0045935	positive regulation of nucleobase-containing compound metabolic process			x	x
4.95E-02	GO:0008152	metabolic process	**x**	x	x	x

CCs						
**p-value**	**term ID**	**term name**	**Distribution of genes of interest (and PPIs) within terms**			
			MAPT	*MARK2*	*EP300*	*AKT1*
3.69E-05	GO:0005634	nucleus	**x**	x	x	x

8.95E-04	GO:0031981	nuclear lumen	**x**		x	x
2.20E-03	GO:0044451	nucleoplasm part			x	
2.90E-03	GO:0005654	nucleoplasm			x	x
3.63E-03	GO:0005622	intracellular	**x**	x	x	x
3.88E-03	GO:0044424	intracellular part	**x**	x	x	x
4.82E-03	GO:0044428	nuclear part	**x**		x	x
4.86E-03	GO:0070013	intracellular organelle lumen	**x**		x	x
8.20E-03	GO:0043233	organelle lumen	**x**		x	x
1.36E-02	GO:0031974	membrane-enclosed lumen	**x**		x	x
2.45E-02	GO:0043231	intracellular membrane-bounded organelle	**x**	x	x	x
**MFs**						
**p-value**	**term ID**	**term name**	**Distribution of genes of interest (and PPIs) within terms**			
			MAPT	*MARK2*	*EP300*	*AKT1*
2.93E-04	GO:0005515	protein binding	**x**	x	x	x
1.42E-02	GO:0008134	transcription factor binding			x	
1.63E-02	GO:0003676	nucleic acid binding		x	x	
1.95E-02	GO:0003712	transcription cofactor activity			x	
2.98E-02	GO:0044822	poly(A) RNA binding		x		
4.48E-02	GO:0000989	transcription factor binding transcription factor activity			x	
4.74E-02	GO:0000988	protein binding transcription factor activity			x	

ST 7. Functional annotation for BPs, CCs and MFs for the cyan module in TCTX. Summary of functional annotation analysis for the cyan module in TCTX. All significant terms are shown (p<0.05) and those with p<10-4 are highlighted in grey. CCs= cellular compartments (only data available). The terms where our genes of interest are included are indicated with a 'x'. In addition, the protein-protein interactors (PPIs; see **Table IV**) of our genes of interest and their distribution within the terms are also indicated.

CCs					
**p-value**	**term ID**	**term name**	**Distribution of genes of interest (and PPIs) within terms**		
			GRN	*TLE3*	*CRKL*
5.64E-03	GO:0044424	intracellular part	**x**	x	x
6.85E-03	GO:0005622	intracellular	**x**	x	x
7.56E-03	GO:0043231	intracellular membrane-bounded organelle	**x**	x	x
3.27E-02	GO:0043227	membrane-bounded organelle	**x**	x	x
4.80E-02	GO:0043229	intracellular organelle	**x**	x	x

ST 8. Functional annotation for BPs, CCs and MFs for the green module in TCTX. Summary of functional annotation analysis for the green module in TCTX. All significant terms are shown (p<0.05) and those with p<10-4 are highlighted in grey. BPs=biological processes; CCs= cellular compartments; MFs=molecular functions. The terms where our genes of interest are included are indicated with a 'x'. In addition, the protein-protein interactors (PPIs; see **Table IV**) of our genes of interest and their distribution within the terms are also indicated.

BPs					
**p-value**	**term ID**	**term name**	**Distribution of genes of interest (and PPIs) within terms**		
			CHMP2B	*C7orf30*	*VHL*
8.91E-08	GO:0046907	intracellular transport	**x**		
9.04E-08	GO:0045184	establishment of protein localization	**x**		
1.76E-07	GO:0015031	protein transport	**x**		
6.90E-07	GO:1902582	single-organism intracellular transport	**x**		
1.31E-06	GO:0016482	cytoplasmic transport			
1.59E-05	GO:0006996	organelle organization	**x**		
1.97E-05	GO:0008104	protein localization	**x**		
2.34E-05	GO:0051649	establishment of localization in cell	**x**		
3.24E-05	GO:0007005	mitochondrion organization			
4.21E-05	GO:0006886	intracellular protein transport			
8.64E-05	GO:0044267	cellular protein metabolic process			x
1.26E-04	GO:0033036	macromolecule localization	**x**		
2.23E-04	GO:0051641	cellular localization	**x**		
4.60E-04	GO:0072594	establishment of protein localization to organelle			
4.90E-04	GO:0042254	ribosome biogenesis			
5.87E-04	GO:0006396	RNA processing			
6.70E-04	GO:0034613	cellular protein localization			
6.86E-04	GO:0071702	organic substance transport	**x**		
1.01E-03	GO:0070727	cellular macromolecule localization			
1.35E-03	GO:0034660	ncRNA metabolic process			
4.03E-03	GO:0022613	ribonucleoprotein complex biogenesis			
4.23E-03	GO:1902580	single-organism cellular localization			
4.77E-03	GO:0034470	ncRNA processing			
6.57E-03	GO:0033365	protein localization to organelle			
8.27E-03	GO:0016072	rRNA metabolic process			
1.18E-02	GO:0006364	rRNA processing			
1.30E-02	GO:0043412	macromolecule modification			x
2.07E-02	GO:0070647	protein modification by small protein conjugation or removal			x
2.15E-02	GO:0000209	protein polyubiquitination			
2.26E-02	GO:0016339	calcium-dependent cell-cell adhesion via plasma membrane cell adhesion molecules			
2.63E-02	GO:0051168	nuclear export			
2.86E-02	GO:0006605	protein targeting			
3.02E-02	GO:0044265	cellular macromolecule catabolic process			
3.09E-02	GO:0044260	cellular macromolecule metabolic process			x

3.26E-02	GO:0032446	protein modification by small protein conjugation			x
3.82E-02	GO:0036211	protein modification process			x
3.82E-02	GO:0006464	cellular protein modification process			x
**CC**					
**p-value**	**term ID**	**term name**	**Distribution of genes of interest (and PPIs) within terms**		
			CHMP2B	*C7orf30*	*VHL*
3.14E-13	GO:0044446	intracellular organelle part	**x**	x	x
7.04E-13	GO:0044422	organelle part	**x**		x
7.43E-11	GO:0005622	intracellular	**x**		x
1.73E-10	GO:0044424	intracellular part	**x**		x
6.88E-10	GO:0043231	intracellular membrane-bounded organelle	**x**		x
8.94E-09	GO:0070013	intracellular organelle lumen	**x**		x
1.10E-08	GO:0043227	membrane-bounded organelle	**x**		x
1.23E-08	GO:0043229	intracellular organelle	**x**		x
4.34E-08	GO:0031974	membrane-enclosed lumen	**x**		x
6.23E-08	GO:0043233	organelle lumen	**x**		x
8.27E-08	GO:0043226	organelle	**x**		x
1.66E-07	GO:0044428	nuclear part	**x**		x
3.34E-07	GO:0005737	cytoplasm	**x**		x
4.30E-07	GO:0031981	nuclear lumen	**x**		x
2.04E-05	GO:0044444	cytoplasmic part	**x**		x
1.98E-04	GO:0031090	organelle membrane	**x**		
7.01E-04	GO:1902494	catalytic complex			
7.66E-04	GO:0032991	macromolecular complex	**x**		
1.01E-03	GO:0005654	nucleoplasm	**x**		x
1.31E-03	GO:0044464	cell part	**x**		x
1.31E-03	GO:0005623	cell	**x**		x
1.46E-03	GO:1990234	transferase complex			
2.23E-03	GO:0005730	nucleolus			
3.83E-03	GO:0005739	mitochondrion	**x**		x
6.58E-03	GO:0005634	nucleus	**x**		x
1.60E-02	GO:0030529	ribonucleoprotein complex			
2.37E-02	GO:0043232	intracellular non-membrane-bounded organelle			
2.37E-02	GO:0043228	non-membrane-bounded organelle			x
4.95E-02	GO:0043234	protein complex	**x**		
**MF**					

p-value	term ID	term name	Distribution of genes of interest (and PPIs) within terms		
			CHMP2B	*C7orf30*	*VHL*
3.27E-09	GO:0044822	poly(A) RNA binding			
3.68E-07	GO:0003723	RNA binding			
2.67E-04	GO:0003824	catalytic activity			x
2.57E-03	GO:0016740	transferase activity			x
4.34E-03	GO:0008565	protein transporter activity			
3.09E-02	GO:0019787	small conjugating protein transferase activity			x
4.61E-02	GO:0000166	nucleotide binding			
4.61E-02	GO:1901265	nucleoside phosphate binding			

ST 9. Functional annotation for BPs, CCs and MFs for the lightcyan module in TCTX. Summary of functional annotation analysis for the lightcyan module in TCTX. All significant terms are shown (p<0.05) and those with p<10-4 are highlighted in grey. BPs=biological processes; CCs= cellular compartments; MFs=molecular functions.

BPs		
**p-value**	**term ID**	**term name**
1.35E-38	GO:0006955	immune response
1.89E-37	GO:0002376	immune system process
1.51E-32	GO:0006952	defense response
2.91E-31	GO:0002682	regulation of immune system process
3.45E-30	GO:0002684	positive regulation of immune system process
6.11E-30	GO:0050778	positive regulation of immune response
1.22E-29	GO:0050776	regulation of immune response
5.97E-26	GO:0002253	activation of immune response
1.14E-23	GO:0045087	innate immune response
6.66E-23	GO:0002757	immune response-activating signal transduction
1.12E-21	GO:0045321	leukocyte activation
8.48E-20	GO:0002252	immune effector process
1.52E-19	GO:0001775	cell activation
9.40E-19	GO:0002764	immune response-regulating signaling pathway
1.48E-15	GO:0048584	positive regulation of response to stimulus
1.50E-15	GO:0002694	regulation of leukocyte activation
1.67E-15	GO:0001817	regulation of cytokine production
1.88E-15	GO:0050865	regulation of cell activation
2.06E-15	GO:0001816	cytokine production
3.86E-15	GO:0050867	positive regulation of cell activation
3.91E-15	GO:0006950	response to stress
4.97E-15	GO:0046649	lymphocyte activation
1.31E-14	GO:0002696	positive regulation of leukocyte activation
2.84E-14	GO:0009607	response to biotic stimulus
2.91E-14	GO:0043207	response to external biotic stimulus
2.91E-14	GO:0051707	response to other organism
2.67E-13	GO:0006954	inflammatory response
2.95E-13	GO:0031347	regulation of defense response
3.60E-13	GO:0048583	regulation of response to stimulus
7.74E-13	GO:0048518	positive regulation of biological process
1.14E-12	GO:0051249	regulation of lymphocyte activation
1.76E-12	GO:0001819	positive regulation of cytokine production
2.16E-12	GO:0046651	lymphocyte proliferation
2.46E-12	GO:0032943	mononuclear cell proliferation
4.65E-12	GO:0002429	immune response-activating cell surface receptor signaling pathway
7.63E-12	GO:0070661	leukocyte proliferation

1.57E-11	GO:0051251	positive regulation of lymphocyte activation
6.38E-11	GO:0050896	response to stimulus
7.48E-11	GO:0002274	myeloid leukocyte activation
8.20E-10	GO:0002697	regulation of immune effector process
1.08E-09	GO:0002250	adaptive immune response
1.09E-09	GO:0050670	regulation of lymphocyte proliferation
1.25E-09	GO:0032944	regulation of mononuclear cell proliferation
1.33E-09	GO:0034341	response to interferon-gamma
1.38E-09	GO:0031349	positive regulation of defense response
1.84E-09	GO:0030097	hemopoiesis
2.20E-09	GO:0045088	regulation of innate immune response
2.38E-09	GO:0034109	homotypic cell-cell adhesion
2.43E-09	GO:0070663	regulation of leukocyte proliferation
2.61E-09	GO:0098542	defense response to other organism
2.74E-09	GO:0009605	response to external stimulus
2.98E-09	GO:0070489	T cell aggregation
2.98E-09	GO:0042110	T cell activation
3.21E-09	GO:0071593	lymphocyte aggregation
3.61E-09	GO:0007159	leukocyte cell-cell adhesion
4.07E-09	GO:0050851	antigen receptor-mediated signaling pathway
4.32E-09	GO:0070486	leukocyte aggregation
5.97E-09	GO:0002520	immune system development
6.46E-09	GO:0051240	positive regulation of multicellular organismal process
6.98E-09	GO:0048534	hematopoietic or lymphoid organ development
8.18E-09	GO:0002768	immune response-regulating cell surface receptor signaling pathway
1.72E-08	GO:0002699	positive regulation of immune effector process
1.76E-08	GO:0050900	leukocyte migration
1.78E-08	GO:0002521	leukocyte differentiation
1.86E-08	GO:0019724	B cell mediated immunity
1.99E-08	GO:0007165	signal transduction
2.22E-08	GO:0009617	response to bacterium
2.33E-08	GO:0034097	response to cytokine
4.03E-08	GO:0002221	pattern recognition receptor signaling pathway
5.10E-08	GO:0002758	innate immune response-activating signal transduction
5.10E-08	GO:0050870	positive regulation of T cell activation
5.21E-08	GO:0002443	leukocyte mediated immunity
5.72E-08	GO:1903039	positive regulation of leukocyte cell-cell adhesion
6.26E-08	GO:0051716	cellular response to stimulus
6.42E-08	GO:0034112	positive regulation of homotypic cell-cell adhesion
7.19E-08	GO:0002460	adaptive immune response based on somatic recombination of immune receptors built from immunoglobulin superfamily domains

8.25E-08	GO:0016337	single organismal cell-cell adhesion
9.00E-08	GO:0002218	activation of innate immune response
1.01E-07	GO:0071345	cellular response to cytokine stimulus
1.15E-07	GO:0007155	cell adhesion
1.22E-07	GO:0019221	cytokine-mediated signaling pathway
1.27E-07	GO:0022610	biological adhesion
1.34E-07	GO:0060333	interferon-gamma-mediated signaling pathway
1.77E-07	GO:0050863	regulation of T cell activation
1.96E-07	GO:0016064	immunoglobulin mediated immune response
2.13E-07	GO:0045089	positive regulation of innate immune response
2.53E-07	GO:1903037	regulation of leukocyte cell-cell adhesion
2.63E-07	GO:0042113	B cell activation
2.73E-07	GO:0042098	T cell proliferation
3.07E-07	GO:0071346	cellular response to interferon-gamma
4.45E-07	GO:0080134	regulation of response to stress
4.48E-07	GO:0098602	single organism cell adhesion
4.64E-07	GO:0034110	regulation of homotypic cell-cell adhesion
5.39E-07	GO:0045621	positive regulation of lymphocyte differentiation
5.48E-07	GO:0044700	single organism signaling
5.65E-07	GO:0002366	leukocyte activation involved in immune response
5.65E-07	GO:0002263	cell activation involved in immune response
5.91E-07	GO:0023052	signaling
6.90E-07	GO:0050853	B cell receptor signaling pathway
7.12E-07	GO:0022409	positive regulation of cell-cell adhesion
1.15E-06	GO:0030155	regulation of cell adhesion
1.24E-06	GO:1903706	regulation of hemopoiesis
1.28E-06	GO:0051239	regulation of multicellular organismal process
1.29E-06	GO:0050864	regulation of B cell activation
1.57E-06	GO:0007154	cell communication
1.89E-06	GO:0045785	positive regulation of cell adhesion
3.34E-06	GO:0002224	toll-like receptor signaling pathway
3.69E-06	GO:0050671	positive regulation of lymphocyte proliferation
3.90E-06	GO:0009611	response to wounding
4.17E-06	GO:1902105	regulation of leukocyte differentiation
4.18E-06	GO:0071216	cellular response to biotic stimulus
4.25E-06	GO:0032946	positive regulation of mononuclear cell proliferation
5.00E-06	GO:0032101	regulation of response to external stimulus
5.12E-06	GO:0002237	response to molecule of bacterial origin
6.43E-06	GO:0070665	positive regulation of leukocyte proliferation
6.66E-06	GO:0002275	myeloid cell activation involved in immune response
8.67E-06	GO:0030098	lymphocyte differentiation
9.59E-06	GO:1902107	positive regulation of leukocyte differentiation

9.71E-06	GO:0002683	negative regulation of immune system process
1.01E-05	GO:0050707	regulation of cytokine secretion
1.39E-05	GO:0006909	phagocytosis
1.41E-05	GO:0045619	regulation of lymphocyte differentiation
1.66E-05	GO:0032496	response to lipopolysaccharide
1.73E-05	GO:1903708	positive regulation of hemopoiesis
1.98E-05	GO:0002449	lymphocyte mediated immunity
2.61E-05	GO:0022407	regulation of cell-cell adhesion
3.12E-05	GO:0007166	cell surface receptor signaling pathway
3.55E-05	GO:0006959	humoral immune response
3.62E-05	GO:0032103	positive regulation of response to external stimulus
3.90E-05	GO:0048522	positive regulation of cellular process
4.05E-05	GO:0097529	myeloid leukocyte migration
5.30E-05	GO:0070887	cellular response to chemical stimulus
5.77E-05	GO:0071219	cellular response to molecule of bacterial origin
7.59E-05	GO:0050663	cytokine secretion
9.23E-05	GO:0030593	neutrophil chemotaxis
9.53E-05	GO:0097530	granulocyte migration
1.22E-04	GO:0050852	T cell receptor signaling pathway
1.60E-04	GO:0045577	regulation of B cell differentiation
1.65E-04	GO:0050871	positive regulation of B cell activation
1.65E-04	GO:1990266	neutrophil migration
1.71E-04	GO:0030183	B cell differentiation
1.84E-04	GO:0051704	multi-organism process
1.86E-04	GO:0042116	macrophage activation
2.69E-04	GO:0016477	cell migration
2.84E-04	GO:0045582	positive regulation of T cell differentiation
3.64E-04	GO:0030595	leukocyte chemotaxis
4.14E-04	GO:0040011	locomotion
5.19E-04	GO:0002532	production of molecular mediator involved in inflammatory response
6.47E-04	GO:0071621	granulocyte chemotaxis
6.47E-04	GO:0042100	B cell proliferation
6.72E-04	GO:0030099	myeloid cell differentiation
7.11E-04	GO:0042129	regulation of T cell proliferation
7.20E-04	GO:0002755	MyD88-dependent toll-like receptor signaling pathway
8.79E-04	GO:0042102	positive regulation of T cell proliferation
1.06E-03	GO:0009615	response to virus
1.26E-03	GO:0048870	cell motility
1.26E-03	GO:0051674	localization of cell
1.40E-03	GO:0046634	regulation of alpha-beta T cell activation
1.50E-03	GO:0002495	antigen processing and presentation of peptide antigen via MHC class II
1.67E-03	GO:0030888	regulation of B cell proliferation

1.69E-03	GO:0002504	antigen processing and presentation of peptide or polysaccharide antigen via MHC class II
1.73E-03	GO:0002888	positive regulation of myeloid leukocyte mediated immunity
1.73E-03	GO:0033005	positive regulation of mast cell activation
1.74E-03	GO:0060326	cell chemotaxis
1.97E-03	GO:0002444	myeloid leukocyte mediated immunity
2.00E-03	GO:0071222	cellular response to lipopolysaccharide
2.73E-03	GO:0050715	positive regulation of cytokine secretion
2.77E-03	GO:0045579	positive regulation of B cell differentiation
3.01E-03	GO:0002886	regulation of myeloid leukocyte mediated immunity
3.17E-03	GO:0051607	defense response to virus
3.28E-03	GO:0046635	positive regulation of alpha-beta T cell activation
3.40E-03	GO:0065007	biological regulation
3.83E-03	GO:0042221	response to chemical
4.22E-03	GO:0048002	antigen processing and presentation of peptide antigen
4.32E-03	GO:0031295	T cell costimulation
4.32E-03	GO:0031294	lymphocyte costimulation
4.86E-03	GO:0032501	multicellular organismal process
5.14E-03	GO:0033003	regulation of mast cell activation
5.46E-03	GO:0002703	regulation of leukocyte mediated immunity
5.63E-03	GO:0035556	intracellular signal transduction
5.81E-03	GO:0045576	mast cell activation
5.97E-03	GO:0010033	response to organic substance
6.03E-03	GO:0042742	defense response to bacterium
6.11E-03	GO:0050727	regulation of inflammatory response
6.59E-03	GO:0002712	regulation of B cell mediated immunity
6.64E-03	GO:0002695	negative regulation of leukocyte activation
6.94E-03	GO:0042108	positive regulation of cytokine biosynthetic process
7.03E-03	GO:0002478	antigen processing and presentation of exogenous peptide antigen
7.03E-03	GO:0042089	cytokine biosynthetic process
8.72E-03	GO:0002700	regulation of production of molecular mediator of immune response
8.79E-03	GO:0045580	regulation of T cell differentiation
8.79E-03	GO:0042107	cytokine metabolic process
9.62E-03	GO:0002399	MHC class II protein complex assembly
9.62E-03	GO:0002238	response to molecule of fungal origin
9.62E-03	GO:0002503	peptide antigen assembly with MHC class II protein complex
9.70E-03	GO:0044707	single-multicellular organism process
9.73E-03	GO:0050789	regulation of biological process
9.78E-03	GO:0043299	leukocyte degranulation
9.80E-03	GO:0046631	alpha-beta T cell activation
1.01E-02	GO:0019884	antigen processing and presentation of exogenous antigen
1.01E-02	GO:0002283	neutrophil activation involved in immune response
1.01E-02	GO:0001774	microglial cell activation

1.05E-02	GO:0030225	macrophage differentiation
1.09E-02	GO:0019886	antigen processing and presentation of exogenous peptide antigen via MHC class II
1.33E-02	GO:0071310	cellular response to organic substance
1.34E-02	GO:0051250	negative regulation of lymphocyte activation
1.61E-02	GO:0043303	mast cell degranulation
1.61E-02	GO:0002279	mast cell activation involved in immune response
1.61E-02	GO:0002455	humoral immune response mediated by circulating immunoglobulin
1.65E-02	GO:0007229	integrin-mediated signaling pathway
1.73E-02	GO:0042119	neutrophil activation
1.73E-02	GO:0035588	G-protein coupled purinergic receptor signaling pathway
1.85E-02	GO:0031532	actin cytoskeleton reorganization
1.95E-02	GO:0050866	negative regulation of cell activation
2.07E-02	GO:0033993	response to lipid
2.09E-02	GO:0050729	positive regulation of inflammatory response
2.19E-02	GO:0051046	regulation of secretion
2.30E-02	GO:1903034	regulation of response to wounding
2.31E-02	GO:0043304	regulation of mast cell degranulation
2.31E-02	GO:0006911	phagocytosis, engulfment
2.31E-02	GO:0033006	regulation of mast cell activation involved in immune response
2.39E-02	GO:0002448	mast cell mediated immunity
2.39E-02	GO:0045058	T cell selection
2.70E-02	GO:0050794	regulation of cellular process
2.93E-02	GO:0042035	regulation of cytokine biosynthetic process
2.96E-02	GO:0043306	positive regulation of mast cell degranulation
2.96E-02	GO:0035589	G-protein coupled purinergic nucleotide receptor signaling pathway
2.96E-02	GO:0033008	positive regulation of mast cell activation involved in immune response
2.96E-02	GO:0019882	antigen processing and presentation
3.80E-02	GO:0002396	MHC protein complex assembly
3.80E-02	GO:0002501	peptide antigen assembly with MHC protein complex
3.84E-02	GO:0002831	regulation of response to biotic stimulus
3.95E-02	GO:0036230	granulocyte activation
4.12E-02	GO:0002702	positive regulation of production of molecular mediator of immune response
4.59E-02	GO:0042832	defense response to protozoan
4.59E-02	GO:0045059	positive thymic T cell selection
4.88E-02	GO:0032418	lysosome localization
4.88E-02	GO:0032729	positive regulation of interferon-gamma production

CCs

p-value	term ID	term name
1.28E-09	GO:0071944	cell periphery
2.16E-09	GO:0005886	plasma membrane
1.58E-08	GO:0016021	integral component of membrane

2.84E-08	GO:0031224	intrinsic component of membrane
1.99E-06	GO:0044425	membrane part
3.02E-06	GO:0042613	MHC class II protein complex
5.97E-06	GO:0044459	plasma membrane part
7.73E-06	GO:0016020	membrane
5.43E-05	GO:0000323	lytic vacuole
5.43E-05	GO:0005764	lysosome
4.00E-04	GO:0042611	MHC protein complex
6.00E-04	GO:0005765	lysosomal membrane
6.18E-04	GO:0005773	vacuole
8.09E-04	GO:0098552	side of membrane
8.29E-04	GO:0009986	cell surface
1.69E-03	GO:0043235	receptor complex
2.27E-03	GO:0005887	integral component of plasma membrane
3.82E-03	GO:0005774	vacuolar membrane
3.87E-03	GO:0044437	vacuolar part
5.72E-03	GO:0031226	intrinsic component of plasma membrane
7.06E-03	GO:0044440	endosomal part
7.78E-03	GO:0009897	external side of plasma membrane
1.31E-02	GO:0030669	clathrin-coated endocytic vesicle membrane
1.56E-02	GO:0030139	endocytic vesicle
1.56E-02	GO:0010008	endosome membrane
1.83E-02	GO:0031982	vesicle
1.97E-02	GO:0032588	trans-Golgi network membrane
2.15E-02	GO:0031988	membrane-bounded vesicle
2.89E-02	GO:0045334	clathrin-coated endocytic vesicle
3.05E-02	GO:0071556	integral component of lumenal side of endoplasmic reticulum membrane
3.05E-02	GO:0098553	lumenal side of endoplasmic reticulum membrane
3.05E-02	GO:0098576	lumenal side of membrane

MFs

p-value	term ID	term name
4.41E-10	GO:0004872	receptor activity
1.15E-09	GO:0038023	signaling receptor activity
6.86E-09	GO:0060089	molecular transducer activity
6.86E-09	GO:0004871	signal transducer activity

1.60E-07	GO:0004888	transmembrane signaling receptor activity
5.75E-05	GO:0003823	antigen binding
2.22E-03	GO:0019864	IgG binding
6.30E-03	GO:0023023	MHC protein complex binding
2.96E-02	GO:0045028	G-protein coupled purinergic nucleotide receptor activity
2.96E-02	GO:0001608	G-protein coupled nucleotide receptor activity
3.05E-02	GO:0035586	purinergic receptor activity

ST 10. Functional annotation for BPs, CCs and MFs for the darkturquoise module in TCTX. Summary of functional annotation analysis for the darkturquoise module in TCTX. All significant terms are shown (p<0.05) and those with p<10-4 are highlighted in grey. CCs= cellular compartments (only data available). The terms where our gene of interest is included are indicate with a 'x'. No protein-protein interactors (PPIs; see **Table IV**) of TMEM106B distributed within the terms.

CCs			
**p-value**	**term ID**	**term name**	**Distribution of genes of interest within terms**
			TMEM106B
1.96E-06	GO:0044424	intracellular part	x
4.21E-06	GO:0044446	intracellular organelle part	x
4.75E-06	GO:0005622	intracellular	x
5.56E-06	GO:0043229	intracellular organelle	x
9.32E-06	GO:0043231	intracellular membrane-bounded organelle	x
2.17E-05	GO:0044422	organelle part	x
2.80E-05	GO:0005737	cytoplasm	x
4.33E-05	GO:0005654	nucleoplasm	
1.14E-04	GO:0043226	organelle	x
2.04E-04	GO:0043227	membrane-bounded organelle	x
2.09E-04	GO:0044428	nuclear part	
2.49E-04	GO:0005634	nucleus	
9.73E-04	GO:0031981	nuclear lumen	
8.52E-03	GO:0070013	intracellular organelle lumen	
1.34E-02	GO:0043233	organelle lumen	
2.09E-02	GO:0031974	membrane-enclosed lumen	
2.84E-02	GO:0044464	cell part	x
2.84E-02	GO:0005623	cell	x

ST 11. Functional annotation for BPs, CCs and MFs for the purple module in FCTX. Summary of functional annotation analysis for the purple module in FCTX. All significant terms are shown (p<0.05) and those with p<10-4 are highlighted in grey. BPs=biological processes; CCs= cellular compartments; MFs=molecular functions. The terms where our genes of interest and their PPIs (identified by the same colour) are included are indicated with a 'x'.

BPs																										
p-value	term ID	term name	Distribution of genes of interest (and PPIs) within terms																							
			C9orf72	*VCP*	*OPTN*	*UBQLN2*	*APP*	*ELAVL1*	*EIF2B2*	*CUL2*	*UBQLN1*	*NF1*	*NIPSNAP1*	*BTRC*	*ARFGEF2*	*COPS3*	*PLAA*	*CLTA*	*ANXA7*	*UBQLN1*	*SEC23A*	*USP9X*	*STAM*	*HSPA13*	*RAB11A*	*RTN3*
3.90E-07	GO:0043632	modification-dependent macromolecule catabolic process		x						x		x		x		x						x				
1.08E-06	GO:0006511	ubiquitin-dependent protein catabolic process		x						x		x		x		x						x				
1.12E-06	GO:0019941	modification-dependent protein catabolic process		x						x		x		x		x						x				
1.46E-06	GO:0044237	cellular metabolic process	x	x	x		x	x		x	x	x		x	x	x	x		x	x	x	x				
1.72E-06	GO:0051603	proteolysis involved in cellular protein catabolic process		x						x		x		x		x						x				
4.88E-06	GO:0044257	cellular protein catabolic process		x						x		x		x		x						x				
1.95E-05	GO:0044265	cellular macromolecule catabolic process		x						x		x		x		x						x				
7.23E-05	GO:0071704	organic substance metabolic process		x	x		x	x		x	x	x		x	x	x	x		x	x	x	x	x			
1.01E-04	GO:0008152	metabolic process	x	x	x		x	x		x	x	x		x	x	x	x		x	x	x	x	x		x	
1.71E-04	GO:0030163	protein catabolic process		x						x		x		x		x						x				
3.29E-04	GO:0009161	ribonucleoside monophosphate metabolic process																								
3.89E-04	GO:0009167	purine ribonucleoside monophosphate metabolic process																								
4.72E-04	GO:0009126	purine nucleoside monophosphate metabolic process																								
5.84E-04	GO:0044238	primary metabolic process		x			x	x		x	x	x		x		x	x			x	x	x	x			
7.21E-04	GO:0008150	biological_process	x	x	x		x	x		x	x	x	x	x	x	x	x	x	x	x	x	x	x		x	x
9.04E-04	GO:0046907	intracellular transport		x	x		x					x			x			x			x		x		x	
1.14E-03	GO:0045333	cellular respiration																								
1.19E-03	GO:0009057	macromolecule catabolic process		x						x		x		x		x						x				
1.19E-03	GO:0009144	purine nucleoside triphosphate metabolic process																								
1.35E-03	GO:0009123	nucleoside monophosphate metabolic process																								
1.69E-03	GO:0043161	proteasome-mediated ubiquitin-dependent protein catabolic process		x								x		x								x				
1.77E-03	GO:0046034	ATP metabolic process																								
2.21E-03	GO:0044248	cellular catabolic process	x	x	x					x		x		x		x			x			x				
3.01E-03	GO:0009205	purine ribonucleoside triphosphate metabolic process																								
4.61E-03	GO:0007005	mitochondrion organization																								
4.88E-03	GO:0048002	antigen processing and presentation of peptide antigen															x			x						
5.32E-03	GO:0009141	nucleoside triphosphate metabolic process																								
5.54E-03	GO:0031329	regulation of cellular catabolic process		x										x								x				
5.61E-03	GO:0006412	translation					x	x																		

6.74E-03	GO:0032543	mitochondrial translation																								
6.90E-03	GO:0030433	ER-associated ubiquitin-dependent protein catabolic process		x																						
7.43E-03	GO:0044267	cellular protein metabolic process		x			x	x		x	x	x		x		x				x	x	x				
7.50E-03	GO:1902582	single-organism intracellular transport		x	x		x					x			x			x			x		x		x	
7.57E-03	GO:0009199	ribonucleoside triphosphate metabolic process																								
8.63E-03	GO:0006119	oxidative phosphorylation																								
8.84E-03	GO:0016197	endosomal transport										x											x		x	
9.04E-03	GO:0010498	proteasomal protein catabolic process		x								x		x								x				
9.20E-03	GO:0042278	purine nucleoside metabolic process																								
9.45E-03	GO:1903047	mitotic cell cycle process		x	x		x			x				x								x			x	
1.06E-02	GO:0070647	protein modification by small protein conjugation or removal		x						x	x	x		x		x				x		x				
1.43E-02	GO:0009894	regulation of catabolic process		x										x								x				
1.84E-02	GO:0046128	purine ribonucleoside metabolic process																								
2.17E-02	GO:0002478	antigen processing and presentation of exogenous peptide antigen																x			x					
2.35E-02	GO:0044260	cellular macromolecule metabolic process		x	x		x	x		x	x	x		x	x	x				x	x	x				
2.81E-02	GO:0022904	respiratory electron transport chain																								
4.03E-02	GO:0022900	electron transport chain																								
4.26E-02	GO:0042773	ATP synthesis coupled electron transport																								
4.26E-02	GO:0042775	mitochondrial ATP synthesis coupled electron transport																								
4.38E-02	GO:0000278	mitotic cell cycle		x	x		x			x				x								x			x	
4.44E-02	GO:0019884	antigen processing and presentation of exogenous antigen																x			x					
4.48E-02	GO:0006996	organelle organization		x	x							x			x						x	x	x		x	x
5.00E-02	GO:0009987	cellular process	x	x	x		x	x		x	x	x		x	x	x	x	x	x	x	x	x	x		x	x

CCs

p-value	term ID	term name	Distribution of genes of interest (and PPIs) within terms																							
			C9orf72	*VCP*	*OPTN*	*UBQLN2*	*APP*	*ELAVL1*	*EIF2B2*	*CUL2*	*UBQLN1*	*NF1*	*NIPSNAP1*	*BTRC*	*ARFGEF2*	*COPS3*	*PLAA*	*CLTA*	*ANXA7*	*UBQLN1*	*SEC23A*	*USP9X*	*STAM*	*HSPA13*	*RAB11A*	*RTN3*
3.48E-17	GO:0005622	intracellular	x	x	x	x	x	x	x	x	x	x	x	x	x	x		x	x	x	x	x	x	x	x	x
7.14E-17	GO:0044424	intracellular part	x	x	x	x	x	x	x	x	x	x	x	x	x	x		x	x	x	x	x	x	x	x	x
2.41E-15	GO:0043231	intracellular membrane-bounded organelle	x	x	x	x	x	x		x	x	x	x	x	x	x		x	x	x	x		x	x	x	x
2.95E-14	GO:0005737	cytoplasm	x	x	x	x	x	x	x	x	x	x	x	x	x	x		x	x	x	x	x	x	x	x	x
3.10E-13	GO:0043227	membrane-bounded organelle	x	x	x	x	x	x		x	x	x	x	x	x	x	x	x	x	x	x		x	x	x	x
5.85E-13	GO:0043229	intracellular organelle	x	x	x	x	x	x		x	x	x	x	x	x	x		x	x	x	x		x	x	x	x
2.40E-12	GO:0005739	mitochondrion										x	x												x	
4.52E-11	GO:0043226	organelle	x	x	x	x	x	x		x	x	x	x	x	x	x	x	x	x	x	x		x	x	x	x
1.38E-10	GO:0044446	intracellular organelle part	x	x	x		x	x		x	x	x	x	x	x	x		x	x	x	x		x		x	x
1.76E-10	GO:0044444	cytoplasmic part	x	x	x		x	x	x	x	x	x	x	x	x			x	x	x	x	x	x	x	x	x

9.05E-10	GO:0044429	mitochondrial part										x	x													
1.14E-09	GO:0044422	organelle part	x	x	x		x	x		x	x	x	x	x	x	x		x	x	x	x		x		x	x
1.56E-09	GO:0031090	organelle membrane		x	x		x					x	x		x			x	x		x		x		x	x
3.46E-07	GO:0005623	cell	x	x	x	x	x	x	x	x	x	x	x	x	x	x		x	x	x	x	x	x	x	x	x
3.46E-07	GO:0044464	cell part	x	x	x	x	x	x	x	x	x	x	x	x	x	x		x	x	x	x	x	x	x	x	x
7.61E-07	GO:0031967	organelle envelope					x					x	x						x							
1.04E-06	GO:0031975	envelope					x					x	x						x							
2.73E-06	GO:0031966	mitochondrial membrane										x	x													
5.94E-06	GO:0005740	mitochondrial envelope										x	x													
9.87E-05	GO:0005743	mitochondrial inner membrane											x													
2.81E-04	GO:0000502	proteasome complex		x							x									x						
4.86E-04	GO:0005829	cytosol		x	x		x	x	x	x				x	x			x	x		x	x	x		x	
5.04E-04	GO:0032991	macromolecular complex		x			x		x	x	x			x	x	x		x		x	x				x	
5.08E-04	GO:0044455	mitochondrial membrane part																								
5.12E-04	GO:0019866	organelle inner membrane											x													
9.39E-04	GO:0005759	mitochondrial matrix																								
2.44E-03	GO:0005634	nucleus	x	x	x	x	x	x		x	x	x		x		x			x	x						
5.22E-03	GO:0043234	protein complex		x			x		x	x	x			x	x	x		x		x	x				x	
1.90E-02	GO:0098588	bounding membrane of organelle		x	x							x			x			x	x		x		x		x	x
2.52E-02	GO:0031974	membrane-enclosed lumen	x	x	x		x	x		x	x	x		x		x				x						
3.48E-02	GO:0070013	intracellular organelle lumen	x	x	x		x	x		x	x	x		x		x				x						

MFs

p-value	term ID	term name	Distribution of genes of interest (and PPIs) within terms																							
			C9orf72	*VCP*	*OPTN*	*UBQLN2*	*APP*	*ELAVL1*	*EIF2B2*	*CUL2*	*UBQLN1*	*NF1*	*NIPSNAP1*	*BTRC*	*ARFGEF2*	*COPS3*	*PLAA*	*CLTA*	*ANXA7*	*UBQLN1*	*SEC23A*	*USP9X*	*STAM*	*HSPA13*	*RAB11A*	*RTN3*
3.55E-04	GO:0003824	catalytic activity		x			x					x		x								x	x		x	
1.80E-02	GO:0051219	phosphoprotein binding												x												
2.81E-02	GO:0016787	hydrolase activity		x			x															x	x		x	
4.93E-02	GO:0003723	RNA binding		x				x											x							

ST 12. Functional annotation for BPs, CCs and MFs for the lightcyan module in FCTX. Summary of functional annotation analysis for the lightcyan module in FCTX. All significant terms are shown (p<0.05) and those with p<10-4 are highlighted in grey. BPs=biological processes; CCs= cellular compartments; MFs=molecular functions. The terms where our gene of interest is included are indicated with a 'x'.

BPs			
**p-value**	**term ID**	**term name**	**Distribution of genes of interest within terms**
			***FUS* (ID3656904)**
5.69E-09	GO:0090304	nucleic acid metabolic process	x
1.34E-08	GO:0006139	nucleobase-containing compound metabolic process	x
3.64E-08	GO:0046483	heterocycle metabolic process	x
4.39E-08	GO:0006725	cellular aromatic compound metabolic process	x
9.49E-08	GO:0016070	RNA metabolic process	x
1.00E-07	GO:0034641	cellular nitrogen compound metabolic process	x
1.74E-07	GO:1901360	organic cyclic compound metabolic process	x
5.61E-07	GO:0006396	RNA processing	x
8.38E-07	GO:0010467	gene expression	x
4.52E-06	GO:0006807	nitrogen compound metabolic process	x
2.10E-05	GO:0006397	mRNA processing	x
8.46E-05	GO:0016071	mRNA metabolic process	x
2.58E-03	GO:0008380	RNA splicing	x
8.76E-03	GO:0044260	cellular macromolecule metabolic process	x
2.67E-02	GO:0010468	regulation of gene expression	/
2.76E-02	GO:0051252	regulation of RNA metabolic process	/
3.17E-02	GO:0006281	DNA repair	/
4.34E-02	GO:0019219	regulation of nucleobase-containing compound metabolic process	/

CCs			
**p-value**	**term ID**	**term name**	**Distribution of genes of interest within terms**
			***FUS* (ID3656904)**
8.65E-07	GO:0005634	nucleus	x
1.10E-02	GO:0031981	nuclear lumen	x
2.19E-02	GO:0044428	nuclear part	x

MFs			
**p-value**	**term ID**	**term name**	**Distribution of genes of interest within terms**
			***FUS* (ID3656904)**
5.63E-05	GO:0003676	nucleic acid binding	x
8.60E-05	GO:1901363	heterocyclic compound binding	x
1.67E-04	GO:0097159	organic cyclic compound binding	x
8.31E-03	GO:0003677	DNA binding	x

ST 13. Functional annotation for BPs, CCs and MFs for the turquoise module in FCTX. Summary of functional annotation analysis for the turquoise module in FCTX. All significant terms are shown (p<0.05) and those with p<10-4 are highlighted in grey. BPs=biological processes; CCs= cellular compartments; MFs=molecular functions. TDP-43 did not distribute within any of the GO terms.

BPs			
**p-value**	**term ID**	**term name**	**Distribution of genes of interest within terms**
			TDP-43
4.76E-08	GO:0007606	sensory perception of chemical stimulus	
2.47E-07	GO:0007600	sensory perception	
5.25E-06	GO:0050907	detection of chemical stimulus involved in sensory perception	
6.48E-06	GO:0050911	detection of chemical stimulus involved in sensory perception of smell	
7.70E-06	GO:0051606	detection of stimulus	
1.16E-05	GO:0007608	sensory perception of smell	
1.48E-04	GO:0009593	detection of chemical stimulus	
3.70E-04	GO:0050906	detection of stimulus involved in sensory perception	
1.02E-03	GO:0031424	keratinization	
1.40E-03	GO:0043588	skin development	
4.06E-03	GO:0008544	epidermis development	
7.89E-03	GO:0007601	visual perception	
9.32E-03	GO:0035270	endocrine system development	
1.27E-02	GO:0007186	G-protein coupled receptor signaling pathway	
2.11E-02	GO:0050953	sensory perception of light stimulus	
2.11E-02	GO:0042035	regulation of cytokine biosynthetic process	
2.79E-02	GO:0042107	cytokine metabolic process	
CCs			
**p-value**	**term ID**	**term name**	**Distribution of genes of interest within terms**
			TDP-43
1.91E-10	GO:0045095	keratin filament	
8.68E-10	GO:0005615	extracellular space	
2.10E-09	GO:0005882	intermediate filament	
2.46E-07	GO:0005578	proteinaceous extracellular matrix	
9.57E-07	GO:0031012	extracellular matrix	
3.75E-05	GO:0005887	integral component of plasma membrane	
6.10E-05	GO:0045111	intermediate filament cytoskeleton	
6.10E-05	GO:0005581	collagen trimer	
7.11E-05	GO:0031226	intrinsic component of plasma membrane	
4.62E-02	GO:0016324	apical plasma membrane	
4.63E-02	GO:0098590	plasma membrane region	
MFs			

p-value	term ID	term name	Distribution of genes of interest within terms
			TDP-43
4.79E-11	GO:0004930	G-protein coupled receptor activity	
4.89E-10	GO:0004888	transmembrane signaling receptor activity	
1.25E-09	GO:0004872	receptor activity	
2.14E-08	GO:0004252	serine-type endopeptidase activity	
7.57E-08	GO:0038023	signaling receptor activity	
6.48E-06	GO:0004984	olfactory receptor activity	
1.73E-05	GO:0008236	serine-type peptidase activity	
2.54E-05	GO:0017171	serine hydrolase activity	
3.04E-02	GO:0005044	scavenger receptor activity	
3.78E-02	GO:0060089	molecular transducer activity	
3.78E-02	GO:0004871	signal transducer activity	
4.18E-02	GO:0005125	cytokine activity	

ST 14. Functional annotation for BPs, CCs and MFs for the purple in TCTX. Summary of functional annotation analysis for the purple module in TCTX. All significant terms are shown (p<0.05) and those with p<10-4 are highlighted in grey. BPs=biological processes; CCs= cellular compartments; MFs=molecular functions. The terms where our genes of interest and their PPIs (identified by the same colour) are included are indicated with a 'x'.

BPs

p-value	term ID	term name	Distribution of genes of interest (and PPIs) within terms																
			C9orf72	*VCP*	*UBQLN2*	*APP*	*ELAVL1*	*UBQLN1*	*BTRC*	*PSMA1*	*CUL2*	*ARIH1*	*PSMA4*	*NF1*	*UBQLN1*	*USP9X*	*STAM*	*SEC23A*	*HSPA13*
9.03E-09	GO:0044267	cellular protein metabolic process		x		x	x	x	x		x	x	x	x	x	x		x	
1.18E-08	GO:0043632	modification-dependent macromolecule catabolic process		x					x		x	x	x	x		x			
1.81E-08	GO:0051603	proteolysis involved in cellular protein catabolic process		x					x		x	x	x	x		x			
2.98E-08	GO:0044257	cellular protein catabolic process		x					x		x	x	x	x		x			
3.04E-08	GO:0006511	ubiquitin-dependent protein catabolic process		x					x		x	x	x	x		x			
6.35E-08	GO:0019941	modification-dependent protein catabolic process		x					x		x	x	x	x		x			
1.98E-07	GO:0070647	protein modification by small protein conjugation or removal		x				x	x		x	x	x	x	x	x			
1.87E-06	GO:0030163	protein catabolic process		x					x		x	x	x	x		x			
4.22E-06	GO:0044265	cellular macromolecule catabolic process		x					x		x	x	x	x		x			
4.25E-06	GO:0043412	macromolecule modification		x		x		x	x		x	x	x	x	x	x		x	
9.28E-06	GO:0019538	protein metabolic process		x		x	x	x	x		x	x	x	x	x	x		x	
1.83E-05	GO:0036211	protein modification process		x		x		x	x		x	x	x	x	x	x		x	
1.83E-05	GO:0006464	cellular protein modification process		x		x		x	x		x	x	x	x	x	x		x	
8.06E-05	GO:0044237	cellular metabolic process	x	x		x	x	x	x		x	x	x	x	x	x		x	
1.34E-04	GO:0016567	protein ubiquitination		x				x	x		x	x	x	x	x	x			
1.81E-04	GO:0044260	cellular macromolecule metabolic process		x		x	x	x	x		x	x	x	x	x			x	
2.82E-04	GO:0032446	protein modification by small protein conjugation		x				x	x		x	x	x	x	x	x			
7.63E-04	GO:0043161	proteasome-mediated ubiquitin-dependent protein catabolic process		x					x				x	x		x			
1.07E-03	GO:0010498	proteasomal protein catabolic process		x					x				x	x		x			
1.40E-03	GO:0008152	metabolic process	x	x		x	x	x	x		x	x	x	x	x	x		x	
1.59E-03	GO:0008150	biological_process	x	x		x	x	x	x		x	x	x	x	x	x	x	x	
1.61E-03	GO:0009057	macromolecule catabolic process		x					x		x	x	x	x		x			
2.80E-03	GO:0043170	macromolecule metabolic process		x		x	x	x	x		x	x	x	x	x	x		x	
6.58E-03	GO:0071704	organic substance metabolic process		x		x	x	x	x		x	x	x	x	x	x		x	
6.72E-03	GO:0044238	primary metabolic process		x		x	x	x	x		x	x	x	x	x			x	
1.08E-02	GO:0051437	positive regulation of ubiquitin-protein ligase activity involved in regulation of mitotic cell cycle transition							x				x						
1.16E-02	GO:0046907	intracellular transport		x		x								x			x	x	
1.49E-02	GO:0044403	symbiosis, encompassing mutualism through parasitism							x		x		x						
1.49E-02	GO:0044419	interspecies interaction between organisms							x		x		x						

1.51E-02	GO:0016032	viral process							x		x		x						
1.61E-02	GO:0000209	protein polyubiquitination							x				x	x					
2.04E-02	GO:0070727	cellular macromolecule localization		x		x								x			x	x	
2.35E-02	GO:0044764	multi-organism cellular process							x		x		x						
3.02E-02	GO:1901990	regulation of mitotic cell cycle phase transition				x							x						
3.03E-02	GO:0006996	organelle organization												x		x	x	x	
3.25E-02	GO:0033036	macromolecule localization		x		x								x			x	x	
4.21E-02	GO:0006508	proteolysis		x		x			x		x	x	x	x		x			

CCs

p-value	term ID	term name	Distribution of genes of interest (and PPIs) within terms																
			C9orf72	*VCP*	*UBQLN2*	*APP*	*ELAVL1*	*UBQLN1*	*BTRC*	*PSMA1*	*CUL2*	*ARIH1*	*PSMA4*	*NF1*	*UBQLN1*	*USP9X*	*STAM*	*SEC23A*	*HSPA13*
2.18E-17	GO:0044446	intracellular organelle part	x	x		x	x	x	x		x		x	x	x		x	x	
3.41E-17	GO:0043231	intracellular membrane-bounded organelle	x	x	x	x	x	x	x		x		x	x	x		x	x	x
3.35E-16	GO:0044424	intracellular part	x	x	x	x	x	x	x		x	x	x	x	x	x	x	x	x
4.24E-16	GO:0044422	organelle part	x	x		x	x	x	x		x		x	x	x		x	x	
5.45E-16	GO:0005622	intracellular	x	x	x	x	x	x	x		x	x	x	x	x	x	x	x	x
1.58E-14	GO:0043229	intracellular organelle	x	x	x	x	x	x	x		x		x	x	x		x	x	x
4.68E-13	GO:0043227	membrane-bounded organelle	x	x	x	x	x	x	x		x		x	x	x		x	x	x
1.28E-12	GO:0005737	cytoplasm	x	x	x	x	x	x	x		x	x	x	x	x	x	x	x	x
1.54E-11	GO:0043226	organelle	x	x	x	x	x	x	x		x		x	x	x		x	x	x
4.09E-10	GO:0044444	cytoplasmic part	x	x		x	x	x	x		x	x	x	x	x	x	x	x	x
1.02E-09	GO:0031090	organelle membrane		x		x								x			x	x	
1.32E-06	GO:0098588	bounding membrane of organelle		x										x			x	x	
3.91E-06	GO:0070013	intracellular organelle lumen	x	x		x	x	x	x		x		x	x	x				
4.27E-06	GO:0031974	membrane-enclosed lumen	x	x		x	x	x	x		x		x	x	x				
8.72E-06	GO:0043233	organelle lumen	x	x		x	x	x	x		x		x	x	x				
1.16E-05	GO:0000502	proteasome complex		x				x					x		x				
3.73E-05	GO:0044428	nuclear part	x	x		x	x	x	x		x		x	x	x				
7.20E-05	GO:0031981	nuclear lumen	x	x		x	x	x	x		x		x	x	x				
9.12E-05	GO:0005634	nucleus	x	x	x	x	x	x	x		x		x	x	x				
9.48E-05	GO:0005829	cytosol		x		x	x		x		x	x	x			x	x	x	
2.65E-04	GO:0044464	cell part	x	x	x	x	x	x	x		x	x	x	x	x	x	x	x	x
2.65E-04	GO:0005623	cell	x	x	x	x	x	x	x		x	x	x	x	x	x	x	x	x
1.10E-03	GO:0005654	nucleoplasm	x	x		x	x	x	x		x		x		x				
1.11E-03	GO:0012505	endomembrane system	x	x		x		x						x	x		x	x	x
1.18E-03	GO:0042175	nuclear outer membrane-endoplasmic reticulum membrane network		x										x				x	
2.74E-03	GO:0032991	macromolecular complex		x		x		x	x		x	x	x		x			x	
3.81E-03	GO:0031967	organelle envelope				x								x					
4.53E-03	GO:0031975	envelope				x								x					
5.21E-03	GO:0005789	endoplasmic reticulum membrane		x										x				x	

5.42E-03	GO:0005739	mitochondrion												x					
1.25E-02	GO:0044429	mitochondrial part												x					
2.28E-02	GO:0005730	nucleolus									x			x					
2.84E-02	GO:0005798	Golgi-associated vesicle																	
**MFs**																			
**p-value**	**term ID**	**term name**	**Distribution of genes of interest (and PPIs) within terms**																
			C9orf72	*VCP*	*UBQLN2*	*APP*	*ELAVL1*	*UBQLN1*	*BTRC*	*PSMA1*	*CUL2*	*ARIH1*	*PSMA4*	*NF1*	*UBQLN1*	*USP9X*	*STAM*	*SEC23A*	*HSPA13*
1.08E-04	GO:0003824	catalytic activity		x		x			x			x	x	x		x			
3.96E-03	GO:0005488	binding	x	x	x	x	x	x	x		x	x	x	x	x	x	x	x	x
5.43E-03	GO:0003723	RNA binding		x			x												

ST 15. Functional annotation for BPs, CCs and MFs for the mignightblue module in TCTX. Summary of functional annotation analysis for the mignightblue module in TCTX. All significant terms are shown (p<0.05) and those with p<10-4 are highlighted in grey. BPs=biological processes; CCs= cellular compartments; MFs=molecular functions. The terms where our gene of interest is included are indicated with a 'x'.

BPs			
**p-value**	**term ID**	**term name**	**Distribution of genes of interest within terms**
			***FUS* (ID3656904)**
1.11E-12	GO:0090304	nucleic acid metabolic process	x
7.49E-12	GO:0016070	RNA metabolic process	x
1.20E-11	GO:0010467	gene expression	x
1.69E-10	GO:0006139	nucleobase-containing compound metabolic process	x
5.53E-10	GO:0044260	cellular macromolecule metabolic process	x
1.30E-09	GO:0016071	mRNA metabolic process	x
2.03E-09	GO:0046483	heterocycle metabolic process	x
2.54E-09	GO:0006725	cellular aromatic compound metabolic process	x
1.76E-08	GO:1901360	organic cyclic compound metabolic process	x
2.02E-08	GO:0034641	cellular nitrogen compound metabolic process	x
2.32E-08	GO:0043170	macromolecule metabolic process	x
4.15E-08	GO:0006397	mRNA processing	x
6.22E-08	GO:0006807	nitrogen compound metabolic process	x
3.55E-07	GO:0010556	regulation of macromolecule biosynthetic process	
5.54E-07	GO:0044238	primary metabolic process	x
6.21E-07	GO:0031326	regulation of cellular biosynthetic process	
1.04E-06	GO:0019219	regulation of nucleobase-containing compound metabolic process	
1.11E-06	GO:0009889	regulation of biosynthetic process	
1.21E-06	GO:2000112	regulation of cellular macromolecule biosynthetic process	
1.77E-06	GO:0006396	RNA processing	x
1.88E-06	GO:0009059	macromolecule biosynthetic process	
2.73E-06	GO:0051252	regulation of RNA metabolic process	
4.91E-06	GO:0031323	regulation of cellular metabolic process	
5.20E-06	GO:0008152	metabolic process	x
6.61E-06	GO:0080090	regulation of primary metabolic process	
8.16E-06	GO:0044237	cellular metabolic process	x
9.17E-06	GO:0071704	organic substance metabolic process	x
9.20E-06	GO:0019222	regulation of metabolic process	
1.34E-05	GO:0051171	regulation of nitrogen compound metabolic process	
1.44E-05	GO:0010468	regulation of gene expression	
1.62E-05	GO:0034645	cellular macromolecule biosynthetic process	
2.61E-05	GO:0060255	regulation of macromolecule metabolic process	
2.83E-05	GO:0032774	RNA biosynthetic process	
6.09E-05	GO:0044249	cellular biosynthetic process	
1.44E-04	GO:1901576	organic substance biosynthetic process	

1.68E-04	GO:0009058	biosynthetic process	
1.86E-04	GO:0097659	nucleic acid-templated transcription	
1.96E-04	GO:0051276	chromosome organization	
2.29E-04	GO:2001141	regulation of RNA biosynthetic process	
2.81E-04	GO:0022618	ribonucleoprotein complex assembly	
3.13E-04	GO:0044271	cellular nitrogen compound biosynthetic process	
3.16E-04	GO:0006351	transcription, DNA-templated	
4.00E-04	GO:1903506	regulation of nucleic acid-templated transcription	
5.58E-04	GO:0034654	nucleobase-containing compound biosynthetic process	
5.87E-04	GO:0071826	ribonucleoprotein complex subunit organization	
1.24E-03	GO:0018130	heterocycle biosynthetic process	
1.32E-03	GO:0019438	aromatic compound biosynthetic process	
1.33E-03	GO:0006355	regulation of transcription, DNA-templated	
2.17E-03	GO:0022613	ribonucleoprotein complex biogenesis	
2.45E-03	GO:1901362	organic cyclic compound biosynthetic process	
2.69E-03	GO:0031123	RNA 3'-end processing	
3.92E-03	GO:0031124	mRNA 3'-end processing	
4.45E-03	GO:0006325	chromatin organization	
5.04E-03	GO:0008380	RNA splicing	**x**
5.07E-03	GO:0016568	chromatin modification	
5.54E-03	GO:0016570	histone modification	
6.10E-03	GO:0006479	protein methylation	
6.10E-03	GO:0008213	protein alkylation	
6.53E-03	GO:0016571	histone methylation	
6.60E-03	GO:0016569	covalent chromatin modification	
7.52E-03	GO:0006366	transcription from RNA polymerase II promoter	
1.00E-02	GO:0000288	nuclear-transcribed mRNA catabolic process, deadenylation-dependent decay	
1.67E-02	GO:0006357	regulation of transcription from RNA polymerase II promoter	
1.69E-02	GO:0006996	organelle organization	
1.74E-02	GO:1903311	regulation of mRNA metabolic process	
2.50E-02	GO:0018023	peptidyl-lysine trimethylation	
2.88E-02	GO:0000377	RNA splicing, via transesterification reactions with bulged adenosine as nucleophile	**x**
2.88E-02	GO:0000398	mRNA splicing, via spliceosome	**x**
3.37E-02	GO:0000375	RNA splicing, via transesterification reactions	**x**
3.77E-02	GO:0030518	intracellular steroid hormone receptor signaling pathway	
4.24E-02	GO:0044265	cellular macromolecule catabolic process	
4.53E-02	GO:0016574	histone ubiquitination	
4.63E-02	GO:0050684	regulation of mRNA processing	

**CCs**			
**p-value**	**term ID**	**term name**	**Distribution of genes of interest within terms**
			***FUS* (ID3656904)**
6.70E-15	GO:0043231	intracellular membrane-bounded organelle	**x**

1.58E-13	GO:0031981	nuclear lumen	x
3.23E-13	GO:0043229	intracellular organelle	x
9.08E-13	GO:0044428	nuclear part	x
1.34E-12	GO:0070013	intracellular organelle lumen	x
3.68E-12	GO:0005634	nucleus	x
3.74E-12	GO:0043233	organelle lumen	x
4.65E-12	GO:0044451	nucleoplasm part	
1.02E-11	GO:0031974	membrane-enclosed lumen	x
1.41E-11	GO:0043227	membrane-bounded organelle	x
6.95E-11	GO:0043226	organelle	x
2.39E-10	GO:0044424	intracellular part	x
2.43E-10	GO:0005622	intracellular	x
9.95E-10	GO:0005654	nucleoplasm	x
5.10E-09	GO:0044446	intracellular organelle part	x
9.46E-09	GO:0044422	organelle part	x
1.51E-05	GO:0016604	nuclear body	
1.10E-04	GO:0005730	nucleolus	
1.57E-04	GO:0044464	cell part	x
1.57E-04	GO:0005623	cell	x
2.81E-03	GO:0032991	macromolecular complex	
4.97E-03	GO:0035097	histone methyltransferase complex	
1.52E-02	GO:1990234	transferase complex	
1.76E-02	GO:0016607	nuclear speck	
1.84E-02	GO:0030529	ribonucleoprotein complex	
2.39E-02	GO:0034708	methyltransferase complex	
4.50E-02	GO:0043232	intracellular non-membrane-bounded organelle	
4.50E-02	GO:0043228	non-membrane-bounded organelle	
4.55E-02	GO:0005694	chromosome	

**MFs**			
**p-value**	**term ID**	**term name**	**Distribution of genes of interest within terms**
			***FUS* (ID3656904)**
3.08E-12	GO:0003723	RNA binding	x
5.15E-12	GO:0044822	poly(A) RNA binding	x
1.20E-10	GO:0003676	nucleic acid binding	x
5.80E-06	GO:1901363	heterocyclic compound binding	x
6.03E-06	GO:0097159	organic cyclic compound binding	x
2.95E-03	GO:0005488	binding	x
1.16E-02	GO:0000989	transcription factor binding transcription factor activity	
1.25E-02	GO:0000988	protein binding transcription factor activity	
1.49E-02	GO:0003712	transcription cofactor activity	
1.89E-02	GO:0004386	helicase activity	

ST 16. Functional annotation for BPs, CCs and MFs for the magenta module in TCTX. Summary of functional annotation analysis for the magenta module in TCTX. All significant terms are shown (p<0.05) and those with p<10-4 are highlighted in grey. BPs=biological processes; CCs= cellular compartments; MFs=molecular functions. Our genes of interest were not included in any GO term.

BPs				
**p-value**	**term ID**	**term name**	**Distribution of genes of interest within terms**	
			***FUS* (ID3656954)**	***TDP-43***
9.53E-06	GO:0098662	inorganic cation transmembrane transport		
9.55E-06	GO:0034220	ion transmembrane transport		
1.06E-05	GO:0055085	transmembrane transport		
4.68E-05	GO:0098655	cation transmembrane transport		
8.60E-05	GO:0015672	monovalent inorganic cation transport		
3.95E-04	GO:0006812	cation transport		
4.96E-04	GO:0098660	inorganic ion transmembrane transport		
1.09E-03	GO:0007268	synaptic transmission		
2.31E-03	GO:0006811	ion transport		
4.00E-03	GO:0071805	potassium ion transmembrane transport		
4.00E-03	GO:0071804	cellular potassium ion transport		
4.41E-03	GO:0006813	potassium ion transport		
1.16E-02	GO:0007267	cell-cell signaling		
1.39E-02	GO:0043269	regulation of ion transport		
1.40E-02	GO:0030001	metal ion transport		
3.96E-02	GO:0034765	regulation of ion transmembrane transport		

CCs				
**p-value**	**term ID**	**term name**	**Distribution of genes of interest within terms**	
			***FUS* (ID3656954)**	***TDP-43***
2.86E-02	GO:0008076	voltage-gated potassium channel complex		
3.47E-02	GO:0034705	potassium channel complex		

MFs				
**p-value**	**term ID**	**term name**	**Distribution of genes of interest within terms**	
			***FUS* (ID3656954)**	***TDP-43***
1.94E-05	GO:0022890	inorganic cation transmembrane transporter activity		
3.72E-05	GO:0008324	cation transmembrane transporter activity		
5.70E-05	GO:0046873	metal ion transmembrane transporter activity		
2.86E-04	GO:0015075	ion transmembrane transporter activity		
4.69E-04	GO:0022857	transmembrane transporter activity		
1.01E-03	GO:0005261	cation channel activity		
1.11E-03	GO:0015077	monovalent inorganic cation transmembrane transporter activity		
1.39E-03	GO:0022891	substrate-specific transmembrane transporter activity		
2.03E-03	GO:0015079	potassium ion transmembrane transporter activity		

2.28E-03	GO:0022843	voltage-gated cation channel activity		
3.78E-03	GO:0005215	transporter activity		
1.44E-02	GO:0022832	voltage-gated channel activity		
1.44E-02	GO:0005244	voltage-gated ion channel activity		
1.66E-02	GO:0022892	substrate-specific transporter activity		
1.82E-02	GO:0005267	potassium channel activity		
2.24E-02	GO:0005249	voltage-gated potassium channel activity		

ST 17. Summary of pathway terms associated with all modules in FCTX and TCTX.

module	tissue	p-value	term ID	term name
Black	FCTX	4.84E-03	REAC:4839726	Chromatin organization
		4.84E-03	REAC:3247509	Chromatin modifying enzymes
Darkolivegreen		1.21E-02	KEGG:04146	Peroxisome
		1.79E-02	REAC:390918	Peroxisomal lipid metabolism
		2.64E-02	REAC:75896	Plasmalogen biosynthesis
Darkred		7.23E-23	KEGG:05150	Staphylococcus aureus infection
		4.80E-21	REAC:168256	Immune System
		4.52E-17	KEGG:05152	Tuberculosis
		1.64E-13	KEGG:05322	Systemic lupus erythematosus
		1.99E-13	KEGG:05140	Leishmaniasis
		6.69E-12	KEGG:04145	Phagosome
		1.70E-10	REAC:168249	Innate Immune System
		5.08E-10	REAC:1280218	Adaptive Immune System
		1.13E-09	KEGG:04612	Antigen processing and presentation
		2.52E-09	KEGG:05310	Asthma
		4.45E-09	KEGG:05332	Graft-versus-host disease
		1.16E-08	KEGG:04380	Osteoclast differentiation
		1.22E-08	KEGG:05330	Allograft rejection
		1.75E-08	REAC:877300	Interferon gamma signaling
		3.98E-08	KEGG:04514	Cell adhesion molecules (CAMs)
		6.33E-08	KEGG:04940	Type I diabetes mellitus
		6.33E-08	KEGG:05320	Autoimmune thyroid disease
		1.26E-07	KEGG:04672	Intestinal immune network for IgA production
		1.58E-07	KEGG:00000	KEGG pathways
		1.80E-07	KEGG:05416	Viral myocarditis
		2.63E-07	REAC:1280215	Cytokine Signaling in Immune system
		3.81E-07	KEGG:05323	Rheumatoid arthritis
		9.33E-07	REAC:202427	Phosphorylation of CD3 and TCR zeta chains
		1.48E-06	REAC:389948	PD-1 signaling
		2.44E-06	REAC:202733	Cell surface interactions at the vascular wall
		3.65E-06	KEGG:05164	Influenza A
		4.59E-06	KEGG:05168	Herpes simplex infection
		5.87E-06	REAC:109582	Hemostasis
		7.31E-06	REAC:202403	TCR signaling
		1.01E-05	KEGG:05321	Inflammatory bowel disease (IBD)
		2.66E-05	REAC:202430	Translocation of ZAP-70 to Immunological synapse

	3.95E-05	REAC:2132295	MHC class II antigen presentation
	4.41E-05	REAC:913531	Interferon Signaling
	5.03E-05	REAC:202433	Generation of second messenger molecules
	7.45E-05	KEGG:05133	Pertussis
	8.93E-05	KEGG:04666	Fc gamma R-mediated phagocytosis
	9.47E-05	KEGG:04640	Hematopoietic cell lineage
	1.20E-04	REAC:168898	Toll-Like Receptors Cascades
	1.58E-04	REAC:0000000	Reactome pathways
	1.80E-04	REAC:202424	Downstream TCR signaling
	2.21E-04	REAC:388841	Costimulation by the CD28 family
	4.83E-04	REAC:1679131	Trafficking and processing of endosomal TLR
	8.25E-04	KEGG:04610	Complement and coagulation cascades
	8.89E-04	KEGG:04611	Platelet activation
	1.03E-03	KEGG:04650	Natural killer cell mediated cytotoxicity
	1.12E-03	REAC:418038	Nucleotide-like (purinergic) receptors
	1.53E-03	KEGG:04662	B cell receptor signaling pathway
	1.61E-03	REAC:2029481	FCGR activation
	3.02E-03	KEGG:05145	Toxoplasmosis
	3.03E-03	REAC:166663	Initial triggering of complement
	5.11E-03	REAC:173623	Classical antibody-mediated complement activation
	8.25E-03	KEGG:05340	Primary immunodeficiency
	1.33E-02	KEGG:05166	HTLV-I infection
	1.78E-02	KEGG:04015	Rap1 signaling pathway
	1.85E-02	REAC:2029485	Role of phospholipids in phagocytosis
	2.10E-02	REAC:417957	P2Y receptors
	3.29E-02	REAC:373076	Class A/1 (Rhodopsin-like receptors)
	3.49E-02	REAC:5579029	Metabolic disorders of biological oxidation enzymes
	3.63E-02	REAC:418594	G alpha (i) signalling events
	4.01E-02	REAC:198933	Immunoregulatory interactions between a Lymphoid and a non-Lymphoid cell
	4.07E-02	REAC:2162123	Synthesis of Prostaglandins (PG) and Thromboxanes (TX)
	4.07E-02	REAC:166786	Creation of C4 and C2 activators
	4.89E-02	KEGG:04142	Lysosome
Red	1.10E-02	REAC:4839726	Chromatin organization
	1.10E-02	REAC:3247509	Chromatin modifying enzymes
	1.30E-02	REAC:3214847	HATs acetylate histones
	3.17E-02	REAC:3214815	HDACs deacetylate histones
Purple	1.01E-05	KEGG:04141	Protein processing in endoplasmic reticulum
	3.27E-05	REAC:162906	HIV Infection
	9.66E-05	REAC:1428517	The citric acid (TCA) cycle and respiratory electron transport

1.11E-04	KEGG:00190	Oxidative phosphorylation
3.56E-04	REAC:5362768	Hh ligand biogenesis disease
3.88E-04	REAC:162909	Host Interactions of HIV factors
9.10E-04	REAC:1430728	Metabolism
1.20E-03	REAC:5387390	Processing-defective Hh variants abrogate ligand secretion
1.20E-03	REAC:5358346	Hedgehog ligand biogenesis
2.06E-03	REAC:180585	Vif-mediated degradation of APOBEC3G
2.28E-03	REAC:5368287	Mitochondrial translation
2.35E-03	REAC:74160	Gene Expression
2.52E-03	REAC:5357801	Programmed Cell Death
3.47E-03	REAC:163200	Respiratory electron transport, ATP synthesis by chemiosmotic coupling, and heat production by uncoupling proteins.
3.94E-03	KEGG:05016	Huntington's disease
4.47E-03	KEGG:05010	Alzheimer's disease
5.77E-03	REAC:180534	Vpu mediated degradation of CD4
7.18E-03	REAC:109581	Apoptosis
8.83E-03	REAC:5368286	Mitochondrial translation initiation
1.03E-02	REAC:174113	SCF-beta-TrCP mediated degradation of Emi1
1.08E-02	REAC:5419276	Mitochondrial translation termination
1.08E-02	REAC:5389840	Mitochondrial translation elongation
1.29E-02	REAC:182971	EGFR downregulation
1.46E-02	REAC:68882	Mitotic Anaphase
1.60E-02	REAC:211733	Regulation of activated PAK-2p34 by proteasome mediated degradation
1.67E-02	REAC:2555396	Mitotic Metaphase and Anaphase
1.73E-02	KEGG:01100	Metabolic pathways
1.83E-02	REAC:611105	Respiratory electron transport
2.12E-02	REAC:69017	CDK-mediated phosphorylation and removal of Cdc6
2.12E-02	REAC:69229	Ubiquitin-dependent degradation of Cyclin D1
2.12E-02	REAC:75815	Ubiquitin-dependent degradation of Cyclin D
2.12E-02	REAC:169911	Regulation of Apoptosis
2.20E-02	REAC:68886	M Phase
2.37E-02	REAC:174184	Cdc20:Phospho-APC/C mediated degradation of Cyclin A
2.41E-02	KEGG:03050	Proteasome
2.67E-02	REAC:2467813	Separation of Sister Chromatids
2.79E-02	REAC:69610	p53-Independent DNA Damage Response
2.79E-02	REAC:69613	p53-Independent G1/S DNA damage checkpoint
2.79E-02	REAC:349425	Autodegradation of the E3 ubiquitin ligase COP1
2.79E-02	REAC:69601	Ubiquitin Mediated Degradation of Phosphorylated Cdc25A
2.96E-02	REAC:174084	Autodegradation of Cdh1 by Cdh1:APC/C
2.99E-02	REAC:179419	APC:Cdc20 mediated degradation of cell cycle proteins prior to satisfation of the cell cycle checkpoint

		3.61E-02	REAC:176408	Regulation of APC/C activators between G1/S and early anaphase
		3.64E-02	REAC:69541	Stabilization of p53
		3.77E-02	REAC:174154	APC/C:Cdc20 mediated degradation of Securin
		3.77E-02	REAC:187577	SCF(Skp2)-mediated degradation of p27/p21
		4.67E-02	REAC:176409	APC/C:Cdc20 mediated degradation of mitotic proteins
		4.77E-02	REAC:5610785	GLI3 is processed to GLI3R by the proteasome
		4.77E-02	REAC:5610780	Degradation of GLI1 by the proteasome
		4.77E-02	REAC:5610783	Degradation of GLI2 by the proteasome
Lightcyan		1.57E-02	REAC:74160	Gene Expression
		2.52E-02	KEGG:03040	Spliceosome
Blue		1.45E-11	REAC:212436	Generic Transcription Pathway
		2.46E-04	REAC:74160	Gene Expression
		7.06E-03	KEGG:04060	Cytokine-cytokine receptor interaction
Turquoise		2.24E-07	REAC:5579029	Metabolic disorders of biological oxidation enzymes
		4.01E-07	REAC:500792	GPCR ligand binding
		4.01E-07	REAC:5579031	Defective ACTH causes Obesity and Pro-opiomelanocortinin deficiency (POMCD)
		9.72E-07	KEGG:04974	Protein digestion and absorption
		4.59E-06	REAC:381753	Olfactory Signaling Pathway
		7.05E-06	REAC:373076	Class A/1 (Rhodopsin-like receptors)
		4.44E-04	REAC:372790	Signaling by GPCR
		5.12E-04	KEGG:04740	Olfactory transduction
		9.14E-04	REAC:388396	GPCR downstream signaling
		1.21E-03	REAC:2022090	Assembly of collagen fibrils and other multimeric structures
		5.44E-03	KEGG:04060	Cytokine-cytokine receptor interaction
		5.47E-03	REAC:1650814	Collagen biosynthesis and modifying enzymes
		9.17E-03	REAC:1474290	Collagen formation
		3.67E-02	REAC:549132	Organic cation/anion/zwitterion transport
		4.21E-02	KEGG:05217	Basal cell carcinoma
Lightyellow	TCTX	4.21E-02	REAC:75955	RNA Polymerase II Transcription Elongation
		4.21E-02	REAC:167246	Tat-mediated elongation of the HIV-1 transcript
		4.21E-02	REAC:167152	Formation of HIV elongation complex in the absence of HIV Tat
		4.21E-02	REAC:112382	Formation of RNA Pol II elongation complex
		4.21E-02	REAC:167169	HIV Transcription Elongation
		4.21E-02	REAC:167200	Formation of HIV-1 elongation complex containing HIV-1 Tat
Cyan		6.29E-03	REAC:4791275	Signaling by WNT in cancer
		7.61E-03	REAC:195721	Signaling by Wnt
		1.21E-02	KEGG:04142	Lysosome
		4.99E-02	REAC:201681	TCF dependent signaling in response to WNT
		4.99E-02	REAC:5339717	misspliced LRP5 mutants have enhanced beta-catenin-dependent signaling

	4.99E-02	REAC:5545619	XAV939 inhibits tankyrase, stabilizing AXIN
	4.99E-02	REAC:5340588	RNF mutants show enhanced WNT signaling and proliferation
Green	2.30E-05	REAC:983168	Antigen processing: Ubiquitination & Proteasome degradation
	9.76E-05	REAC:3214847	HATs acetylate histones
	1.17E-04	REAC:983169	Class I MHC mediated antigen processing & presentation
	2.40E-04	REAC:4839726	Chromatin organization
	2.40E-04	REAC:3247509	Chromatin modifying enzymes
	1.68E-03	REAC:1852241	Organelle biogenesis and maintenance
	2.24E-03	KEGG:04120	Ubiquitin mediated proteolysis
	2.89E-02	REAC:390450	Folding of actin by CCT/TriC
	3.01E-02	REAC:379716	Cytosolic tRNA aminoacylation
Lightcyan	3.13E-22	KEGG:05150	Staphylococcus aureus infection
	8.03E-22	REAC:168256	Immune System
	3.33E-18	KEGG:05152	Tuberculosis
	4.40E-13	KEGG:05140	Leishmaniasis
	7.20E-13	KEGG:04145	Phagosome
	1.04E-11	REAC:168249	Innate Immune System
	1.72E-11	KEGG:05322	Systemic lupus erythematosus
	4.00E-10	KEGG:04380	Osteoclast differentiation
	1.64E-09	REAC:1280215	Cytokine Signaling in Immune system
	2.56E-09	REAC:1280218	Adaptive Immune System
	3.89E-09	KEGG:05332	Graft-versus-host disease
	1.29E-08	KEGG:04612	Antigen processing and presentation
	2.81E-08	REAC:877300	Interferon gamma signaling
	1.19E-07	KEGG:05133	Pertussis
	1.25E-07	KEGG:05310	Asthma
	2.55E-07	KEGG:05323	Rheumatoid arthritis
	5.96E-07	KEGG:05330	Allograft rejection
	7.44E-07	REAC:168898	Toll-Like Receptors Cascades
	1.83E-06	KEGG:05321	Inflammatory bowel disease (IBD)
	2.15E-06	KEGG:04514	Cell adhesion molecules (CAMs)
	3.02E-06	KEGG:04940	Type I diabetes mellitus
	3.02E-06	KEGG:05320	Autoimmune thyroid disease
	5.97E-06	KEGG:04672	Intestinal immune network for IgA production
	1.35E-05	KEGG:05416	Viral myocarditis
	1.57E-05	KEGG:00000	KEGG pathways
	2.37E-05	REAC:202427	Phosphorylation of CD3 and TCR zeta chains
	3.27E-05	KEGG:04640	Hematopoietic cell lineage
	3.37E-05	REAC:913531	Interferon Signaling

3.64E-05	KEGG:05134	Legionellosis
3.75E-05	REAC:389948	PD-1 signaling
3.80E-05	KEGG:04662	B cell receptor signaling pathway
7.88E-05	KEGG:04650	Natural killer cell mediated cytotoxicity
7.88E-05	KEGG:04666	Fc gamma R-mediated phagocytosis
1.31E-04	KEGG:05164	Influenza A
1.66E-04	KEGG:05168	Herpes simplex infection
1.86E-04	KEGG:04611	Platelet activation
3.61E-04	KEGG:05145	Toxoplasmosis
3.77E-04	REAC:202733	Cell surface interactions at the vascular wall
3.96E-04	REAC:202430	Translocation of ZAP-70 to Immunological synapse
4.30E-04	REAC:2029485	Role of phospholipids in phagocytosis
4.56E-04	REAC:202403	TCR signaling
5.31E-04	REAC:198933	Immunoregulatory interactions between a Lymphoid and a non-Lymphoid cell
7.06E-04	REAC:449147	Signaling by Interleukins
7.15E-04	REAC:388841	Costimulation by the CD28 family
1.19E-03	REAC:202433	Generation of second messenger molecules
1.71E-03	REAC:109582	Hemostasis
2.30E-03	REAC:983695	Antigen activates B Cell Receptor (BCR) leading to generation of second messengers
2.75E-03	KEGG:04620	Toll-like receptor signaling pathway
3.01E-03	REAC:0000000	Reactome pathways
3.47E-03	REAC:2132295	MHC class II antigen presentation
4.13E-03	REAC:202424	Downstream TCR signaling
4.54E-03	REAC:166016	Toll Like Receptor 4 (TLR4) Cascade
5.39E-03	KEGG:05340	Primary immunodeficiency
8.21E-03	REAC:512988	Interleukin-3, 5 and GM-CSF signaling
8.68E-03	REAC:2029480	Fcgamma receptor (FCGR) dependent phagocytosis
9.72E-03	REAC:418038	Nucleotide-like (purinergic) receptors
1.26E-02	KEGG:05020	Prion diseases
1.39E-02	REAC:2029481	FCGR activation
1.41E-02	KEGG:05166	HTLV-I infection
1.57E-02	KEGG:04064	NF-kappa B signaling pathway
1.89E-02	KEGG:04610	Complement and coagulation cascades
2.07E-02	REAC:2871809	FCERI mediated Ca+2 mobilization
2.58E-02	REAC:166663	Initial triggering of complement
2.65E-02	REAC:173623	Classical antibody-mediated complement activation
2.70E-02	REAC:373076	Class A/1 (Rhodopsin-like receptors)
2.82E-02	KEGG:04664	Fc epsilon RI signaling pathway
4.40E-02	KEGG:04015	Rap1 signaling pathway

	4.92E-02	KEGG:05142	Chagas disease (American trypanosomiasis)
Darkturquoise	4.86E-05	KEGG:04141	Protein processing in endoplasmic reticulum
Purple	5.25E-06	KEGG:04141	Protein processing in endoplasmic reticulum
	4.79E-05	REAC:74160	Gene Expression
	6.86E-05	REAC:180585	Vif-mediated degradation of APOBEC3G
	1.13E-04	REAC:983168	Antigen processing: Ubiquitination & Proteasome degradation
	1.14E-04	REAC:983169	Class I MHC mediated antigen processing & presentation
	2.64E-04	REAC:5362768	Hh ligand biogenesis disease
	2.73E-04	REAC:162906	HIV Infection
	3.09E-04	REAC:180534	Vpu mediated degradation of CD4
	3.84E-04	REAC:450531	Regulation of mRNA stability by proteins that bind AU-rich elements
	5.28E-04	REAC:174113	SCF-beta-TrCP mediated degradation of Emi1
	6.55E-04	REAC:162909	Host Interactions of HIV factors
	6.86E-04	REAC:5387390	Processing-defective Hh variants abrogate ligand secretion
	6.86E-04	REAC:5358346	Hedgehog ligand biogenesis
	1.36E-03	REAC:211733	Regulation of activated PAK-2p34 by proteasome mediated degradation
	1.62E-03	REAC:69202	Cyclin E associated events during G1/S transition
	1.76E-03	REAC:69017	CDK-mediated phosphorylation and removal of Cdc6
	1.76E-03	REAC:69229	Ubiquitin-dependent degradation of Cyclin D1
	1.76E-03	REAC:75815	Ubiquitin-dependent degradation of Cyclin D
	1.76E-03	REAC:169911	Regulation of Apoptosis
	1.77E-03	REAC:187577	SCF(Skp2)-mediated degradation of p27/p21
	1.99E-03	REAC:69656	Cyclin A:Cdk2-associated events at S phase entry
	2.21E-03	REAC:5610785	GLI3 is processed to GLI3R by the proteasome
	2.21E-03	REAC:5610780	Degradation of GLI1 by the proteasome
	2.21E-03	REAC:5610783	Degradation of GLI2 by the proteasome
	2.26E-03	REAC:69610	p53-Independent DNA Damage Response
	2.26E-03	REAC:69613	p53-Independent G1/S DNA damage checkpoint
	2.26E-03	REAC:349425	Autodegradation of the E3 ubiquitin ligase COP1
	2.26E-03	REAC:69601	Ubiquitin Mediated Degradation of Phosphorylated Cdc25A
	2.88E-03	REAC:69541	Stabilization of p53
	3.39E-03	REAC:1236974	ER-Phagosome pathway
	3.55E-03	REAC:176408	Regulation of APC/C activators between G1/S and early anaphase
	4.16E-03	REAC:174184	Cdc20:Phospho-APC/C mediated degradation of Cyclin A
	4.58E-03	REAC:4641257	degradation of AXIN
	5.08E-03	REAC:179419	APC:Cdc20 mediated degradation of cell cycle proteins prior to satisfation of the cell cycle checkpoint
	5.39E-03	KEGG:03050	Proteasome
	5.71E-03	REAC:450408	AUF1 (hnRNP D0) destabilizes mRNA
	5.71E-03	REAC:68827	CDT1 association with the CDC6:ORC:origin complex

6.49E-03	KEGG:04120	Ubiquitin mediated proteolysis
7.08E-03	REAC:4641258	degradation of DVL
7.47E-03	REAC:176409	APC/C:Cdc20 mediated degradation of mitotic proteins
7.49E-03	REAC:1236978	Cross-presentation of soluble exogenous antigens (endosomes)
7.57E-03	KEGG:00510	N-Glycan biosynthesis
8.72E-03	REAC:69580	p53-Dependent G1/S DNA damage checkpoint
8.72E-03	REAC:174084	Autodegradation of Cdh1 by Cdh1:APC/C
8.72E-03	REAC:69563	p53-Dependent G1 DNA Damage Response
9.00E-03	REAC:176814	Activation of APC/C and APC/C:Cdc20 mediated degradation of mitotic proteins
9.00E-03	REAC:1169091	Activation of NF-kappaB in B cells
1.02E-02	REAC:174143	APC/C-mediated degradation of cell cycle proteins
1.02E-02	REAC:453276	Regulation of mitotic cell cycle
1.07E-02	REAC:174154	APC/C:Cdc20 mediated degradation of Securin
1.08E-02	REAC:69052	Switching of origins to a post-replicative state
1.08E-02	REAC:68949	Orc1 removal from chromatin
1.19E-02	REAC:350562	Regulation of ornithine decarboxylase (ODC)
1.30E-02	REAC:69615	G1/S DNA Damage Checkpoints
1.42E-02	REAC:4086400	PCP/CE pathway
1.53E-02	REAC:69300	Removal of licensing factors from origins
1.91E-02	REAC:5467345	deletions in the AXIN genes in hepatocellular carcinoma result in elevated WNT signaling
1.91E-02	REAC:5467343	deletions in the AMER1 gene destabilize the destruction complex
1.91E-02	REAC:5358749	S37 mutants of beta-catenin aren't phosphorylated
1.91E-02	REAC:4839744	truncated APC mutants destabilize the destruction complex
1.91E-02	REAC:5467340	AXIN missense mutants destabilize the destruction complex
1.91E-02	REAC:5339700	TCF7L2 mutants don't bind CTBP
1.91E-02	REAC:4839735	AXIN mutants destabilize the destruction complex, activating WNT signaling
1.91E-02	REAC:4839748	AMER1 mutants destabilize the destruction complex
1.91E-02	REAC:5358751	S45 mutants of beta-catenin aren't phosphorylated
1.91E-02	REAC:5339716	misspliced GSK3beta mutants stabilize beta-catenin
1.91E-02	REAC:5358747	S33 mutants of beta-catenin aren't phosphorylated
1.91E-02	REAC:5467348	truncations of AMER1 destabilize the destruction complex
1.91E-02	REAC:195253	Degradation of beta-catenin by the destruction complex
1.91E-02	REAC:5358752	T41 mutants of beta-catenin aren't phosphorylated
1.91E-02	REAC:5467337	APC truncation mutants have impaired AXIN binding
1.91E-02	REAC:5467333	APC truncation mutants are not K63 polyubiquitinated
1.91E-02	REAC:4839743	phosphorylation site mutants of CTNNB1 are not targeted to the proteasome by the destruction complex
2.14E-02	REAC:69304	Regulation of DNA replication
2.52E-02	REAC:1236975	Antigen processing-Cross presentation
2.72E-02	REAC:174178	APC/C:Cdh1 mediated degradation of Cdc20 and other APC/C:Cdh1 targeted proteins in late mitosis/early G1

	2.72E-02	REAC:68867	Assembly of the pre-replicative complex
	2.72E-02	REAC:4608870	Asymmetric localization of PCP proteins
	2.85E-02	REAC:69239	Synthesis of DNA
	2.97E-02	REAC:5389840	Mitochondrial translation elongation
	3.07E-02	REAC:1280218	Adaptive Immune System
	3.43E-02	REAC:199991	Membrane Trafficking
Grey60	8.98E-04	KEGG:05032	Morphine addiction
	2.04E-03	KEGG:04723	Retrograde endocannabinoid signaling
	3.64E-03	KEGG:04080	Neuroactive ligand-receptor interaction
	5.08E-03	KEGG:04024	cAMP signaling pathway
	1.18E-02	REAC:111885	Opioid Signalling
	3.66E-02	REAC:112316	Neuronal System
	4.87E-02	KEGG:04727	GABAergic synapse
Midnightblue	8.33E-05	REAC:74160	Gene Expression
	1.23E-02	REAC:159236	Transport of Mature mRNA derived from an Intron-Containing Transcript
	1.30E-02	REAC:72203	Processing of Capped Intron-Containing Pre-mRNA
	1.50E-02	REAC:180910	Vpr-mediated nuclear import of PICs
	2.18E-02	REAC:72202	Transport of Mature Transcript to Cytoplasm
	2.22E-02	REAC:176033	Interactions of Vpr with host cellular proteins
	2.22E-02	REAC:159227	Transport of the SLBP independent Mature mRNA
	2.49E-02	REAC:429914	Deadenylation-dependent mRNA decay
	2.67E-02	REAC:159230	Transport of the SLBP Dependant Mature mRNA
	3.77E-02	REAC:159231	Transport of Mature mRNA Derived from an Intronless Transcript
	4.44E-02	REAC:159234	Transport of Mature mRNAs Derived from Intronless Transcripts
	4.86E-02	REAC:162587	HIV Life Cycle
Pink	5.68E-11	REAC:212436	Generic Transcription Pathway
	1.84E-05	REAC:74160	Gene Expression
	4.00E-02	REAC:72689	Formation of a pool of free 40S subunits
Magenta	3.15E-06	REAC:112316	Neuronal System
	5.85E-03	REAC:1296071	Potassium Channels
	9.81E-03	REAC:1296072	Voltage gated Potassium channels

ST 18. Statistics of modules in brain areas other than FCTX and TCTX. Module size and associated module membership (MM) and 1-quantile of MM (1-q) are shown. All FTD-genes (pure and spectrum) were assessed in PUTM, THAL, HIPP and WHMT. In addition, the spectrum FTD-genes were also evaluated in CRBL and MEDU.

Gene	Type	XID	PUTM				THAL				HIPP				WHMT				CRBL				MEDU			
			module	sz	MM	1-q	module	sz	MM	1-q	module	sz	MM	1-q	module	sz	MM	1-q	module	sz	MM	1-q	module	sz	MM	1-q
**MAPT**	pure	X3723687	purple	322	0.54	0.50	skyblue	59	0.78	0.29	green	670	0.57	0.4	darkgrey	125	0.49	0.83	darkred	169	0.72	0.11	darkgrey	97	0.85	0.07
**GRN**	pure	X3722917	purple	322	0.53	0.53	royalblue	2042	0.16	0.95	darkorange	169	0.41	0.78	brown	2140	0.59	0.23	blue	2917	0.39	0.45	pink	548	0.59	0.68
**CHMP2B**	pure	X2631845	green	1249	0.65	0.25	green	1620	0.47	0.65	greenyellow	426	**0.61**	**0.28**	salmon	311	0.7	0.24	lightyellow	193	0.73	0.08	blue	4141	0.64	0.31
**HLA-DRA**	pure	X2903189	royalblue	138	0.79	0.21	midnightblue	187	0.72	0.53	**darkgrey**	**200**	**0.77**	**0.42**	**black**	**715**	**0.78**	**0.21**	grey60	235	0.7	0.46	pink	548	0.84	0.19
**CTSC**	pure	X3385769	royalblue	138	0.68	0.60	darkorange	312	0.78	0.18	**darkgrey**	**200**	**0.79**	**0.35**	**black**	**715**	**0.87**	**0.07**	grey60	235	0.82	0.17	pink	548	0.89	0.1
**TMEM106B**	pure	X2990342	green	1249	**0.87**	**0.00**	darkred	561	0.72	0.43	greenyellow	426	**0.69**	**0.08**	tan	347	**0.78**	**0.08**	red	786	0.8	0.02	darkgreen	111	0.75	0.55
**C9orf72**	spectrum	X3202421	brown	1777	0.43	0.53	lightyellow	862	**0.8**	**0.15**	**grey60**	**357**	**0.7**	**0.13**	royalblue	186	0.69	0.37	**purple**	**564**	**0.59**	**0.36**	**grey60**	**292**	**0.69**	**0.41**
**VCP**	spectrum	X3204404	greenyellow	299	0.79	0.16	lightyellow	862	**0.84**	**0.05**	black	521	**0.78**	**0.17**	darkturquoise	137	0.82	**0.09**	magenta	665	0.57	0.31	**grey60**	**292**	**0.72**	**0.32**
**OPTN**	spectrum	X3235726	blue	2455	0.37	0.71	cyan	229	0.65	0.42	**grey60**	**357**	**0.56**	**0.48**	blue	2675	0.28	0.85	**purple**	**564**	**0.5**	**0.55**	green	808	0.64	0.56
**UBQLN2**	spectrum	X3978999	brown	1777	**0.84**	**0.02**	blue	2691	0.71	0.18	black	521	**0.8**	**0.11**	royalblue	186	0.57	0.74	greenyellow	526	0.5	0.55	greenyellow	510	0.75	0.43
**FUS**	spectrum	X3656954	turquoise	7809	0.45	0.60	turquoise	3224	0.37	0.87	turquoise	4305	0.27	0.95	turquoise	4815	0.31	0.85	turquoise	6022	0.44	0.51	turquoise	5207	0.46	0.62
**FUS**	spectrum	X3656950	turquoise	7809	0.32	0.78	magenta	682	0.1	0.99	yellow	808	0.31	0.94	green	1244	0.39	0.78	lightcyan	257	0.5	0.76	turquoise	5207	0.27	0.88
**FUS**	spectrum	X3656904	magenta	328	0.62	0.45	green	1620	0.13	0.99	skyblue	**157**	**0.75**	**0.33**	yellow	1727	0.56	0.4	yellow	981	0.65	0.2	blue	4141	0.56	0.42
**TDP-43**	spectrum	X2320048	blue	2455	0.1	0.99	green	1620	0.41	0.72	skyblue	**157**	**0.51**	**0.64**	pink	535	0.54	0.46	turquoise	6022	0.36	0.61	blue	4141	0.42	0.63

ST 19. Functional annotation analysis of modules in brain areas other than FCTX and TCTX.

		BPs			CCs			MFs		
module	tissue	p-value	term ID	term name	p-value	term ID	term name	p-value	term ID	term name
purple	PUTM	1.49E-03	GO:0019219	regulation of nucleobase-containing compound metabolic process	3.96E-04	GO:0005654	nucleoplasm	3.63E-02	GO:0008134	transcription factor binding
		2.18E-03	GO:0051171	regulation of nitrogen compound metabolic process	3.12E-03	GO:0031981	nuclear lumen			
		2.81E-03	GO:0001701	in utero embryonic development	5.18E-03	GO:0043233	organelle lumen			
		5.97E-03	GO:0050794	regulation of cellular process	9.43E-03	GO:0070013	intracellular organelle lumen			
		1.13E-02	GO:0045934	negative regulation of nucleobase-containing compound metabolic process	9.74E-03	GO:0031974	membrane-enclosed lumen			
		1.39E-02	GO:0032774	RNA biosynthetic process	2.57E-02	GO:0005634	nucleus			
		1.93E-02	GO:0010629	negative regulation of gene expression	3.58E-02	GO:0044428	nuclear part			
		2.24E-02	GO:0006366	transcription from RNA polymerase II promoter						
		2.49E-02	GO:0050789	regulation of biological process						
		2.54E-02	GO:0010468	regulation of gene expression						
		2.63E-02	GO:0051252	regulation of RNA metabolic process						
		2.78E-02	GO:0097659	nucleic acid-templated transcription						
		2.88E-02	GO:0031326	regulation of cellular biosynthetic process						
		3.26E-02	GO:0006357	regulation of transcription from RNA polymerase II promoter						
		3.38E-02	GO:2000112	regulation of cellular macromolecule biosynthetic process						
		3.50E-02	GO:0019222	regulation of metabolic process						
		3.74E-02	GO:0034654	nucleobase-containing compound biosynthetic process						
		4.08E-02	GO:0006351	transcription, DNA-templated						
		4.28E-02	GO:1903506	regulation of nucleic acid-templated transcription						
		4.42E-02	GO:0009889	regulation of biosynthetic process						
		4.93E-02	GO:0016070	RNA metabolic process						
		4.98E-02	GO:0051253	negative regulation of RNA metabolic process						
green		1.96E-10	GO:0046907	intracellular transport	4.61E-24	GO:0043231	intracellular membrane-bounded organelle	7.82E-08	GO:0003723	RNA binding
		3.38E-09	GO:1902582	single-organism intracellular transport	1.21E-21	GO:0043227	membrane-bounded organelle	2.69E-07	GO:0003824	catalytic activity
		1.18E-08	GO:0016482	cytoplasmic transport	2.87E-21	GO:0044446	intracellular organelle part	3.35E-07	GO:0044822	poly(A) RNA binding
		2.36E-08	GO:0015031	protein transport	4.31E-20	GO:0043229	intracellular organelle	9.91E-04	GO:1901363	heterocyclic compound binding
		4.65E-08	GO:0045184	establishment of protein localization	1.45E-19	GO:0044422	organelle part	2.08E-03	GO:0000166	nucleotide binding
		1.55E-07	GO:0008104	protein localization	2.32E-19	GO:0005622	intracellular	2.08E-03	GO:1901265	nucleoside phosphate binding
		4.41E-07	GO:0033036	macromolecule localization	1.64E-18	GO:0044424	intracellular part	2.27E-03	GO:0097159	organic cyclic compound binding
		2.00E-06	GO:0044267	cellular protein metabolic process	3.92E-17	GO:0043226	organelle	3.78E-03	GO:0008565	protein transporter activity
		5.51E-06	GO:0071702	organic substance transport	4.30E-16	GO:0005737	cytoplasm	6.54E-03	GO:0016874	ligase activity
		7.04E-06	GO:0051649	establishment of localization in cell	1.35E-15	GO:0044444	cytoplasmic part	1.08E-02	GO:0016740	transferase activity
		9.27E-06	GO:0051641	cellular localization	2.87E-12	GO:0044428	nuclear part	1.19E-02	GO:0036094	small molecule binding
		1.40E-05	GO:0033365	protein localization to organelle	2.15E-11	GO:0070013	intracellular organelle lumen	1.24E-02	GO:0019787	small conjugating protein transferase activity
		2.34E-05	GO:0044260	cellular macromolecule metabolic process	9.16E-11	GO:0031981	nuclear lumen	1.58E-02	GO:0032549	ribonucleoside binding
		2.71E-05	GO:0006886	intracellular protein transport	1.52E-10	GO:0031974	membrane-enclosed lumen	2.12E-02	GO:0001882	nucleoside binding
		3.49E-05	GO:1902580	single-organism cellular localization	1.68E-10	GO:0043233	organelle lumen	2.87E-02	GO:0032553	ribonucleotide binding

	6.32E-05	GO:0034613	cellular protein localization	8.60E-10	GO:0031090	organelle membrane	3.21E-02	GO:0032550	purine ribonucleoside binding
	9.69E-05	GO:0070727	cellular macromolecule localization	3.34E-09	GO:0005634	nucleus	3.57E-02	GO:0001883	purine nucleoside binding
	9.96E-05	GO:0044237	cellular metabolic process	1.03E-08	GO:0005654	nucleoplasm	3.65E-02	GO:0017076	purine nucleotide binding
	4.20E-04	GO:0008152	metabolic process	2.55E-06	GO:0044464	cell part	3.67E-02	GO:0035639	purine ribonucleoside triphosphate binding
	4.49E-04	GO:0044238	primary metabolic process	2.55E-06	GO:0005623	cell			
	7.55E-04	GO:0071704	organic substance metabolic process	5.77E-06	GO:0005739	mitochondrion			
	9.84E-04	GO:0072594	establishment of protein localization to organelle	1.97E-05	GO:0032991	macromolecular complex			
	1.50E-03	GO:0048193	Golgi vesicle transport	4.36E-05	GO:0012505	endomembrane system			
	1.65E-03	GO:0007005	mitochondrion organization	1.08E-04	GO:0030529	ribonucleoprotein complex			
	2.08E-03	GO:0043170	macromolecule metabolic process	1.87E-04	GO:0031967	organelle envelope			
	2.71E-03	GO:0044265	cellular macromolecule catabolic process	2.37E-04	GO:0031975	envelope			
	2.81E-03	GO:0019941	modification-dependent protein catabolic process	4.06E-04	GO:0005829	cytosol			
	2.85E-03	GO:0006605	protein targeting	5.86E-04	GO:1902494	catalytic complex			
	2.87E-03	GO:0019538	protein metabolic process	5.94E-04	GO:0098588	bounding membrane of organelle			
	3.64E-03	GO:0022613	ribonucleoprotein complex biogenesis	7.27E-04	GO:0044429	mitochondrial part			
	4.28E-03	GO:0030163	protein catabolic process	1.69E-03	GO:0005794	Golgi apparatus			
	4.42E-03	GO:0043632	modification-dependent macromolecule catabolic process	1.56E-02	GO:1990234	transferase complex			
	5.38E-03	GO:0009057	macromolecule catabolic process	3.96E-02	GO:0043234	protein complex			
	6.85E-03	GO:0006511	ubiquitin-dependent protein catabolic process						
	7.20E-03	GO:0044257	cellular protein catabolic process						
	7.76E-03	GO:0006396	RNA processing						
	8.88E-03	GO:0070647	protein modification by small protein conjugation or removal						
	9.72E-03	GO:0061024	membrane organization						
	9.82E-03	GO:0006412	translation						
	1.36E-02	GO:0044248	cellular catabolic process						
	1.69E-02	GO:0051603	proteolysis involved in cellular protein catabolic process						
	3.49E-02	GO:0036211	protein modification process						
	3.49E-02	GO:0006464	cellular protein modification process						
	4.22E-02	GO:0032446	protein modification by small protein conjugation						
	4.83E-02	GO:0034660	ncRNA metabolic process						
	1.32E-32	GO:0006955	immune response	3.39E-10	GO:0071944	cell periphery	4.44E-12	GO:0004872	receptor activity
	1.65E-29	GO:0002376	immune system process	4.10E-10	GO:0005886	plasma membrane	1.36E-08	GO:0038023	signaling receptor activity
	2.41E-25	GO:0006952	defense response	8.26E-10	GO:0016021	integral component of membrane	1.94E-08	GO:0060089	molecular transducer activity
	1.50E-22	GO:0002684	positive regulation of immune system process	1.19E-09	GO:0044459	plasma membrane part	1.94E-08	GO:0004871	signal transducer activity
	2.28E-22	GO:0002682	regulation of immune system process	1.53E-09	GO:0016020	membrane	1.43E-07	GO:0004888	transmembrane signaling receptor activity
royalblue	1.19E-19	GO:0050778	positive regulation of immune response	1.57E-09	GO:0044425	membrane part	4.57E-06	GO:0003823	antigen binding
	5.12E-18	GO:0001775	cell activation	2.76E-09	GO:0031224	intrinsic component of membrane	1.95E-04	GO:0019864	IgG binding
	1.03E-17	GO:0050776	regulation of immune response	8.20E-08	GO:0042613	MHC class II protein complex	8.83E-03	GO:0023026	MHC class II protein complex binding
	6.36E-17	GO:0002253	activation of immune response	2.04E-06	GO:0005887	integral component of plasma membrane	1.23E-02	GO:0019865	immunoglobulin binding
	8.28E-17	GO:0045321	leukocyte activation	5.18E-06	GO:0031226	intrinsic component of plasma membrane	1.66E-02	GO:0023023	MHC protein complex binding
	1.04E-	GO:0050	response to stimulus	8.85E-	GO:0031	membrane-bounded vesicle	2.93E-	GO:0032	MHC class II receptor activity

16	896		06	988		02	395	
2.24E-15	GO:0045087	innate immune response	1.14E-05	GO:0042611	MHC protein complex	3.48E-02	GO:0032403	protein complex binding
1.23E-14	GO:0002252	immune effector process	2.90E-05	GO:0031982	vesicle			
2.47E-14	GO:0006950	response to stress	7.52E-05	GO:0098552	side of membrane			
7.40E-13	GO:0002757	immune response-activating signal transduction	1.97E-04	GO:0044421	extracellular region part			
1.19E-12	GO:0048584	positive regulation of response to stimulus	3.47E-04	GO:0005576	extracellular region			
1.11E-11	GO:0007159	leukocyte cell-cell adhesion	4.00E-04	GO:0030669	clathrin-coated endocytic vesicle membrane			
2.62E-11	GO:0048583	regulation of response to stimulus	9.06E-04	GO:0045334	clathrin-coated endocytic vesicle			
3.31E-11	GO:0042098	T cell proliferation	1.60E-03	GO:0071556	integral component of lumenal side of endoplasmic reticulum membrane			
4.30E-11	GO:0006954	inflammatory response	1.60E-03	GO:0098553	lumenal side of endoplasmic reticulum membrane			
6.13E-11	GO:0046651	lymphocyte proliferation	1.60E-03	GO:0098576	lumenal side of membrane			
6.76E-11	GO:0032943	mononuclear cell proliferation	2.46E-03	GO:0009986	cell surface			
1.08E-10	GO:0050900	leukocyte migration	4.54E-03	GO:1903561	extracellular vesicle			
1.60E-10	GO:0070661	leukocyte proliferation	4.54E-03	GO:0070062	extracellular vesicular exosome			
1.76E-10	GO:0002764	immune response-regulating signaling pathway	4.54E-03	GO:0043230	extracellular organelle			
1.78E-10	GO:0042110	T cell activation	4.54E-03	GO:0065010	extracellular membrane-bounded organelle			
1.78E-10	GO:0070489	T cell aggregation	5.87E-03	GO:0005764	lysosome			
1.90E-10	GO:0071593	lymphocyte aggregation	5.87E-03	GO:0000323	lytic vacuole			
2.45E-10	GO:0070486	leukocyte aggregation	6.16E-03	GO:0005765	lysosomal membrane			
2.85E-10	GO:0002274	myeloid leukocyte activation	6.24E-03	GO:0009897	external side of plasma membrane			
3.19E-10	GO:0046649	lymphocyte activation	6.62E-03	GO:0043235	receptor complex			
3.55E-10	GO:0034109	homotypic cell-cell adhesion	9.85E-03	GO:0030666	endocytic vesicle membrane			
5.99E-10	GO:0016337	single organismal cell-cell adhesion	1.10E-02	GO:0030139	endocytic vesicle			
1.08E-09	GO:0071345	cellular response to cytokine stimulus	1.37E-02	GO:0012507	ER to Golgi transport vesicle membrane			
1.42E-09	GO:0034097	response to cytokine	1.62E-02	GO:0032588	trans-Golgi network membrane			
2.75E-09	GO:0098602	single organism cell adhesion	1.94E-02	GO:0005615	extracellular space			
4.26E-09	GO:0051716	cellular response to stimulus	2.31E-02	GO:0005774	vacuolar membrane			
6.03E-09	GO:0032103	positive regulation of response to external stimulus	2.81E-02	GO:0005773	vacuole			
6.31E-09	GO:0007165	signal transduction	2.93E-02	GO:0036019	endolysosome			
8.60E-09	GO:1903037	regulation of leukocyte cell-cell adhesion	3.58E-02	GO:0044437	vacuolar part			
1.22E-08	GO:0002694	regulation of leukocyte activation	4.06E-02	GO:0030665	clathrin-coated vesicle membrane			
1.39E-08	GO:0007155	cell adhesion	4.80E-02	GO:0005575	cellular_component			
1.48E-08	GO:0002696	positive regulation of leukocyte activation						
1.51E-08	GO:0022610	biological adhesion						
1.60E-08	GO:0034341	response to interferon-gamma						
2.33E-08	GO:0043207	response to external biotic stimulus						
2.33E-08	GO:0051707	response to other organism						
2.88E-08	GO:0050867	positive regulation of cell activation						
2.97E-08	GO:0030595	leukocyte chemotaxis						
3.68E-08	GO:0050670	regulation of lymphocyte proliferation						
4.08E-08	GO:0032944	regulation of mononuclear cell proliferation						

4.68E-08	GO:0050865	regulation of cell activation
4.78E-08	GO:0097529	myeloid leukocyte migration
4.83E-08	GO:0048518	positive regulation of biological process
5.67E-08	GO:0001817	regulation of cytokine production
6.11E-08	GO:0002521	leukocyte differentiation
6.67E-08	GO:0070663	regulation of leukocyte proliferation
6.85E-08	GO:0009607	response to biotic stimulus
7.14E-08	GO:0050863	regulation of T cell activation
8.30E-08	GO:0002250	adaptive immune response
9.25E-08	GO:0042129	regulation of T cell proliferation
1.18E-07	GO:0050870	positive regulation of T cell activation
1.29E-07	GO:1903039	positive regulation of leukocyte cell-cell adhesion
1.38E-07	GO:0019221	cytokine-mediated signaling pathway
1.38E-07	GO:0098542	defense response to other organism
1.41E-07	GO:0034112	positive regulation of homotypic cell-cell adhesion
1.59E-07	GO:0034110	regulation of homotypic cell-cell adhesion
2.17E-07	GO:0002429	immune response-activating cell surface receptor signaling pathway
2.90E-07	GO:0002366	leukocyte activation involved in immune response
2.90E-07	GO:0002263	cell activation involved in immune response
2.95E-07	GO:0002443	leukocyte mediated immunity
3.66E-07	GO:0044700	single organism signaling
3.88E-07	GO:0023052	signaling
4.60E-07	GO:0051251	positive regulation of lymphocyte activation
5.57E-07	GO:0001816	cytokine production
5.66E-07	GO:0022407	regulation of cell-cell adhesion
7.55E-07	GO:0051249	regulation of lymphocyte activation
7.78E-07	GO:0006959	humoral immune response
8.16E-07	GO:0007154	cell communication
8.81E-07	GO:0071346	cellular response to interferon-gamma
9.64E-07	GO:0022409	positive regulation of cell-cell adhesion
1.04E-06	GO:0009611	response to wounding
1.49E-06	GO:0032101	regulation of response to external stimulus
1.52E-06	GO:0016064	immunoglobulin mediated immune response
1.62E-06	GO:0060326	cell chemotaxis
1.92E-06	GO:0002460	adaptive immune response based on somatic recombination of immune receptors built from immunoglobulin superfamily domains
2.01E-06	GO:0019724	B cell mediated immunity
2.31E-06	GO:0031347	regulation of defense response
2.44E-06	GO:0001819	positive regulation of cytokine production
2.79E-06	GO:0030155	regulation of cell adhesion
3.25E-06	GO:0009605	response to external stimulus
4.26E-	GO:0048	antigen processing and presentation of peptide antigen

06	002	
5.35E-06	GO:0042102	positive regulation of T cell proliferation
5.73E-06	GO:0002495	antigen processing and presentation of peptide antigen via MHC class II
5.78E-06	GO:0007166	cell surface receptor signaling pathway
6.48E-06	GO:0002504	antigen processing and presentation of peptide or polysaccharide antigen via MHC class II
8.32E-06	GO:1902105	regulation of leukocyte differentiation
9.91E-06	GO:0070887	cellular response to chemical stimulus
9.91E-06	GO:0002768	immune response-regulating cell surface receptor signaling pathway
1.17E-05	GO:0002478	antigen processing and presentation of exogenous peptide antigen
1.35E-05	GO:0016477	cell migration
1.46E-05	GO:0050671	positive regulation of lymphocyte proliferation
1.63E-05	GO:0032946	positive regulation of mononuclear cell proliferation
1.71E-05	GO:0002683	negative regulation of immune system process
1.72E-05	GO:0002275	myeloid cell activation involved in immune response
1.73E-05	GO:0019884	antigen processing and presentation of exogenous antigen
2.24E-05	GO:0060333	interferon-gamma-mediated signaling pathway
2.25E-05	GO:0070665	positive regulation of leukocyte proliferation
2.41E-05	GO:0048534	hematopoietic or lymphoid organ development
2.98E-05	GO:1903706	regulation of hemopoiesis
3.03E-05	GO:0051272	positive regulation of cellular component movement
3.65E-05	GO:0019882	antigen processing and presentation
4.31E-05	GO:0040017	positive regulation of locomotion
4.51E-05	GO:0006909	phagocytosis
5.19E-05	GO:0048870	cell motility
5.19E-05	GO:0051674	localization of cell
5.50E-05	GO:0030097	hemopoiesis
5.59E-05	GO:0002520	immune system development
5.64E-05	GO:0040011	locomotion
5.78E-05	GO:0042116	macrophage activation
6.09E-05	GO:0002699	positive regulation of immune effector process
6.09E-05	GO:0031349	positive regulation of defense response
6.99E-05	GO:0045785	positive regulation of cell adhesion
7.11E-05	GO:0042221	response to chemical
7.53E-05	GO:0019886	antigen processing and presentation of exogenous peptide antigen via MHC class II
8.38E-05	GO:0071621	granulocyte chemotaxis
1.13E-04	GO:0002697	regulation of immune effector process
1.19E-04	GO:0030335	positive regulation of cell migration
1.23E-04	GO:0035556	intracellular signal transduction
1.28E-04	GO:0002449	lymphocyte mediated immunity
1.33E-04	GO:0009617	response to bacterium
1.45E-04	GO:0097530	granulocyte migration

1.50E-04	GO:1901570	fatty acid derivative biosynthetic process
1.50E-04	GO:0046456	icosanoid biosynthetic process
1.52E-04	GO:2000147	positive regulation of cell motility
1.65E-04	GO:0002687	positive regulation of leukocyte migration
1.90E-04	GO:0065007	biological regulation
3.44E-04	GO:0030593	neutrophil chemotaxis
4.01E-04	GO:0002690	positive regulation of leukocyte chemotaxis
4.50E-04	GO:0071216	cellular response to biotic stimulus
4.50E-04	GO:0050851	antigen receptor-mediated signaling pathway
4.66E-04	GO:0006636	unsaturated fatty acid biosynthetic process
4.96E-04	GO:0002455	humoral immune response mediated by circulating immunoglobulin
5.39E-04	GO:1990266	neutrophil migration
8.21E-04	GO:0045582	positive regulation of T cell differentiation
8.95E-04	GO:0002283	neutrophil activation involved in immune response
8.95E-04	GO:0002281	macrophage activation involved in immune response
8.95E-04	GO:0001774	microglial cell activation
1.08E-03	GO:0044699	single-organism process
1.30E-03	GO:0051240	positive regulation of multicellular organismal process
1.33E-03	GO:0006935	chemotaxis
1.33E-03	GO:0042330	taxis
1.38E-03	GO:0002688	regulation of leukocyte chemotaxis
1.45E-03	GO:0071219	cellular response to molecule of bacterial origin
1.49E-03	GO:0002503	peptide antigen assembly with MHC class II protein complex
1.49E-03	GO:0002399	MHC class II protein complex assembly
1.50E-03	GO:0050921	positive regulation of chemotaxis
1.56E-03	GO:0032729	positive regulation of interferon-gamma production
1.65E-03	GO:0002573	myeloid leukocyte differentiation
1.72E-03	GO:0030099	myeloid cell differentiation
1.80E-03	GO:0002685	regulation of leukocyte migration
1.86E-03	GO:0030098	lymphocyte differentiation
2.08E-03	GO:0001773	myeloid dendritic cell activation
2.12E-03	GO:0006928	movement of cell or subcellular component
2.34E-03	GO:0050789	regulation of biological process
2.44E-03	GO:0051239	regulation of multicellular organismal process
2.80E-03	GO:0045621	positive regulation of lymphocyte differentiation
2.87E-03	GO:0044707	single-multicellular organism process
3.13E-03	GO:0044763	single-organism cellular process
3.88E-03	GO:0050729	positive regulation of inflammatory response
4.13E-03	GO:0045059	positive thymic T cell selection
4.18E-03	GO:0080134	regulation of response to stress
4.21E-	GO:0071	cellular response to organic substance

03	310	
4.27E-03	GO:0048520	positive regulation of behavior
4.56E-03	GO:0010033	response to organic substance
4.91E-03	GO:0006897	endocytosis
5.00E-03	GO:0032501	multicellular organismal process
5.01E-03	GO:1902107	positive regulation of leukocyte differentiation
5.29E-03	GO:0002889	regulation of immunoglobulin mediated immune response
5.93E-03	GO:0002396	MHC protein complex assembly
5.93E-03	GO:0002501	peptide antigen assembly with MHC protein complex
6.10E-03	GO:0050764	regulation of phagocytosis
6.16E-03	GO:0002888	positive regulation of myeloid leukocyte mediated immunity
6.16E-03	GO:0050794	regulation of cellular process
6.33E-03	GO:0045619	regulation of lymphocyte differentiation
6.50E-03	GO:0002712	regulation of B cell mediated immunity
7.08E-03	GO:1901568	fatty acid derivative metabolic process
7.08E-03	GO:0006690	icosanoid metabolic process
7.79E-03	GO:1902531	regulation of intracellular signal transduction
7.92E-03	GO:0032602	chemokine production
8.98E-03	GO:0002444	myeloid leukocyte mediated immunity
9.57E-03	GO:0030225	macrophage differentiation
1.01E-02	GO:0045088	regulation of innate immune response
1.13E-02	GO:0050920	regulation of chemotaxis
1.22E-02	GO:0045580	regulation of T cell differentiation
1.35E-02	GO:0002221	pattern recognition receptor signaling pathway
1.37E-02	GO:0032722	positive regulation of chemokine production
1.48E-02	GO:0008284	positive regulation of cell proliferation
1.50E-02	GO:0002758	innate immune response-activating signal transduction
1.62E-02	GO:0031294	lymphocyte costimulation
1.62E-02	GO:0031295	T cell costimulation
1.66E-02	GO:0002891	positive regulation of immunoglobulin mediated immune response
1.66E-02	GO:0002714	positive regulation of B cell mediated immunity
1.81E-02	GO:0032649	regulation of interferon-gamma production
1.86E-02	GO:0033559	unsaturated fatty acid metabolic process
1.91E-02	GO:0045058	T cell selection
1.97E-02	GO:0002218	activation of innate immune response
2.20E-02	GO:0046633	alpha-beta T cell proliferation
2.43E-02	GO:0007162	negative regulation of cell adhesion
2.65E-02	GO:0043085	positive regulation of catalytic activity
2.65E-02	GO:1903708	positive regulation of hemopoiesis
2.93E-02	GO:0045588	positive regulation of gamma-delta T cell differentiation
3.01E-02	GO:0002702	positive regulation of production of molecular mediator of immune response

	3.04E-02	GO:0002705	positive regulation of leukocyte mediated immunity						
	3.14E-02	GO:0030217	T cell differentiation						
	3.17E-02	GO:0009966	regulation of signal transduction						
	3.28E-02	GO:0030334	regulation of cell migration						
	3.37E-02	GO:0007596	blood coagulation						
	3.47E-02	GO:0032755	positive regulation of interleukin-6 production						
	3.47E-02	GO:0046635	positive regulation of alpha-beta T cell activation						
	3.57E-02	GO:0002224	toll-like receptor signaling pathway						
	3.65E-02	GO:0035588	G-protein coupled purinergic receptor signaling pathway						
	3.65E-02	GO:0042119	neutrophil activation						
	3.67E-02	GO:0042060	wound healing						
	3.73E-02	GO:0050817	coagulation						
	3.82E-02	GO:0007599	hemostasis						
	4.06E-02	GO:0032675	regulation of interleukin-6 production						
	4.28E-02	GO:0002703	regulation of leukocyte mediated immunity						
	4.45E-02	GO:0032609	interferon-gamma production						
	4.55E-02	GO:0006956	complement activation						
	4.55E-02	GO:0050853	B cell receptor signaling pathway						
	4.59E-02	GO:0043368	positive T cell selection						
	4.59E-02	GO:0006911	phagocytosis, engulfment						
	4.59E-02	GO:0042742	defense response to bacterium						
	4.72E-02	GO:0040012	regulation of locomotion						
	1.85E-09	GO:0006996	organelle organization	1.43E-13	GO:0044424	intracellular part	6.14E-16	GO:0005515	protein binding
	7.56E-09	GO:0019222	regulation of metabolic process	3.27E-13	GO:0005622	intracellular	1.69E-09	GO:0005488	binding
	1.50E-06	GO:0060255	regulation of macromolecule metabolic process	6.81E-10	GO:0043229	intracellular organelle	7.16E-07	GO:0003674	molecular_function
	1.95E-06	GO:1902589	single-organism organelle organization	2.74E-08	GO:0043226	organelle	6.69E-04	GO:0044877	macromolecular complex binding
	3.51E-06	GO:0016043	cellular component organization	3.20E-07	GO:0043231	intracellular membrane-bounded organelle	2.35E-03	GO:0008092	cytoskeletal protein binding
	4.25E-06	GO:0008152	metabolic process	1.22E-06	GO:0043227	membrane-bounded organelle	2.36E-03	GO:0019899	enzyme binding
	4.61E-06	GO:0008150	biological_process	1.55E-06	GO:0005634	nucleus	6.20E-03	GO:0032403	protein complex binding
	6.39E-06	GO:0009987	cellular process	2.80E-06	GO:0005623	cell	3.73E-02	GO:0042802	identical protein binding
	7.24E-06	GO:0071840	cellular component organization or biogenesis	2.80E-06	GO:0044464	cell part	3.89E-02	GO:0003676	nucleic acid binding
blue	1.61E-05	GO:0050794	regulation of cellular process	2.80E-05	GO:0005737	cytoplasm			
	2.02E-05	GO:0006325	chromatin organization	5.32E-05	GO:0070161	anchoring junction			
	2.04E-05	GO:0031323	regulation of cellular metabolic process	9.71E-05	GO:0005912	adherens junction			
	2.44E-05	GO:0006807	nitrogen compound metabolic process	1.80E-04	GO:0030055	cell-substrate junction			
	3.38E-05	GO:0050789	regulation of biological process	2.69E-04	GO:0005925	focal adhesion			
	4.98E-05	GO:0090304	nucleic acid metabolic process	4.61E-04	GO:0005924	cell-substrate adherens junction			
	6.93E-05	GO:0050793	regulation of developmental process	6.97E-04	GO:0044428	nuclear part			
	8.33E-05	GO:0080090	regulation of primary metabolic process	1.15E-03	GO:0005575	cellular_component			
	9.29E-05	GO:0065007	biological regulation	1.21E-03	GO:0044446	intracellular organelle part			
	1.20E-	GO:0044	cellular metabolic process	1.25E-	GO:0043	myelin sheath			

04	237	
1.46E-04	GO:0007272	ensheathment of neurons
1.46E-04	GO:0008366	axon ensheathment
1.91E-04	GO:0051276	chromosome organization
2.03E-04	GO:0034641	cellular nitrogen compound metabolic process
2.18E-04	GO:0042552	myelination
2.50E-04	GO:0048869	cellular developmental process
2.92E-04	GO:0010468	regulation of gene expression
3.25E-04	GO:0016568	chromatin modification
4.06E-04	GO:0071704	organic substance metabolic process
4.72E-04	GO:0043170	macromolecule metabolic process
5.21E-04	GO:1901360	organic cyclic compound metabolic process
5.92E-04	GO:0032502	developmental process
6.12E-04	GO:0043933	macromolecular complex subunit organization
6.38E-04	GO:0048523	negative regulation of cellular process
6.92E-04	GO:0048518	positive regulation of biological process
7.40E-04	GO:0046483	heterocycle metabolic process
9.12E-04	GO:2000026	regulation of multicellular organismal development
9.41E-04	GO:0048468	cell development
9.50E-04	GO:0044767	single-organism developmental process
1.07E-03	GO:0044238	primary metabolic process
1.09E-03	GO:0016070	RNA metabolic process
1.10E-03	GO:0006139	nucleobase-containing compound metabolic process
1.17E-03	GO:0048519	negative regulation of biological process
1.29E-03	GO:0048856	anatomical structure development
1.56E-03	GO:0010467	gene expression
1.91E-03	GO:0019228	neuronal action potential
2.01E-03	GO:0006725	cellular aromatic compound metabolic process
2.27E-03	GO:0045595	regulation of cell differentiation
2.57E-03	GO:2000112	regulation of cellular macromolecule biosynthetic process
2.64E-03	GO:0006355	regulation of transcription, DNA-templated
3.61E-03	GO:1903506	regulation of nucleic acid-templated transcription
4.53E-03	GO:0044260	cellular macromolecule metabolic process
6.35E-03	GO:0051252	regulation of RNA metabolic process
6.47E-03	GO:2001141	regulation of RNA biosynthetic process
6.99E-03	GO:0010556	regulation of macromolecule biosynthetic process
7.28E-03	GO:0032774	RNA biosynthetic process
1.13E-02	GO:0030154	cell differentiation
1.19E-02	GO:0006351	transcription, DNA-templated
1.21E-02	GO:0030099	myeloid cell differentiation
1.30E-02	GO:0097659	nucleic acid-templated transcription

03	209	
2.66E-03	GO:0044422	organelle part
7.38E-03	GO:0005654	nucleoplasm
7.99E-03	GO:0031981	nuclear lumen
1.80E-02	GO:0043228	non-membrane-bounded organelle
1.80E-02	GO:0043232	intracellular non-membrane-bounded organelle
2.58E-02	GO:0031974	membrane-enclosed lumen
2.64E-02	GO:0031252	cell leading edge

	1.66E-02	GO:0006950	response to stress						
	1.75E-02	GO:0019219	regulation of nucleobase-containing compound metabolic process						
	3.03E-02	GO:0019226	transmission of nerve impulse						
	3.07E-02	GO:0048731	system development						
	3.23E-02	GO:1902531	regulation of intracellular signal transduction						
	3.57E-02	GO:0007010	cytoskeleton organization						
	3.64E-02	GO:0051171	regulation of nitrogen compound metabolic process						
	3.74E-02	GO:0016570	histone modification						
	4.12E-02	GO:0010001	glial cell differentiation						
	4.51E-02	GO:0033554	cellular response to stress						
	4.77E-02	GO:0009893	positive regulation of metabolic process						
turquoise	2.68E-10	GO:0050907	detection of chemical stimulus involved in sensory perception	5.50E-09	GO:0005615	extracellular space	1.45E-13	GO:0004930	G-protein coupled receptor activity
	4.99E-10	GO:0050911	detection of chemical stimulus involved in sensory perception of smell	7.77E-07	GO:0045095	keratin filament	1.51E-10	GO:0004888	transmembrane signaling receptor activity
	5.17E-09	GO:0051606	detection of stimulus	1.02E-05	GO:0031012	extracellular matrix	1.91E-10	GO:0004872	receptor activity
	6.44E-09	GO:0050906	detection of stimulus involved in sensory perception	1.18E-05	GO:0005578	proteinaceous extracellular matrix	4.99E-10	GO:0004984	olfactory receptor activity
	1.98E-08	GO:0009593	detection of chemical stimulus	2.17E-05	GO:0005581	collagen trimer	1.12E-09	GO:0038023	signaling receptor activity
	1.23E-07	GO:0007606	sensory perception of chemical stimulus	2.44E-04	GO:0005788	endoplasmic reticulum lumen	3.41E-05	GO:0004252	serine-type endopeptidase activity
	1.30E-05	GO:0007600	sensory perception	2.27E-03	GO:0005882	intermediate filament	2.81E-03	GO:0008236	serine-type peptidase activity
	1.94E-05	GO:0007608	sensory perception of smell				4.36E-03	GO:0017171	serine hydrolase activity
	4.29E-03	GO:0007186	G-protein coupled receptor signaling pathway				5.87E-03	GO:0005125	cytokine activity
	7.54E-03	GO:0007586	digestion				2.31E-02	GO:0005044	scavenger receptor activity
	1.07E-02	GO:0042035	regulation of cytokine biosynthetic process				4.06E-02	GO:0005179	hormone activity
	1.07E-02	GO:0044243	multicellular organismal catabolic process						
	1.66E-02	GO:0030574	collagen catabolic process						
	1.89E-02	GO:0006954	inflammatory response						
	2.35E-02	GO:0042089	cytokine biosynthetic process						
	2.48E-02	GO:0042107	cytokine metabolic process						
	2.57E-02	GO:0031424	keratinization						
brown	1.21E-03	GO:0051648	vesicle localization	1.30E-10	GO:0044424	intracellular part	3.98E-02	GO:0017075	syntaxin-1 binding
	1.46E-03	GO:0051603	proteolysis involved in cellular protein catabolic process	4.64E-10	GO:0043231	intracellular membrane-bounded organelle			
	1.53E-03	GO:0043632	modification-dependent macromolecule catabolic process	5.74E-10	GO:0005622	intracellular			
	1.74E-03	GO:0019941	modification-dependent protein catabolic process	3.03E-09	GO:0043229	intracellular organelle			
	3.23E-03	GO:0006511	ubiquitin-dependent protein catabolic process	3.96E-08	GO:0044446	intracellular organelle part			
	3.28E-03	GO:0035308	negative regulation of protein dephosphorylation	6.59E-07	GO:0043227	membrane-bounded organelle			
	4.73E-03	GO:0070647	protein modification by small protein conjugation or removal	6.84E-07	GO:0044422	organelle part			
	6.83E-03	GO:0044257	cellular protein catabolic process	1.10E-06	GO:0005737	cytoplasm			
	7.07E-03	GO:0051650	establishment of vesicle localization	2.86E-06	GO:0031090	organelle membrane			
	1.31E-02	GO:0022406	membrane docking	4.11E-06	GO:0043226	organelle			
	2.73E-02	GO:0048278	vesicle docking	4.80E-06	GO:0005739	mitochondrion			
				2.00E-04	GO:0044444	cytoplasmic part			
				2.79E-	GO:0005	cell			

					04	623				
					2.79E-04	GO:0044464	cell part			
					3.78E-03	GO:0043005	neuron projection			
					7.04E-03	GO:0044429	mitochondrial part			
					7.09E-03	GO:0044456	synapse part			
					7.18E-03	GO:0045202	synapse			
					2.54E-02	GO:0031967	organelle envelope			
					2.99E-02	GO:0005634	nucleus			
					3.19E-02	GO:0031975	envelope			
					3.29E-02	GO:0097060	synaptic membrane			
green		1.51E-05	GO:0007272	ensheathment of neurons	1.34E-07	GO:0043209	myelin sheath	2.03E-02	GO:0008092	cytoskeletal protein binding
		1.51E-05	GO:0008366	axon ensheathment						
		3.10E-05	GO:0042552	myelination						
		5.45E-05	GO:0019228	neuronal action potential						
		6.57E-04	GO:0045665	negative regulation of neuron differentiation						
		8.06E-04	GO:0050768	negative regulation of neurogenesis						
		1.33E-03	GO:0019226	transmission of nerve impulse						
		3.76E-03	GO:0010721	negative regulation of cell development						
		4.64E-03	GO:0051961	negative regulation of nervous system development						
		5.18E-03	GO:0050770	regulation of axonogenesis						
		1.07E-02	GO:0031344	regulation of cell projection organization						
		2.04E-02	GO:0048468	cell development						
		4.48E-02	GO:0035637	multicellular organismal signaling						
		4.85E-02	GO:0050771	negative regulation of axonogenesis						
lightyellow		2.51E-10	GO:0044265	cellular macromolecule catabolic process	6.67E-20	GO:0043231	intracellular membrane-bounded organelle	1.07E-12	GO:0003723	RNA binding
	THAL	5.09E-10	GO:0043632	modification-dependent macromolecule catabolic process	1.43E-19	GO:0005622	intracellular	1.10E-07	GO:0044822	poly(A) RNA binding
		2.77E-09	GO:0019941	modification-dependent protein catabolic process	4.66E-19	GO:0044424	intracellular part	1.46E-04	GO:0005488	binding
		2.96E-09	GO:0051603	proteolysis involved in cellular protein catabolic process	7.23E-19	GO:0043227	membrane-bounded organelle	7.84E-04	GO:0043021	ribonucleoprotein complex binding
		5.41E-09	GO:0044257	cellular protein catabolic process	1.64E-17	GO:0043229	intracellular organelle	1.88E-03	GO:0008135	translation factor activity, nucleic acid binding
		1.21E-08	GO:0006511	ubiquitin-dependent protein catabolic process	9.08E-17	GO:0043226	organelle	4.53E-03	GO:0005515	protein binding
		1.55E-08	GO:0009057	macromolecule catabolic process	4.65E-16	GO:0044446	intracellular organelle part	1.38E-02	GO:0016462	pyrophosphatase activity
		1.26E-07	GO:0006412	translation	8.95E-16	GO:0005737	cytoplasm	1.54E-02	GO:0000166	nucleotide binding
		3.38E-07	GO:1903047	mitotic cell cycle process	2.82E-15	GO:0044422	organelle part	1.54E-02	GO:1901265	nucleoside phosphate binding
		5.11E-07	GO:0070647	protein modification by small protein conjugation or removal	2.14E-12	GO:0044444	cytoplasmic part	1.60E-02	GO:0016818	hydrolase activity, acting on acid anhydrides, in phosphorus-containing anhydrides
		9.19E-07	GO:0000278	mitotic cell cycle	4.98E-10	GO:0032991	macromolecular complex	1.60E-02	GO:0016817	hydrolase activity, acting on acid anhydrides
		1.45E-06	GO:0030163	protein catabolic process	3.81E-09	GO:0000502	proteasome complex	1.80E-02	GO:0017111	nucleoside-triphosphatase activity
		2.82E-06	GO:0044260	cellular macromolecule metabolic process	1.61E-08	GO:0070013	intracellular organelle lumen	2.73E-02	GO:1901363	heterocyclic compound binding
		3.52E-06	GO:0044238	primary metabolic process	2.04E-08	GO:0044428	nuclear part	4.15E-02	GO:0097159	organic cyclic compound binding
		3.89E-06	GO:0044267	cellular protein metabolic process	2.17E-08	GO:0031974	membrane-enclosed lumen	4.56E-02	GO:0016787	hydrolase activity
		5.63E-06	GO:0044237	cellular metabolic process	4.56E-08	GO:0005634	nucleus			
		7.58E-06	GO:0044772	mitotic cell cycle phase transition	7.46E-08	GO:0043233	organelle lumen			

1.43E-05	GO:0044248	cellular catabolic process
1.50E-05	GO:0022613	ribonucleoprotein complex biogenesis
2.13E-05	GO:1903050	regulation of proteolysis involved in cellular protein catabolic process
2.17E-05	GO:0043170	macromolecule metabolic process
2.35E-05	GO:0044770	cell cycle phase transition
5.11E-05	GO:0022402	cell cycle process
5.38E-05	GO:1903362	regulation of cellular protein catabolic process
6.07E-05	GO:0043161	proteasome-mediated ubiquitin-dependent protein catabolic process
6.47E-05	GO:0008152	metabolic process
6.63E-05	GO:0071704	organic substance metabolic process
6.84E-05	GO:0002478	antigen processing and presentation of exogenous peptide antigen
7.37E-05	GO:0031145	anaphase-promoting complex-dependent proteasomal ubiquitin-dependent protein catabolic process
9.16E-05	GO:0007049	cell cycle
9.73E-05	GO:0010498	proteasomal protein catabolic process
1.28E-04	GO:0031329	regulation of cellular catabolic process
1.43E-04	GO:0019884	antigen processing and presentation of exogenous antigen
1.96E-04	GO:0051352	negative regulation of ligase activity
1.96E-04	GO:0051444	negative regulation of ubiquitin-protein transferase activity
1.96E-04	GO:0051438	regulation of ubiquitin-protein transferase activity
2.18E-04	GO:0051436	negative regulation of ubiquitin-protein ligase activity involved in mitotic cell cycle
2.19E-04	GO:0048002	antigen processing and presentation of peptide antigen
2.23E-04	GO:0019882	antigen processing and presentation
2.26E-04	GO:1901575	organic substance catabolic process
2.28E-04	GO:0006996	organelle organization
2.83E-04	GO:1901990	regulation of mitotic cell cycle phase transition
3.96E-04	GO:0009894	regulation of catabolic process
4.36E-04	GO:0019538	protein metabolic process
4.61E-04	GO:0032446	protein modification by small protein conjugation
5.16E-04	GO:0051340	regulation of ligase activity
6.14E-04	GO:0072431	signal transduction involved in mitotic G1 DNA damage checkpoint
6.14E-04	GO:1902400	intracellular signal transduction involved in G1 DNA damage checkpoint
6.14E-04	GO:0051437	positive regulation of ubiquitin-protein ligase activity involved in regulation of mitotic cell cycle transition
7.05E-04	GO:0006413	translational initiation
8.33E-04	GO:1901991	negative regulation of mitotic cell cycle phase transition
8.50E-04	GO:0006396	RNA processing
8.78E-04	GO:1901987	regulation of cell cycle phase transition
9.64E-04	GO:0016197	endosomal transport
1.04E-03	GO:0016567	protein ubiquitination
1.09E-03	GO:0009056	catabolic process
1.26E-03	GO:1902403	signal transduction involved in mitotic DNA integrity checkpoint
1.26E-	GO:0072	signal transduction involved in mitotic cell cycle checkpoint

1.25E-07	GO:0031981	nuclear lumen
7.21E-07	GO:0030529	ribonucleoprotein complex
1.54E-06	GO:0005654	nucleoplasm
3.65E-06	GO:0005829	cytosol
1.38E-05	GO:0043234	protein complex
1.49E-05	GO:0005739	mitochondrion
3.93E-05	GO:0031090	organelle membrane
5.44E-05	GO:0044464	cell part
5.44E-05	GO:0005623	cell
1.29E-04	GO:0005730	nucleolus
3.38E-04	GO:0031967	organelle envelope
4.12E-04	GO:0031975	envelope
1.69E-02	GO:0031982	vesicle
1.70E-02	GO:1902494	catalytic complex
2.13E-02	GO:0031988	membrane-bounded vesicle
2.33E-02	GO:1903561	extracellular vesicle
2.33E-02	GO:0070062	extracellular vesicular exosome
2.33E-02	GO:0043230	extracellular organelle
2.33E-02	GO:0065010	extracellular membrane-bounded organelle
2.57E-02	GO:0005740	mitochondrial envelope

03	413	
1.26E-03	GO:1902402	signal transduction involved in mitotic DNA damage checkpoint
1.26E-03	GO:0002479	antigen processing and presentation of exogenous peptide antigen via MHC class I, TAP-dependent
1.26E-03	GO:0031397	negative regulation of protein ubiquitination
1.53E-03	GO:0051443	positive regulation of ubiquitin-protein transferase activity
1.58E-03	GO:0051439	regulation of ubiquitin-protein ligase activity involved in mitotic cell cycle
1.91E-03	GO:1901988	negative regulation of cell cycle phase transition
1.97E-03	GO:0072422	signal transduction involved in DNA damage checkpoint
1.97E-03	GO:0072401	signal transduction involved in DNA integrity checkpoint
2.06E-03	GO:0031396	regulation of protein ubiquitination
2.45E-03	GO:0072395	signal transduction involved in cell cycle checkpoint
2.88E-03	GO:1903321	negative regulation of protein modification by small protein conjugation or removal
3.00E-03	GO:0042176	regulation of protein catabolic process
3.03E-03	GO:0042590	antigen processing and presentation of exogenous peptide antigen via MHC class I
3.08E-03	GO:0006977	DNA damage response, signal transduction by p53 class mediator resulting in cell cycle arrest
3.26E-03	GO:2000045	regulation of G1/S transition of mitotic cell cycle
3.40E-03	GO:0051351	positive regulation of ligase activity
3.84E-03	GO:1903320	regulation of protein modification by small protein conjugation or removal
3.98E-03	GO:0007093	mitotic cell cycle checkpoint
4.02E-03	GO:0046907	intracellular transport
4.29E-03	GO:0010467	gene expression
4.30E-03	GO:0071826	ribonucleoprotein complex subunit organization
4.32E-03	GO:1902806	regulation of cell cycle G1/S phase transition
4.57E-03	GO:0031571	mitotic G1 DNA damage checkpoint
4.84E-03	GO:0010948	negative regulation of cell cycle process
5.58E-03	GO:0044783	G1 DNA damage checkpoint
5.58E-03	GO:0044819	mitotic G1/S transition checkpoint
5.95E-03	GO:2000058	regulation of protein ubiquitination involved in ubiquitin-dependent protein catabolic process
7.75E-03	GO:0007067	mitotic nuclear division
7.88E-03	GO:0044774	mitotic DNA integrity checkpoint
8.22E-03	GO:2000060	positive regulation of protein ubiquitination involved in ubiquitin-dependent protein catabolic process
9.82E-03	GO:0002474	antigen processing and presentation of peptide antigen via MHC class I
9.82E-03	GO:1902807	negative regulation of cell cycle G1/S phase transition
9.82E-03	GO:2000134	negative regulation of G1/S transition of mitotic cell cycle
1.06E-02	GO:0071840	cellular component organization or biogenesis
1.36E-02	GO:0008150	biological_process
1.57E-02	GO:0044773	mitotic DNA damage checkpoint
1.70E-02	GO:0000075	cell cycle checkpoint
2.11E-02	GO:0031570	DNA integrity checkpoint
2.13E-02	GO:0022618	ribonucleoprotein complex assembly
2.47E-02	GO:0032543	mitochondrial translation

		2.54E-02	GO:0033554	cellular response to stress						
		2.84E-02	GO:0007005	mitochondrion organization						
		2.92E-02	GO:0000082	G1/S transition of mitotic cell cycle						
		3.03E-02	GO:0007346	regulation of mitotic cell cycle						
		3.17E-02	GO:1902582	single-organism intracellular transport						
		3.22E-02	GO:0000077	DNA damage checkpoint						
		3.32E-02	GO:0006886	intracellular protein transport						
		3.47E-02	GO:0044843	cell cycle G1/S phase transition						
		3.77E-02	GO:0000280	nuclear division						
		3.90E-02	GO:0031398	positive regulation of protein ubiquitination						
		4.04E-02	GO:0048285	organelle fission						
		4.20E-02	GO:0070727	cellular macromolecule localization						
		4.48E-02	GO:0000209	protein polyubiquitination						
		4.54E-02	GO:0042254	ribosome biogenesis						
		4.55E-02	GO:0044403	symbiosis, encompassing mutualism through parasitism						
		4.55E-02	GO:0044419	interspecies interaction between organisms						
		4.73E-02	GO:0015031	protein transport						
		4.85E-02	GO:0007050	cell cycle arrest						
		4.99E-02	GO:0006508	proteolysis						
greenyellow	HIPP	2.12E-03	GO:0000375	RNA splicing, via transesterification reactions	9.83E-11	GO:0005622	intracellular	1.87E-02	GO:0003676	nucleic acid binding
		1.70E-03	GO:0000377	RNA splicing, via transesterification reactions with bulged adenosine as nucleophile	3.56E-03	GO:0005623	cell	2.79E-03	GO:0003723	RNA binding
		1.70E-03	GO:0000398	mRNA splicing, via spliceosome	8.09E-06	GO:0005634	nucleus	2.81E-04	GO:0044822	poly(A) RNA binding
		2.00E-03	GO:0006396	RNA processing	3.65E-06	GO:0005654	nucleoplasm	1.90E-02	GO:0097159	organic cyclic compound binding
		3.22E-02	GO:0006418	tRNA aminoacylation for protein translation	1.60E-03	GO:0005737	cytoplasm	1.42E-02	GO:1901363	heterocyclic compound binding
		3.99E-02	GO:0010467	gene expression	1.97E-06	GO:0031974	membrane-enclosed lumen			
		2.08E-02	GO:0022613	ribonucleoprotein complex biogenesis	2.68E-06	GO:0031981	nuclear lumen			
		4.21E-02	GO:0022618	ribonucleoprotein complex assembly	5.66E-08	GO:0043226	organelle			
		1.22E-02	GO:0034660	ncRNA metabolic process	5.64E-10	GO:0043227	membrane-bounded organelle			
		4.03E-03	GO:0043038	amino acid activation	9.79E-10	GO:0043229	intracellular organelle			
		4.03E-03	GO:0043039	tRNA aminoacylation	1.72E-12	GO:0043231	intracellular membrane-bounded organelle			
		7.18E-03	GO:0044260	cellular macromolecule metabolic process	7.44E-07	GO:0043233	organelle lumen			
					2.68E-11	GO:0044422	organelle part			
					1.28E-10	GO:0044424	intracellular part			
					3.10E-08	GO:0044428	nuclear part			
					1.82E-05	GO:0044444	cytoplasmic part			
					4.30E-12	GO:0044446	intracellular organelle part			
					3.56E-03	GO:0044464	cell part			
					2.73E-07	GO:0070013	intracellular organelle lumen			
					1.01E-02	GO:1990234	transferase complex			
darkgrey		3.48E-39	GO:0002376	immune system process	1.06E-13	GO:0071944	cell periphery	4.88E-11	GO:0004872	receptor activity
		4.64E-	GO:0006	immune response	9.29E-	GO:0005	plasma membrane	2.04E-	GO:0038	signaling receptor activity

37	955	
1.17E-34	GO:0006952	defense response
1.11E-29	GO:0002684	positive regulation of immune system process
1.04E-27	GO:0002682	regulation of immune system process
4.25E-27	GO:0001775	cell activation
4.87E-27	GO:0050778	positive regulation of immune response
6.90E-26	GO:0045087	innate immune response
4.37E-24	GO:0050776	regulation of immune response
4.62E-24	GO:0045321	leukocyte activation
2.53E-23	GO:0002253	activation of immune response
1.07E-21	GO:0002252	immune effector process
5.88E-19	GO:0006950	response to stress
8.71E-19	GO:0002757	immune response-activating signal transduction
5.91E-18	GO:0046651	lymphocyte proliferation
6.88E-18	GO:0032943	mononuclear cell proliferation
2.60E-17	GO:0070661	leukocyte proliferation
5.79E-17	GO:0048584	positive regulation of response to stimulus
2.53E-16	GO:0002764	immune response-regulating signaling pathway
7.57E-16	GO:0050896	response to stimulus
1.62E-15	GO:0046649	lymphocyte activation
1.88E-15	GO:0006954	inflammatory response
1.92E-15	GO:0050865	regulation of cell activation
2.08E-15	GO:0002694	regulation of leukocyte activation
5.36E-15	GO:0002274	myeloid leukocyte activation
7.11E-15	GO:0050670	regulation of lymphocyte proliferation
8.39E-15	GO:0032944	regulation of mononuclear cell proliferation
1.88E-14	GO:0070663	regulation of leukocyte proliferation
2.31E-14	GO:0050867	positive regulation of cell activation
4.57E-14	GO:0001816	cytokine production
7.56E-14	GO:0048518	positive regulation of biological process
9.68E-14	GO:0002696	positive regulation of leukocyte activation
4.13E-13	GO:0051249	regulation of lymphocyte activation
9.04E-13	GO:0009605	response to external stimulus
2.38E-12	GO:0043207	response to external biotic stimulus
2.38E-12	GO:0051707	response to other organism
7.76E-12	GO:0048583	regulation of response to stimulus
1.10E-11	GO:0009607	response to biotic stimulus
1.41E-11	GO:0007165	signal transduction
1.45E-11	GO:0007166	cell surface receptor signaling pathway
1.59E-11	GO:0002521	leukocyte differentiation
1.89E-11	GO:0051251	positive regulation of lymphocyte activation

13	886	
1.09E-10	GO:0016021	integral component of membrane
1.63E-10	GO:0031224	intrinsic component of membrane
1.19E-09	GO:0016020	membrane
5.73E-09	GO:0044425	membrane part
5.20E-08	GO:0044459	plasma membrane part
5.71E-07	GO:0031982	vesicle
8.63E-07	GO:0000323	lytic vacuole
8.63E-07	GO:0005764	lysosome
8.97E-07	GO:0031988	membrane-bounded vesicle
1.15E-05	GO:0005773	vacuole
8.07E-05	GO:0005575	cellular_component
8.25E-05	GO:0005887	integral component of plasma membrane
9.55E-05	GO:0042613	MHC class II protein complex
2.17E-04	GO:0031226	intrinsic component of plasma membrane
2.24E-04	GO:0009986	cell surface
9.67E-04	GO:0005576	extracellular region
1.41E-03	GO:0098552	side of membrane
1.52E-03	GO:0030139	endocytic vesicle
2.48E-03	GO:0030666	endocytic vesicle membrane
4.31E-03	GO:0042611	MHC protein complex
4.92E-03	GO:0009897	external side of plasma membrane
8.79E-03	GO:1903561	extracellular vesicle
8.79E-03	GO:0070062	extracellular vesicular exosome
8.79E-03	GO:0043230	extracellular organelle
8.79E-03	GO:0065010	extracellular membrane-bounded organelle
1.39E-02	GO:0043235	receptor complex
1.90E-02	GO:0032009	early phagosome
2.10E-02	GO:0044437	vacuolar part
3.32E-02	GO:0044421	extracellular region part
4.23E-02	GO:0045335	phagocytic vesicle
4.32E-02	GO:0005765	lysosomal membrane
4.61E-02	GO:0044433	cytoplasmic vesicle part

09	023	
2.35E-09	GO:0060089	molecular transducer activity
2.35E-09	GO:0004871	signal transducer activity
3.32E-08	GO:0004888	transmembrane signaling receptor activity
9.07E-04	GO:0019864	IgG binding
1.77E-03	GO:0003823	antigen binding
5.78E-03	GO:0005515	protein binding
1.05E-02	GO:0035586	purinergic receptor activity
1.05E-02	GO:0001948	glycoprotein binding
1.08E-02	GO:0032403	protein complex binding
1.22E-02	GO:0045028	G-protein coupled purinergic nucleotide receptor activity
1.22E-02	GO:0001608	G-protein coupled nucleotide receptor activity
2.19E-02	GO:0004896	cytokine receptor activity
2.81E-02	GO:0008329	signaling pattern recognition receptor activity
2.81E-02	GO:0038187	pattern recognition receptor activity
4.02E-02	GO:0023026	MHC class II protein complex binding

2.46E-11	GO:0050900	leukocyte migration
2.77E-11	GO:0051716	cellular response to stimulus
3.36E-11	GO:0001817	regulation of cytokine production
6.12E-11	GO:0031347	regulation of defense response
6.48E-11	GO:0034097	response to cytokine
6.52E-11	GO:0042098	T cell proliferation
7.00E-11	GO:0002429	immune response-activating cell surface receptor signaling pathway
8.37E-11	GO:0071345	cellular response to cytokine stimulus
1.08E-10	GO:0002443	leukocyte mediated immunity
1.47E-10	GO:0070887	cellular response to chemical stimulus
1.53E-10	GO:0002366	leukocyte activation involved in immune response
1.53E-10	GO:0002263	cell activation involved in immune response
2.06E-10	GO:0002250	adaptive immune response
3.52E-10	GO:0009611	response to wounding
3.81E-10	GO:0048534	hematopoietic or lymphoid organ development
5.86E-10	GO:0034109	homotypic cell-cell adhesion
5.87E-10	GO:0030097	hemopoiesis
6.30E-10	GO:0044700	single organism signaling
6.82E-10	GO:0023052	signaling
1.10E-09	GO:0007159	leukocyte cell-cell adhesion
1.24E-09	GO:0019221	cytokine-mediated signaling pathway
1.45E-09	GO:0002520	immune system development
1.56E-09	GO:0071216	cellular response to biotic stimulus
1.90E-09	GO:0007154	cell communication
4.88E-09	GO:0002768	immune response-regulating cell surface receptor signaling pathway
5.88E-09	GO:0050864	regulation of B cell activation
7.73E-09	GO:0016337	single organismal cell-cell adhesion
8.39E-09	GO:0070489	T cell aggregation
8.39E-09	GO:0042110	T cell activation
8.97E-09	GO:0071593	lymphocyte aggregation
9.01E-09	GO:0002697	regulation of immune effector process
1.03E-08	GO:0031349	positive regulation of defense response
1.05E-08	GO:0007155	cell adhesion
1.06E-08	GO:0034341	response to interferon-gamma
1.15E-08	GO:0022610	biological adhesion
1.17E-08	GO:0070486	leukocyte aggregation
1.24E-08	GO:0009617	response to bacterium
1.61E-08	GO:0006959	humoral immune response
1.80E-08	GO:0098542	defense response to other organism
1.88E-08	GO:0050671	positive regulation of lymphocyte proliferation
1.97E-	GO:0050	antigen receptor-mediated signaling pathway

08	851			
2.04E-08	GO:0042221	response to chemical		
2.07E-08	GO:0019724	B cell mediated immunity		
2.20E-08	GO:0032946	positive regulation of mononuclear cell proliferation		
3.21E-08	GO:0071219	cellular response to molecule of bacterial origin		
3.50E-08	GO:0070665	positive regulation of leukocyte proliferation		
3.63E-08	GO:0002275	myeloid cell activation involved in immune response		
4.05E-08	GO:0098602	single organism cell adhesion		
8.19E-08	GO:0065007	biological regulation		
8.45E-08	GO:0042113	B cell activation		
1.45E-07	GO:0002699	positive regulation of immune effector process		
1.51E-07	GO:0002237	response to molecule of bacterial origin		
1.78E-07	GO:0060333	interferon-gamma-mediated signaling pathway		
1.81E-07	GO:0032101	regulation of response to external stimulus		
1.99E-07	GO:0050878	regulation of body fluid levels		
2.01E-07	GO:0032103	positive regulation of response to external stimulus		
2.53E-07	GO:0016064	immunoglobulin mediated immune response		
2.54E-07	GO:0002460	adaptive immune response based on somatic recombination of immune receptors built from immunoglobulin superfamily domains		
2.81E-07	GO:0071346	cellular response to interferon-gamma		
2.81E-07	GO:0097529	myeloid leukocyte migration		
3.11E-07	GO:0001819	positive regulation of cytokine production		
3.13E-07	GO:0050863	regulation of T cell activation		
4.33E-07	GO:1903037	regulation of leukocyte cell-cell adhesion		
5.68E-07	GO:0042129	regulation of T cell proliferation		
6.61E-07	GO:0030099	myeloid cell differentiation		
6.71E-07	GO:0006909	phagocytosis		
7.36E-07	GO:0071310	cellular response to organic substance		
7.42E-07	GO:0097530	granulocyte migration		
7.51E-07	GO:0034110	regulation of homotypic cell-cell adhesion		
7.90E-07	GO:0007599	hemostasis		
8.18E-07	GO:0051240	positive regulation of multicellular organismal process		
9.04E-07	GO:0051239	regulation of multicellular organismal process		
1.02E-06	GO:0042060	wound healing		
1.13E-06	GO:0002449	lymphocyte mediated immunity		
1.33E-06	GO:0050871	positive regulation of B cell activation		
1.37E-06	GO:0045088	regulation of innate immune response		
1.43E-06	GO:0044763	single-organism cellular process		
1.68E-06	GO:0002573	myeloid leukocyte differentiation		
1.80E-06	GO:0002683	negative regulation of immune system process		
1.85E-06	GO:0050870	positive regulation of T cell activation		
1.86E-06	GO:0050789	regulation of biological process		

2.03E-06	GO:1903039	positive regulation of leukocyte cell-cell adhesion		
2.06E-06	GO:0071222	cellular response to lipopolysaccharide		
2.24E-06	GO:0034112	positive regulation of homotypic cell-cell adhesion		
2.38E-06	GO:0050853	B cell receptor signaling pathway		
3.19E-06	GO:0007596	blood coagulation		
3.31E-06	GO:0030595	leukocyte chemotaxis		
3.81E-06	GO:0050817	coagulation		
4.01E-06	GO:0050794	regulation of cellular process		
4.60E-06	GO:0032496	response to lipopolysaccharide		
4.73E-06	GO:0022407	regulation of cell-cell adhesion		
5.24E-06	GO:0040011	locomotion		
6.40E-06	GO:0071621	granulocyte chemotaxis		
6.47E-06	GO:0010033	response to organic substance		
1.01E-05	GO:0080134	regulation of response to stress		
1.29E-05	GO:1902105	regulation of leukocyte differentiation		
1.32E-05	GO:1903706	regulation of hemopoiesis		
1.34E-05	GO:0044699	single-organism process		
1.42E-05	GO:0002221	pattern recognition receptor signaling pathway		
1.43E-05	GO:0048522	positive regulation of cellular process		
1.47E-05	GO:0030593	neutrophil chemotaxis		
1.64E-05	GO:0022409	positive regulation of cell-cell adhesion		
1.70E-05	GO:0002758	innate immune response-activating signal transduction		
1.80E-05	GO:0030888	regulation of B cell proliferation		
1.85E-05	GO:0060326	cell chemotaxis		
2.03E-05	GO:0002224	toll-like receptor signaling pathway		
2.19E-05	GO:0002444	myeloid leukocyte mediated immunity		
2.61E-05	GO:0002218	activation of innate immune response		
2.65E-05	GO:1990266	neutrophil migration		
2.67E-05	GO:0030155	regulation of cell adhesion		
4.33E-05	GO:0016477	cell migration		
5.30E-05	GO:0030168	platelet activation		
7.01E-05	GO:0045576	mast cell activation		
7.46E-05	GO:0006897	endocytosis		
7.52E-05	GO:0002703	regulation of leukocyte mediated immunity		
8.51E-05	GO:0051704	multi-organism process		
9.40E-05	GO:0030098	lymphocyte differentiation		
9.42E-05	GO:0002695	negative regulation of leukocyte activation		
1.06E-04	GO:0042100	B cell proliferation		
1.20E-04	GO:0030225	macrophage differentiation		
1.30E-04	GO:0043299	leukocyte degranulation		
1.31E-	GO:0050	T cell receptor signaling pathway		

04	852	
1.45E-04	GO:0042102	positive regulation of T cell proliferation
1.68E-04	GO:0044707	single-multicellular organism process
1.97E-04	GO:0048870	cell motility
1.97E-04	GO:0051674	localization of cell
2.02E-04	GO:0043303	mast cell degranulation
2.02E-04	GO:0002279	mast cell activation involved in immune response
2.12E-04	GO:0051250	negative regulation of lymphocyte activation
2.12E-04	GO:0002495	antigen processing and presentation of peptide antigen via MHC class II
2.30E-04	GO:0050672	negative regulation of lymphocyte proliferation
2.30E-04	GO:0032945	negative regulation of mononuclear cell proliferation
2.37E-04	GO:0006911	phagocytosis, engulfment
2.39E-04	GO:0002504	antigen processing and presentation of peptide or polysaccharide antigen via MHC class II
2.40E-04	GO:0045089	positive regulation of innate immune response
2.90E-04	GO:0051607	defense response to virus
3.26E-04	GO:0050866	negative regulation of cell activation
3.26E-04	GO:0002448	mast cell mediated immunity
3.89E-04	GO:0070664	negative regulation of leukocyte proliferation
3.90E-04	GO:0048002	antigen processing and presentation of peptide antigen
4.65E-04	GO:0001773	myeloid dendritic cell activation
4.95E-04	GO:0032501	multicellular organismal process
5.81E-04	GO:0002888	positive regulation of myeloid leukocyte mediated immunity
6.84E-04	GO:0006935	chemotaxis
6.84E-04	GO:0042330	taxis
7.66E-04	GO:0002478	antigen processing and presentation of exogenous peptide antigen
7.72E-04	GO:0008283	cell proliferation
7.74E-04	GO:0042116	macrophage activation
7.74E-04	GO:0032418	lysosome localization
8.04E-04	GO:0016192	vesicle-mediated transport
8.46E-04	GO:0002886	regulation of myeloid leukocyte mediated immunity
1.05E-03	GO:0042127	regulation of cell proliferation
1.05E-03	GO:1903708	positive regulation of hemopoiesis
1.11E-03	GO:0019884	antigen processing and presentation of exogenous antigen
1.12E-03	GO:0045577	regulation of B cell differentiation
1.21E-03	GO:0042089	cytokine biosynthetic process
1.52E-03	GO:0042107	cytokine metabolic process
1.61E-03	GO:0035556	intracellular signal transduction
1.77E-03	GO:0002700	regulation of production of molecular mediator of immune response
1.85E-03	GO:0045785	positive regulation of cell adhesion
1.87E-03	GO:0030851	granulocyte differentiation
1.87E-03	GO:0002712	regulation of B cell mediated immunity

1.89E-03	GO:0019886	antigen processing and presentation of exogenous peptide antigen via MHC class II
2.02E-03	GO:0045055	regulated secretory pathway
2.29E-03	GO:0008284	positive regulation of cell proliferation
2.32E-03	GO:0009615	response to virus
2.37E-03	GO:0010324	membrane invagination
2.38E-03	GO:0065008	regulation of biological quality
2.60E-03	GO:0030183	B cell differentiation
2.97E-03	GO:0019882	antigen processing and presentation
2.98E-03	GO:0045621	positive regulation of lymphocyte differentiation
2.99E-03	GO:0002532	production of molecular mediator involved in inflammatory response
3.81E-03	GO:0050764	regulation of phagocytosis
4.15E-03	GO:0046645	positive regulation of gamma-delta T cell activation
4.15E-03	GO:0002283	neutrophil activation involved in immune response
4.30E-03	GO:0050729	positive regulation of inflammatory response
4.44E-03	GO:0046634	regulation of alpha-beta T cell activation
4.61E-03	GO:0030890	positive regulation of B cell proliferation
4.61E-03	GO:0002455	humoral immune response mediated by circulating immunoglobulin
4.61E-03	GO:0097028	dendritic cell differentiation
4.79E-03	GO:0002399	MHC class II protein complex assembly
4.79E-03	GO:0002238	response to molecule of fungal origin
4.79E-03	GO:0002503	peptide antigen assembly with MHC class II protein complex
5.06E-03	GO:0032940	secretion by cell
5.21E-03	GO:0006887	exocytosis
5.90E-03	GO:0042119	neutrophil activation
5.90E-03	GO:0035588	G-protein coupled purinergic receptor signaling pathway
5.98E-03	GO:0061515	myeloid cell development
6.09E-03	GO:0042035	regulation of cytokine biosynthetic process
6.38E-03	GO:0046903	secretion
6.88E-03	GO:0045058	T cell selection
7.92E-03	GO:0043304	regulation of mast cell degranulation
7.92E-03	GO:0033006	regulation of mast cell activation involved in immune response
8.26E-03	GO:0050663	cytokine secretion
8.40E-03	GO:1901701	cellular response to oxygen-containing compound
9.85E-03	GO:1902107	positive regulation of leukocyte differentiation
1.03E-02	GO:0050730	regulation of peptidyl-tyrosine phosphorylation
1.05E-02	GO:0051046	regulation of secretion
1.05E-02	GO:0002576	platelet degranulation
1.19E-02	GO:0050848	regulation of calcium-mediated signaling
1.19E-02	GO:0002702	positive regulation of production of molecular mediator of immune response
1.22E-02	GO:0043306	positive regulation of mast cell degranulation
1.22E-	GO:0035	G-protein coupled purinergic nucleotide receptor signaling pathway

	02	589							
	1.22E-02	GO:0033008	positive regulation of mast cell activation involved in immune response						
	1.27E-02	GO:0045619	regulation of lymphocyte differentiation						
	1.27E-02	GO:0019722	calcium-mediated signaling						
	1.36E-02	GO:0009620	response to fungus						
	1.36E-02	GO:0036230	granulocyte activation						
	1.73E-02	GO:0050777	negative regulation of immune response						
	1.73E-02	GO:0046631	alpha-beta T cell activation						
	1.73E-02	GO:0045637	regulation of myeloid cell differentiation						
	1.84E-02	GO:0050727	regulation of inflammatory response						
	1.90E-02	GO:0046643	regulation of gamma-delta T cell activation						
	1.90E-02	GO:0045059	positive thymic T cell selection						
	1.90E-02	GO:0034154	toll-like receptor 7 signaling pathway						
	1.90E-02	GO:0002396	MHC protein complex assembly						
	1.90E-02	GO:0002501	peptide antigen assembly with MHC protein complex						
	1.90E-02	GO:0050707	regulation of cytokine secretion						
	1.91E-02	GO:1903036	positive regulation of response to wounding						
	1.96E-02	GO:0006956	complement activation						
	2.09E-02	GO:0002819	regulation of adaptive immune response						
	2.30E-02	GO:0070527	platelet aggregation						
	2.45E-02	GO:0002705	positive regulation of leukocyte mediated immunity						
	2.67E-02	GO:0042108	positive regulation of cytokine biosynthetic process						
	2.67E-02	GO:0031663	lipopolysaccharide-mediated signaling pathway						
	2.75E-02	GO:0043300	regulation of leukocyte degranulation						
	2.81E-02	GO:0033005	positive regulation of mast cell activation						
	2.88E-02	GO:1902533	positive regulation of intracellular signal transduction						
	3.10E-02	GO:0046456	icosanoid biosynthetic process						
	3.10E-02	GO:1901570	fatty acid derivative biosynthetic process						
	3.40E-02	GO:0035587	purinergic receptor signaling pathway						
	3.40E-02	GO:0033003	regulation of mast cell activation						
	3.40E-02	GO:0002889	regulation of immunoglobulin mediated immune response						
	3.57E-02	GO:1903034	regulation of response to wounding						
	3.81E-02	GO:0002698	negative regulation of immune effector process						
	3.94E-02	GO:0006928	movement of cell or subcellular component						
	4.02E-02	GO:0045579	positive regulation of B cell differentiation						
	4.02E-02	GO:0043302	positive regulation of leukocyte degranulation						
	4.02E-02	GO:0032660	regulation of interleukin-17 production						
	4.16E-02	GO:0098751	bone cell development						
	4.19E-02	GO:0032844	regulation of homeostatic process						
grey60	2.91E-08	GO:0044238	primary metabolic process	1.18E-12	GO:0043231	intracellular membrane-bounded organelle	1.43E-05	GO:0003676	nucleic acid binding
	1.07E-07	GO:0071704	organic substance metabolic process	1.65E-12	GO:0005634	nucleus	1.30E-02	GO:0003723	RNA binding

1.44E-07	GO:0010467	gene expression
1.53E-07	GO:0008152	metabolic process
3.84E-07	GO:0044260	cellular macromolecule metabolic process
4.91E-07	GO:0044237	cellular metabolic process
2.82E-06	GO:0043170	macromolecule metabolic process
7.97E-06	GO:0016070	RNA metabolic process
9.63E-06	GO:0044249	cellular biosynthetic process
1.78E-05	GO:0034645	cellular macromolecule biosynthetic process
2.02E-05	GO:0009058	biosynthetic process
3.51E-05	GO:0090304	nucleic acid metabolic process
5.27E-05	GO:1901576	organic substance biosynthetic process
6.95E-05	GO:0009059	macromolecule biosynthetic process
1.76E-04	GO:0034641	cellular nitrogen compound metabolic process
2.54E-04	GO:0006139	nucleobase-containing compound metabolic process
2.74E-04	GO:0006351	transcription, DNA-templated
3.63E-04	GO:0097659	nucleic acid-templated transcription
5.08E-04	GO:0006807	nitrogen compound metabolic process
1.13E-03	GO:1901360	organic cyclic compound metabolic process
1.20E-03	GO:0006725	cellular aromatic compound metabolic process
1.46E-03	GO:0032774	RNA biosynthetic process
1.83E-03	GO:0046483	heterocycle metabolic process
2.92E-03	GO:0044271	cellular nitrogen compound biosynthetic process
7.47E-03	GO:0010556	regulation of macromolecule biosynthetic process
7.59E-03	GO:2000112	regulation of cellular macromolecule biosynthetic process
7.68E-03	GO:0010468	regulation of gene expression
8.25E-03	GO:0051171	regulation of nitrogen compound metabolic process
8.71E-03	GO:2001141	regulation of RNA biosynthetic process
8.98E-03	GO:0051252	regulation of RNA metabolic process
9.40E-03	GO:0006355	regulation of transcription, DNA-templated
1.03E-02	GO:1901362	organic cyclic compound biosynthetic process
1.11E-02	GO:0016071	mRNA metabolic process
1.30E-02	GO:1903506	regulation of nucleic acid-templated transcription
1.52E-02	GO:0031326	regulation of cellular biosynthetic process
1.58E-02	GO:0019219	regulation of nucleobase-containing compound metabolic process
2.01E-02	GO:0034654	nucleobase-containing compound biosynthetic process
2.19E-02	GO:1901575	organic substance catabolic process
2.41E-02	GO:0009889	regulation of biosynthetic process
2.51E-02	GO:0018130	heterocycle biosynthetic process
2.65E-02	GO:0019438	aromatic compound biosynthetic process
3.18E-02	GO:0044265	cellular macromolecule catabolic process
3.20E-	GO:0060	regulation of macromolecule metabolic process

5.17E-11	GO:0043229	intracellular organelle
5.21E-09	GO:0043227	membrane-bounded organelle
1.18E-08	GO:0044424	intracellular part
1.85E-08	GO:0005622	intracellular
2.25E-08	GO:0043226	organelle
9.16E-08	GO:0044428	nuclear part
1.83E-06	GO:0044446	intracellular organelle part
2.56E-06	GO:0005654	nucleoplasm
3.00E-06	GO:0031981	nuclear lumen
1.04E-05	GO:0044422	organelle part
1.48E-05	GO:0070013	intracellular organelle lumen
1.64E-05	GO:0043233	organelle lumen
3.70E-05	GO:0031974	membrane-enclosed lumen
6.85E-03	GO:0044451	nucleoplasm part

	02	255							
	3.29E-02	GO:0080090	regulation of primary metabolic process						
	3.81E-02	GO:0031323	regulation of cellular metabolic process						
	3.82E-02	GO:0008150	biological_process						
black	3.61E-07	GO:0044237	cellular metabolic process	1.88E-14	GO:0005622	intracellular	1.08E-03	GO:0003824	catalytic activity
	6.57E-06	GO:0044267	cellular protein metabolic process	1.36E-13	GO:0044424	intracellular part	3.44E-03	GO:0003723	RNA binding
	2.56E-05	GO:0008152	metabolic process	3.05E-13	GO:0044446	intracellular organelle part			
	1.01E-04	GO:0044265	cellular macromolecule catabolic process	2.00E-12	GO:0043231	intracellular membrane-bounded organelle			
	1.55E-04	GO:0070647	protein modification by small protein conjugation or removal	3.58E-12	GO:0044422	organelle part			
	3.49E-04	GO:0043632	modification-dependent macromolecule catabolic process	5.42E-12	GO:0043229	intracellular organelle			
	4.72E-04	GO:0006511	ubiquitin-dependent protein catabolic process	3.42E-11	GO:0044444	cytoplasmic part			
	5.23E-04	GO:0044257	cellular protein catabolic process	9.76E-10	GO:0005739	mitochondrion			
	6.47E-04	GO:0044248	cellular catabolic process	1.96E-09	GO:0005737	cytoplasm			
	6.61E-04	GO:0071704	organic substance metabolic process	8.11E-09	GO:0043227	membrane-bounded organelle			
	7.33E-04	GO:0019941	modification-dependent protein catabolic process	8.59E-09	GO:0044429	mitochondrial part			
	1.01E-03	GO:0046907	intracellular transport	3.74E-08	GO:0043226	organelle			
	1.02E-03	GO:0033554	cellular response to stress	6.27E-07	GO:0031966	mitochondrial membrane			
	1.47E-03	GO:0007005	mitochondrion organization	6.48E-07	GO:0031090	organelle membrane			
	1.61E-03	GO:0051603	proteolysis involved in cellular protein catabolic process	1.42E-06	GO:0005740	mitochondrial envelope			
	1.94E-03	GO:0019538	protein metabolic process	1.94E-06	GO:0031967	organelle envelope			
	3.25E-03	GO:1903047	mitotic cell cycle process	2.34E-06	GO:0031975	envelope			
	3.82E-03	GO:0044260	cellular macromolecule metabolic process	1.12E-05	GO:0032991	macromolecular complex			
	5.08E-03	GO:0070727	cellular macromolecule localization	2.26E-05	GO:0031974	membrane-enclosed lumen			
	5.72E-03	GO:0006996	organelle organization	3.39E-05	GO:0070013	intracellular organelle lumen			
	6.06E-03	GO:0009057	macromolecule catabolic process	7.92E-05	GO:0005743	mitochondrial inner membrane			
	7.64E-03	GO:0045333	cellular respiration	8.96E-05	GO:0043233	organelle lumen			
	8.16E-03	GO:0034613	cellular protein localization	2.26E-04	GO:0000502	proteasome complex			
	8.16E-03	GO:1902582	single-organism intracellular transport	9.10E-04	GO:0019866	organelle inner membrane			
	8.38E-03	GO:0044238	primary metabolic process	2.60E-03	GO:0044428	nuclear part			
	9.13E-03	GO:0016567	protein ubiquitination	2.74E-03	GO:0005634	nucleus			
	9.53E-03	GO:0030163	protein catabolic process	5.71E-03	GO:0043234	protein complex			
	1.02E-02	GO:0008104	protein localization	6.29E-03	GO:0031981	nuclear lumen			
	1.12E-02	GO:0044772	mitotic cell cycle phase transition	1.43E-02	GO:0044464	cell part			
	1.27E-02	GO:0033036	macromolecule localization	1.43E-02	GO:0005623	cell			
	1.33E-02	GO:0043412	macromolecule modification	1.44E-02	GO:0044455	mitochondrial membrane part			
	2.18E-02	GO:0044770	cell cycle phase transition	2.34E-02	GO:1902494	catalytic complex			
	2.85E-02	GO:0009056	catabolic process	4.14E-02	GO:0030529	ribonucleoprotein complex			
	3.09E-02	GO:0000278	mitotic cell cycle						
	3.35E-02	GO:0006412	translation						
	3.81E-02	GO:0015031	protein transport						
	3.89E-02	GO:0006839	mitochondrial transport						

		3.90E-02	GO:0061024	membrane organization						
		4.35E-02	GO:0007006	mitochondrial membrane organization						
		4.38E-02	GO:0008150	biological_process						
		4.52E-02	GO:0032446	protein modification by small protein conjugation						
		4.57E-02	GO:0022613	ribonucleoprotein complex biogenesis						
skyblue		7.11E-09	GO:0016070	RNA metabolic process	3.48E-09	GO:0005634	nucleus	5.47E-07	GO:0003723	RNA binding
		7.55E-09	GO:0006397	mRNA processing	1.43E-07	GO:0044428	nuclear part	1.06E-06	GO:0003676	nucleic acid binding
		2.48E-08	GO:0016071	mRNA metabolic process	2.50E-07	GO:0016607	nuclear speck	1.71E-05	GO:1901363	heterocyclic compound binding
		3.29E-08	GO:0006396	RNA processing	4.01E-07	GO:0031981	nuclear lumen	3.11E-05	GO:0097159	organic cyclic compound binding
		3.83E-08	GO:0010467	gene expression	4.78E-07	GO:0044451	nucleoplasm part	4.36E-05	GO:0044822	poly(A) RNA binding
		5.41E-08	GO:0090304	nucleic acid metabolic process	1.59E-06	GO:0016604	nuclear body			
		1.62E-06	GO:0006139	nucleobase-containing compound metabolic process	5.41E-06	GO:0005654	nucleoplasm			
		2.63E-06	GO:0046483	heterocycle metabolic process	3.64E-05	GO:0070013	intracellular organelle lumen			
		3.04E-06	GO:0006725	cellular aromatic compound metabolic process	6.21E-05	GO:0043233	organelle lumen			
		4.57E-06	GO:0008380	RNA splicing	9.29E-05	GO:0043231	intracellular membrane-bounded organelle			
		6.70E-06	GO:1901360	organic cyclic compound metabolic process	1.04E-04	GO:0031974	membrane-enclosed lumen			
		4.05E-05	GO:0034641	cellular nitrogen compound metabolic process	1.97E-03	GO:0043229	intracellular organelle			
		2.64E-04	GO:0006807	nitrogen compound metabolic process	2.34E-03	GO:0043227	membrane-bounded organelle			
		3.00E-04	GO:0050658	RNA transport	2.30E-02	GO:0005622	intracellular			
		3.00E-04	GO:0051236	establishment of RNA localization	4.23E-02	GO:0043226	organelle			
		3.00E-04	GO:0050657	nucleic acid transport						
		4.65E-04	GO:0044260	cellular macromolecule metabolic process						
		4.86E-04	GO:0008152	metabolic process						
		6.04E-04	GO:0051028	mRNA transport						
		6.42E-04	GO:0006403	RNA localization						
		1.22E-03	GO:0043170	macromolecule metabolic process						
		2.15E-03	GO:0015931	nucleobase-containing compound transport						
		5.10E-03	GO:0071704	organic substance metabolic process						
		6.31E-03	GO:0044237	cellular metabolic process						
		8.36E-03	GO:0044238	primary metabolic process						
		8.77E-03	GO:0010468	regulation of gene expression						
		1.37E-02	GO:0051252	regulation of RNA metabolic process						
		2.06E-02	GO:0006406	mRNA export from nucleus						
		2.55E-02	GO:0019219	regulation of nucleobase-containing compound metabolic process						
black	WH MT	6.54E-70	GO:0002376	immune system process	5.03E-13	GO:0071944	cell periphery	2.70E-10	GO:0005515	protein binding
		2.69E-61	GO:0006955	immune response	2.25E-12	GO:0005886	plasma membrane	2.70E-09	GO:0008047	enzyme activator activity
		2.97E-51	GO:0002682	regulation of immune system process	1.09E-09	GO:0031988	membrane-bounded vesicle	1.18E-08	GO:0005096	GTPase activator activity
		6.00E-50	GO:0006952	defense response	1.87E-09	GO:0031982	vesicle	5.22E-07	GO:0030695	GTPase regulator activity
		6.48E-50	GO:0002684	positive regulation of immune system process	2.93E-09	GO:0098552	side of membrane	8.47E-07	GO:0060089	molecular transducer activity
		4.13E-45	GO:0001775	cell activation	9.20E-09	GO:0000323	lytic vacuole	8.47E-07	GO:0004871	signal transducer activity
		3.45E-	GO:0045	innate immune response	9.20E-	GO:0005	lysosome	1.20E-	GO:0060	nucleoside-triphosphatase regulator activity

44	087	
3.94E-44	GO:0050776	regulation of immune response
3.38E-42	GO:0050778	positive regulation of immune response
1.31E-38	GO:0045321	leukocyte activation
5.15E-38	GO:0002253	activation of immune response
2.70E-36	GO:0048583	regulation of response to stimulus
3.51E-33	GO:0002757	immune response-activating signal transduction
7.76E-33	GO:0006950	response to stress
9.44E-33	GO:0050896	response to stimulus
1.08E-31	GO:0046649	lymphocyte activation
2.26E-31	GO:0002252	immune effector process
2.28E-31	GO:0048584	positive regulation of response to stimulus
2.83E-30	GO:0002764	immune response-regulating signaling pathway
2.87E-30	GO:0043207	response to external biotic stimulus
2.87E-30	GO:0051707	response to other organism
7.52E-30	GO:0048518	positive regulation of biological process
8.69E-30	GO:0007165	signal transduction
2.53E-29	GO:0051716	cellular response to stimulus
3.60E-29	GO:0009607	response to biotic stimulus
1.62E-25	GO:0044700	single organism signaling
2.03E-25	GO:0023052	signaling
1.50E-24	GO:0007154	cell communication
2.02E-23	GO:0031347	regulation of defense response
3.79E-22	GO:0007159	leukocyte cell-cell adhesion
5.38E-22	GO:0050865	regulation of cell activation
5.81E-22	GO:0001816	cytokine production
3.67E-21	GO:0071593	lymphocyte aggregation
7.49E-21	GO:0070486	leukocyte aggregation
9.09E-21	GO:0045088	regulation of innate immune response
9.33E-21	GO:0035556	intracellular signal transduction
9.73E-21	GO:0009617	response to bacterium
1.10E-20	GO:0034109	homotypic cell-cell adhesion
1.17E-20	GO:0009605	response to external stimulus
1.80E-20	GO:0070489	T cell aggregation
1.80E-20	GO:0042110	T cell activation
2.27E-20	GO:0070887	cellular response to chemical stimulus
3.19E-20	GO:0034097	response to cytokine
4.08E-20	GO:0002521	leukocyte differentiation
5.39E-20	GO:0031349	positive regulation of defense response
1.36E-19	GO:0001817	regulation of cytokine production
1.65E-19	GO:0098602	single organism cell adhesion

09	764	
1.13E-07	GO:0009897	external side of plasma membrane
1.18E-07	GO:0044459	plasma membrane part
3.45E-07	GO:0045335	phagocytic vesicle
3.65E-07	GO:0005773	vacuole
4.11E-07	GO:1903561	extracellular vesicle
4.11E-07	GO:0070062	extracellular vesicular exosome
4.11E-07	GO:0043230	extracellular organelle
4.11E-07	GO:0065010	extracellular membrane-bounded organelle
8.52E-07	GO:0016020	membrane
2.56E-06	GO:0030139	endocytic vesicle
4.00E-06	GO:0005576	extracellular region
5.98E-06	GO:0044421	extracellular region part
1.19E-05	GO:0009986	cell surface
2.21E-05	GO:0042613	MHC class II protein complex
2.22E-05	GO:0042611	MHC protein complex
6.83E-05	GO:0000786	nucleosome
6.83E-05	GO:1990104	DNA bending complex
2.88E-04	GO:0030666	endocytic vesicle membrane
3.33E-04	GO:0044815	DNA packaging complex
1.30E-03	GO:0044425	membrane part
1.52E-03	GO:0044444	cytoplasmic part
2.02E-03	GO:0036019	endolysosome
2.04E-03	GO:0031226	intrinsic component of plasma membrane
2.22E-03	GO:0030670	phagocytic vesicle membrane
2.32E-03	GO:0044440	endosomal part
2.79E-03	GO:0005829	cytosol
5.13E-03	GO:0005768	endosome
7.87E-03	GO:0001772	immunological synapse
8.19E-03	GO:0045121	membrane raft
8.63E-03	GO:0005615	extracellular space
1.09E-02	GO:0016021	integral component of membrane
1.13E-02	GO:0005887	integral component of plasma membrane
1.15E-02	GO:0005765	lysosomal membrane
1.15E-02	GO:0031224	intrinsic component of membrane
1.56E-02	GO:0043235	receptor complex
1.81E-02	GO:0010008	endosome membrane
1.93E-02	GO:0044437	vacuolar part
3.31E-02	GO:0031091	platelet alpha granule
3.95E-02	GO:0016023	cytoplasmic membrane-bounded vesicle
4.47E-02	GO:0005774	vacuolar membrane

06	589	
5.53E-06	GO:0004872	receptor activity
2.10E-05	GO:0005488	binding
4.97E-05	GO:0030234	enzyme regulator activity
1.24E-04	GO:0038023	signaling receptor activity
2.09E-04	GO:0005099	Ras GTPase activator activity
3.18E-04	GO:0003823	antigen binding
4.44E-04	GO:0005100	Rho GTPase activator activity
4.93E-04	GO:0030675	Rac GTPase activator activity
1.14E-03	GO:0004888	transmembrane signaling receptor activity
1.31E-03	GO:0019899	enzyme binding
1.38E-03	GO:0005102	receptor binding
1.40E-03	GO:0005083	small GTPase regulator activity
1.61E-03	GO:0046983	protein dimerization activity
2.02E-03	GO:0019864	IgG binding
2.22E-03	GO:0004896	cytokine receptor activity
2.45E-03	GO:0046982	protein heterodimerization activity
3.17E-03	GO:0042802	identical protein binding
4.07E-03	GO:0003674	molecular_function
2.01E-02	GO:0023026	MHC class II protein complex binding
2.23E-02	GO:0032403	protein complex binding
2.51E-02	GO:0017124	SH3 domain binding

2.47E-19	GO:0007166	cell surface receptor signaling pathway
2.93E-19	GO:0002694	regulation of leukocyte activation
3.05E-19	GO:0030097	hemopoiesis
4.34E-19	GO:0006954	inflammatory response
5.45E-19	GO:0002520	immune system development
5.71E-19	GO:0050867	positive regulation of cell activation
6.05E-19	GO:0046651	lymphocyte proliferation
7.07E-19	GO:0016337	single organismal cell-cell adhesion
7.75E-19	GO:0032943	mononuclear cell proliferation
8.93E-19	GO:0070661	leukocyte proliferation
1.33E-18	GO:0051249	regulation of lymphocyte activation
1.51E-18	GO:0048534	hematopoietic or lymphoid organ development
1.39E-17	GO:0002237	response to molecule of bacterial origin
2.75E-17	GO:0002218	activation of innate immune response
3.08E-17	GO:0071310	cellular response to organic substance
3.27E-17	GO:0080134	regulation of response to stress
7.15E-17	GO:0045089	positive regulation of innate immune response
1.41E-16	GO:0002429	immune response-activating cell surface receptor signaling pathway
1.75E-16	GO:0002696	positive regulation of leukocyte activation
2.21E-16	GO:0009611	response to wounding
3.02E-16	GO:0071345	cellular response to cytokine stimulus
3.41E-16	GO:0042113	B cell activation
3.46E-16	GO:0098542	defense response to other organism
5.05E-16	GO:0002758	innate immune response-activating signal transduction
8.86E-16	GO:0034341	response to interferon-gamma
1.15E-15	GO:0002768	immune response-regulating cell surface receptor signaling pathway
1.23E-15	GO:0051251	positive regulation of lymphocyte activation
2.22E-15	GO:0010033	response to organic substance
2.44E-15	GO:0002221	pattern recognition receptor signaling pathway
3.95E-15	GO:1902531	regulation of intracellular signal transduction
4.87E-15	GO:0009966	regulation of signal transduction
6.75E-15	GO:0002274	myeloid leukocyte activation
7.23E-15	GO:0002224	toll-like receptor signaling pathway
8.08E-15	GO:0060333	interferon-gamma-mediated signaling pathway
9.56E-15	GO:0071346	cellular response to interferon-gamma
9.62E-15	GO:0050900	leukocyte migration
1.12E-14	GO:0042221	response to chemical
1.31E-14	GO:0019221	cytokine-mediated signaling pathway
1.54E-14	GO:0002443	leukocyte mediated immunity
2.40E-14	GO:0002250	adaptive immune response
2.40E-	GO:0032	response to lipopolysaccharide

14	496	
5.60E-14	GO:0007155	cell adhesion
6.84E-14	GO:0022610	biological adhesion
1.03E-13	GO:0071216	cellular response to biotic stimulus
1.39E-13	GO:0044093	positive regulation of molecular function
2.98E-13	GO:0042098	T cell proliferation
3.75E-13	GO:0001819	positive regulation of cytokine production
3.88E-13	GO:0009893	positive regulation of metabolic process
4.03E-13	GO:1903037	regulation of leukocyte cell-cell adhesion
4.06E-13	GO:0051240	positive regulation of multicellular organismal process
4.14E-13	GO:0070663	regulation of leukocyte proliferation
4.21E-13	GO:0030098	lymphocyte differentiation
4.48E-13	GO:0002460	adaptive immune response based on somatic recombination of immune receptors built from immunoglobulin superfamily domains
6.20E-13	GO:0023051	regulation of signaling
7.48E-13	GO:0050670	regulation of lymphocyte proliferation
8.60E-13	GO:0071219	cellular response to molecule of bacterial origin
9.47E-13	GO:0032944	regulation of mononuclear cell proliferation
9.96E-13	GO:0010646	regulation of cell communication
1.14E-12	GO:0050863	regulation of T cell activation
1.20E-12	GO:0002683	negative regulation of immune system process
1.31E-12	GO:0065007	biological regulation
1.39E-12	GO:0034110	regulation of homotypic cell-cell adhesion
2.89E-12	GO:0048522	positive regulation of cellular process
4.68E-12	GO:0043085	positive regulation of catalytic activity
6.25E-12	GO:0050851	antigen receptor-mediated signaling pathway
6.99E-12	GO:0050789	regulation of biological process
9.99E-12	GO:0051239	regulation of multicellular organismal process
1.38E-11	GO:0030155	regulation of cell adhesion
1.80E-11	GO:0042060	wound healing
2.23E-11	GO:0007599	hemostasis
2.42E-11	GO:0006909	phagocytosis
2.56E-11	GO:0002449	lymphocyte mediated immunity
3.28E-11	GO:0050794	regulation of cellular process
4.73E-11	GO:0051704	multi-organism process
4.84E-11	GO:0007596	blood coagulation
5.24E-11	GO:0022407	regulation of cell-cell adhesion
7.13E-11	GO:0002366	leukocyte activation involved in immune response
7.13E-11	GO:0002263	cell activation involved in immune response
7.18E-11	GO:0050817	coagulation
7.24E-11	GO:0050870	positive regulation of T cell activation
7.71E-11	GO:0071222	cellular response to lipopolysaccharide

8.86E-11	GO:1903039	positive regulation of leukocyte cell-cell adhesion	
1.05E-10	GO:0009615	response to virus	
1.08E-10	GO:0034112	positive regulation of homotypic cell-cell adhesion	
1.31E-10	GO:0051345	positive regulation of hydrolase activity	
1.54E-10	GO:0002697	regulation of immune effector process	
3.47E-10	GO:0065009	regulation of molecular function	
3.89E-10	GO:0030168	platelet activation	
4.97E-10	GO:0050878	regulation of body fluid levels	
5.53E-10	GO:0060326	cell chemotaxis	
6.13E-10	GO:0044763	single-organism cellular process	
8.74E-10	GO:0032101	regulation of response to external stimulus	
1.14E-09	GO:0050790	regulation of catalytic activity	
2.45E-09	GO:0051607	defense response to virus	
3.97E-09	GO:0030099	myeloid cell differentiation	
4.32E-09	GO:0051336	regulation of hydrolase activity	
5.65E-09	GO:0043087	regulation of GTPase activity	
5.92E-09	GO:1903706	regulation of hemopoiesis	
6.88E-09	GO:0022409	positive regulation of cell-cell adhesion	
7.65E-09	GO:0043547	positive regulation of GTPase activity	
7.74E-09	GO:0008219	cell death	
9.26E-09	GO:0016265	death	
1.12E-08	GO:0030595	leukocyte chemotaxis	
1.12E-08	GO:0002755	MyD88-dependent toll-like receptor signaling pathway	
1.15E-08	GO:0016477	cell migration	
1.16E-08	GO:0012501	programmed cell death	
1.20E-08	GO:0044707	single-multicellular organism process	
1.85E-08	GO:0070665	positive regulation of leukocyte proliferation	
2.16E-08	GO:0045785	positive regulation of cell adhesion	
2.91E-08	GO:0006915	apoptotic process	
5.31E-08	GO:0050671	positive regulation of lymphocyte proliferation	
5.31E-08	GO:0097529	myeloid leukocyte migration	
5.60E-08	GO:0019724	B cell mediated immunity	
5.66E-08	GO:0051250	negative regulation of lymphocyte activation	
6.12E-08	GO:0050866	negative regulation of cell activation	
6.74E-08	GO:0032946	positive regulation of mononuclear cell proliferation	
7.30E-08	GO:0050864	regulation of B cell activation	
7.30E-08	GO:0030183	B cell differentiation	
7.81E-08	GO:0044699	single-organism process	
1.13E-07	GO:1902105	regulation of leukocyte differentiation	
1.35E-07	GO:0040011	locomotion	
1.53E-	GO:0032	positive regulation of response to external stimulus	

07	103	
1.80E-07	GO:0032501	multicellular organismal process
2.11E-07	GO:0002695	negative regulation of leukocyte activation
2.74E-07	GO:1902533	positive regulation of intracellular signal transduction
2.77E-07	GO:0016064	immunoglobulin mediated immune response
3.00E-07	GO:0048870	cell motility
3.00E-07	GO:0051674	localization of cell
3.55E-07	GO:0043067	regulation of programmed cell death
5.06E-07	GO:0008283	cell proliferation
5.14E-07	GO:0042981	regulation of apoptotic process
5.42E-07	GO:0010941	regulation of cell death
6.45E-07	GO:0009967	positive regulation of signal transduction
6.84E-07	GO:0050853	B cell receptor signaling pathway
6.98E-07	GO:0048519	negative regulation of biological process
1.15E-06	GO:0042129	regulation of T cell proliferation
1.46E-06	GO:0006897	endocytosis
1.51E-06	GO:0042108	positive regulation of cytokine biosynthetic process
1.51E-06	GO:0031663	lipopolysaccharide-mediated signaling pathway
1.68E-06	GO:0038093	Fc receptor signaling pathway
2.05E-06	GO:0002573	myeloid leukocyte differentiation
2.14E-06	GO:0034142	toll-like receptor 4 signaling pathway
2.16E-06	GO:0006959	humoral immune response
2.61E-06	GO:0050852	T cell receptor signaling pathway
3.15E-06	GO:0023056	positive regulation of signaling
3.17E-06	GO:0050871	positive regulation of B cell activation
3.69E-06	GO:0097190	apoptotic signaling pathway
3.74E-06	GO:0042100	B cell proliferation
5.83E-06	GO:0042089	cytokine biosynthetic process
6.07E-06	GO:0002703	regulation of leukocyte mediated immunity
6.30E-06	GO:0050793	regulation of developmental process
6.53E-06	GO:0042102	positive regulation of T cell proliferation
6.75E-06	GO:0097191	extrinsic apoptotic signaling pathway
6.81E-06	GO:0002699	positive regulation of immune effector process
7.84E-06	GO:0007249	I-kappaB kinase/NF-kappaB signaling
8.23E-06	GO:0042035	regulation of cytokine biosynthetic process
9.15E-06	GO:0010647	positive regulation of cell communication
9.18E-06	GO:0042107	cytokine metabolic process
1.05E-05	GO:0034134	toll-like receptor 2 signaling pathway
1.13E-05	GO:0065008	regulation of biological quality
1.15E-05	GO:0050777	negative regulation of immune response
1.67E-05	GO:0002275	myeloid cell activation involved in immune response

1.67E-05	GO:0030888	regulation of B cell proliferation
1.90E-05	GO:0009987	cellular process
2.04E-05	GO:0050730	regulation of peptidyl-tyrosine phosphorylation
2.31E-05	GO:1903708	positive regulation of hemopoiesis
2.92E-05	GO:0048513	organ development
3.49E-05	GO:1903034	regulation of response to wounding
3.49E-05	GO:0033993	response to lipid
3.53E-05	GO:0048585	negative regulation of response to stimulus
3.92E-05	GO:0048523	negative regulation of cellular process
4.23E-05	GO:1901700	response to oxygen-containing compound
4.45E-05	GO:0050663	cytokine secretion
4.77E-05	GO:0050727	regulation of inflammatory response
4.81E-05	GO:0042116	macrophage activation
4.89E-05	GO:0051094	positive regulation of developmental process
5.75E-05	GO:1901701	cellular response to oxygen-containing compound
6.39E-05	GO:1902107	positive regulation of leukocyte differentiation
7.56E-05	GO:0002431	Fc receptor mediated stimulatory signaling pathway
7.67E-05	GO:0030217	T cell differentiation
8.70E-05	GO:0050764	regulation of phagocytosis
9.31E-05	GO:0050707	regulation of cytokine secretion
9.63E-05	GO:0051247	positive regulation of protein metabolic process
1.04E-04	GO:0008154	actin polymerization or depolymerization
1.08E-04	GO:0045619	regulation of lymphocyte differentiation
1.10E-04	GO:0045621	positive regulation of lymphocyte differentiation
1.13E-04	GO:0002819	regulation of adaptive immune response
1.23E-04	GO:0051246	regulation of protein metabolic process
1.39E-04	GO:1903555	regulation of tumor necrosis factor superfamily cytokine production
1.44E-04	GO:0002822	regulation of adaptive immune response based on somatic recombination of immune receptors built from immunoglobulin superfamily domains
1.48E-04	GO:0043122	regulation of I-kappaB kinase/NF-kappaB signaling
1.58E-04	GO:0032879	regulation of localization
1.61E-04	GO:0051056	regulation of small GTPase mediated signal transduction
1.63E-04	GO:0010942	positive regulation of cell death
1.73E-04	GO:0007162	negative regulation of cell adhesion
2.03E-04	GO:0002444	myeloid leukocyte mediated immunity
2.03E-04	GO:0070664	negative regulation of leukocyte proliferation
2.11E-04	GO:0007264	small GTPase mediated signal transduction
2.12E-04	GO:0002440	production of molecular mediator of immune response
2.17E-04	GO:0001932	regulation of protein phosphorylation
2.19E-04	GO:0040017	positive regulation of locomotion
2.37E-04	GO:0006935	chemotaxis
2.37E-	GO:0042	taxis

04	330			
2.51E-04	GO:0042742	defense response to bacterium		
2.70E-04	GO:0071706	tumor necrosis factor superfamily cytokine production		
3.24E-04	GO:0007169	transmembrane receptor protein tyrosine kinase signaling pathway		
3.28E-04	GO:0042127	regulation of cell proliferation		
3.41E-04	GO:0031325	positive regulation of cellular metabolic process		
3.46E-04	GO:0043068	positive regulation of programmed cell death		
3.81E-04	GO:0051272	positive regulation of cellular component movement		
3.87E-04	GO:0030335	positive regulation of cell migration		
3.96E-04	GO:0001774	microglial cell activation		
4.34E-04	GO:0042095	interferon-gamma biosynthetic process		
4.58E-04	GO:0043901	negative regulation of multi-organism process		
4.80E-04	GO:0045637	regulation of myeloid cell differentiation		
5.01E-04	GO:0032680	regulation of tumor necrosis factor production		
5.03E-04	GO:0032940	secretion by cell		
5.52E-04	GO:2001233	regulation of apoptotic signaling pathway		
5.82E-04	GO:0051241	negative regulation of multicellular organismal process		
6.04E-04	GO:2000147	positive regulation of cell motility		
6.10E-04	GO:0046903	secretion		
6.24E-04	GO:0097530	granulocyte migration		
6.24E-04	GO:0032640	tumor necrosis factor production		
6.43E-04	GO:0043065	positive regulation of apoptotic process		
6.68E-04	GO:0010604	positive regulation of macromolecule metabolic process		
7.25E-04	GO:0031295	T cell costimulation		
7.25E-04	GO:0031294	lymphocyte costimulation		
7.38E-04	GO:0030334	regulation of cell migration		
7.67E-04	GO:0023014	signal transduction by phosphorylation		
7.68E-04	GO:0050672	negative regulation of lymphocyte proliferation		
7.68E-04	GO:0032945	negative regulation of mononuclear cell proliferation		
7.73E-04	GO:0032675	regulation of interleukin-6 production		
9.63E-04	GO:0002831	regulation of response to biotic stimulus		
9.69E-04	GO:0002285	lymphocyte activation involved in immune response		
9.98E-04	GO:0046634	regulation of alpha-beta T cell activation		
1.01E-03	GO:0044403	symbiosis, encompassing mutualism through parasitism		
1.01E-03	GO:0044419	interspecies interaction between organisms		
1.10E-03	GO:0002576	platelet degranulation		
1.12E-03	GO:0048002	antigen processing and presentation of peptide antigen		
1.23E-03	GO:1903036	positive regulation of response to wounding		
1.28E-03	GO:0045576	mast cell activation		
1.37E-03	GO:0002685	regulation of leukocyte migration		
1.40E-03	GO:0006468	protein phosphorylation		

1.44E-03	GO:0038094	Fc-gamma receptor signaling pathway
1.44E-03	GO:0032635	interleukin-6 production
1.44E-03	GO:0050729	positive regulation of inflammatory response
1.44E-03	GO:0038123	toll-like receptor TLR1:TLR2 signaling pathway
1.44E-03	GO:0038124	toll-like receptor TLR6:TLR2 signaling pathway
1.45E-03	GO:0002448	mast cell mediated immunity
1.52E-03	GO:0001818	negative regulation of cytokine production
1.71E-03	GO:0016192	vesicle-mediated transport
1.72E-03	GO:0007167	enzyme linked receptor protein signaling pathway
1.79E-03	GO:0071621	granulocyte chemotaxis
1.81E-03	GO:0043900	regulation of multi-organism process
1.84E-03	GO:0000165	MAPK cascade
1.93E-03	GO:0038095	Fc-epsilon receptor signaling pathway
2.08E-03	GO:0030041	actin filament polymerization
2.12E-03	GO:0034162	toll-like receptor 9 signaling pathway
2.17E-03	GO:2000026	regulation of multicellular organismal development
2.17E-03	GO:2001236	regulation of extrinsic apoptotic signaling pathway
2.17E-03	GO:0002478	antigen processing and presentation of exogenous peptide antigen
2.19E-03	GO:0019882	antigen processing and presentation
2.55E-03	GO:0001933	negative regulation of protein phosphorylation
2.57E-03	GO:0002706	regulation of lymphocyte mediated immunity
2.72E-03	GO:0032318	regulation of Ras GTPase activity
2.73E-03	GO:1903038	negative regulation of leukocyte cell-cell adhesion
2.89E-03	GO:2000145	regulation of cell motility
2.89E-03	GO:0043299	leukocyte degranulation
2.89E-03	GO:0032623	interleukin-2 production
3.08E-03	GO:0032479	regulation of type I interferon production
3.11E-03	GO:0018108	peptidyl-tyrosine phosphorylation
3.42E-03	GO:0040012	regulation of locomotion
3.65E-03	GO:0018212	peptidyl-tyrosine modification
3.65E-03	GO:0042326	negative regulation of phosphorylation
3.82E-03	GO:0006928	movement of cell or subcellular component
3.87E-03	GO:0019884	antigen processing and presentation of exogenous antigen
4.08E-03	GO:0002532	production of molecular mediator involved in inflammatory response
4.09E-03	GO:0034146	toll-like receptor 5 signaling pathway
4.16E-03	GO:0042325	regulation of phosphorylation
4.18E-03	GO:0032320	positive regulation of Ras GTPase activity
4.18E-03	GO:0032606	type I interferon production
4.21E-03	GO:0045597	positive regulation of cell differentiation
4.33E-03	GO:0043123	positive regulation of I-kappaB kinase/NF-kappaB signaling
4.78E-	GO:0033	cell adhesion mediated by integrin

03	627			
4.86E-03	GO:0022408	negative regulation of cell-cell adhesion		
4.89E-03	GO:0032729	positive regulation of interferon-gamma production		
4.97E-03	GO:2000379	positive regulation of reactive oxygen species metabolic process		
4.97E-03	GO:0002687	positive regulation of leukocyte migration		
5.22E-03	GO:0050921	positive regulation of chemotaxis		
5.49E-03	GO:0051270	regulation of cellular component movement		
5.83E-03	GO:0045072	regulation of interferon-gamma biosynthetic process		
6.01E-03	GO:0034111	negative regulation of homotypic cell-cell adhesion		
6.01E-03	GO:0032609	interferon-gamma production		
6.01E-03	GO:0002698	negative regulation of immune effector process		
6.37E-03	GO:0032649	regulation of interferon-gamma production		
6.55E-03	GO:0030036	actin cytoskeleton organization		
6.58E-03	GO:0051092	positive regulation of NF-kappaB transcription factor activity		
6.83E-03	GO:0045078	positive regulation of interferon-gamma biosynthetic process		
7.02E-03	GO:0048731	system development		
7.25E-03	GO:0038096	Fc-gamma receptor signaling pathway involved in phagocytosis		
7.25E-03	GO:0002433	immune response-regulating cell surface receptor signaling pathway involved in phagocytosis		
7.54E-03	GO:0008064	regulation of actin polymerization or depolymerization		
7.64E-03	GO:1902532	negative regulation of intracellular signal transduction		
7.67E-03	GO:0030593	neutrophil chemotaxis		
7.80E-03	GO:0030890	positive regulation of B cell proliferation		
7.80E-03	GO:0043303	mast cell degranulation		
7.80E-03	GO:0002279	mast cell activation involved in immune response		
7.80E-03	GO:0097028	dendritic cell differentiation		
7.83E-03	GO:0050868	negative regulation of T cell activation		
7.87E-03	GO:0045824	negative regulation of innate immune response		
8.38E-03	GO:0045936	negative regulation of phosphate metabolic process		
8.38E-03	GO:0010563	negative regulation of phosphorus metabolic process		
8.39E-03	GO:0009968	negative regulation of signal transduction		
8.47E-03	GO:0030832	regulation of actin filament length		
8.58E-03	GO:0050920	regulation of chemotaxis		
8.75E-03	GO:0008150	biological_process		
8.83E-03	GO:0001934	positive regulation of protein phosphorylation		
9.34E-03	GO:0045859	regulation of protein kinase activity		
9.49E-03	GO:0043408	regulation of MAPK cascade		
1.06E-02	GO:0032608	interferon-beta production		
1.12E-02	GO:0050855	regulation of B cell receptor signaling pathway		
1.13E-02	GO:0045577	regulation of B cell differentiation		
1.20E-02	GO:0032956	regulation of actin cytoskeleton organization		
1.21E-02	GO:0045580	regulation of T cell differentiation		

1.24E-02	GO:0007265	Ras protein signal transduction
1.28E-02	GO:0051090	regulation of sequence-specific DNA binding transcription factor activity
1.38E-02	GO:0042119	neutrophil activation
1.41E-02	GO:0002700	regulation of production of molecular mediator of immune response
1.42E-02	GO:0046631	alpha-beta T cell activation
1.49E-02	GO:1990266	neutrophil migration
1.52E-02	GO:0042327	positive regulation of phosphorylation
1.56E-02	GO:0048872	homeostasis of number of cells
1.66E-02	GO:0032321	positive regulation of Rho GTPase activity
1.67E-02	GO:0032845	negative regulation of homeostatic process
1.70E-02	GO:0045055	regulated secretory pathway
1.74E-02	GO:0071396	cellular response to lipid
1.76E-02	GO:0032494	response to peptidoglycan
1.76E-02	GO:0031342	negative regulation of cell killing
1.76E-02	GO:0001911	negative regulation of leukocyte mediated cytotoxicity
1.85E-02	GO:0006521	regulation of cellular amino acid metabolic process
1.87E-02	GO:0050854	regulation of antigen receptor-mediated signaling pathway
1.95E-02	GO:0051223	regulation of protein transport
2.01E-02	GO:0001562	response to protozoan
2.04E-02	GO:0034166	toll-like receptor 10 signaling pathway
2.11E-02	GO:0002707	negative regulation of lymphocyte mediated immunity
2.11E-02	GO:0010562	positive regulation of phosphorus metabolic process
2.11E-02	GO:0045937	positive regulation of phosphate metabolic process
2.13E-02	GO:0046578	regulation of Ras protein signal transduction
2.23E-02	GO:0002704	negative regulation of leukocyte mediated immunity
2.24E-02	GO:0002495	antigen processing and presentation of peptide antigen via MHC class II
2.44E-02	GO:0033628	regulation of cell adhesion mediated by integrin
2.49E-02	GO:0045595	regulation of cell differentiation
2.60E-02	GO:0002504	antigen processing and presentation of peptide or polysaccharide antigen via MHC class II
2.67E-02	GO:0030100	regulation of endocytosis
2.73E-02	GO:0045582	positive regulation of T cell differentiation
2.91E-02	GO:0048525	negative regulation of viral process
2.92E-02	GO:0031334	positive regulation of protein complex assembly
2.98E-02	GO:0048520	positive regulation of behavior
3.07E-02	GO:0016310	phosphorylation
3.16E-02	GO:0050848	regulation of calcium-mediated signaling
3.16E-02	GO:0032615	interleukin-12 production
3.16E-02	GO:0032663	regulation of interleukin-2 production
3.38E-02	GO:0002507	tolerance induction
3.45E-02	GO:0032481	positive regulation of type I interferon production
3.47E-	GO:0048	positive regulation of smooth muscle cell proliferation

	02	661							
	3.47E-02	GO:0002286	T cell activation involved in immune response						
	3.71E-02	GO:0050708	regulation of protein secretion						
	3.76E-02	GO:0006461	protein complex assembly						
	3.87E-02	GO:0002716	negative regulation of natural killer cell mediated immunity						
	3.87E-02	GO:0045953	negative regulation of natural killer cell mediated cytotoxicity						
	3.87E-02	GO:0002291	T cell activation via T cell receptor contact with antigen bound to MHC molecule on antigen presenting cell						
	3.89E-02	GO:0070271	protein complex biogenesis						
	4.05E-02	GO:0046635	positive regulation of alpha-beta T cell activation						
	4.05E-02	GO:0032418	lysosome localization						
	4.07E-02	GO:0048856	anatomical structure development						
	4.08E-02	GO:2000106	regulation of leukocyte apoptotic process						
	4.10E-02	GO:0045123	cellular extravasation						
	4.10E-02	GO:0030225	macrophage differentiation						
	4.12E-02	GO:0023057	negative regulation of signaling						
	4.30E-02	GO:0044767	single-organism developmental process						
	4.37E-02	GO:0008284	positive regulation of cell proliferation						
	4.39E-02	GO:0032502	developmental process						
	4.50E-02	GO:0001773	myeloid dendritic cell activation						
	4.50E-02	GO:0036230	granulocyte activation						
	4.57E-02	GO:0051050	positive regulation of transport						
	4.73E-02	GO:0010648	negative regulation of cell communication						
	4.75E-02	GO:0019220	regulation of phosphate metabolic process						
	4.75E-02	GO:0051049	regulation of transport						
	4.80E-02	GO:0071260	cellular response to mechanical stimulus						
	4.80E-02	GO:0002260	lymphocyte homeostasis						
	4.81E-02	GO:0001776	leukocyte homeostasis						
	4.94E-02	GO:0070201	regulation of establishment of protein localization						
	4.80E-03	GO:0044267	cellular protein metabolic process	1.49E-05	GO:0043231	intracellular membrane-bounded organelle	1.57E-03	GO:0016874	ligase activity
	1.40E-02	GO:0016482	cytoplasmic transport	1.52E-05	GO:0043227	membrane-bounded organelle	1.86E-02	GO:0019787	small conjugating protein transferase activity
				1.98E-05	GO:0044446	intracellular organelle part			
				7.52E-05	GO:0005622	intracellular			
				9.82E-05	GO:0044422	organelle part			
				2.44E-04	GO:0044424	intracellular part			
tan				1.19E-03	GO:0043229	intracellular organelle			
				2.30E-03	GO:0043226	organelle			
				1.23E-02	GO:0031090	organelle membrane			
				2.58E-02	GO:0070013	intracellular organelle lumen			
				4.56E-02	GO:0005737	cytoplasm			
				4.76E-02	GO:0043233	organelle lumen			
				4.98E-02	GO:0031974	membrane-enclosed lumen			

darkturquoise		/	/	/	3.29E-03	GO:0098588	bounding membrane of organelle	/	/	/
					2.20E-02	GO:0031090	organelle membrane			
					2.29E-02	GO:0042175	nuclear outer membrane-endoplasmic reticulum membrane network			
royalblue		4.14E-03	GO:0070647	protein modification by small protein conjugation or removal	6.46E-06	GO:0005737	cytoplasm	/	/	/
		1.73E-02	GO:0007005	mitochondrion organization	3.83E-05	GO:0044446	intracellular organelle part			
		4.03E-02	GO:0061024	membrane organization	2.01E-04	GO:0044422	organelle part			
					1.24E-03	GO:0005622	intracellular			
					1.73E-03	GO:0043231	intracellular membrane-bounded organelle			
					2.43E-03	GO:0031967	organelle envelope			
					2.66E-03	GO:0031975	envelope			
					3.93E-03	GO:0044424	intracellular part			
					7.29E-03	GO:0031090	organelle membrane			
					8.93E-03	GO:0043229	intracellular organelle			
					1.04E-02	GO:0044444	cytoplasmic part			
					2.02E-02	GO:0032991	macromolecular complex			
					3.77E-02	GO:0005794	Golgi apparatus			
purple		1.09E-04	GO:0044237	cellular metabolic process	5.24E-10	GO:0005622	intracellular	1.10E-02	GO:0005488	binding
		1.30E-04	GO:0008152	metabolic process	1.84E-09	GO:0044424	intracellular part	1.44E-02	GO:0019783	small conjugating protein-specific protease activity
		2.10E-04	GO:0008150	biological_process	7.87E-07	GO:0005634	nucleus	1.75E-02	GO:0005515	protein binding
		7.84E-04	GO:0070647	protein modification by small protein conjugation or removal	8.97E-07	GO:0043229	intracellular organelle	3.04E-02	GO:0004843	ubiquitin-specific protease activity
		1.19E-03	GO:0006511	ubiquitin-dependent protein catabolic process	2.72E-06	GO:0043231	intracellular membrane-bounded organelle	4.87E-02	GO:0051219	phosphoprotein binding
		1.44E-03	GO:0044265	cellular macromolecule catabolic process	1.08E-05	GO:0043226	organelle			
		1.63E-03	GO:0051603	proteolysis involved in cellular protein catabolic process	1.56E-05	GO:0043227	membrane-bounded organelle			
		1.84E-03	GO:0019941	modification-dependent protein catabolic process	4.06E-05	GO:0044446	intracellular organelle part			
		2.53E-03	GO:0043632	modification-dependent macromolecule catabolic process	5.81E-05	GO:0005737	cytoplasm			
		3.04E-03	GO:0006996	organelle organization	9.53E-05	GO:0044422	organelle part			
		3.57E-03	GO:0044260	cellular macromolecule metabolic process	1.39E-04	GO:0044464	cell part			
		4.02E-03	GO:0044257	cellular protein catabolic process	1.39E-04	GO:0005623	cell			
	CRBL	9.19E-03	GO:0044248	cellular catabolic process	2.25E-04	GO:0044428	nuclear part			
		1.40E-02	GO:0000278	mitotic cell cycle	4.09E-03	GO:1902494	catalytic complex			
		2.57E-02	GO:0071704	organic substance metabolic process	6.76E-03	GO:0005654	nucleoplasm			
		2.72E-02	GO:0044267	cellular protein metabolic process	1.30E-02	GO:0031981	nuclear lumen			
		3.23E-02	GO:0043170	macromolecule metabolic process	1.64E-02	GO:1990234	transferase complex			
		3.52E-02	GO:0009057	macromolecule catabolic process	3.06E-02	GO:0032991	macromolecular complex			
		4.01E-02	GO:0019222	regulation of metabolic process	3.20E-02	GO:0070013	intracellular organelle lumen			
		4.71E-02	GO:0070646	protein modification by small protein removal						
		4.93E-02	GO:0071840	cellular component organization or biogenesis						
turquoise		1.01E-09	GO:0051606	detection of stimulus	4.58E-10	GO:0005615	extracellular space	6.28E-17	GO:0004930	G-protein coupled receptor activity
		1.20E-09	GO:0050907	detection of chemical stimulus involved in sensory perception	7.94E-08	GO:0031012	extracellular matrix	7.74E-15	GO:0004872	receptor activity
		1.12E-08	GO:0007606	sensory perception of chemical stimulus	8.48E-08	GO:0045095	keratin filament	2.27E-11	GO:0038023	signaling receptor activity
		1.54E-	GO:0050	detection of chemical stimulus involved in sensory perception of smell	2.65E-	GO:0005	proteinaceous extracellular matrix	8.92E-	GO:0004	transmembrane signaling receptor activity

		07	911		07	578		11	888	
		1.98E-07	GO:0050906	detection of stimulus involved in sensory perception	1.66E-04	GO:0005882	intermediate filament	1.54E-07	GO:0004984	olfactory receptor activity
		5.03E-07	GO:0007600	sensory perception	4.60E-03	GO:0005581	collagen trimer	7.42E-06	GO:0004252	serine-type endopeptidase activity
		7.16E-07	GO:0009593	detection of chemical stimulus	4.29E-02	GO:0045111	intermediate filament cytoskeleton	8.71E-04	GO:0008236	serine-type peptidase activity
		7.61E-07	GO:0007586	digestion				1.27E-03	GO:0017171	serine hydrolase activity
		3.82E-06	GO:0007608	sensory perception of smell				2.40E-03	GO:0060089	molecular transducer activity
		5.48E-06	GO:0007186	G-protein coupled receptor signaling pathway				2.40E-03	GO:0004871	signal transducer activity
		1.81E-02	GO:0007588	excretion				2.81E-02	GO:0008528	G-protein coupled peptide receptor activity
		2.05E-02	GO:0022600	digestive system process				3.20E-02	GO:1901618	organic hydroxy compound transmembrane transporter activity
		2.73E-02	GO:0031424	keratinization						
		3.48E-02	GO:0009582	detection of abiotic stimulus						
		3.75E-02	GO:0030574	collagen catabolic process						
		4.10E-02	GO:0007601	visual perception						
		4.42E-02	GO:0009584	detection of visible light						
		2.60E-08	GO:0051603	proteolysis involved in cellular protein catabolic process	7.67E-09	GO:0005737	cytoplasm	1.12E-03	GO:0003824	catalytic activity
		7.05E-08	GO:0044257	cellular protein catabolic process	2.18E-07	GO:0031090	organelle membrane	1.46E-02	GO:0016787	hydrolase activity
		1.07E-07	GO:0043632	modification-dependent macromolecule catabolic process	3.83E-07	GO:0000502	proteasome complex	3.91E-02	GO:0051219	phosphoprotein binding
		2.23E-07	GO:0006511	ubiquitin-dependent protein catabolic process	4.15E-07	GO:0044444	cytoplasmic part			
		3.50E-07	GO:0019941	modification-dependent protein catabolic process	3.03E-06	GO:0005622	intracellular			
		3.61E-07	GO:0070647	protein modification by small protein conjugation or removal	6.26E-06	GO:0044424	intracellular part			
		6.04E-07	GO:0044265	cellular macromolecule catabolic process	1.40E-05	GO:0043231	intracellular membrane-bounded organelle			
		7.51E-07	GO:1901575	organic substance catabolic process	1.72E-05	GO:0044446	intracellular organelle part			
		9.31E-07	GO:0044248	cellular catabolic process	3.46E-05	GO:0044422	organelle part			
		1.66E-06	GO:0044267	cellular protein metabolic process	5.13E-04	GO:0043227	membrane-bounded organelle			
		2.80E-06	GO:0030163	protein catabolic process	8.65E-04	GO:0005829	cytosol			
		9.15E-06	GO:0009057	macromolecule catabolic process	3.01E-03	GO:0043229	intracellular organelle			
		1.35E-05	GO:0009056	catabolic process	4.66E-03	GO:0098588	bounding membrane of organelle			
grey60	MEDU	5.79E-05	GO:1903050	regulation of proteolysis involved in cellular protein catabolic process	1.11E-02	GO:0005739	mitochondrion			
		7.82E-05	GO:0016567	protein ubiquitination	1.63E-02	GO:0032991	macromolecular complex			
		1.02E-04	GO:1903362	regulation of cellular protein catabolic process	2.23E-02	GO:0043226	organelle			
		1.09E-04	GO:0032446	protein modification by small protein conjugation	2.59E-02	GO:0012505	endomembrane system			
		1.36E-04	GO:0010498	proteasomal protein catabolic process						
		2.36E-04	GO:0043161	proteasome-mediated ubiquitin-dependent protein catabolic process						
		2.93E-04	GO:2000058	regulation of protein ubiquitination involved in ubiquitin-dependent protein catabolic process						
		6.16E-04	GO:0000209	protein polyubiquitination						
		7.08E-04	GO:0019538	protein metabolic process						
		1.10E-03	GO:2000060	positive regulation of protein ubiquitination involved in ubiquitin-dependent protein catabolic process						
		1.32E-03	GO:0031329	regulation of cellular catabolic process						
		1.38E-03	GO:0042787	protein ubiquitination involved in ubiquitin-dependent protein catabolic process						
		1.91E-03	GO:0033572	transferrin transport						
		2.03E-03	GO:0051437	positive regulation of ubiquitin-protein ligase activity involved in regulation of mitotic cell cycle transition						

	2.42E-03	GO:0015682	ferric iron transport						
	2.42E-03	GO:0072512	trivalent inorganic cation transport						
	3.63E-03	GO:0051439	regulation of ubiquitin-protein ligase activity involved in mitotic cell cycle						
	3.70E-03	GO:0042176	regulation of protein catabolic process						
	3.82E-03	GO:0030177	positive regulation of Wnt signaling pathway						
	8.53E-03	GO:0009894	regulation of catabolic process						
	1.03E-02	GO:0051443	positive regulation of ubiquitin-protein transferase activity						
	1.13E-02	GO:0015031	protein transport						
	1.19E-02	GO:1903320	regulation of protein modification by small protein conjugation or removal						
	1.19E-02	GO:0051436	negative regulation of ubiquitin-protein ligase activity involved in mitotic cell cycle						
	1.43E-02	GO:0044238	primary metabolic process						
	1.64E-02	GO:0051351	positive regulation of ligase activity						
	1.76E-02	GO:0044237	cellular metabolic process						
	1.81E-02	GO:0006977	DNA damage response, signal transduction by p53 class mediator resulting in cell cycle arrest						
	2.02E-02	GO:0031396	regulation of protein ubiquitination						
	2.06E-02	GO:0072431	signal transduction involved in mitotic G1 DNA damage checkpoint						
	2.06E-02	GO:1902400	intracellular signal transduction involved in G1 DNA damage checkpoint						
	2.28E-02	GO:0031145	anaphase-promoting complex-dependent proteasomal ubiquitin-dependent protein catabolic process						
	2.37E-02	GO:1903322	positive regulation of protein modification by small protein conjugation or removal						
	2.43E-02	GO:0043412	macromolecule modification						
	2.56E-02	GO:0007005	mitochondrion organization						
	2.79E-02	GO:0044403	symbiosis, encompassing mutualism through parasitism						
	2.79E-02	GO:0044419	interspecies interaction between organisms						
	3.00E-02	GO:1903052	positive regulation of proteolysis involved in cellular protein catabolic process						
	3.02E-02	GO:1902403	signal transduction involved in mitotic DNA integrity checkpoint						
	3.02E-02	GO:0072413	signal transduction involved in mitotic cell cycle checkpoint						
	3.02E-02	GO:1902402	signal transduction involved in mitotic DNA damage checkpoint						
	3.02E-02	GO:0051352	negative regulation of ligase activity						
	3.02E-02	GO:0051444	negative regulation of ubiquitin-protein transferase activity						
	3.02E-02	GO:0002479	antigen processing and presentation of exogenous peptide antigen via MHC class I, TAP-dependent						
	3.73E-02	GO:0036211	protein modification process						
	3.73E-02	GO:0006464	cellular protein modification process						
	3.78E-02	GO:1903364	positive regulation of cellular protein catabolic process						
	3.86E-02	GO:0072422	signal transduction involved in DNA damage checkpoint						
	3.86E-02	GO:0072401	signal transduction involved in DNA integrity checkpoint						
	4.34E-02	GO:0072395	signal transduction involved in cell cycle checkpoint						
	4.77E-02	GO:1903047	mitotic cell cycle process						
	4.87E-02	GO:0006996	organelle organization						
	4.87E-02	GO:0042590	antigen processing and presentation of exogenous peptide antigen via MHC class I						
turquoise	9.31E-16	GO:0050911	detection of chemical stimulus involved in sensory perception of smell	2.55E-12	GO:0045095	keratin filament	1.59E-18	GO:0004930	G-protein coupled receptor activity
	1.56E-	GO:0050	detection of chemical stimulus involved in sensory perception	4.42E-	GO:0005	intermediate filament	9.31E-	GO:0004	olfactory receptor activity

	15	907		07	882		16	984	
	2.81E-13	GO:0007606	sensory perception of chemical stimulus	5.32E-06	GO:0005615	extracellular space	6.90E-10	GO:0004872	receptor activity
	4.42E-13	GO:0050906	detection of stimulus involved in sensory perception	5.67E-06	GO:0005578	proteinaceous extracellular matrix	7.07E-10	GO:0004888	transmembrane signaling receptor activity
	2.15E-12	GO:0051606	detection of stimulus	1.31E-05	GO:0031012	extracellular matrix	2.68E-09	GO:0038023	signaling receptor activity
	4.04E-11	GO:0007608	sensory perception of smell	1.21E-03	GO:0005581	collagen trimer	2.81E-07	GO:0004252	serine-type endopeptidase activity
	5.54E-11	GO:0009593	detection of chemical stimulus	2.06E-02	GO:0016324	apical plasma membrane	1.80E-04	GO:0008236	serine-type peptidase activity
	2.45E-09	GO:0007600	sensory perception				2.58E-04	GO:0017171	serine hydrolase activity
	2.25E-07	GO:0007586	digestion				3.87E-03	GO:0043565	sequence-specific DNA binding
	1.06E-05	GO:0007186	G-protein coupled receptor signaling pathway				1.48E-02	GO:0060089	molecular transducer activity
	2.45E-04	GO:0031424	keratinization				1.48E-02	GO:0004871	signal transducer activity
	3.34E-03	GO:0035270	endocrine system development				1.55E-02	GO:0005125	cytokine activity
	7.32E-03	GO:0021983	pituitary gland development						
	7.57E-03	GO:0042742	defense response to bacterium						
	9.66E-03	GO:0030216	keratinocyte differentiation						
	1.33E-02	GO:0008544	epidermis development						
	2.69E-02	GO:0009913	epidermal cell differentiation						
blue	2.16E-24	GO:0090304	nucleic acid metabolic process	1.08E-34	GO:0005634	nucleus	2.02E-19	GO:0003676	nucleic acid binding
	2.69E-22	GO:0043170	macromolecule metabolic process	3.47E-31	GO:0043231	intracellular membrane-bounded organelle	1.09E-11	GO:1901363	heterocyclic compound binding
	3.67E-22	GO:0044260	cellular macromolecule metabolic process	1.31E-27	GO:0043229	intracellular organelle	4.74E-11	GO:0097159	organic cyclic compound binding
	3.77E-22	GO:0006139	nucleobase-containing compound metabolic process	2.37E-26	GO:0043227	membrane-bounded organelle	9.07E-10	GO:0003677	DNA binding
	1.25E-21	GO:0044238	primary metabolic process	5.69E-26	GO:0005622	intracellular	1.48E-08	GO:0003723	RNA binding
	5.25E-21	GO:0071704	organic substance metabolic process	1.06E-25	GO:0044424	intracellular part	4.00E-08	GO:0044822	poly(A) RNA binding
	7.29E-21	GO:0046483	heterocycle metabolic process	6.89E-22	GO:0043226	organelle	2.80E-07	GO:0005488	binding
	7.51E-21	GO:1901360	organic cyclic compound metabolic process	1.69E-18	GO:0044428	nuclear part	9.51E-07	GO:0046872	metal ion binding
	7.70E-21	GO:0016070	RNA metabolic process	2.91E-17	GO:0031981	nuclear lumen	3.44E-06	GO:0043169	cation binding
	1.27E-20	GO:0008152	metabolic process	1.74E-13	GO:0005654	nucleoplasm	5.81E-04	GO:0043167	ion binding
	1.77E-20	GO:0006725	cellular aromatic compound metabolic process	7.83E-13	GO:0044446	intracellular organelle part	7.22E-04	GO:0003674	molecular_function
	1.79E-20	GO:0006807	nitrogen compound metabolic process	1.28E-11	GO:0044422	organelle part	3.20E-02	GO:0000989	transcription factor binding transcription factor activity
	3.52E-20	GO:0044237	cellular metabolic process	1.43E-11	GO:0070013	intracellular organelle lumen	4.05E-02	GO:0000988	protein binding transcription factor activity
	1.36E-19	GO:0034641	cellular nitrogen compound metabolic process	2.98E-11	GO:0031974	membrane-enclosed lumen	4.37E-02	GO:0003712	transcription cofactor activity
	2.08E-19	GO:0010467	gene expression	4.20E-11	GO:0043233	organelle lumen			
	7.39E-19	GO:0044249	cellular biosynthetic process	6.50E-10	GO:0005623	cell			
	4.18E-18	GO:1901576	organic substance biosynthetic process	6.50E-10	GO:0044464	cell part			
	2.15E-17	GO:0034645	cellular macromolecule biosynthetic process	1.10E-04	GO:0044451	nucleoplasm part			
	4.34E-17	GO:0009058	biosynthetic process	1.21E-04	GO:0005730	nucleolus			
	2.99E-16	GO:0009059	macromolecule biosynthetic process	2.32E-04	GO:0005681	spliceosomal complex			
	4.03E-15	GO:0010468	regulation of gene expression	2.13E-03	GO:0043228	non-membrane-bounded organelle			
	1.65E-14	GO:0060255	regulation of macromolecule metabolic process	2.13E-03	GO:0043232	intracellular non-membrane-bounded organelle			
	3.47E-14	GO:2000112	regulation of cellular macromolecule biosynthetic process	3.13E-03	GO:0016607	nuclear speck			
	6.02E-14	GO:0032774	RNA biosynthetic process	2.39E-02	GO:0043209	myelin sheath			
	9.49E-14	GO:0010556	regulation of macromolecule biosynthetic process	2.84E-02	GO:0005801	cis-Golgi network			

	1.98E-13	GO:0051252	regulation of RNA metabolic process	3.68E-02	GO:0005789	endoplasmic reticulum membrane	
	2.47E-13	GO:0019219	regulation of nucleobase-containing compound metabolic process	4.52E-02	GO:0042175	nuclear outer membrane-endoplasmic reticulum membrane network	
	4.38E-13	GO:0097659	nucleic acid-templated transcription				
	6.82E-13	GO:0019222	regulation of metabolic process				
	1.74E-12	GO:0031326	regulation of cellular biosynthetic process				
	2.20E-12	GO:0006351	transcription, DNA-templated				
	2.66E-12	GO:1901362	organic cyclic compound biosynthetic process				
	3.11E-12	GO:2001141	regulation of RNA biosynthetic process				
	5.36E-12	GO:0080090	regulation of primary metabolic process				
	5.75E-12	GO:1903506	regulation of nucleic acid-templated transcription				
	5.90E-12	GO:0044271	cellular nitrogen compound biosynthetic process				
	6.46E-12	GO:0019438	aromatic compound biosynthetic process				
	9.42E-12	GO:0018130	heterocycle biosynthetic process				
	1.49E-11	GO:0034654	nucleobase-containing compound biosynthetic process				
	1.57E-11	GO:0009889	regulation of biosynthetic process				
	1.91E-11	GO:0051171	regulation of nitrogen compound metabolic process				
	3.23E-11	GO:0031323	regulation of cellular metabolic process				
	3.72E-11	GO:0006355	regulation of transcription, DNA-templated				
	3.25E-10	GO:0006396	RNA processing				
	9.50E-10	GO:0016071	mRNA metabolic process				
	1.29E-09	GO:0006397	mRNA processing				
	3.09E-08	GO:0008380	RNA splicing				
	3.50E-06	GO:0000377	RNA splicing, via transesterification reactions with bulged adenosine as nucleophile				
	3.50E-06	GO:0000398	mRNA splicing, via spliceosome				
	7.82E-06	GO:0000375	RNA splicing, via transesterification reactions				
	4.92E-04	GO:0051276	chromosome organization				
	5.40E-04	GO:0008150	biological_process				
	2.88E-03	GO:0031124	mRNA 3'-end processing				
	3.04E-03	GO:0006403	RNA localization				
	4.79E-03	GO:0009987	cellular process				
	9.56E-03	GO:0006325	chromatin organization				
	1.09E-02	GO:0015931	nucleobase-containing compound transport				
	1.72E-02	GO:0050657	nucleic acid transport				
	1.72E-02	GO:0050658	RNA transport				
	1.72E-02	GO:0051236	establishment of RNA localization				
	2.74E-02	GO:0051028	mRNA transport				
	3.22E-02	GO:0031123	RNA 3'-end processing				
	4.72E-02	GO:0016568	chromatin modification				

ST 20. Transcripts interconnected with FTD-genes. Complete list of transcripts that are co-expressed with each pure FTD-gene included in the black, darkred, red and purple modules identified in FCTX. Interconnections are based on TOM values that are explained in Figures 2-5. Transcripts that are interconnected with more than one gene are highlighted in grey. Besides *MYCBP2*, *PDK3*, *KRT222*, *ATP5B*, *PSMD12* and *RHOT1* (unique interactors of *C9orf72*), and *UBQLN1* (unique interactor of *VCP*), all the remaining transcripts were at least shared across 2 of our genes of interest. Of note, *CLTC* interacted with all 4 (*C9orf72*, *VCP*, *OPTN* and *UBQLN2*), whilst *DNM1L* and *AASDHPPT* interacted with 3 (*C9orf72*, *VCP* and *UBQLN2*) spectrum FTD-genes. These genes might be novel risk factors within the spectrum of neurodegenerative disease, particularly FTD.

Modules								
Black		Darkred		Red	Purple			
MAPT	***GRN***	***HLA-DRA***	***CTSC***	***TMEM106B***	***C9orf72***	***VCP***	***OPTN***	***UBQNL2***
KHSRP	*KHSRP*	*TBXAS1*	*CYBB*	*LOC100131015*	*MYCBP2*	*DMXL2*	*YME1L1*	*AASDHPPT*
HCFC1	*TSC2*	*CYBB*	*DOCK8*	*COPB2*	*PDK3*	*WDR7*	*CLTC*	*DNM1L*
MLL2	*SF3A1*	*DOCK8*	*NCKAP1L*	*DMXL1*	*DMXL2*	*PPM1E*		*CLTC*
CREBBP		*NCKAP1L*	*FYB*	*TTC37*	*WDR7*	*ATL1*		
SRCAP				*SERINC1*	*KRT222*	*ITFG1*		
PRR12				*NRD1*	*ATP5B*	*CLTC*		
SEC16A					*PSMD12*	*DNM1L*		
ATXN2L					*PPM1E*	*AASDHPPT*		
TSC2					*CLTC*	*UBQLN1*		
KIAA0664					*DNM1L*	*YME1L1*		
CHERP					*RHOT1*	*CAND1*		
					ATL1			
					ITFG1			
					AASDHPPT			
					CAND1			

ST 21. Protein-protein interactors (PPI) for pure (a) and spectrum (b) FTD-genes. For each of the pure FTD-genes the protein-protein interaction (PPI) has been researched and obtained from the Biogrid database and that of the IMEx consortium (output for the latter was from IntAct and Mint). For each gene of interest: 1) the interactors which overlap with WGCNA data are bolded and italicised; 2) each protein that interacts with more than one FTD-gene is hallmarked by a border, and; 3) each protein identified by more than one databse are highlighted in grey.

(a)

MAPT			GRN			CTSC			HLA-DRA			TMEM106B		
**Biogrid**	**IntAct**	**Mint**	**Biogrid**	**IntAct**	**Mint**	**Biogrid**	**IntAct**	**Mint**	**Biogrid**	**IntAct**	**Mint**	**Biogrid**	**IntAct**	**Mint**
AATF	LRRK2	PASK	ABI2	ATXN7	GLRX3	ADSL	/	CST7	ANXA11	HLA-DRB1	***CD74***	BTNL8	C6orf201	/
ABL1	YWHAZ	PIN1P1	ARFGAP1	CACNA1A	TNFRSF1B	ANKMY2			ATP1B1	***HLA-DMA***		UBC		
AKT1	***MARK2***	RPS6KB1	***ATN1***	***CRKL***	TNFRSF1A	ARIH1			CD63					
APOE	P4HB	GSK3B	ATXN7	CFTR	SORT1	ARMC9			CD82					
APP	PRNP	CDK5	CACNA1A	***ATN1***		CAPN1			CELSR1					
ARMC1	GSK3B	SGK1	CACNG5	GFI1B		CD81			CHST12					
BAG1	EGFR	RPS6KA3	CCDC8	SIRT3		FBXO6			CNTNAP3					
CALM1	HSP90AB1	***AKT1***	CCDC33	HOXA1		NEDD8			DCBLD2					
CAMK2A	BIN1	RPS6KA5	CCNG1	FAM131C		SUMO1			DDX19B					
CAPN2	***MARK4***	RPS6KA1	CCNT1	RPS6KA1		UBA2			EIF2AK3					
CDK2	MARK1	SLC1A2	CD68	PIK3R2		UBC			EPHA3					
CDK5R1	MARK3	YWHAZ	CDK2	PRKAB2		XRN2			EPHB4					
CDK5		DCTN1	CDK9	YY1					***HLA-DMB***					
CSNK1A1		SNCA	CELA2B	TGM2					HLA-DRB1					
CSNK1D		HSPA1A	CFTR	RAC1					HLA-DRB5					
CSNK2A1		FYN	CLEC4M	FRAT1					HSP90AA1					
DCTN1		TTLL6	COX6B1	CCNG1					HSP90AB1					
DGUOK			***CRKL***	CRY1					HSPA8					
DNAAF2			CRY1	KRT18					IMPAD1					
EGFR			CTSO	POT1					KIAA0922					
EP300			CUL7	FANCL					KLK4					
FKBP4			DLK1	***SGTA***					LNPEP					
FYN			DLX2	GLRX3					LRIG1					
GNPNAT1			EED	DLX2					MAN2A2					
GSK3A			EGFL7	OTX1					MARCH1					
GSK3B			EGFR	KRTAP10-7					MBP					
HAX1			FAM131C	KRTAP26-1					MS4A1					
HDAC6			FAM207A	ARFGAP1					NETO2					
HSPA1A			FANCL	NLK					PKM2					
HSPA4			FBXO6						PLXNA1					
HSPA8			FLYWCH2						POMT2					
LIMS1			FRAT1						SCARB1					
LRRK2			GFI1B						STIM1					

MAPK8	GLRX3		TBC1D22B	
MAPK11	GNB2		TCTN2	
MAPK13	HECW2		TMEM97	
MARCH7	HOXA1		TMEM206	
MARK1	KRT18		UBC	
MARK2	KRTAP10-7		YWHAE	
MARK3	KRTAP26-1			
MARK4	NAPSA			
NME2	NLK			
NUB1	NPM1			
NUFIP1	NXF1			
OGT	OTUD5			
PARK2	OTX1			
PEG10	PIK3R2			
PIN1	PKP2			
PKN1	POT1			
PPP2R4	PRKAB2			
PPP5C	RAC1			
PRKACA	RAPGEF6			
PRKAR1A	RPS6KA1			
PSEN1	SAV1			
PSMC2	***SGTA***			
PTK2B	SIRT3			
RPS6KA1	SLPI			
RPS6KA3	SMAD9			
RPS6KA5	SPACA4			
RPS6KB1	TAT			
S100B	TGM2			
SGK1	***TLE3***			
SIRT1	TOP3B			
SLC1A2	TRIB3			
SNCA	UPK1A			
SQSTM1	VHL			
SRC	YY1			
STUB1	ZC3HC1			
STXBP1	ZFP41			
SYK	ZNF408			
TRAF6	ZNF517			
TUBA1A				
TUBA1B				
TUBA4A				
TUBB3				

UBASH3B				
UBC				
UBE2D2				
UBLCP1				
YWHAB				
YWHAQ				
YWHAZ				

(b)

C9orf72			VCP			UBQLN2			OPTN		
Biogrid	IntAct	Mint	Biogrid	IntAct	Mint	Biogrid	IntAct	Mint	Biogrid	IntAct	Mint
APP	RB1CC1	***EIF2B2***	ABCC1	FAF1	***ATXN3***	AARSD1	ATXN7	ADRM1	AIMP1	***CCDC53***	FAM189A2
CRX	CRX		ABCC3	FAF2	NGLY1	ADSL	UBQLN4	PSMD4	BRE	CENPB	***CCDC53***
EIF2B2	REL		***ACACA***	UBXN7	***NPLOC4***	ATXN7	CD93	SHFM1	CALM1	EBNA1BP2	CDC23
ELAVL1	NMI		ACTB		***NSFL1C***	BCL2L11	C1QA		***CCDC53***	EIF4G2	***LNX2***
MMS19	***EIF2B2***		ACTN1		SYVN1	BID			CDC23	***FAM175B***	***TBK1***
NMI	SRPK1		ACTN4		UBE4B	C1QA			CDKN1A	FAM178A	TNIP1
REL			ADCK3		UFD1L	C12ORF10			***CYLD***	GNAS	
SRPK1			ADD1			CALU			DAZAP2	HSPA9	
			ADRB2			CD93			EIF5A2	MACF1	
			AKT1			***CUL5***			EPS15	NDUFAF2	
			ALDH18A1			***DNAJB1***			FAM178A	PPARG	
			ALS2			EDEM1			***GABARAPL1***	RPL37A	
			AMFR			EEF1B2			GABARAPL2	***RTN3***	
			ANAPC7			***ELAVL1***			***GABARAP***	SYNGAP1	
			ANKHD1			EMC3			GNAS	TBC1D17	
			ANKLE2			EMC7			GRM1	UQCRQ	
			ANKZF1			EMC8			HACE1	WDR20	
			ANXA2			EPN1			HSPB1	ZMAT2	
			ANXA5			EPN2			HTT		
			ANXA7			EPS15			***KCTD17***		
			AP1B1			FAF2			MAP1LC3A		
			AP2A2			FBXO25			MAP1LC3B		
			APOA1			HERC3			MPP1		
			APOB			HERC6			MPP6		
			APPL1			HERPUD1			MYO6		
			ARF6			***HSPA13***			PARK2		
			ARFGAP2			***IGBP1***			PIAS4		
			ARFGEF2			INSIG2			RAB3IL1		
			ARHGAP17			MARCKS			RAB8A		

	ARHGEF2	***MMGT1***	RAB10
	ARIH1	***NPLOC4***	***RAB11A***
	ARIH2	NTRK1	RAB11B
	ARPC2	P4HB	***RAB12***
	ARRB2	PDE12	***RAB14***
	AR	PFDN2	RAB25
	ASPSCR1	PLAUR	RIPK1
	ATAD3A	PRKACA	RNF11
	ATAD3B	PRPF40A	***SOD1***
	ATG5	PSMA2	SQSTM1
	ATG9A	PSMA6	STX12
	ATXN1	PSMC1	TAX1BP1
	ATXN3	PSMC2	TBC1D15
	ATXN7	RAD23A	TBC1D17
	ATXN10	***RAD23B***	***TBK1***
	AUP1	RNASEH2B	TFRC
	AVPR2	RNF11	TNFRSF1A
	BAD	***RNF185***	TNF
	BAG2	RNH1	TNIP1
	BAG6	RPA2	TRADD
	BAIAP2L1	***SEC23A***	TRAF3
	BAX	SHFM1	TRIM16
	BCCIP	SLC17A2	UBC
	BCLAF1	SNAP23	UQCRQ
	BID	SSSCA1	WDR20
	BLM	***STAM***	***YWHAB***
	BRAT1	TARDBP	YWHAG
	BRSK2	TP53	YWHAH
	BSG	***TPD52L2***	YWHAQ
	BTRC	TTC1	YWHAZ
	BZW2	UBA2	***ZDHHC17***
	C3ORF17	***UBE2A***	ZMYM6
	CAAP1	***UBE3A***	***ZNF329***
	CAB39	UBL7	ZNF384
	CACNA1C	***UBQLN1***	ZNF398
	CALR	UBQLN4	ZNF670
	CALU	***USP9X***	
	CANX	USP34	
	CASP7	***VBP1***	
	CASP9	WDR4	
	CASR	YAP1	

	CAV1	ZFYVE19
	CCDC8	ZMYM3
	CCDC132	ZRANB2
	CCDC134	
	CCNB1	
	CCT2	
	CCT3	
	CCT4	
	CCT5	
	CCT6A	
	CCT7	
	CCT8	
	CD3D	
	CD4	
	CDC25A	
	CDC27	
	CDC37	
	CDC42EP1	
	CDC42EP4	
	CDK1	
	CDK2AP1	
	CDK2	
	CDK4	
	CDKN1A	
	CDKN1B	
	CDKN2AIP	
	CENPH	
	CEP19	
	CEP55	
	CFTR	
	CHEK1	
	CHEK2	
	CIDEC	
	CLASP1	
	CLGN	
	CLN6	
	CLTA	
	CNOT2	
	CNOT8	
	CNOT10	
	COG4	
	COG5	

	COIL		
	COMMD1		
	COMMD6		
	COMT		
	COPE		
	COPS3		
	COPS5		
	COPS7A		
	CRMP1		
	CSK		
	CSNK2B		
	CTNNA1		
	CTNNBL1		
	CTNND1		
	CUL1		
	CUL2		
	CUL3		
	CUL4A		
	CUL5		
	CUL7		
	DAP3		
	DCAF7		
	DCAF11		
	DDIAS		
	DDX54		
	DERL1		
	DERL2		
	DGCR6		
	DGCR14		
	DIAPH3		
	DIO2		
	DNAJB9		
	DNAJB11		
	DNAJC10		
	DNM3		
	DOCK7		
	DSP		
	DSTN		
	DTNB		
	DUSP9		
	DYNC1I2		
	DYNC1LI1		

	EEA1		
	EEF1A2		
	EIF4A2		
	EIF5A		
	ELAVL1		
	EPPK1		
	ESPL1		
	ESR1		
	EZH2		
	F7		
	FAF1		
	FAF2		
	FAM104A		
	FAM189B		
	FANCI		
	FBF1		
	FBXO2		
	FBXO6		
	FBXW11		
	FCHSD2		
	FERMT2		
	FHOD1		
	FKBP15		
	FLNB		
	FN1		
	FUS		
	G3BP2		
	GBAS		
	GBF1		
	GET4		
	GGA1		
	GGA2		
	GIGYF2		
	GLB1		
	GOLPH3L		
	GRIN2D		
	GRWD1		
	GTF3C1		
	GTF3C3		
	GTF3C5		
	GZMK		
	H2AFJ		

	H2AFV		
	HAUS1		
	HDAC5		
	HDLBP		
	HEATR1		
	HELLS		
	HERPUD1		
	HIF1A		
	HIP1R		
	HLA-A		
	HLA-B		
	HLA-DRB1		
	HMGCR		
	HNF1A		
	HNRNPA1		
	HNRNPH2		
	HNRNPH3		
	HNRNPK		
	HOOK1		
	HS1BP3		
	HSBP1		
	HSP90AA1		
	HSP90AB1		
	HSP90B1		
	HSP90B2P		
	HSPA1A		
	HSPA4		
	HSPA5		
	HSPA8		
	HSPB1		
	HSPD1		
	HSPE1		
	HTT		
	HUWE1		
	ICK		
	IKBKE		
	INF2		
	INSIG1		
	INSIG2		
	IPO4		
	IQCB1		
	IQGAP1		

	IRS4		
	ISG15		
	ISYNA1		
	ITGA4		
	ITGB1		
	ITPR1		
	KCMF1		
	KDM3B		
	KDSR		
	KIAA1279		
	KIAA1468		
	KIAA1524		
	L3MBTL1		
	LMAN1		
	LMNA		
	LMNB2		
	LNX1		
	LRBA		
	LRIG1		
	LYAR		
	MAP1LC3A		
	MAP2K1		
	MAP7D3		
	MAPK1		
	MAPK3		
	MAPK8IP2		
	MAPK13		
	MARK2		
	MCC		
	MDM2		
	MDN1		
	MRPS18B		
	MRPS23		
	MSH4		
	MTOR		
	MUS81		
	NACA2		
	NAPA		
	NASP		
	NBEA		
	NCAPH		
	NCDN		

	NCOA1		
	NDRG1		
	NEK2		
	NF1		
	NFKB2		
	NFKBIA		
	NGLY1		
	NIPSNAP1		
	NMD3		
	NME2		
	NOS2		
	NPLOC4		
	NPM1		
	NSFL1C		
	NSF		
	NTRK1		
	NUB1		
	NUMA1		
	NUP54		
	NUP62		
	NUP107		
	NUP205		
	NUPL1		
	OBSL1		
	OS9		
	OTULIN		
	P4HB		
	PACRG		
	PARK2		
	PDCD4		
	PDCD6		
	PDCD10		
	PDXDC1		
	PEX19		
	PHB		
	PHKG2		
	PIK3C2B		
	PIK3R2		
	PKM		
	PKN2		
	PLAA		
	PLEC		

	PLK1		
	PNO1		
	POLR2B		
	POLR3C		
	PPFIBP1		
	PPM1B		
	PPP1CC		
	PPP2CA		
	PPP2CB		
	PPP2R1A		
	PPP3CA		
	PPP6C		
	PPT1		
	PRKAA1		
	PRKAR2A		
	PRKCD		
	PRMT3		
	PRMT5		
	PRPF4		
	PRPF19		
	PRPF31		
	PSMA1		
	PSMA2		
	PSMA3		
	PSMA4		
	PSMA6		
	PSMA7		
	PSMC1		
	PSMC4		
	PSMD4		
	PTCRA		
	PTGES3		
	PTGS2		
	PTPN9		
	PTPN22		
	PTPN23		
	PTPRO		
	RAB3GAP1		
	RAB3GAP2		
	RAB7A		
	RAB10		

	RAB11B	
	RAB14	
	RABGAP1	
	RAD23A	
	RAF1	
	RANBP2	
	RBBP4	
	RBFOX2	
	RBM23	
	RCN1	
	RDX	
	RFC3	
	RFC5	
	RHBDL3	
	RIF1	
	RNF2	
	RNF7	
	RNF8	
	RNF19A	
	RNF31	
	RNF103	
	RNF126	
	RPL6	
	RPL9	
	RPL13	
	RPL18A	
	RPL22	
	RPL23	
	RPL24	
	RPL30	
	RPN1	
	RPN2	
	RPS3A	
	RPS3	
	RPS4X	
	RPS6KA1	
	RPS6	
	RPS8	
	RPS9	
	RPS11	
	RPS13	
	RPS25	

	RQCD1		
	RRBP1		
	RRP12		
	RSU1		
	RUVBL2		
	SAP18		
	SCD		
	SCFD1		
	SDCCAG3		
	SEC16A		
	SEC22B		
	SERPINA1		
	SESN2		
	SFTPC		
	SGK1		
	SH2D2A		
	SHFM1		
	SIK2		
	SIRT7		
	SKP1		
	SLC3A2		
	SLC17A2		
	SLC25A3		
	SLIRP		
	SLX4		
	SMARCA5		
	SMARCC1		
	SMURF1		
	SNX3		
	SON		
	SPAST		
	SPC24		
	SPRTN		
	SPTAN1		
	SRRM2		
	SRSF3		
	SRSF11		
	ST13		
	STAG2		
	STAM2		
	STAT1		
	STIP1		

	STMN1		
	STUB1		
	SUMO1		
	SUMO2		
	SUPT6H		
	SUPT16H		
	SUZ12		
	SVIP		
	SYMPK		
	SYVN1		
	TAF6L		
	TARDBP		
	TAX1BP1		
	TBC1D9B		
	TBC1D10B		
	TCP1		
	TDP1		
	TELO2		
	TERF2		
	TIMM44		
	TKT		
	TMED10		
	TMEM33		
	TMEM129		
	TMPRSS13		
	TNKS1BP1		
	TNPO3		
	TOM1L1		
	TOM1		
	TOMM34		
	TOP1		
	TP53BP1		
	TP53		
	TP63		
	TPD52L2		
	TPM3P4		
	TRAF6		
	TRA		
	TRIM13		
	TRIM21		
	TRIM25		
	TRIP12		

	TSG101		
	TTC4		
	TTK		
	TUBA1C		
	TUBB2B		
	TUBB3		
	TUBB4B		
	TUBB		
	TUBGCP2		
	TXN2		
	TXNDC5		
	UBA5		
	UBB		
	UBC		
	UBE2J1		
	UBE2M		
	UBE2S		
	UBE4B		
	UBL4A		
	UBL7		
	UBOX5		
	UBQLN1		
	UBR4		
	UBR5		
	UBXN1		
	UBXN2A		
	UBXN2B		
	UBXN4		
	UBXN6		
	UBXN7		
	UBXN8		
	UBXN10		
	UBXN11		
	UFD1L		
	ULK3		
	USP10		
	USP13		
	VAPA		
	VAPB		
	VBP1		
	VCAM1		
	VCL		

	VCPIP1	
	VCPKMT	
	VIL1	
	VIMP	
	VIM	
	VPS53	
	WAC	
	WAPAL	
	WBSCR22	
	WDR43	
	WDR82	
	WDYHV1	
	WNK1	
	WRAP73	
	WRNIP1	
	WRN	
	YOD1	
	YWHAB	
	YWHAE	
	YWHAG	
	YWHAH	
	YWHAQ	
	YWHAZ	
	ZFAND2B	
	ZFR	
	ZNF326	
